# Heavy-flavour and quarkonium production in the LHC era: from proton–proton to heavy-ion collisions

**DOI:** 10.1140/epjc/s10052-015-3819-5

**Published:** 2016-02-29

**Authors:** A. Andronic, F. Arleo, R. Arnaldi, A. Beraudo, E. Bruna, D. Caffarri, Z. Conesa del Valle, J. G. Contreras, T. Dahms, A. Dainese, M. Djordjevic, E. G. Ferreiro, H. Fujii, P.-B. Gossiaux, R. Granier de Cassagnac, C. Hadjidakis, M. He, H. van Hees, W. A. Horowitz, R. Kolevatov, B. Z. Kopeliovich, J.-P. Lansberg, M. P. Lombardo, C. Lourenço, G. Martinez-Garcia, L. Massacrier, C. Mironov, A. Mischke, M. Nahrgang, M. Nguyen, J. Nystrand, S. Peigné, S. Porteboeuf-Houssais, I. K. Potashnikova, A. Rakotozafindrabe, R. Rapp, P. Robbe, M. Rosati, P. Rosnet, H. Satz, R. Schicker, I. Schienbein, I. Schmidt, E. Scomparin, R. Sharma, J. Stachel, D. Stocco, M. Strickland, R. Tieulent, B. A. Trzeciak, J. Uphoff, I. Vitev, R. Vogt, K. Watanabe, H. Woehri, P. Zhuang

**Affiliations:** Research Division, ExtreMe Matter Institute (EMMI), GSI Helmholzzentrum für Schwerionenforschung, Darmstadt, Germany; Laboratoire Leprince-Ringuet, Ecole Polytechnique, CNRS/IN2P3, Université Paris–Saclay, Palaiseau, France; Laboratoire d’Annecy-le-Vieux de Physique Théorique (LAPTh), Université de Savoie, CNRS, Annecy-le-Vieux, France; Sezione di Torino, INFN, Turin, Italy; European Organization for Nuclear Research (CERN), Geneva, Switzerland; IPNO, Univ. Paris-Sud, CNRS/IN2P3, Université Paris–Saclay, 91406 Orsay Cedex, France; Faculty of Nuclear Sciences and Physical Engineering, Czech Technical University in Prague, Prague, Czech Republic; Excellence Cluster Universe, Technische Universität München, Munich, Germany; Sezione di Padova, INFN, Padua, Italy; Institute of Physics Belgrade, University of Belgrade, Belgrade, Serbia; Departamento de Física de Partículas, IGFAE, Universidad de Santiago de Compostela, Santiago de Compostela, Spain; Institute of Physics, University of Tokyo, Tokyo, Japan; SUBATECH, Ecole des Mines de Nantes, Université de Nantes, CNRS-IN2P3, Nantes, France; Department of Applied Physics, Nanjing University of Science and Technology, Nanjing, China; FIAS, Institute for Theoretical Physics, Frankfurt, Germany; Department of Physics, University of Cape Town, Cape Town, South Africa; Department of High Energy Physics, Saint-Petersburg State University, Ulyanovskaya 1, Saint Petersburg, Russia; Departamento de Física, Centro Científico-Tecnológico de Valparaíso, Universidad Técnica Federico Santa María, Valparaiso, Chile; INFN, Laboratori Nazionali di Frascati, Frascati, Italy; LAL, Univ. Paris-Sud, CNRS/IN2P3, Université Paris–Saclay, Orsay, France; Faculty of Science, Institute for Subatomic Physics, Utrecht University, Utrecht, The Netherlands; National Institute for Subatomic Physics, Amsterdam, The Netherlands; Department of Physics, Duke University, Durham, USA; Department of Physics and Technology, University of Bergen, Bergen, Norway; Laboratoire de Physique Corpusculaire (LPC), Université Clermont Auvergne, Université Blaise Pascal, CNRS/IN2P3, Clermont-Ferrand, France; IRFU/SPhN, CEA Saclay, 91191 Gif-sur-Yvette Cedex, France; Department of Physics and Astronomy, Cyclotron Institute, Texas A&M University, College Station, USA; Iowa State University, Ames, USA; Fakultät für Physik, Universität Bielefeld, Bielefeld, Germany; Physikalisches Institut, Ruprecht-Karls-Universität Heidelberg, Heidelberg, Germany; Laboratoire de Physique Subatomique et de Cosmologie, Université Grenoble-Alpes, CNRS/IN2P3, Grenoble, France; Department of Theoretical Physics, Tata Institute of Fundamental Research, Mumbai, India; Department of Physics, Kent State University, Kent, USA; IPN-Lyon, Université de Lyon, Université Lyon 1, CNRS/IN2P3, Villeurbanne, France; Institut für Theoretische Physik, Johann Wolfgang Goethe-Universität, Frankfurt am Main, Germany; Theoretical Division, Los Alamos National Laboratory, Los Alamos, USA; Physics Division, Lawrence Livermore National Laboratory, Livermore, USA; Physics Department, University of California, Davis, USA; Institute of Physics, University of Tokyo, Tokyo, Japan; Key Laboratory of Quark and Lepton Physics (MOE), Institute of Particle Physics, Central China Normal University, Wuhan, China; Physics Department, Collaborative Innovation Center of Quantum Matter, Tsinghua University, Beijing, China

## Abstract

This report reviews the study of open heavy-flavour and quarkonium production in high-energy hadronic collisions, as tools to investigate fundamental aspects of Quantum Chromodynamics, from the proton and nucleus structure at high energy to deconfinement and the properties of the Quark–Gluon Plasma. Emphasis is given to the lessons learnt from LHC Run 1 results, which are reviewed in a global picture with the results from SPS and RHIC at lower energies, as well as to the questions to be addressed in the future. The report covers heavy flavour and quarkonium production in proton–proton, proton–nucleus and nucleus–nucleus collisions. This includes discussion of the effects of hot and cold strongly interacting matter, quarkonium photoproduction in nucleus–nucleus collisions and perspectives on the study of heavy flavour and quarkonium with upgrades of existing experiments and new experiments. The report results from the activity of the SaporeGravis network of the I3 Hadron Physics programme of the European Union 7$\mathrm{th}$ Framework Programme.

## Introduction

Heavy-flavour hadrons, containing open or hidden charm and beauty flavour, are among the most important tools for the study of Quantum Chromodynamics (QCD) in high-energy hadronic collisions, from the production mechanisms in proton–proton collisions (pp) and their modification in proton–nucleus collisions (p–A) to the investigation of the properties of the hot and dense strongly interacting Quark–Gluon Plasma (QGP) in nucleus–nucleus collisions (AA).

Heavy-flavour production in pp collisions provides important tests of our understanding of various aspects of QCD. The heavy-quark mass acts as a long distance cut-off so that the partonic hard-scattering process can be calculated in the framework of perturbative QCD down to low transverse momenta ($p_{\mathrm {T}}$). When the heavy-quark pair forms a quarkonium bound state, this process is non-perturbative as it involves long distances and soft momentum scales. Therefore, the detailed study of heavy-flavour production and the comparison to experimental data provides an important testing ground for both perturbative and non-perturbative aspects of QCD calculations.

In nucleus–nucleus collisions, open and hidden heavy-flavour production constitutes a sensitive probe of the hot strongly interacting medium, because hard-scattering processes take place in the early stage of the collision on a time scale that is in general shorter than the QGP thermalisation time. Disentangling the medium-induced effects and relating them to its properties requires an accurate study of the so-called cold nuclear matter (CNM) effects, which modify the production of heavy quarks in nuclear collisions with respect to proton–proton collisions. CNM effects, which can be measured in proton–nucleus interactions, include: the modification of the effective partonic luminosity in nuclei (which can be described using nuclear-modified parton densities), due to saturation of the parton kinematics phase space; the multiple scattering of partons in the nucleus before and after the hard scattering; the absorption or break-up of quarkonium states, and the interaction with other particles produced in the collision (denoted as comovers).

The nuclear modification of the parton distribution functions can also be studied, in a very clean environment, using quarkonium photoproduction in ultra-peripheral nucleus–nucleus collisions, in which a photon from the coherent electromagnetic field of an accelerated nucleus interacts with the coherent gluon field of the other nucleus or with the gluon field of a single nucleon in the other nucleus.

During their propagation through the QGP produced in high-energy nucleus–nucleus collisions, heavy quarks interact with the constituents of this medium and lose a part of their momentum, thus being able to reveal some of the QGP properties. QCD energy loss is expected to occur via both inelastic (radiative energy loss, via medium-induced gluon radiation) and elastic (collisional energy loss) processes. Energy loss is expected to depend on the parton colour-charge and mass. Therefore, charm and beauty quarks provide important tools to investigate the energy-loss mechanisms, in addition to the QGP properties. Furthermore, low-$p_{\mathrm {T}}$ heavy quarks could participate, through their interactions with the medium, in the collective expansion of the system and possibly reach thermal equilibrium with its constituents.

In nucleus–nucleus collisions, quarkonium production is expected to be significantly suppressed as a consequence of the colour screening of the force that binds the ${c\overline{c}}$ (${b\overline{b}}$) state. In this scenario, quarkonium suppression should occur sequentially, according to the binding energy of each state. As a consequence, the in-medium dissociation probability of these states are expected to provide an estimate of the initial temperature reached in the collisions. At high centre-of-mass energy, a new production mechanism could be at work in the case of charmonium: the abundance of *c* and $\overline{c}$ quarks might lead to charmonium production by (re)combination of these quarks. An observation of the recombination of heavy quarks would therefore directly point to the existence of a deconfined QGP.

The first run of the Large Hadron Collider (LHC), from 2009 to 2013, has provided a wealth of measurements in pp collisions with unprecedented centre-of-mass energies $\sqrt{s}$ from 2.76 to 8 TeV, in p–Pb collisions at $\sqrt{s_{\mathrm{NN}}} =5.02$ TeV per nucleon–nucleon interaction, in Pb–Pb collisions at $\sqrt{s_{\mathrm{NN}}} = 2.76$ TeV, as well as in photon-induced collisions. In the case of heavy-ion collisions, with respect to the experimental programmes at SPS and RHIC, the LHC programme has not only extended by more than one order of magnitude the range of explored collision energies, but it has also largely enriched the studies of heavy-flavour production, with a multitude of new observables and improved precision. Both these aspects were made possible by the energy increase, on the one hand, and by the excellent performance of the LHC and the experiments, on the other hand.

This report results from the activity of the SaporeGravis network^1^ of the I3 Hadron Physics programme of the European Union $7\mathrm{th}$ FP. The network was structured in working groups, that are reflected in the structure of this review, and it focussed on supporting and strengthening the interactions between the experimental and theoretical communities. This goal was, in particular, pursued by organising two large workshops, in Nantes (France)^2^ in December 2013 and in Padova (Italy)^3^ in December 2014.

The report is structured in eight sections. Sections 2, 3, 4, 5 and 6 review, respectively: heavy-flavour and quarkonium production in proton–proton collisions, the cold nuclear matter effects on heavy-flavour and quarkonium production in proton–nucleus collisions, the QGP effects on open heavy-flavour production in nucleus–nucleus collisions, the QGP effects on quarkonium production in nucleus–nucleus collisions, and the production of charmonium in photon-induced collisions. Sect. 7 presents an outlook of future heavy-flavour studies with the LHC and RHIC detector upgrades and with new experiments. A short summary concludes the report in Sect. 8.

## Heavy flavour and quarkonium production in proton–proton collisions

### Production mechanisms of open and hidden heavy-flavour in proton–proton collisions

#### Open-heavy-flavour production

Open-heavy-flavour production in hadronic collisions provides important tests of our understanding of various aspects of Quantum Chromodynamics (QCD). First of all, the heavy-quark mass ($m_Q$) acts as a long distance cut-off so that this process can be calculated in the framework of perturbative QCD down to low $p_{\mathrm {T}}$ and it is possible to compute the total cross section by integrating over $p_{\mathrm {T}}$. Second, the presence of multiple hard scales ($m_Q$, $p_{\mathrm {T}}$) allows us to study the perturbation series in different kinematic regions ($p_{\mathrm {T}} < m_Q$, $p_{\mathrm {T}} \sim m_Q$, $p_{\mathrm {T}} \gg m_Q$). Multiple hard scales are also present in other collider processes of high interest such as weak boson production, Higgs boson production and many cases of physics Beyond the Standard Model. Therefore, the detailed study of heavy-flavour production and the comparison to experimental data provides an important testing ground for the theoretical ideas that deal with this class of problems.

On the phenomenological side, the (differential) cross section for open-heavy-flavour production is sensitive to the gluon and the heavy-quark content in the nucleon, so that LHC data in $\mathrm pp$ and p–Pb collisions can provide valuable constraints on these parton-distribution functions (PDFs) inside the proton and the lead nucleus, respectively. In addition, these cross sections in $\mathrm pp$ and p–A collisions establish the baseline for the study of heavy-quark production in heavy-ion collisions. This aspect is a central point in heavy-ion physics since the suppression of heavy quarks at large $p_{\mathrm {T}}$ is an important signal of the QGP (see Sect. 4). Finally, let us also mention that a solid understanding of open-charm production is needed in cosmic-ray and neutrino astrophysics [1]. In the following, we will focus on $\mathrm pp$ collisions and review the different theoretical approaches to open-heavy-flavour production.

*Fixed-Flavour-Number Scheme* Conceptually, the simplest scheme is the Fixed-Flavour-Number Scheme (FFNS) where the heavy quark is not an active parton in the proton. Relying on a factorisation theorem, the differential cross section for the inclusive production of a heavy quark *Q* can be calculated as follows:

1 $$\begin{aligned}&\mathrm {d}\sigma ^{Q+X}[s,p_{\mathrm {T}},y, m_Q]\nonumber \\&\quad \simeq \sum _{i,j} \int _0^1 \mathrm {d}x_i \int _0^1 dx_j\, f^A_i(x_i,\mu _F) f^B_j(x_j,\mu _F) \nonumber \\&\qquad \times \mathrm {d}{\tilde{\sigma }}_{ij\rightarrow Q+X}[x_i,x_j,s,p_{\mathrm {T}},y,m_Q,\mu _F,\mu _R], \end{aligned}$$

or in short

2 $$\begin{aligned} \mathrm {d}\sigma ^{Q+X} \simeq \sum _{i,j} f^A_i \otimes f^B_j \otimes \mathrm {d}{\tilde{\sigma }}_{ij\rightarrow Q+X}, \end{aligned}$$

where $p_{\mathrm {T}}$ and *y* are the transverse momentum and the rapidity of the heavy quark and *s* is the square of the hadron centre-of-mass energy. The PDFs $f_i^A$ ($f_j^B$) give the number density of the parton of flavour ‘*i*’ (‘*j*’) inside the hadron ‘*A*’ (‘*B*’) carrying a fraction $x_i$ ($x_j$) of the hadron momentum at the factorisation scale $\mu _F$. Furthermore, the short-distance cross section $\mathrm {d}{\tilde{\sigma }}$ is the partonic cross section from which the so-called mass singularities or collinear singularities associated to the light quarks and the gluon have been removed via the mass-factorisation procedure and which therefore also depends on $\mu _F$. The partonic cross section also depends on the strong-coupling constant $\alpha _s$, which is evaluated at the renormalisation scale $\mu _R$. As a remainder of this procedure, the short-distance cross section will depend on logarithms of the ratio of $\mu _F$ with the hard scale. In order to avoid large logarithmic contributions, the factorisation scale $\mu _F$ should be chosen in the vicinity of the hard scale. Also the renormalisation scale $\mu _R$ is determined by the hard scale. The tilde is used to indicate that the finite collinear logarithms of the heavy-quark mass present in the partonic cross section have not been removed by the mass-factorisation procedure. These logarithms are therefore not resummed to all orders in the FFNS but are accounted for in Fixed-Order (FO) perturbation theory. The error of the approximation in Eq. (1) is suppressed by an inverse power of the hard scale which is set by the mass or the transverse momentum of the heavy quark, i.e. it is on the order of $\mathcal{O}((\Lambda /\mu _F)^p)$ where $\Lambda \sim 200~\text {MeV}$ is a typical hadronic scale, and $p=1$ or 2.

In Eq. (1), a sum over all possible sub-processes $i+j \rightarrow Q + X$ is understood, where *i*, *j* are the active partons in the proton: $i,j \in \{q,\overline{q}=(u,\overline{u}, d, \overline{d}, s, \overline{s}), g\}$ for a FFNS with three active flavours (3-FFNS) usable for both charm and beauty production, and $i,j \in \{q,\overline{q}=(u,\overline{u}, d, \overline{d}, s, \overline{s}, c, \overline{c}), g\}$ in the case of four active flavours (4-FFNS) often used for beauty production. In the latter case, the charm quark is also an active parton (for $\mu _F > m_c$) and the charm-quark mass is neglected in the hard-scattering cross section $\mathrm {d}{\tilde{\sigma }}$ whereas the beauty quark mass $m_b$ is retained. At the leading order (LO) in $\alpha _S$, there are only two sub-processes which contribute: (i) $q+\overline{q} \rightarrow Q+ \overline{Q}$, (ii) $g + g \rightarrow Q+ \overline{Q}$. At the next-to-leading order (NLO), the virtual one-loop corrections to these $2\rightarrow 2$ processes have to be included in addition to the following $2\rightarrow 3$ processes: (i) $q+\overline{q} \rightarrow Q+ \overline{Q} + g$, (ii) $g + g \rightarrow Q+ \overline{Q} + g$, (iii) $g+q\rightarrow q + Q+ \overline{Q}$ and $g+\overline{q}\rightarrow \overline{q} + Q+ \overline{Q}$. Complete NLO calculations of the integrated/total cross section and of one-particle inclusive distributions were performed in the late 80s [2–5]. These calculations form also the basis for more differential observables/codes [6] (where the phase space of the second heavy quark has not been integrated out) allowing us to study the correlations between the heavy quarks – sometimes referred to as NLO MNR. They are also an important ingredient to the other theories discussed below (FONLL, GM-VFNS, POWHEG, MC@NLO).

The typical range of applicability of the FFNS at NLO is roughly $0 \le p_{\mathrm {T}} \lesssim 5 \times m_Q$. A representative comparison with data has been made for $\mathrm B^{+}$ production in [7] where it is clear that the predictions of the FFNS at NLO using the branching fraction $B(b \rightarrow \mathrm{B}) = 39.8~\%$ starts to overshoot the Tevatron data for $p_{\mathrm {T}} \gtrsim 15~~\text {GeV}/c$ even considering the theoretical uncertainties evaluated by varying the renormalisation and factorisation scales by factors of 2 and 1/2 around the default value^4^$\mu _F =\mu _R =m_\mathrm{T}$ with $m_\mathrm{T}=\sqrt{m_Q^2+p_{\mathrm {T}} ^2}$.

Such a kind of discrepancies at increasing $p_{\mathrm {T}}$ can be attributed to the shift of the momentum between the *b* quark and the *B* meson which can be accounted for by a fragmentation function (FF). Indeed, the scope of the FFNS can be extended to slightly larger $p_{\mathrm {T}}$ by convolving the differential cross section for the production of the heavy quark *Q* with a suitable, scale-independent, FF $D_Q^H(z)$ describing the transition of the heavy quark with momentum $p_Q$ into the observed heavy-flavoured hadron *H* with momentum $p_H=z\,p_Q$ (see [7]):

3 $$\begin{aligned} \mathrm {d}\sigma ^H = \mathrm {d}\sigma ^Q \otimes D_Q^H(z). \end{aligned}$$

At large transverse momenta, the differential cross section falls off with a power-like behaviour $$\mathrm {d}\sigma ^Q/\mathrm {d}p_{\mathrm {T}} \propto 1/p_{\mathrm {T}} ^n$$ with $$n=4,5$$ so that the convolution with the fragmentation function (FF) effectively corresponds to a multiplication with the fourth or fifth Mellin moment of this FF which lowers the cross section and leads to an improved agreement with the data at large $$p_{\mathrm {T}}$$. It should be noted that this FF is included on a purely phenomenological basis and there are ambiguities on how the convolution prescription is implemented ($$E_H = z\, E_Q$$, $$\vec {p}_\mathrm{T}^H = z\, \vec {p}_\mathrm{T}^Q$$) leading to differences at $$p_{\mathrm {T}} \simeq m_Q$$. Furthermore, at NLO, a harder FF should be used than at LO in order to compensate for the softening effects of the gluon emissions. Apart from this, it is generally believed that this scale-independent FF is universal and can be extracted from data, e.g. from $$e^{+}e^{-} $$ data.

The same conclusions about the range of applicability of the FFNS apply at the LHC where the heavy-quark production is dominated by the *gg*-channel (see, e.g. Figure 3 in [7]) over the ${q\overline{q}}$ one. As can be seen, the uncertainty at NLO due to the scale choice is very large (about a factor of two). For the case of top pair production, complete NNLO calculations are now available for both the total cross section [8] and, most recently, the differential distributions [9]. To make progress, it will be crucial to have NNLO predictions for charm and beauty production as well.

*ZM-VFNS* For $p_{\mathrm {T}} \gg m_Q$, the logarithms of the heavy-quark mass ($\frac{\alpha _s}{2\pi } \ln (p_{\mathrm {T}} ^2/m_Q^2)$) become large and will eventually have to be resummed to all orders in the perturbation theory. This resummation is realised by absorbing the large logarithmic terms into the PDFs and FFs whose scale-dependence is governed by renormalisation group equations, the DGLAP evolution equations. This approach requires that the heavy quark is treated as an active parton for factorisation scales $\mu _F \ge \mu _T$ where the transition scale $\mu _T$ is usually (for simplicity) identified with the heavy-quark mass. Such a scheme, where the number of active flavours is changed when crossing the transition scales is called a Variable-Flavour-Number Scheme (VFNS). If, in addition, the heavy-quark mass $m_Q$ is neglected in the calculation of the short-distance cross sections, the scheme is called Zero-Mass VFNS (ZM-VFNS). The theoretical foundation of this scheme is provided by a well-known factorisation theorem and the differential cross section for the production of a heavy-flavoured hadron ($A+B \rightarrow H +X$) is calculated as follows:

4 $$\begin{aligned}&\mathrm {d}\sigma ^{H+X} \simeq \sum _{i,j,k} \int _0^1 \mathrm {d}x_i \int _0^1 \mathrm {d}x_j\, \int _0^1 \mathrm {d}z f^A_i(x_i,\mu _F) \nonumber \\&\quad \times f^B_j(x_j,\mu _F) \mathrm {d}{\hat{\sigma }}_{ij\rightarrow k+X} D_k^H(z,\mu _F') +\mathcal{O}(m_Q^2/p_{\mathrm {T}} ^2). \end{aligned}$$

Because the heavy-quark mass is neglected in the short-distance cross sections ($\mathrm {d}{\hat{\sigma }}$), the predictions in the ZM-VFNS are expected to be reliable only for very large transverse momenta. The sum in Eq. (4) extends over a large number of sub-processes $i+j\rightarrow k+X$ since *a*, *b*, *c* can be gluons, light quarks, and heavy quarks. A calculation of all sub-processes at NLO has been performed in the late 1980s [10].

Concerning the FFs into the heavy-flavoured hadron $H=\mathrm{D,B},\Lambda _c,\ldots$, two main approaches are employed in the literature:

- In the Perturbative-Fragmentation Functions (PFF) approach [11], the FF $D_k^H(z,\mu _F')$ is given by a convolution of a PFF accounting for the fragmentation of the parton *k* into the heavy quark *Q*, $D_k^Q(z,\mu _F')$, with a scale-independent FF $D_Q^H(z)$ describing the hadronisation of the heavy quark into the hadron *H*:
  5 $$\begin{aligned} D_k^H(z,\mu _F') = D_k^Q(z,\mu _F') \otimes D_Q^H(z). \end{aligned}$$
  The PFFs resum the final-state collinear logarithms of the heavy-quark mass. Their scale-dependence is governed by the DGLAP evolution equations and the boundary conditions for the PFFs at the initial scale are calculable in the perturbation theory. On the other hand, the scale-independent FF is a non-perturbative object (in the case of heavy-light-flavoured hadrons) which is assumed to be universal. It is usually determined by a fit to $$e^{+}e^{-} $$ data, although approaches exist in the literature which attempt to compute these functions. It is reasonable to identify the scale-independent fragmentation function in Eq. (3) with the one in Eq. (5). This function describing the hadronisation process involves long-distance physics and might be modified in the presence of a QGP, whereas the PFFs (or the unresummed collinear logarithms $$\ln p_{\mathrm {T}} ^2/m_Q^2$$ in the FFNS) involve only short-distance physics and are the same in $$\mathrm pp$$, p–A, and AA collisions.
- In the Binnewies–Kniehl–Kramer (BKK) approach [12–14], the FFs are not split up into a perturbative and a non-perturbative piece. Instead, boundary conditions at an initial scale $\mu _F' \simeq m_Q$ are determined from $e^{+}e^{-}$ data for the full non-perturbative FFs, $D_k^H(z,\mu _F')$, in complete analogy with the treatment of FFs into light hadrons (pions, kaons). These boundary conditions are again evolved to larger scales $\mu _F'$ with the help of the DGLAP equations.

It is also noteworthy that the BKK FFs ($$D(z,\mu _F')$$) are directly determined as functions in *z*-space whereas the FFs in the PFF approach are determined in Mellin-*N*-space where the *N*th Mellin moment of a function *f*(*z*) ($$0<z<1$$) is defined as $$f(N) = \int _0^1 \mathrm{d}z\ z^{N-1} f(z)$$. *GM-VFNS* The FFNS and the ZM-VFNS are valid only in restricted and complementary regions of the transverse momentum. For this reason, it is crucial to have a unified framework which combines the virtues of the massive FO calculation in the FFNS and the massless calculation in the ZM-VFNS. The General-Mass VFNS (GM-VFNS) [15, 16] is such a framework which is valid in the entire kinematic range from the smallest to the largest transverse momenta ($$p_{\mathrm {T}} \ll m_Q, p_{\mathrm {T}} \simeq m_Q, p_{\mathrm {T}} \gg m_Q$$). It is very similar to the ACOT heavy-flavour scheme [17, 18] which has been formulated for structure functions in deep inelastic scattering (DIS). Different variants of the ACOT scheme exist like the S-ACOT scheme [19] and the (S)-ACOT$$_\chi $$ scheme [20] which are used in global analyses of PDFs by the CTEQ Collaboration and the ACOT scheme has been extended to higher orders in Refs. [21–23]. The theoretical basis for the ACOT scheme has been laid out in an all-order proof of a factorisation theorem with massive quarks by Collins [24]. While the discussion in [24] deals with inclusive DIS, it exemplifies the general principles for the treatment of heavy quarks in perturbative QCD (see also [25, 26]) which should be applicable to other processes as well. Therefore, it is very important to test these ideas also in the context of less inclusive observables. First steps in this direction had been undertaken in [27, 28] where the ACOT scheme had been applied to inclusive D-meson production in DIS. The case of hadroproduction in the ACOT scheme had been studied for the first time in [29] taking into account the contributions from the NLO calculation in the FFNS combined with the massless contributions in the ZM-VFNS from all other sub-processes at $$\mathcal{O}(\alpha _s^2)$$ resumming the collinear logarithms associated to the heavy quark at the leading-logarithmic (LL) accuracy. In contrast, the GM-VFNS has a NLO+NLL accuracy. It has been worked out for $$\gamma \gamma $$, $$\mathrm pp $$, $${\mathrm{p}\overline{\mathrm{p}}} $$, $$e^{+}e^{-} $$, *e*p, and $$\gamma $$p collisions in a series of papers [7, 15, 30–38] and has been successfully compared to experimental data from LEP, HERA, Tevatron and the LHC. Furthermore, inclusive lepton spectra from heavy-hadron decays have been studied for $$\mathrm pp$$ collisions at the LHC at 2.76 and 7 $$\text {TeV}$$ centre-of-mass energy [39] and compared to data from ALICE, ATLAS and CMS. In addition, predictions have been obtained for D mesons produced at $$\sqrt{s}$$$$=$$ 7 $$\text {TeV}$$ from B decays [40]. A number of comparisons with hadroproduction data are discussed in Sect. 2.2.

The cross section for inclusive heavy-flavour hadroproduction in the GM-VFNS is calculated using a factorisation formula similar to the one in Eq. (4):

6 $$\begin{aligned}&\mathrm {d}\sigma ^{H+X} \simeq \sum _{i,j,k} \int _0^1 \mathrm {d}x_i \int _0^1 \mathrm {d}x_j\, \int _0^1 \mathrm {d}z\nonumber \\&\quad \times f^A_i(x_i,\mu _F) f^B_j(x_j,\mu _F) \mathrm {d}{\hat{\sigma }}_{ij\rightarrow k+X}[p_{\mathrm {T}},m_Q] D_k^H(z,\mu _F').\nonumber \\ \end{aligned}$$

In particular, the same sub-processes as in the ZM-VFNS are taken into account. However, the finite heavy-quark-mass terms (powers of $$m_Q^2/p_{\mathrm {T}} ^2$$) are retained in the short-distance cross sections of sub-processes involving heavy quarks. More precisely, the heavy-quark-mass terms are taken into account in the sub-processes $$q+\overline{q} \rightarrow Q+ X$$, $$g + g \rightarrow Q+X$$, $$g+q\rightarrow Q+ X$$ and $$g+\overline{q}\rightarrow Q+X$$, which are also present in the FFNS. However, in the current implementation, they are neglected in the heavy-quark-initiated sub-processes ($$Q+g\rightarrow Q+X$$, $$Q+g\rightarrow g+X$$, ...) as is done in the S-ACOT scheme [19]. The massive hard-scattering cross sections are defined in a way that they approach, in the limit $$m_Q/p_{\mathrm {T}} \rightarrow 0$$, the massless hard-scattering cross sections defined in the $$\overline{\mathrm{MS}}$$ scheme. Therefore, the GM-VFNS approaches the ZM-VNFS at large $$p_{\mathrm {T}} \gg m_Q$$. It can be shown that the GM-VFNS converges formally to the FFNS at small $$p_{\mathrm {T}}$$. However, while the S-ACOT scheme works well for the computation of DIS structure functions at NLO, this scheme causes problems in the hadroproduction case at low $$p_{\mathrm {T}}$$ because the massless $$b $$-quark initiated cross sections diverge in the limit $$p_{\mathrm {T}} \rightarrow 0$$. This problem can be circumvented by a suitable choice for the factorisation scale so that the heavy-quark PDF is switched off sufficiently rapidly and the GM-VFNS approaches the FFNS at small $$p_{\mathrm {T}} $$ [7].

*FONLL* Similar to the GM-VFNS, the Fixed-Order plus Next-to-Leading Logarithms (FONLL) approach [41] is a unified framework which is valid in the entire kinematic range ($$p_{\mathrm {T}} \ll m_Q, p_{\mathrm {T}} \simeq m_Q, p_{\mathrm {T}} \gg m_Q$$). This approach has also been applied to DIS structure functions and is used in the global analyses of PDFs by the NNPDF Collaboration [42, 43]. Predictions for $$c $$ and $$b $$ quark production at the LHC with a centre-of-mass energy of 7 $$\text {TeV}$$ have been presented in [44]. The FONLL scheme is based on the matching of the massive NLO cross section in the FFNS ($$=$$FO) with the massless NLO calculation in the ZM-VNFS ($$=$$RS) according to the prescription

7 $$\begin{aligned} \mathrm {d}\sigma _\mathrm{FONLL} = \mathrm {d}\sigma _\mathrm{FO} + G(m_Q,p_{\mathrm {T}}) \times \left( \mathrm {d}\sigma _\mathrm{RS} - \mathrm {d}\sigma _\mathrm{FOM0}\right) \end{aligned}$$

where $\mathrm {d}\sigma _\mathrm{FOM0}$ is the cross section $\mathrm {d}\sigma _\mathrm{FO}$ in the asymptotic limit $p_{\mathrm {T}} \gg m_Q$ where the finite power-like mass terms can be neglected and the cross section is dominated by the collinear logarithm of the heavy-quark mass.

The condition $\mathrm {d}\sigma _\mathrm{FONLL} \rightarrow d\sigma _\mathrm{RS}$ for $p_{\mathrm {T}} \gg m_Q$ implies that the matching function $G(m_Q,p_T)$ has to approach unity in this limit. Furthermore, in the limit of small transverse momenta $\mathrm {d}\sigma _\mathrm{FONLL}$ has to approach the fixed-order calculation $\mathrm {d}\sigma _\mathrm{FO}$. This can be achieved by demanding that $G(m_Q,p_T)\rightarrow 0$ for $p_{\mathrm {T}} \rightarrow 0$, which effectively suppresses the contribution from the divergent *b*-quark initiated contributions in $\mathrm {d}\sigma _{RS}$. In the FONLL, the interpolating function is chosen to be $G(m_Q,p_{\mathrm {T}}) = p_{\mathrm {T}} ^2/(p_{\mathrm {T}} ^2 + a^2 m_Q^2)$ where the constant is set to $a=5$ on phenomenological grounds. In this language the GM-VFNS is given by $\mathrm {d}\sigma _\mathrm{GM-VFNS} = \mathrm {d}\sigma _\mathrm{FO} + \mathrm {d}\sigma _\mathrm{RS} - \mathrm {d}\sigma _\mathrm{FOM0}$, i.e. no interpolating factor is used.

Other differences concern the non-perturbative input. In particular, the FONLL scheme uses fragmentation functions in the PFF formalism whereas the GM-VFNS uses fragmentation functions which are determined in the *z*-space in the BKK approach.

*Monte Carlo generators* The GM-VFNS and FONLL calculations are mostly analytic and provide a precise description of the inclusive production of a heavy hadron or its decay products at NLO$+$NLL accuracy. Compared to this, general-purpose Monte-Carlo generators like PYTHIA [45] or HERWIG [46] allow for a more complete description of the hadronic final state but only work at LO$+$LL accuracy. However, in the past decade, NLO Monte Carlo generators have been developed using the MC@NLO [47] and POWHEG [48] methods for a consistent matching of NLO calculations with parton showers. They, therefore, have all the strengths of Monte Carlo generators, which allow for a complete modelling of the hadronic final state (parton showering, hadronisation, decay, detector response), while, at the same time, the NLO accuracy in the hard scattering is kept and the soft/collinear regimes are resummed at the LL accuracy. A comparison of POWHEG NLO Monte Carlo predictions for heavy-quark production in $\mathrm pp$ collisions at the LHC with the ones from the GM-VFNS and FONLL can be found in [49].

#### Quarkonium-production mechanism

The theoretical study of quarkonium-production processes involves both pertubative and non-perturbative aspects of QCD. On one side, the production of the heavy-quark pair, ${Q\overline{Q}}$, which will subsequently form the quarkonium, is expected to be perturbative since it involves momentum transfers at least as large as the mass of the considered heavy quark, as for open-heavy-flavour production discussed in the previous section. On the other side, the evolution of the ${Q\overline{Q}}$ pair into the physical quarkonium state is non-perturbative, over long distances, with typical momentum scales such as the momentum of the heavy-quarks in the bound-state rest frame, $m_Q v$ and their binding energy $m_Q v^2$, *v* being the typical velocity of the heavy quark or antiquark in the quarkonium rest frame ($v^2\sim 0.3$ for the charmonium and 0.1 for the bottomonium).

In nearly all the models or production mechanisms discussed nowadays, the idea of a factorisation between the pair production and its binding is introduced. Different approaches differ essentially in the treatment of the hadronisation, although some may also introduce new ingredients in the description of the heavy-quark-pair production. In the following, we briefly describe three of them which can be distinguished in their treatment of the non-perturbative part: the Colour-Evaporation Model (CEM), the Colour-Singlet Model (CSM), the Colour-Octet Mechanism (COM), the latter two being encompassed in an effective theory referred to as Non-Relativistic QCD (NRQCD).

*The Colour-Evaporation Model (CEM)* This approach is in line with the principle of quark–hadron duality [50, 51]. As such, the production cross section of quarkonia is expected to be directly connected to that to produce a ${Q\overline{Q}}$ pair in an invariant-mass region where its hadronisation into a quarkonium is possible, that is between the kinematical threshold to produce a quark pair, $2m_Q$, and that to create the lightest open-heavy-flavour hadron pair, $2m_{H}$.

The cross section to produce a given quarkonium state is then supposed to be obtained after a multiplication by a phenomenological factor $F_\mathcal{Q}$ related to a process-independent probability that the pair eventually hadronises into this state. One assumes that a number of non-perturbative-gluon emissions occur once the $Q \overline{Q}$ pair is produced and that the quantum state of the pair at its hadronisation is essentially decorrelated – at least colour-wise – with that at its production. From the reasonable assumption [52] that one ninth – one colour-*singlet*${Q\overline{Q}}$ configuration out of nine possible – of the pairs in the suitable kinematical region hadronises in a quarkonium, a simple statistical counting [52] was proposed based on the spin $J_\mathcal{Q}$ of the quarkonium $\mathcal{Q}$, $F_\mathcal{Q}= {1}/{9} \times {(2 J_\mathcal{Q} +1)}/{\sum _i (2 J_i +1)}$, where the sum over *i* runs over all the charmonium states below the open heavy-flavour threshold. It was shown to reasonably account for existing $\mathrm {J}/\psi$ hadroproduction data of the late 1990s and, in fact, is comparable to the fit value in [53].

Mathematically, one has

8 $$\begin{aligned} \sigma ^\mathrm{(N)LO}_\mathcal{Q}= F_\mathcal{Q}\int _{2m_Q}^{2m_H} \frac{\mathrm {d}\sigma _{Q\overline{Q}}^\mathrm{(N)LO}}{\mathrm {d}m_{Q\overline{Q}}}\mathrm {d}m_{Q\overline{Q}} \end{aligned}$$

In the latter formula, a factorisation between the short-distance ${Q\overline{Q}}$-pair production and its hadronisation is the quarkonium state is of course implied although it does not rely on any factorisation proof. In spite of this, this model benefits – as some figures will illustrate it in the next section – from a successful phenomenology but for the absence of predictions for polarisation observables and discrepancies in some transverse momentum spectra.

*The Colour-Singlet Model (CSM)* The second simplest model to describe quarkonium production relies on the rather opposite assumption that the quantum state of the pair does *not* evolve between its production and its hadronisation, neither in spin, nor in colour [54–56] – gluon emissions from the heavy-quark are suppressed by powers of $\alpha _s(m_Q)$. In principle, they are taken into account in the (p)QCD corrections to the hard-scattering part account for the ${Q\overline{Q}}$-pair production. If one further assumes that the quarkonia are non-relativistic bound states with a highly peaked wave function in the momentum space, it can be shown that partonic cross section for quarkonium production should then be expressed as that for the production of a heavy-quark pair with zero relative velocity, *v*, in a colour-singlet state and in the same angular-momentum and spin state as that of the to-be produced quarkonium, and the square of the Schrödinger wave function at the origin in the position space. In the case of hadroproduction, which interests us most here, one should further account for the parton *i*, *j* densities in the colliding hadrons, $f_{i,j}(x)$, in order to get the following hadronic cross section:

9 $$\begin{aligned} \mathrm{d}\sigma [\mathcal{Q}+X]= & {} \sum _{i,j}\!\int \! \mathrm {d}x_{i} \,\mathrm {d}x_{j} \,f_{i}(x_i,\mu _F) \,f_{j}(x_j,\mu _F)\nonumber \\&\times \mathrm {d}\hat{\sigma }_{i+j\rightarrow (Q\overline{Q})+X}(\mu _R,\mu _F) |\psi (0)|^2 \end{aligned}$$

In the case of *P*-waves, $|\psi (0)|^2$ vanishes and, in principle, one should consider its derivative and that of the hard scattering. In the CSM, $|\psi (0)|^2$ or $|\psi '(0)|^2$ also appear in decay processes and can be extracted from decay-width measurements. The model then becomes fully predictive but for the usual unknown values of the non-physical factorisation and renormalisation scales and of the heavy-quark mass entering the hard part. A bit less than ten years ago, appeared the first evaluations of the QCD corrections [57–61] to the yields of $\mathrm {J}/\psi$ and $\Upsilon$ (also commonly denoted $\mathcal Q$) in hadron collisions in the CSM. It is now widely accepted [62–64] that $\alpha ^4_s$ and $\alpha ^5_s$ corrections to the CSM are significantly larger than the LO contributions at $\alpha ^3_s$ at mid and large $p_{\mathrm {T}}$ and that they should systematically be accounted for in any study of their $p_{\mathrm {T}}$ spectrum.

Possibly due to its high predictive power, the CSM has faced several phenomenological issues although it accounts reasonably well for the bulk of hadroproduction data from RHIC to LHC energies [65–67], $e^{+}e^{-}$ data at *B* factories [68–70] and photoproduction data at HERA [71]. Taking into account NLO – one loop – corrections and approximate NNLO contributions (dubbed NNLO$^\star$ in the following) has reduced the most patent discrepancies in particular for $p_{\mathrm {T}}$ up to a couple of $m_\mathcal{Q}$ [72–75]. A full NNLO computation (i.e.  at $\alpha ^5_s$) is, however, needed to confirm this trend.

It is, however, true that the CSM is affected by infra-red divergences in the case of *P*-wave decay at NLO, which were earlier regulated by an ad hoc binding energy [76]. These can nevertheless be rigorously cured [77] in the more general framework of NRQCD which we discuss now and which introduce the concept of colour-octet states.

*The Colour-Octet Mechanism (COM) and NRQCD* Based on the effective theory NRQCD [78], one can express in a more rigorous way the hadronisation probability of a heavy-quark pair into a quarkonium via long-distance matrix elements (LDMEs). In addition to the usual expansion in powers of $\alpha _s$, NRQCD further introduces an expansion in *v*. It is then natural to account for the effect of higher-Fock states (in *v*) where the ${Q\overline{Q}}$ pair is in an octet state with a different angular-momentum and spin states – the sole consideration of the *leading* Fock state (in *v*) amounts to the CSM, which is thus a priori the *leading* NRQCD contribution (in *v*). However, this opens the possibility for non-perturbative transitions between these coloured states and the physical meson. One of the virtues of this is the consideration of $^3S_1^{[8]}$ states in *P*-wave productions, whose contributions cancel the aforementioned divergences in the CSM. The necessity for such a cancellation does not, however, fix the relative importance of these contributions. In this precise case, it depends on a non-physical scale $\mu _\Lambda$.

As compared to the Eq. (9), one has to further consider additional quantum numbers (angular momentum, spin and colour), generically denoted *n*, involved in the production mechanism:

10 $$\begin{aligned}&\mathrm {d}\sigma [\mathcal{Q}+X] =\sum _{i,j,n}\!\int \! \mathrm {d}x_{i} \,\mathrm {d}x_{j} \,f_{i}(x_i,\mu _F) \,f_{j}(x_j,\mu _F)\nonumber \\&\quad \times \mathrm {d}\hat{\sigma }_{i+j\rightarrow (Q\overline{Q})_{n}+X} (\mu _R,\mu _F,\mu _\Lambda ) \langle \mathcal{O}_\mathcal{Q}^{n} \rangle . \end{aligned}$$

Instead of the Schrödinger wave function at the origin squared, the former equation involves the aforementioned LDMEs, $\langle \mathcal{O}_\mathcal{Q}^{n} \rangle$, which *cannot* be fixed by decay-width measurements nor lattice studies^5^ – but the leading CSM ones of course. Only relations based on Heavy-Quark Spin Symmetry (HQSS) can relate some of them.

Three groups (Hamburg [80], IHEP [81] and PKU [82]) have, in recent years, carried out a number of NLO studies^6^ of cross section fits to determine the NRQCD LDMEs. A full description of the differences between these analyses is beyond the scope of this review, it is, however, important to stress that they somehow contradict each other in their results as regards the polarisation observables. In particular, in the case of the $\mathrm {J}/\psi$, the studies of the Hamburg group, which is the only one to fit low $p_{\mathrm {T}}$ data from hadroproduction, electroproduction and $e^{+}e^{-}$ collisions at *B* factories, predict a strong transverse polarised yield at variance with the experimental data.

*Theory prospects* Although NRQCD is 20 years old, there does not exist yet a complete proof of factorisation, in particular, in the case of hadroproduction. A discussion of the difficulties in establishing NRQCD factorisation can be found in [64]. A first step was achieved in 2005 by the demonstration [84, 85] that, in the large-$p_{\mathrm {T}}$ region where a description in terms of fragmentation functions is justified, the infra-red poles at NNLO could be absorbed in the NRQCD LDMEs, provided that the NRQCD production operators were modified to include nonabelian phases.

As mentioned above, it seems that the mere expansion of the hard matrix elements in $\alpha _s$ is probably not optimal since higher QCD corrections receive contributions which are enhanced by powers of $p_{\mathrm {T}}/m_\mathcal{Q}$. It may therefore be expedient to organise the quarkonium-production cross section in powers of $p_{\mathrm {T}}/m_\mathcal{Q}$ before performing the $\alpha _s$-expansion of the short-distance coefficients for the ${Q\overline{Q}}$ production. This is sometimes referred to as the fragmentation-function approach (see [86, 87]) which offers new perspectives in the theoretical description of quarkonium hadroproduction especially at mid and large $p_{\mathrm {T}}$. Complementary information could also be obtained from similar studies based on Soft Collinear Effective Theory (SCET); see [88].

At low $$p_{\mathrm {T}}$$, it was recently emphasised in [67] that one-loop results show an intriguing energy dependence which might hint at a break-down of NRQCD factorisation in this kinematical region. In any case, as for now, past claims that colour-octet transitions are the dominant source of the low-$$p_{\mathrm {T}}$$$$\mathrm {J}/\psi $$ and $$\Upsilon $$ cannot be confirmed at one loop accuracy. Approaches such as the $$k_{\mathrm {T}}$$ factorisation based on the Lipatov action in the quasi-multi Regge kinematics (see [89, 90] for quarkonium studies), the TMD factorisation (see [91, 92] for recent applications to quarkonium production) or the combined use of the CGC formalism and NRQCD [93, 94] may therefore bring their specific bricks in the building of a consistent theory of quarkonium production. Finally, let us mention the relevance of the colour-transfer mechanism [95], beyond NRQCD, in the case of production of a quarkonium in the vicinity of another heavy quark.

**Fig. 1:**
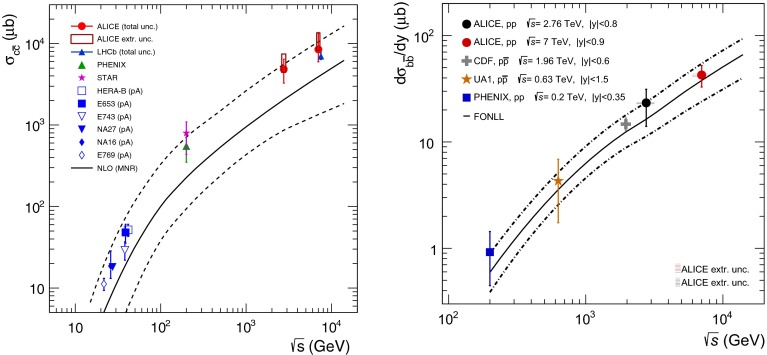
*Left* Total (extrapolated) ${c\overline{c}}$ cross section as a function of $\sqrt{s}$  [100–106]. Data in proton–nucleus (p–A) or deuteron–nucleus (d–A) collisions were scaled down assuming no nuclear effect. *Right* A compilation of the ${b\overline{b}}$ differential cross section measurements at mid-rapidity in $\mathrm pp$ and ${\mathrm{p}\overline{\mathrm{p}}}$ collisions [107–111]. Results are compared to pQCD calculations, NLO MNR [6] and FONLL [44, 99] for ${c\overline{c}}$ and ${b\overline{b}}$, respectively

**Fig. 2:**
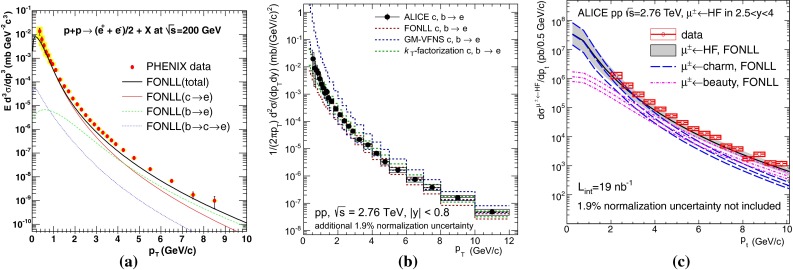
$\mathrm{d}\sigma /\mathrm{d}p_{\mathrm {T}}$ for heavy-flavour decay leptons in $\mathrm pp$ collisions: **a** electrons at mid-rapidity for $\sqrt{s}$ $=$ 200$~\text {GeV}$ from PHENIX [112], **b** electrons at mid-rapidity for $\sqrt{s}$ $=$ 2.76 $\text {TeV}$  [118] and **c** muons at forward-rapidity for $\sqrt{s}$ $=$ 2.76 $\text {TeV}$ from ALICE [119]. FONLL [44, 99] predictions are also shown; in **a** and **c** the calculations for leptons from charm and beauty decays are shown separately (without theoretical uncertainty bands in **a**). GM-VFNS [15, 16] and $k_{\mathrm {T}}$-factorisation [122] calculations are also drawn in **b**

### Recent cross section measurements at hadron colliders

Due to their short lifetimes (at most a picosecond), the production of open-heavy-flavour particles is studied through their decay products. Four main techniques are used:

1. Fully reconstruction of exclusive decays, such as $\mathrm{B}^0 \rightarrow \mathrm {J}/\psi \, \mathrm{K^0_S}$ or $\mathrm {D}^{0} \rightarrow \mathrm{K}^{-} \, \pi ^{+}$.
2. Selection of specific (semi-)inclusive decays. For beauty production, one looks for a specific particle, for example $\mathrm {J}/\psi$, and imposes it to point to a secondary vertex displaced a few hundred^7^$\upmu$m from the primary vertex. Such *displaced* or *non-prompt* mesons are therefore supposed to come from *b*-decay only.
3. Detection of leptons from these decays. This can be done (i) by subtracting a cocktail of known/measured sources (photon conversions, Dalitz decays of $\pi ^0$ and $\eta$ in the case of electrons, light hadron, Drell–Yan pair, $\mathrm {J}/\psi$,...) to the lepton yield. Alternatively, the photon conversion and Dalitz decay contribution can be evaluated via an invariant-mass analysis of the $e^{+}e^{-}$ pairs. (ii) By selecting displaced leptons with a track pointing to a secondary vertex separated by few hundred $\upmu$m from the primary vertex.
4. Reconstruction of $$c $$- and $$b $$-jets. Once a jet is reconstructed, a variety of reconstructed objects, such as tracks, vertices and identified leptons, are used to distinguish between jets from light or from heavy flavour. A review of $$b $$-tagging methods used by the CMS Collaboration can be found in [96].

Different methods are used in different contexts, depending on the detector information available, the trigger strategy, the corresponding statistics (hadronic decays are less abundant than leptonic ones), the required precision (only exclusive decay channels allow for a full control of the kinematics), the kinematical range ($$b $$-tagged jets give access to very large $$p_{\mathrm {T}}$$ whereas exclusive-decay channels allow for differential studies down to $$p_{\mathrm {T}}$$ equal 0). A fifth method based on the indirect extraction of the total charm- and beauty-production from *dileptons* – as opposed to single leptons – (see e.g.  [97]) is not discussed in this review.

Hidden-heavy-flavour, i.e. quarkonia, are also analysed through their decay products. The triplet *S*-waves are the most studied since they decay up to a few per cent of the time in dileptons. This is the case for $\mathrm {J}/\psi$, $\psi \text {(2S)}$, $\Upsilon \text {(1S)}$, $\Upsilon \text {(2S)}$, $\Upsilon \text {(3S)}$. The triplet *P*-waves, such as the $\chi _c$ and $\chi _b$, are usually reconstructed via their radiative decays into a triplet *S*-wave. For other states, such as the singlet *S*-wave, studies are far more complex. The very first inclusive hadroproduction study of $\eta _c$ was just carried out this year in the ${\mathrm{p}\overline{\mathrm{p}}}$ decay channel by the LHCb Collaboration [98].

A compilation of the measurements of the $p_{\mathrm {T}}$-integrated ${c\overline{c}}$ and ${b\overline{b}}$ cross section, $\sigma _{{c\overline{c}}}$ and $\sigma _{{b\overline{b}}}$, is shown in Fig. 1 from SPS to LHC energies. Let us stress that most of the $p_{\mathrm {T}}$-integrated results and nearly all *y*-integrated ones are based on different extrapolations, which significantly depend on theoretical inputs and which are not necessarily identical in the presence of nuclear effects. The results are described within the uncertainties by pQCD calculations, NLO MNR [6] and FONLL [44, 99] for the ${c\overline{c}}$ and ${b\overline{b}}$, respectively. Note that most of the experimental results for $\sigma _{{c\overline{c}}}$, in particular at high energies, lie on the upper edge of the NLO MNR uncertainties.

**Fig. 3:**
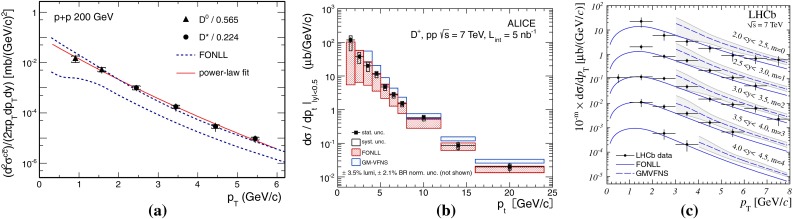
$\mathrm{d}\sigma /\mathrm{d}p_{\mathrm {T}}$ for D-meson production in $\mathrm pp$ collisions at different energies. **a** $\mathrm {D}^{0}$ and $\mathrm {D}^{*+}$ measurements at $\sqrt{s}$ $=$ 200$~\text {GeV}$ with the STAR detector [103]. **b** $\mathrm {D}^{+}$ data at $\sqrt{s}$ $=$ 7 $\text {TeV}$ with the ALICE detector [125]. **c** $\mathrm {D}^{+}_{s}$ data at $\sqrt{s}$ $=$ 7 $\text {TeV}$ with the LHCb detector [106], where data from different *y* ranges are scaled by factors $10^{-m}$, with *m* shown in the plot. The measurements are compared to FONLL [44, 99] and GM-VFNS [15, 16] calculations

#### Leptons from heavy-flavour decays

The first open-heavy-flavour measurements in heavy-ion collisions were performed by exploiting heavy-flavour decay leptons at RHIC by the PHENIX and STAR Collaborations. These were done both in $$\mathrm pp$$ and AA collisions [112–116]. At the LHC, the ATLAS and ALICE Collaborations have also performed such studies in heavy-ion collisions [117–121]. A selection of the $$p_{\mathrm {T}}$$-differential production cross sections of heavy-flavour decay leptons in $$\mathrm pp$$ collisions at different rapidities and energies is presented in Fig. 2. The measurements are reported together with calculations of FONLL [44, 99] for $$\sqrt{s}$$$$=$$ 0.2 and 2.76  $$\text {TeV}$$, GM-VFNS [15, 16] and $$k_{\mathrm {T}}$$-factorisation [122] at $$\sqrt{s}$$$$=$$ 2.76 $$\text {TeV}$$. The POWHEG predictions [49], not shown in this figure, show a remarkable agreement with the FONLL ones. The differential cross sections of heavy-flavour-decay leptons are well described by pQCD calculations.

In addition, leptons from open charm and beauty production can be separated out via: (i) a cut on the lepton impact parameter, i.e.  the distance between the origin of the lepton and the collision primary vertex, (ii) a fit of the lepton impact-parameter distribution using templates of the different contributions to the inclusive spectra, (iii) studies of the azimuthal angular correlations between heavy-flavour decay leptons and charged hadrons (see e.g.  [107, 123]). These measurements are also described by pQCD calculations.

#### Open charm

Recently, D-meson production has been studied at RHIC, Tevatron and LHC energies [102–104, 106, 124–126]. The measurements were performed by fully reconstructing the hadronic decays of the D mesons, e.g.  $$\mathrm {D}^{0} \rightarrow \mathrm{K}^{-}\pi ^{+}$$ and charge conjugates. D-meson candidates are built up of pairs or triplets of tracks with the appropriate charge sign combination. The analyses exploit the detector particle identification abilities to reduce the combinatorial background, which is important at low $$p_{\mathrm {T}}$$. For the measurements at Tevatron and LHC, the background is also largely reduced by applying topological selections on the kinematics of the secondary decay vertices, typically displaced by few hundred $$\upmu $$m from the interaction vertex. The results at RHIC energies report the *inclusive* D-meson yields [103], i.e. those from both $$c $$ and $$b $$ quark fragmentation. The former are called *prompt*, and the later *secondary* D mesons. The measurements at Tevatron and LHC energies report prompt D-meson yields. Prompt yields include both the *direct* production and the *feed-down* from excited charmed resonances. The secondaries contribution to the D-meson yields is evaluated and subtracted by: (i) either scrutinising the D-meson candidates impact-parameter distribution, exploiting the larger lifetime of $$b $$- than $$c $$-flavoured hadrons [102, 106, 124], which requires large statistics, (ii) or evaluating the beauty hadron contribution using pQCD-based calculations [104, 125, 126], advantageous strategy for smaller data samples but limited by the theoretical uncertainties.

**Fig. 4:**
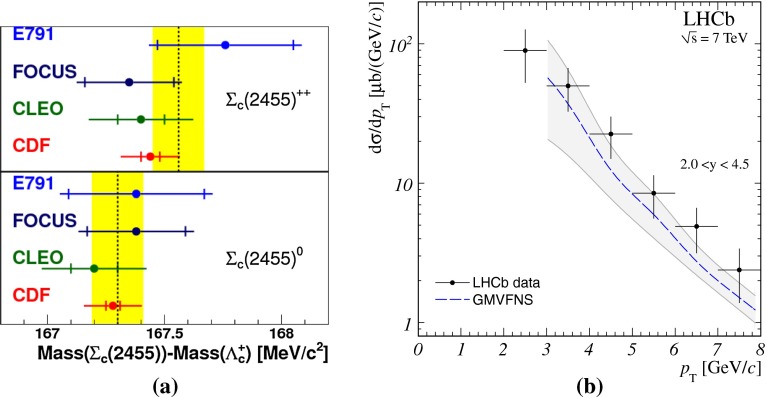
**a** $\Lambda _c^{+}$ and $\Sigma _c^{+} (2455)$ mass differences as measured by CDF [127], Fermilab E791 [128], FOCUS [129], and CLEO [130]. The *vertical dashed line* together with the *surrounding* *box* symbolises the world average value and its uncertainty taken from Ref. [131]. **b** $\mathrm{d}\sigma /\mathrm{d}p_{\mathrm {T}}$ for $\Lambda _c^{+}$ production in $\mathrm pp$ collisions at $\sqrt{s}$ $=$ 7 $\text {TeV}$  [106] compared to GM-VFNS [15, 16] calculations

Figure 3 presents a selection of the D-meson measurements compared to pQCD calculations. The $$\mathrm {D}^{0} $$, $$\mathrm {D}^{+} $$ and $$\mathrm {D}^{*+} $$$$\mathrm{d}\sigma /\mathrm{d}p_{\mathrm {T}} $$ are reproduced by the theoretical calculations within uncertainties. Yet, FONLL [44, 99] and POWHEG [48] predictions tend to underestimate the data, whereas GM-VFNS [15, 16] calculations tend to overestimate the data (see Figures 3 and 4 in [49]). At low $$p_{\mathrm {T}}$$, where the quark mass effects are important, the FONLL and POWHEG predictions show a better agreement with data. At intermediate to high $$p_{\mathrm {T}}$$, where the quark mass effects are less important, all the FONLL, POWHEG, GM-VFNS and $$k_{\mathrm {T}}$$-factorisation calculations agree with data. The agreement among the FONLL and POWHEG calculations is better for heavy-flavour decay leptons than for charmed mesons, which seems to be related to the larger influence of the fragmentation model on the latter. The $$\mathrm {D}^{+}_{s} $$$$p_{\mathrm {T}} $$-differential cross section is compared to calculations in Fig. 3c. The $$\mathrm {D}^{+}_{s} $$ measurements are also reproduced by FONLL, GM-VFNS and $$k_{\mathrm {T}}$$-factorisation predictions, but POWHEG calculations predict a lower production cross section than data.

Charmed baryon production measurements in hadron colliders are scarce. The properties and decay branching ratios of the $$\Lambda _c$$, $$\Sigma _c$$ and $$\Xi _c$$ states have been studied at the charm- and B-factories and fixed-target experiments; see e.g.  [132–135]. An example are the results by Fermilab E791 [128], FOCUS [129], and CLEO [130] Collaborations. The CDF Collaboration measured charmed baryons in $${\mathrm{p}\overline{\mathrm{p}}} $$ collisions at $$\sqrt{s}$$$$=$$ 1.96 $$\text {TeV}$$; see for example [127]. For illustration, a compilation of the $$\Sigma _c^{+} $$ and $$\Lambda _c^{+} $$ mass difference is shown in Fig. 4a. The LHCb Collaboration measured the $$p_{\mathrm {T}}$$ and *y* differential production cross section of $$\Lambda _c$$ in $$\mathrm pp$$ collisions at $$\sqrt{s}$$$$=$$ 7 $$\text {TeV}$$  [106]. Figure 4b shows the $$p_{\mathrm {T}} $$-differential cross section compared to GM-VFNS calculations. No dedicated FONLL calculation is available for $$\Lambda _c$$ production due to the lack of knowledge of the fragmentation function. The GM-VFNS predictions include the fragmentation functions resulting from a fit to $$e^{+}e^{-} $$ collider data [34], where the prompt and secondary contributions to the measurements were not separated.

**Fig. 5:**
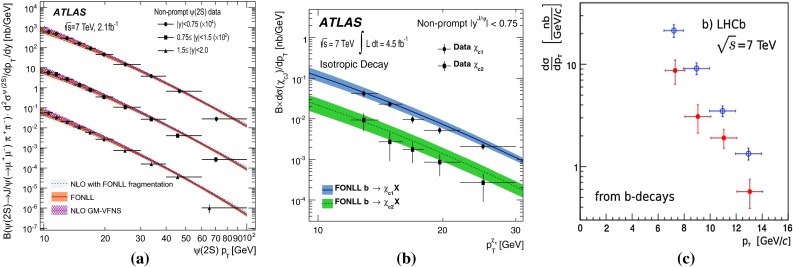
$$p_{\mathrm {T}} $$-differential cross sections for non-prompt charmonia – assumed to come from $$b $$ decays – for **a** $$\psi \text {(2S)} $$ [136] $$\mathrm{d}^2\sigma /\mathrm{d}p_{\mathrm {T}} \mathrm{d}y$$ by ATLAS compared to NLO [6], FONLL [44, 99] and GM-VFNS [15, 16] calculations, **b** $$\chi _{c1}$$ and $$\chi _{c2}$$ $$\mathrm{d}\sigma /\mathrm{d}p_{\mathrm {T}} $$ by ATLAS [137] compared to FONLL [44], **c** $$\eta _c $$ and $$\mathrm {J}/\psi $$ $$\mathrm{d}\sigma /\mathrm{d}p_{\mathrm {T}} $$ by LHCb [98]

#### Open beauty

Open-beauty production is usually measured by looking for $$b $$-jets or for beauty hadrons via their hadronic decays, similarly to D mesons. They have been traditionally studied at the $$e^{+}e^{-} $$ B-factories (see e.g.  [134, 135]), where, despite the small $$b $$-quark production cross section, the large luminosity allows for precise measurements, such as those of the CKM matrix. Yet, heavier states like the $$\mathrm {B}_s$$, $$\mathrm {B}_c$$ or $$\Lambda _c$$ cannot be produced at the B-factories. They are, however, studied at Tevatron and at the LHC hadron colliders. The higher collision energy increases their production cross section, although their reconstruction is more difficult at hadron colliders due to the larger combinatorics compared to the $$e^{+}e^{-} $$ environment. It should also be kept in mind that the experiments optimised to study the high-$$p_{\mathrm {T}} $$ probes, like top production, are not as good for low-$$p_{\mathrm {T}} $$ measurements, and often require the usage of dedicated triggers.

As discussed in the Sect. 2.1.1, predictions for open-beauty cross sections rely on the fragmentation functions derived from fits to $$e^{+}e^{-} $$ data [35, 138]. A high accuracy on the $$e^{+}e^{-} $$ measurements and on the fragmentation function parametrisations is required to calculate the $$b $$-hadron production cross section at hadron colliders. $$b $$-jet measurements have the advantage to be the least dependent on the $$b $$-quark fragmentation characteristics.

In addition, measurements of the B cross section via a displaced charmonium have been performed multiple times at Tevatron and at LHC. Charmonia from beauty decays are selected by fitting the pseudo-proper decay length distribution of the charmonium candidates, $$L_{xy} \left( m / p_{\mathrm {T}} \right) _{\mathrm {J}/\psi }$$. Figure 5 presents a selection of the LHC results: the non-prompt $$p_{\mathrm {T}} $$-differential cross section of $$\mathrm {J}/\psi $$, $$\psi \text {(2S)} $$, $$\eta _c $$, $$\chi _{c1}$$ and $$\chi _{c2}$$ in $$\mathrm pp$$ collisions at $$\sqrt{s}$$$$=$$ 7 $$\text {TeV}$$  [98, 136, 137]. The results at intermediate to low $$p_{\mathrm {T}}$$ are well reproduced by the FONLL [44, 99], NLO GM-VFNS [15, 16] and NLO [6] with FONLL fragmentation calculations. At high-$$p_{\mathrm {T}} $$ the predictions tend to overestimate data. This could be related to the usage of the $$e^{+}e^{-} $$ fragmentation functions in an unexplored kinematic range. Figure 5c, which reports the first measurement of non-prompt charmonium in a purely hadronic decay channel at hadron colliders, shows a similar transverse-momentum spectrum for non-prompt singlet and triplet *S*-wave charmonia.

**Fig. 6:**
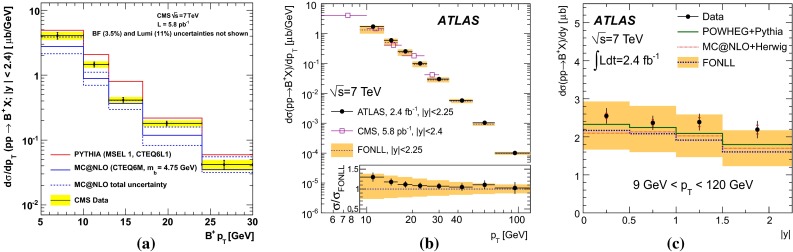
$\mathrm {B}^{+}$differential cross sections $\mathrm{d}\sigma /\mathrm{d}p_{\mathrm {T}}$ (**a**, **b**) and $\mathrm{d}\sigma /\mathrm{d}y$ (**c**) in $\mathrm pp$ collisions at $\sqrt{s}$ $=$ 7 $\text {TeV}$ compared to theory predictions [139, 140]. In **c** the *error bars* correspond to the differential cross-section measurement with total uncertainty (*lines on the error bars* indicate the statistical component)

Studies of open-beauty production have also been performed in exclusive channels at Tevatron and at the LHC, e.g. in the case of $$\mathrm {B}^{\pm }$$, $$\mathrm {B}^{0}$$ and $$\mathrm {B}^0_s$$ [139, 140, 142–148]. As example, Fig. 6 presents the $$\mathrm {B}^{+}$$$$p_{\mathrm {T}} $$ and *y* differential cross section in $$\mathrm pp$$ collisions at $$\sqrt{s}$$$$=$$ 7 $$\text {TeV}$$ compared to theory predictions [139, 140]. PYTHIA (D6T tune), that has LO $$+$$ LL accuracy, does not provide a good description of the data. This could be explained by the choice of $$m_b$$ and by the fact that for $$p_{\mathrm {T}} \simeq m_b$$, NLO and resummation effects become important, which are, in part, accounted for in FONLL [44, 99] or MC@NLO. POWHEG and MC@NLO calculations are quoted with an uncertainty of the order of 20–40 %, from $$m_b$$ and the renormalisation and factorisation scales, and describe the data within uncertainties. The FONLL prediction provides a good description of the measurements within uncertainties.

**Fig. 7:**
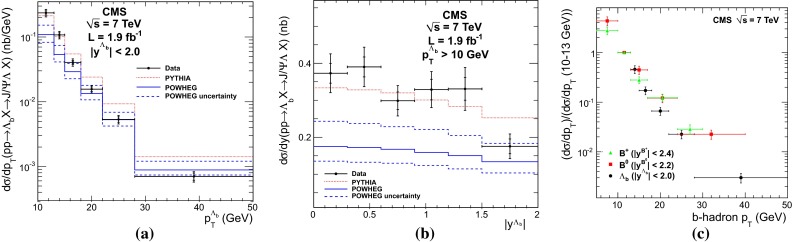
$\Lambda _b^{+}$ studies at the LHC in $\mathrm pp$ collisions at $\sqrt{s}$ $=$ 7 $\text {TeV}$ by CMS [141] compared to PYTHIA and POWHEG calculations for **a** $\mathrm{d}\sigma /\mathrm{d}p_{\mathrm {T}}$ for $|y|<2.0$ and for **b** $\mathrm{d}\sigma /\mathrm{d}y$ for $\Lambda _b^{+}$ for $p_{\mathrm {T}} > 10$ $~\text {GeV}$. **c** Comparison of the (self-normalised) $p_{\mathrm {T}}$ differential cross section for $\mathrm {B}^{+}$, $\mathrm {B}^0$ and $\Lambda _b^{+}$

Measurements of the beauty and charm baryon production in $${\mathrm{p}\overline{\mathrm{p}}} $$ collisions at $$\sqrt{s}$$$$=$$ 1.96 $$\text {TeV}$$ are summarised in Ref. [154]. In particular, the doubly strange $$b $$-baryon $$\Omega _b^{-}$$ and measurement of the $$\Xi _b^{-}$$ and $$\Omega _b^{-}$$ properties can be found in Refs. [155, 156]. Such measurements have also been performed at the LHC in $$\mathrm pp$$ collisions at $$\sqrt{s}$$$$=$$ 7 and 8 $$\text {TeV}$$. For example, the observation of the $$\Xi _b$$ baryon states was reported in Refs. [157, 158]. The measured mass and width of the $$\Xi _b$$ baryon states is consistent with theoretical expectations [133, 159–166]. The $$\Lambda _b^{+} $$$$p_{\mathrm {T}} $$ and *y* differential production cross section in $$\mathrm pp$$ collisions at $$\sqrt{s}$$$$=$$ 7 $$\text {TeV}$$ by CMS [141] is reported in Fig. 7. The $$\Lambda _b $$$$\mathrm{d}\sigma /\mathrm{d}p_{\mathrm {T}} $$ and $$\mathrm{d}\sigma /\mathrm{d}y $$ are not reproduced by neither PYTHIA (Z2 tune) nor POWHEG calculations: PYTHIA expects a harder $$p_{\mathrm {T}}$$-distribution and flatter *y* distribution than data, while POWHEG underestimates its production cross section, particularly at low $$p_{\mathrm {T}}$$; see Fig. 7a and b. The measured $$\Lambda _b $$$$p_{\mathrm {T}} $$-spectrum at mid-rapidity seems to fall more steeply than the $$\mathrm {B}^0$$ and $$\mathrm {B}^{+}$$ ones, see Fig. 7c, and falls also faster than predicted by PYTHIA and POWHEG. As discussed for the non-prompt charmonium measurements, this could be influenced by the lack of data to extract the fragmentation functions in this kinematic region.

**Fig. 8:**
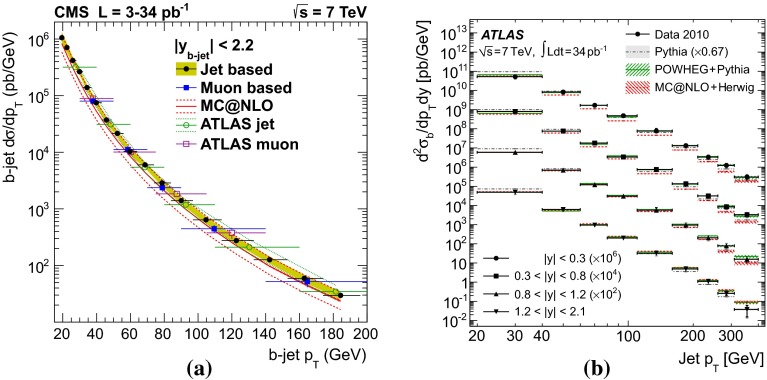
$$b $$-jet cross section as a function of $$p_{\mathrm {T}}$$ in $$\mathrm pp$$ collisions at $$\sqrt{s}$$ $$=$$ 7 $$\text {TeV}$$: **a** $$\mathrm{d}\sigma /\mathrm{d}p_{\mathrm {T}} $$ from the lifetime-based and muon-based analyses by CMS [149] and ATLAS [150] compared to the MC@NLO calculation, and **b** $$\mathrm{d}^2\sigma /\mathrm{d}p_{\mathrm {T}} \mathrm{d}y$$ by ATLAS from the lifetime-based analysis [150] compared to the predictions of PYTHIA, POWHEG (matched to PYTHIA) and MC@NLO (matched to HERWIG) [46, 47, 151–153]

The fragmentation of the $$b $$ quark is relatively hard compared to that of lighter flavours, with the $$b $$-hadron taking about 70 % of the parton momentum on average at the *Z*-pole [167]. Identification of jets from beauty quark fragmentation or “$$b$$-tagging” can be achieved by direct reconstruction of displaced vertices, although the efficiency for doing so is limited. Higher efficiency can be achieved by looking for large impact-parameter tracks inside jets, or by a combination of the two methods, which are collectively known as lifetime tagging. Leptons inside jets can also be used for $$b $$-tagging, but, due to the branching fraction, are usually only used as a calibration of the lifetime methods. At the LHC, both ATLAS and CMS have performed measurements of the $$b $$-jet cross section [149, 150]. Theoretical comparisons can be made to models which calculate fully exclusive final states, which can be achieved by matching NLO calculations to parton showers [168]. Figure 8 shows the $$b $$-jet cross section measurement by ATLAS and CMS in $$\mathrm pp$$ collisions at $$\sqrt{s}$$$$=$$ 7 $$\text {TeV}$$. The measurements are shown as a function of $$p_{\mathrm {T}}$$ and in several bins of rapidity. Calculations from POWHEG [152] (matched to PYTHIA [151]) and MC@NLO [47, 153] (matched to HERWIG [46]), are found to reproduce the data. Measurements from both lifetime- and lepton-based tagging methods are shown.

**Fig. 9:**
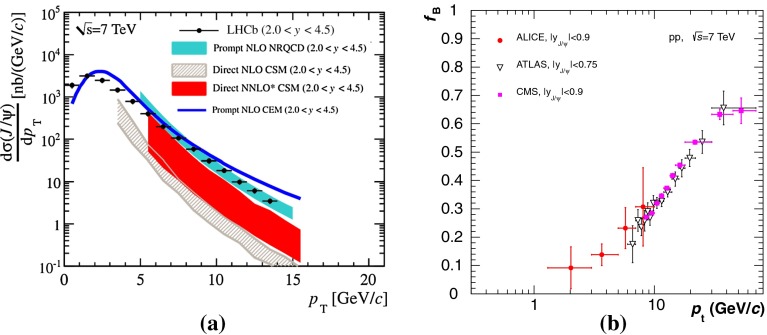
**a** Prompt $\mathrm {J}/\psi$ yield as measured by LHCb [172] at $\sqrt{s}$ $=$ 7 $\text {TeV}$ compared to different theory predictions referred to as “prompt NLO NRQCD”[173], “Direct NLO CS”[57, 58], “Direct NNLO$^\star$ CS” [61, 62] and “Prompt NLO CEM” [174]. **b** Fraction of $\mathrm {J}/\psi$ from B as measured by ALICE [108], ATLAS [170] and CMS [171] at $\sqrt{s}$ $=$ 7 $\text {TeV}$ in the central rapidity region

**Fig. 10:**
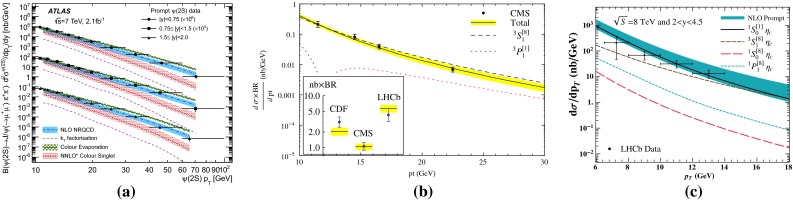
**a** ATLAS $\psi \text {(2S)}$ differential cross section [136] compared to different theoretical curves. **b** Prompt *X*(3872) production cross section measured by the CDF [175, 176], CMS [177], and LHCb [178] Collaborations compared with NLO NRQCD allowing the CS contribution to differ from that from HQSS [179]. **c** Prompt-$\eta _c$ transverse-momentum cross section in $\mathrm pp$ collisions at $\sqrt{s}$ $=$ 8 $\text {TeV}$ measured by LHCb [98] compared to the CS contribution following HQSS and fitted CO contributions at NLO [180]

#### Prompt charmonium

In this section, we show and discuss a selection of experimental measurements of prompt charmonium production at RHIC and LHC energies. We thus focus here on the production channels which do not involve beauty decays; these were discussed in the Sect. 2.2.3.

Historically, promptly produced $$\mathrm {J}/\psi $$ and $$\psi \text {(2S)} $$ have always been studied in the dilepton channels. Except for the PHENIX, STAR and ALICE experiments, the recent studies in fact only considered dimuons which offer a better signal-over-background ratio and a purer triggering. There are many recent experimental studies. In Fig. 9, we show only two of these. First we show $$\mathrm{d}\sigma /\mathrm{d}p_{\mathrm {T}} $$ for prompt $$\mathrm {J}/\psi $$ at $$\sqrt{s}$$$$=$$ 7$$~\text {GeV}$$ as measured by LHCb compared to a few predictions for the prompt yield from the CEM and from NRQCD at NLO^8^ as well as the direct yield^9^ compared to a NNLO$$^\star $$ CS evaluation. Our point here is to emphasise the precision of the data and to illustrate that at low and mid $$p_{\mathrm {T}}$$– which is the region where heavy-ion studies are carried out – none of the models can simply be ruled out owing to their theoretical uncertainties (heavy-quark mass, scales, non-perturbative parameters, unknown QCD and relativistic corrections, ...). Second, we show the fraction of $$\mathrm {J}/\psi $$ from $$b $$ decay for *y* close to 0 at $$\sqrt{s}$$$$=$$ 7 $$\text {TeV}$$ as function of $$p_{\mathrm {T}}$$ as measured by ALICE [108], ATLAS [170] and CMS [171]. At low $$p_{\mathrm {T}}$$, the difference between the inclusive and prompt yield should not exceed 10 % – from the determination of the $$\sigma _{{b\overline{b}}}$$, it is expected to be a few percent at RHIC energies [111]. It, however, steadily grows with $$p_{\mathrm {T}}$$. At the highest $$p_{\mathrm {T}}$$ reached at the LHC, the majority of the inclusive $$\mathrm {J}/\psi $$ is from $$b $$ decays. At $$p_{\mathrm {T}} \simeq $$ 10 $$~\text {GeV}$$, which could be reached in future quarkonium measurements in Pb–Pb collisions, it is already three times higher than at low $$p_{\mathrm {T}}$$: 1 $$\mathrm {J}/\psi $$ out of three comes from $$b $$ decays.

For excited states, there is an interesting alternative to the sole dilepton channel, namely $$\mathrm {J}/\psi \!+\!\pi \pi $$. This is particularly relevant since more than 50 % of the $$\psi \text {(2S)} $$ decay in this channel. The decay chain $$\psi \text {(2S)} \!\rightarrow \! \mathrm {J}/\psi +\pi \pi \!\rightarrow \! \mu ^{+}\mu ^{-} \!+\!\pi \pi $$ is four times more likely than $$\psi \text {(2S)} \rightarrow \mu ^{+}\mu ^{-} $$. The final state $$\mathrm {J}/\psi +\pi \pi $$ is also the one via which the *X*(3872) was first seen at $$\mathrm pp$$ colliders [175, 181]. ATLAS released [136] the most precise study to date of $$\psi \text {(2S)} $$ production up to $$p_{\mathrm {T}}$$ of 70$$~\text {GeV}$$ at $$\sqrt{s}$$$$=$$ 7 $$\text {TeV}$$, precisely in this channel. The measured differential cross section is shown for three rapidity intervals in Fig. 10a with four theoretical predictions. Along the same lines, the CDF, CMS and LHCb Collaborations measured the prompt *X*(3872) yields at different values of $$p_{\mathrm {T}}$$ (see Fig. 10b). In the NRQCD framework, these measurements tend to contradict [179] a possible assignment of the *X*(3872) as a radially excited *P*-wave state above the open-charm threshold. Such a statement should, however, be considered with care owing to the recurrent issues in understanding prompt quarkonium production. In addition, LHCb determined the *X*(3872) quantum numbers to be $$J^{PC}\!=\!1^{++}$$, excluding explanation of the *X*(3872) as a conventional $$\eta _{c2}(1^1 D_2)$$ state [182]. A brief survey of the new charmonium states above he $$D\bar{D}$$ threshold and their interpretation can be found in Ref. [131].

**Fig. 11:**
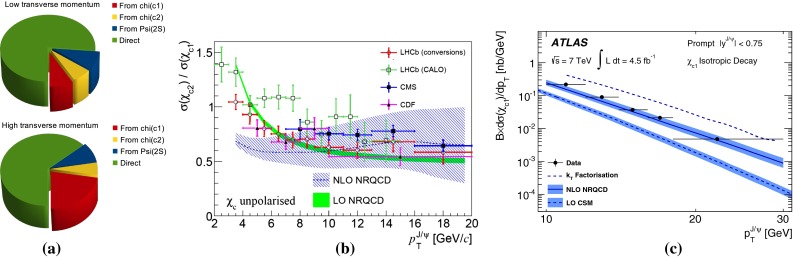
**a** Typical source of prompt $\mathrm {J}/\psi$ at low and high $p_{\mathrm {T}}$. **b** Ratio of $\chi _{\mathrm{c}1}$ to $\chi _{\mathrm{c}2}$ as measured by the LHCb experiment in $\mathrm pp$  collisions at $\sqrt{s}$ $=$ 7 $\text {TeV}$ compared to results from other experiments [185, 190, 191] and NRQCD calculations [189, 192]. **c** $\chi _{\mathrm{c}1}$ $\mathrm{d}\sigma /\mathrm{d}p_{\mathrm {T}}$ [137] as compared to LO CSM^10^, NLO NRQCD [173, 189, 193] and $k_{\mathrm {T}}$ factorisation [194, 195]

Ultimately the best channel to look at all $n=1$ charmonium yields at once is that of baryon-antibaryon decay. Indeed, all $n=1$ charmonia can decay in this channel with a similar branching ratio, which is small, i.e.  on the order of $10^{-3}$. LHCb is a pioneer in such a study with the first measurement of $\mathrm {J}/\psi$ into ${\mathrm{p}\overline{\mathrm{p}}}$, made along that of the $\eta _c$. The latter case is the first measurement of the inclusive production of the charmonium ground state. It indubitably opens a new era in the study of quarkonia at colliders. The resulting cross section is shown in Fig. 10c and was shown to bring about constraints [180, 183, 184] on the existing global fits of NRQCD LDMEs by virtue of heavy-quark spin symmetry (HQSS) which is an essential property of NRQCD. As for now, it seems that the CS contributions to $\eta _c$ are large – if not dominant – in the region covered by the LHCb data and the different CO have to cancel each others not to overshoot the measured yield. The canonical channel used to study $\chi _{c1,2}$ production at hadron colliders corresponds to the studies involving *P* waves decaying into $\mathrm {J}/\psi$ and a photon. Very recently the measurement of $\chi _{c0}$ relative yield was performed by LHCb [185] despite the very small branching ratio $\chi _{c0} \rightarrow \mathrm {J}/\psi +\gamma$ of the order of one percent, that is 30 (20) times smaller than that of $\chi _{c1}$ ($\chi _{c2}$). LHCb found that $\sigma (\chi _{c0})/\sigma (\chi _{c2})$ is compatible with unity for $p_{\mathrm {T}} >$4$~\text {GeV}/c$, in striking contradiction with statistical counting, 1/5.

Currently, the experimental studies are focusing on the ratio of the $\chi _{cJ}$ yields which are expected to be less sensitive to the photon acceptance determination. They bring about constraints on production mechanism but much less than the absolute cross section measurements which can also be converted into the fraction of $\mathrm {J}/\psi$ from $\chi _{cJ}$. This was the first measurement of this fraction at the Tevatron by CDF in 1997 [186] which confirmed that our understanding of quarkonium production at colliders was incorrect (for reviews see e.g.  [187, 188]). It showed that the $\mathrm {J}/\psi$ yield at Tevatron energies was mostly from *direct*$\mathrm {J}/\psi$ and *not* from $\chi _{cJ}$ decays. The latter fraction was found to be at most 30 %. Similar information are also fundamental to use charmonia as probes of QGP, especially for the interpretation of their possible sequential suppression. It is also very important to understand the evolution of such a fraction as function of $\sqrt{s}$, *y* and $p_{\mathrm {T}}$.

**Fig. 12:**
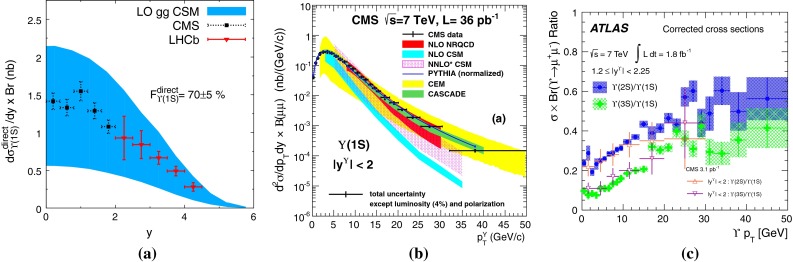
**a** $\Upsilon \text {(1S)}$ rapidity differential cross section as measured by ATLAS, CMS and LHCb [197, 198]. **b** Transverse momentum dependence of the $\Upsilon \text {(1S)}$ states as measured by CMS [198]. **c** Transverse momentum dependence of the $\Upsilon$ states ratio as measured by ATLAS [197]

Fig. 11a shows the typical size of the feed-down fraction of the $\chi _c$ and $\psi \text {(2S)}$ into $\mathrm {J}/\psi$ at low and high $p_{\mathrm {T}}$, which are different. One should therefore expect differences in these fraction between $p_{\mathrm {T}}$-integrated yields and yields measured at $p_{\mathrm {T}} = 10~\text {GeV}/c$ and above. Figure 11b shows the ratio of the $\chi _{c2}$ over $\chi _{c1}$ yields as measured^11^ at the LHC by LHCb, CMS and at the Tevatron by CDF. On the experimental side, the usage of the conversion method to detect the photon becomes an advantage. LHCb is able to carry out measurements down to $p_{\mathrm {T}}$ as small as 2$~\text {GeV}/c$, where the ratio seems to strongly increase. This increase is in line with the Landau–Yang theorem according to which $\chi _{c1}$ production from collinear and on-shell gluons at LO is forbidden. Such an increase appears in the LO NRQCD band, less in the NLO NRQCD one. At larger $p_{\mathrm {T}}$, such a measurement helps to fix the value of the NRQCD LDMEs (see the pioneering study of Ma et al. [189]). As we just discussed, once the photon reconstruction efficiencies and acceptance are known, one can derive the $\chi _c$ feed-down fractions which are of paramount importance to interpret inclusive $\mathrm {J}/\psi$ results. One can of course also derive absolute cross section measurements which are interesting to understand the production mechanism of the *P*-wave quarkonia per se; these may not be the same as that of *S*-wave quarkonia. Figure 11c shows the $p_{\mathrm {T}}$ dependence of the yield of the $\chi _{c1}$ measured by ATLAS (under the hypothesis of an isotropic decay), which is compared to predictions from the LO CSM,^12^ NLO NRQCD and $k_{\mathrm {T}}$ factorisation. The NLO NRQCD predictions, whose parameters have been fitted to reproduce the Tevatron measurement, is in good agreement with the data. Similar cross sections have been measured for the $\chi _{c2}$.

#### Bottomonium

The study of bottomonium production at LHC energies offers some advantages. First, there is no beauty feed-down. Second, owing to their larger masses, their decay products – usually leptons – are more energetic and more easily detectable (detector acceptance, trigger bandwidth, ...). Third, the existence of three sets of bottomonia with their principal quantum number $n=1,2,3$ below the open-beauty threshold offers a wider variety of states that can be detected in the dilepton decay channel – this, however, introduces a complicated feed-down pattern which we discuss later on. Fourth, at such high energies, their production rates with respect to those of charmonia are not necessarily much lower. It was for instance noticed [196] that, for their production in association with a *Z* boson, the cross sections are similar.

**Fig. 13:**
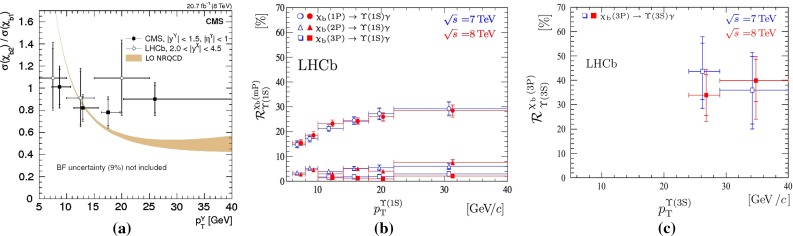
a Ratio of the production cross section of $\chi _{b2}$ and $\chi _{b1}$ in $\mathrm pp$ collisions at $\sqrt{s}$ $=$ 8 $\text {TeV}$  [201, 202]. **b** and **c** Fractions of $\chi _b$ to $\Upsilon \text {(1S)}$ as a function of $\Upsilon$ $p_{\mathrm {T}}$ [203]. For better visualisation the data points are slightly displaced from the bin centres. The *inner error bars* represent statistical uncertainties, while the *outer error bars* indicate statistical and systematic uncertainties added in quadrature

Fig. 12a shows the rapidity dependence of the $\Upsilon \text {(1S)}$ yield from two complementary measurements, one at forward rapidities by LHCb and the other at central rapidities by CMS (multiplied by the expected fraction of direct $\Upsilon \text {(1S)}$ as discussed below). These data are in line with the CS expectations; at least, they do not show an evident need for CO contributions, nor they exclude their presence. As for the charmonia, the understanding of their production mechanism for mid and high $p_{\mathrm {T}}$ is a challenge. Figure 12b shows a typical comparison with five theory bands. In general, LHC data are much more precise than theory. It is not clear that pushing the measurement to higher $p_{\mathrm {T}}$ would provide striking evidence in favour of one or another mechanism – associated-production channels, which we discuss in Sect. 2.4, are probably more promising. Figure 12c shows ratios of different *S*-wave bottomonium yields. These are clearly not constant as one might anticipate following the idea of the CEM. Simple mass effects through feed-down decays can induce an increase of these ratios [74, 199], but these are likely not sufficient to explain the observed trend if all the *direct* yields have the same $p_{\mathrm {T}}$ dependence. The $\chi _b$ feed-down, which we discuss in the following, can also affect these ratios.

Since the discovery of the $\chi _b(3P)$ by ATLAS [200], we know that the three $n=1,2,3$ families likely completely lie under the open-beauty threshold. This means, for instance, that we should not only care about $mS\rightarrow nS$ and $nP\rightarrow nS+\gamma$ transitions but also of $mP\rightarrow nS+\gamma$ ones. Obviously, the $n=1$ family is the better known of the three. Figure 13a shows the ratio of the production cross section of $\chi _{b2}(1P)$ over that of $\chi _{b1}(1P)$ measured by CMS and LHCb. Although the experimental uncertainties are significant, one does not observe the same trend as the LO NRQCD, i.e. an increase at low $p_{\mathrm {T}}$ due to the Landau–Yang theorem. Besides, the ratio is close to unity which also seems to be in contradiction to the simple spin-state counting.

**Fig. 14:**
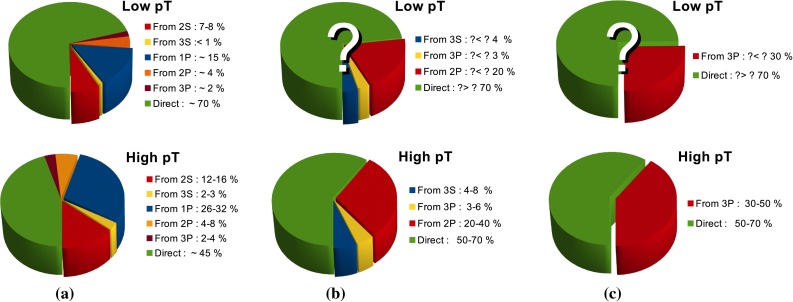
Typical sources of $\Upsilon (nS)$ at low and high $p_{\mathrm {T}}$. These numbers are mostly derived from LHC measurements [197–199, 203–208] assuming an absence of a significant rapidity dependence. **a** $\Upsilon (1\mathrm{S})$; **b** $\Upsilon (2\mathrm{S})$; **c** $\Upsilon (3\mathrm{S})$

Recently, LHCb performed a thorough analysis [203] of all the possible $$mP\rightarrow nS+\gamma $$ transitions in the bottomonium system. These new measurements along with the precise measurements of $$\Upsilon \text {(2S)}$$ and $$\Upsilon \text {(3S)}$$$$p_{\mathrm {T}}$$-differential cross section show that the feed-down structure is quite different from that commonly accepted ten years ago based on the CDF measurement [209]. The latter, made for $$p_{\mathrm {T}} >8$$$$~\text {GeV}/c$$  [209], suggested that the $$\chi (nP)\rightarrow \Upsilon (1S) + \gamma $$ feed-down could be as large as 40 % (without excluding values of the order of 25 %) and that only 50 % of the $$\Upsilon \text {(1S)}$$ were direct. Based on the LHC results, one should rather say that, at low $$p_{\mathrm {T}}$$, where heavy-ion measurements are mostly carried out, 70 % of the $$\Upsilon \text {(1S)}$$ are direct; the second largest source is from $$\chi _b(1P)$$ – approximately two thirds from $$\chi _{b1}(1P)$$ and one third from $$\chi _{b2}(1P)$$ [201, 202]. At larger $$p_{\mathrm {T}}$$ (above 20$$~\text {GeV}/c$$, say), the current picture is similar to the old one, i.e.   less than half of the $$\Upsilon \text {(1S)}$$ are direct and each of the feed-down is nearly doubled. For the $$\Upsilon \text {(2S)}$$, there is no $$\chi _{b}(2P)\rightarrow \Upsilon (2S) + \gamma $$ measurement at $$p_{\mathrm {T}}$$ lower than 20$$~\text {GeV}/c$$. Above, it is measured to be about 30 % with an uncertainty of 10 %. The feed-down from $$\chi _{b}(3P)$$ is slightly lower than from $$\Upsilon \text {(3S)}$$. Taken together they may account for 10–15 % of the $$\Upsilon \text {(2S)}$$ yield. For the $$\Upsilon \text {(3S)}$$, the only existing measurement [203] is at large $$p_{\mathrm {T}}$$ and also shows (see Fig. 13c) a feed-down fraction of 40 % with a significant uncertainty (up to 15 %). The situation is schematically summarised on Fig. 14.

**Fig. 15:**
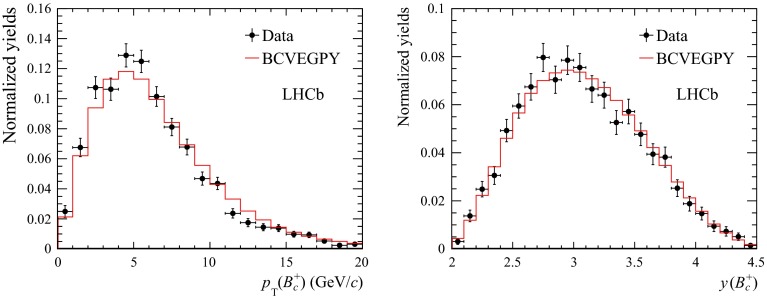
$\mathrm{B}_c^{+}$ meson production in $\mathrm pp$ collisions at $\sqrt{s}$ $=$ 8 $\text {TeV}$ as measured by the LHCb Collaboration in its $\mathrm{B}_c^{+} \rightarrow \mathrm {J}/\psi \ \pi ^{+}$ decay [210] within $0 < p_{\mathrm {T}} < 20$ $~\text {GeV}/c$ and $2.0 < y < 4.5$. The solid histogram is a theory evaluation based on the complete order-$\alpha _s^4$ calculation – as opposed to fragmentation-function-based computations – implemented in the $\mathrm{B}_c$ generator BCVEGPY [211, 212]

#### $\mathrm{B}_c$ and multiple-charm baryons

After a discovery phase during which the measurement of the mass and the lifetime of the $\mathrm{B}_c$ was the priority, the first measurement of the $p_{\mathrm {T}}$ and *y* spectra of promptly produced $\mathrm{B}_c^{+}$ was carried out by the LHCb Collaboration [210]. Unfortunately, as for now, the branching $\mathrm{B}_c^{+} \rightarrow \mathrm {J}/\psi \ \pi ^{+}$ is not yet known. This precludes the extraction of $\sigma _{\mathrm{pp}\rightarrow \mathrm{B}_c^{+} +X}$ and the comparison with the existing theoretical predictions [213–220]. Aside from this normalisation issue, the $p_{\mathrm {T}}$ and *y* spectra are well reproduced by the theory (see a comparison in Fig. 15 with BCVEGPY [211, 212], which is based on NRQCD where the CS contribution is dominant).

Searches for doubly charmed baryons are being carried out (see e.g.  [221]) on the existing data sample collected in $\mathrm pp$  collisions at 7 and 8 $\text {TeV}$. As for now, no analysis could confirm the signals seen by the fixed-target experiment SELEX at Fermilab [222, 223].

### Quarkonium-polarisation studies

Measurements of quarkonium polarisation can shed more light on the long-standing puzzle of the quarkonium hadroproduction. Various models of the quarkonium production, described in the previous Sect. 2.1.2, are in reasonable agreement with the cross section measurements but they usually fail to describe the measured polarisation.

We have collected in this section all results of polarisation measurements performed by different experiments at different collision energies $\sqrt{s_{\mathrm{NN}}}$ and in different kinematic regions. The results for $\mathrm {J}/\psi$ and $\psi \text {(2S)}$ can be found in Tables 1 and 2 for $\mathrm pp$ and p–A collisions. Since there is no known mechanism that would change quarkonium polarisation from proton–proton to proton–nucleus collisions, results from p–A collisions are also shown in this section. Tables 3, 4 and  5 gather the results for, respectively, the $\Upsilon \text {(1S)}$, $\Upsilon \text {(2S)}$ and $\Upsilon \text {(3S)}$ in $\mathrm pp$ collisions.

Polarisation of a vector quarkonium state is analysed experimentally via the angular distribution of the leptons from the quarkonium dilepton decay, that is parametrised by:

11 $$\begin{aligned}&\frac{\mathrm {d}^{2}N}{\mathrm {d}(\cos \theta )\mathrm {d}\phi } \propto 1+\lambda _\theta \cos ^2\theta + \lambda _\phi \sin ^2\theta \cos 2\phi \nonumber \\&\qquad \qquad \qquad \quad +\, \lambda _{\theta \phi }\sin 2\theta \cos \phi , \end{aligned}$$

$\theta$ is the polar angle between the positive lepton in the quarkonium rest frame and the chosen polarisation axis and $\phi$ angle is the corresponding azimuthal angle defined with respect to the plane of colliding hadrons. The angular decay coefficients, $\lambda _\theta$, $\lambda _\phi$ and $\lambda _{\theta \phi }$, are the polarisation parameters. In the case of an unpolarised yield, one would have ($\lambda _{\theta }, \lambda _{\phi }, \lambda _{\theta \phi }) = (0,0,0)$ for an isotropic decay angular distribution, whereas (1, 0, 0) and $(-1,0,0)$ refer to fully transverse and fully longitudinal polarisation, respectively.

**Table 1:** World existing data for $\mathrm {J}/\psi$ polarisation in $\mathrm pp$ and p–A collisions

$\sqrt{s_{\mathrm{NN}}}$ (GeV)	Colliding system	Experiment	$y$ range	$p_{\mathrm {T}}$ range (GeV/*c*)	Feed-down	Fit	Measured parameter(s)	Observed trend
200	$\mathrm pp$	PHENIX [238]	$\vert y \vert <$ 0.35	0–5	*B*: $2\div 15~\%$ [239], $\chi _c$: $23\div 41~\%$ [240], $\psi \text {(2S)}$: $5\div 20~\%$ [240]	1-D	$\lambda _{\theta }$ vs $p_{\mathrm {T}}$ in HX & GJ	$\lambda _{\theta }$ values from slightly positive (consistent with 0) to negative as $p_{\mathrm {T}}$ increases
200	$\mathrm pp$	STAR [241]	$\vert y \vert <$ 1	2–6	*B*: $2\div 15~\%$ [239], $\chi _c$: $23\div 41~\%$ [240], $\psi \text {(2S)}$: $5\div 20~\%$ [240]	1-D	$\lambda _{\theta }$ vs $p_{\mathrm {T}}$ in HX	$\lambda _{\theta }$ values from slightly positive (consistent with 0) to negative as $p_{\mathrm {T}}$ increases
1800	${\mathrm{p}\overline{\mathrm{p}}}$	CDF [242]	$\vert y \vert <$ 0.6	4–20	$\chi _c$: $25\div 35~\%$ [186], $\psi \text {(2S)}$: $10\div 25~\%$ [243]	1-D	$\lambda _{\theta }$ vs $p_{\mathrm {T}}$ in HX	Small positive $\lambda _{\theta }$ at smaller $p_{\mathrm {T}}$ then for $p_{\mathrm {T}}$ $>$ 12 $~\text {GeV}$ trend towards negative values
1960	${\mathrm{p}\overline{\mathrm{p}}}$	CDF [244]	$\vert y \vert <$ 0.6	5–30	$\chi _c$: $25\div 35~\%$ [186], $\psi \text {(2S)}$: $10\div 25\%$ [243]	1-D	$\lambda _{\theta }$ vs $p_{\mathrm {T}}$ in HX	$\lambda _{\theta }$ values from 0 to negative as $p_{\mathrm {T}}$ increases
7000	$\mathrm pp$	ALICE [245]	2.5–4.0	2–8	*B*: $10\div 30~\%$ [108], $\chi _c$: $15\div 30~\%$ [246], $\psi \text {(2S)}$: $8\div 20~\%$ [199]	1-D	$\lambda _{\theta }$, $\lambda _{\phi }$ vs $p_{\mathrm {T}}$ in HX & CS	$\lambda _{\theta }$ and $\lambda _{\phi }$ consistent with 0, with a possible hint for a longitudinal polarisation at low $p_{\mathrm {T}}$ in the *HX* frame
7000	$\mathrm pp$	LHCb [229]	2.0–4.5	2–15	$\chi _c$: $15\div 30~\%$ [246], $\psi \text {(2S)}$: $8\div 25~\%$ [247]	2-D	$\lambda _{\theta }$, $\lambda _{\phi }$, $\lambda _{\theta \phi }$ vs $p_{\mathrm {T}}$ and *y* in HX & CS	$\lambda _{\phi }$ and $\lambda _{\theta \phi }$ consistent with 0 in the *HX* frame and $\lambda _{\theta }$ ($\lambda _{\theta } =$ $-0.145$ $\pm$ 0.027) shows small longitudinal polarisation; $\tilde{\lambda }$ in agreement in the *HX* and *CS* frames
7000	$\mathrm pp$	CMS [232]	$\vert y \vert <$ 1.2	14–70	$\chi _c$: $25\div 35~\%$, $\psi \text {(2S)}$: $15\div 20~\%$ [171]	2-D	$\lambda _{\theta }$, $\lambda _{\phi }$, $\lambda _{\theta \phi }$, $\tilde{\lambda }$ vs $p_{\mathrm {T}}$ and in 2 $\vert y \vert$ bins in HX,CS,PX	In the three frames, no evidence of large $|\lambda _{\theta }|$ anywhere; $\tilde{\lambda }$ in good agreement in all reference frames
17.2	p–A	NA60 [236]	0.28–0.78	–	*B*: $2\div 8~\%$, $\chi _c$: $25\div 40~\%$ [248], $\psi \text {(2S)}$: $7\div 10~\%$ [249]	1-D	$\lambda _{\theta }$, $\lambda _{\phi }$ vs. $p_{\mathrm {T}}$ in HX	$\lambda _{\theta }$ and $\lambda _{\phi }$ consistent with 0; slight increase of the $\lambda _{\theta }$ value with increasing $p_{\mathrm {T}}$, no $p_{\mathrm {T}}$ dependence $\lambda _{\phi }$
27.4	p–A	NA60 [236]	$-$0.17 to 0.33	–	*B*: $2\div 8~\%$, $\chi _c$: $25\div 40~\%$ [248], $\psi \text {(2S)}$: $7\div 10~\%$ [249]	1-D	$\lambda _{\theta }$, $\lambda _{\phi }$ vs $p_{\mathrm {T}}$ in HX	$\lambda _{\theta }$ and $\lambda _{\phi }$ consistent with 0, no $p_{\mathrm {T}}$ dependence observed
31.5	$p\mathrm {-Be}$	E672/E706 [250]	$x_{F}$: 0.00–0.60	0–10	*B*: $2\div 8~\%$, $\chi _c$: $25\div 40~\%$ [248], $\psi \text {(2S)}$: $7\div 10~\%$ [249]	1-D	$\lambda _{\theta }$ in GJ	$\lambda _{\theta } =$ 0.01 $\pm$ 0.12 $\pm$ 0.09, consistent with no polarisation
38.8	$p\mathrm {-Be}$	E672/E706 [250]	$x_{F}$: 0.00–0.60	0–10	*B*: $2\div 8~\%$, $\chi _c$: $25\div 40~\%$ [248], $\psi \text {(2S)}$: $7\div 10~\%$ [249]	1-D	$\lambda _{\theta }$ in GJ	$\lambda _{\theta } =$ $-0.11$ $\pm$ 0.12 $\pm$ 0.09, consistent with no polarisation
38.8	$p\mathrm {-Si}$	E771 [251]	$x_{F}$: $-0.05$ to 0.25	0–3.5	*B*: $2\div 8~\%$, $\chi _c$: $25\div 40~\%$ [248], $\psi \text {(2S)}$: $7\div 10~\%$ [249]	1-D	$\lambda _{\theta }$ in GJ	$\lambda _{\theta } =$ $-0.09$ $\pm$ 0.12, consistent with no polarisation
38.8	$p\mathrm {-Cu}$	E866/NuSea [252]	$x_{F}$: 0.25–0.9	0–4	$\chi _c$: $25\div 40~\%$ [248], $\psi \text {(2S)}$: $7\div 10~\%$ [249]	1-D	$\lambda _{\theta }$ vs $p_{\mathrm {T}}$ and $x_{F}$ in CS	$\lambda _{\theta }$ values from small positive to negative with increasing $x_{F}$, no significant $p_{\mathrm {T}}$ dependence observed
41.6	$p\mathrm {-C,W}$	HERA-B [253]	$x_{F}$: $-0.34$ to 0.14	0–5.4	$\chi _c$: $25\div 40~\%$ [248], $\psi \text {(2S)}$: $5\div 15~\%$ [254]	1-D	$\lambda _{\theta }$, $\lambda _{\phi }$, $\lambda _{\theta \phi }$ vs $p_{\mathrm {T}}$ and $x_{F}$ in HX, CS, GJ	$\lambda _{\theta }$ and $\lambda _{\phi }$ $<0$, $\lambda _{\theta }$ ($\lambda _{\phi }$) decrease (increase) with increasing $p_{\mathrm {T}}$; no strong $x_{F}$ dependence; for $p_{\mathrm {T}} > 1$ $~\text {GeV}/c$, $\lambda _{\theta ,\phi }$ depends on the frame: $\lambda ^\mathrm{CS}_{\theta }>\lambda ^\mathrm{HX}_{\theta }\simeq 0$ and $\lambda ^\mathrm{HX} _{\phi } \ne 0$

**Table 2:** World existing data for $\psi \text {(2S)}$ polarisation in $\mathrm pp$ collisions

$\sqrt{s}$ (GeV)	Colliding system	Experiment	$y$ range	$p_{\mathrm {T}}$ range (GeV/*c*)	Feed-down	Fit	Measured parameter(s)	Observed trend
1800	${\mathrm{p}\overline{\mathrm{p}}}$	CDF [242]	$\vert y \vert <$ 0.6	5.5–20	None	1-D	$\lambda _{\theta }$ vs $p_{T}$ in HX	$\lambda _{\theta }$ consistent with 0
1960	${\mathrm{p}\overline{\mathrm{p}}}$	CDF [244]	$\vert y \vert <$ 0.6	5–30	None	1-D	$\lambda _{\theta }$ vs $p_{T}$ in HX	$\lambda _{\theta }$ values vs $p_{\mathrm {T}}$ go from slightly positive to small negative
7000	$\mathrm pp$	LHCb [230]	2.0–4.5	3.5–15	None	2-D	$\lambda _{\theta }$, $\lambda _{\phi }$, $\lambda _{\theta \phi }$, $\tilde{\lambda }$ vs. $p_{\mathrm {T}}$ and 3 $\vert y \vert$ bins in HX, CS	No significant polarisation found, with an indication of small longitudinal polarisation – $\tilde{\lambda }$ is negative with no strong $p_{\mathrm {T}}$ and *y* dependence
7000	$\mathrm pp$	CMS [232]	$\vert y \vert <$ 1.5	14–50	None	2-D	$\lambda _{\theta }$, $\lambda _{\phi }$, $\lambda _{\theta \phi }$, $\tilde{\lambda }$ vs. $p_{\mathrm {T}}$ and in 3 $\vert y \vert$ bins in HX, CS, PX	Non of the three reference frames show evidence of large transverse or longitudinal polarisation, in the whole measured kinematic range; $\tilde{\lambda }$ in good agreement in all reference frames

**Table 3:** World existing data for $\Upsilon \text {(1S)}$ polarisation in $\mathrm pp$ collisions

$\sqrt{s}$ (GeV)	Colliding system	Experiment	$y$ range	$p_{\mathrm {T}}$ range (GeV/*c*)	Feed-down	Fit	Measured parameter(s)	Observed trend
1800	${\mathrm{p}\overline{\mathrm{p}}}$	CDF [255]	$\vert y \vert <$ 0.4	0–20	$\Upsilon \text {(2S)}$: $6\div 18~\%$ [209], $\Upsilon \text {(3S)}$: $0.4\div 1.4~\%$ [209], $\chi _{b}$: $30\div 45~\%$ [209]	1-D	$\lambda _{\theta }$ vs $p_{\mathrm {T}}$ in HX	$\lambda _{\theta }$ consistent with 0, no significant $p_{\mathrm {T}}$ dependence
1960	${\mathrm{p}\overline{\mathrm{p}}}$	CDF [256]	$\vert y \vert <$ 0.6	0–40	$\Upsilon \text {(2S)}$: $6\div 18~\%$ [209], $\Upsilon \text {(3S)}$: $0.4\div 1.4~\%$ [209], $\chi _{b}$: $30\div 45~\%$ [209]	2-D	$\lambda _{\theta }$, $\lambda _{\phi }$, $\lambda _{\theta \phi }$, $\tilde{\lambda }$ vs $p_{\mathrm {T}}$ in HX, CS	The angular distribution found to be nearly isotropic
1960	${\mathrm{p}\overline{\mathrm{p}}}$	D0 [257]	$\vert y \vert <$ 0.4	0–20	$\Upsilon \text {(2S)}$: $6\div 18~\%$ [209], $\Upsilon \text {(3S)}$: $0.4\div 1.4~\%$ [209], $\chi _{b}$: $30\div 45~\%$ [209]	1-D	$\lambda _{\theta }$ vs $p_{\mathrm {T}}$ in HX	Significant negative $\lambda _{\theta }$ at low $p_{\mathrm {T}}$ with decreasing magnitude as $p_{\mathrm {T}}$ increases
7000	$\mathrm pp$	CMS [233]	$\vert y \vert <$ 1.2	10–50	$\Upsilon \text {(2S)}$: $10\div 15~\%$, $\Upsilon \text {(3S)}$: $0.5\div 3~\%$ [198], $\chi _{b}$: $25\div 40~\%$ [203]	2-D	$\lambda _{\theta }$, $\lambda _{\phi }$, $\lambda _{\theta \phi }$, $\tilde{\lambda }$ vs $p_{\mathrm {T}}$ and in 2 $\vert y \vert$ bins in HX, CS, PX	No evidence of large transverse or longitudinal polarisation in the whole kinematic range in the three reference frames

**Table 4:** World existing data for $\Upsilon \text {(2S)}$ polarisation in $\mathrm pp$ collisions

$\sqrt{s}$ (GeV)	Colliding system	Experiment	$y$ range	$p_{\mathrm {T}}$ range (GeV/*c*)	Feed-down	Fit	Measured parameter(s)	Observed trend
1960	${\mathrm{p}\overline{\mathrm{p}}}$	D0 [257]	$\vert y \vert <$ 0.4	0–20	$\Upsilon \text {(3S)}$: $2.5\div 5~\%$ [198], $\chi _{b}$: $25\div 35~\%$ [203]	1-D	$\lambda _{\theta }$ vs $p_{\mathrm {T}}$ in HX	$\lambda _{\theta }$ consistent with zero at low $p_{\mathrm {T}}$, trend towards strong transverse polarisation at $p_{\mathrm {T}} >5$ $~\text {GeV}/c$
1960	${\mathrm{p}\overline{\mathrm{p}}}$	CDF [256]	$\vert y \vert <$ 0.6	0–40	$\Upsilon \text {(3S)}$: $2.5\div 5~\%$ [198], $\chi _{b}$: $25\div 35~\%$ [203]	2-D	$\lambda _{\theta }$, $\lambda _{\phi }$, $\lambda _{\theta \phi }$, $\tilde{\lambda }$ vs $p_{\mathrm {T}}$ in HX, CS	The angular distribution found to be nearly isotropic
7000	$\mathrm pp$	CMS [233]	$\vert y \vert <$ 1.2	10–50	$\Upsilon \text {(3S)}$: $2.5\div 5~\%$ [198], $\chi _{b}$: $25\div 35~\%$ [203]	2-D	$\lambda _{\theta }$, $\lambda _{\phi }$, $\lambda _{\theta \phi }$, $\tilde{\lambda }$ vs $p_{\mathrm {T}}$ and in 2 $\vert y \vert$ bins in HX, CS, PX	No evidence of large transverse or longitudinal polarisation in whole kinematic range in the three reference frames

**Table 5:** World existing data for $\Upsilon \text {(3S)}$ polarisation in $\mathrm pp$ collisions

$\sqrt{s}$ (GeV)	Colliding system	Experiment	$y$ range	$p_{\mathrm {T}}$ range (GeV/*c*)	Feed-down	Fit	Measured parameter(s)	Observed trend
1960	${\mathrm{p}\overline{\mathrm{p}}}$	CDF [256]	$\vert y \vert <$ 0.6	0–40	$\chi _{b}$: $30\div 50~\%$ [203]	2-D	$\lambda _{\theta }$, $\lambda _{\phi }$, $\lambda _{\theta \phi }$, $\tilde{\lambda }$ vs $p_{\mathrm {T}}$ in HX, CS	The angular distribution found to be nearly isotropic
7000	$\mathrm pp$	CMS [233]	$\vert y \vert <$ 1.2	10–50	$\chi _{b}$: $30\div 50~\%$ [203]	2-D	$\lambda _{\theta }$, $\lambda _{\phi }$, $\lambda _{\theta \phi }$, $\tilde{\lambda }$ vs $p_{\mathrm {T}}$ and in 2 $\vert y \vert$ bins in HX, CS, PX	No evidence of large transverse or longitudinal polarisation in whole kinematic range in the three reference frames

It is, however, very important to bear in mind that the angular distribution of Eq. (11) is frame dependent as the polarisation parameters. All experimental analyses have been carried in a few specific reference frames, essentially defined by their polarisation axis,^13^ namely: the helicity (*HX*) frame, the Collins–Soper (*CS*) [225] frame, the Gottfried–Jackson (*GJ*) [226] frame as well as the perpendicular helicity (*PX*) [227] frame.

**Fig. 16:**
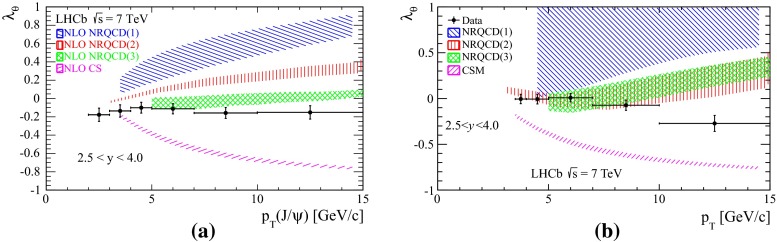
Polarisation parameter $\lambda _{\theta }$ for prompt $\mathrm {J}/\psi$  [229] (**a**) and $\psi \text {(2S)}$  [230] (**b**) from LHCb compared to different model predictions: direct NLO CSM [80] and three NLO NRQCD calculations [80–82], at $2.5<y<4.0$ in the helicity frame

In spite of the frame dependence of $\lambda _{\theta }, \lambda _{\phi }, \lambda _{\theta \phi }$, there exist some combinations which are frame invariant [224, 228]. An obvious one is the yield, another one is $\tilde{\lambda } = (\lambda _{\theta } + 3 \lambda _{\phi }) / (1 - \lambda _{\phi })$ [224]. As such, it can be used as a good cross-check between measurements done in different reference frames. Different methods have been used to extract the polarisation parameter(s) from the angular dependence of the yields. In the following, we divide them into two groups: *(i)* 1-D technique: fitting $\cos \theta$ distribution with the angular distribution, Eq. (11), averaged over the azimuthal $\phi$ angle, and fitting the $\phi$ distribution, Eq. (11), averaged over the polar $\theta$ angle (ii) 2-D technique: fitting a two-dimensional $\cos \theta$ vs $\phi$ distribution with the full angular distribution, Eq. (11).

Beyond the differences in the methods employed to extract these parameters, one should also take into consideration that some samples are cleaner than other ones, physics-wise.^14^ Indeed, as we discussed in the previous section, a given quarkonium yield can come from different sources, some of which are not of specific interests for data–theory comparisons. The most obvious one is the non-prompt charmonium yield, which is expected to be the result of quite different mechanism that the prompt yield. Nowadays, the majority of the studies are carried out on a prompt sample thanks to a precise vertexing of the events. Yet, a further complication also comes from feed-down from the excited states in which case vertexing is of no help. As for now, no attempt of removing it from e.g. prompt $\mathrm {J}/\psi$ and inclusive $\Upsilon \text {(1S)}$ samples has been made owing its intrinsic complication. We have therefore found it important to specify what kind of feed-down could be expected in the analysed sample.

In view of this, Tables 1, 2, 3, 4 and 5 contain, in addition to the information on the colliding systems and the kinematical coverages, information on the fit technique and a short reminder of the expected feed-down. For each measurement, we also briefly summarise the observed trend. The vast majority of the experimental data do not show a significant quarkonium polarisation, neither polar nor azimuthal anisotropy. Yet, values as large as $\pm 0.3$ are often not excluded either – given the experimental uncertainties. Despite these, a simultaneous description of both measured quarkonium cross sections and polarisations is still challenging for theoretical models of quarkonium hadroproduction.

As an example, we show in Fig. 16 the $$p_{\mathrm {T}}$$-dependence of $$\lambda _{\theta }$$ for prompt $$\mathrm {J}/\psi $$  [229] (left panel) and $$\psi \text {(2S)}$$  [230] (right panel) measured by LHCb at $$2.5<y<4.0$$ in the helicity frame compared with a few theoretical predictions. NLO NRQCD calculations [80–82] show mostly positive or zero values of $$\lambda _{\theta }$$ with a trend towards the transverse polarisation with increasing $$p_{\mathrm {T}}$$, and a magnitude of the $$\lambda _{\theta }$$ depending on the specific calculation and the kinematical region. On the other hand, NLO CSM models [59, 72] tend to predict an unpolarised yield at low $$p_{\mathrm {T}}$$ and an increasingly longitudinal yield ($$\lambda _{\theta } < 0$$) for increasing $$p_{\mathrm {T}}$$. None of these predictions correctly describes the measured $$\mathrm {J}/\psi $$ and $$\psi \text {(2S)}$$$$\lambda _{\theta }$$ parameters and their $$p_{\mathrm {T}}$$ trends. The NLO NRQCD fits of the PKU group [180, 231], however, open the possibility for an unpolarised *direct yield* but at the cost of not describing the world existing data in *e*p and $$e^{+}e^{-}$$ collisions and data in $$\mathrm pp$$ collisions for $$p_{\mathrm {T}} \le 5$$$$~\text {GeV}/c$$.

**Fig. 17:**
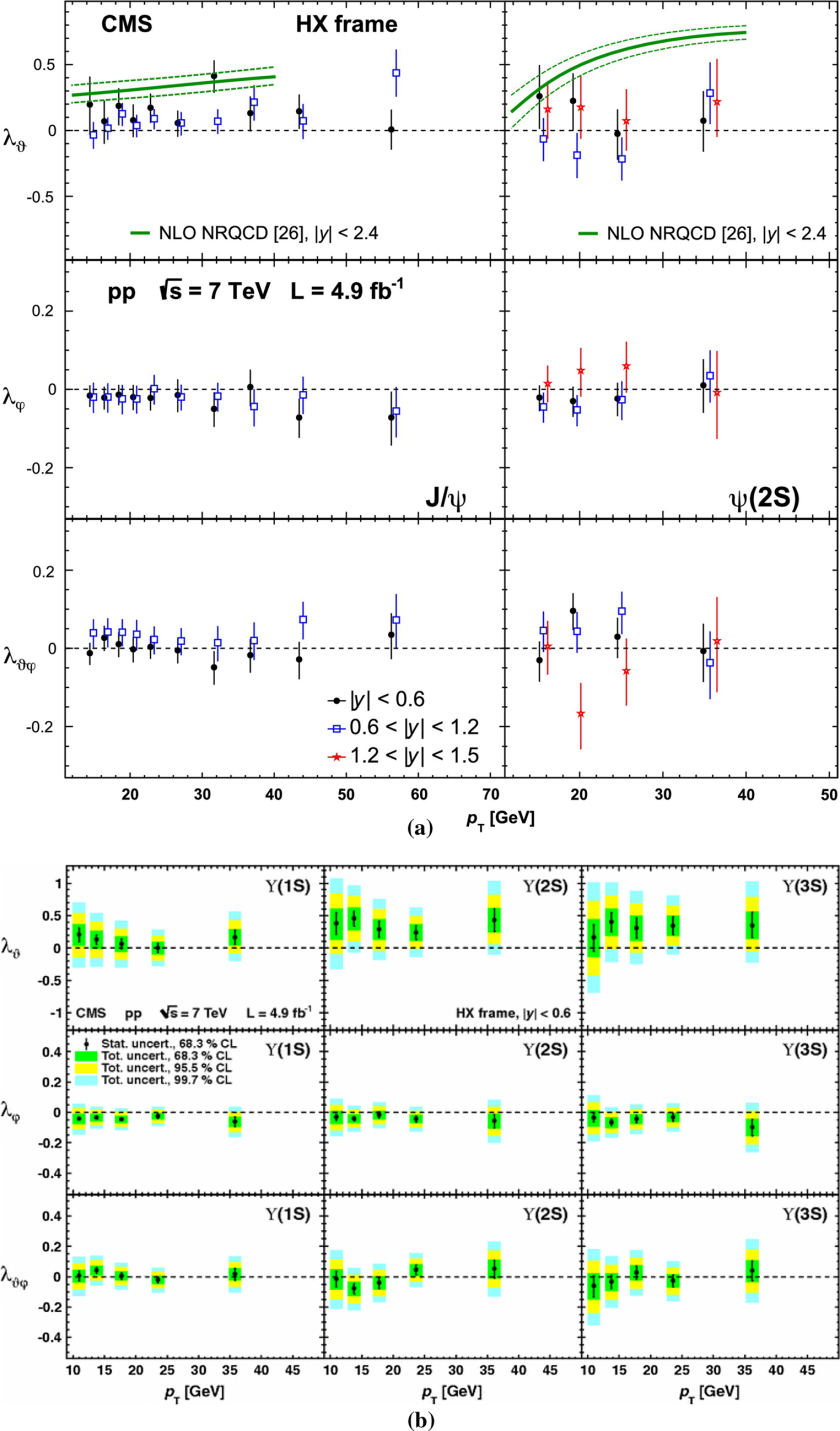
**a** Polarisation parameters, $\lambda _{\theta }$, $\lambda _{\phi }$ and $\lambda _{\theta \phi }$, as a function of $p_{\mathrm {T}}$ measured in the *HX* frame of prompt $\mathrm {J}/\psi$, $\psi \text {(2S)}$  [232]. *Upper panels* show also NLO NRQCD calculations [81] of $\lambda _{\theta }$ for prompt $\mathrm {J}/\psi$ and $\psi \text {(2S)}$ for $\vert y \vert <$ 2.4. **b** Polarisation parameters, $\lambda _{\theta }$, $\lambda _{\phi }$ and $\lambda _{\theta \phi }$, as a function of $p_{\mathrm {T}}$ measured by CMS in the *HX* frame of $\Upsilon \text {(1S)}$, $\Upsilon \text {(2S)}$, $\Upsilon \text {(3S)}$  [233] for $\vert y \vert < 0.6$

In order to illustrate the recent progress in these delicate studies, let us stress that LHC experiments have performed measurements of the three polarisation parameters as well as in different reference frames. This has not always been the case before by lack of statistics and of motivation since it is difficult to predict theoretically azimuthal effects, e.g. $\lambda _{\theta \phi }$. Figures 17a and b show CMS measurements of $\lambda _{\theta }$, $\lambda _{\phi }$ and $\lambda _{\theta \phi }$, in the *HX* frame for $\mathrm {J}/\psi$, $\psi \text {(2S)}$  [232] and $\Upsilon \text {(1S)}$, $\Upsilon \text {(2S)}$, $\Upsilon \text {(3S)}$  [233], respectively. CMS has also conducted polarisation measurements in the *CS* and *PX* frames, in addition to the *HX* frame and they could cross-check their analysis by obtaining the consistency in $\tilde{\lambda }$ in these three frames for different $p_{\mathrm {T}}$ and *y*. As for most of the previous measurements, no evidence of a large transverse or longitudinal quarkonium polarisation is observed in any reference frame, and in the whole measured kinematic range.

To conclude, let us also mention the importance of measuring the polarisation of *P*-wave states in order to refine our test of e.g. NRQCD [234]. This can be done either directly via the measurement of the angular dependence of the emitted photon or indirectly via that of the polarisation of the *S*-wave ($\mathrm {J}/\psi$ or $\Upsilon$) in which they decay [235]. Such studies are very important to constrain experimentally the effect of the feed-downs on the polarisation of the available samples. Let us also stress that such a measurement in heavy-ion collisions (along the line of the first study in In–In collisions [236]) may also be used as a tool to study a possible sequential suppression of the quarkonia [237].

### New observables

Thanks to the large heavy-flavour samples available at hadron colliders, studies of the production of open or hidden heavy-flavour production in association with another particle (light or heavy hadrons, quarkonium, or vector boson) are possible. The cross section of these processes is heavily sensitive to the particle production mechanisms and can help distinguishing between them. In addition, these final states can also results from multiple parton–parton interactions (or double-parton scatterings, DPS), where several hard parton-parton interactions occur in the same event [258–261]. Analogously, heavy-flavour-production dependence with the underlying event multiplicity brings information about their production mechanisms. A complete understanding of heavy-flavour production in hadronic collisions is mandatory to interpret heavy-flavour measurements in p–A and AA collisions, and disentangle cold (see Sect. 3) and hot (see Sects. 4 and 5) nuclear matter effects at play.

#### Production as a function of multiplicity

The correlation of open or hidden heavy-flavour yields with charged particles produced in hadronic collisions can provide insight into their production mechanism and into the interplay between hard and soft mechanisms in particle production. In high energy hadronic collisions, multiple parton–parton interactions may also affect heavy-flavour production [262, 263], in competition to a large amount of QCD-radiation associated to hard processes. In addition to these initial-state effects, heavy-flavour production could suffer from final-state effects due to the high multiplicity environment produced in high energy $\mathrm pp$ collisions [264, 265].

**Fig. 18:**
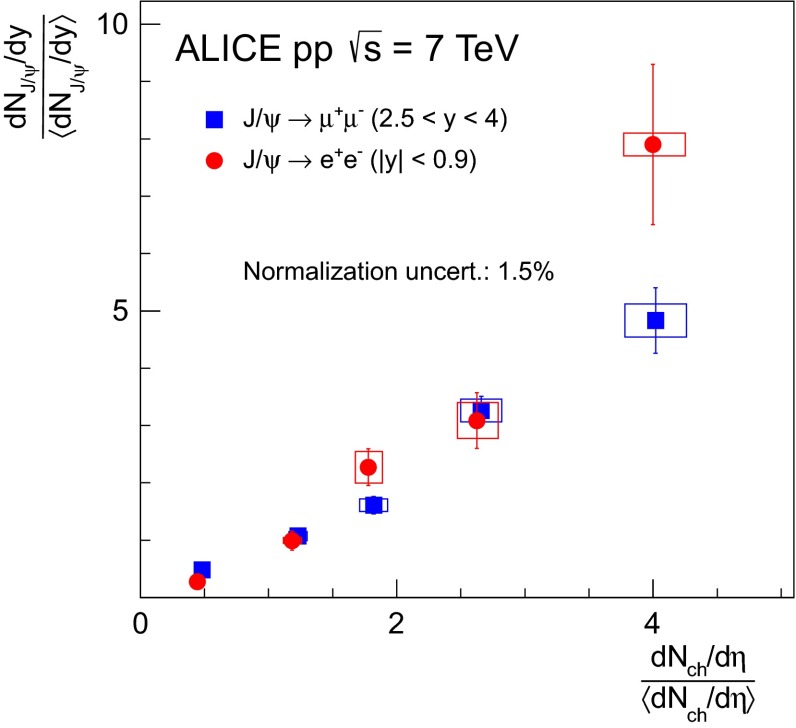
$$\mathrm {J}/\psi $$ yield as a function of the charged-particle density at mid-rapidity in $$\mathrm pp$$ collisions at $$\sqrt{s}$$ $$=$$ 7 $$\text {TeV}$$  [266]. Both the yields at forward- ($$\mathrm {J}/\psi \rightarrow \mu ^{+}\mu ^{-} $$, $$2.5 < y < 4$$) and at mid-rapidity ($$\mathrm {J}/\psi \rightarrow e^{+}e^{-} $$, $$|y| < $$ 0.9) are shown

At the LHC, $$\mathrm {J}/\psi $$ yields were measured as a function of the charged-particle density at mid-rapidity by the ALICE Collaboration in $$\mathrm pp$$ collisions at $$\sqrt{s}$$$$=$$ 7 $$\text {TeV}$$  [266]. Figure 18 shows the $$\mathrm {J}/\psi $$ yields at forward rapidity, studied via the dimuon-decay channel at $$2.5 < y < 4$$, and at mid-rapidity, analysed in its dielectron-decay channel at $$|y| < 0.9$$. The results at mid- and forward-rapidities are compatible within the measurement uncertainties, indicating similar correlations over three units of rapidity and up to four times the average charged-particle multiplicity. The relative $$\mathrm {J}/\psi $$ yield increases with the relative charged-particle multiplicity. This increase can be interpreted in terms of the hadronic activity accompanying $$\mathrm {J}/\psi $$ production, as well as in terms of parton-parton interactions, or in the percolation scenario [267].

**Fig. 19:**
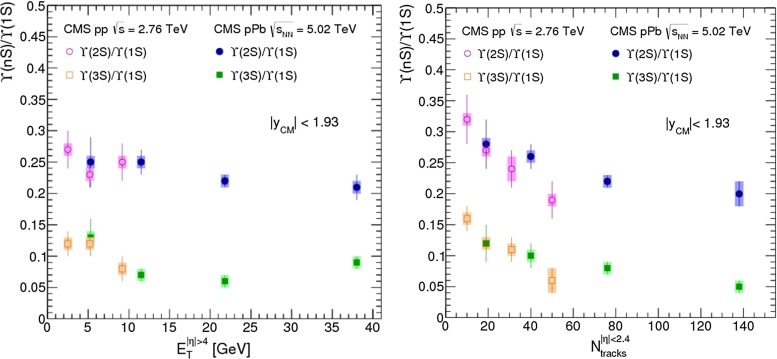
Cross section ratio of $\Upsilon \text {(2S)}/\Upsilon \text {(1S)}$ and $\Upsilon \text {(3S)}/\Upsilon \text {(1S)}$ for $|y| < 1.93$ as a function of the transverse energy ($E_\mathrm{T}$) measured in $4.0 < |\eta | < 5.2$ (*left*) and the number of charged tracks ($N_\mathrm{tracks}$) measured in $|\eta |<2.4$ (*right*), in $\mathrm pp$ collisions at $\sqrt{s}$ $=$ 2.76 $\text {TeV}$ (*open symbols*) and p–Pb collisions at $\sqrt{s_{\mathrm{NN}}}$ $=$ 5.02 $\text {TeV}$  (*filled symbols*) [268]

A similar study of the $$\Upsilon $$ yields was performed by the CMS Collaboration in $$\mathrm pp$$ collisions at $$\sqrt{s}$$$$=$$ 2.76 $$\text {TeV}$$  [268]. The self-normalised differential cross sections of $$\Upsilon \text {(1S)}/\langle \Upsilon \text {(1S)} \rangle $$, $$\Upsilon \text {(2S)}/\langle \Upsilon \text {(2S)} \rangle $$ and $$\Upsilon \text {(3S)}/\langle \Upsilon \text {(3S)} \rangle $$ at mid-rapidity are found to increase with the charged-particle multiplicity. To unveil possible variations of the different $$\Upsilon $$ states, the ratio of the $$\Upsilon \text {(2S)} $$ and $$\Upsilon \text {(3S)} $$ yields with respect to the $$\Upsilon \text {(1S)} $$ yield is shown in Fig. 19. The left figure presents the production cross section ratio as a function of the transverse energy ($$E_\mathrm{T}$$) measured in $$4.0 < |\eta | < 5.2$$, whereas the right figure shows the values as a function of the number of charged tracks ($$N_\mathrm{tracks}$$) measured in $$|\eta | < 2.4$$. The excited-to-ground-states cross section ratios seem independent of the event activity when they are evaluated as a function of $${E}_\mathrm{T}$$ measured in the forward-rapidity. Given the current experimental uncertainties, no strong conclusion can be drawn although these ratios seem to decrease with $$N_\mathrm{tracks}^{|\eta |<2.4}$$. The $$\Upsilon \text {(1S)}$$ is produced on average with two extra charged tracks than excited states. Feed-down contribution cannot solely explain the observed trend. If $$\Upsilon $$ states originated from the same initial partons, the mass difference between the ground and the excited states could generate extra particles produced with $$\Upsilon \text {(1S)}$$.

**Fig. 20:**
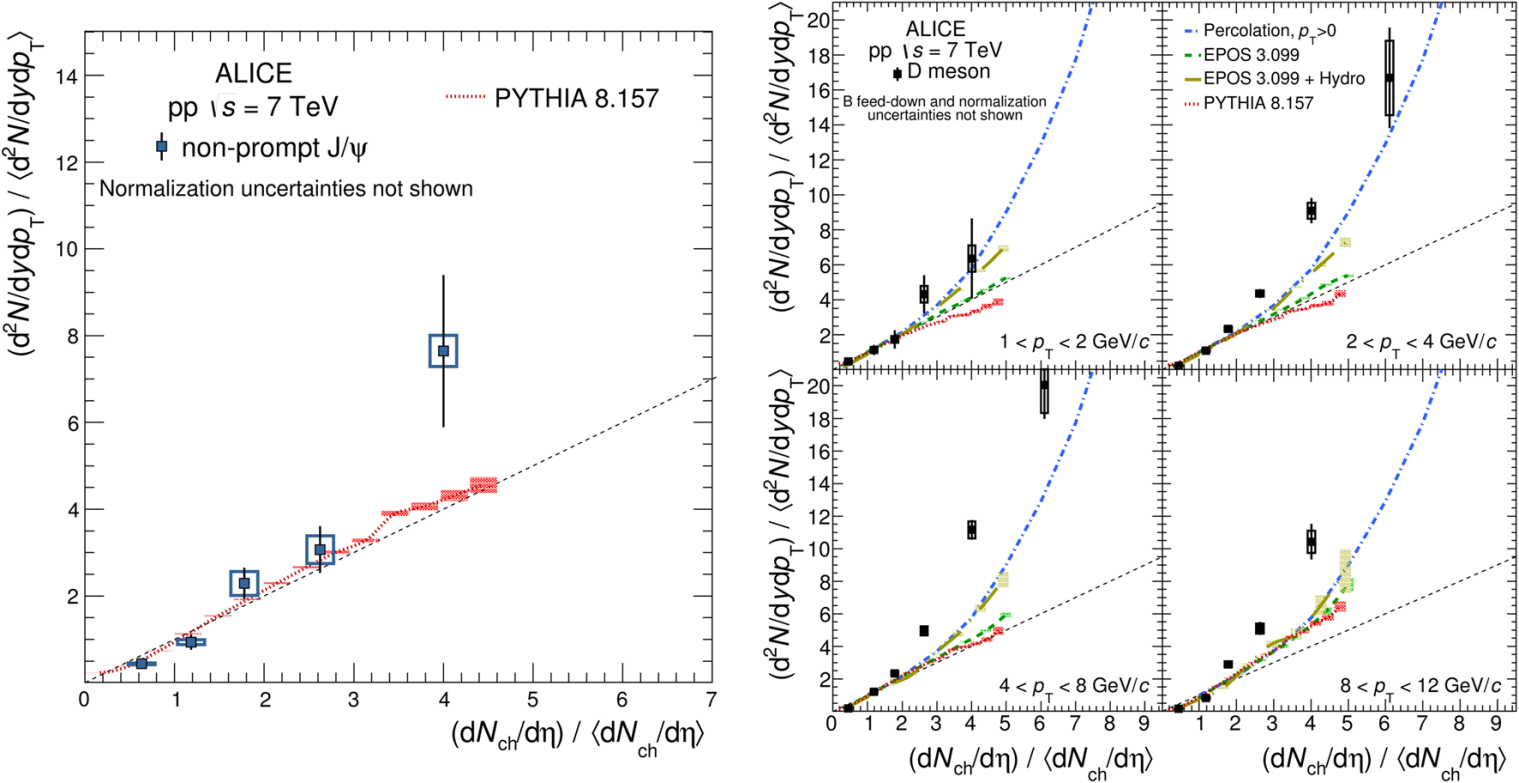
D-meson production (*left*) and non-prompt $\mathrm {J}/\psi$ (*right*) as a function of charged-particle multiplicity in $\mathrm pp$ collisions at $\sqrt{s}$ $=$ 7 $\text {TeV}$  [269] compared to PYTHIA 8 [45, 151], EPOS 3 [271, 272] and the percolation scenario [267, 270]

The measurement of open heavy-flavour production (via D mesons and non-prompt $$\mathrm {J}/\psi $$) as a function of charged-particle multiplicity at mid-rapidity in $$\mathrm pp$$ collisions at $$\sqrt{s}$$$$=$$ 7 $$\text {TeV}$$ was recently carried out by the ALICE Collaboration [269]. Figure 20 (left) presents the results for D mesons in four $$p_{\mathrm {T}}$$ bins compared to the percolation scenario [267, 270], EPOS 3 with or without hydro [271, 272] and PYTHIA 8 simulations [45, 151]. D-meson per-event yields are independent of $$p_{\mathrm {T}}$$ within the measurement uncertainties ($$1 < p_{\mathrm {T}} < 12~\text {GeV}/c $$) and increase with multiplicity faster than linearly at high multiplicities. Figure 20 (right) shows non-prompt $$\mathrm {J}/\psi $$ yields compared to PYTHIA 8 simulations. D-meson and non-prompt $$\mathrm {J}/\psi $$ yields present a similar increase with charged-particle multiplicity. The heavy-flavour relative yield enhancement as a function of the charged-particle multiplicity is qualitatively described by the percolation model, EPOS 3 and PYTHIA 8 for D mesons and PYTHIA 8 for non-prompt $$\mathrm {J}/\psi $$. However, the PYTHIA 8 event generator seems to underestimate the increase of heavy-flavour yields with the charged-particle multiplicity at high multiplicities. Open (D and non-prompt $$\mathrm {J}/\psi $$) and hidden (inclusive $$\mathrm {J}/\psi $$) heavy-flavour yields present a similar increase with the charged-particle multiplicity at mid-rapidity. This similarity suggests that the enhancement is likely related to heavy-quark-production mechanisms and is not significantly influenced by the hadronisation. It could be described by the hadronic activity associated to heavy-flavour production, multiple parton–parton interactions, or the percolation scenario [262, 264, 270].

Hidden and open heavy-flavour production measurements as a function of the event activity were initiated during the LHC Run 1 leading to unexpected results with impact on our understanding of the production mechanisms and the interpretation of p–Pb and Pb–Pb results. Run 2 data, with the increased centre-of-mass energy of 13 $\text {TeV}$ in $\mathrm pp$ collisions and larger luminosities, will allow one to reach higher multiplicities and to perform $p_{\mathrm {T}}$-differential studies of hidden and open heavy-flavour hadron production.

#### Associated production

Heavy-flavour azimuthal correlations in hadronic collisions allow for studies of heavy-quark fragmentation and jet structure at different collision energies, which help to constrain Monte Carlo models, and to understand the different production processes for heavy flavour. Heavy quarks can originate from flavour creation, flavour excitation, and parton shower or fragmentation processes of a gluon or a light (anti-)quark including gluon splitting [273]. These three different sources of the heavy-flavour production are expected to lead to different correlations between heavy quark and antiquark, and so a measurement of the opening angle in azimuth ($\Delta \phi$) of two heavy-flavour particles gives an access to different underlying production sub-processes. Azimuthal correlations arising from the flavour creation populate mostly the away-side ($\Delta \phi \approx \pi$), while the near-side region ($\Delta \phi \approx 0$) is sensitive to the presence of the flavour excitation and gluon splitting [273]. Since D–D and B–B correlation measurements are statistically demanding one can also look at angular correlations between heavy-flavour particles with charged hadrons (e.g. D–*h*) and correlations between electrons from heavy-flavour decays with charged (e.g. $e_{HF}$–*h*) or heavy-flavour hadrons (e.g. $e_{HF}$–D).

**Fig. 21:**
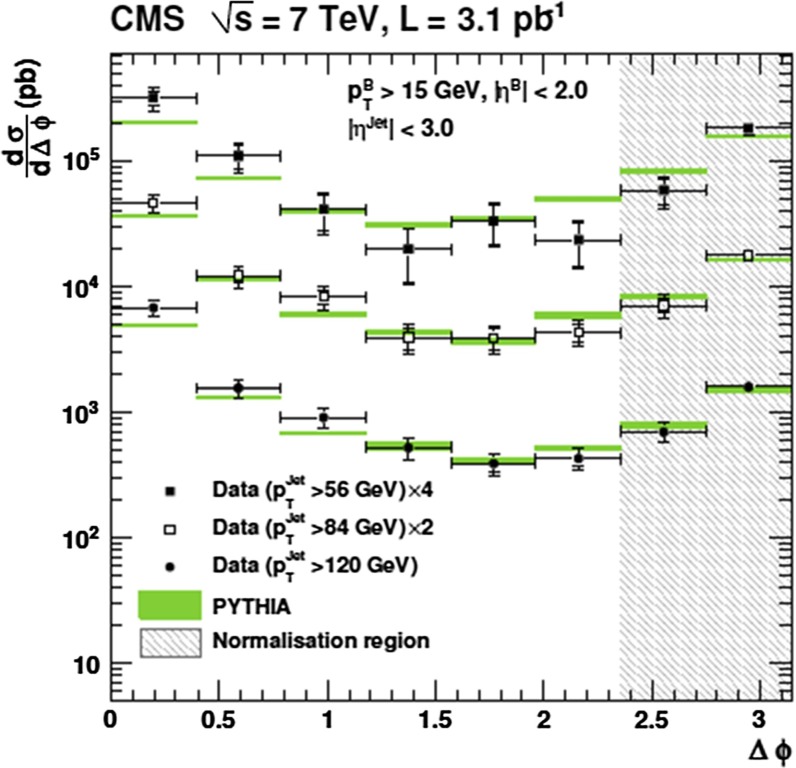
Azimuthal correlation of B–$\overline{\mathrm{B}}$ mesons measured by CMS in different ranges of the leading jet $p_{\mathrm {T}}$ and compared to PYTHIA [275]

Studies of heavy-flavour angular correlations in hadronic collisions were carried out at Tevatron with D–D correlations [124] in $${\mathrm{p}\overline{\mathrm{p}}} $$ collisions at $$\sqrt{s}$$$$=$$ 1.96 $$\text {TeV}$$ and at RHIC with *e*–$$\mu $$ correlations in $$\mathrm pp$$ collisions at $$\sqrt{s}$$$$=$$ 0.2 $$\text {TeV}$$  [114], where electrons and muons come from heavy-flavour decays and have a large $$\eta $$ gap. Results on heavy-flavour correlation measurements were also reported by the LHC experiments [150, 274, 275] with $$\mathrm pp$$ collisions at $$\sqrt{s}$$$$=$$ 7 $$\text {TeV}$$, as shown in Fig. 21. The LHC measurements of the azimuthal correlations between charm (beauty) and anti-charm (anti-beauty) hadrons (see e.g.  [274, 275]) show an enhancement at small $$\vert \Delta \phi \vert $$, not reproduced by PYTHIA, pointing to the importance of the near production (via the gluon splitting mechanism) in addition to the back-to-back production (mostly via flavour creation). At RHIC, the comparison of the *e*–$$\mu $$ azimuthal correlations [114] with PYTHIA suggests that 32 % of *e*–$$\mu $$ pairs are from the gluon fusion, which agrees with the charm production expectation [64]. These *e*–$$\mu $$ correlations show a peak at $$\Delta \phi = \pi $$ dominated by LO gluon process while the observed continuum is from higher-order contributions, like flavour excitation and gluonsplitting.

**Fig. 22:**
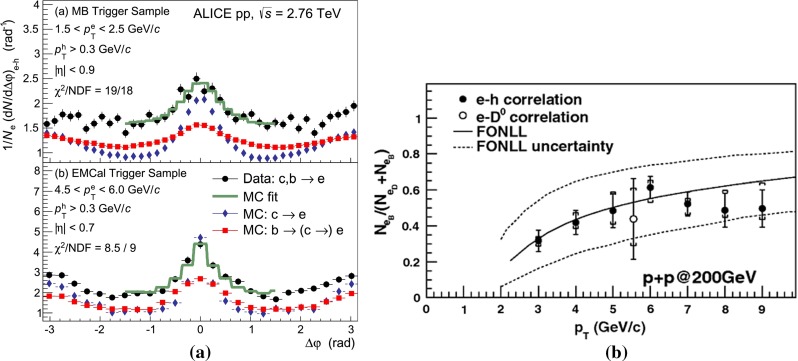
**a** Angular correlations between heavy-flavour decay electrons and charged hadrons measured by ALICE in $\mathrm pp$ collisions at $\sqrt{s} = 2.76$ $\text {TeV}$, compared to PYTHIA [107]. **b** Relative beauty contribution to the heavy-flavour electron yield measured by STAR in $\mathrm pp$ collisions at $\sqrt{s} = 0.2$ $\text {TeV}$, compared to FONLL calculations [115]

In addition to providing information on the heavy-flavour production mechanisms, the azimuthal correlations of heavy-flavour hadrons with light particles allow one to extract the relative contribution of charm and beauty hadron decays to the heavy-hadron yields. Due to the different decay kinematics, the azimuthal distribution of the particles produced from B-hadron decays presents a wider distribution at $$\Delta \phi \approx 0$$ than the one for D decays. The $$e_{HF}$$–*h* angular correlations were measured at mid-rapidity in $$\mathrm pp$$ collisions at $$\sqrt{s} = 200$$$$~\text {GeV}$$  [111, 113, 115] and at $$\sqrt{s} = 2.76$$ $$\text {TeV}$$  [107]. Figure 22a presents the azimuthal correlation of $$e_{HF}$$–h at the LHC. PYTHIA calculations of the D and B decay contributions are also shown. The contribution of beauty decays to the heavy-flavour electron yield increases with $$p_{\mathrm {T}} $$ and is described by FONLL pQCD calculations, both at $$\sqrt{s} =200$$$$~\text {GeV}$$ and at $$\sqrt{s} = 2.76$$ $$\text {TeV}$$ (see Fig. 22 [107, 115]). The beauty contribution to heavy-flavour electron yields becomes as important as the charm one at $$p_{\mathrm {T}} \sim 5$$$$~\text {GeV}/c$$. The results of $$e_{HF}$$ – $$\mathrm {D}^{0} $$ angular correlations at $$\sqrt{s} = 200$$$$~\text {GeV}$$ are consistent with the $$e_{HF}$$–h ones [115]; see Fig. 22b.

At the LHC, the preliminary results of D–h angular correlations in $\mathrm pp$ collisions at $\sqrt{s} = 7$ $\text {TeV}$ are described by various recent PYTHIA tunes [276]. Analogously, the azimuthal correlations of $\mathrm {J}/\psi$ with charged hadrons ($\mathrm {J}/\psi$–h) can be used to estimate beauty contribution to the inclusive $\mathrm {J}/\psi$ production [239, 277]. The near-side $\mathrm {J}/\psi$–h azimuthal correlations originate mostly from non-prompt $\mathrm {J}/\psi$ coming from B-meson decays, $\mathrm{B} \rightarrow \mathrm {J}/\psi + X$.

**Fig. 23:**
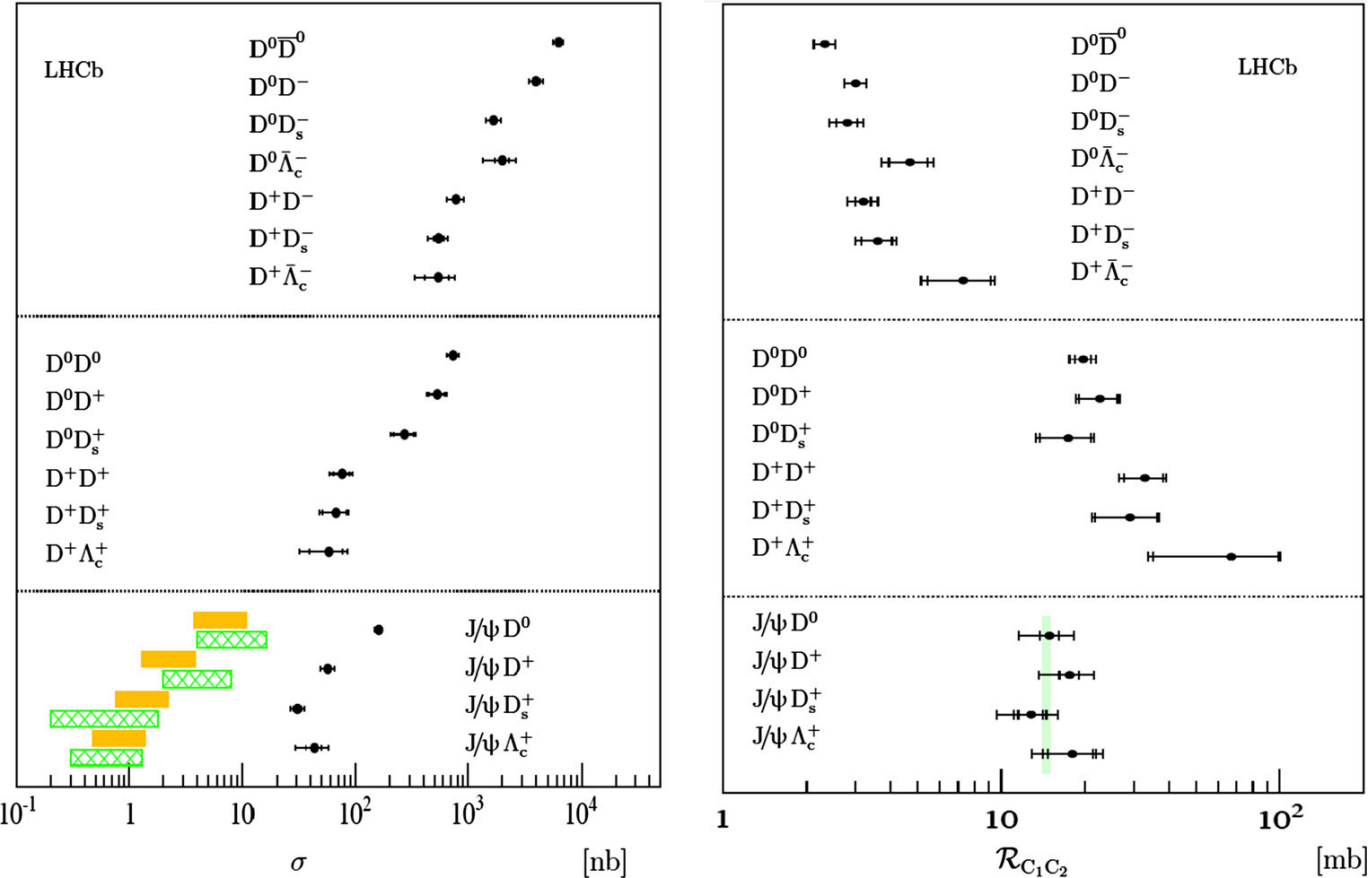
*Left* cross sections for double open charm hadron production (*top*) and open charm hadron plus $\mathrm {J}/\psi$ meson (*bottom*) in $\mathrm pp$ collisions at $\sqrt{s} =7$ TeV. *Right* measurement of $R_{C_1 \, C_2}$ for double open charm hadron production (*top*) and open charm hadron plus $\mathrm {J}/\psi$ meson (*bottom*) [274]

Recent experimental analyses of associated heavy-flavour production include the measurements of:

1. double $\mathrm {J}/\psi$ production at LHCb [278], D0 [279] and CMS [280],
2. open charm hadron plus a $\mathrm {J}/\psi$ or another open charm hadron at LHCb [274],
3. open charm meson or jet plus a Z boson at LHCb [281] and D0 [282],
4. open charm hadron plus a W boson at CMS [283],
5. $\mathrm {J}/\psi$ and W production at ATLAS [284],
6. $\mathrm {J}/\psi$ and Z production at ATLAS [285],
7. open beauty hadron or jet plus a Z boson at CDF [286] and D0 [287], and at ATLAS [288], CMS [289] and LHCb [290],
8. the search of production of $\Upsilon (1S)$ associated with W or Z production at CDF [291],
9. the search of the exclusive decay of $H^0$ into $\mathrm {J}/\psi +\gamma$ and $\Upsilon +\gamma$ [292].

The measurements of $\mathrm {J}/\psi$ plus open charm hadron and of double open charm hadron cross sections are summarised in Fig. 23 (left). The measurements of the production associated with a $\mathrm {J}/\psi$ are compared to two computations of the cross sections shown as green hatched areas [293] and yellow shaded areas [62]. These are calculations of charm production in the hard-scattering process of the collision, and underestimate by one order of magnitude the measured cross sections. This suggests that a large contribution to double charm production arises from double-parton scatterings (DPS) where both scatterings involve charm production. Therefore, in addition to providing useful information on the quarkonium-production mechanisms, associate-quarkonium-production observables can also be a rich source of information to understand the physics underlying DPS.

This is also supported by the measurement of the ratio of the double and inclusive production cross sections, defined as $R_{C_1 \, C_2}=\alpha ( \sigma _{C_1} \sigma _{C_2} / \sigma _{C_1C_2} )$, where $\alpha = 1/4$ if $C_1$ and $C_2$ are identical and non-self-conjugate, $\alpha = 1$ if $C_1$ and $C_2$ are different and either $C_1$ or $C_2$ is self-conjugate, and $\alpha = 1/2$ otherwise. This quantity, which would be equal to $\sigma _\mathrm{eff}$ in the case of a pure DPS yield, was evaluated by LHCb for the different aforementioned observed systems. These are plotted in Fig. 23 (right) and are compared, in the case of $\mathrm {J}/\psi +$ charm, to the results obtained from multi-jet events at the Tevatron, displayed by a green shaded area in the figure. They point at values close to 15 mb.

As regards $$\mathrm {J}/\psi $$-pair production, the cross section measured by LHCb in the region $$2<y<4.5$$ and $$0<p_{\mathrm {T}} <10$$$$~\text {GeV}/c$$ is [278]

12 $$\begin{aligned} \sigma _{\mathrm{pp} \rightarrow \mathrm {J}/\psi \, \mathrm {J}/\psi + X} = 5.1 \pm 1.0\,(\mathrm{stat.}) \pm 1.1\,(\mathrm{syst.})\,\mathrm{nb}, \end{aligned}$$

and was found to be in agreement with various theoretical models (e.g. dominated [294–297] or not [298–307] by DPS contributions). At this stage, the experimental and theoretical uncertainties both on the yield and the invariant-mass spectrum are certainly too large to draw any firm conclusion, as recently discussed in [306, 308].

However, double $\mathrm {J}/\psi$ production has recently been studied by D0 [279] and CMS [280] respectively at large rapidity separations and large transverse momenta. As for now, the D0 [279] study is the only one which really separated out the double- and single-parton-scattering contributions by using the yield dependence on the (pseudo)rapidity difference between the J$/\psi$ pair, $\Delta y$, an analysis which was first proposed in Ref. [294]. The DPS rapidity-separation spectrum is much broader and it dominates at large $\Delta y$. D0 has obtained the result that, in the region where DPS should dominate, the extracted value of $\sigma _\mathrm{eff}$ is on the order of 5  mb, that is significantly smaller than the values obtained with multi-jet events and $\mathrm {J}/\psi +$ charm as just discussed. At small rapidity separations, the usual single-parton-scattering (SPS) contribution is found to be dominant and the yield is well accounted for by the CSM at NLO [306–308]. CO contributions are only expected to matter at very large transverse momenta, in particular at large values of the smaller $p_{\mathrm {T}}$ of both $p_{\mathrm {T}}$ of each $\mathrm {J}/\psi$.

Such a small value of $\sigma _\mathrm{eff}$ (meaning a large DPS yield) has been shown to be supported by the CMS measurement [280] at 7 TeV which overshoots by orders of magnitude the NLO SPS predictions at large transverse momenta. Indeed, adding the DPS yield obtained with $\sigma _\mathrm{eff}=5$ mb solves [308] this apparent discrepancy first discussed in [307].

**Fig. 24:**
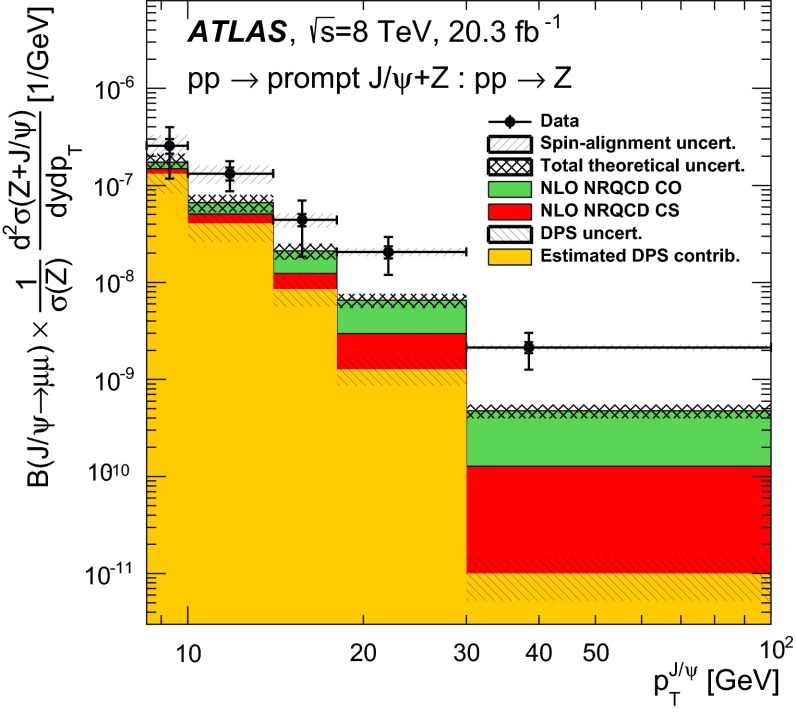
Production cross section of $\mathrm {J}/\psi$ mesons in association with a Z boson (normalised to that of a Z boson) as a function of the $\mathrm {J}/\psi$ $p_{\mathrm {T}}$ in $\mathrm pp$ collisions at $\sqrt{s} =8$ TeV [285] compared to CO and CS theoretical predictions [196, 309]

Finally, measurements of vector bosons, W and Z, associated with a heavy quark or with a $\mathrm {J}/\psi$ could also give access to the PDF as well as to DPS studies, in addition to providing complementary information on quarkonium production. As for now, both ATLAS measurements involving a $\mathrm {J}/\psi$ and a vector boson [284, 285] are difficult to interpret. It seems that the observed yields are systematically higher than the expectations from the DPS and SPS yields as shown for $\mathrm {J}/\psi +$Z in Fig. 24.

To summarise, the study of associated production of heavy quarks and heavy quarkonia has really taken off with the advent of the LHC and the analysis of the complete data sample taken at the Tevatron. There is no doubt that forthcoming studies will provide much more new information – and probably also puzzles – on the production of these particles. It is also probable that some of these observables at LHC energies are dominated by DPS contributions and, in such a case, specific nuclear dependences should be observed in proton–nucleus and nucleus–nucleus collisions (see e.g. [310, 311]).

### Summary and outlook

The LHC Run 1 provided a complete set of cross section and polarisation measurements in the charm and beauty sector in $\mathrm pp$ collisions at $\sqrt{s}$ = 2.76, 7 and 8 $\text {TeV}$, that can be summarised as follows:

- Heavy-flavour decay lepton $p_{\mathrm {T}}$- and $y$-differential production cross sections are well described by pQCD calculations.
- D-meson $$p_{\mathrm {T}}$$-differential cross sections are well described by pQCD calculations within uncertainties. FONLL and POWHEG central calculations tend to underestimate the data, whereas GM-VFNS tends to overestimate it. The $$\Lambda _c^{+}$$$$p_{\mathrm {T}}$$-differential cross section was measured up to 8$$~\text {GeV}/c$$ and is well described by GM-VFNS.
- The $p_{\mathrm {T}}$-differential cross section of charmonia from beauty decays (non-prompt $\mathrm {J}/\psi$, $\psi \text {(2S)}$, $\eta _c$, $\chi _{c1}$ and $\chi _{c2}$) at low to intermediate $p_{\mathrm {T}}$ is well described by pQCD calculations. At high $p_{\mathrm {T}}$ the predictions tend to overestimate the data. $p_{\mathrm {T}}$ and $y$-differential cross section measurements were performed for exclusive decays: $\mathrm {B}^{\pm }$, $\mathrm {B}^{0}$ and $\mathrm {B}^0_s$. *b*-jet cross section measurements are well described by pQCD calculations taking into account the matching between NLO calculations and parton showers.
- The $$\mathrm{B}_c^{+}$$$$p_{\mathrm {T}}$$ and $$y$$-differential cross section was for the first time measured at the LHC and it is well reproduced by theory.
- Prompt $\mathrm {J}/\psi$ and $\psi \text {(2S)}$ differential cross sections were measured, none of the tested models can be ruled out due to large theoretical uncertainties.
- $\Upsilon \text {(1S)}$ differential cross section description remains a challenge at mid and high $p_{\mathrm {T}}$, LHC data being more precise than theory.
- Quarkonium polarisation studies were performed in various reference frames for $\mathrm {J}/\psi$, $\psi \text {(2S)}$ and $\Upsilon$. At present, none of the models can describe all observed features.

In summary, open charm and beauty differential cross sections are globally well described by pQCD, although the theoretical uncertainties are quite large at low $p_{\mathrm {T}}$, especially in the case of charm production. On the other hand, quarkonium production mechanisms remain a puzzle, especially if one aims at describing the $p_{\mathrm {T}}$- and $y$-differential cross section and polarisation in the same framework, or predict low and high $p_{\mathrm {T}}$ quarkonium production. The comparison of data with model calculations is still limited by the theoretical uncertainties.

In addition to the $p_{\mathrm {T}}$- and $y$-differential production cross sections, the LHC Run 1 has allowed first measurements of heavy-flavour production versus charged-particle multiplicity, azimuthal angular correlations to charged-particles or heavy-flavour hadrons, and of associated heavy-flavour production, giving more insight into the production mechanisms. Those measurements can be summarised as follows:

- Inclusive $$\mathrm {J}/\psi $$ (at central and forward rapidity), prompt D meson and non-prompt $$\mathrm {J}/\psi $$ (at central rapidity) yields were measured at $$\sqrt{s}$$$$=$$ 7 $$\text {TeV}$$ versus charged-particle multiplicity. Heavy-flavour yields increase as a function of charged-particle multiplicity at mid-rapidity; D-meson results present a faster-than-linear increase at the highest multiplicities. Possible interpretations of these results are the contribution of multiple-parton interactions or the event activity accompanying heavy-flavour hadrons. The increase of the prompt D-meson yields is qualitatively reproduced by an hydrodynamic calculation with the EPOS event generator and the percolation scenario. The $$\Upsilon $$ measurement at $$\sqrt{s}$$$$=$$ 2.76 $$\text {TeV}$$ also presents an increase with charged-particle multiplicity but the decrease of the fraction of the $$\Upsilon \text {(nS)}$$ to the $$\Upsilon \text {(1S)}$$ state is at present not understood.
- Measurements of the azimuthal correlations between charm (beauty) and anti-charm (anti-beauty) point to the importance of the near production via the gluon splitting mechanism in addition to the back-to-back production.
- $\mathrm {J}/\psi$ plus open charm and double open charm hadron production cross section measurements suggest a non-negligible contribution of double-parton scatterings to double charm production. Measurements of vector boson production in association with a $\mathrm {J}/\psi$ provide further constraints to the model calculations.

The LHC Run 2 will provide more precise and more differential cross section measurements at the centre-of-mass energy $\sqrt{s} = 13$ $\text {TeV}$. This will provide strong constraints to the theoretical calculations and further understanding on the production mechanisms.

## Cold nuclear matter effects on heavy flavour and quarkonium production in proton–nucleus collisions

Characterising the hot and dense medium produced in heavy-ion (AA) collisions requires a quantitative understanding of the effects induced by the presence of nuclei in the initial-state, the so-called cold nuclear matter (CNM) effects. These effects can be studied in proton–nucleus (p–A) or deuteron–nucleus (d–A) collisions.^15^

A way to quantify CNM effects is to measure the nuclear modification factor $R_{\mathrm {pA}} ^\mathcal{C}$ of hard processes, defined as the ratio of their production yield $N_\mathrm{pA}^\mathcal{C}$ in p–A collisions (in a given centrality class $\mathcal{C}$) and their $\mathrm pp$ production cross section $\sigma _\mathrm{pp}$ at the same energy, scaled by the average nuclear overlap function ${\langle T_\mathrm{pA} \rangle }_\mathcal{C}$ (obtained with the Glauber model[312]),

13 $$\begin{aligned} R_{\mathrm {pA}} ^\mathcal{C} = \frac{N_\mathrm{pA}^\mathcal{C}}{{\langle T_\mathrm{pA} \rangle }_\mathcal{C}\ \sigma _\mathrm{pp}}. \end{aligned}$$

In “minimum-bias” p–A collisions (i.e. without a selection on centrality), $R_{\mathrm {pA}} ^\mathcal{C}$ reduces to

14 $$\begin{aligned} R_{\mathrm {pA}} = \frac{\sigma _\mathrm{pA}}{A\ \sigma _\mathrm{pp}}, \end{aligned}$$

where *A* is the mass number. The nuclear dependence of a centrality-integrated hard cross section p–A is sometimes parametrised by $\alpha$ defined as

15 $$\begin{aligned} \sigma _\mathrm{pA}=\sigma _\mathrm{pp}\,A^\alpha , \end{aligned}$$

In the absence of CNM effects, the p–A production is expected to be proportional to *A*, leading to $R_{\mathrm {pA}} =1$ and $\alpha =1$.

This section starts (Sect. 3.1) with a brief introduction to the physics of CNM effects on heavy flavour and with a compilation of available p–A data. Next, the different theoretical approaches are discussed in Sect. 3.2, before a review of recent RHIC and LHC experimental results in Sect. 3.3. Afterwards, the extrapolation of CNM effects from p–A to AA collisions is discussed in  Sect. 3.4, from both the theoretical and the experimental points of view. Finally, Sect. 3.5 includes a summary and a discussion of short-term perspectives.

### Heavy flavour in p–A collisions

Open and hidden heavy flavour production constitutes a sensitive probe of medium effects because heavy quarks are produced in hard processes in the early stage of the nucleus–nucleus collision. Open and hidden heavy-flavour production can be affected by the following CNM effects:

- *Modification of the effective partonic luminosity* in colliding nuclei, with respect to colliding protons. This effect is due to the different dynamics of partons within free protons with respect those in nucleons, mainly as a consequence of the larger resulting density of partons. These effects depend on *x* and on the scale of the parton–parton interaction $Q^2$ (the square of the four-momentum transfer). In collinearly factorised pQCD calculations the nuclear effects on the parton dynamics are described in terms of nuclear-modified PDFs (hereafter indicated as nPDF). Quite schematically three regimes can be identified for the nPDF to PDF ratio of parton flavour *i*, $R_i(x,Q^2)$, depending on the values of *x*: a depletion ($R_i<1$) – often referred to as *shadowing* and related to phase-space saturation – at small $x \lesssim 10^{-2}$, a possible enhancement $R_i>1$ (*anti-shadowing*) at intermediate values $10^{-2} \lesssim x \lesssim 10^{-1}$, and the EMC effect, a depletion taking place at large $x \gtrsim 10^{-1}$. The $R_i(x,Q^2)$ parametrisations are determined from a global fit analyses of lepton–nucleus and proton–nucleus data (see Sect. 3.2.2).
- The physics of *parton saturation* at small *x* can also be described within the *Colour Glass Condensate (CGC)* theoretical framework. Unlike the nPDF approach, which uses DGLAP linear evolution equations, the CGC framework is based on the Balitsky–Kovchegov or JIMWLK non-linear evolution equations (see Sect. 3.2.3).
- *Multiple scattering of partons* in the nucleus before and/or after the hard scattering, leading to parton energy-loss (either radiative or collisional) and transverse momentum broadening (known as the Cronin effect). In most approaches (see Sect. 3.2.4) it is characterised by the transport coefficient of cold nuclear matter, $\hat{q}$.
- Final-state inelastic interaction, or *nuclear absorption*, of ${Q\overline{Q}}$ bound states when passing through the nucleus. The important parameter of these calculations is the “absorption” (or break-up) cross section $\sigma _\mathrm {abs}$, namely the inelastic cross section of a heavy-quarkonium state with a nucleon.
- On top of the above genuine CNM effects, the large set of particles (partons or hadrons) produced in p–A collisions at high energy may be responsible for a modification of open heavy flavour or quarkonium production. It is still highly debated whether this set of particles could form a “medium” with some degree of collectivity. If this was the case, this medium could impart a flow to heavy-flavour hadrons. Moreover, heavy quarkonia can be dissociated by *comovers*, i.e., the partons or hadrons produced in the collision in the vicinity of the heavy-quarkonium state (see Sect. 3.2.5).

Assuming factorisation, and neglecting isospin effects, the hadroproduction cross section of a heavy-quark pair ${Q\overline{Q}}$ is given by

16 $$\begin{aligned}&\sigma _{\mathrm{pA}\rightarrow {Q\overline{Q}} +X}[\sqrt{s_{\mathrm{NN}}} ] = A \sum _{i,j} \int _0^1 \mathrm {d}x_i \int _0^1 \mathrm {d}x_j\,f_i^\mathrm{N}(x_i,\mu _F^2)\nonumber \\&\quad \times f_j^\mathrm{N}(x_j,\mu _F^2)\,\hat{\sigma }_{ij\rightarrow {Q\overline{Q}} +X}[x_i, x_j, \sqrt{s_{\mathrm{NN}}}, \mu _F^2,\mu _R^2], \end{aligned}$$

where $f_i^\mathrm{N}$ are the nucleon parton distributions, *i* (*j*) denotes all possible partons in the proton (nucleus) carrying a fraction $x_i$ ($x_j$) of the nucleon momentum, $\hat{\sigma }_{ij\rightarrow {Q\overline{Q}} +X}$ is the partonic cross section, $\sqrt{s_{\mathrm{NN}}}$ is the nucleon–nucleon centre-of-mass energy of the collision, and $\mu _F$ ($\mu _R$) is the factorisation (renormalisation) scale of the process. In high energy hadron collisions (especially at RHIC and LHC), heavy quarks are mainly produced by gluon fusion [138].

For a $2 \rightarrow 1$ partonic process giving a particle of mass *m*, at leading order there is a direct correspondence between the momentum fractions and the rapidity *y* of the outgoing particle in the nucleon–nucleon centre-of-mass (CM) frame,

17 $$\begin{aligned} x_1 = \frac{m}{\sqrt{s_{\mathrm{NN}}}}\exp (y)\quad \text {and}\quad x_2 = \frac{m}{\sqrt{s_{\mathrm{NN}}}}\exp (-y). \end{aligned}$$

For a $2 \rightarrow 2$ partonic process, the extra degree of freedom coming from the transverse momentum results in a less direct correspondence leading to the following useful relations:

18 $$\begin{aligned}&\text {open heavy-flavour (D and B mesons...)}\,\nonumber \\&\quad x_2\approx \frac{2 m_{\mathrm {T}}}{\sqrt{s_{\mathrm{NN}}}}\exp (-y), \end{aligned}$$

19 $$\begin{aligned}&\text {quarkonia} (\mathrm {J}/\psi , \Upsilon ...) \nonumber \\&x_2 \approx \frac{m_{\mathrm {T}} +p_{\mathrm {T}}}{\sqrt{s_{\mathrm{NN}}}}\exp (-y). \end{aligned}$$

where $$m_{\mathrm {T}} = \sqrt{m^2+p_{\mathrm {T}} ^2}$$ is the transverse mass of the outgoing particle of mass *m*, transverse momentum $$p_{\mathrm {T}} $$ and rapidity $$y $$ in the centre-of-mass frame. So, the typical resolution scale should be of the order of the transverse mass of the particle produced.

The typical range for the momentum fractions probed is therefore a function of both the acceptance of the detector (rapidity coverage) and the nature of the particles produced and their associated energy scale. Moreover, assuming different underlying partonic production processes can end up in average values of *x* that may differ from one another.

Studies of p–A collisions since 1980 were first performed on fixed-target experiments at SPS, Tevatron and HERA, and more recently at colliders, RHIC and LHC. Current available data are summarised in Table 6 for collider experiments and in Table 7 for fixed-target experiments. This section is focussed on the most recent results from the RHIC and LHC experiments, and their theoretical interpretation.

**Table 6:** Available p–A data in collider: the probes, the colliding system, $$\sqrt{s_{\mathrm{NN}}} $$, the kinematic range (with $$y $$ the rapidity in the centre-of-mass frame), the observables (as a function of variables) are given as well as the references

Probes	Colliding system	$\sqrt{s_{\mathrm{NN}}}$ (TeV)	$y$	Observables (variables)	References
PHENIX					
$\mathrm{HF} \rightarrow e^{\pm }$	d–Au	0.2	$|y |<0.35$	$R_{\mathrm {dAu}}$ ($p_{\mathrm {T}}$,$\mathrm {N_{coll}}$), $\langle p_{\mathrm {T}} ^2\rangle$	[313]
$\mathrm{HF} \rightarrow \mu ^{\pm }$			$1.4<|y |<2$	$R_{\mathrm {dAu}}$ ($\mathrm {N_{coll}}$,$p_{\mathrm {T}}$)	[314]
${b\overline{b}}$			$|y |<0.5$	$\sigma (y)$	[315]
$e^\pm ,\mu ^\pm$			$|y |<0.5$ & $1.4<y <2.1$	$\Delta \phi$, $J_\mathrm{dAu}$	[114]
$\mathrm {J}/\psi$			$-2.2<y <2.4$	$R_{\mathrm {dAu}}$, $R_{\mathrm {CP}}$ ($\mathrm {N_{coll}}$,$y$,$x_2$,$x_{\mathrm {F}}$,$p_{\mathrm {T}}$), $\alpha$	[316–318]
			$-2.2<y <2.2$	$R_{\mathrm {dAu}}$ ($p_{\mathrm {T}}$,$y$,$\mathrm {N_{coll}}$), $\langle p_{\mathrm {T}} ^2\rangle$	[319]
$\mathrm {J}/\psi$, $\psi \text {(2S)}$, $\chi _c$			$|y |<0.35$	$R_{\mathrm {dAu}}$ ($\mathrm {N_{coll}}$), double ratio	[320]
$\Upsilon$			$1.2<|y |<2.2$	$R_{\mathrm {dAu}}$ ($y$,$x_2$,$x_{\mathrm {F}}$), $\alpha$	[321]
STAR					
$\mathrm {D}^{0}$, $\mathrm{HF} \rightarrow e^{\pm }$	d–Au	0.2	$|y |<1$	Yield($y$,$p_{\mathrm {T}}$)	[322]
$\Upsilon$			$|y |<1$	$\sigma$, $R_{\mathrm {dAu}}$ ($y$,$x_{\mathrm {F}}$), $\alpha$	[323]
ALICE					
*D*	p–Pb	5.02	$-0.96<y <0.04$	$\sigma$, $R_{\mathrm {pPb}}$ ($p_{\mathrm {T}}$,$y$)	[324]
$\mathrm {J}/\psi$			$-4.96<y <-2.96$ & $2.03<y <3.53$	$\sigma$, $R_{\mathrm {pPb}}$ ($y$), $R_{\mathrm {FB}}$	[325]
$\mathrm {J}/\psi$, $\psi \text {(2S)}$				$\sigma$, $R_{\mathrm {pPb}}$ ($y$,$p_{\mathrm {T}}$), double ratio	[326]
$\mathrm {J}/\psi$			& $-1.37<y <0.43$	$\sigma$($y$,$p_{\mathrm {T}}$), $R_{\mathrm {pPb}}$ ($y$,$p_{\mathrm {T}}$), [$R_{\mathrm {pPb}}$ (+$y$)$\cdot$ $R_{\mathrm {pPb}}$ (-$y$)] ($p_{\mathrm {T}}$)	[327]
$\Upsilon \text {(1S)}$, $\Upsilon \text {(2S)}$				$\sigma$, $R_{\mathrm {pPb}}$ ($y$), $R_{\mathrm {FB}}$, ratio	[328]
ATLAS					
$\mathrm {J}/\psi$ (from B)	p–Pb	5.02	$-2.87< y <1.94$	$\sigma (y,p_{\mathrm {T}})$, ratio(*y*,$p_{\mathrm {T}}$), $R_{\mathrm {FB}}$ (|*y*|,$p_{\mathrm {T}}$)	[329]
CMS					
$\Upsilon \text {(nS)}$	p–Pb	5.02	$|y |<1.93$	Double ratio ($E_\mathrm{T}^{\eta >4},N_\mathrm{tracks}^{|\eta |<2.4})$	[268]
LHCb					
$\mathrm {J}/\psi$ (from B)	p–Pb	5.02	$-5.0<y <-2.5$ & $1.5<y<4.0$	$\sigma (p_{\mathrm {T}},y)$, $R_{\mathrm {pPb}}$ ($y$), $R_{\mathrm {FB}}$ ($y$,$p_{\mathrm {T}}$)	[330]
$\Upsilon \text {(nS)}$				$\sigma (y)$, ratio($y$), $R_{\mathrm {pPb}}$ ($y$), $R_{\mathrm {FB}}$	[331]

**Table 7:** Available p–A data in fixed target: the probes, the target, $$\sqrt{s_{\mathrm{NN}}} $$, the kinematic range (with $$y $$ the rapidity in the centre-of-mass frame), the observables (as a function of variables) are given as well as the references. The superscript letter a ($$^\mathrm{a}$$) means that a cut on $$|\cos \theta _\mathrm{CS}|<0.5$$ is applied in the analysis, where $$\theta _\mathrm{CS}$$ is the decay muon angle in the Collins–Soper frame. Feynman-*x* variable $$x_{\mathrm {F}} =\frac{2p_\mathrm{L,CM}}{\sqrt{s_\mathrm{NN}}}$$, where $$p_\mathrm{L,CM}$$ is the longitudinal momentum of the partonic system in the CM frame, is connected to the momentum fraction variables by $$x_{\mathrm {F}} \approx x_1-x_2$$, in the limit $$p_\mathrm{T} \ll p$$

Probes	Target	$\sqrt{s_{\mathrm{NN}}}$ (GeV)	$y$ (or $x_{\mathrm {F}}$)	Observables (variables)	References
NA3					
$\mathrm {J}/\psi$	H$_2$, Pt	16.8–27.4	$0<x_{\mathrm {F}} <0.9$	$\sigma (x_{\mathrm {F}},p_{\mathrm {T}})$	[332, 333]
NA38					
$\mathrm {J}/\psi$, $\psi \text {(2S)}$	Cu, U	19.4	$-0.2<y<1.1$	$\sigma (E_\mathrm{T},A)$, $\langle p_{\mathrm {T}} ^{(2)}\rangle (\epsilon )$, ratio($\epsilon ,E_\mathrm{T},A,L)$	[334–336]
$\mathrm {J}/\psi$, $\psi \text {(2S)}$, ${c\overline{c}}$	W	19.4	$0<y<1$	Ratio($\epsilon$), $\sigma _{{c\overline{c}}}(p_\mathrm{lab})$	[337]
$\mathrm {J}/\psi$, $\psi \text {(2S)}$	C, Al, Cu, W	29.1	$-0.4<y <0.6^\mathrm{a}$	$\sigma (A)$ and ratio(*A*)	[338]
$\mathrm {J}/\psi$, $\psi \text {(2S)}$, DY	O, S	19.4 (29.1)	$0(-0.4)<y <1(0.6)$	$\sigma (A,L)$, ratio(*A*, *L*)	[339]
NA38/NA50					
${c\overline{c}}$	Al, Cu, Ag, W	29.1	$-0.52<y <0.48^\mathrm{a}$	$\sigma _{{c\overline{c}}}$	[97]
NA50					
$\mathrm {J}/\psi$, $\psi \text {(2S)}$, DY	Be, Al, Cu, Ag, W	29.1	$-0.4<y <0.6$	$\sigma (A)$, ratio$(A,E_\mathrm{T},L)$, $\sigma _\mathrm {abs}$	[340, 341]
$\mathrm {J}/\psi$, $\psi \text {(2S)}$			$-0.1<x_{\mathrm {F}} <0.1^\mathrm{a}$	$\sigma (A,L)$, $\sigma _\mathrm {abs}$ ($x_{\mathrm {F}}$)	[342]
$\Upsilon$, DY			$-0.5<y <0.5^\mathrm{a}$	$\sigma (A)$, $\langle p_{\mathrm {T}} ^2\rangle (L)$, $\langle p_{\mathrm {T}} \rangle$	[343]
NA60					
$\mathrm {J}/\psi$	Be, Al, Cu, In, W, Pb, U	17.3 (27.5)	$0.3(-0.2)<y<0.8(0.3)^\mathrm{a}$	$\sigma$, $\sigma _\mathrm {abs}$, ratio(*L*), $\alpha (x_{\mathrm {F}},x_2)$	[344]
E772					
$\mathrm {J}/\psi$,$\psi \text {(2S)}$	H$_2$, C, Ca, W	38.8	$0.1<x_{\mathrm {F}} <0.7$	Ratio(*A*,$x_{\mathrm {F}}$,$p_{\mathrm {T}}$), $\alpha (x_{\mathrm {F}},x_2,p_{\mathrm {T}})$	[345]
$\Upsilon$			$-0.15<x_{\mathrm {F}} <0.5$	$\sigma (p_{\mathrm {T}},x_{\mathrm {F}})$, ratio(*A*), $\alpha (x_{\mathrm {F}},x_2,p_{\mathrm {T}})$	[346]
E789					
$\mathrm {D}^{0}$	Be, Au	38.8	$0<x_{\mathrm {F}} <0.08$	$\sigma (p_{\mathrm {T}})$, $\alpha (x_{\mathrm {F}},p_{\mathrm {T}})$, ratio	[347]
${b\overline{b}}$			$0<x_{\mathrm {F}} <0.1$	$\sigma (x_{\mathrm {F}},p_{\mathrm {T}})$	[348]
$\mathrm {J}/\psi$	Be, Cu	38.8	$0.3<x_{\mathrm {F}} <0.95$	$\sigma (x_{\mathrm {F}})$, $\alpha (x_{\mathrm {F}})$	[349]
$\mathrm {J}/\psi$,$\psi \text {(2S)}$	Be, Au		$-0.03<x_{\mathrm {F}} <0.15$	$\sigma (p_{\mathrm {T}},x_{\mathrm {F}},y)$, ratio($p_{\mathrm {T}}$,$x_{\mathrm {F}}$)	[350]
$\mathrm {J}/\psi$	Be, C, W		$-0.1<x_{\mathrm {F}} <0.1$	$\alpha$ ($x_{\mathrm {F}}$,$x_\mathrm{target}$,$p_{\mathrm {T}}$)	[351]
E866/NuSea					
$\mathrm {J}/\psi$, $\psi \text {(2S)}$	Be, Fe, W	38.8	$-0.1<x_{\mathrm {F}} <0.93$	$\alpha$ ($p_{\mathrm {T}}$,$x_{\mathrm {F}}$)	[352]
$\mathrm {J}/\psi$	Cu		$0.3<x_{\mathrm {F}} <0.9$	$\lambda _\theta (p_{\mathrm {T}},x_{\mathrm {F}})$	[252]
$\Upsilon \text {(nS)}$, DY			$0<x_{\mathrm {F}} <0.6$	$\lambda _\theta (p_{\mathrm {T}},x_{\mathrm {F}})$	[353]
HERA-B					
D	C, Ti, W	41.6	$-0.15<x_{\mathrm {F}} <0.05$	$\sigma (x_{\mathrm {F}},p_{\mathrm {T}} ^2)$	[354]
${b\overline{b}}$, $\mathrm {J}/\psi$			$-0.35<x_{\mathrm {F}} <0.15$	$\sigma$, ratio	[355]
${b\overline{b}}$			$-0.3<x_{\mathrm {F}} <0.15$	$\sigma$	[356]
${b\overline{b}}$	C, Ti		$-0.25<x_{\mathrm {F}} <0.15$	$\sigma$	[357]
$\mathrm {J}/\psi$	C, Ti, W		$-0.225<x_{\mathrm {F}} <0.075$	$\sigma (A,y)$	[358]
			$-0.34<x_{\mathrm {F}} <0.14$	$\langle p_{\mathrm {T}} ^2\rangle (A)$, $\alpha (p_{\mathrm {T}},x_{\mathrm {F}})$	[359]
	C, W		$-0.34<x_{\mathrm {F}} <0.14$	$\lambda _\theta ,\lambda _\phi ,\lambda _{\theta \phi }(p_{\mathrm {T}},x_{\mathrm {F}})$	[253]
$\mathrm {J}/\psi$, $\psi \text {(2S)}$	C, Ti, W		$-0.35<x_{\mathrm {F}} <0.1$	Ratio$(x_{\mathrm {F}},p_{\mathrm {T}},A)$, $\alpha ^\prime$-$\alpha$($x_{\mathrm {F}}$)	[254]
$\mathrm {J}/\psi$, $\chi _c$				Ratio($x_{\mathrm {F}}$,$p_{\mathrm {T}}$)	[360]
$\Upsilon$	C, Ti, W		$-0.6<x_{\mathrm {F}} <0.15$	$\sigma (y)$	[361]

**Fig. 25:**
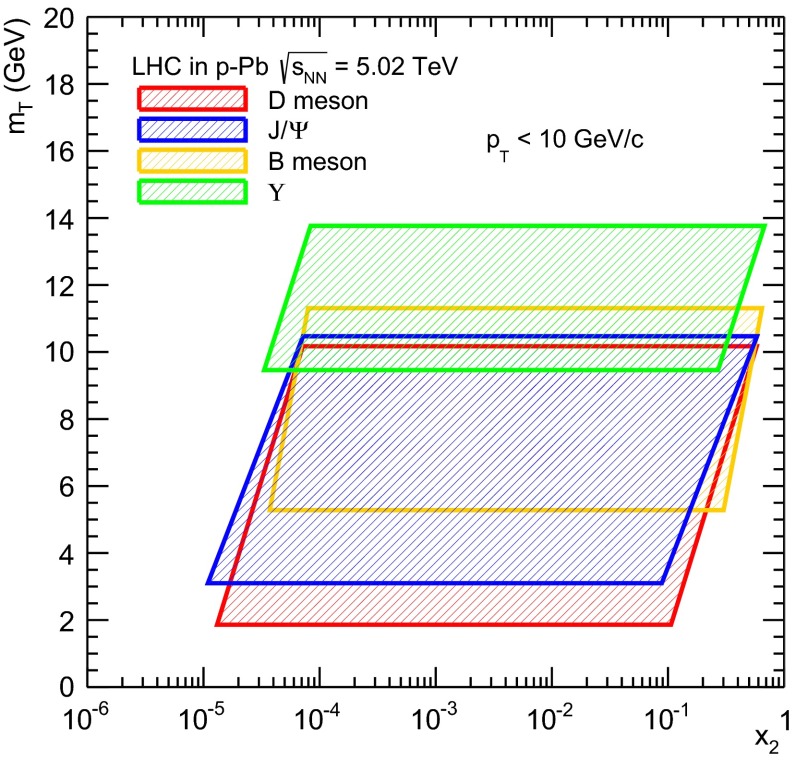
Accessible $x_2$ and $m_\mathrm{T}$ range at the LHC ($|y_{\mathrm {lab}} | < 4.5$) in p–Pb collisions at $\sqrt{s_{\mathrm{NN}}} =5.02$ TeV for different heavy-flavour probes (D and B mesons, $\mathrm {J}/\psi$ and $\Upsilon$) with $0<p_{\mathrm {T}} < 10$ GeV/*c*

In LHC Run 1 p–Pb collisions, protons have an energy of 4 TeV and the Pb nuclei an energy $Z/A(4 \mathrm{TeV})=1.58$ TeV ($Z=82$, $A=208$), leading to $\sqrt{s_{\mathrm{NN}}} =5.02$ TeV and a relative velocity of the CM with respect to the laboratory frame $\beta =0.435$ in the direction of the proton beam. The rapidity of any particle in the CM frame is thus shifted, $y=y_{\mathrm {lab}}-0.465$. Applying those experimental conditions to heavy-flavour probes such as D and B mesons and quarkonia, and according to Eqs. (18) and (19), leads to a large coverage of $x_2$ from $10^{-5}$ for the D meson at forward rapidity, to 0.5 for 10 GeV/*c*$\Upsilon$ at backward rapidity, as reported in Fig. 25.

### Theoretical models for CNM effects

We discuss in this section various theoretical approaches to treat CNM effects, with emphasis on heavy-quark and quarkonium production at the LHC.

#### Typical time scales

Before discussing the various theoretical approaches on cold nuclear matter effects, it is useful to recall the typical time scales entering the process of heavy-quark hadron and quarkonium production in p–A collisions:

- The typical time to produce a heavy-quark pair ${Q\overline{Q}}$, sometimes referred to as the coherence time, which is of the order of $\tau _c \sim 1/m_{{Q\overline{Q}}} \lesssim 0.1$ fm/*c* in the ${Q\overline{Q}}$ rest frame. In the rest frame of the target nucleus, however, this coherence time, $t_{c}=E_{{Q\overline{Q}}}/m^2_{{Q\overline{Q}}}$ (where $E_{{Q\overline{Q}}}$ is the ${Q\overline{Q}}$ energy in the nucleus rest frame), can be larger than the nuclear size, leading to shadowing effects due to the destructive interferences from the scattering on different nucleons.
- The time needed to produce the quarkonium state, also known as the formation time, is much larger than the coherence time. It corresponds to the time interval taken by the ${Q\overline{Q}}$ pair to develop the quarkonium wave function. Using the uncertainty principle, it should be related to the mass splitting between the 1S and 2S states [362], i.e. $\tau _\mathrm{f}\sim (m_{2S}-m_{1S} )^{-1}\sim$0.3–0.4 fm/*c*. Because of the Lorentz boost, this formation time in the nucleus rest frame, $t_\mathrm{f}$, becomes much larger than the nuclear size at the LHC. Consequently the quarkonium state is produced far outside the nucleus and should not be sensitive to nuclear absorption. The time to produce a heavy-quark hadron is longer than for quarkonium production, of the order of ${\Lambda _\mathrm{QCD}}^{-1} \simeq 1$ fm /*c* in its rest frame.
- Another important time scale is the typical time needed for the ${Q\overline{Q}}$ pair to neutralise its colour. In the colour singlet model, this process occurs through the emission of a perturbative gluon and should thus occur in a time comparable to $\tau _{c}$. In the colour octet model (or colour evaporation model), colour neutralisation happens through a soft process, i.e. on “long” time scales, typically of the order the quarkonium formation time $\tau _\mathrm{f}$.

When discussing the possible nuclear absorption of a quarkonium state in the nucleus, it is common to compare the crossing time of the nucleus, $\tau _\mathrm{cross}$, which is the time spent by the state in the nucleus [320] to its formation time $\tau _f$. It is given by $\tau _\mathrm{cross} = L / (\beta _z\ \gamma )$, where *L* is the longitudinal path of the ${Q\overline{Q}}$ pair through the nucleus, $\beta _z$ and $\gamma = \sqrt{1-\beta _z^2}$ are the velocity and Lorentz factor of the quarkonium along the beam direction, both given in the nuclear rest frame.

#### Nuclear PDFs

The modification of parton densities in nuclei affects the yields of heavy-quark and quarkonium production. In this section, the effects of nPDF on $\mathrm {J}/\psi$ and $\Upsilon$ production in p–Pb collisions at the LHC are first presented. The production of open beauty (through its decay into non-prompt $\mathrm {J}/\psi$) is then discussed.

$\mathrm {J}/\psi$*and *$\Upsilon$*production* The predictions for $\mathrm {J}/\psi$ suppression due to the nuclear modifications of the parton densities are described in this section and discussed by Vogt in [363]. Here we show results for the rapidity dependence of nPDF effects on $\mathrm {J}/\psi$ and $\Upsilon$ production in p–Pb collisions at $\sqrt{s_{\mathrm{NN}}} = 5.02$ TeV and neglecting any other CNM effect.

The results are obtained in the colour evaporation model (CEM) at next-to-leading order in the total cross section. In the CEM, the quarkonium $\Phi$ production cross section in p–Pb collisions is some fraction, $F_\Phi$, of all $Q \overline{Q}$ pairs below the $H \overline{H}$ threshold where *H* is the lowest-mass heavy-flavour hadron,

20 $$\begin{aligned}&\sigma _{\mathrm{pPb}\rightarrow \Phi +X}^\mathrm{CEM}[\sqrt{s} ] \nonumber \\&\quad =A \cdot F_\Phi \sum _{i,j} \int _{4m_\mathrm{Q}^2}^{4m_H^2} \mathrm {d}\hat{s} \int _0^1 \mathrm {d}x_i \int _0^1 \mathrm {d}x_j~ f_i(x_i,\mu _F^2)\nonumber \\&\qquad \times R_j^\mathrm{Pb}(x_j,\mu _F^2)f_j(x_j,\mu _F^2)~ \mathcal{J}~{\hat{\sigma }}_{ij\rightarrow {Q\overline{Q}} +X}[\hat{s},\mu _F^2, \mu _R^2],\nonumber \\ \end{aligned}$$

where *A* is the Pb mass number, $ij = {q\overline{q}}$ or *gg*, and ${\hat{\sigma }}_{ij\rightarrow {Q\overline{Q}} +X}$ is the $ij\rightarrow {Q\overline{Q}} +X$ sub-process cross section of centre-of-mass energy $\hat{s}$. $\mathcal{J}$ is an appropriate Jacobian with dimension $1/\hat{s}$. $f_{i,j}$ is the proton PDF for the parton species *i*, while $R_j^\mathrm{Pb}$ is a nuclear PDF parametrisation for the parton species *j* (EPS09 [364] for the results shown in this section). The normalisation factor $F_\Phi$ is fitted to the forward (integrated over $x_{\mathrm {F}} > 0$) $\mathrm {J}/\psi$ cross section data on p, Be, Li, C, and Si targets (see [169] for details). In this way, uncertainties due to ignoring any cold nuclear matter effects which are on the order of a few percent in light targets are avoided. The fits are restricted to the forward cross sections only.

The values of the central charm quark mass and scale parameters are $m_c = 1.27 \pm 0.09$ GeV/$c^2$, $\mu _F/m_c = 2.10 ^{+2.55}_{-0.85}$, and $\mu _R/m_c = 1.60 ^{+0.11}_{-0.12}$ [169]. The normalisation $F_\Phi$ is obtained for the central set, $(m_c,\mu _F/m_c, \mu _R/m_c) = (1.27 \, \mathrm{GeV}/c^2, 2.1,1.6)$. The calculations for the estimation of the mass and scale uncertainties are multiplied by the same value of $F_\Phi$ to obtain the $\mathrm {J}/\psi$ uncertainty band [169]. $\Upsilon$ production is calculated in the same manner, with the central result obtained for $(m_b,\mu _F/m_b, \mu _R/m_b) = (4.65 \pm 0.09 \, \mathrm{GeV}/c^2, 1.4^{+0.77}_{-0.49},1.1^{+0.22}_{-0.20})$ [365]. In the NLO calculations of the rapidity and $p_{\mathrm {T}}$ dependence, instead of $m_Q$, the transverse mass, $m_{\mathrm {T}}$, is used with $m_{\mathrm {T}} = \sqrt{m_Q^2 + p_{\mathrm {T}} ^2}$ where $p_{\mathrm {T}} ^2 = 0.5\,(p_{\mathrm{T}_Q}^2 + p_{\mathrm{T}_{\overline{Q}}}^2)$. All the calculations are NLO in the total cross section and assume that the intrinsic $k_{\mathrm {T}}$ broadening is the same in $\mathrm pp$ as in p–Pb.

The mass and scale uncertainties are calculated based on results using the one standard deviation uncertainties on the quark mass and scale parameters. If the central, higher and lower limits of $\mu _{R,F}/m$ are denoted *C*, *H*, and *L*, respectively, then the seven sets corresponding to the scale uncertainty are $\{(\mu _F/m,\mu _R/m)\}$ = $\{(C,C), (H,H), (L,L), (C,L), (L,C), (C,H), (H,C)\}$. The uncertainty band can be obtained for the best fit sets by adding the uncertainties from the mass and scale variations in quadrature. The uncertainty band associated to the EPS09 NLO set is obtained by calculating the deviations from the central EPS09 set for the 15 parameter variations on either side of the central set and adding them in quadrature. The uncertainty on $R_\mathrm{pA}$ associated to the EPS09 NLO variations turns out to be larger than that coming from the mass and scale variation, as can be seen below.

**Fig. 26:**
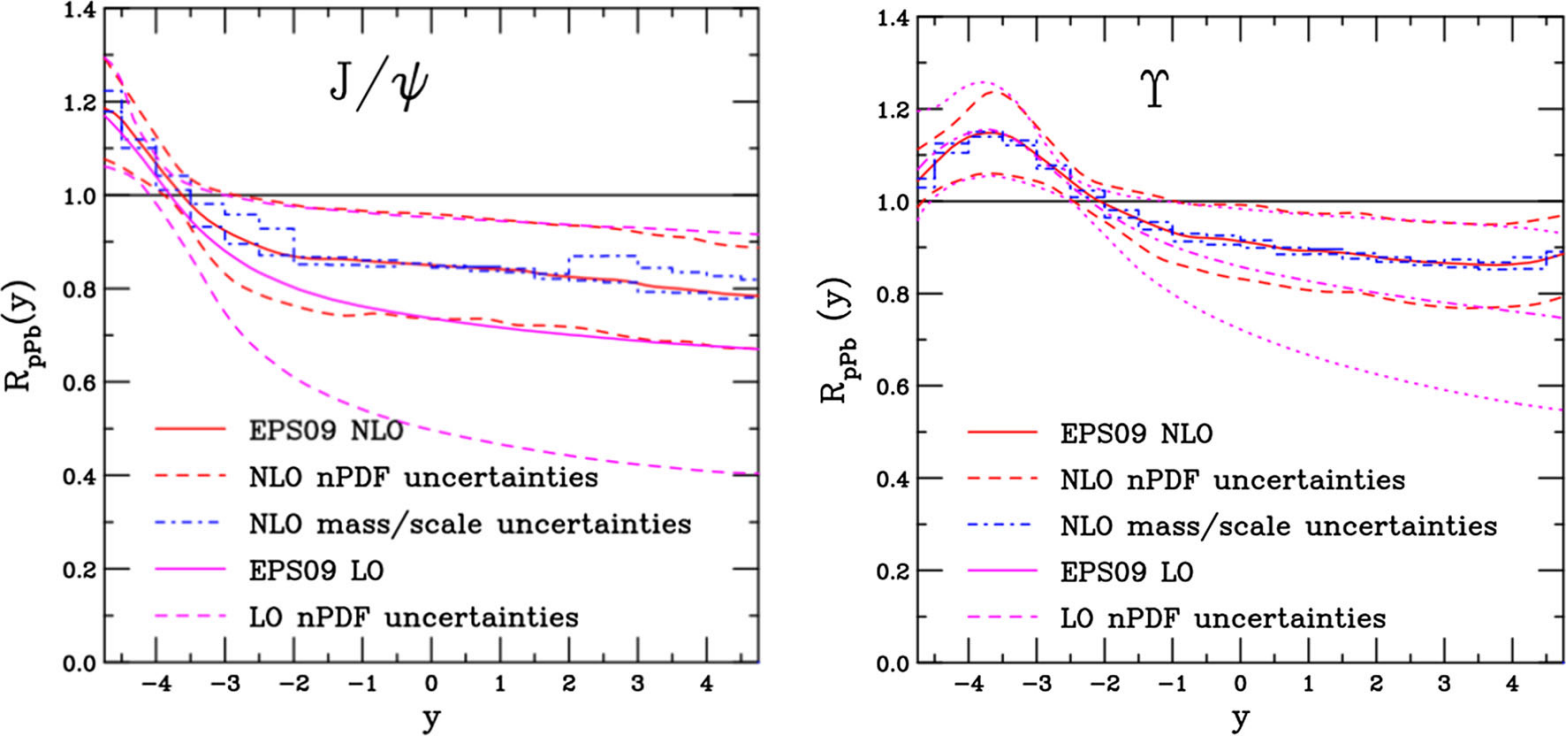
The nuclear modification factor $R_{\mathrm {pPb}}$ for $\mathrm {J}/\psi$ (*left*) and $\Upsilon$ (*right*) production calculated using the EPS09 modifications as a function of rapidity. The *solid red histogram* shows the central EPS09 NLO prediction (with its uncertainties shown as *red dashed histograms*) in p–Pb collisions at $\sqrt{s_{\mathrm{NN}}} =5.02$ TeV (integrated over $p_{\mathrm {T}}$) while the *dot-dashed blue histogram* shows the dependence on mass and scale. The *magenta curves* show the LO modification and the corresponding uncertainty band. The NLO $\mathrm {J}/\psi$ results were originally shown in Ref. [366]

Figure 26 (left) shows the uncertainty in the shadowing effect on $\mathrm {J}/\psi$ due to the variations in the 30 EPS09 NLO sets [364] (dashed red) as well as those due to the mass and scale uncertainties (dashed blue) calculated with the EPS09 NLO central set. The uncertainty band calculated in the CEM at LO with the EPS09 LO sets [364] is shown for comparison. It is clear that the LO results, represented by the smooth magenta curves in Fig. 26, exhibit a larger shadowing effect. This difference between the LO results, also shown in Ref. [363], and the NLO calculations arises because the gluon distributions in the proton that the EPS09 LO and NLO gluon shadowing parametrisations are based on CTEQ61L and CTEQ6M, respectively, which behave very differently at low *x* and moderate values of the factorisation scale [364]. If one uses instead the nDS or nDSg parametrisations [367], based on the GRV98 LO and NLO proton PDFs, the LO and NLO results differ by only a few percent. The right panel shows the same calculation for $\Upsilon$ production. Here the difference between the LO and NLO calculations is reduced because the mass scale, and hence the factorisation scale, is larger. The *x* values probed are also correspondingly larger.

The $p_{\mathrm {T}}$ dependence of the nPDF effects at forward rapidity for $\mathrm {J}/\psi$ and $\Upsilon$ has also been computed in Ref. [366]. There is no LO comparison because the $p_{\mathrm {T}}$ dependence cannot be calculated in the LO CEM. The effect is rather mild and $R_{\mathrm {pPb}}$ increases slowly with $p_{\mathrm {T}}$, from roughly $R_{\mathrm {pPb}} \simeq 0.7$–0.9 for $\mathrm {J}/\psi$ at low $p_{\mathrm {T}}$ to $R_{\mathrm {pPb}} \simeq 1$ at $p_{\mathrm {T}} = 20$ GeV/c. There is little difference between the $\mathrm {J}/\psi$ and $\Upsilon$ results for $R_{\mathrm {pPb}} (p_{\mathrm {T}})$ because, for $p_{\mathrm {T}}$ above a few GeV / *c*, the $p_{\mathrm {T}}$ scale dominates over the mass scale. The nPDF effects are somewhat similar for open heavy flavour as a function of $p_{\mathrm {T}}$, yet the effects (estimated using EPS09 NLO) tend to go away faster with $p_{\mathrm {T}}$ due to the different production dynamics between quarkonium and open heavy flavour.

*Non-prompt*$\mathrm {J}/\psi$*production* The nPDF effects on non-prompt $\mathrm {J}/\psi$ (coming from B decays) has been investigated by Ferreiro et al. in [368]. Contrary to the more complex case of bottomonium production, it is sufficient to rely on LO calculations [369] to deal with open-beauty production data integrated in $p_{\mathrm {T}}$ as those of LHCb [330]. Indeed such computations are sufficient to describe the low-$p_{\mathrm {T}}$ cross section up to (1–2) $m_b$, where the bulk of the yield lies.

The nPDF effects on non-prompt $\mathrm {J}/\psi$ have been evaluated using two parametrisations,^16^ namely EPS09 LO [364]^17^ and nDSg LO [367]. In addition to the choice of the nPDFs, one also has to fix the value of the factorisation scale $\mu _F$, which is set to $\mu _F=\sqrt{m_Q^2+p_{\mathrm {T}} ^2}$. One can also consider the spatial dependence of the nPDFs, either by simply assuming an inhomogeneous shadowing proportional to the local density [370, 371] or extracting it from a fit [372]. These effects would then translate into a non-trivial centrality (or impact parameter *b*) dependence of the nuclear modification factor. To this end, it is ideal to rely on a Glauber Monte-Carlo which does not factorise the different nuclear effects (such as JIN [373] which is used to study the nuclear matter effects on quarkonium production both at RHIC [374, 375] and LHC [376, 377] energies).

**Fig. 27:**
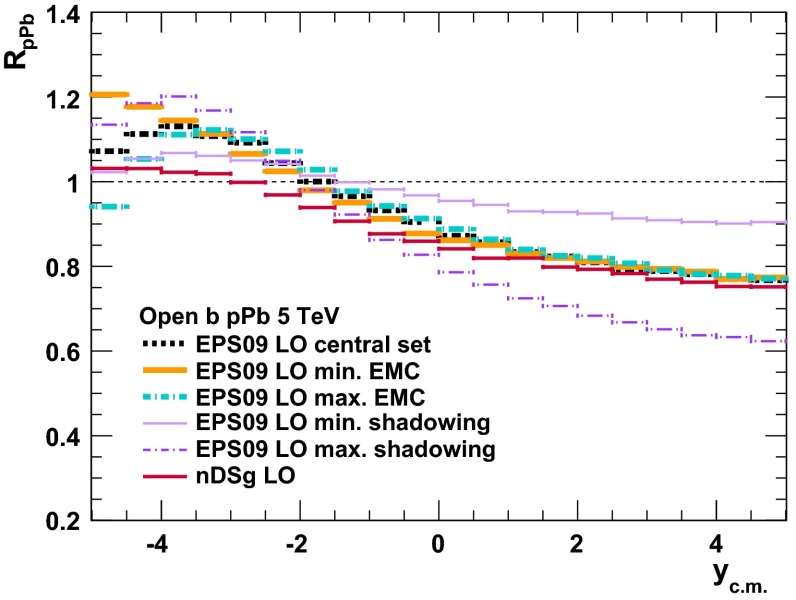
Effect of nPDF as encoded in EPS09 LO on ${R_{\mathrm {pPb}}}^{b\rightarrow \mathrm {J}/\psi }$ at $\sqrt{s_{\mathrm{NN}}} =5$ TeV

This results in the nuclear modification factor $R_{\mathrm {pPb}}$ for open beauty in p–Pb collisions at 5 TeV shown in Fig. 27. These values will be compared to the data measured by the LHCb Collaboration [330] and shown in Fig. 37, at backward and forward rapidity. As discussed in [368], the measured values of $R_{\mathrm {pPb}}$ slightly favour the nDSg parametrisation, which does not include anti-shadowing. One should, however, stress that such a direct theory-data comparison relies on a good control of the interpolated $\mathrm pp$ cross section, while the forward-over-backward production ratio is not affected by any kind of uncertainty on the $\mathrm pp$ measurements or modelling. In this case, there is no tension with EPS09. Finally, one can stress that the nuclear modification factor predicted for open beauty is similar to that of inclusive $\Upsilon$(1S).

#### Saturation in the colour glass condensate approach

Fujii and Watanabe recently computed the heavy quark production cross section in high energy p–A collisions in the colour Glass Condensate (CGC) framework [378, 379], which is given at the leading order in the strong-coupling constant $\alpha _s$, but includes multiple-scattering effects on the gluons and heavy quarks by the dense target [380]. It is expressed in terms of hard matrix elements, 2-point gluon function in the dilute projectile and multi-point gluon functions in the dense target, which breaks the $k_{\mathrm {T}}$-factorisation [381]. The energy dependence in this approach is incorporated through the gluon functions which obey the non-linear *x*-evolution equation leading to the gluon saturation phenomenon.

**Fig. 28:**
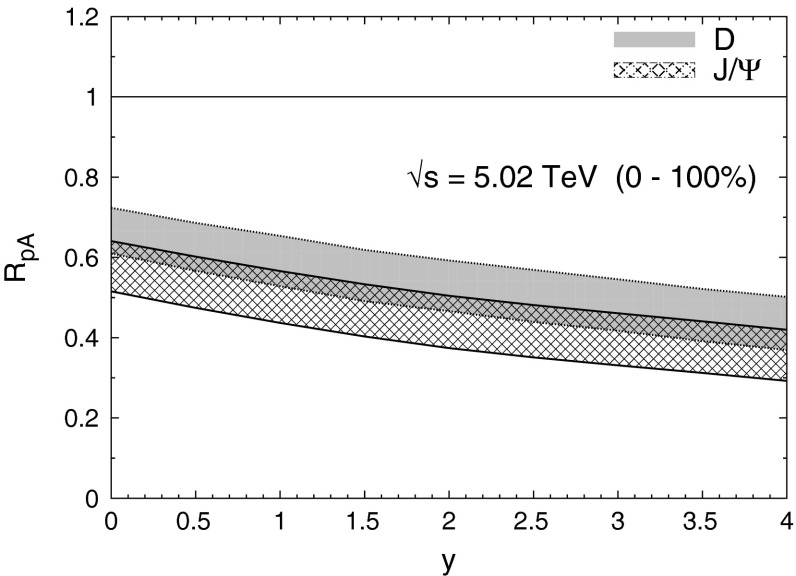
Nuclear modification factor $R_{\mathrm {pA}} (y)$ for D and $\mathrm {J}/\psi$ in p–Pb collisions at $\sqrt{s_{\mathrm{NN}}} =5.02$ TeV in the CGC approach of Fuji and Watanabe [378, 379]. The *bands* indicate the uncertainties from the variations $m_c=1.2$–1.5 GeV$/c^2$ and $Q_{s,A}^2(x_0)= (4-6) Q_{s,p}^2(x_0)$

In the large-$N_c$ approximation (where $N_c=3$ is the number of colours in QCD), the multi-point functions reduce to a product of two dipole amplitudes in the fundamental representation and the evolution equation for the dipole has a closed form, called the Balitsky–Kovchegov (BK) equation. The BK equation with running coupling corrections (rcBK) is today widely exploited for phenomenological studies of saturation, and its numerical solution for $x<x_0=0.01$ is constrained with HERA DIS data and has been applied to hadronic reactions successfully [382]. Nuclear dependence is taken into account here in the initial condition for the rcBK equation by setting larger initial saturation scales, $Q_{s,A}^2(x_0)$ (below which gluon distribution in a nucleus starts to saturate) depending on the nuclear thickness.

References [378, 379] show the evaluation of heavy quark production applying the CGC framework in the large-$N_c$ approximation with the numerical solution of the rcBK equation. In hadronisation processes, the colour evaporation model (CEM) is used for $\mathrm {J}/\psi$ ($\Upsilon$) and the vacuum fragmentation function for D meson production, assuming that the hadronisation occurs outside the target as mentioned in Sect. 3.2.1. The rapidity dependence of the nuclear modification factor $R_{\mathrm {pA}} (y)$ of $\mathrm {J}/\psi$ is one of the significant observables to investigate the saturation effect and the CGC-based model reproduced the RHIC data by setting $Q_{s,A}^2(x_0)=(4 - 6) Q_{s,p}^2(x_0)$. Extrapolation to the LHC energy predicted a stronger suppression, reflecting stronger saturation effects at the smaller values of *x* (Fig. 28). Quarkonium suppression in this framework also includes the multiple scattering effects on the quark pair traversing the dense target. The comparison with experimental results will shown in Sect. 3.3.

Several improvements to this approach can be performed. The CGC expression for the heavy quark production is derived at LO in the eikonal approximation for the colour sources. The NLO extension should be investigated to be consistent with the use of the rcBK equation. Furthermore, for quarkonium production, colour channel dependence of the hadronisation process will be important and brings in a new multi-point function, which is simply ignored in CEM. Finally, using a similar approach but with an improved treatment of the nuclear geometry and a different parametrisation of the dipole cross section, Ducloué et al. [383] showed that the $\mathrm {J}/\psi$ suppression in p–Pb collisions was less pronounced.

More recently, attempts to compute quarkonium production in $\mathrm pp$ and p–A collisions have been made by implementing small-*x* evolution and multiple scattering effects in the NRQCD formalism [93]. Depending on which NRQCD channel dominates the $\mathrm {J}/\psi$ production cross section in p–Pb collisions at the LHC, the $\mathrm {J}/\psi$ suppression predicted in this formalism may agree with the current ALICE and LHCb measurements [384].

#### Multiple scattering and energy loss

In this section various approaches of parton multiple scattering in nuclei are discussed. These effects include ${Q\overline{Q}}$ propagation in nuclei, initial- and final-state energy loss, and coherent energy loss.

${Q\overline{Q}}$  *propagation and attenuation in nuclei* This section summarises the approach by Kopeliovich et al. [385, 386]. At LHC energies, the coherence time, $t_c$, for the production of charm quarks exceeds the typical nuclear size, $t_c\gg R_\mathrm{A}$. As a consequence, all the production amplitudes from different bound nucleons are in phase. In terms of the dipole description this means that Lorentz time delay “freezes” the ${c\overline{c}}$ dipole separation during propagation through the nucleus, which simplifies calculations compared with the path-integral technique, required at lower energies [362, 387, 388].

Because of the rescattering of the dipole in the nucleus, the charmonium suppression in p–A collisions with impact parameter *b* has the form [385, 386, 388],

21 $$\begin{aligned}&R_{\mathrm {pA}} ={1\over A}\int \mathrm {d}^2b\int \limits _{-\infty }^{\infty }\mathrm {d}z\, \rho _\mathrm{A}(b,z) |S_{\mathrm {pA}} (b,z)|^2, \end{aligned}$$

22 $$\begin{aligned}&S_{\mathrm {pA}} (b,z)=\int \mathrm {d}^2r_\mathrm{T}\,W_{{c\overline{c}}}(r_\mathrm{T})\times \exp \Big [-{1\over 2}\sigma _{{c\overline{c}} g}(r_\mathrm{T})T_-(b,z)\nonumber \\&\quad -{1\over 2}\sigma _{{c\overline{c}}}(r_\mathrm{T})T_+(b,z)\Big ]. \end{aligned}$$

**Fig. 29:**
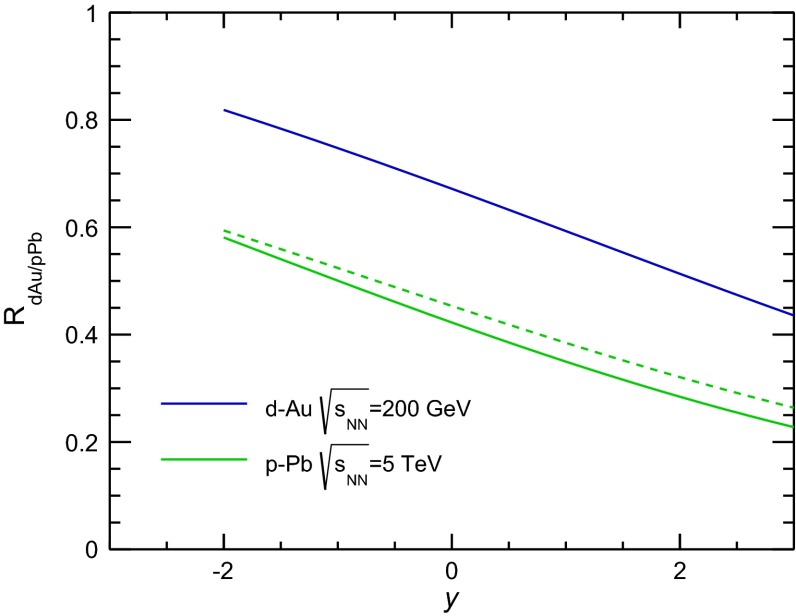
Calculations [385] for the $p_{\mathrm {T}}$-integrated nuclear suppression factor $R_{\mathrm {dAu}} (y)$ for $\mathrm {J}/\psi$ produced in d–Au collisions with rapidity *y* at $\sqrt{s_{\mathrm{NN}}} =200\,$GeV. The *upper solid curve* presents the result of Eqs. (21) and  (22), including a small effect from shadowing. The *lower solid* (*dashed*) *curve* shows calculations for LHC at $\sqrt{s_{\mathrm{NN}}} =5\,$TeV, including (excluding) shadowing

Here $W_{{c\overline{c}}}(r_\mathrm{T})\propto K_0(m_c r_\mathrm{T})\,r_\mathrm{T}^2\,\Psi _{f}(r_\mathrm{T})$ is the distribution function for the dipole size $r_\mathrm{T}$; $K_0(m_c r_\mathrm{T})$ describes the $r_\mathrm{T}$-distribution of the ${c\overline{c}}$ dipole in the projectile gluon; $\Psi _f(r_\mathrm{T})$ is the light-cone wave function of the final charmonium; one factor $r_\mathrm{T}$ comes from the colour-exchange transition $({c\overline{c}})_8\rightarrow ({c\overline{c}})_1$ amplitude, another factor $r_\mathrm{T}$ originates either from radiation of a gluon (colour-singlet model for $\psi$), or from the wave function of a *P*-wave state ($\chi$). The three-body ($g{c\overline{c}}$) dipole cross section $\sigma _{{c\overline{c}} g}(r_\mathrm{T})={9\over 4}\sigma _{{c\overline{c}}}(r_\mathrm{T}/2)-{1\over 8}\sigma _{{c\overline{c}}}(r_\mathrm{T})$ is responsible for the $g\rightarrow {c\overline{c}}$ transition and its nuclear shadowing. The thickness functions are defined by $T_-(b,z)=\int _{-\infty }^z \mathrm {d}z'\rho _\mathrm{A}(b,z')$;  $T_+(b,z)=T_A(b)-T_-(b,z)$ and $T_A(b)=T_-(b,\infty )$ where $\rho _\mathrm{A}$ is the nuclear density profile. The results of parameter-free calculations [386] of $R_{\mathrm {pA}}$ as a function of rapidity at the energies of RHIC and LHC are shown in Fig. 29.

At this point one should emphasise that attenuation of ${c\overline{c}}$ dipoles in nuclear matter is a source of nuclear suppression of $\mathrm {J}/\psi$, although it is often not included in model calculations. Moreover, independently of model details, the general features of dipole interactions are: (i) the dipole cross section studied in detail at HERA, which is proportional to the dipole size squared (of the order of $1/m_c^2$) and to the gluon density, (ii) the rise of the dipole cross section (and therefore the magnitude of the nuclear suppression) coming from the observed steep rise of the gluon density at small *x*. The observed energy independence of nuclear suppression of $\mathrm {J}/\psi$ is incompatible with these features, and the only solution would be the presence of a nuclear enhancement mechanism rising with energy. Indeed, such a mechanism was proposed in [389] and developed in [388]. It comes from new possibilities, compared to a proton target, for $\mathrm {J}/\psi$ production due to multiple colour exchange interaction of a ${c\overline{c}}$ in the nuclear matter, e.g. the relative contribution of double interaction is enhanced in nuclei as $A^{1/3}$ and rises with energy proportionally to the dipole cross section [389]. Numerical evaluation of this effect is under way [390]. This approach for charmonium production cannot be simply extrapolated from p–A to AA collisions [385]. The latter case includes new effects of double colour filtering and a boosted saturation scale [385].

*Initial- and final-state energy loss, power corrections and Cronin effect* The approach by Sharma and Vitev is now described. The basic premiss of this approach is that CNM can be evaluated and related to the transport properties of large nuclei for quarks and gluons [391]. At one extreme, when the scattering from the medium is largely incoherent, the parton modification is dominated by transverse momentum broadening. It leads to a Cronin-like enhancement of the cross sections at intermediate $p_{\mathrm {T}} \sim$ few GeV / *c*. At the other extreme, when the longitudinal momentum transfer is small compared to the inverse of the path length of the parton as it propagates through the nucleus, the scattering becomes coherent, which can lead to attenuation, or shadowing. The coherent limit is described differently in different approaches and its effects are calculated in terms of nuclear-enhanced power corrections to the cross sections. Multiple scattering also leads to medium-induced radiative corrections that, in the soft gluon emission limit, have the interpretation of energy loss [392].

**Fig. 30:**
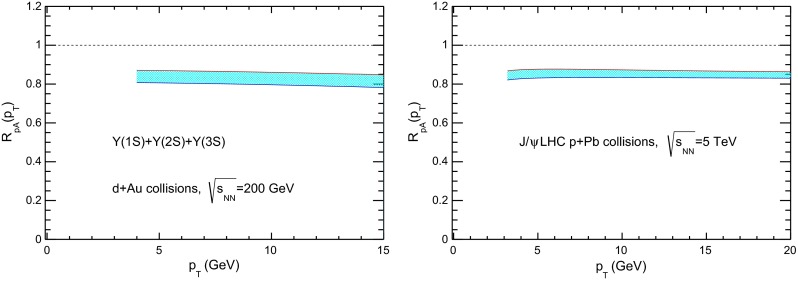
Theoretical calculations [83] for $\Upsilon$ and $\mathrm {J}/\psi$ $R_{\mathrm {pA}}$, as a function of $p_{\mathrm {T}}$ and at mid-rapidity, in minimum-bias collisions with a small (*upper curve, red*) and large (*lower curve, blue*) energy-loss effect at RHIC and LHC, respectively

The effects are implemented via modifications to the kinematics of hard parton scattering $a+b\rightarrow c+d$. For example, in p–A collisions

23 $$\begin{aligned}&\mathrm{Initial{-}state \; energy \; loss }\nonumber \\&[ \phi _a({{x}}_{a}) ]_\mathrm{pA} = \left[ \phi _a\left( \frac{ {{x}}_{a}}{1-\epsilon _{a}}\right) \right] _\mathrm{NN}, \quad \epsilon _{a}=\frac{\Delta E_{a}}{E_{a}}, \end{aligned}$$

24 $$\begin{aligned}&\mathrm{Power \; corrections } \nonumber \\&( {x}_{b})_\mathrm{pA} = (x_b)_\mathrm{NN}\left[ 1+\frac{\xi _d^2(A^{1/3}-1)}{-\hat{t}+m_d^2}\right] , \end{aligned}$$

25 $$\begin{aligned}&\mathrm{Cronin \; effect }\;\nonumber \\&\quad \langle {k}_{\mathrm{T}a}^2 \rangle _\mathrm{pA} = \langle {k}_\mathrm{T}^2 \rangle _\mathrm{NN} + \langle {k}_{\mathrm{T}a}^2 \rangle _\mathrm{IS}\, , \; \nonumber \\&\quad \langle {k}_{\mathrm{T}a}^2 \rangle _\mathrm{IS}= \left\langle \frac{2 \mu ^2 L}{\lambda _{a}} \right\rangle . \end{aligned}$$

In Eq. (23), $\epsilon _{a}$ is the fractional energy loss for parton *a* prior to the hard collision, which increases linearly with medium opacity. When the inverse longitudinal momentum transferred from the nucleus is larger than the Lorentz-contracted longitudinal size, the scattering can become coherent. This effect can be included in an effective modification of the Bjorken-*x* variable, as shown in Eq. (24), in which $\xi _d$ is a parameter monitoring the strength of power corrections. The momentum broadening leading to Cronin effect is given in Eq. (25) in which $\mu$ is the typical transverse momentum transfer in a parton–nucleon scattering and $\lambda _a$ the parton mean free path in the nuclear medium. The typical transverse momentum scales and scattering lengths are $\xi _d^2 / 1\; \mathrm{fm} \sim \mu ^2/\lambda \simeq 0.1$ GeV$^2$/fm (0.225 GeV$^2$/fm) for quarks (gluons), respectively. These yield a quark radiation length $X_0 \sim 50$ fm [393]. For further details, see [83, 391, 393]. This approach has successfully described the experimentally observed suppression of light hadron, photon and dilepton production cross sections.

**Fig. 31:**
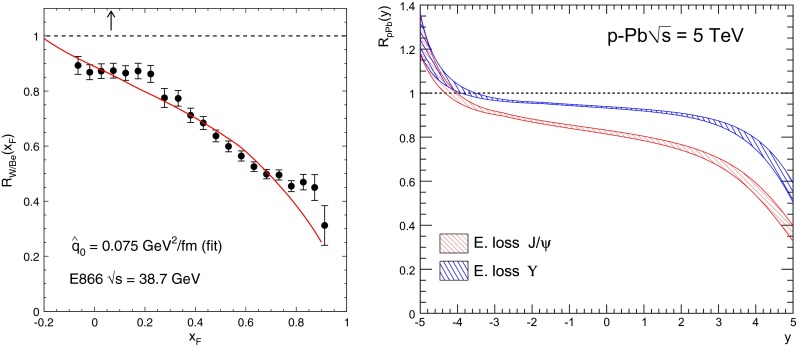
*Left* $\mathrm {J}/\psi$ suppression due to coherent energy-loss effects, fitted to E866 data in p–W collisions at $\sqrt{s_{\mathrm{NN}}} =38.7$ GeV, as a function of Feynman-*x*, $x_\mathrm{F} \simeq 2 p_z^{\mathrm {J}/\psi }/\sqrt{s}$. The *vertical arrow* indicates below which $x_\mathrm{F}$ values $\mathrm {J}/\psi$ production may be sensitive to nuclear absorption. *Right* predictions of $\mathrm {J}/\psi$ and $\Upsilon$ suppression in p–Pb collisions at the LHC. From Refs. [394–398]

As the heavy quark introduces a new mass scale, the dependence of CNM corrections on this scale and their relative significance needs to be reassessed in light of the experimental data.

For the case of quarkonium production, a large uncertainty arises form the fact that the Cronin effect is not understood [83], nor have there been attempts to fit it in this approach. Consequently, for $$\mathrm {J}/\psi $$ and $$\Upsilon $$ results with only CNM energy loss are shown. Due to the uncertainties in the magnitude of the Cronin effect and the magnitude of the cold nuclear matter energy loss, the nuclear modification for open heavy flavour can show either small enhancement and small suppression in the region of $$p_{\mathrm {T}} \sim $$ few GeV / *c*. The uncertainties in the magnitude of $$\Delta E / E$$ can be quite significant [393]. Motivated by other multiple parton scattering effects, such as the Cronin and the coherent power corrections, which are both compatible with possibly smaller transport parameters of cold QCD matter we also consider an energy loss that is $$35~\%$$ smaller than the one from using the parameters above. The results for quarkonium modification in p–A collisions is then presented as a band. The left panel of Fig. 30 shows theoretical predictions for $$\Upsilon $$$$R_{\mathrm {dAu}} $$ at RHIC [83]. The right panel of Fig. 30 shows theoretical predictions for $$\mathrm {J}/\psi $$$$R_{\mathrm {pPb}} $$ at the LHC [83] that will be compared to data in Sect. 3.3.

*Coherent energy loss* Another approach of parton energy loss in cold nuclear matter has been suggested by Arleo et al. in Refs. [394–398]. A few years ago it was emphasised that the medium-induced radiative energy loss $\Delta E$ of a high-energy gluon crossing a nuclear medium and being scattered to small angle is proportional to the gluon energy *E* [394, 397]. The behaviour $\Delta E \propto E$ arises from soft gluon radiation which is *fully coherent* over the medium. Coherent energy loss is expected in all situations where the hard partonic process looks like forward scattering of an incoming parton to an outgoing *compact* and *colourful* system of partons [398]. In the case of $\mathrm {J}/\psi$ hadroproduction at low $p_{\mathrm {T}} \lesssim m_{\mathrm {J}/\psi }$, viewed in the target rest frame as the scattering of an incoming gluon to an outgoing *colour octet*${c\overline{c}}$ pair,^18^ such an energy loss provides a successful description of $\mathrm {J}/\psi$ nuclear suppression in p–A as compared to $\mathrm pp$ collisions, from fixed-target (SPS, HERA, FNAL) to collider (RHIC, LHC) energies [395, 396].

In Refs. [395, 396], the $\mathrm {J}/\psi$ differential cross section $\mathrm {d}^2\sigma _\mathrm{pp}/\mathrm {d}y \, \mathrm {d}p_{\mathrm {T}}$ is determined from a fit of the $\mathrm pp$ data, and $\mathrm {d}^2\sigma _\mathrm{pA}/\mathrm {d}y \, \mathrm {d}p_{\mathrm {T}}$ is obtained by performing a shift in rapidity (and in $p_{\mathrm {T}}$) accounting for the energy loss $\varepsilon$ with probability $\mathcal{P}(\varepsilon )$ (and for the transverse broadening $\Delta p_{\mathrm {T}}$) incurred by the compact octet state propagating through the nucleus. Independent of the $\mathrm pp$ production mechanism, the model is thus able to predict $\mathrm {J}/\psi$ and $\Upsilon$ nuclear *suppression*, $R_{\mathrm {pA}}$, as a function of *y*, $p_{\mathrm {T}}$ and centrality. The model depends on a single parameter $\hat{q}_0$, which fully determines both the broadening $\Delta p_{\mathrm {T}}$ and the energy-loss probability distribution, $\mathcal{P}(\varepsilon )$. It is determined by fitting the model calculations to the E866 measurements [352] in p–W collisions at $\sqrt{s_{\mathrm{NN}}} =38.7$ GeV. The result of the fit, which yields $\hat{q}_0=0.075$ GeV$^2$/fm, is shown in Fig. 31 (left) in comparison to the data.

In order to assess the uncertainties of the model predictions, the parameter entering the $\mathrm pp$ data parametrisation is varied around its central value, as well as the magnitude of the transport coefficient from 0.07 to 0.09 GeV$^2$/fm [395]. The prescription for computing the model uncertainties can be found in [404]. The model predictions for $\mathrm {J}/\psi$ and $\Upsilon$ suppression in p–Pb collisions at the LHC as a function of rapidity are shown in Fig. 31 (right). The extrapolation of the model to AA collisions is discussed in Sect. 3.4.

#### Nuclear absorption

The quarkonium nuclear absorption is characterised by an “effective” cross section $\sigma _\mathrm{abs}$. In Ref. [405], Arleo and Tram analysed all the $\mathrm {J}/\psi$ cross section measurements available at the time, taking into account nuclear absorption and nPDF effects. They found that, within the experimental uncertainties, the absorption cross section does not show a dependence on the $\mathrm {J}/\psi$–N centre-of-mass energy, when going from fixed-target to RHIC energy. In the approach of Ref. [399] discussed below, Lourenço, Vogt and Woehri studied the available fixed-target data to discern a possible dependence of the $\mathrm {J}/\psi$ normal absorption at mid-rapidity as a function of the nucleon–nucleon centre-of-mass energy, both with and without considering nuclear modifications of the parton distributions.

**Fig. 32:**
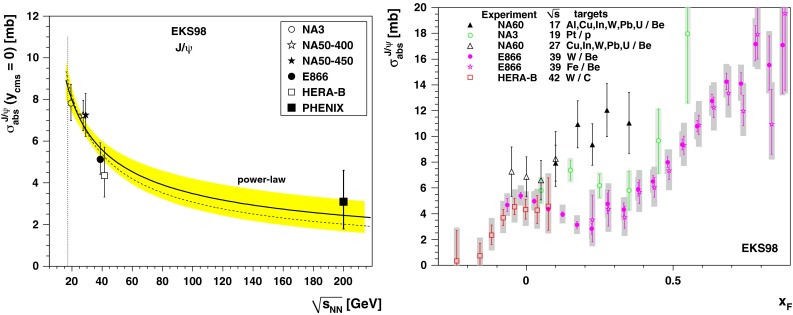
*Left* energy dependence of $\sigma _\mathrm{abs}^{\mathrm {J}/\psi }$ at mid-rapidity [399] using the EKS98-CTEQ61L nPDFs [400–403]. *Right* the $x_{\mathrm {F}}$ dependence of $\sigma _\mathrm{abs}^{\mathrm {J}/\psi }$, determined [399] from fixed-target measurements and using the EKS98 nPDFs [400, 401]

The $\mathrm {J}/\psi$ absorption cross section, $\sigma _\mathrm{abs}^{\mathrm {J}/\psi }$, was traditionally assumed to be independent of the production kinematics until measurements covering broad phase-space regions showed clear dependences of the nuclear effects on $x_{\mathrm {F}}$ and $p_{\mathrm {T}}$. It was further assumed to be independent of collision centre-of-mass energy, $\sqrt{s_{\mathrm{NN}}}$, neglecting any nuclear effects on the parton distributions. However, $\mathrm {J}/\psi$ production is sensitive to the gluon distribution in the nucleus and the fixed-target measurements probe parton momentum fractions, *x*, in the possible anti-shadowing region. This effect may enhance the $\mathrm {J}/\psi$ production rate at mid-rapidity and a larger absorption cross section would be required to match the data.

If one focuses on the behavior of $\mathrm {J}/\psi$ production at $x_{\mathrm {F}} \approx 0$, the absorption cross section is found to depend on $\sqrt{s_{\mathrm{NN}}}$, essentially independent of the chosen nPDF parametrisation [399], as shown in Fig. 32 (left). The yellow band represents the uncertainty corresponding to an empirical power-law fit (solid curve) to all the data points analysed in [399] from measurements by NA3 [332], NA50 [341, 342], E866 [352], HERA-B [359], NA60 [406] and PHENIX [407]. The extrapolation of the power-law fit in Fig. 32 (left) to the current LHC p–A energy leads to a vanishingly small cross section within the illustrated uncertainties.

Away from mid-rapidity, the extracted $\sigma _\mathrm{abs}^{\mathrm {J}/\psi }$ grows with $x_{\mathrm {F}}$ up to unrealistically large values, as shown in Fig. 32 (right). This seems to indicate that another mechanism, in addition to absorption and shadowing, such as initial-state energy loss, may be responsible for the $\mathrm {J}/\psi$ suppression in the forward region ($x_{\mathrm {F}} > 0.25$). This confirms that the effective parameter $\sigma _\mathrm{abs}^{\mathrm {J}/\psi }$ should not be interpreted as a genuine inelastic cross section. It seems that the rise starts closer to $x_{\mathrm {F}} = 0$ for lower collision energies [64]. More recent analyses [408], using EPS09 [364], are in general agreement with the results of Ref. [399].

Despite different conclusions on the possible energy dependence of $\sigma _\mathrm{abs}$ from fixed-target experiments to RHIC energy in [405] and [399], one expects nuclear absorption effects to become negligible at the LHC since the quarkonium formation time becomes significantly larger than the nuclear size at all values of the rapidity. This is also confirmed by a more recent analysis. In Ref. [408], the authors show that the $\mathrm {J}/\psi$ suppression seems to scale with the crossing time $\tau _\mathrm{cross}$ (see Sect. 3.2.1), independently of the centre-of-mass energy, above a typical crossing time $\tau _\mathrm{cross} \gtrsim 0.05$ fm/*c*. Below this scale, however, the lack of scaling indicates that nuclear absorption is probably not the dominant effect. Using the $2\rightarrow 1$ kinematics, $\tau _\mathrm{cross} \simeq 2 m_p\ L\ e^{-y} / \sqrt{s_{\mathrm{NN}}}$, the condition $\tau _\mathrm{cross} < 0.05$ fm/*c* would correspond to $y > - 3.8$ (using $L_\mathrm{Pb}\simeq 3/4\ R_\mathrm{Pb} \simeq 5$ fm) at the LHC.

#### Summary of CNM models

A brief summary of these different approaches is given in Table 8, in which the dominant physical effects and ingredients used in each calculation are given. The model acronyms given in the table match those in the legends of the figures in the next section.

**Table 8:** Summary of the various models of CNM approaches discussed in the text and compared to data in Sect. 3.3. The main physical processes and ingredients used in each calculation are listed

Acronym	Production mechanism	Medium effects	Main parameters	References
Open heavy flavour				
pQCD$+$EPS09 LO	pQCD LO	nPDF	4$+$1 EPS09 LO sets	[368]
SAT	pQCD LO$+$CGC	Saturation	$Q_{s,p}^2(x_0)$, $Q_{s,A}^2(x_0)$	[378]
ELOSS	pQCD LO	E. loss, power cor., broa.	$\epsilon _a$, $\xi _d$, $\mu ^2$, $\lambda$	[391]
Quarkonia				
EXT$+$EKS98LO$+$ABS	Generic $2\rightarrow 2$ LO	nPDF and absorption	EKS98 LO, $\sigma _\mathrm {abs}$	[374, 375]
EXT$+$EPS09 LO	Generic $2\rightarrow 2$ LO	nPDF	4$+$1 EPS09 LO sets	[376, 377]
CEM$+$EPS09 NLO	CEM NLO	nPDF	30$+$1 EPS09 NLO sets	[363]
SAT	CEM LO$+$CGC	Saturation	$Q_{s,p}^2(x_0)$, $Q_{s,A}^2(x_0)$	[379]
ELOSS	NRQCD LO	E. loss, power cor.	$\epsilon _a$, $\xi _d$, $\mu ^2$, $\lambda$	[83]
COH.ELOSS	$\mathrm pp$ data	Coherent E. loss	$\hat{q}$	[395, 396]
KPS	dipole model	Dipole absorption	$\sigma _{c\bar{c}}$	[385, 386]

### Recent RHIC and LHC results

In this section we summarise the recent measurements in p–A collisions at RHIC and at the LHC. Open heavy-flavour results are described in Sect. 3.3.2 and hidden heavy-flavour data in Sect. 3.3.3. As described in the previous section, in order to understand the role of the CNM effects, the interpretation of these measurements is commonly obtained by a comparison with measurements in $\mathrm pp$ collisions at the same centre-of-mass energy as for p–A and in the same rapidity interval. At the LHC, so far it has not been possible to carry out $\mathrm pp$ measurements at the same energy and rapidity as for p–Pb. In Sect. 3.3.1 the procedures to define the pp reference for $R_\mathrm{pA}$ are described.

#### Reference for p–A measurements at the LHC

The $\mathrm pp$ reference for open heavy-flavour measurements at $\sqrt{s} =5.02$ $\text {TeV}$ was obtained either from pQCD calculations or by a pQCD-based $\sqrt{s}$-scaling of the measurements performed at $\sqrt{s} =7$ $\text {TeV}$. In some cases, it was also possible to evaluate the $\sqrt{s}$-scaled spectra of both the 7 and the 2.76 $\text {TeV}$ data to $\sqrt{s} =5.02$ $\text {TeV}$ and combine them. The pQCD-based $\sqrt{s}$-scaling procedure is described in reference [409]. The scaling factor is evaluated as the ratio of the theoretical calculation at the two energies. The scaling uncertainties are determined considering the prediction uncertainties, the variation of the renormalisation and factorisation scales, the heavy-quark mass and the PDF uncertainties. The assumption behind this calculation is that the values of these parameters remain the same at both energies. The scaling factor and uncertainties computed with different heavy-quark production models, FONLL [44, 99] and GM-VFNS [16, 410], are in excellent agreement. This procedure was verified by comparing the D-meson CDF measurements at $\sqrt{s} =1.96~\text {TeV}$ at mid-rapidity to a $\sqrt{s}$-scaling of the ALICE data [409]. A different strategy was used in order to evaluate the $\mathrm pp$ reference for $\mathrm {J}/\psi$ from B decays: the procedure is the same as for $\mathrm {J}/\psi$ at forward rapidity and it is described in the following.

**Fig. 33:**
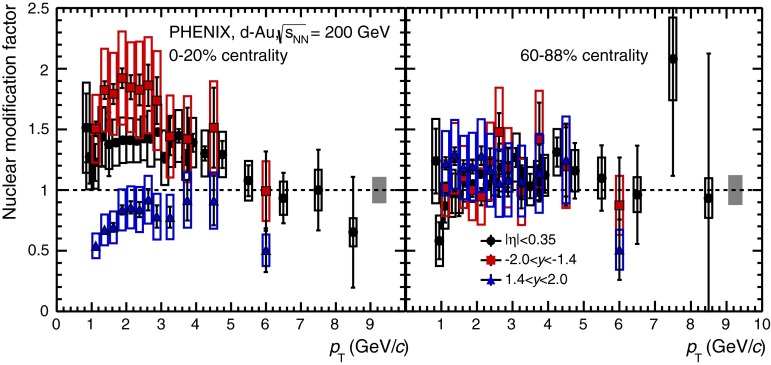
Nuclear modification factor of heavy-flavour decay leptons in d–Au collisions at $\sqrt{s_{\mathrm{NN}}} =200$ $~\text {GeV}$ as a function of transverse momentum in the 0–20 and 60–88 % centrality classes, as measured with the PHENIX detector [313, 314]

In the quarkonium analyses, different strategies have been adopted depending on the precision of the existing measurements. They are mainly based on phenomenological functions and are briefly described in the following.

At mid-rapidity in ALICE, the $$\mathrm {J}/\psi $$$$\mathrm pp$$ integrated cross section reference has been obtained by performing an interpolation based on $$\mathrm {J}/\psi $$ measurements at mid-rapidity in $$\mathrm pp$$ collisions at $$\sqrt{s}$$  $$=$$ 200$$~\text {GeV}$$  [411], 2.76 $$\text {TeV}$$  [412] and 7 $$\text {TeV}$$  [413], and in $${\mathrm{p}\overline{\mathrm{p}}} $$ collisions at $$\sqrt{s}$$  $$=$$ 1.96 $$\text {TeV}$$  [414]. Several functions (linear, power law and exponential) were used to parametrise the cross section dependence as a function of $$\sqrt{s}$$. The interpolation leads to a total uncertainty of $$17~\%$$ on the integrated cross section. The effect of the asymmetric rapidity coverage, due to the shift of the rapidity by 0.465 in the centre-of-mass system in p–Pb collisions at the LHC, was found to be negligible as compared to the overall uncertainty of the interpolation procedure. Then the same method as described in [415] was followed to obtain the $$p_{\mathrm {T}}$$-dependent cross section. The method is based on the empirical observation that the $$\mathrm {J}/\psi $$ cross sections measured at different energy and rapidity scale with $$p_{\mathrm {T}}$$/$$\langle p_{\mathrm {T}} \rangle $$. The $$\langle p_{\mathrm {T}} \rangle $$ value was evaluated at $$\sqrt{s}$$  $$=$$ 5.02 TeV by an interpolation of the $$\langle p_{\mathrm {T}} \rangle $$ measured at mid-rapidity [411, 413, 414] using exponential, logarithmic and power-law functions.

At forward rapidity, a similar procedure for the $\mathrm {J}/\psi$ cross section interpolation has been adopted by ALICE and LHCb and is described in [416]. In order to ease the treatment of the systematics correlated with energy, the interpolation was limited to results obtained with a single apparatus. The inclusive $\mathrm {J}/\psi$ cross sections measured at 2.76 [412] and 7 $\text {TeV}$  [199] were included in the ALICE procedure while the inclusive, prompt $\mathrm {J}/\psi$ and $\mathrm {J}/\psi$ from B-mesons cross sections measured at 2.76 [417], 7 [172] and 8 $\text {TeV}$  [206] were considered in the LHCb one. The interpolation of the cross section with energy is based, as in the mid-rapidity case, on three empirical shapes (linear, power law and exponential). The resulting interpolated cross section for inclusive $\mathrm {J}/\psi$ obtained by ALICE and LHCb in $2.5<y <4$ at $\sqrt{s}$  $=$ 5.02 $\text {TeV}$ were found to be in good agreement with a total uncertainty of $\sim$8  and $\sim$5 % for ALICE and LHCb, respectively. The interpolation in $\sqrt{s}$ was also performed by ALICE independently for each $p_{\mathrm {T}}$ interval and outside of the rapidity range of $\mathrm pp$ data in order to cope with the p–Pb centre-of-mass rapidity shift. In that case an additional interpolation with rapidity was carried out by using several empirical functions (Gaussian, second- and fourth-order polynomials).

In the case of the $\Upsilon$ at forward rapidity, the interpolation procedure results also from a common approach by ALICE and LHCb and is described in [418]. It is based on LHCb measurements in $\mathrm pp$ collisions at 2.76 $\text {TeV}$  [419], 7 $\text {TeV}$  [199] and 8 $\text {TeV}$  [206]. Various phenomenological functions and/or the $\sqrt{s}$-dependence of the CEM and FONLL models are used for the $\sqrt{s}$-dependence of the cross section, similarly to the $\mathrm {J}/\psi$ interpolation procedure at forward rapidity. This interpolation results in a systematic uncertainty that ranges from 8 to $12~\%$ depending on the rapidity interval.

#### Open heavy-flavour measurements

Open heavy-flavour production occurs in hard processes at the early stages of the collision (see Sect. 2.1.1 for an introduction to the different calculations). As explained in Sect. 3.2, their production in a nuclear environment is affected by the modification of the parton probability density in the nucleus (nPDFs or parton saturation formalisms) and by the multiple scattering of partons in the nucleus (radiative or collisional parton energy loss, $k_{\mathrm {T}}$ broadening). Due to their short lifetimes, open heavy-flavour hadrons are measured via their decay products. Different analyses methods exist: (i) study leptons from heavy-flavour decays, (ii) examine the $p_{\mathrm {T}}$-integrated dilepton invariant-mass distribution, to evaluate the charm and beauty cross sections, (iii) fully reconstruct exclusive decays, such as $\mathrm {D}^{0} \rightarrow \mathrm{K}^{+} \, \pi ^{-}$ or $\mathrm{B}^0 \rightarrow \mathrm {J}/\psi \, \mathrm{K}^0_\mathrm{S}$, (iv) select specific (semi-)inclusive decays with a displaced vertex topology, such as beauty decays to leptons or $\mathrm {J}/\psi$, (v) identify *c*- or *b*-jets from reconstructed jets, (vi) inspect heavy-flavour azimuthal correlations. In the analyses where not all the decay products are reconstructed, the correlation between the heavy-flavour hadron kinematics and that of the decay particles has to be considered to properly interpret the measurements.

**Fig. 34:**
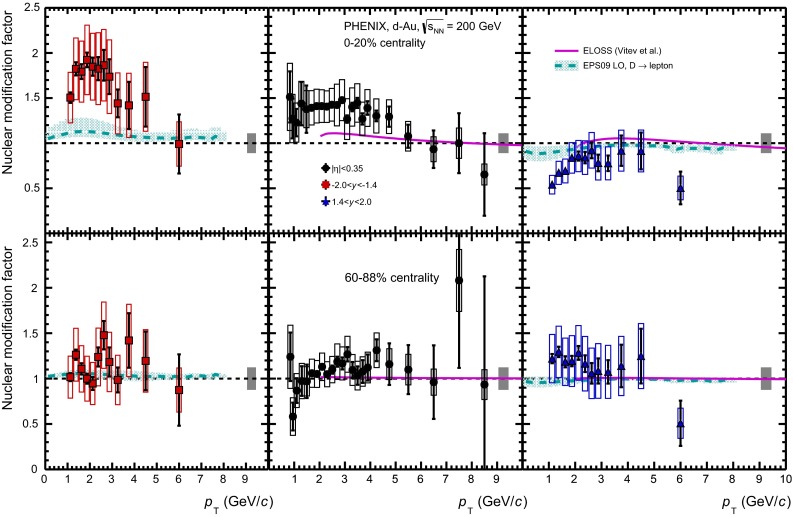
Nuclear modification factor of heavy-flavour decay leptons in d–Au collisions at $\sqrt{s_{\mathrm{NN}}} =200$ $~\text {GeV}$ as a function of transverse momentum in the 0–20 and 60–88 % centrality classes, as measured with the PHENIX detector [313, 314]. A PYTHIA calculation considering EPS09 LO is also shown, courtesy of Sanghoon Lim. The calculation by Vitev et al. considering nPDFs, $k_{\mathrm {T}}$ broadening and CNM energy loss is also shown [391, 392]

*Heavy-flavour decay leptons* The production of heavy-flavour decay leptons, i.e. leptons from charm and beauty decays, has been studied at RHIC and at LHC energies in d–Au and p–Pb collisions at $$\sqrt{s_{\mathrm{NN}}} =200$$$$~\text {GeV}$$ and 5.02 $$\text {TeV}$$, respectively. The p–A measurements exploit the inclusive lepton $$p_{\mathrm {T}} $$-spectrum, electrons at mid-rapidity ($$|\eta |<0.5$$ for PHENIX, $$0<\eta <0.7$$ for STAR and $$|\eta |<0.6$$ for ALICE) and muons at forward rapidities ($$1.4<|\eta |<2.0$$ for PHENIX and $$2.5<\eta <4.0$$ for ALICE). The heavy-flavour decay spectrum is determined by extracting the non-heavy-flavour contribution to the inclusive lepton distribution. The photonic background sources are electrons from photon conversions in the detector material and $$\pi ^0$$ and $$\eta $$ Dalitz decays, which involve virtual photon conversion. The contribution of photon conversions is evaluated with the invariant-mass method or via Montecarlo simulations. The Dalitz decays contribution can be determined considering the measured $$\pi ^0$$ and $$\eta $$ distributions. Background from light hadrons, hard processes (prompt photons and Drell–Yan) and quarkonia is determined with Montecarlo simulations, based, when possible, on the measured spectrum. STAR data is not corrected for the of $$\mathrm {J}/\psi $$ decays contribution, which is non-negligible at high $$p_{\mathrm {T}} $$. Beauty decay-electron spectra can be obtained from the heavy-flavour decay-electron spectra by a cut or fit of the lepton impact-parameter distribution, i.e. the distance between the lepton track and the interaction vertex, or exploiting the lepton azimuthal correlation to heavy flavours or charged hadrons. For the latter see the last paragraph of this section.

Heavy-flavour decay lepton $$R_{\mathrm {dAu}} $$ measurements at mid-rapidity in minimum-bias d–Au collisions at $$\sqrt{s_{\mathrm{NN}}} =200$$$$~\text {GeV}$$ by STAR and PHENIX [313, 420] are consistent and suggest no modification of the multiplicity-integrated yields for $$1<p_{\mathrm {T}} <10~\text {GeV}/c $$ within uncertainties. The $$p_{\mathrm {T}} $$ dependence of $$R_{\mathrm {dAu}} $$ on the multiplicity and the rapidity was studied by PHENIX [313, 314] and is reported in Fig. 33. It shows a mild dependence with the multiplicity at mid-rapidity. The results at forward and backward rapidities are similar for peripheral collisions, but evidence a strong deviation for the most central events. As shown in Fig. 34 and in [313], the measurements at forward rapidity are described both by the model of Vitev et al. [391, 392] – considering nPDFs, $$k_{\mathrm {T}} $$ broadening and CNM energy loss – (ELOSS model described in Sect. 3.2.4) or by nPDFs alone. Data at backward rapidity cannot be described considering only the nPDFs, suggesting that other mechanisms are at work.

The preliminary results at LHC energies by the ALICE Collaboration [421] present $R_{\mathrm {pPb}}$ multiplicity-integrated values close to unity at mid-rapidity, as observed at lower energies. The rapidity dependence of the multiplicity-integrated $R_{\mathrm {pPb}}$ is also similar to that observed at RHIC. In contrast to RHIC, model calculations with nPDFs present a fair agreement with LHC data. The first preliminary measurements of the beauty-hadron decay-electron $R_{\mathrm {pPb}}$ at mid-rapidity by ALICE are consistent with unity within larger uncertainties [421].

The similar behaviour of RHIC and LHC heavy-flavour decay lepton $R_{\mathrm {pA}}$, within the large uncertainties, despite the different *x*-Bjorken ranges covered, suggests that nPDFs might not be the dominant effect in heavy-flavour production. Additional mechanisms like $k_\mathrm{T}$-broadening, initial- or final-state energy loss could be at play.

**Fig. 35:**
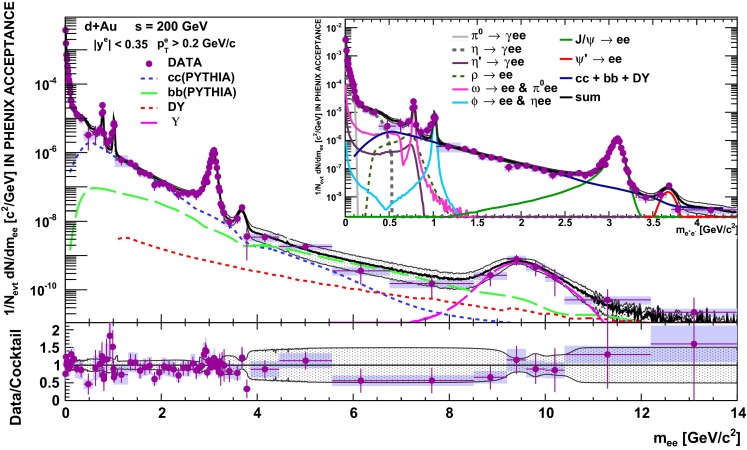
Inclusive $$e^{+}e^{-} $$ pair yield from minimum bias d–Au collisions at $$\sqrt{s_{\mathrm{NN}}} =200$$ $$~\text {GeV}$$ as a function of dilepton invariant mass [315]. The data are compared to the PHENIX model of expected sources. The *inset* shows in detail the mass range up to $$4.5~\text {GeV}/c ^{2}$$. In the *lower panel*, the ratio of data to expected sources is shown with systematic uncertainties

*Dilepton invariant mass* The $${c\overline{c}} $$ and $${b\overline{b}} $$ production cross sections can be obtained by a fit of the $$p_{\mathrm {T}} $$-integrated dilepton yields as a function of the pair mass. Such measurement has been performed by PHENIX at mid-rapidity in d–Au collisions [315] at $$\sqrt{s_{\mathrm{NN}}} =200$$$$~\text {GeV}$$ (see Fig. 35). The contributions of pseudo-scalar mesons, $$\pi ^0$$ and $$\eta $$, and vector mesons, $$\omega $$, $$\phi $$, $$\mathrm {J}/\psi $$ and $$\Upsilon $$ were simulated based on the measured d–Au cross sections. The sources not directly measured ($$\eta ^{\prime }$$, $$\rho $$, $$\psi ^{\prime }$$) were studied in simulation and their contribution determined relative to the measured particles. The Drell–Yan mechanism contribution was simulated with the PYTHIA event generator, and its normalisation was one of the fit parameters. The resulting $${b\overline{b}} $$ production cross section is $$\mathrm{d}\sigma _{{b\overline{b}}}/ \mathrm{d}y = 0.54 \pm 0.11 \, \mathrm { (stat)} \pm 0.18 \, \mathrm { (syst)}$$ mb. The large model dependence prevents an accurate measurement of $$\sigma _{{c\overline{c}}}$$.

**Fig. 36:**
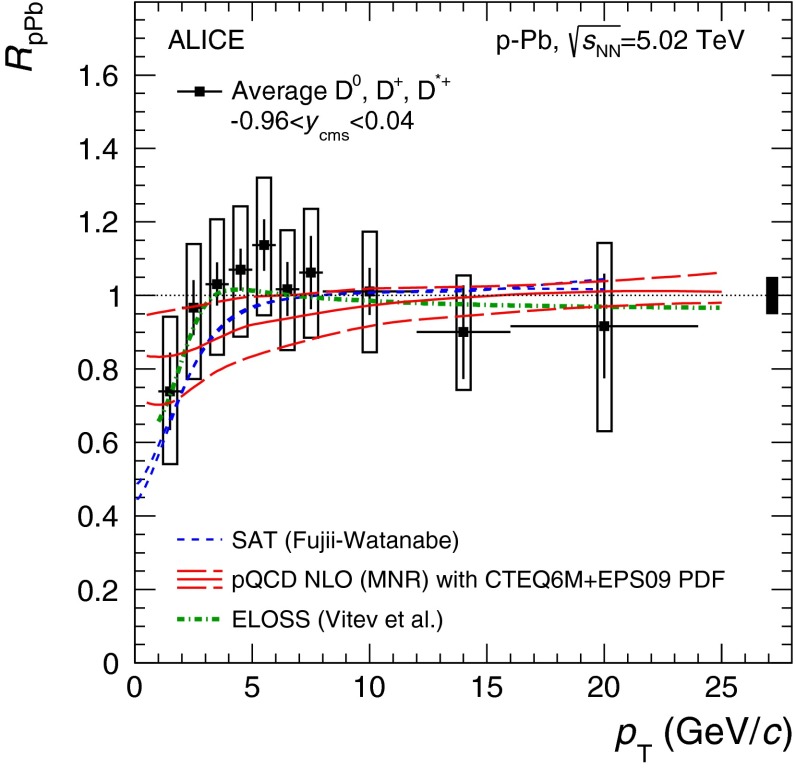
Nuclear modification factor of prompt D mesons in p–Pb collisions at $\sqrt{s_{\mathrm{NN}}} =5.02$ $\text {TeV}$ as a function of transverse momentum as measured with the ALICE detector [324]. The measurements are compared with theoretical calculations including various CNM effects

*D mesons* The $p_{\mathrm {T}}$-differential production cross section of $\mathrm {D}^{0}$, $\mathrm {D}^{+}$, $\mathrm {D}^{*+}$ and $\mathrm {D}^{+}_{s}$ in minimum bias p–Pb collisions at $\sqrt{s_{\mathrm{NN}}} =5.02$ TeV for $|y_\mathrm{lab}|<0.5$ was published in [324] by ALICE. D mesons are reconstructed via their hadronic decays in different $p_{\mathrm {T}}$ intervals from $1~\text {GeV}/c$ up to $24~\text {GeV}/c$. Prompt D-meson yields are obtained by subtracting the contribution of secondaries from B-hadron decays, determined using pQCD-based estimates [125, 324]. No significant variation of the $R_{\mathrm {pPb}}$ among the D-meson species is observed within uncertainties. The multiplicity-integrated prompt D (average of $\mathrm {D}^{0}$, $\mathrm {D}^{+}$ and $\mathrm {D}^{*+}$) meson $R_{\mathrm {pPb}}$ is shown in Fig. 36 together with model calculations. $R_{\mathrm {pPb}}$ is compatible with unity in the measurement $p_{\mathrm {T}}$ interval, indicating smaller than 10–20 % nuclear effects for $p_{\mathrm {T}} >2~\text {GeV}/c$. Data are described by calculations considering only initial-state effects: NLO pQCD estimates (MNR [6]) considering EPS09 nPDFs [364] or Colour Glass Condensate computations [378] (SAT model described in Sect. 3.2.3). Predictions including nPDFs, initial- or final-state energy loss and $k_{\mathrm {T}}$-broadening [422] (ELOSS model discussed in Sect. 3.2.4) also describe the measurements.

Preliminary measurements of the prompt D-meson production as a function of the multiplicity were performed by ALICE [423]. The nuclear modification factor of D mesons was evaluated as a function of the event activity, defined in intervals of multiplicity measured in different rapidity intervals. No event-activity dependence is observed within uncertainties. D meson production has also been studied as a function of charged-particle multiplicity. The D-meson per-event yields increase as a function of the multiplicity at mid-rapidity. The enhancement of the relative D-meson yields is similar to that of $\mathrm pp$ collisions at $\sqrt{s} =7$ $\text {TeV}$, described in Sect. 2.4.1. The results in $\mathrm pp$ collisions favour the scenarios including the contribution of multiple-parton interactions (MPI), parton-percolation or hydrodynamic effects. In p–Pb collisions, the cold nuclear matter effects and the contribution of multiple binary nucleon collisions should also be taken into account.

**Fig. 37:**
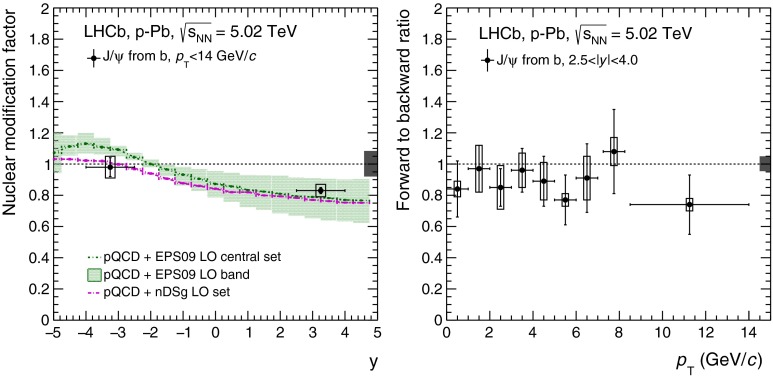
LHCb measurements of non-prompt $\mathrm {J}/\psi$ mesons in p–Pb collisions at $\sqrt{s_{\mathrm{NN}}} =5.02$ $\text {TeV}$  [330]. *Left* nuclear modification factor as a function of rapidity, compared to nPDF-based calculations [368]. *Right* forward- to backward-rapidity ratio as a function of transverse momentum

*Open beauty measurements* The first measurements of the beauty production cross section in p–A collisions down to $$p_{\mathrm {T}} =0$$ were carried out by LHCb in p–Pb collisions at $$\sqrt{s_{\mathrm{NN}}} =5.02$$ TeV [330]. These results were achieved via the analysis of non-prompt $$\mathrm {J}/\psi $$ mesons at large rapidities, $$2<y_{\mathrm {lab}} <4.5$$. $$\mathrm {J}/\psi $$ mesons were reconstructed by an invariant mass analysis of opposite sign muon pairs. The fraction of $$\mathrm {J}/\psi $$ originating with beauty decays, or non-prompt $$\mathrm {J}/\psi $$ fraction, was evaluated from a fit of the component of the pseudo-proper decay time of the $$\mathrm {J}/\psi $$ along the beam direction. The $$R_{\mathrm {pPb}} $$ of non-prompt $$\mathrm {J}/\psi $$ was computed considering as $$\mathrm pp$$ reference an interpolation of the measurements performed in the same rapidity interval at $$\sqrt{s} =2.76$$, 7 and 8 $$\text {TeV}$$ (see Sect. 3.3.1). Figure 37 (left) reports the $$p_{\mathrm {T}} $$-integrated $$R_{\mathrm {pPb}} $$ as a function of rapidity, whereas Fig. 37 (right) presents the double ratio of the production cross section at positive and negative rapidities, $$R_{\mathrm {FB}} $$, as a function of the $$\mathrm {J}/\psi $$ transverse momentum. The $$p_{\mathrm {T}} $$-integrated $$R_{\mathrm {pPb}} $$ is close to unity in the backward-rapidity range, and shows a modest suppression in the forward-rapidity region. $$R_{\mathrm {FB}} $$ is compatible with unity within the uncertainties in the measured $$p_{\mathrm {T}} $$ interval, with values almost systematically smaller than unity. These results indicate a moderate rapidity asymmetry and are consistent with the $$R_{\mathrm {pPb}} $$ ones. The results are in agreement with LO pQCD calculations including EPS09 or nDSg nuclear PDF parametrisations. The ATLAS Collaboration has also measured the $$R_{\mathrm {FB}} $$ of non-prompt $$\mathrm {J}/\psi $$ for $$8<p_{\mathrm {T}} <30~\text {GeV}/c $$ and $$|y |<1.94$$ [329]. These results are consistent with unity within experimental uncertainties and no significant $$p_{\mathrm {T}} $$ or $$y $$ dependence is observed within the measured kinematic ranges.

A preliminary measurement of the production of B mesons in p–Pb collisions at $$\sqrt{s_{\mathrm{NN}}} =5.02$$ $$\text {TeV}$$ was carried out by the CMS Collaboration [424, 425]. B$$^0$$, B$$^{+}$$ and B$$_s^0$$ mesons are reconstructed via their decays to $$\mathrm {J}/\psi + \mathrm{K}$$ or $$\phi $$ at mid-rapidity for $$10<p_{\mathrm {T}} <60~\text {GeV}/c $$. The $$\mathrm{d}\sigma /\mathrm{d}p_{\mathrm {T}} $$ of B$$^0$$, B$$^{+}$$ and B$$_s^0$$ are described within uncertainties by FONLL predictions scaled by the number of nucleons in the nucleus. B$$^{+}$$$$\mathrm{d}\sigma /\mathrm{d}y $$ is also described by FONLL binary-scaled calculations, and presents no evidence of rapidity asymmetry within the measurement uncertainties. These results suggest that B-hadron production for $$p_{\mathrm {T}} >10~\text {GeV}/c $$ is not affected, or mildly, by CNM effects.

Preliminary results of the $p_{\mathrm {T}}$ and $\eta$ differential cross section of *b*-jets in p–Pb collisions at $\sqrt{s_{\mathrm{NN}}} =5.02$ $\text {TeV}$ have been reported by CMS at mid-rapidity [426]. Jets from *b*-quark fragmentation are identified studying the distribution of secondary vertices, typically displaced by several mm for jets of $p_{\mathrm {T}} \sim 100~\text {GeV}/c$. The measured *b*-jet fraction for $50< p_{\mathrm {T}} ^{b-jet}<400~\text {GeV}/c$ is consistent with PYTHIA simulations with the Z2 tune [45, 151]. The $p_{\mathrm {T}}$- and $\eta$-differential spectra are also described by binary-scaled PYTHIA simulations within uncertainties. $R_{\mathrm {pPb}}$ is computed using PYTHIA as $\mathrm pp$ reference and is compatible with unity. These results conform with the expectations that cold nuclear matter effects are not sizeable at large $p_{\mathrm {T}}$.

**Fig. 38:**
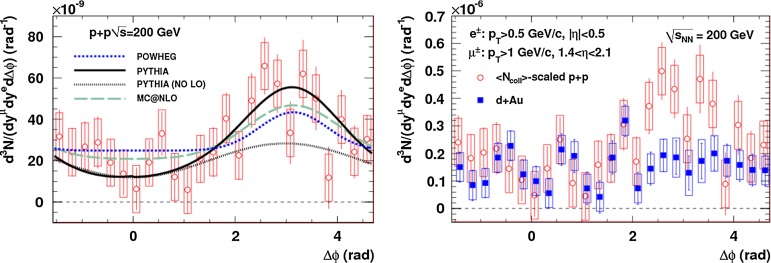
Heavy-flavour decay electron ($p_{\mathrm {T}} >0.5~\text {GeV}/c$, $|\eta |<0.5$) to heavy-flavour decay muon ($p_{\mathrm {T}} >1~\text {GeV}/c$, $1.4<\eta <2.1$) $\Delta \phi$ correlations in $\mathrm pp$ (*left*) and d–Au (*right*) collisions at $\sqrt{s} =200$ $~\text {GeV}$  [114]. The $\mathrm pp$ results are compared to POWHEG, PYTHIA and MCNLO calculations

**Fig. 39:**
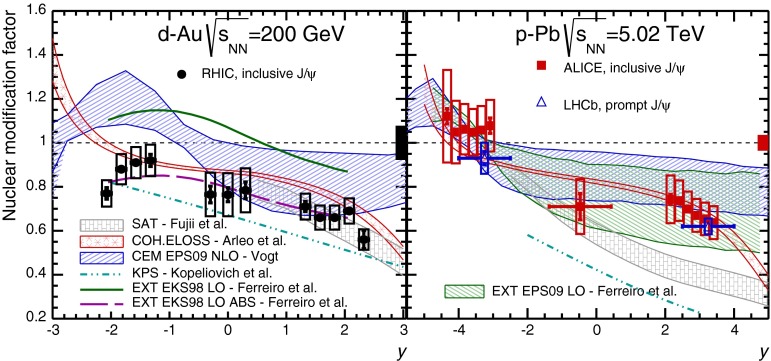
*Left* rapidity dependence of $R_{\mathrm {dAu}}$ for inclusive $\mathrm {J}/\psi$ in PHENIX [318]. The *error bars* represent the uncorrelated uncertainties (statistical and systematic), the *open boxes* the point-to-point correlated systematic uncertainties and the *box at unity* the correlated one. *Right* rapidity dependence of $R_{\mathrm {pPb}}$ for inclusive $\mathrm {J}/\psi$ in ALICE [325, 327] and prompt $\mathrm {J}/\psi$ in LHCb [330]. The *error bars* represent the statistical uncertainties while the *open boxes* the systematic uncertainties. Other uncertainties are displayed similarly to PHENIX

*Heavy-flavour azimuthal correlations* As described in Sect. 2.4.2, heavy-flavour particle production inherits the heavy-quark pair correlation, bringing information on the production mechanisms. Heavy-flavour production in p–A collisions is influenced by initial- and/or final-state effects. The modification of the PDFs or the saturation of the gluon wave function in the nucleus predict a reduction of the overall particle yields. The CGC formalism also predicts a broadening and suppression of the two-particle away-side azimuthal correlations, more prominent at forward rapidities [427–429]. Energy loss or multiple scattering processes in the initial or final state are also expected to cause a depletion of the two-particle correlation away-side yields [430]. These effects could also affect heavy-flavour correlations in p–A collisions.

Heavy-flavour decay electron ($$p_{\mathrm {T}} >0.5~\text {GeV}/c $$, $$|\eta |<0.5$$) to heavy-flavour decay muon ($$p_{\mathrm {T}} >1~\text {GeV}/c $$, $$1.4<\eta <2.1$$) $$\Delta \phi $$ azimuthal correlations have been studied by PHENIX in $$\mathrm pp$$ and d–Au collisions at $$\sqrt{s} =200$$$$~\text {GeV}$$  [114]. They exploit the forward-rapidity muon measurements in order to probe the low-*x* region in the gold nucleus. The analysis considers the angular correlations of all sign combinations of electron–muon pairs. The contribution from light-flavour decays and conversions is removed by subtracting the like-sign yield from the unlike-sign yield. Figure 38 presents the electron–muon heavy-flavour decay $$\Delta \phi $$ correlations. Model calculations are compared to data for $$\mathrm pp$$ collisions; see Fig. 38 (left). Calculations from NLO generators seem to fit better the $$\Delta \phi $$ distribution than LO simulations. The corresponding measurement in d–Au collision; see Fig. 38 (right), shows a reduction of the away-side peak as compared to $$\mathrm pp$$ scaled data, indicating a modification of the charm kinematics due to CNM effects.

Preliminary results of D-hadron azimuthal correlations in p–Pb collisions at $\sqrt{s_{\mathrm{NN}}} =5.02$ $\text {TeV}$ were carried out by the ALICE Collaboration [276]. The measurement uncertainties do not allow a clear conclusion to be drawn on a possible modification of heavy-quark azimuthal correlations with respect to pp collisions.

#### Quarkonium measurements

Quarkonia are mainly measured via their leptonic decay channels. In the PHENIX experiment, the Ring Imaging Cherenkov associated with the Electromagnetic Calorimeter (EMCAL) allows one to identify electrons at mid-rapidity ($|y| < 0.35$). In this rapidity range where the EMCAL can reconstruct the photons, $\chi _c$ can also be measured from its decay channel to $\mathrm {J}/\psi$ and photon. At backward and forward rapidity ($1.2 < |y| < 2.2$), two muon spectrometers allow for the reconstruction of quarkonia via their muonic decay channel. In the STAR experiment, quarkonia are reconstructed at mid-rapidity ($|y| < 1$) thanks to the electron identification and momentum measurements from the TPC. In the ALICE experiment, a TPC at mid-rapidity ($|y_{\mathrm {lab}} | < 0.9$) is used for electron reconstruction and identification and a spectrometer at forward rapidity for muon reconstruction ($2.5 < y_{\mathrm {lab}} < 4$). The LHCb experiment is a forward spectrometer that allows for the quarkonium measurement via their muonic decay channel for $2 < y_{\mathrm {lab}} < 4.5$. In the CMS experiment, quarkonia are reconstructed in a large range around mid-rapidity ($|y_{\mathrm {lab}} | < 2.4$) via the muonic decay channel. In LHCb, CMS and in ALICE at mid-rapidity, the separation of prompt $\mathrm {J}/\psi$ from inclusive $\mathrm {J}/\psi$ exploits the long lifetime of *b* hadrons, with $c\tau$ value of about 500 $\upmu$m, using the good resolution of the vertex detector.

*Charmonium* The nuclear modification factor for inclusive and/or prompt $\mathrm {J}/\psi$ has been measured for a large range in rapidity and is shown in Fig. 39 for RHIC (left) and LHC (right). It should be emphasised that there are no $\mathrm pp$ measurements at $\sqrt{s_{\mathrm{NN}}}$  $=$ 5.02 TeV at the LHC and the $\mathrm pp$ cross section interpolation procedure described in Sect. 3.3.1 results in additional uncertainties.

The measurements from PHENIX [318] in d–Au collisions at $$\sqrt{s_{\mathrm{NN}}}$$  $$=$$ 200 $$~\text {GeV}$$ cover four units of rapidity. The $$\mathrm {J}/\psi $$ is suppressed with respect to binary-scaled $$\mathrm pp$$ collisions in the full rapidity range with a suppression that can reach more than 40 % at $$y=2.3$$. Inclusive $$\mathrm {J}/\psi $$ includes a contribution from prompt $$\mathrm {J}/\psi $$ (direct $$\mathrm {J}/\psi $$ and excited charmonium states, $$\chi _c$$ and $$\mathrm \psi (2S)$$) and a contribution from decays of B mesons. At RHIC energy, the contribution from B-meson decays to the inclusive yield is expected to be small, of the order of $$3~\%$$ [431], but has not been measured so far in d–Au collisions. The contribution from excited states such as $$\chi _c$$ and $$\mathrm \psi (2S)$$ has been measured at mid-rapidity [320] and is discussed later in this section. While the inclusive $$\mathrm {J}/\psi $$$$R_{\mathrm {dAu}}$$ for $$ |y| < 0.9$$ is found to be $$0.77\pm 0.02\mathrm { (stat)}\pm 0.16\mathrm { (syst)}$$, the correction from $$\chi _c$$ and $$\mathrm \psi (2S)$$ amounts to $$5~\%$$ and leads to a feed-down corrected $$\mathrm {J}/\psi $$$$R_{\mathrm {dAu}}$$ of $$0.81\pm 0.12\mathrm { (stat)}\pm 0.23\mathrm { (syst)}$$.

At the LHC, the results for inclusive $\mathrm {J}/\psi$ from ALICE [325, 327] and for prompt $\mathrm {J}/\psi$ from LHCb [330] show a larger suppression of the $\mathrm {J}/\psi$ production with respect to the binary-scaled $\mathrm {J}/\psi$ production in $\mathrm pp$ collisions at forward rapidity (40 % at $y =3.5$). In the backward-rapidity region the nuclear modification factor is slightly suppressed (prompt $\mathrm {J}/\psi$ from LHCb) or enhanced (inclusive $\mathrm {J}/\psi$ from ALICE) but within the uncertainties compatible with unity. ATLAS and LHCb have also measured the production of $\mathrm {J}/\psi$ from B mesons [329, 330]: they contribute to the inclusive $\mathrm {J}/\psi$ yield integrated over $p_{\mathrm {T}}$ by 8 % at $-4 < y < -2.5$ and 12 % at $1.5<y <4$ with an increase towards mid-rapidity and high $p_{\mathrm {T}}$ region. At $p_{\mathrm {T}} >8$ GeV/c, the fraction of B mesons can reach up to 34 % at mid-rapidity and up to 26 % in the backward-rapidity region covered by LHCb. In addition, the nuclear modification factor for $\mathrm {J}/\psi$ from B mesons is above 0.8 when integrated over $p_{\mathrm {T}}$, as shown in Fig. 37, At low $p_{\mathrm {T}}$, a small effect from B mesons on inclusive $\mathrm {J}/\psi$ measurements is therefore expected at LHC energy and this is confirmed by the comparison of prompt to inclusive $\mathrm {J}/\psi$ that shows good agreement as seen in the right panel of Fig. 39.

The models based on nuclear PDFs [363, 377, 432] (CEM EPS09 NLO, EXT EKS98 LO and EXT EPS09 LO), gluon saturation [379] (SAT), multiple scattering and energy loss [386, 394] (COH.ELOSS and KPS) described in Sect. 3.2 are also shown in Fig. 39. The uncertainty from the nuclear PDFs on the gluon distribution function is large as discussed in Sect. 3.2.2 and is shown by the uncertainty band of the corresponding calculations. The models based on nPDFs overestimate the data at RHIC in particular at backward rapidity, the anti-shadowing region. At forward rapidity, a strong shadowing with the EPS09 NLO nPDFs parametrisation is favoured by the RHIC data. By including a $\mathrm {J}/\psi$ absorption cross section, $\sigma _\mathrm{abs}^{\mathrm {J}/\psi }=4.2$ mb, the calculation from EXT EKS98 LO ABS that uses EKS98 LO nPDFs can describe the $R_{\mathrm {dAu}}$ measured at RHIC in the full rapidity range. In the latter calculations, since the behaviour of EKS98 is very close to the one of the central set of EPS09 LO, the theoretical curves are expected to be similar to those of EPS09 LO nPDFs. At the LHC, while the backward-rapidity data is well described by the nPDF models, a strong shadowing is favoured by the data at forward rapidity. Both EPS09 LO and the lower uncertainty band of EPS09 NLO parametrisations provide such a strong shadowing. In the COH.ELOSS approach, the rapidity dependence of the nuclear modification factor is well described both at RHIC and LHC energies. In the KPS model, the rapidity dependence of the RHIC data is correctly described but the calculations are systematically lower than the measurements. At LHC energy, the KPS model overestimates the $\mathrm {J}/\psi$ suppression over the full rapidity range. Finally, the SAT model is not valid for the full rapidity range; see Sect. 3.2.3. While it describes correctly the data for $y>0.5$ at $\sqrt{s_{\mathrm{NN}}} =200$ $~\text {GeV}$ and the mid-rapidity data at $\sqrt{s_{\mathrm{NN}}} =5.02$ $\text {TeV}$, it overestimates the $\mathrm {J}/\psi$ suppression at forward rapidity at $\sqrt{s_{\mathrm{NN}}} =5.02$ $\text {TeV}$.

**Fig. 40:**
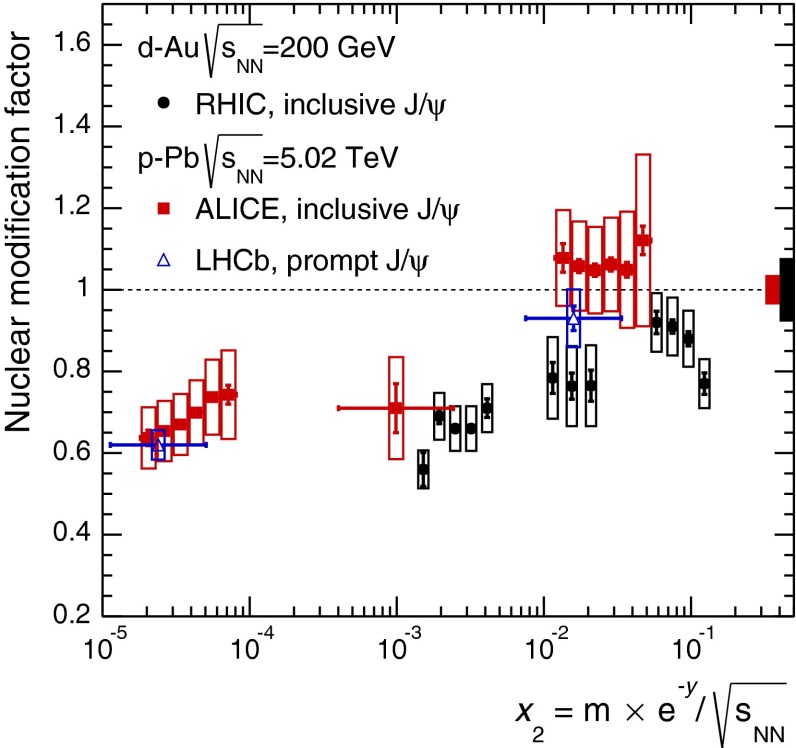
$x_2 = \frac{m}{\sqrt{s_{\mathrm{NN}}}}\exp (-y)$ dependence of $R_{\mathrm {pA}}$ for inclusive J/$\psi$ in PHENIX [318] and ALICE [325, 327] and for prompt $\mathrm {J}/\psi$ in LHCb [330]. The uncertainties are described in Fig. 39

**Fig. 41:**
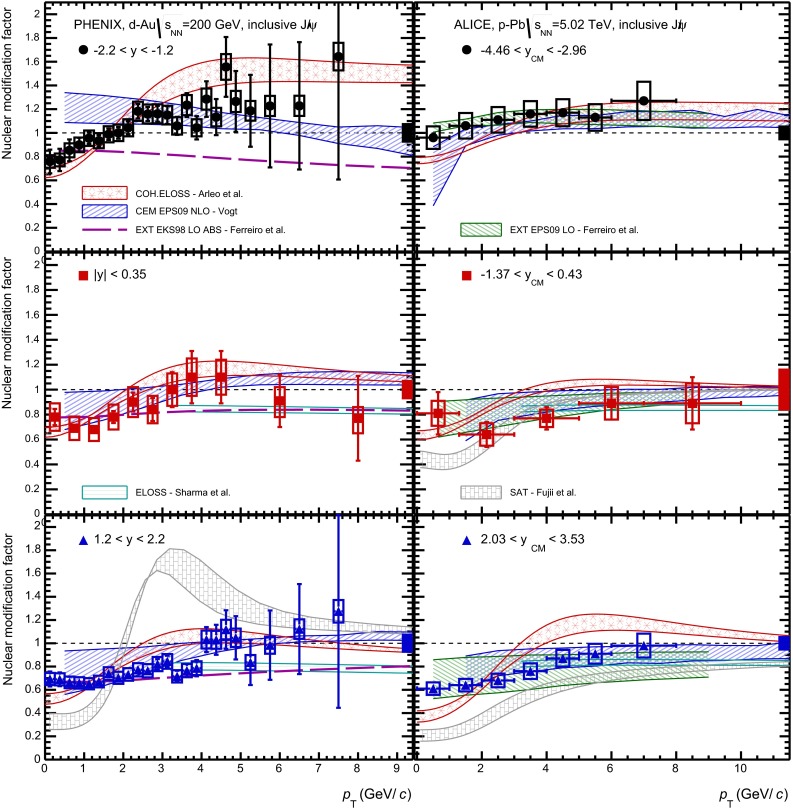
*Left* transverse momentum dependence of $R_{\mathrm {dAu}}$ for three rapidity ranges for inclusive $\mathrm {J}/\psi$ in PHENIX [319]. *Right* transverse momentum dependence of $R_{\mathrm {pPb}}$ for inclusive $\mathrm {J}/\psi$ in ALICE [327]. The uncertainties are the same as described in Fig. 39

It is interesting to check whether a simple scaling exists on $\mathrm {J}/\psi$ suppression between RHIC and LHC. The effects of nPDF or saturation are expected to scale with the momentum fraction $x_2$, independently of the centre-of-mass energy of the collision. It is also the case of nuclear absorption, since the $\mathrm {J}/\psi$ formation time is proportional to the Lorentz factor $\gamma$, which is uniquely related to $x_2$, $\gamma = m / (2 m_p\ x_2)$, assuming $2\rightarrow 1$ kinematics for the production process. In order to test the possible $x_2$ scaling expected in the case of nPDF and nuclear absorption theoretical approaches, the data from RHIC and LHC [318, 325, 327, 330] of Fig. 39 are shown together in Fig. 40 as a function of $x_2 = \frac{m}{\sqrt{s_{\mathrm{NN}}}}\exp (-y)$ where the low $x_2$ values correspond to forward-rapidity data. Note that Eq. (19) for the calculation of $x_2$, which refers to a $2 \rightarrow 2$ partonic process, cannot be used since the $\langle p_{\mathrm {T}} \rangle$ values for all the data points have not been measured. While at $x_2 < 10^{-2}$, the nuclear modification factors are compatible at RHIC and LHC energy within uncertainties, the data presents some tension with a $x_2$ scaling at large $x_2$.

The transverse momentum distribution of the nuclear modification factor is shown for different rapidity ranges in Fig. 41 for RHIC (left) and LHC (right) energies. At $$\sqrt{s_{\mathrm{NN}}}$$  $$=$$ 200 $$~\text {GeV}$$, the $$\mathrm {J}/\psi $$$$R_{\mathrm {dAu}}$$ is suppressed at low $$p_{\mathrm {T}}$$ and increases with $$p_{\mathrm {T}}$$ for the full rapidity range. The mid- and forward rapidity results show a similar behaviour: $$R_{\mathrm {dAu}}$$ increases gradually with $$p_{\mathrm {T}}$$ and is consistent with unity at $$p_{\mathrm {T}} \gtrsim 4$$$$~\text {GeV}/c$$. At backward rapidity, $$R_{\mathrm {dAu}}$$ increases rapidly to reach 1 at $$p_{\mathrm {T}} \approx 1.5$$$$~\text {GeV}/c$$ and is above unity for $$p_{\mathrm {T}} > 2.5$$$$~\text {GeV}/c$$. At $$\sqrt{s_{\mathrm{NN}}}$$  $$=$$ 5.02 $$\text {TeV}$$, a similar shape and amplitude is observed for $$R_{\mathrm {pPb}}$$ at mid- and forward rapidity: in that case it is consistent with unity at $$p_{\mathrm {T}} \gtrsim 5$$$$~\text {GeV}/c$$. The backward-rapidity results are consistent throughout the full $$p_{\mathrm {T}}$$ interval with unity.

In addition to the aforementioned models, the calculations based on the energy-loss approach from [83] (ELOSS), valid for $y \ge 0$ and $p_{\mathrm {T}} > 3$ GeV/c, are also compared to the data. Among these models, only the COH.ELOSS and SAT model includes effects from initial- or final-state multiple scattering that may lead to a $p_{\mathrm {T}}$ broadening. The $p_{\mathrm {T}}$ dependence of $R_{\mathrm {pA}}$ is correctly described by the CEM EPS09 NLO model except at backward rapidity and $\sqrt{s_{\mathrm{NN}}} =200$ $~\text {GeV}$. The model based on EXT EKS98 LO ABS with an absorption cross section of 4.2 mb describes the mid- and forward rapidity results at $\sqrt{s_{\mathrm{NN}}} =200$ $~\text {GeV}$ but not the $p_{\mathrm {T}}$ dependence at backward rapidity. A good agreement is reached at $\sqrt{s_{\mathrm{NN}}} =5.02$ $\text {TeV}$ with CEM EPS09 NLO and EXT EPS09 LO calculations. The ELOSS model describes correctly the $p_{\mathrm {T}}$ dependence at mid- and forward rapidity at both energies. The COH.ELOSS calculations describe correctly the data with, however, a steeper $p_{\mathrm {T}}$ dependence at forward rapidity and $\sqrt{s_{\mathrm{NN}}} =5.02$ TeV. Finally the SAT model gives a good description of the data at mid-rapidity at $\sqrt{s_{\mathrm{NN}}} =5.02$ TeV but does not describe the $p_{\mathrm {T}}$ dependence at forward rapidity at $\sqrt{s_{\mathrm{NN}}} =$200 $~\text {GeV}$ and overestimates the suppression at forward rapidity at $\sqrt{s_{\mathrm{NN}}} =5.02$ TeV.

It is also worth mentioning that the ratio $R_{\mathrm {FB}}$ of the nuclear modification factors for a rapidity range symmetric with respect to $y \sim 0$ has also been extracted as a function of rapidity and $p_{\mathrm {T}}$ at $\sqrt{s_{\mathrm{NN}}} =5.02$ TeV [325, 329, 330]. Despite the reduction of statistics (since the rapidity range is limited), the $\mathrm pp$ cross section and its associated systematics cancels out in the ratio and results on $R_{\mathrm {FB}}$ provides additional constraints to the models.

**Fig. 42:**
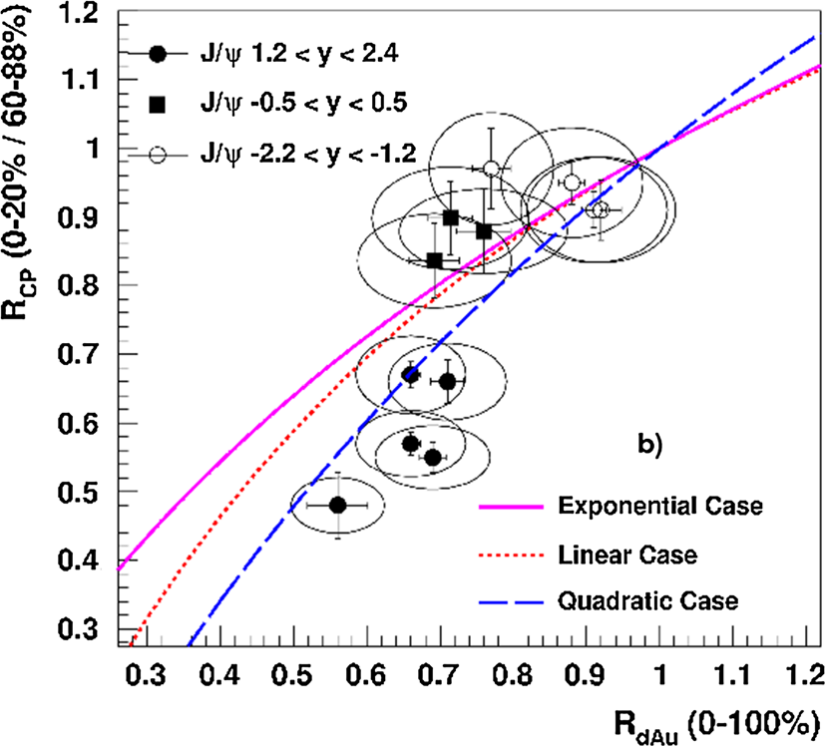
$R_{\mathrm {CP}}$ as a function of $R_{\mathrm {dAu}}$ for inclusive $\mathrm {J}/\psi$ in PHENIX [318]. The *curves* are *constraint* *lines* for three geometric dependencies of the nuclear modification. The *ellipses* represent a one standard deviation contour for the systematic uncertainties

The dependence of the $\mathrm {J}/\psi$ suppression has also been measured in d–Au as a function of the centrality of the collision in PHENIX [318, 319]. The centrality of the d–Au collision is determined thanks to the total energy deposited in the beam-beam counter (BBC) located in the nucleus direction. A larger suppression is observed in central (0–20 %) as compared to peripheral (60–88 %) collisions. In order to study the centrality dependence of the nuclear effect, the nuclear modification factor between central and peripheral collisions, $R_\mathrm{CP}$, has been measured. Figure 42 shows $R_{\mathrm {CP}}$ as a function of $R_{\mathrm {dAu}}$: the forward-rapidity measurements correspond to the lowest $R_{\mathrm {CP}}$ values. The nuclear effect has been parametrised by three functional forms (exponential, linear or quadratic) that depend on the density-weighted longitudinal thickness through the nucleus $\Lambda (r_\mathrm {T})=\frac{1}{\rho _0}\int \mathrm {d}z\rho (z,r_\mathrm {T})$. Here $\rho _0$ is the density in the centre of the nucleus and $r_\mathrm {T}$ the transverse radial position of the nucleon–nucleon collision relative to the centre of the nucleus. While the effect from nuclear absorption is expected to follow an exponential dependence, other models like nPDF assume a linear form to describe the centrality dependence of the nuclear effect. While at backward and mid-rapidity the data cannot discriminate between the functional forms, the forward-rapidity data suggest that the dependence on $\Lambda (r_\mathrm {T})$ is non-linear and closer to quadratic.

**Fig. 43:**
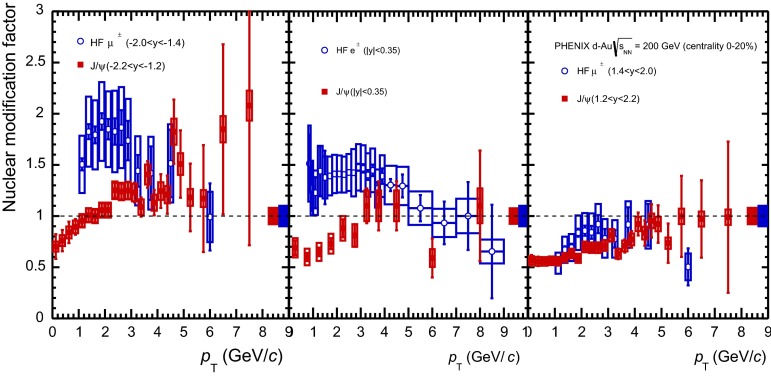
Transverse momentum of $R_{\mathrm {dAu}}$ of inclusive J/$\psi$ for three different rapidity ranges in 0–20 % centrality bin [319] and comparison to heavy flavour electron and muon in PHENIX [313, 314]

The centrality dependence of the $$\mathrm {J}/\psi $$ nuclear modification factor has also been studied in ALICE [433, 434]. In these analyses, the event activity is determined from the energy measured along the beam line by the Zero Degree Neutron (ZN) calorimeter located in the nucleus direction. In the hybrid method described in [435], the centrality of the collision in each ZN energy event class is determined assuming that the charged-particle multiplicity measured at mid-rapidity is proportional to the number of participants in the collision. In the $$\mathrm {J}/\psi $$ case, the data is compatible, within uncertainties, to the binary-scaled $$\mathrm pp$$ production for peripheral events at backward and forward rapidity. The $$\mathrm {J}/\psi $$ production in p–Pb is, however, significantly modified towards central events. At backward rapidity the nuclear modification factor is compatible with unity at low $$p_{\mathrm {T}}$$ and increases with $$p_{\mathrm {T}}$$ reaching about 1.4 at $$p_{\mathrm {T}}$$$$\sim 7$$$$~\text {GeV}/c$$. At forward rapidity the suppression of $$\mathrm {J}/\psi $$ production shows an increase towards central events specially at low $$p_{\mathrm {T}}$$. The $$\mathrm {J}/\psi $$ production was also studied as a function of the relative charged-particle multiplicity measured at mid-rapidity as was already done in $$\mathrm pp$$ collisions [266]. The results show an increase with the relative multiplicity at backward and forward rapidity. At forward rapidity the multiplicity dependence becomes weaker than at backward rapidity for high relative multiplicities. In $$\mathrm pp$$ collisions [266] this increase is interpreted in terms of the hadronic activity accompanying $$\mathrm {J}/\psi $$ production, from contribution of multiple parton–parton interactions or in the parton-percolation scenario. In p–Pb collisions, in addition to the previous contributions, the cold nuclear matter effects should be considered when interpreting these results.

Since open and hidden heavy flavour hadrons are characterised by the same production process for the heavy quark pair, a direct comparison of their productions, if measured over the entire phase space, is expected to single out final-state effects on $$\mathrm {J}/\psi $$ production. In Fig. 43, the $$\mathrm {J}/\psi $$$$R_{\mathrm {dAu}}$$  [319] is compared to the one of open heavy-flavour decay leptons [313, 314] as measured by PHENIX in central d–Au collisions. Despite the fact that the open beauty contribution is not subtracted and the measurement is carried out only down to $$p_{\mathrm {T}}$$$$=$$ 1$$~\text {GeV}/c$$, this comparison may already give some hint on the final-state effects on $$\mathrm {J}/\psi $$ production. A similar behaviour across the entire $$p_{\mathrm {T}}$$ range is observed for $$R_{\mathrm {dAu}}$$ at forward rapidity, suggesting that the suppression of $$\mathrm {J}/\psi $$ production is related to the suppression of $${c\overline{c}}$$ pair production. At backward and mid-rapidity the $$\mathrm {J}/\psi $$ is clearly more suppressed than leptons from open heavy-flavour decays at low $$p_{\mathrm {T}}$$, where the charm contributions dominate over those from bottom [111]. This difference between $$\mathrm {J}/\psi $$ and open charm may originate from additional effects beyond charm quark pair production, such as a longer crossing time $$\tau _\mathrm{cross}$$ of the $${c\overline{c}}$$ state in the nuclear matter or a larger density of comoving medium [436]. This comparison suggests that an additional CNM final-state effect significantly affects $$\mathrm {J}/\psi $$ production at backward and mid-rapidity at $$\sqrt{s_{\mathrm{NN}}}$$$$=$$ 200 $$~\text {GeV}$$. One should, however, emphasise that the comparison of the open heavy-flavour and $$\mathrm {J}/\psi $$ production is carried out as a function of $$p_{\mathrm {T}}$$. The c quark fragments into a charm mesons which in turn decays into a lepton and it is not straightforward to relate the decay lepton momentum to the parent quark momentum in order to interpret accurately this comparison.

**Fig. 44:**
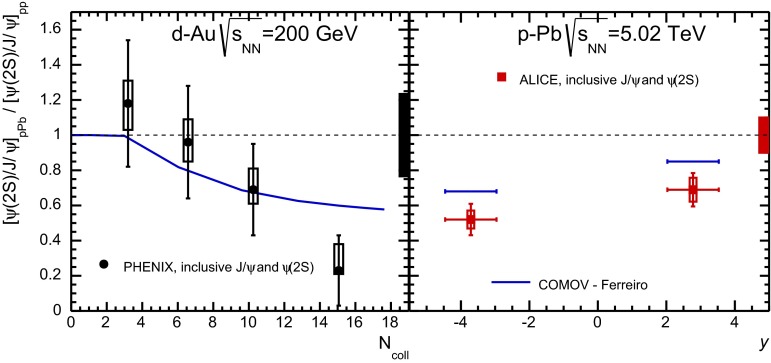
*Left* relative nuclear modification ($[ \psi \text {(2S)}/\mathrm {J}/\psi ]_\mathrm{dAu} / [ \psi \text {(2S)}/\mathrm {J}/\psi ]_\mathrm{pp}$) of inclusive $\mathrm {J}/\psi$ to $\psi \text {(2S)}$ as a function of $\mathrm {N_{coll}}$ in PHENIX [320]. *Right* transverse momentum dependence of the relative nuclear modification of $\mathrm {J}/\psi$ to $\psi (2S)$ in ALICE [326]. The uncertainties are the same as described in Fig. 39

**Fig. 45:**
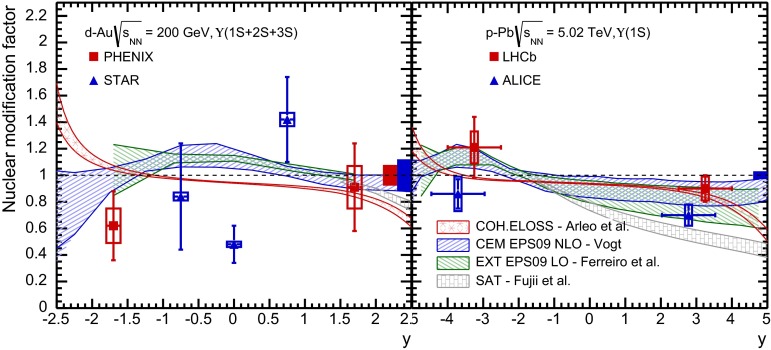
*Left* rapidity dependence of $\Upsilon \text {(1S+2S+3S)}$ in PHENIX [321] and STAR [323]. *Right* rapidity dependence of $R_{\mathrm {pPb}}$ for $\Upsilon \text {(1S)}$ in ALICE [328] and LHCb [331]

The binding energy of the excited charmonium states is significantly smaller than that of the ground state [437]: the $\psi \text {(2S)}$ has the lowest binding energy (0.05 GeV), following by the $\chi _c$ (0.20 GeV) and the $\mathrm {J}/\psi$ (0.64 GeV). The excited charmonium states are then expected to be more sensitive to the nuclear environment as compared to the $\mathrm {J}/\psi$. The relative suppression of the $\psi \text {(2S)}$ to $\mathrm {J}/\psi$ from earlier measurements at lower energy and at mid-rapidity [254, 341, 352] has been understood as a larger absorption of the $\psi \text {(2S)}$ in the nucleus since, under these conditions, the crossing time $\tau _\mathrm{cross}$ of the ${c\overline{c}}$ pair through the nucleus is larger than the charmonium formation time $\tau _\mathrm{f}$. At higher energy, $\tau _\mathrm{cross}$ is expected to be always lower than $\tau _\mathrm{f}$  [438] except maybe for backward-rapidity ranges. This means that the ${c\overline{c}}$ is nearly always in a pre-resonant state when traversing the nuclear matter and the nuclear break-up should be the same for the $\psi \text {(2S)}$ and $\mathrm {J}/\psi$.

The PHENIX experiment has measured $R_{\mathrm {dAu}} =0.54\pm 0.11\mathrm { (stat)}^{+0.19}_{-0.16}\mathrm { (syst)}$ for the $\psi \text {(2S)}$ and $R_{\mathrm {dAu}} =0.77\pm 0.41\mathrm { (stat)}\pm 0.18\mathrm { (syst)}$ for the $\chi _c$ for $|y|<0.35$ [320]. While the large uncertainty prevents any conclusion for the $\chi _c$, the relative modification factor of the $\psi \text {(2S)}$ to inclusive $\mathrm {J}/\psi$ in d–Au collisions, $[ \psi \text {(2S)}/\mathrm {J}/\psi ]_\mathrm{dAu} / [ \psi \text {(2S)}/\mathrm {J}/\psi ]_\mathrm{pp}$ equivalent to $R_{\mathrm {dAu}} ^{\psi \text {(2S)}}/R_{\mathrm {dAu}} ^{\mathrm {J}/\psi }$, has been found to be $0.68\pm 0.14\mathrm { (stat)}^{+0.21}_{-0.18}\mathrm { (syst)}$, i.e. 1.3 $\sigma$ lower than 1. The relative modification factor as a function of $\mathrm {N_{coll}}$ is shown in the left panel of Fig. 44. In the most central collisions, the $\psi \text {(2S)}$ is more suppressed than the $\mathrm {J}/\psi$ by about $2 \,\sigma$.

ALICE has also measured in p–Pb collisions at $$\sqrt{s_{\mathrm{NN}}}$$$$=$$ 5.02 TeV the $$\psi \text {(2S)}$$ to $$\mathrm {J}/\psi $$ relative modification factor and has found $$0.52\pm 0.09\mathrm { (stat)}\pm 0.08\mathrm { (syst)}$$ for $$-4.46<y <-2.96$$ and $$0.69\pm 0.09\mathrm { (stat)}\pm 0.10\mathrm { (syst)}$$ for $$2.03<y <3.53$$ [326], respectively $$4 \,\sigma $$ and $$2 \,\sigma $$ lower than unity. In the right panel of Fig. 44, the relative modification factor is shown as a function of rapidity. This double ratio has also been measured as a function of $$p_{\mathrm {T}}$$  [326] and does not exhibit a significant $$p_{\mathrm {T}}$$ dependence. In addition, preliminary results [439] show that the nuclear modification factor of the $$\psi \text {(2S)}$$ follows a similar trend as the $$\mathrm {J}/\psi $$ as a function of event activity at forward rapidity but is significantly more suppressed at backward rapidity towards central events.

Models based on initial-state effects [363, 438] or coherent energy loss [397] do not predict a relative suppression of the $\psi \text {(2S)}$ production with respect to the $\mathrm {J}/\psi$ one. These measurements could indicate that the $\psi \text {(2S)}$ production is sensitive to final-state effects in p–A collisions. A recent theoretical work uses EPS09 LO nPDF and includes the interactions of the quarkonium states with a comoving medium [436] (COMOV). The COMOV calculations are shown in Fig. 44. They describe fairly well the PHENIX and ALICE results. Hot nuclear matter effects were also proposed as a possible explanation for the $\psi \text {(2S)}$ relative suppression in central p–Pb collisions at the LHC [440].

*Bottomonium measurements* The nuclear modification factor for bottomonium is shown in Fig. 45 at RHIC [321, 323] (left) and LHC [328, 331] (right). At RHIC the 3 $\Upsilon$ states cannot be measured separately due to the poor statistics and invariant-mass resolution. At $\sqrt{s_{\mathrm{NN}}}$  $=$ 200$~\text {GeV}$, the $R_{\mathrm {dAu}}$ is compatible with no or a slight suppression over the full rapidity range except at mid-rapidity where a suppression by a factor of two is found in d–Au with respect to (binary-scaled) $\mathrm pp$ collisions. The data suggests a larger suppression by about $40~\%$ at backward rapidity but the uncertainties are large and $R_{\mathrm {dAu}}$ is lower than unity by only $1.3 \,\sigma$. At $\sqrt{s_{\mathrm{NN}}}$  $=$ 5.02 $\text {TeV}$, the $\Upsilon \text {(1S)}$ measurements from LHCb, despite slightly different rapidity ranges, are systematically higher than those of ALICE but the two measurements are consistent within uncertainties. The measured $R_{\mathrm {pPb}}$ is consistent with unity at backward rapidity and below unity by, at most, $30~\%$ at forward rapidity.

**Fig. 46:**
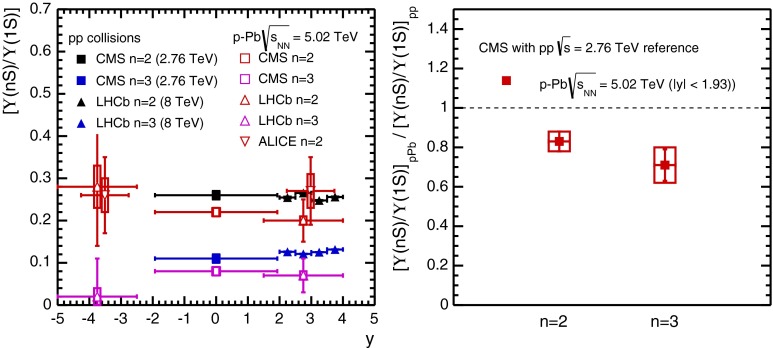
*Left* $\Upsilon \text {(nS)}/\Upsilon \text {(1S)}$ ratio in $\mathrm pp$ and p–Pb collisions in ALICE [328], LHCb [331] and CMS [268]. For a better visibility, the ALICE data points are displaced by $+0.2$ in rapidity. *Right* relative modification factor $[ \Upsilon \text {(nS)}/\Upsilon \text {(1S)} ]_\mathrm{pPb} / [ \Upsilon \text {(nS)}/\Upsilon \text {(1S)} ]_\mathrm{pp}$ in CMS [268]

The data are compared to models based on nPDFs (CEM EPS09 NLO, EXT EPS09 LO), coherent energy loss (COH.ELOSS) and gluon saturation (SAT). Given the limited statistics, the data cannot constrain the models in most of the phase space and is in good agreement with the theory calculations. Only at mid-rapidity at $\sqrt{s_{\mathrm{NN}}}$  $=$ 200$~\text {GeV}$, the observed suppression is challenging for all the models, where no suppression is expected. In the nPDF based model, the rapidity range where $R_{\mathrm {pA}}$ is higher than unity corresponds to the anti-shadowing region. Clearly the data is not precise enough to conclude on the strength of gluon anti-shadowing.

As in the $\mathrm {J}/\psi$ case, the ratio $R_{\mathrm {FB}}$ of the nuclear modification factors for a rapidity range symmetric with respect to $y \sim 0$ has also been extracted for the $\Upsilon \text {(1S)}$ at $\sqrt{s_{\mathrm{NN}}}$  $=$ 5.02 $\text {TeV}$  [328, 331].

Comparison of $$\Upsilon \text {(1S)}$$$$R_{\mathrm {pPb}}$$ to the one from open beauty from Fig. 37 can give a hint on final-state effects on $$\Upsilon \text {(1S)}$$. A similar level of suppression is observed for the $$\Upsilon \text {(1S)}$$ and the $$\mathrm {J}/\psi $$ from B mesons. Larger statistics data however would be needed to rule out any final-state effect on $$\Upsilon \text {(1S)}$$ production in p–Pb.

The study of excited bottomonium states in p–Pb collisions may indicate the presence of final-state effects in bottomonium production. The $\Upsilon \text {(3S)}$ has the smallest binding energy (0.2$~\text {GeV}$), followed by the $\Upsilon \text {(2S)}$ (0.54 $~\text {GeV}$) and the $\Upsilon \text {(1S)}$ (1.10$~\text {GeV}$) [437]. Since the bottomonium formation time is expected to be larger than the nuclear size, the suppression in p–Pb is expected to be the same for all $\Upsilon$ states.

The CMS experiment has measured the ratio of the excited to the ground-state cross section, $$\Upsilon \text {(nS)}/\Upsilon \text {(1S)} $$, for $$n=2,3$$ at mid-rapidity in p–Pb collisions. ALICE (only for $$n=2$$) and LHCb have performed similar measurements at backward and forward rapidity. The measured ratios $$\Upsilon \text {(nS)}/\Upsilon \text {(1S)} $$, shown in the left panel of Fig. 46 are compared to the ratios measured in $$\mathrm pp$$ collisions at, however, different energies ($$\sqrt{s}$$  $$=$$ 2.76 and 8 $$\text {TeV}$$) and in addition for the backward and forward rapidities, in slightly different rapidity ranges. It is worth noting that the ratio $$\Upsilon \text {(nS)}/\Upsilon \text {(1S)} $$ has been measured for $$n=2,3$$ at $$\sqrt{s}$$ = 1.8, 2.76 and 7 $$\text {TeV}$$  [198, 268, 441] at mid-rapidity and at $$\sqrt{s}$$ = 2.76, 7 and 8 $$\text {TeV}$$  [206, 208, 419] at forward rapidity. The ratio is found to be, within the quoted uncertainties, independent of $$\sqrt{s}$$, and in the rapidity range $$2<y <4$$, independent of $$y $$. A stronger suppression than in $$\mathrm pp$$ is observed at mid-rapidity in p–Pb collisions for $$\Upsilon \text {(2S)}$$ and $$\Upsilon \text {(3S)}$$ as compared to $$\Upsilon \text {(1S)}$$, which could suggest the presence of final-state effects that affect more the excited states as compared to the ground state. At forward rapidity, the ratios measured by ALICE and LHCb are similar in p–Pb and $$\mathrm pp$$ but the measurements are not precise enough to be sensitive to a difference as observed by CMS.

**Fig. 47:**
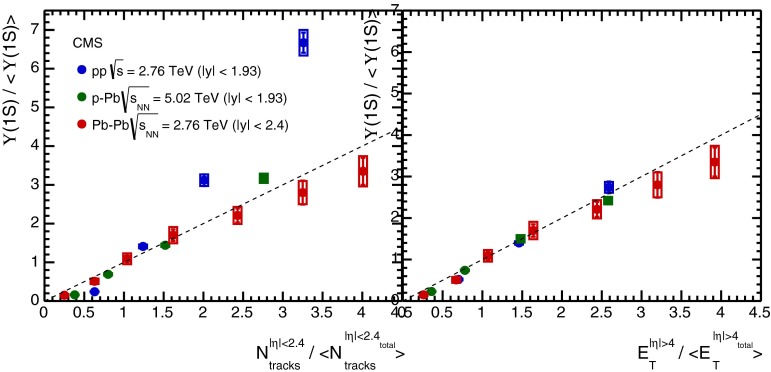
Self-normalised cross section ratio $\frac{\Upsilon \text {(1S)}}{\langle \Upsilon \text {(1S)} \rangle }$ vs. $\frac{N_\mathrm{tracklets}}{\langle N_{\mathrm{tracklets}} \rangle }$ (*left*) and $\frac{E_{\mathrm{T}}}{\langle E_{\mathrm{T}} \rangle }$ (*right*) in $\mathrm pp$ collisions at 2.76 $\text {TeV}$, p–Pb collisions at 5.02 $\text {TeV}$ and Pb–Pb collisions at 2.76 $\text {TeV}$ in CMS [268]. Here $N_\mathrm{tracklets}$ is the charged-track multiplicity measured in $|\eta |<2.4$ and $E_\mathrm{T}$ the transverse energy measured in $4<|\eta |<5.2$. The *dotted line* is a linear function with a slope equal to unity

**Fig. 48:**
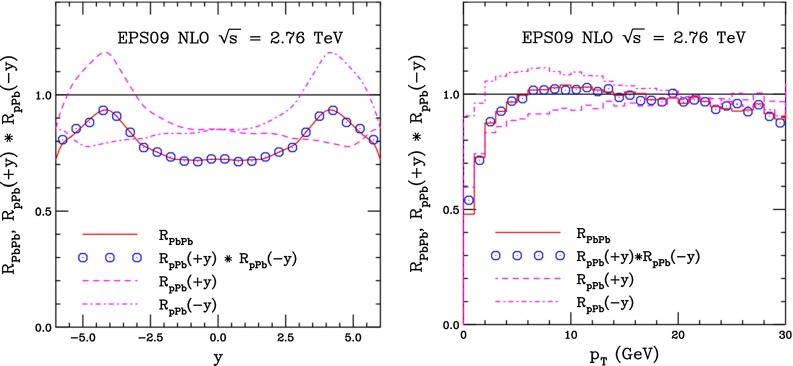
The $\mathrm {J}/\psi$ $R_{\mathrm {AA}}$ (*red*) ratio is compared to the product $R_{\mathrm {pA}} (+y) \cdot R_{\mathrm {pA}} (-y)$ (*points*) along with the individual p–A ratios at forward (*dashed*) and backward (*dot-dashed*) rapidity. Results are compared for the *y* (*left*) and $p_{\mathrm {T}}$ (*right*) dependencies at NLO, from Ref. [442]

To better quantify the modification between $\mathrm pp$ and p–Pb and cancel out some of the systematic uncertainties from the detector set-up, the double ratio $[ \Upsilon \text {(nS)}/\Upsilon \text {(1S)} ]_\mathrm{pPb} / [ \Upsilon \text {(nS)}/\Upsilon \text {(1S)} ]_\mathrm{pp}$ has also been evaluated by CMS at mid-rapidity using $\mathrm pp$ collisions at 2.76 $\text {TeV}$ [268] and is displayed in the right panel of Fig. 46. The double ratio in p–Pb is lower than one by $2.4\,\sigma$ for $\Upsilon \text {(2S)}$ and $\Upsilon \text {(3S)}$. The double ratios signal the presence of different or stronger final-state effect acting on the excited states compared to the ground state from $\mathrm pp$ to p–Pb collisions.

As for the charmonium production, the excited states are not expected to be differentially suppressed by any of the models that include initial-state effects nor from the coherent energy-loss effect. A possible explanation may come from a suppression associated to the comoving medium. Precise measurements in a larger rapidity range, which covers different comoving medium density, would help to confirm this hypothesis.

CMS has also performed measurements as a function of the event activity at forward ($4<|\eta |<5.2$ for the transverse energy $E_\mathrm{T}$) and mid-rapidity ($|\eta |<2.4$ for the charged-track multiplicity $N_\mathrm{tracklets}$) [268]. Figure 47 shows the $\Upsilon$ self-normalised cross section ratios $\Upsilon \text {(1S)}$/$\langle \Upsilon \text {(1S)} \rangle$ where $\langle \Upsilon \text {(1S)} \rangle$ is the event-activity integrated value for $\mathrm pp$, p–Pb and Pb–Pb collisions. The self-normalised cross section ratios are found to rise with the event activity as measured by these two estimators and similar results are obtained for $\Upsilon \text {(2S)}$ and $\Upsilon \text {(3S)}$. When Pb ions are involved, the increase can be related to the increase in the number of nucleon–nucleon collisions. A possible interpretation of the positive correlation between the $\Upsilon$ production yield and the underlying activity of the $\mathrm pp$ event is related to Multiple-Parton Interactions (MPI) occurring in a single $\mathrm pp$ collisions. Linear fits performed separately for the three collision systems show that the self-normalised ratios have a slope consistent with unity in the case of forward event activity. Hence, no significant difference between $\mathrm pp$, p–Pb and Pb–Pb is observed when correlating $\Upsilon$ production yields with forward event activity. On the contrary in the case of mid-rapidity event activity, different slopes are found for the three collisions systems. These observations are also related to the single cross section ratios $\Upsilon \text {(nS)}/\Upsilon \text {(1S)}$ as shown in Fig. 19 and discussed in detail in Sect. 2.4.1.

### Extrapolation of CNM effects from p–A to AA collisions

It is an important question to know whether cold nuclear matter effects can be simply extrapolated from p–A to AA collisions. Some of the CNM effects discussed in Sect. 3.2 can in principle be extrapolated to AA collisions. This is the case of nPDF and coherent energy-loss effects, discussed below. Some other approaches, on the contrary, are affected by interference effects between the two nuclei involved in the collision, making delicate an extrapolation to AA collisions.

*Nuclear PDF* Regarding the nPDF effects discussed in Sect. 3.2.2, it is straightforward to make this comparison at leading order in the colour evaporation model (CEM) where the $p_{\mathrm {T}}$ of the ${Q\overline{Q}}$ pair is zero and the $x_1$ and $x_2$ values are related to the quarkonium rapidity by Eq. (17). As long as the production cross section obeys the factorisation hypothesis, Eq. (16), the nuclear modification factors (taken at the same energy) also factorise, i.e. the following relation is exact:

26 $$\begin{aligned} R_{\mathrm {AA}} ^\mathrm{CNM}(y) = R_{\mathrm {pA}} (+y) \cdot R_{\mathrm {pA}} (-y). \end{aligned}$$

At next-to-leading order in the CEM, however, the assumption of factorisation of nPDF effects is less simple to understand because of the large contribution from $2 \rightarrow 2$ diagrams. For such processes, the correlation between the initial momentum fractions $x_1, x_2$ and the rapidity of the quarkonium state is less straightforward. However, the factorisation hypothesis (see Eq. (26)) is seen to still hold at NLO, as shown by a calculation using EPS09 NLO central nPDF set at $\sqrt{s_{\mathrm{NN}}} = 2.76$ TeV in Fig. 48 as a function of *y* (left) and $p_{\mathrm {T}}$ (right) [442].

**Fig. 49:**
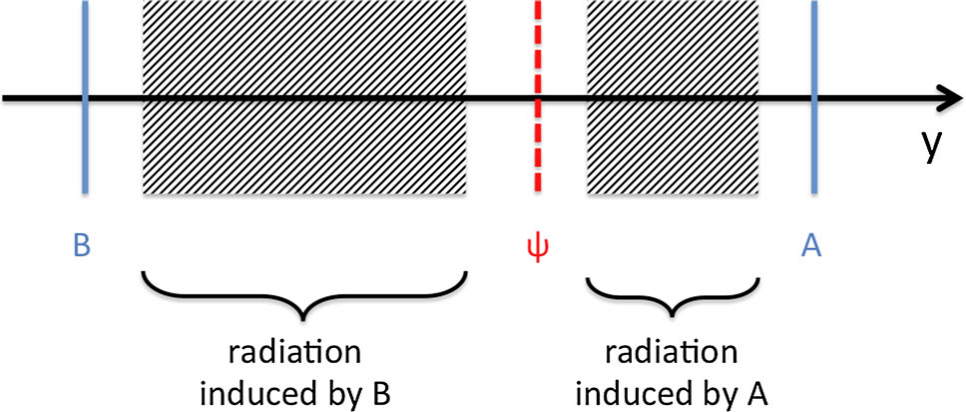
Sketch of the rapidity regions populated by medium-induced radiation in an A–B collision in the coherent energy loss model. The ‘target’ B and ‘projectile’ A move with, respectively, negative and positive rapidities

In principle, this factorisation hypothesis can also be applied to open heavy flavour.

*Multiple scattering and energy loss* Let us first discuss how predictions can be extrapolated in the coherent energy-loss model. In a generic A–B collision both incoming partons, respectively from the ‘projectile’ nucleus A and the ‘target’ nucleus B, might suffer multiple scattering in the nucleus B and A, respectively. Consequently, gluon radiation off both partons can interfere with that of the final-state particle (here, the compact colour octet ${Q\overline{Q}}$ pair), making a priori difficult the calculation of the medium-induced gluon spectrum in the collision of two heavy ions.

However, it was shown in [404] that the gluon radiation induced by rescattering in nuclei A and B occurs in distinct regions of phase space (see Fig. 49). As a consequence, the energy loss induced by the presence of each nucleus can be combined in a probabilistic manner, making a rather straightforward extrapolation of the model predictions from p–A to AA collisions. Remarkably, it is possible to show that the quarkonium suppression in AA collisions follows the factorisation hypothesis (see Eq. (26)). However, since the energy-loss effects do not scale with the momentum fraction $x_2$, the data-driven extrapolation of p–Pb data at $\sqrt{s_{\mathrm{NN}}} =5$ TeV to Pb–Pb data at $\sqrt{s_{\mathrm{NN}}} =2.76$ TeV, discussed below, and which assumes nPDF effects only is not expected to hold [404].

The model by Sharma and Vitev can also easily be generalised to AA reactions where both incoming and outgoing partons undergo elastic, inelastic and coherent soft interactions in the large nuclei. In contrast, the Kopeliovich, Potashnikova and Schmidt approach for charmonium production cannot be simply extrapolated from p–A to AA collisions, because nucleus–nucleus collisions include new effects of double colour filtering and a boosted saturation scale, as explained in detail in [385].

*Data-driven extrapolation* At RHIC, the d–Au collisions are performed with symmetrical beam energies, so that $$y_\mathrm{CM/lab} = 0$$, and at the same nucleon–nucleon centre-of-mass energy than for heavy-ion collisions. The direct comparison of d–Au data to heavy-ion data is then easier. In this context, the PHENIX experiment has evaluated the $$\mathrm {J}/\psi $$ break-up (i.e. absorption) cross section by fitting $$R_{\mathrm {dAu}}$$ as a function of the rapidity, and also as a function of the average number of binary collisions ($$\mathrm {N_{coll}}$$), and by assuming different shadowing scenarios (EKS and NDSG) [317]. The two shadowing scenarios with their resulting break-up cross section were applied to $$\mathrm {J}/\psi $$$$R_{\mathrm {AA}}$$, both for Cu–Cu and Au–Au collisions. Moreover, an alternative data-driven method [443] was applied to PHENIX data [317]. This method assumes that all cold nuclear matter effects are parametrised with a modification factor consisting of a function of the radial position in the nucleus. Note that the use of d–Au data in [443] may not be appropriate for peripheral collisions where the size of the deuteron causes significant averaging over impact parameter; on the contrary it should be adequated in central collisions for which the averaging is not so important. An attempt to solve this issue has been proposed in [64] where an estimate of $$R_{\mathrm {pAu}}$$ was derived from $$R_{\mathrm {dAu}} $$ using a Glauber model including EKS98 nuclear PDF.

A more recent investigation of RHIC data by Ferreiro et al. [374] showed how the use of $2 \rightarrow 2$ partonic process instead of the usual $2 \rightarrow 1$ can imply a different value of the absorption cross section [444], since the anti-shadowing peak is systematically shifted towards larger rapidities in d–Au. The other noticeable consequence is that $R_{\mathrm {dAu}}$ versus *y* is not symmetric anymore around $y \approx 0$. This implies that the CNM effects in $R_{\mathrm {AA}}$ at RHIC will also show a rapidity dependence, with less suppression from CNM effects at mid-rapidity than at forward rapidity, in the same direction as the one exhibited by the Au–Au and Cu–Cu data from PHENIX (see extensive comparisons in [374]). This is quite important since this shape of $R_{\mathrm {AA}}$ at RHIC was also considered as a possible hint for hot in-medium recombination effects, while it might come from CNM effects only.

At LHC, the p–Pb results cannot easily be compared to Pb–Pb collisions. Indeed, the nucleon–nucleon centre-of-mass energies are not the same (5.02 versus 2.76 TeV) and, moreover, the p and the Pb beam energies per nucleon are different, leading to a rapidity shift of the centre-of-mass frame with respect to the lab frame. But assuming factorisation and Eq. (26), a data-driven extrapolation of p–A data to AA can be performed.

In a given detector acceptance (at fixed $y_\mathrm{lab}$), the ratio of $x_2$ values probed in a given process in Pb–Pb and p–Pb collisions is

27 $$\begin{aligned} \frac{x_2^\mathrm{PbPb}}{x_2^\mathrm{pPb}} = \frac{\sqrt{s_\mathrm{NN}^\mathrm{pPb}}}{\sqrt{s_\mathrm{NN}^\mathrm{PbPb}}} \exp (-y_\mathrm{CM/lab}^\mathrm{pPb}). \end{aligned}$$

At the LHC, the rapidity shift is $y_\mathrm{CM/lab}^\mathrm{pPb} = 0.465$. In Run 1 conditions, one has $\sqrt{s_\mathrm{NN}^\mathrm{PbPb}} = 2.76$ TeV and $\sqrt{s_\mathrm{NN}^\mathrm{pPb}} = 5.02$ TeV and the ratio is $\frac{x_2^\mathrm{PbPb}}{x_2^\mathrm{pPb}} = 8\ \mathrm{TeV} / 7\ \mathrm{TeV}\simeq 1.14$. The typical momentum fraction ranges involved in p–Pb collisions are shown in Fig. 25.

This data-driven extrapolation of p–A collisions to AA collisions applied by the ALICE Collaboration to $\mathrm {J}/\psi$ production lead to [325]: $[R_{\mathrm {pPb}} (2<y<3.5) \cdot R_{\mathrm {pPb}} (-4.5<y<-3)]^{\mathrm {J}/\psi } = 0.75 \pm 0.10 \pm 0.12$, the first uncertainty being the quadratic combination of statistical and uncorrelated systematic uncertainties and the second one the linear combination of correlated uncertainties. The application of this result to the interpretation of Pb–Pb data is discussed in Sect. 5.3.

In summary, according to the theoretical and data-driven extrapolation approaches, one can conclude that there are non-negligible CNM effects on AA results at the LHC (up to 50 % at low $$p_{\mathrm {T}}$$). A $$p_{\mathrm {T}}$$ dependence of $$\mathrm {J}/\psi $$$$R_{\mathrm {pPb}}$$ factorisation will be presented in Sect. 5.1.2.

### Summary and outlook

The LHC p–Pb Run 1 has opened a new window on the study of the CNM effects. The broad kinematical range probed by the different LHC experiments and the comparison to RHIC d–Au results bring new constraints on the theoretical models. The main observations resulting from the open and hidden heavy-flavour data can be summarised in the following way:

- The nuclear modification factor of open heavy-flavour decay leptons in d–Au collisions at RHIC shows a dependence on centrality and on rapidity, with values smaller than unity at forward rapidity and larger than unity at mid- and backward rapidity in the most central collisions.
- In p–Pb collisions at the LHC, the D-meson nuclear modification factor at mid-rapidity and $$1<p_{\mathrm {T}} <16$$$$~\text {GeV}/c$$ is consistent with unity within uncertainties of about 20 %.
- The $R_{\mathrm {pA}}$ of $\mathrm {J}/\psi$ from B mesons at the LHC shows a modest suppression at forward rapidity and is consistent with unity at backward rapidity.
- A rapidity dependence of $\mathrm {J}/\psi$ suppression has been measured at RHIC and LHC. At both energies the suppression is more pronounced at forward than at mid-rapidity. At backward rapidity, $\mathrm {J}/\psi$ production is slightly suppressed at RHIC but is compatible with no suppression at the LHC.
- There is no evidence of $\mathrm {J}/\psi$ suppression at large $p_{\mathrm {T}}$ in the full rapidity range at RHIC and LHC.
- At RHIC, open heavy flavour from lepton decay and $\mathrm {J}/\psi$ suppression for $p_{\mathrm {T}} > 1~\text {GeV}/c$ are of the same order at forward rapidity but not at backward and mid-rapidity: suppression mechanisms from final-state effects may be at play on $\mathrm {J}/\psi$ production at backward and mid-rapidity.
- $$\Upsilon \text {(1S)}$$$$R_{\mathrm {pA}}$$ measurements are compatible with unity except at mid-rapidity at RHIC and forward rapidity at the LHC. Similar level of suppression is observed for the $$\Upsilon \text {(1S)}$$ and the $$\mathrm {J}/\psi $$ from B-mesons at the LHC. However, the $$\Upsilon \text {(1S)}$$$$R_{\mathrm {pA}}$$ measurements have large statistical uncertainties.
- Excited states are more suppressed than 1S states at RHIC and LHC suggesting the presence of final-state CNM effects.

For the theoretical interpretation of the data, the following conclusions can be drawn:

- Open heavy-flavour current data do not allow one to favour specific models based on nuclear PDF, parton saturation, or initial-parton energy loss.
- Regarding $\mathrm {J}/\psi$ production, one can conclude the following:
  - The nuclear PDFs describe well the $R_{\mathrm {pA}}$ despite large theoretical uncertainties at forward rapidity where the data would require strong shadowing effects. At backward rapidity, while the nPDF models describe correctly the LHC data, they do not describe the RHIC data without considering additional effects such as nuclear absorption.
  - The early CGC prediction of $$\mathrm {J}/\psi $$$$R_{\mathrm {pA}}$$ by Fuji and Watanabe is ruled out by the present LHC data at forward rapidity. The calculations do not describe either the $$p_{\mathrm {T}}$$ dependence of the RHIC data at forward rapidity. Refinements of the model have now been proposed, leading to less disagreement with data.
  - The predictions of the coherent energy-loss model describes well the rapidity dependence of $$\mathrm {J}/\psi $$$$R_{\mathrm {pA}}$$ both at RHIC and at LHC. Regarding the $$p_{\mathrm {T}} $$ dependence, the shape of the data is also rather well captured, although the dependence is slightly more abrupt in the model than in the data, especially at forward rapidity. The predicted $$\mathrm {J}/\psi $$ suppression expected in the dipole propagation model by Kopeliovich, Potashnikova and Schmidt seems much larger than seen in data, suggesting the need for additional effects to compensate the suppression. Finally, the approach based on energy loss and power corrections by Sharma and Vitev predicts a moderate and flat $$\mathrm {J}/\psi $$ and $$\Upsilon $$ suppression as a function of $$p_{\mathrm {T}}$$, above $$p_{\mathrm {T}} =4$$ GeV/*c*, somehow in contradiction with data.
- The suppression of excited states relative to 1S state is described so far only by considering the effect from a comoving medium.

The main limitations for the interpretation of the current experimental results are, on the one hand, the sizeable experimental uncertainties, on the other hand, the large uncertainties on the nuclear modification of the PDFs in the low-*x* region.

Regarding the experimental uncertainties, in the case of rare probes like B mesons, $\psi \text {(2S)}$ and $\Upsilon$, but also high $p_{\mathrm {T}}$ yields, the experimental data suffer from limited statistics. For more abundant probes, like heavy-flavour decay leptons. D mesons, B from $\mathrm {J}/\psi$ and prompt $\mathrm {J}/\psi$, the size of the systematic uncertainties is the main limitation.

To address part of these issues, a reference $\mathrm pp$ period at $\sqrt{s} =5.02$ $\text {TeV}$ and a higher-statistics p–Pb period at $\sqrt{s} =5.02$ $\text {TeV}$, which will allow a better control of the systematics, during the LHC Run 2 would be very helpful to improve the precision of the current measurements. However, for the probes which are using the full LHC luminosity and have already a $\mathrm pp$ reference at 8 $\text {TeV}$ from the 2012 Run 1 data taking period, it would be more interesting to get a new p–Pb run at $\sqrt{s_{\mathrm{NN}}} =8$ $\text {TeV}$ in order to study the CNM effects at higher energy. These aspects have to be balanced in order to choose the energy for the p–Pb run in Run 2.

New observables could help to disentangle the various CNM effects. First studies of the heavy-flavour azimuthal correlations at RHIC and LHC were carried out and (at RHIC) suggest a modification of charm production kinematics in d–Au. A comparison of open to hidden heavy flavour production from $p_{\mathrm {T}} =0$ would allow one to separate initial- from final-state effects on quarkonia. Another open question is related to quarkonium polarisation: can the CNM effects modify the polarisation of quarkonia? In addition, the RHIC capability to collide a polarised-proton beam with nuclei can be used to explore new observables.

Finally, it is not clear whether the CNM effects can be extrapolated from p–A to AA collisions. At present, only phenomenological works based on nPDF and coherent energy-loss effects have shown that this extrapolation is possible, although with some caveats. On the one hand, in the nPDF-based models, the main parameter is the probed momentum fraction *x*. Since there is a rapidity shift of the centre-of-mass in p–Pb collisions at LHC, the optimal strategy would be to choose the LHC beam energy according to Eq. (27). On the other hand, in the coherent energy-loss model, the relevant parameter is the $\sqrt{s_{\mathrm{NN}}}$ value and p–A collisions can be directly related to AA collisions only if taken at the same energy.

## Open heavy flavour in nucleus–nucleus collisions

Heavy-flavour hadrons are effective probes of the conditions of the high-energy-density QGP medium formed in ultra-relativistic nucleus–nucleus collisions.

Heavy quarks, are produced in primary hard QCD scattering processes in the early stage of the nucleus–nucleus collision and the time scale of their production (or coherence time) is, generally, shorter than the formation time of the QGP, $\tau _0\sim 0.1$–1 fm / *c*. More in detail, the coherence time of the heavy quark-antiquark pair is of the order of the inverse of the virtuality *Q* of the hard scattering, $\Delta \tau \sim 1/Q$. The minimum virtuality $2\,m_{c,b}$ in the production of a ${c\overline{c}}$ or ${b\overline{b}}$ pair implies a space-time scale of $\sim 1/3~\text {GeV} ^{-1}\sim 0.07~\mathrm{fm}$ and $\sim 1/10~\text {GeV} ^{-1}\sim 0.02~\mathrm{fm}$ for charm and for beauty, respectively. One exception to this picture are configurations where the quark and antiquark are produced with a small relative opening angle in the so-called gluon splitting processes $g\rightarrow {q\overline{q}}$. In this case, the coherence time is increased by a boost factor $E_g/(2\,m_{c,b})\sim E_{c,b}/m_{c,b}$ and becomes $\Delta t \sim E_{c,b}/(2\,m_{c,b}^2)$. This results, for example, in a coherence time of about 1 fm/*c* (0.1 fm/*c*) for charm (beauty) quarks with energy of 15$~\text {GeV}$, and of about 1 fm/*c* for beauty quark jets with energy of about 150$~\text {GeV}$. The fraction of heavy quarks produced in gluon splitting processes has been estimated using perturbative calculations and Monte Carlo event generators, resulting in moderate values of the order of 10–20 % for charm [445, 446] and large values of the order of 50 % for beauty [447]. Given that the gluon splitting fraction is moderate for charm and the coherence time is small for beauty from gluon splitting when $p_{\mathrm {T}}$ is smaller than about 50$~\text {GeV}/c$, it is reasonable to conclude that heavy-flavour hadrons in this $p_{\mathrm {T}}$ range probe the heavy quark in-medium interactions.

During their propagation through the medium, heavy quarks interact with its constituents and lose a part of their energy, thus being sensitive to the medium properties. Various approaches have been developed to describe the interaction of the heavy quarks with the surrounding plasma. In a perturbative treatment, QCD energy loss is expected to occur via both inelastic (radiative energy loss, via medium-induced gluon radiation) [448, 449] and elastic (collisional energy loss) [450–452] processes. However, this distinction is no longer meaningful in strongly coupled approaches relying for instance on the AdS/CFT conjecture [453, 454]. In QCD, quarks have a smaller colour coupling factor with respect to gluons, so that the energy loss for quarks is expected to be smaller than for gluons. In addition, the “dead-cone effect” should reduce small-angle gluon radiation for heavy quarks with moderate energy-over-mass values, thus further attenuating the effect of the medium. This idea was first introduced in [455]. Further theoretical studies confirmed the reduction of the total induced gluon radiation [456–459], although they did not support the expectation of a “dead cone”. Other mechanisms such as in-medium hadron formation and dissociation [422, 460], would determine a stronger suppression effect on heavy-flavour hadrons than light-flavour hadrons, because of their smaller formationtimes.

In contrast to light quarks and gluons, which can be produced or annihilated during the entire evolution of the medium, heavy quarks are produced in initial hard-scattering processes and their annihilation rate is small [461]. Secondary “thermal” charm production from processes like $gg\rightarrow {c\overline{c}}$ in the QGP is expected to be negligible, unless initial QGP temperatures much larger than that accessible at RHIC and LHC are assumed [462]. Therefore, heavy quarks preserve their flavour and mass identity while traversing the medium and can be tagged throughout all momentum ranges, from low to high $p_{\mathrm {T}}$, through the measurement of heavy-flavour hadrons in the final state of thecollision.

The nuclear modification factor

28 $$\begin{aligned} R_{\mathrm {AA}} (p_{\mathrm {T}})= \frac{1}{\left\langle T_{\mathrm {AA}} \right\rangle } \cdot \frac{\mathrm {d}N_\mathrm{AA}/\mathrm {d}p_{\mathrm {T}}}{\mathrm {d}\sigma _\mathrm{pp}/\mathrm {d}p_{\mathrm {T}}}, \end{aligned}$$

of which a detailed definition is given in Sect. 3, is well established as a sensitive observable for the study of the interaction of hard partons with the medium. At large $p_{\mathrm {T}}$, $R_{\mathrm {AA}}$ is expected to be mostly sensitive to the average energy loss of heavy quarks in the hot medium. The study of more differential observables can provide important insights into the relevance of the various interaction mechanisms and the properties of the medium. In particular, the dependence of the partonic energy loss on the in-medium path length is expected to be different for each mechanism (linear for collisional processes [450–452] and close to quadratic for radiative processes in a plasma [449]). Moreover, it is still unclear if low-momentum heavy quarks can reach thermal equilibrium with the medium constituents and participate in the collective expansion of the system [463, 464]. It was also suggested that low-momentum heavy quarks could hadronise not only via fragmentation in the vacuum, but also via the mechanism of recombination with other quarks from the medium [464, 465].

**Table 9:** Open heavy flavour published measurements in Au–Au and Cu–Cu collisions at RHIC. The nucleon–nucleon energy in the centre-of-mass system ($\sqrt{s_{\mathrm{NN}}}$), the covered kinematic ranges and the observables are indicated

Probe	Colliding system	$\sqrt{s_{\mathrm{NN}}}$ ($\text {TeV}$)	$y_\mathrm{cms}$ (or $\eta _\mathrm{cms}$)	$p_{\mathrm {T}}$ ($\text {GeV}/c$)	Observables	References
PHENIX						
$\mathrm{HF} \rightarrow e^{\pm }$	Au–Au	62.4	$|y|<0.35$	1–5	Yields ($p_{\mathrm {T}}$,centrality)	[467]
					$R_{\mathrm {CP}}$($p_{\mathrm {T}}$)	
					$R_{\mathrm {AA}}$($p_{\mathrm {T}}$,centrality)	
					$R_{\mathrm {AA}}$($\mathrm {N_{coll}}$,$p_{\mathrm {T}}$)	
				1.3–3.5	$$v_{2} $$($$p_{\mathrm {T}}$$,centrality)	
				1.3–2.5	$$v_{2} $$($$\sqrt{s_{\mathrm{NN}}}$$,centrality)	
		130	$|y|<0.35$	0.4–3	Yields ($p_{\mathrm {T}}$,centrality)	[468]
		200	$|\eta |<0.35$	0.3–9	Yields ($p_{\mathrm {T}}$,centrality)	[469–472]
					$R_{\mathrm {AA}}$($p_{\mathrm {T}}$,centrality)	
					$R_{\mathrm {AA}}$($\mathrm {N_{part}}$,$p_{\mathrm {T}}$)	
				$>0.4$	$\frac{\mathrm {d}\sigma _{NN}}{\mathrm {d}y}$($\mathrm {N_{coll}}$)	
				$>0$	$\frac{\mathrm {d}\sigma _{NN}}{\mathrm {d}y}$(centrality)	
					$$\sigma _{NN}^{{c\overline{c}}}$$(centrality)	
				0.3–5	$$v_{2} $$($$p_{\mathrm {T}}$$,centrality)	
		200	$|y|<0.35$	2–4	$\frac{1}{N_{\mathrm {trig}}^{e_{HF}}}\frac{\mathrm {d}N_{\mathrm {assoc.}}^{\mathrm {h}}}{\mathrm {d}p_{\mathrm {T}}}(p_{\mathrm {T}} ^{\mathrm {h}},\Delta \phi )$	[113]
					$$I_{\mathrm {AA}} ^{e_{HF}-h}(p_{\mathrm {T}} ^{\mathrm {h}},\Delta \phi )$$	
				2–3	$R_{\mathrm {HS}}(p_{\mathrm {T}} ^{\mathrm {h}})$	
	Cu–Cu	200	$|y|<0.35$	0.5–7	Yields ($p_{\mathrm {T}}$,centrality)	[473]
					$R_{\mathrm {AA}}$($p_{\mathrm {T}}$,centrality)	
					$R_{\mathrm {AA}}$($\mathrm {N_{coll}}$,$p_{\mathrm {T}}$)	
					$R_{\mathrm {AA}}$($\mathrm {N_{part}}$,$p_{\mathrm {T}}$)	
				0.5–6	$R_{\mathrm {CP}}$($p_{\mathrm {T}}$)	
$\mathrm{HF} \rightarrow \mu ^{\pm }$	Cu–Cu	200	$1.4<|y|<1.9$	1–4	Yields ($p_{\mathrm {T}}$,centrality)	[474]
					$R_{\mathrm {AA}}$($p_{\mathrm {T}}$,centrality)	
					$R_{\mathrm {AA}}$($\mathrm {N_{part}}$)	
STAR						
$\mathrm {D}^{0}$	Au–Au	200	$|y|<1$	0–6	Yields ($p_{\mathrm {T}}$,centrality)	[475]
					$R_{\mathrm {AA}}$($p_{\mathrm {T}}$,centrality)	
				0–8	$R_{\mathrm {AA}}$($\langle \mathrm {N_{part}} \rangle$,$p_{\mathrm {T}}$)	
$\mathrm{HF} \rightarrow e^{\pm }$	Au–Au	200	$0<\eta <0.7$	1.2–8.4	Yields ($p_{\mathrm {T}}$,centrality)	[420]
					$R_{\mathrm {AA}}$($p_{\mathrm {T}}$,centrality)	
		39, 62.4, 200	$$|\eta |<0.7$$	0–7	$$v_{2} $$($$p_{\mathrm {T}}$$)	[476]

These questions can be addressed both with the study of the $R_{\mathrm {AA}}$ at low and intermediate $p_{\mathrm {T}}$ (smaller than about five times the heavy-quark mass) and with azimuthal anisotropy measurements of heavy-flavour hadron production with respect to the reaction plane, defined by the beam axis and the impact parameter of the collision. For non-central collisions, the two nuclei overlap in an approximately lenticular region, the short axis of which lies in the reaction plane. Hard partons are produced at an early stage, when the geometrical anisotropy is not yet reduced by the system expansion. Therefore, partons emitted in the direction of the reaction plane (in-plane) have, on average, a shorter in-medium path length than partons emitted orthogonally (out-of-plane), leading a priori to a stronger high-$p_{\mathrm {T}}$ suppression in the latter case. In the low-momentum region, the in-medium interactions can also modify the parton emission directions, thus translating the initial spatial anisotropy into a momentum anisotropy of the final-state particles. Both effects cause a momentum anisotropy that can be characterised with the coefficients $v_n$ and the symmetry planes $\Psi _n$ of the Fourier expansion of the $p_{\mathrm {T}}$-dependent particle distribution $\mathrm {d}^2N/\mathrm {d}p_{\mathrm {T}} \mathrm {d}\phi$ in azimuthal angle $\phi$. The elliptic flow is the second Fourier coefficient$v_{2}$.

The final ambitious goal of the heavy-flavour experimental programmes in nucleus–nucleus collisions is the characterisation of the properties of the produced QCD matter, in particular getting access to the transport coefficients of the QGP. Theoretical calculations encoding the interaction of the heavy quarks with the plasma into a few transport coefficients (see e.g.  [466]) provide the tools to achieve this goal: through a comparison of the experimental data with the numerical outcomes obtained with different choices of the transport coefficients it should be possible, in principle, to put tight constraints on the values of the latter. This would be the analogous of the way of extracting information on the QGP viscosity through the comparison of soft-particle spectra with predictions from fluid dynamic models. An even more intriguing challenge would be to derive the heavy-flavour transport coefficients through a first principle QCD calculation and confront them with experimental data, via model implementations that describe the medium evolution. This chapter reviews the present status of this quest, from the experimental and theoreticalviewpoints.

The chapter is organised as follows. The first part of the chapter presents a brief overview of the available data of heavy-flavour production in nucleus–nucleus collisions at the RHIC and LHC colliders: in particular, Sect. 4.1 describes the measurements of the nuclear modification factor $R_{\mathrm {AA}}$, while Sect. 4.2 focuses on the azimuthal anisotropy. The published RHIC and LHC data are summarised in Tables 9 and 10, respectively. The second part of the chapter includes a review of the theoretical models for heavy-quark interactions and energy loss in the medium, with a detailed description of the model ingredients in terms of the quark–medium interaction (Sect. 4.3) and of the medium modelling (Sect. 4.4). A comparative overview of the models and comprehensive comparison with data from RHIC and LHC are presented in Sect. 4.5. Finally, the theoretical and experimental prospects for the study of heavy-flavour correlations are discussed in Sect. 4.6.

**Table 10:** Open heavy flavour published measurements in Pb–Pb collisions at LHC. The nucleon–nucleon energy in the centre-of-mass system ($\sqrt{s_{\mathrm{NN}}}$), the covered kinematic ranges and the observables are indicated

Probe	Colliding system	$\sqrt{s_{\mathrm{NN}}}$ ($\text {TeV}$)	$y_\mathrm{cms}$ (or $\eta _\mathrm{cms}$)	$p_{\mathrm {T}}$ ($\text {GeV}/c$)	Observables	References
ALICE						
$\mathrm {D}^{0}$, $\mathrm {D}^{+}$, $\mathrm {D}^{*+}$	Pb–Pb	2.76	$|y|<0.5$	2–16	Yields ($p_{\mathrm {T}}$)	[477]
					$R_{\mathrm {AA}}$($p_{\mathrm {T}}$)	
				2–12	$R_{\mathrm {AA}} (\mathrm{centrality})$	
				6–12	$R_{\mathrm {AA}} (\mathrm{centrality})$	
			$$|y|<0.8$$	2–16	$$v_{2} $$($$p_{\mathrm {T}}$$)	[478, 479]
					$$v_{2} $$(centrality,$$p_{\mathrm {T}}$$)	
					$R_{\mathrm {AA}} ^{\text {in/out plane}}$($p_{\mathrm {T}}$)	
$\mathrm{HF} \rightarrow \mu ^{\pm }$	Pb–Pb	2.76	$2.5<y<4$	4–10	$R_{\mathrm {AA}}$($p_{\mathrm {T}}$)	[120]
				6–10	$R_{\mathrm {AA}} (\mathrm{centrality})$	
Non-prompt $\mathrm {J}/\psi$	Pb–Pb	2.76	$|y|<0.8$	1.5–10	$R_{\mathrm {AA}}$ ($p_{\mathrm {T}}$)	[480]
CMS						
*b*-Jets	Pb–Pb	2.76	$|\eta |<2$	80–250	Yields ($p_{\mathrm {T}}$)	[481]
					$R_{\mathrm {AA}}$($p_{\mathrm {T}}$)	
				80–110	$R_{\mathrm {AA}} (\mathrm{centrality})$	
Non-prompt $\mathrm {J}/\psi$	Pb–Pb	2.76	$|y|<2.4$	6.5–30	Yields (centrality)	[482]
					$$R_{\mathrm {AA}} $$(centrality)	

### Experimental overview: production and nuclear modification factor measurements

#### Inclusive measurements with leptons

Heavy-flavour production can be measured inclusively via the semi-leptonic decay channels. The key points of the measurement are the lepton identification and background subtraction.

In the STAR experiment, electrons are identified using the specific energy-loss ($${\mathrm {d}}E/{\mathrm {d}}x$$) measurement from the Time Projection Chamber (TPC) together with the Time of Flight information at $$p_{\mathrm {T}} < 1.5$$$$~\text {GeV}/c$$, and energy and shower shape measurements in the Electro-Magnetic Calorimeter (EMCal) at $$p_{\mathrm {T}} > 1.5$$$$~\text {GeV}/c$$. The background contribution to the electron yield from photonic sources (mainly from photon conversion in the detector material and $$\pi ^0$$ and $$\eta $$ Dalitz decays) are subtracted statistically using the invariant-mass method [115, 116]. The electron identification in the PHENIX experiment is based on the Ring Imaging Cherenkov detector in conjunction with a highly granular EMCal. The subtraction of the electron background is performed by the converter and cocktail methods [112, 471]. In the ALICE experiment, electrons are identified in the central pseudo-rapidity region ($$|\eta |<0.9$$) using four detector systems: the Time Projection Chamber, the Time Of Flight, the EMCal and the Transition Radiation Detector. Background electrons are subtracted using both the invariant-mass and the cocktail methods [117].

**Fig. 50:**
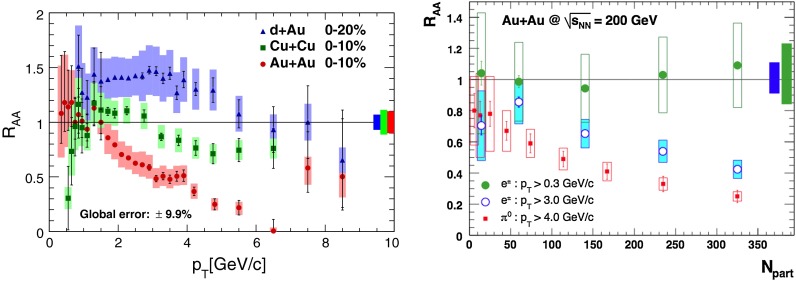
*Left* the transverse momentum dependence of the nuclear modification factors of heavy-flavour decay electrons at mid-rapidity in central d–Au  [313], Cu–Cu  [473] and Au–Au collisions [469] at $\sqrt{s_{\mathrm{NN}}} = 200$ $~\text {GeV}$. *Right* $R_{\mathrm {AA}}$ of heavy-flavour decay electrons at mid-rapidity with $p_{\mathrm {T}}$ above 0.3 and 3$~\text {GeV}/c$ and of $\pi ^0$ with $p_{\mathrm {T}} > 4$ $~\text {GeV}/c$ as a function of number of participants $\mathrm {N_{part}}$ [470]

**Fig. 51:**
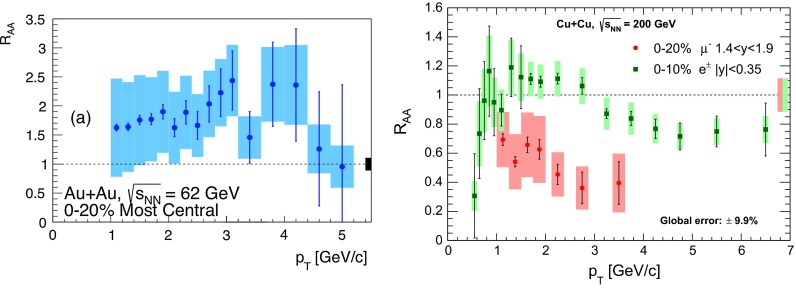
*Left* $R_{\mathrm {AA}}$ of heavy-flavour decay electrons at mid-rapidity measured in Au–Au collisions at $\sqrt{s_{\mathrm{NN}}} = 62.4$ $~\text {GeV}$ as a function of $p_{\mathrm {T}}$ in the 20 % most central collisions [467]. *Right* $R_{\mathrm {AA}}$ of heavy-flavour decay electrons at mid-rapidity [473] and muons at forward rapidity [474] for the most central Cu–Cu collisions at $\sqrt{s_{\mathrm{NN}}} = 200$ $~\text {GeV}$

In PHENIX, muons are measured with two muon spectrometers that provide pion rejection at the level of $2.5 \times 10^{-4}$ in the pseudo-rapidity range $-2.2 < \eta < -1.2$ and $1.2 < \eta < 2.4$ over the full azimuth.

Muons are detected in ALICE with the forward muon spectrometer in the pseudo-rapidity range $-4 < \eta < -2.5$. The extraction of the heavy-flavour contribution to the single muon spectra requires the subtraction of muons from the decay in flight of pions and kaons, estimated through the extrapolations of the measurements at mid-rapidity.

In ATLAS, muons are reconstructed in the pseudo-rapidity range $|\eta |<1.05$ by matching the tracks in the Inner silicon Detector (ID) with the ones in the Muon Spectrometer (MS), surrounding the electromagnetic and hadronic calorimeters. The background muons arise from pion and kaon decays, muons produced in showers in the calorimeters and mis-association of MS and ID tracks. The signal component is extracted through a MC template fit of a discriminant variable that depends on the difference between the ID and MS measurements of the muon momentum, after accounting for energy loss in the calorimeters, and the deflections in the trajectory resulting from decay in flight [483].

The STAR [420] and PHENIX [469–471] Collaborations measured the yield of electrons from heavy-flavour decays at various centre-of-mass energies and in various colliding systems. The $$p_{\mathrm {T}}$$ dependence of the nuclear modification factor measured in the 10 % most central Au–Au collisions at $$\sqrt{s_{\mathrm{NN}}} =200$$$$~\text {GeV}$$ is shown in Fig. 50 (left panel). The suppression increases with the transverse momentum, reaching a factor of about four for $$p_{\mathrm {T}} > 4$$$$~\text {GeV}/c$$. This strong effect is not observed in Au–Au collisions at $$\sqrt{s_{\mathrm{NN}}} = 62.4$$$$~\text {GeV}$$  [467, 484] for which, however, the $$\mathrm pp$$ reference was not measured at RHIC but taken from ISR data (see left panel of Fig. 51). The left panel of Fig. 50 also shows the comparison with the $$R_{\mathrm {AA}}$$ measured in d–Au and Cu–Cu collisions at $$\sqrt{s_{\mathrm{NN}}} =200$$$$~\text {GeV}$$: a clear dependence on the colliding system is found. In particular, the observation that the nuclear modification factor is consistent or larger than unity in d–Au collisions demonstrates that the high-$$p_{\mathrm {T}}$$ suppression in nucleus–nucleus collisions is induced by the presence of the hot and dense medium. The $$R_{\mathrm {AA}}$$ of heavy-flavour decay electrons at mid-rapidity as a function of the collision centrality (represented by the number of participants $$\mathrm {N_{part}}$$) [470] is shown in the right panel of Fig. 50. The high-$$p_{\mathrm {T}}$$ production shows a clear centrality-dependent suppression, reaching a maximum of a factor four in central collisions ($$R_{\mathrm {AA}} \approx 0.25$$). At variance, the production of electrons with $$p_{\mathrm {T}} >0.3$$$$~\text {GeV}/c$$ (which measures the charm production yield essentially down to $$p_{\mathrm {T}}$$$$=$$ 0) is consistent with scaling with the number of binary collisions, within experimental uncertainties of about 20 %. PHENIX also measured the $$R_{\mathrm {AA}}$$ of heavy-flavour decay muons at forward rapidity [474] for the most central Cu–Cu collisions at $$\sqrt{s_{\mathrm{NN}}} =200$$$$~\text {GeV}$$: the observed suppression is stronger than for heavy-flavour decay electrons at mid-rapidity (see Fig. 51, right).

**Fig. 52:**
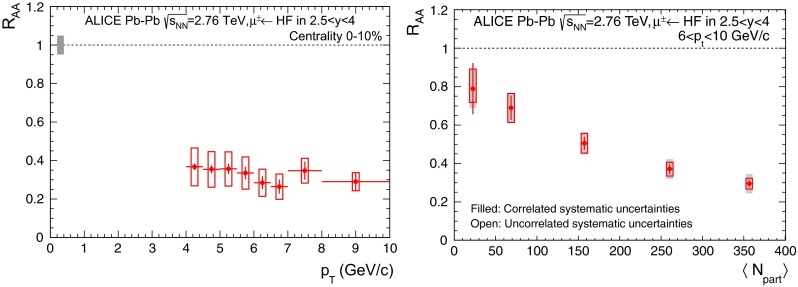
Nuclear modification factor $R_{\mathrm {AA}}$ of heavy-flavour decay muons with $2.5<y<4$ measured in Pb–Pb collisions at $\sqrt{s_{\mathrm{NN}}}$ $=$ 2.76 $\text {TeV}$ as a function of $p_{\mathrm {T}}$ in the 10 % most central collisions (*left panel*) and as a function of the mean number of participating nucleons $\left\langle \mathrm {N_{part}} \right\rangle$ (*right panel*) [120]

**Fig. 53:**
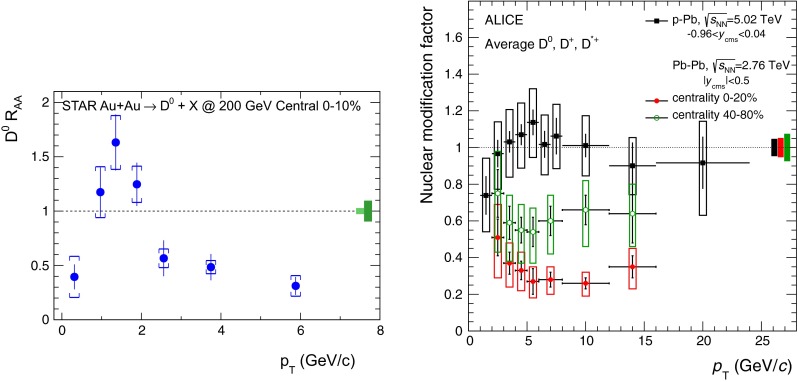
*Left* transverse momentum ($p_{\mathrm {T}}$) dependence of the nuclear modification factor $R_{\mathrm {AA}}$ of $\mathrm {D}^{0}$ mesons in the 10 % most central Au–Au collisions at $\sqrt{s_{\mathrm{NN}}} = 200$ $~\text {GeV}$  [475]. *Right* $R_{\mathrm {AA}}$ of prompt D mesons (averaged) versus $p_{\mathrm {T}}$ for the 0–20 % (*red discs*) and 40–80 % (*green circles*) centrality classes in Pb–Pb collisions at $\sqrt{s_{\mathrm{NN}}} = 2.76$ $\text {TeV}$  [477] and minimum-bias p–Pb collisions at $\sqrt{s_{\mathrm{NN}}} = 5.02$ $\text {TeV}$ (*black squares*) [324]

At the LHC, heavy-flavour production was measured in the leptonic decay channels in Pb–Pb collisions at $$\sqrt{s_{\mathrm{NN}}} = 2.76$$ $$\text {TeV}$$. Figure 52 shows the nuclear modification factors of muons from heavy-flavour decays in $$2.5<y<4$$ measured by ALICE as a function of $$p_{\mathrm {T}}$$ in the 10 % most central collisions (left panel) and as a function of centrality in $$6 < p_{\mathrm {T}} < 10$$$$~\text {GeV}/c$$ (right panel) [120]. The observed suppression increases from peripheral to central collisions, up to a factor of three in central collisions. The result is consistent with a preliminary measurement of the $$R_{\mathrm {AA}}$$ of heavy-flavour decay electrons at mid-rapidity (with $$4<p_{\mathrm {T}} <18$$$$~\text {GeV}/c$$) [485]. Moreover, it is also in qualitative agreement with a preliminary measurement of the heavy-flavour decay muon central-to-peripheral nuclear modification factor $$R_{\mathrm {CP}}$$ at mid-rapidity (with $$4<p_{\mathrm {T}} <14$$$$~\text {GeV}/c$$), carried out by the ATLAS Collaboration [486], which shows a suppression of a factor about two, independent of $$p_{\mathrm {T}}$$, for the centrality ratio 0–10 %/60–80 %. The comparison of the results at forward and mid-rapidity indicate a weak dependence on this variable in the rapidity region $$|y|<4$$.

####  D meson measurements

The differential charm production cross section is determined from measurements of open charm mesons (STAR and ALICE). D mesons are reconstructed via the hadronic decays $\mathrm {D}^{0} \rightarrow \mathrm {K} ^{-} \pi ^{+}$, $\mathrm {D}^{+} \rightarrow \mathrm {K} ^{-} \pi ^{+} \pi ^{+}$, and $\mathrm {D}^{*+} (2010) \rightarrow \mathrm {D}^{0} \pi ^{+}$ with $\mathrm {D}^{0} \rightarrow \mathrm {K} ^{-} \pi ^{+}$, and their charge conjugates. The mean proper decay lengths of $\mathrm {D}^{0}$ and $\mathrm {D}^{+}$ are of about 120 and 300 $\upmu \mathrm{m}$, respectively, while the $\mathrm {D}^{*+}$ decays strongly with no significant separation from the interaction vertex. In the STAR and ALICE experiments, charmed hadrons are measured with an invariant mass analysis of the fully reconstructed decay topologies in the hadronic decay channels. In both experiments, the kaon and pion identification is performed by combining the information of the Time Of Flight and of the specific ionisation energy loss in the TPC [103, 104, 125]. The spatial resolution of the ALICE silicon tracker allows, in addition, to reconstruct the decay vertex and apply a topological selection on its separation from the interaction vertex [104].

The left panel of Fig. 53 shows the transverse momentum dependence of the nuclear modification factor $$R_{\mathrm {AA}}$$ for $$\mathrm {D}^{0}$$ mesons in the most central Au–Au collisions at $$\sqrt{s_{\mathrm{NN}}} = 200$$$$~\text {GeV}$$ from the STAR experiment [475]. The $$R_{\mathrm {AA}}$$ is enhanced at around 1.5$$~\text {GeV}/c$$ and shows a strong suppression at $$p_{\mathrm {T}} > 3$$$$~\text {GeV}/c$$. STAR also measured $$\mathrm {D}^{0}$$ mesons in U–U collisions at $$\sqrt{s_{\mathrm{NN}}} = 193$$$$~\text {GeV}$$ and observed a similar trend for the $$R_{\mathrm {AA}}$$ as seen in Au–Au collisions [487].

The ALICE experiment measured the production of prompt $$\mathrm {D}^{0} $$, $$\mathrm {D}^{+} $$ and $$\mathrm {D}^{*+} $$ mesons in Pb–Pb collisions at $$\sqrt{s_{\mathrm{NN}}} = 2.76$$ $$\text {TeV}$$  [477]. The average $$R_{\mathrm {AA}}$$ of D mesons for two centrality classes is shown in the right panel of Fig. 53. The high-$$p_{\mathrm {T}}$$ D-meson yield for the most central events is strongly suppressed (by factor of about four at 10$$~\text {GeV}/c$$). The analysis of the Pb–Pb data collected in 2011 allowed to extend the measurement to higher transverse momenta: a similar suppression pattern is observed up to $$p_{\mathrm {T}} =30$$$$~\text {GeV}/c$$ in the 7.5 % most central collisions [488]. In addition, the $$\mathrm {D}^{+}_{s}$$ meson, consisting of a charm and an antistrange quark, was measured for the first time in Pb–Pb collisions [489]. The $$\mathrm {D}^{+}_{s}$$ meson is expected to be sensitive to the possible hadronisation of charm quarks via recombination with light quarks from the medium: the expected abundance of strange quarks in the QGP may lead to an increase of the ratio of strange over non-strange D mesons with respect to $$\mathrm pp$$ collisions in the momentum range where recombination can be relevant [490, 491]. The observed central value of the $$\mathrm {D}^{+}_{s}$$$$R_{\mathrm {AA}}$$ is larger than that of $$\mathrm {D}^{0}$$, $$\mathrm {D}^{+}$$ and $$\mathrm {D}^{*+}$$ mesons at low $$p_{\mathrm {T}}$$, although the large statistical and systematic uncertainties prevent from drawing any conclusion.

Initial-state effects were investigated by the ALICE Collaboration by measuring D production in p–Pb collisions [324] (see Sect. 3.3.2). The nuclear modification factor of prompt D mesons in minimum-bias p–Pb at $$\sqrt{s_{\mathrm{NN}}} = 5.02$$ $$\text {TeV}$$ is shown in Fig. 53 (right panel). The $$R_{\mathrm {AA}}$$ is compatible with unity within systematic uncertainties. This indicates that the suppression of the D-meson yield observed for $$p_{\mathrm {T}} >3$$$$~\text {GeV}/c$$ in central Pb–Pb collisions is a final-state effect, most likely induced by the interactions of charm quarks within the QGP.

**Fig. 54:**
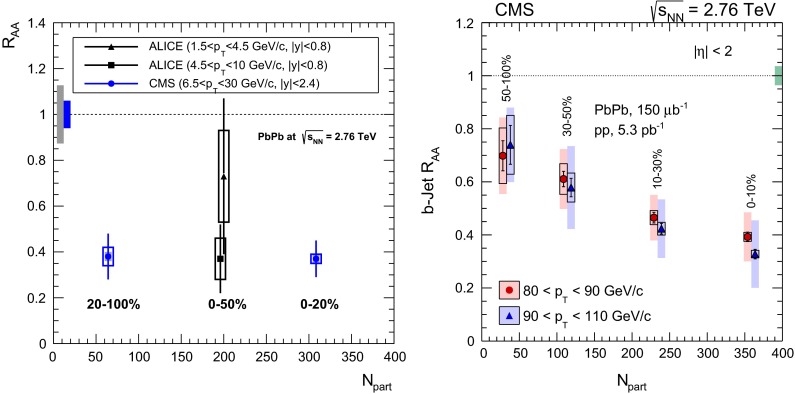
*Left* non-prompt $\mathrm {J}/\psi$ $R_{\mathrm {AA}}$ measured in two centrality bins from CMS [482] and in one centrality bin for two $p_{\mathrm {T}}$ ranges from ALICE [480]. The ALICE points are slightly shifted horizontally for better visibility. The correlated uncertainties are shown as *filled box* at $R_{\mathrm {AA}}$ $=$ 1. *Right* $R_{\mathrm {AA}}$ of $b$ jets, as a function of $\mathrm {N_{part}}$ from CMS [481], for two jet $p_{\mathrm {T}}$ selections as indicated in the legend. Systematic uncertainties are shown as *filled boxes*, except the $T_{\mathrm {AA}}$ uncertainties, depicted as *open boxes*. The luminosity uncertainty is represented by the *green box*

#### Beauty production measurements

The detection and identification of beauty hadrons usually exploit their long life times, with $c\tau$ values of about 500 $\upmu$m. Precise charged particle tracking and vertexing are of crucial importance, with the required resolution of the track impact parameter in the transverse plane being of the order of 100 $\upmu$m. Most decay channels proceed as a $b \rightarrow c$ hadron cascade, giving rise to a topology that contains both a secondary and a tertiary decay vertex.

Lepton identification is often exploited in beauty measurements, as the semi-leptonic branching ratio is about 20 %, taking into account both decay vertices. The beauty contribution can be extracted from the semi-electronic decays of heavy flavours through a fit of the impact-parameter distribution. This approach was applied by the ALICE Collaboration in $\mathrm pp$ collisions at the LHC [107, 123] (see Sect. 2.2.3) and recently also in Pb–Pb collisions [492], where preliminary results indicate $R_{\mathrm {AA}}$ values below unity for electron $p_{\mathrm {T}}$ larger than about 5$~\text {GeV}/c$. The charm and beauty contribution can be disentangled also by studying the correlations between electrons and associated charged hadrons, exploiting the larger width of the near-side peak for B hadron decays [107, 115, 493]. The main limitation of the beauty measurements via single electrons (or muons) is the very broad correlation between the momentum of the measured electron and the momentum of the parent B meson.

A more direct measurement is achieved using the inclusive $$\mathrm{B}\rightarrow \mathrm {J}/\psi + X$$ decay mode. Such decays can be measured inclusively by decomposing the $$\mathrm {J}/\psi $$ yield into its prompt and non-prompt components, using a fit to the lifetime distribution. The first measurement with this technique in heavy-ion collisions was performed by the CMS Collaboration, using data from the 2010 Pb–Pb run. The $$R_{\mathrm {AA}}$$ of non-prompt $$\mathrm {J}/\psi $$ in $$6.5 < p_{\mathrm {T}} < 30$$$$~\text {GeV}/c$$ and $$|y| < 2.4$$ was measured to be $$0.37 \pm 0.08 \mathrm{(stat.)} \pm 0.02 \mathrm{(syst.)}$$ in the 20 % most central collisions (see left panel of Fig. 54) [482]. Preliminary measurements from the larger 2011 dataset explore the $$p_{\mathrm {T}}$$ dependence of $$R_{\mathrm {AA}}$$  [494]. A recent measurement from the ALICE Collaboration [480] (left panel of Fig. 54) shows a similar value of $$R_{\mathrm {AA}}$$ in a close kinematic range ($$4.5<p_{\mathrm {T}} <10$$$$~\text {GeV}/c$$ and $$|y|<0.8$$).

Further insights into the parton energy loss can be provided through measurements of reconstructed jets and comparison with theory [495], which is complementary to the studies on B hadrons as the reconstructed jet energy is closely related to that of the $b$ quark. Assuming that the quark hadronises outside the medium, to first approximation the jet energy represents the sum of the parton energy after its interaction with the medium, as well as any transferred energy that remains inside the jet cone. CMS has performed a measurement of $b$ jets in Pb–Pb collisions by direct reconstruction of displaced vertices associated to the jets [482]. Despite the large underlying Pb–Pb event, a light jet rejection factor of about 100 can still be achieved in central Pb–Pb events. The $R_{\mathrm {AA}}$ of $b$ jets as a function of centrality is shown in Fig. 54 (right), for two ranges of jet $p_{\mathrm {T}}$. The observed suppression, which reaches a value of about 2.5 in central collisions, does not show any significant difference compared to a similar measurement of the inclusive jet $R_{\mathrm {AA}}$  [496] within the sizeable systematic uncertainties. While quark mass effects may not play a role at such large values of $p_{\mathrm {T}}$, the difference in energy loss between quarks and gluons should manifest itself as a difference in $R_{\mathrm {AA}}$ for $b$ jets and inclusive jets, as the latter are dominated by gluon jets up to very large $p_{\mathrm {T}}$. It should be noted, however, that the $b$ jets do not always originate from a primary $b$ quark. As discussed in the introduction to this Section, at LHC energies, a significant component of $b$ quarks are produced by splitting of gluons into ${b\overline{b}}$ pairs [447]. For $b$ jets with very large $p_{\mathrm {T}}$ a significant part of the in-medium path length is likely to be traversed by the parent gluon, as opposed to the $b$ quarks (for example, about 1–2 fm for $b$ quarks with $p_{\mathrm {T}}$ of 100–200$~\text {GeV}/c$). The gluon splitting contribution can be minimised by selecting hard fragments, although this is complicated by the fact that the $b$–hadron kinematics are not fully measured. An alternative is to select back-to-back $b$-tagged jets, a configuration in which the gluon splitting contribution is negligible. The dijet asymmetry of $b$ jets can then be compared to that of inclusive jets, a measurement that should be feasible with the luminosity expected from the upcoming LHC Run 2.

#### Comparison of $R_{\mathrm {AA}}$ for charm, beauty and light-flavour hadrons

**Fig. 55:**
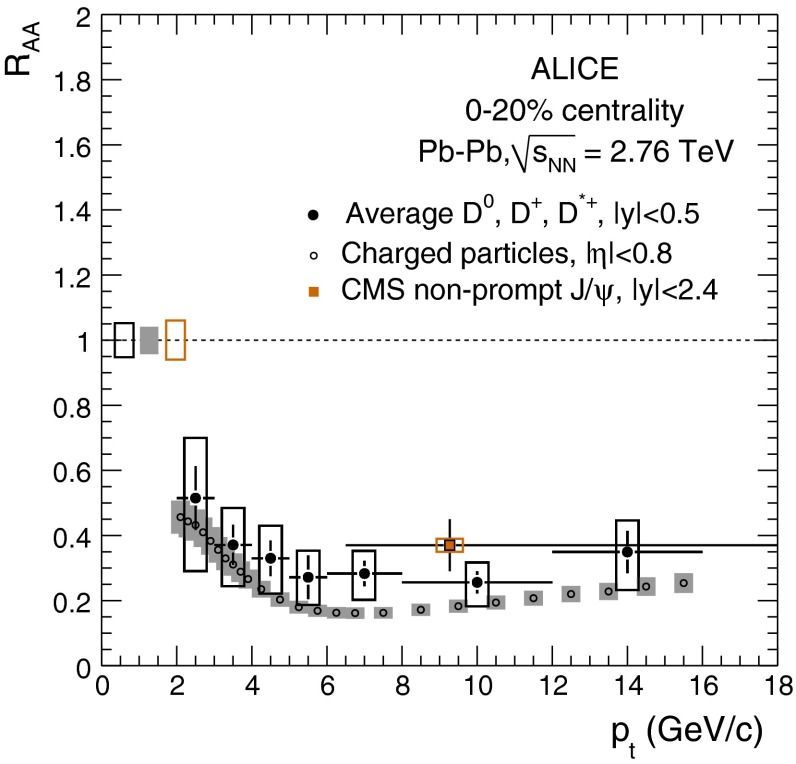
$R_{\mathrm {AA}}$ of D mesons, charged hadrons [477] and non-prompt $\mathrm {J}/\psi$  [482] in Pb–Pb collisions at $\sqrt{s_{\mathrm{NN}}} =2.76~\text {TeV}$ in the 0–20 % centrality class

The expected dependence of in-medium energy loss on the parton colour charge and mass can be investigated by comparing the nuclear modification factor of charged hadrons, mostly originating from gluon fragmentation at the LHC collision energy, with that of hadrons with charm and beauty. The comparison between D-meson and charged particle $$R_{\mathrm {AA}}$$, measured by the ALICE Collaboration [477] in Pb–Pb collisions at LHC in the centrality class 0–20 % and illustrated in Fig. 55, shows that the two nuclear modification factors are compatible within uncertainties, although the central values show an indication for $$R_{\mathrm {AA}} ^\mathrm{D}>R_{\mathrm {AA}} ^\mathrm{charged}$$. In the same figure, the nuclear modification factor measured by the CMS Collaboration for non-prompt $$\mathrm {J}/\psi $$ mesons (from B decays) with $$p_{\mathrm {T}} >6.5$$$$~\text {GeV}/c$$  [482] is also shown. Their suppression is clearly weaker than that of charged particles, while the comparison with D mesons is not conclusive, because of the significant uncertainties of the two measurements. In addition, it is worth noting that the $$p_{\mathrm {T}}$$ of the $$\mathrm {J}/\psi $$ is shifted with respect to the one of the parent B meson (by about 2–3$$~\text {GeV}/c$$ on average in the $$p_{\mathrm {T}}$$ range covered by the CMS measurement), hence the comparison with D mesons is not straightforward.

**Fig. 56:**
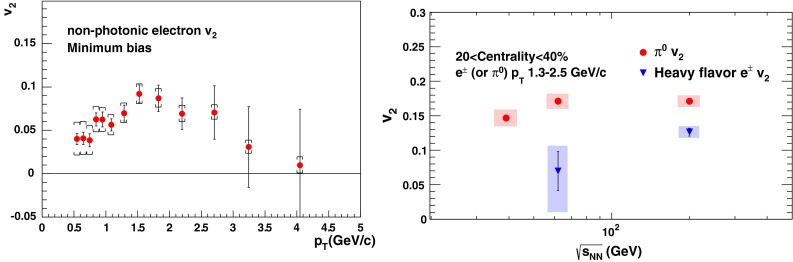
Heavy-flavour decay-electron $v_{2}$ measured by the PHENIX Collaboration in Au–Au collisions at RHIC. *Left* measurement in minimum-bias collisions as a function of $p_{\mathrm {T}}$ at $\sqrt{s_{\mathrm{NN}}} = 200$ $~\text {GeV}$  [469]. *Right* measurements at $\sqrt{s_{\mathrm{NN}}} =62.4$ and 200$~\text {GeV}$ in the 20–40 % centrality class for the interval $1.3<p_{\mathrm {T}} <2.5$ $~\text {GeV}/c$, compared with the $\pi ^0$ $v_{2}$  [467]

Preliminary measurements based on higher-statistics data from the 2011 Pb–Pb run at LHC provide a first indication that the nuclear modification factor of B mesons is larger than that of D at transverse momentum of about 10$$~\text {GeV}/c$$. The measurements were carried out, as a function of collision centrality, for D mesons with $$8<p_{\mathrm {T}} <16$$$$~\text {GeV}/c$$ and $$|y|<0.5$$ by the ALICE Collaboration [497] and for non-prompt $$\mathrm {J}/\psi $$ mesons with $$6.5<p_{\mathrm {T}} <30$$$$~\text {GeV}/c$$ and $$|y|<1.2$$ by the CMS Collaboration [494]. With these $$p_{\mathrm {T}}$$ intervals, the average $$p_{\mathrm {T}}$$ values of the probed D and B mesons are both about 10–11$$~\text {GeV}/c$$. In central collisions (centrality classes 0–10 and 10–20 %) the $$R_{\mathrm {AA}}$$ values are of about 0.2 and 0.4 for D and non-prompt $$\mathrm {J}/\psi $$ mesons, respectively, and they are not compatible within experimental uncertainties. This experimental observation alone does not allow one to draw conclusions on the comparison of energy loss of charm and beauty quarks, because several kinematic effects contribute to the $$R_{\mathrm {AA}}$$ resulting from a given partonic energy loss. In particular, the shape of the quark $$p_{\mathrm {T}}$$ distribution (which is steeper for charm than for beauty quarks) and the shape of the fragmentation function (which is harder for $$b\rightarrow \mathrm B$$ than for $$c\rightarrow \mathrm D$$) have to be taken into account. Model calculations of heavy-quark production, in-medium propagation and fragmentation provide a tool to consistently consider these effects in the comparison of charm and beauty measurements. In Sect. 4.5 we will show that model calculations including a mass-dependent energy loss result in $$R_{\mathrm {AA}}$$ values significantly larger for $$\mathrm {J}/\psi $$ from B decays than for D mesons, consistently with the preliminary results from the ALICE and CMS experiments.

### Experimental overview: azimuthal anisotropy measurements

As mentioned in the introduction to this chapter, the azimuthal anisotropy of particle production in heavy-ion collisions is measured using the Fourier expansion of the azimuthal angle ($\phi$) and the $p_{\mathrm {T}}$-dependent particle distribution $\mathrm {d}^2 N / \mathrm {d}p_{\mathrm {T}} \mathrm {d}\phi$. The second coefficient, $v_{2}$ or elliptic flow, which is the dominant component of the anisotropy in non-central nucleus–nucleus collisions, is measured using these three methods: event plane (EP) [498], scalar product (SP) [499] and multi-particle cumulants [500]. In the following, an overview of the elliptic flow measurements for heavy-flavour particles is presented: the published measurements at RHIC use heavy-flavour decay electrons (Sect. 4.2.1); the published measurements at LHC use D mesons (Sect. 4.2.2).

#### Inclusive measurements with electrons

The measurement of the production of heavy-flavour decay electrons has been presented in Sect. 4.1.1. In order to determine the heavy-flavour decay-electron $v_{2}$, the starting point is the measurement of $v_{2}$ for inclusive electrons. Inclusive electrons include, mainly, the so-called photonic (or background) electrons (from photon conversion in the detector material and internal conversions in the Dalitz decays of light mesons), a possible contamination from hadrons, and heavy-flavour decay electrons. Exploiting the additivity of $v_{2}$, the heavy-flavour decay-electron $v_{2}$ is obtained by subtracting from the inclusive electron $v_{2}$ the $v_{2}$ of photonic electrons and hadrons, weighted by the corresponding contributions to the inclusive yield.

The PHENIX Collaboration measured the heavy-flavour decay-electron $$v_{2}$$ in Au–Au collisions at $$\sqrt{s_{\mathrm{NN}}} = 62.4$$ and 200$$~\text {GeV}$$ using the event-plane method [467, 469]. Electrons were detected at mid-rapidity $$|\eta | < 0.35$$ in the interval $$0.5 < p_{\mathrm {T}} < 5$$$$~\text {GeV}/c$$. The event plane was instead determined using charged particles at forward rapidity $$3.0 < |\eta | < 3.9$$. This large $$\eta $$-gap is expected to reduce the non-flow effects (like auto-correlations) in the $$v_{2}$$ measurement. Figure 56 (left) shows the heavy-flavour decay-electron $$v_{2}$$ for minimum-bias events (without any selection on centrality) [469]. $$v_{2}$$ is larger than zero in the interval $$0.5<p_{\mathrm {T}} <2.5$$$$~\text {GeV}/c$$, with a maximum value of about 0.1 at $$p_{\mathrm {T}}$$ of about 1.5$$~\text {GeV}/c$$. Towards larger $$p_{\mathrm {T}}$$ the data suggest a

**Fig. 57:**
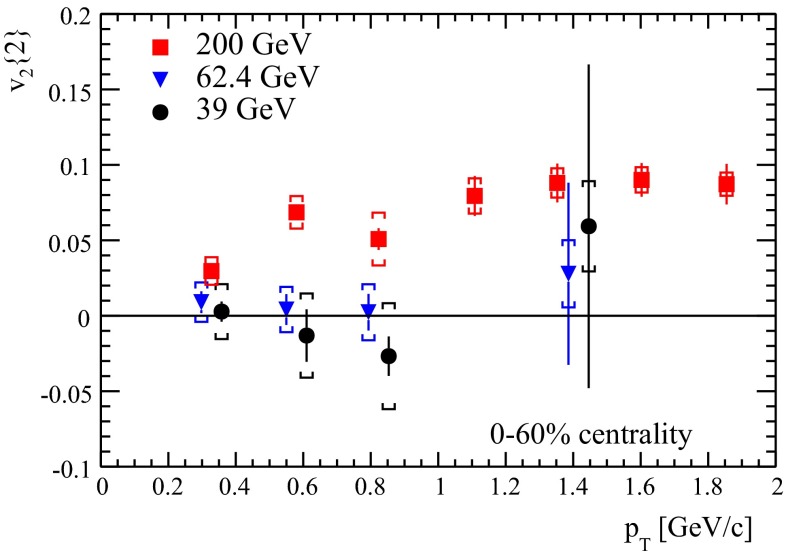
Heavy-flavour decay-electron $v_{2}$ measured by the STAR Collaboration in Au–Au collisions (0–60 % centrality class) at centre-of-mass energies 39, 62.4 and 200$~\text {GeV}$  [476]

**Fig. 58:**
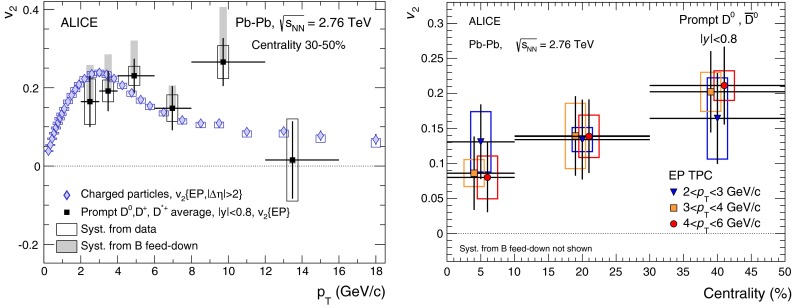
D-meson elliptic flow measured by the ALICE Collaboration in Pb–Pb collisions at $\sqrt{s_{\mathrm{NN}}} =2.76$ $\text {TeV}$  [478, 479]. *Left* average of the $v_{2}$ values for $\mathrm {D}^{0}$, $\mathrm {D}^{+}$ and $\mathrm {D}^{*+}$ mesons in the centrality class 30–50 % as a function of $p_{\mathrm {T}}$, compared with the $v_{2}$ of charged particles. *Right* centrality dependence of the $\mathrm {D}^{0}$ meson $v_{2}$ for three $p_{\mathrm {T}}$ intervals

decreasing trend, although the statistical uncertainties prevent a firm conclusion. The study of the centrality dependence of $$v_{2}$$ (not shown) indicates a maximum effect in the two semi-peripheral centrality classes (20–40 and 40–60 %), for which the initial spatial anisotropy is largest [469]. The central value of the heavy-flavour electron $$v_{2}$$ in Au–Au collisions at $$\sqrt{s_{\mathrm{NN}}} = 62.4$$$$~\text {GeV}$$  [467] is significantly lower than at 200$$~\text {GeV}$$ [see Fig. 56 (right)]. However, the statistical and systematic uncertainties are sizeable and do not allow one to conclude firmly on the energy dependence of $$v_{2}$$. In Fig. 56 (right) the measurements for heavy-flavour decay electrons with $$1.3<p_{\mathrm {T}} <2.5$$$$~\text {GeV}/c$$ are compared with those for neutral pions with the same $$p_{\mathrm {T}}$$: the pions exhibit a larger $$v_{2}$$ than the electrons; however, this comparison should be taken with care, because the $$p_{\mathrm {T}}$$ of the heavy-flavour mesons is significantly larger than that of their decay electrons.

The STAR Collaboration measured the heavy-flavour decay-electron $$v_{2}$$ in Au–Au collisions at $$\sqrt{s_{\mathrm{NN}}} = 39, ~62.4$$ and 200$$~\text {GeV}$$  [476]. The two-particle cumulant method was used to measure the elliptic flow for the two lower collision energies. The event plane, and both two- and four-particle cumulant methods were used at $$\sqrt{s_{\mathrm{NN}}} = 200$$$$~\text {GeV}$$. Figure 57 shows the $$v_{2}$$ measured with two-particle cumulants at the three centre-of-mass energies. At $$\sqrt{s_{\mathrm{NN}}} = 200$$$$~\text {GeV}$$ the measurement shows a $$v_{2}$$ larger than zero for $$p_{\mathrm {T}} >0.3$$$$~\text {GeV}/c$$, compatible with the measurement by the PHENIX Collaboration in the same centrality class (see comparison in [476]). At $$\sqrt{s_{\mathrm{NN}}} = 39$$ and 62.4$$~\text {GeV}$$, the $$v_{2} \{2\}$$ values are consistent with zero within uncertainties.

Preliminary results by the ALICE Collaboration on the elliptic flow of heavy-flavour decay electrons at central rapidity ($|y|<0.6$) and of heavy-flavour decay muons at forward rapidity ($2.5<y<4$) in Pb–Pb collisions at the LHC show a $v_{2}$ significantly larger than zero in both rapidity regions and with central values similar to those measured at top RHIC energy [501].

#### D-meson measurements

The ALICE Collaboration measured the $$v_{2}$$ of prompt D mesons in Pb–Pb collisions at $$\sqrt{s_{\mathrm{NN}}} =2.76$$ $$\text {TeV}$$  [478, 479]. The D mesons ($$\mathrm {D}^{0} $$, $$\mathrm {D}^{+} $$ and $$\mathrm {D}^{*+} $$) were measured in $$|y| < 0.8$$ and $$2 < p_{\mathrm {T}} < 16$$$$~\text {GeV}/c$$ using their hadronic decay channels, and exploiting the separation of a few hundred $$\upmu $$m of the decay vertex from the interaction vertex to reduce the combinatorial background. The measurement of D-meson $$v_{2}$$ was carried out using the event plane, the scalar product and the two-particle cumulant methods.

Figure 58 (left) shows the average of the $$v_{2}$$ measurements for $$\mathrm {D}^{0} $$, $$\mathrm {D}^{+} $$ and $$\mathrm {D}^{*+} $$ in the centrality class 30–50 % as a function of $$p_{\mathrm {T}}$$  [479]. The measurement shows a $$v_{2}$$ larger than zero in the interval $$2 < p_{\mathrm {T}} < 6$$$$~\text {GeV}/c$$ with a $$5.7\sigma $$ significance. In the same figure, the $$v_{2}$$ of charged particles for the same centrality class is reported for comparison: the magnitude of $$v_{2}$$ is similar for charmed and light-flavour hadrons. Figure 58 (right) shows the dependence on collision centrality of the $$\mathrm {D}^{0} $$ meson $$v_{2}$$ for three $$p_{\mathrm {T}}$$ intervals. An increasing trend of $$v_{2}$$ towards more peripheral collisions is observed, as expected due to the increasing initial spatial anisotropy.

**Fig. 59:**
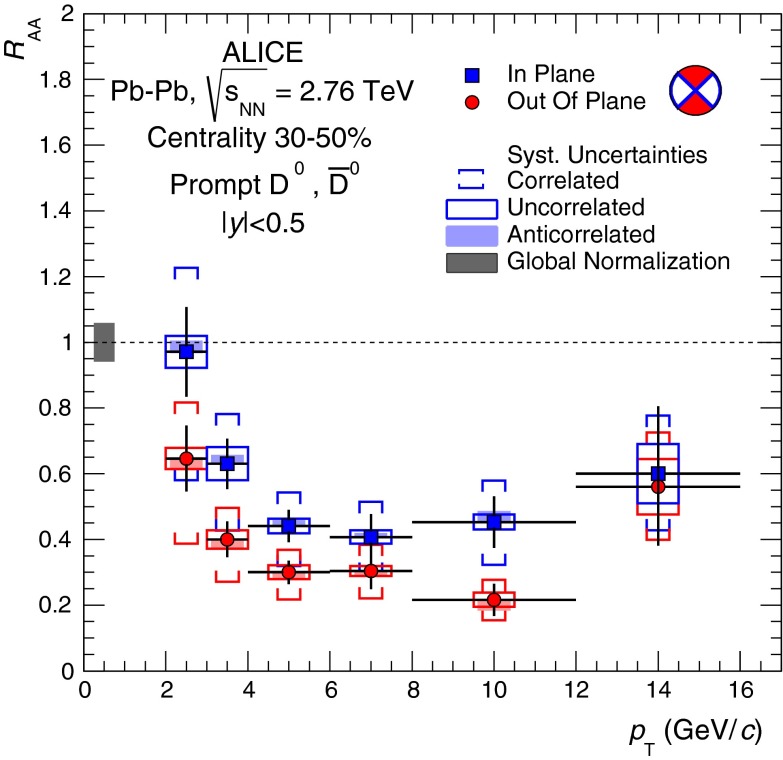
D-meson $R_{\mathrm {AA}}$ in the direction of the event plane and in the direction orthogonal to the event plane, measured by the ALICE Collaboration in Pb–Pb collisions (centrality class 30–50 %) at $\sqrt{s_{\mathrm{NN}}} =2.76$ $\text {TeV}$  [478, 479]

As discussed at the beginning of this Section, the azimuthal dependence of the nuclear modification factor $R_{\mathrm {AA}}$ can provide insight into the path length dependence of heavy-quark energy loss. The nuclear modification factor of $\mathrm {D}^{0}$ mesons in Pb–Pb collisions (30–50 % centrality class) was measured by the ALICE Collaboration in the direction of the event plane (in-plane) and in the direction orthogonal to the event plane (out-of-plane) [478]. The results, shown in Fig. 59, exhibit a larger high-$p_{\mathrm {T}}$ suppression in the out-of-plane direction, where the average path length in the medium is expected to be larger. It is worth noting that the difference between the values of $R_{\mathrm {AA}}$ in-plane and $R_{\mathrm {AA}}$ out-of-plane is equivalent to the observation of $v_{2} > 0$, because the three observables are directly correlated.

### Theoretical overview: heavy flavour interactions in the medium

The approaches describing the heavy-quark–medium interactions aim at determining the probability $\mathcal{P}_{Q\rightarrow H}(p_Q^\mathrm{in},p_{H}^\mathrm{fin})$ that a given heavy quark produced with a four-momentum $p_Q^\mathrm{in}$ escapes the medium as a heavy-flavour hadron of four-momentum $p_{H}^\mathrm{fin}$.

All the approaches include a description of the interactions that occur between the heavy quarks and the partonic constituents of the QGP. For ultra-relativistic heavy quarks ($p_Q\gg m_Q$, say $>10\,m_Q$), the dominant source of the energy loss is commonly considered to be the radiation of gluons resulting from the scattering of the heavy quark on the medium constituents. These are $2\rightarrow 3$ processes $q(g)Q\rightarrow q(g)Qg$, where *q*(*g*) is a medium light quark (or gluon). As this mechanism proceeds through long formation times, several scatterings contribute coherently and quantities like the total energy loss $\Delta E(L)=p_Q^\mathrm{in}-p_Q^\mathrm{fin}$ can only be evaluated at the end of the in-medium path length *L*. This feature is shared by all schemes that have been developed to evaluate radiative energy loss of ultra-relativistic partons [456–458]. For merely relativistic heavy quarks (say $p_Q<10\,m_Q$), elastic (collisional) processes are believed to have an important role as well. These are $2\rightarrow 2$ process $q(g)Q\rightarrow q(g)Q$. The in-medium interactions are gauged by the following, closely related, variables: the *mean free path*$\lambda =1/(\sigma \rho )$ is related to the medium density $\rho$ and to the cross section $\sigma$ of the parton-medium interaction (for $2\rightarrow 2$ or $2\rightarrow 3$ processes); the *Debye mass*$m_D$ is the inverse of the screening length of the colour electric fields in the medium and it is proportional to the temperature *T* of the medium; the *transport coefficients* encode the momentum transfers with the medium (more details are given in the next paragraph).

In the relativistic regime, the gluon formation time for radiative processes becomes small enough that the energy-loss probability $\mathcal{P}(\Delta E)$ can be evaluated as a result of some local transport equation– like the Boltzmann equation, relying on local cross sections – evolving from initial to final time. This simplification can be applied also to collisional processes. When the average momentum transfer is small with respect to the quark mass,^19^ the Boltzmann equation can be reduced to the Fokker–Planck equation, which is often further simplified to the Langevin stochastic equation (see [466] for a recent review). These linear partial-differential equations describe the time-evolution of the momentum distribution $f_Q$ of heavy quarks. The medium properties are encoded in three transport coefficients: (a) the *drift coefficient* – also called *friction* or *drag coefficient* – which represents the fractional momentum loss per unit of time in the absence of fluctuations and admits various equivalent symbolic representations ($\eta _D,\,A_Q,\,\ldots$) and (b) the *longitudinal* and *transverse momentum diffusion coefficients*$B_\mathrm{L}$ and $B_\mathrm{T}$ (or $B_1$ and $B_0$, $\kappa _\mathrm{L}$ and $\kappa _\mathrm{T}$,..., depending on the authors), which represent the increase of the variance of $f_Q$ per unit of time. For small momentum, the drift and diffusion coefficients are linked through the Einstein relation $B=m_Q\,\eta _D\,T$ and also uniquely related to the spatial diffusion coefficient $D_s$, which describes the spread of the distribution in physical space with time. Although the Fokker–Planck approach has some drawbacks,^20^ it can also be deduced from more general considerations [503], so that it may still be considered as a valid approach for describing heavy-quark transport even when the Boltzmann equation does not apply, as for instance in the strong-coupling limit.

Some of the approaches consider only partonic interactions and define the $\mathcal{P}_{Q\rightarrow H}$ probability as a convolution of $\mathcal{P}_{Q\rightarrow Q'}(p_Q^\mathrm{in},p_Q^\mathrm{fin})$ – the probability for the heavy quark to lose $p_Q^\mathrm{in}-p_Q^\mathrm{fin}$ in the medium – with the unmodified fragmentation function. A number of approaches also include, for low-intermediate momentum heavy quarks, a contribution of hadronisation via recombination (also indicated as coalescence). Finally, some approaches consider late-stage interactions of the heavy-flavour hadrons with the partonic or hadronic medium.

In this section, we summarise the various approaches for the calculation of the heavy-quark interactions within the medium.

- Sections 4.3.1 and 4.3.2 are devoted to pQCD and pQCD-inspired calculations of radiative and collisional energy loss, as developed by Gossiaux et al. (MC$@_s$HQ), Beraudo et al. (POWLANG), Djordjevic et al., Vitev et al. and Uphoff et al. (BAMPS); examples of the relative energy loss ($\Delta E/E$) and the momentum loss per unit length ($\mathrm {d}P/\mathrm {d}t$) for $c$ and $b$ quarks are shown.
- Section 4.3.3 focuses on the calculation by Vitev et al. of in-medium formation and dissociation of heavy-flavour hadrons; this proposed mechanism is expected to effectively induce an additional momentum loss with respect to radiative and collisional heavy-quark in-medium interactions alone.
- Sections 4.3.4 and 4.3.5 describe the calculation of transport coefficients through *T*-matrix approach supplemented with a non-perturbative potential extracted from lattice QCD (Rapp et al., TAMU) or through direct ab initio lattice-QCD calculations (Beraudo et al., POWLANG); the transport coefficients that are discussed are the spatial diffusion coefficient (or friction coefficient), for which examples are shown, and the momentum diffusion coefficient.
- Section 4.3.6 presents the AdS/CFT approach for the calculation of the transport coefficients, developed by Horowitz et al..

**Fig. 60:**
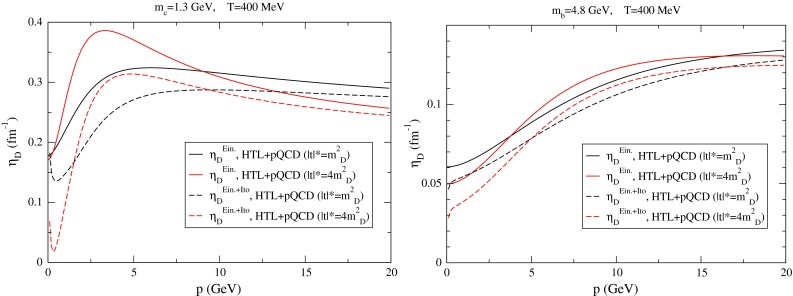
The charm (*left panel*) and beauty (*right panel*) friction coefficients in a Quark Gluon Plasma at a temperature $T = 400$ $~\text {MeV}$. *Continuous curves* refer to the results of the Einstein relation $\eta _D=\kappa _\mathrm{L}/2ET$, while *dashed curves* include the discretisation correction in Eq. (31) within the Ito scheme [510]. The sensitivity of the results to the intermediate cut-off $|t|*$ separating hard and soft collisions is also displayed

The implementation of these various approaches in full models that allow one to compute the final heavy-flavour hadron kinematic distributions will be described in Sect. 4.4, with particular emphasis on the modelling of the QGP and its evolution.

Given the focus of this review, we have chosen not to discuss the theoretical approaches that were not yet applied to LHC energies at the time of writing the document. For example, the modelling of heavy quark energy loss within the Dynamical Quasi-Particle Model (DQPM) approach in [504, 505], recently integrated in the PHSD transport theory [506], appears to be quite promising.

#### pQCD energy loss in a dynamical QCD medium

Within a weak-coupling approach the interaction of heavy quarks with the medium can be described in terms of the uncorrelated scatterings with the light quarks and gluons of the hot deconfined plasma. Neglecting radiative processes one can then attempt an evaluation of the heavy-flavour transport coefficients arising from the $2\rightarrow 2$ elastic collisions suffered in the medium: this was the approach followed in Refs. [507, 508], which we briefly summarise. The approach developed by the authors to simulate the propagation of the heavy quarks in the QGP was based on the relativistic Langevin equation (here written in its discretised form)

29 $$\begin{aligned} \frac{\Delta \vec {p}}{\Delta t}= & {} -\eta _D(p)\vec {p}+\vec {\xi }(t) \quad \mathrm{with}\nonumber \\&\langle \xi ^i(t)\xi ^j(t')\rangle =b^{ij}(\vec {p})\delta _{tt'}/\Delta t \end{aligned}$$

where

30 $$\begin{aligned} b^{ij}(\vec {p})= \kappa _\mathrm{L}(p)\hat{p}^i\hat{p}^j+\kappa _\mathrm{T}(p) (\delta ^{ij}-\hat{p}^i\hat{p}^j). \end{aligned}$$

The right-hand side is given by the sum of a deterministic friction force and a stochastic noise term. The interaction with the background medium is encoded in the transport coefficients $\kappa _{T/L}$ describing the average squared transverse/longitudinal momentum per unit time exchanged with the plasma. In Refs. [507, 508] the latter were evaluated within a weak-coupling set-up, accounting for the collisions with the gluons and light quarks of the plasma. In particular, hard interactions were evaluated through kinetic pQCD calculation; soft collisions, involving the exchange of long-wavelength gluons, required the resummation of medium effects, the latter being provided by the Hard Thermal Loop (HTL) approximation. The friction coefficient $\eta _D(p)$ appearing in Eq. (29) has to be fixed in order to ensure the approach to thermal equilibrium through the Einstein fluctuation–dissipation relation

31 $$\begin{aligned} \eta _D^\mathrm{(Ito)}(p)\equiv & {} \frac{\kappa _\mathrm{L}(p)}{2TE_p}\nonumber \\&-\frac{1}{2p}\left[ \partial _p\kappa _\mathrm{L}(p)+\frac{d-1}{2}(\kappa _\mathrm{L}(p)-\kappa _\mathrm{T}(p))\right] . \end{aligned}$$

In the above, the second term in the right-hand side is a correction (sub-leading by a *T* / *p* factor) depending on the discretisation scheme employed in the numerical solution of the stochastic differential equation (Eq. (29)) in the case of momentum dependent transport coefficients (for more details see Ref. [509]): it ensures that, in the continuum $\Delta t\rightarrow 0$ limit, one recovers a Fokker–Planck equation with a proper Maxwell–Jüttner equilibrium distribution as a stationary solution. Here we have written its expression in the so-called Ito scheme [510], which is the most convenient for a numerical implementation. Results for the friction coefficient $\eta _D(p)$ of $c$ and $b$ quarks are displayed in Fig. 60.

The radiative processes, which are neglected in the model described above, are taken into account in other approaches. Djordjevic et al. developed a state-of-the-art dynamical energy-loss formalism, which (i) is applicable for both light and heavy partons, (ii) computes both radiative [511, 512] and collisional [513] energy loss in the *same* theoretical framework, (iii) takes into account recoil of the medium constituents, i.e. the fact that medium partons are moving (i.e. dynamical) particles, (iv) includes realistic finite size effects, i.e. the fact that the partons are produced inside the medium and that the medium has finite size. Recently, the formalism was also extended to include (v) finite magnetic mass effects [514] and (vi) running coupling (momentum dependence of $\alpha _s$) [515].

In this formalism, radiative and collisional energy losses are calculated for an optically thin dilute QCD medium. Consequently, both collisional and radiative energy losses are computed to the leading order. That is, for collisional energy loss, the loss is calculated for one collisional interaction with the medium, while for radiative energy loss, the loss is calculated for one interaction with the medium accompanied by the emission of one gluon. The medium is described as a thermalised QGP [516, 517] at temperature *T* and zero baryon density, with $n_f$ effective massless quark flavours in equilibrium with the gluons. The Feynman diagrams contributing to the collisional and the radiative quark energy loss are presented in Refs. [511, 513]. A full account of the calculation is presented in Ref. [513] for collisional energy loss, and in Ref. [511] for radiative energy loss. Since the expression for collisional energy loss is lengthy, it will not be presented here, while the expression for radiative energy loss is given by

32 $$\begin{aligned} \frac{\Delta E_{\mathrm {dyn}}}{E}=\int \mathrm {d}x \mathrm {d}^2k\, x \frac{\mathrm {d}^3N^g}{\mathrm {d}x \mathrm {d}^2k} \end{aligned}$$

with the radiation spectrum

33 $$\begin{aligned} \frac{\mathrm {d}^3N^g}{\mathrm {d}x \mathrm {d}^2k}= & {} \frac{C_R \alpha _s}{\pi }\,\frac{L}{\lambda _\mathrm {dyn}} \int \frac{\mathrm {d}^2q}{x \pi ^2} \, v_\mathrm {dyn}(\vec {q})\nonumber \\&\left( 1-\frac{\sin {\left( \frac{(\vec {k}{+}\vec {q})^2+\chi }{x E^{+}} \, L\right) }}{\frac{(\vec {k}{+}\vec {q})^2+\chi }{x E^{+}}\, L}\right) \frac{2(\vec {k}{+}\vec {q})}{(\vec {k}{+}\vec {q})^2{+}\chi }\nonumber \\&\cdot \left( \frac{(\vec {k}{+}\vec {q})}{(\vec {k}{+}\vec {q})^2{+}\chi } - \frac{\vec {k}}{\vec {k}^2{+}\chi } \right) , \end{aligned}$$

where $\vec {q}$ and $\vec {k}$ are, respectively, the momentum of the radiated gluon and the momentum of the exchanged virtual gluon with a parton in the medium, with both $\vec {q}$ and $\vec {k}$ transverse to the jet direction. Here $\lambda _\mathrm {dyn}^{-1} \equiv C_2(G) \alpha _s T$ – where $C_2(G)=3$ is the gluon quadratic Casimir invariant – defines the “dynamical mean free path” [512], $\alpha _s$ is the strong-coupling constant, and $C_R=4/3$ is the Casimir factor. Further, $v_\mathrm {dyn}(\vec {q})=\frac{\mu _E^2}{\vec {q}^2 (\vec {q}^2{+}\mu _E^2)}$ is the effective potential. $\chi$ is defined as $m_Q^2 x^2 + m_g^2$, where $m_Q$ is the heavy-quark mass, *x* is the longitudinal momentum fraction of the heavy quark carried away by the emitted gluon and $m_g=\frac{\mu _E}{\sqrt{2}}$ is the effective mass for gluons with hard momenta $k\gtrsim T$ and $\mu _E$ is the Debye mass. It can be noted that the $C_R$ term encodes the colour charge dependence of the energy loss (for radiative energy loss off a gluon $C_R$ is three instead of 4 / 3). The $\chi$ term encodes the quark mass dependence of the energy loss, which is reduced for increasing values of $m_Q/(\vec {k}+\vec {q})$.

**Fig. 61:**
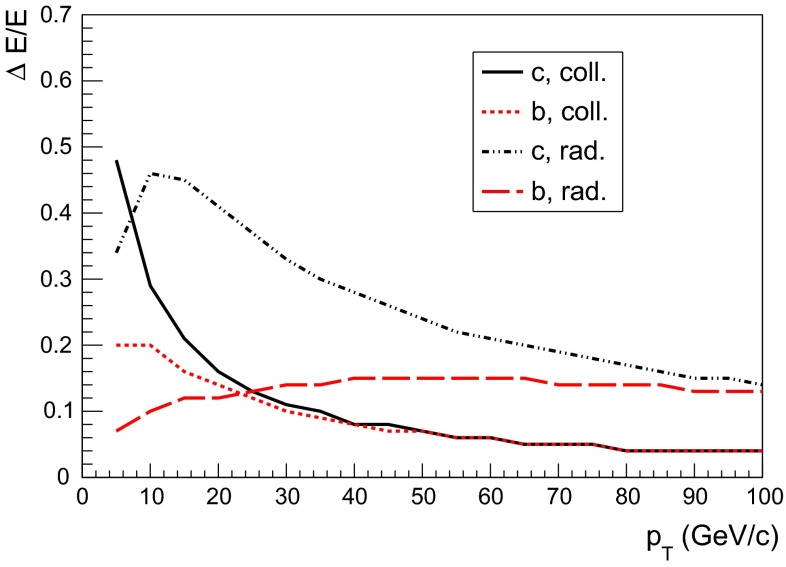
Fractional energy loss (Eq. (33)) evaluated for collisional and radiative processes and for charm and beauty quarks, at $T=304~\mathrm{MeV}$

Note that this dynamical energy loss presents an extension of the well-known static DGLV [457, 518] energy-loss formalism to the dynamical QCD medium. The connection between dynamical and static energy losses was discussed in Refs. [511, 512]. That is, static energy loss can be obtained from the above dynamical energy-loss expression by replacing the dynamical mean free path and effective potential by equivalent expressions for a static QCD medium: $v_\mathrm {dyn}(\vec {q})\rightarrow v_\mathrm {stat}(\vec {q})=\frac{\mu _E^2}{(\vec {q}^2{+}\mu _E^2)^2}$ and $\lambda _\mathrm {dyn}^{-1}\rightarrow \lambda _\mathrm {stat}^{-1}= 6 \frac{1.202}{\pi ^2} \frac{1{+}\frac{n_f}{4}}{1{+}\frac{n_f}{6}} \lambda _\mathrm {dyn}^{-1}$. Note that the static DGLV formalism was also used in the WHDG model [459, 519], as well as for the quark energy-loss calculation by Vitev et al. (see Sect. 4.3.3).

The dynamical energy-loss formalism was further extended to the case of finite magnetic mass, since various non-perturbative approaches suggest a non-zero magnetic mass at RHIC and LHC collision energies (see e.g.  Refs. [520–524]). The finite magnetic mass is introduced through generalised sum-rules [514]. The main effect of the inclusion of finite magnetic mass turns out to be the modification of effective cross section $$v_\mathrm {dyn}(\vec {q})$$ in Eq. (33) to $$v(\vec {q})=\frac{\mu _E^2-\mu _M^2}{(\vec {q}^2+\mu _M^2) (\vec {q}^2{+}\mu _E^2)}$$, where $$\mu _M$$ is the magnetic mass. In Fig. 61, the fractional energy loss $$\frac{\Delta E}{E}$$ corresponding to the full model described above is shown, for a path length $$L=5~\mathrm{fm}$$ and an effective constant temperature of $$T=304~\text {MeV} $$. For charm quarks, radiative energy loss starts to dominate for $$p_{\mathrm {T}} > 10$$$$~\text {GeV}/c$$, while this transition happens for $$p_{\mathrm {T}} >25$$$$~\text {GeV}/c$$ for beauty quark. The comparison of radiative energy loss for the two quark species clearly illustrates the dead cone effect, as well as its disappearance when $$p_{\mathrm {T}} \gg m_Q$$.

In [495], a calculation for the *b*-jet production in AA was performed following very similar ingredients for the energy loss. The medium-induced gluon spectrum in the soft gluon approximation was evaluated as in [392] in a medium which incorporates Glauber geometry and Bjorken expansion. With the QGP-induced distribution of gluons $\frac{\mathrm {d}^3 N^g}{\mathrm {d}x \mathrm {d}^2k}$ – of the type of Eq. (33) – and the related $\frac{\mathrm {d}^2 N^g}{\mathrm {d}\omega \mathrm {d}r}$ ($\omega$ is the energy and *r* is the angle) of gluons at hand, the fraction *f* of the in-medium parton shower energy that is contained in the jet cone of radius *R* was evaluated as

34 $$\begin{aligned} f(R,\omega ^\mathrm{coll})_{(s)}= \frac{\int _0^{R} \mathrm {d}r \int _{\omega ^\mathrm{coll}}^E \mathrm {d}\omega \, \frac{ \omega \mathrm {d}^2N^{g}_{(s)}}{\mathrm {d}\omega \mathrm {d}r }}{\int _0^{{R}^{\infty }} \mathrm {d}r \int _{0}^E \mathrm {d}\omega \, \frac{ \omega \mathrm {d}^2N^{g}_{(s)}}{\mathrm {d}\omega \mathrm {d}r} }. \end{aligned}$$

In Eq. (34) $$f(R,0)_{(s)} $$ takes into account medium-induced parton splitting effects. On the other hand $$f(R^\infty , \omega ^\mathrm{coll})_{(s)} = \Delta E^\mathrm{coll} / E $$ is the energy dissipated by the medium-induced parton shower into the QGP due to collisional processes. $$\Delta E^\mathrm{coll}$$ is evaluated as in Refs. [525, 526] and helps to solve for $$\omega ^\mathrm{coll}$$. Then, for any *R*, Eq. (34) allows one to treat the radiative and collisional energy-loss effects on the same footing. Writing down explicitly the phase-space Jacobian $$|J(\epsilon )|_{(s)} = 1/(1 - [1-f(R,\omega ^\mathrm{coll})_{(s)}] \epsilon )$$ for the case of *b*-jets the cross section per elementary nucleon–nucleon collision writes:

35 $$\begin{aligned}&\frac{1}{\langle N_\mathrm{bin} \rangle } \frac{\mathrm {d}^2 \sigma _{AA}^{b-\mathrm{jet}}(R)}{\mathrm {d}y \mathrm {d}p_{\mathrm {T}}}\!=\!\sum _{(s)} \int _{0}^1 \! \mathrm {d}\epsilon \; \frac{ P_{(s)}(\epsilon ) }{ \left( 1 \!-\! [1\!-\!f(R,\omega ^\mathrm{coll})_{(s)}] \epsilon \right) }\nonumber \\&\quad \times \frac{\mathrm {d}^2\sigma ^\mathrm{CNM,LO+PS}_{(s)} \left( |J(\epsilon )|_{(s)} p_{\mathrm {T}} \right) }{\mathrm {d}y \mathrm {d}p_{\mathrm {T}}}. \end{aligned}$$

Here, the sum runs over the set of final states (s). $\mathrm {d}^2\sigma ^\mathrm{CNM,LO+PS} / {\mathrm {d}y \mathrm {d}p_{\mathrm {T}}}$ includes cold nuclear matter effects.

The same group recently published predictions for photon-tagged and B-meson-tagged *b*-jet production at LHC [527].

#### A pQCD-inspired running $\alpha _s$ energy-loss model in MC$@_s$HQ and BAMPS

In the Monte Carlo at Heavy Quark approach [528–530] (MC$@_s$HQ), heavy quarks lose and gain energy by interacting with light partons from the medium (assumed to be in thermal equilibrium) according to rates which include both collisional and radiative types of processes.

For the collisional energy loss, the elements of the transition matrix are calculated from the pQCD Born approximation [369, 531], supplemented by a running coupling constant $\alpha _s(Q^2)$ evaluated according to 1-loop renormalisation for $|Q^2|\gg \Lambda _{QCD}^2$ and chosen to saturate at small $Q^2$ in order to satisfy the universality relation [532, 533]:

36 $$\begin{aligned} \alpha _s(Q^2)= \frac{4\pi }{\beta _0} {\left\{ \begin{array}{ll} L_-^{-1} &{} Q^2 < 0\\ \frac{1}{2} - \pi ^{-1} \mathrm{arctan}( L_+/\pi ) &{} Q^2 > 0 \end{array}\right. } \end{aligned}$$

with $$\beta _0 = 11-\frac{2}{3}\, n_f$$ and $$L_\pm = \ln (\pm Q^2/\Lambda ^2)$$ with $$\Lambda =200$$$$~\text {MeV}$$ and $$n_f = 3$$. The *t* channel requires infra-red regularisation which describes the physics of the screening at long distances [534]. For this purpose one adopts, in a first stage, a similar HTL polarisation as in the usual weak-coupling calculation of the energy loss [451, 452] for the small momentum-transfers, including the running $$\alpha _s$$ (Eq. (36)), while a semi-hard propagator is adopted for the large momentum-transfers. Then the model is simplified by resorting to an effective scalar propagator $$\frac{1}{t-\kappa \tilde{m}_D^2(T)}$$ for the exchanged thermal gluon, with a self-consistent Debye mass evaluated as $$\tilde{m}_D^2(T)= \frac{N_c}{3} (1+\frac{n_f}{6})4\pi \,\alpha _s(-\tilde{m}_D^2(T))\,T^2$$ [535] and an optimal value of $$\kappa $$ fixed to reproduce the value of the energy loss obtained at the first stage. The resulting model leads to a stronger coupling than previous calculations performed with fixed-order $$\alpha _s=0.3$$. It is also found to be compatible with the calculation of Ref. [536] – where the running of $$\alpha _s$$ is rigorously implemented – in the region where the latter is applicable.

A similar model is implemented in BAMPS [537–540], although with some variations. In BAMPS the Debye mass $m_D^2$ is calculated dynamically from the non-equilibrium distribution functions *f* of gluons and light quarks via [541] $m_D^2 = \pi \alpha _s \nu _g \int \frac{\mathrm {d}^3p}{(2\pi )^3} \frac{1}{p} ( N_c f_g + n_f f_q)$, where $N_c=3$ denotes the number of colours and $\nu _g = 16$ is the gluon degeneracy. While MC@$_s$HQ applies the equilibrium Debye mass with quantum statistics for temperatures extracted from the fluid dynamic background, BAMPS treats all particles as Boltzmann particles, due to the non-equilibrium nature of the cascade. Moreover, in BAMPS the scale of the running coupling in the Debye mass is evaluated at the momentum transfer of the process, e.g. $\alpha _s(t)$. The differences in the treatment lead to a larger energy loss of about a factor of two in MC@$_s$HQ compared to BAMPS.

As for the radiative energy loss, the model mostly concentrates on the case of intermediate energy for which coherence effects do not play the leading role. Exact momentum conservation and scattering on dynamical partons have, however, to be implemented exactly. In the MC@$_s$HQ approach [542, 543], the calculations of Ref. [544] are thus extended for incoherent radiation off a single massless parton to the case of massive quarks. For the central “plateau” of radiation, one obtains that the cross section $\mathrm {d}\sigma (Qq\rightarrow Qqg)$ is dominated by a gauge-invariant subclass of diagrams. It can be factorised as the product of the elastic cross section $\mathrm {d}\sigma (Qq\rightarrow Qq)$ and a factor $P_g$ representing the conditional probability of radiation per elastic collision, which is collinear-safe thanks to the heavy-quark mass $m_Q$. Moreover, it was shown in Ref. [543] that a fair agreement with the exact power spectra can be achieved by considering the eikonal limit in $P_g$ and preserving the phase-space condition. The ensuing relation reads $\mathrm {d}\sigma (Qq\rightarrow Qqg)=\mathrm {d}\sigma (Qq\rightarrow Qq) P_g^\mathrm{eik}$, with

37 $$\begin{aligned} P_g^\mathrm{eik}(x,\vec {k}_t,\vec {l}_t)= & {} \frac{3\alpha _s}{\pi ^2}\frac{1-x}{x}\nonumber \\&\times \bigg (\frac{\vec {k}_t}{k_t^2+x^2m_Q^2} - \frac{\vec {k}_t-\vec {l}_t}{(\vec {k}_t-\vec {l}_t)^2 +x^2 m_Q^2}\bigg )^2,\nonumber \\ \end{aligned}$$

where *x* is the fraction of four-momentum carried by the radiated gluon, $\vec {k}_t$ its transverse momentum and $\vec {l}_t$ the momentum exchanged with the light parton. For the radiation in a medium at finite temperature, the radiated gluon acquires a thermal mass, which leads to a modification $x^2 m_Q^2 \rightarrow x^2 m_Q^2 +(1-x) m_g^2$ in Eq. (37). As a consequence, the power spectra are vastly reduced. In MC@$_s$HQ, an explicit realisation of the elastic process is achieved first, and the radiation factor $P_g$ is then sampled along the variables *x* and $\vec {k}_t$. In Ref. [545], the implementation of radiative processes was generalised to include the coherent radiation, through an interpolation between single and multiple scatterings matched to the BDMPS result [546]. However it neglects the finite path length effects which are important for thin plasmas. Hereafter, this will be referred to as “LPM-radiative”. For further description of the model, the reader is referred to Ref. [530].

Similar considerations apply for radiative energy loss in BAMPS [547, 548]. Due to the semi-classical transport nature of BAMPS, the LPM effect is included effectively by comparing the formation time of the emitted gluon to the mean free path of the jet [549]. Furthermore, the emitted gluon is treated as a massless particle.

**Fig. 62:**
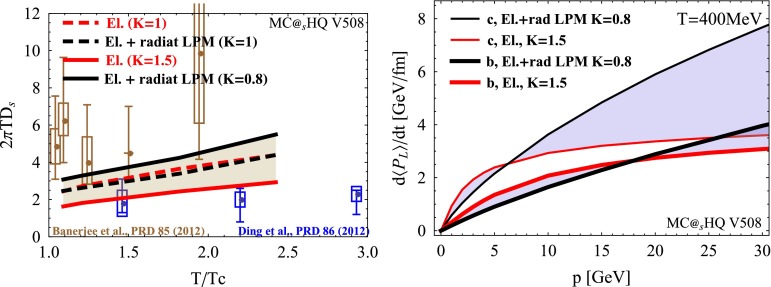
Macroscopic properties for both elastic and elastic plus LPM-radiative model. On the *left panel*, the diffusion coefficient $2\pi T D_s$ is plotted vs. $T/T_c$ and compared to the l-QCD calculations of [550, 551]. On the *right panel*, the average momentum loss per unit of time is plotted vs. heavy-quark momentum both for $c$ and $b$ quarks

Figure 62 illustrates two properties of this energy-loss model as implemented in MC@*s*HQ model. Both the pure elastic case and a combination of the elastic and LPM-radiative energy loss are considered. In both cases, the model is calibrated by applying a multiplicative *K*-factor to the interaction cross sections, in order to describe the $R_{\mathrm {AA}}$ of D mesons for intermediate $p_{\mathrm {T}}$ range in Pb–Pb collisions at $\sqrt{s_{\mathrm{NN}}} =2.76$ $\text {TeV}$ in the 0–20 % centrality class. This leads to $K_\mathrm{el}=1.5$ and $K_\mathrm{el+LPM-rad}=0.8$, while one obtains $K_\mathrm{el}=1.8$ and $K_\mathrm{el+LPM-rad}=1$ following a similar procedure at RHIC. For the spatial diffusion coefficient $D_s$, one sees that both combinations are compatible with the l-QCD calculations of Refs. [550, 551] and thus provide some systematic “error band” of the approach. The corresponding average momentum loss per unit of time (or length), shown on the right panel of Fig. 62, illustrates the mass-hierarchy, found to be stronger for the radiative component (black lines in the figure).

#### Collisional dissociation of heavy mesons and quarkonia in the QGP

Heavy flavour dynamics in dense QCD matter critically depends on the time scales involved in the underlying reaction. Two of these time scales, the formation time of the QGP $\tau _0$ and its transverse size $L_{QGP}$, can be related to the nuclear geometry, the QGP expansion, and the bulk particle properties. The formation time $\tau _\mathrm{form}$ of heavy mesons and quarkonia, on the other hand, can be evaluated from the virtuality of the heavy quark *Q* decay into D, B mesons [422, 460] or the time for the ${Q\overline{Q}}$ pair to expand to the size of the $\mathrm {J}/\psi$ or $\Upsilon$ wave function [83]. For a $\pi ^0$ with an energy of 10$~\text {GeV}$, $\tau _\mathrm{form} \sim 25$ fm $\gg L_{QGP}$ affords a relatively simple interpretation of light hadron quenching in terms of radiative and collisional parton-level energy loss [552]. On the other hand, for D, B, $\mathrm {J}/\psi$ and $\Upsilon (1\mathrm{S})$, one obtains $\tau _\mathrm{form} \sim$ 1.6, 0.4, 3.3 and 1.4 fm $\ll L_{QGP}$. Such short formation times necessitate understanding of heavy meson and quarkonium propagation and dissociation in strongly interactingmatter.

The Gulassy–Levai–Vitev (GLV) reaction operator formalism was developed for calculating the interactions of parton systems as they pass through a dense strongly interacting medium. It was generalised to the dissociation of mesons (quark-antiquark binaries), as long as the momentum exchanges from the medium $\mu = g T$ can resolve their internal structure. The dissociation probability and dissociation time

38 $$\begin{aligned}&P_d(p_{\mathrm {T}},m_Q,t) = 1 - \left| \int \mathrm {d}^{2}{\vec \Delta k} \mathrm {d}x \, \psi _{f}^* (\Delta {\vec k},x)\psi _{0}(\Delta {\vec k}, x) \right| ^{2}, \nonumber \\&\quad \frac{1}{\langle \tau _\mathrm{diss}(p_{\mathrm {T}}, t) \rangle } = \frac{\partial }{\partial t} \ln P_d(p_{\mathrm {T}},m_Q,t), \end{aligned}$$

can be obtained from the overlap between the medium-broadened time-evolved and vacuum initial meson wave functions, $\psi _f$ and $\psi _0$, respectively. Here, $\psi _f$ has the resummed collisional interactions in the QGP. Let us denote by

39 $$\begin{aligned}&f^{Q}({p}_{T},t)= \frac{\mathrm {d}\sigma ^Q(t)}{\mathrm {d}y \mathrm {d}^2p_{\mathrm {T}}}, \; f^{Q}({p}_{T},t=0) = \frac{\mathrm {d}\sigma ^Q_{PQCD}}{\mathrm {d}y \mathrm {d}^2p_{\mathrm {T}}}, \nonumber \\&f^{H}({p}_{T},t)= \frac{\mathrm {d}\sigma ^H(t)}{\mathrm {d}y\mathrm {d}^2p_{\mathrm {T}}}, \; f^{H}({p}_{T},t=0) = 0, \end{aligned}$$

the double differential production cross sections for the heavy quarks and hadrons. Initial conditions are also specified above, in particular the heavy quark distribution is given by the perturbative QCD $c$ and $b$ quark cross section. Energy loss in the partonic state can be implemented as quenched initial conditions [83, 422]. Including the loss and gain terms one obtains

40 $$\begin{aligned} \partial _t f^{Q}({p}_\mathrm{T},t)= & {} - \frac{1}{\langle \tau _\mathrm{form}(p_{\mathrm {T}}, t) \rangle } f^{Q}({p}_\mathrm{T},t)\nonumber \\&+ \, \frac{1}{\langle \tau _\mathrm{diss}(p_{\mathrm {T}}/\bar{x}, t) \rangle } \nonumber \\&\times \int _0^1 \mathrm {d}x \frac{1}{x^2} \phi _{Q/H}(x) f^{H}({p}_\mathrm{T}/x,t), \end{aligned}$$

41 $$\begin{aligned} \partial _t f^{H}({p}_\mathrm{T},t)= & {} - \frac{1}{\langle \tau _\mathrm{diss}(p_{\mathrm {T}}, t) \rangle } f^{H}({p}_\mathrm{T},t)\nonumber \\&+\, \frac{1}{\langle \tau _\mathrm{form}(p_{\mathrm {T}}/\bar{z}, t) \rangle } \nonumber \\&\times \int _0^1 \mathrm {d}z\frac{1}{z^2} D_{H/Q}(z) f^{Q}({p}_\mathrm{T}/z,t) . \end{aligned}$$

In Eqs. (40) and (41) $\phi _{Q/H}(x)$ and $D_{H/Q}(z)$ are the distribution function of heavy quarks in a heavy meson and the fragmentation function of a heavy quark into a heavy mesons, respectively, and $\bar{z}$ and $\bar{x}$ are typical fragmentation and dissociation momentum fractions. It was checked that in the absence of a medium, $\tau _\mathrm{diss}(p_{\mathrm {T}}, t) \rightarrow \infty$, so that the pQCD spectrum of heavy hadrons from vacuum jet fragmentation are recovered. Details for the rate equation relevant to quarkonium formation and dissociation are given in Ref. [83]. Solving the above equations in the limit $t \rightarrow \infty$ in the absence and presence of a medium allows one to evaluate the nuclear modification factor for heavy-flavour mesons.

**Fig. 63:**
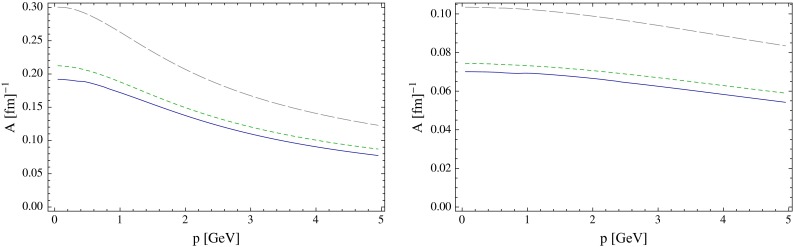
*Left* charm-quark friction coefficient, $A_c$, as a function of momentum in the QGP from non-pertubative *T*-matrix scattering amplitudes off thermal light and strange quarks [558], as well as gluons [564]; the *curves* correspond to temperatures $T=1.2, 1.5$ and 2 $T_\mathrm{c}$ (*bottom to top*); *Right* same as *left panel* but for bottom quarks. Figures are taken from Ref. [564]

#### *T*-matrix approach to heavy-quark interactions in the QGP

The thermodynamic *T*-matrix approach is a first-principles framework to self-consistently compute one- and two-body correlations in hot and dense matter. It has been widely applied to, e.g., electromagnetic plasmas [553] and the nuclear many-body problem [554, 555]. Its main assumption is that the basic two-body interaction can be cast into the form of a potential, *V*(*t*), with the four-momentum transfer approximated by $t=q^2=q_0^2-\vec q^{\,2}\simeq -\vec q^{\,2}$. This relation is satisfied for charm and beauty quarks ($Q = c,b$) in a QGP up to temperatures of 2–3 $T_\mathrm{c}$, since their large masses imply $q_0^2\simeq (\vec q^{\,2}/2m_Q)^2\ll \vec q^{\,2}$ with typical momentum transfers of $\vec q^{\, 2} \sim T^2$. Therefore, the *T*-matrix formalism is a promising framework to treat the non-perturbative physics of heavy-quark (HQ) interactions in the QGP [556–558]. It can be applied to both hidden and open heavy-flavour states, and it provides a comprehensive treatment of bound and scattering states [558]. It can be systematically constrained by lattice data [559], and implemented to calculate heavy-flavour observables in heavy-ion collisions [560, 561].

The potential approximation allows one to reduce the four-dimensional Bethe–Salpeter into a three-dimensional Lippmann–Schwinger equation, schematically given by

42 $$\begin{aligned} T(s,t) = V(t) + \int \mathrm {d}^3k \ V(t') \ G_2(s,k) \ T(s,t''), \end{aligned}$$

where $G_2$ denotes the in-medium 2-particle propagator. Using the well-known Cornell potential in vacuum [562], heavy quarkonium spectroscopy and heavy-light meson masses can be reproduced, while relativistic corrections (magnetic interactions) allow one to recover perturbative results in the high-energy limit for HQ scattering [558].

**Fig. 64:**
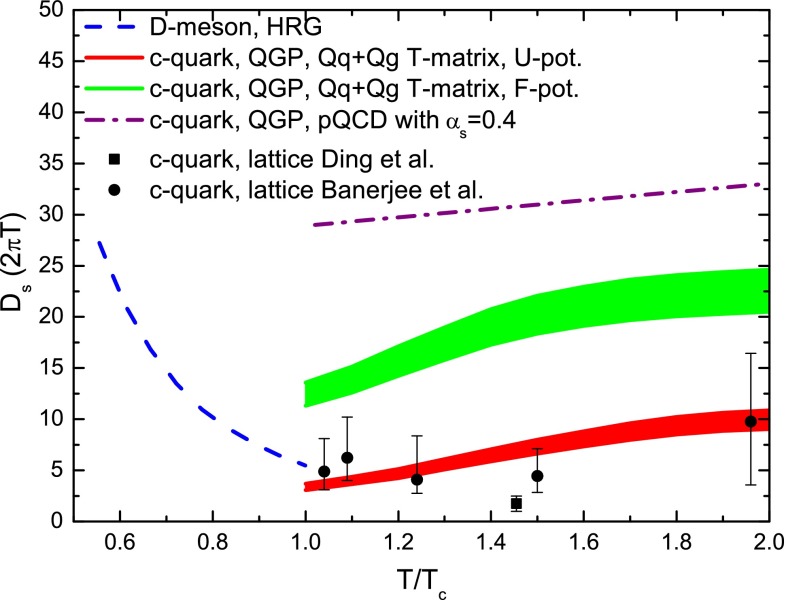
Spatial heavy-flavour diffusion coefficient (defined via the relaxation rate at zero momentum) for the *T*-matrix approach in the QGP using the *U* (*lower red band*) or *F* potential (*upper green band*) [558], or pQCD with $\alpha _s=0.4$ (*dash-dotted line*), in hadronic matter (*dashed line*) [565], and from quenched lattice QCD [551, 566] (*data points*); figure taken from [491]

The pertinent transport coefficients for a heavy quark of momentum $\vec p$ are given by

43 $$\begin{aligned} A_Q(p)= & {} \frac{1}{2\omega _Q(p)(2\pi )^9} \sum \limits _{j=q,\bar{q},g} \int \frac{\mathrm {d}^3k}{2\omega _k} \frac{\mathrm {d}^3k'}{2\omega _{k'}} \frac{\mathrm {d}^3p'}{2\omega _Q(p')} \nonumber \\&\times f^j(\omega _k)\delta ^{(4)}(P_i-P_f) \ |\mathcal{M}_{Qj}(s,t)|^2 \nonumber \\&\times \left( 1-\frac{\vec p \cdot \vec p\,'}{\vec p^{\,2}} \right) \end{aligned}$$

for the friction coefficient (or relaxation rate) and analogous expressions for momentum diffusion [466]. The invariant HQ-parton scattering amplitude, $\mathcal{M}_{Qj}$, is directly proportional to the *T*-matrix. An important ingredient is how the HQ potential *V* is modified in medium. This is currently an open question. As limiting cases the HQ free (*F*) and internal (*U*) energies computed in lattice-QCD (l-QCD) have been employed [563]. The internal energy produces a markedly stronger interaction, and, when employed in the *T*-matrix approach, generally leads to better agreement with other quantities computed on the lattice (e.g., quarkonium correlators, HQ susceptibility, etc. [559]). The resulting $c$-quark relaxation rates, including scattering off thermal $u$, $d$, $s$ quarks and gluons, are enhanced over their perturbatively calculated counterparts by up to a factor of $\sim$6 at low momenta and temperatures close to $T_\mathrm{c}$, cf. left panel of Fig. 63. A similar enhancement is found for $b$ quarks, although the absolute magnitude of the relaxation rate is smaller than for $c$ quarks by about a factor of $m_b/m_c\simeq 3$, cf. right panel of Fig. 63. The non-perturbative enhancement is mostly caused by resonant D/B-meson and di-quark states which emerge in the colour-singlet and anti-triplet channels as $T_\mathrm{c}$ is approached from above. These states naturally provide for HQ coalescence processes in the hadronisation transition, i.e., the same interaction that drives non-perturbative diffusion also induces hadron formation. The resummations in the *T*-matrix, together with the confining interaction in the potential, play a critical role in this framework. At high momenta, both confining and resummation effects become much weaker and the diffusion coefficients approach the perturbative (colour-Coulomb) results, although at $p\simeq 5$ GeV, the enhancement is still about a factor of 2 for charm quarks. With increasing temperature, the colour screening of the l-QCD-based interaction potentials leads to an increase in the (temperature-scaled) spatial diffusion coefficient, $D_s(2\pi T) = 2\pi T^2/(m_Q\,A_Q)$; see Fig. 64.

After coalescence into open-charm mesons, the approach also accounts for the diffusion of heavy-flavour mesons in the hadronic phase. Pertinent transport coefficients have been worked out in [565], based on effective D-meson scattering amplitudes off light hadrons as available from the literature. These include $\pi$, K, $\eta$, $\rho$, $\omega$, as well as nucleons and $\Delta$(1232) and their anti-particles. The combined effect of these scatterings is appreciable, leading to a hadronic diffusion coefficient comparable to the *T*-matrix calculations in the QGP close to $T_\mathrm{c}$. As first pointed out in [491, 565], this suggests a minimum of the (*T*-scaled) heavy-flavour diffusion coefficient via a smooth transition through the pseudo-critical region, as to be expected for a cross-over transition (see Fig. 64).

#### Lattice-QCD

First principle non-perturbative results for the transport coefficients can be obtained, although within a limited kinematic domain and with sizeable systematic uncertainties, from lattice QCD (l-QCD) calculations. The theoretical set-up employed to extract the momentum diffusion coefficient $\kappa$ [567] is described in the following. This approach is valid in the non-relativistic limit (for this calculation heavy quarks on the lattice are taken as static colour sources) where the transport of heavy quarks reduces to the Langevin equation

44 $$\begin{aligned} \frac{\mathrm {d}p^i}{\mathrm {d}t}=-\eta _D p^i+ \xi ^i(t), \end{aligned}$$

where $\eta _D$ and $\kappa$ are the friction and diffusion coefficients and where $\varvec{\xi }$ are stochastic forces autocorrelated according to $\langle \xi ^i(t)\xi ^j(t')\rangle \!=\!\delta ^{ij}\delta (t-t')\kappa$. Hence, in the $p\!\rightarrow \!0$ limit, $\kappa$ is given by the Fourier transform of the following force–force correlator

45 $$\begin{aligned} \kappa= & {} \frac{1}{3}\int _{-\infty }^{+\infty }\!\!\!\mathrm {d}t\langle \xi ^i(t)\xi ^i(0)\rangle _\mathrm{HQ} \approx \frac{1}{3}\int _{-\infty }^{+\infty }\!\!\!\mathrm {d}t{\langle F^i(t)F^i(0)\rangle _\mathrm{HQ}}\nonumber \\\equiv & {} \frac{1}{3}\int _{-\infty }^{+\infty }\!\!\!\mathrm {d}t D^>(t)=\frac{1}{3}D^>(\omega \!=\!0), \end{aligned}$$

where the expectation value is taken over an ensemble of states containing, besides thermal light partons, a static ($m_Q\!=\!\infty$) heavy quark. In a thermal bath, correlators are related by the Kubo–Martin–Schwinger condition, entailing for the spectral function the relation ${\sigma (\omega )}\!\equiv \!D^>(\omega )\!-\!D^<(\omega )={(1-e^{-\beta \omega })D^>(\omega )}$, so that

46 $$\begin{aligned} {\kappa }\equiv \lim _{\omega \rightarrow 0}\frac{{D^>(\omega )}}{3}=\lim _{\omega \rightarrow 0}\frac{1}{3}{\frac{\sigma (\omega )}{1-e^{-\beta \omega }}}\underset{\omega \rightarrow 0}{\sim }\frac{1}{3}\frac{T}{{\omega }}{\sigma (\omega )}. \end{aligned}$$

In the static limit magnetic effects vanish and the force felt by the heavy quark can only be due to the chromo-electric field

47 $$\begin{aligned} {\vec {F}}(t)=g\int \!\mathrm {d}{\vec {x}} \,Q^\dagger (t,{\vec {x}})t^aQ(t,{\vec {x}})\,{\vec {E}}^a(t,{\vec {x}}). \end{aligned}$$

Equation (46) shows how $\kappa$ depends on the low-frequency behaviour of the spectral density $\sigma (\omega )$ of the electric-field correlator in the presence of a heavy quark in the original thermal average in Eq. (45). The latter can be evaluated on the lattice for imaginary times $t\!=\!-i\tau$ [568]:

48 $$\begin{aligned} {D_E(\tau )}=-\frac{\langle \mathrm{Re\,Tr}[U(\beta ,\tau )gE^i(\tau ,\mathbf{0})U(\tau ,0)gE^i(0,\mathbf{0})]\rangle }{\langle \mathrm{Re\,Tr}[U(\beta ,0)]\rangle }.\nonumber \\ \end{aligned}$$

In the above equation the expectation value is now taken over a thermal ensemble of states of gluons and light quarks, with the Wilson lines $U(\tau _1,\tau _2)$ reflecting the presence of a static heavy quark. As it is always the case when attempting to get information on real-time quantities from l-QCD simulations, the major difficulty consists in reconstructing the spectral density $\sigma (\omega )$ from the inversion of

49 $$\begin{aligned} {D_E(\tau )}=\int _0^{+\infty }\frac{\mathrm {d}\omega }{2\pi }\frac{\cosh (\tau -\beta /2)}{\sinh (\beta \omega /2)}{\sigma (\omega )}, \end{aligned}$$

where one knows the correlator $D_E(\tau )$ for a limited set of times $\tau _i\!\in \!(0,\beta )$. Lattice results for the heavy quark diffusion coefficient are currently available for the case of a pure *SU*(3) gluon plasma [569, 570]. In transport calculations, depending on the temperature, one relied on the values $\kappa /T^3\!\equiv \!\overline{\kappa }\!\approx \!2.5-4$ obtained in Ref. [570], which the authors are currently trying to extrapolate to the continuum (i.e. zero lattice-spacing) limit.

Being derived in the static $m_Q\!=\!\infty$ limit and lacking any information on their possible momentum dependence, the above results for $\kappa$ have to be taken with some grain of salt when facing the present experimental data (mostly referring to charm at not so small $p_{\mathrm {T}}$); however, they could represent a really solid theoretical benchmark when beauty measurements, for which $M\!\gg \!T$, at low $p_{\mathrm {T}}$ will become available. Bearing in mind the above caveats and neglecting any possible momentum dependence of $\kappa$, the above l-QCD transport coefficients (the friction coefficient $\eta _D=\kappa /2ET$ being fixed by the Einstein relation) were implemented into POWLANG code [507] in order to provide predictions for D mesons, heavy-flavour electrons and $\mathrm {J}/\psi$ from B decays. One can estimate what the above results would entail for the average heavy-quark energy loss:

50 $$\begin{aligned}&\langle {\mathrm {d}E}/{\mathrm {d}x}\rangle =\langle {\mathrm {d}p}/{\mathrm {d}t}\rangle =-\eta _D\,p=-(\kappa /2ET)\nonumber \\&\quad p=-(\overline{\kappa }T^2/2)\,v. \end{aligned}$$

with *v*, the heavy-quark velocity. Numerically, this would imply a stopping power $$\langle -\mathrm {d}E/\mathrm {d}x\rangle \approx \overline{\kappa }\cdot 0.4\cdot v$$$$~\text {GeV}$$/fm at $$T=400$$$$~\text {MeV}$$ and $$\langle -\mathrm {d}E/\mathrm {d}x\rangle \approx \overline{\kappa }\cdot 0.1\cdot v$$$$~\text {GeV}$$/fm at $$T=200$$$$~\text {MeV}$$.

#### Heavy-flavour interaction with medium in AdS/CFT

The anti-de-Sitter/conformal field theory (AdS/CFT) correspondence [571, 572] is a conjectured dual between field theories in *n* dimensions and string theories in $n+1$ dimensions (times some compact manifold). The correspondence is best understood between $\mathcal {N} = 4$ super Yang–Mills (SYM) and Type IIB string theory; these two theories are generally considered exact duals of one another. The calculational advantage provided by the conjecture is that there is generally speaking an inverse relationship between the strength of the coupling in the dual theories: when the field theory is weakly coupled the string theory is strongly coupled, and vice versa. The advantage for QCD physics accessible at current colliders is clear: the temperatures reached at RHIC and LHC are at most a few times $\Lambda _{\mathrm {QCD}}$; it is therefore reasonable to expect that much of the dynamics in these collisions is dominated by QCD physics that is strongly coupled and hence theoretically accessible only via the lattice, which is then generally restricted to imaginary time correlators, or via the methods of AdS/CFT. The leading-order contribution to string theory calculations (corresponding to a very strong coupling limit in the field theory) comes from classical gravity; one uses the usual tools of Einsteinian General Relativity. Although much research is focussed on finding dual string theories ever closer to QCD, no one has yet found an exact dual; nevertheless, one hopes to gain non-trivial insight into QCD physics by investigating the relevant physics from known AdS/CFT duals. An obvious limitation of the use of AdS/CFT is that it is difficult to quantify the corrections one expects when going from the dual field theory in which a derivation is performed to actual QCD.

**Fig. 65:**
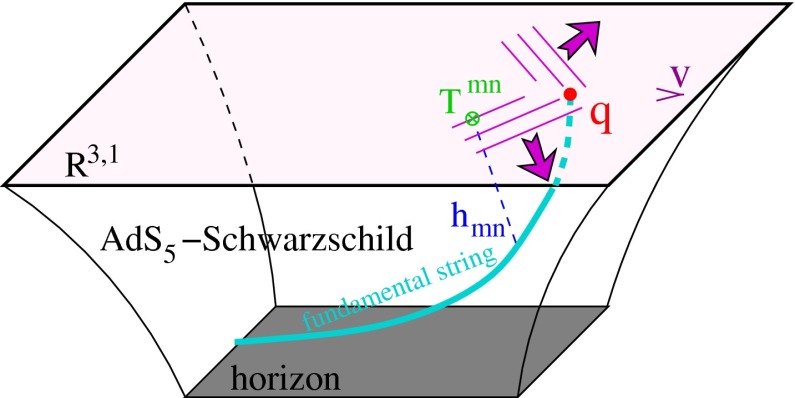
Schema for the heavy quark drag calculation [572]

The main thrust of open heavy flavour suppression research that uses the AdS/CFT correspondence assumes that all couplings are strong, regardless of scale (calculations for light quarks with all couplings assumed strong and calculations for which some couplings are strong and some are weak have also been performed; see [571, 572] and references therein for a review). For reasons soon to be seen, the result is known as “heavy quark drag”; see Fig. 65 for a picture of the set-up. The heavy quark is modelled as a string with one endpoint near (or, for an infinitely massive quark, at) the boundary of the AdS space; the string hangs down in the fifth dimension of the space-time towards a black hole horizon (the Hawking temperature of the black hole is equal to the temperature of the Yang–Mills plasma). As the string endpoint near the boundary moves, momentum flows down the string; this momentum is lost to the thermal plasma. For a heavy quark moving with constant velocity *v* in $\mathcal {N} = 4$ SYM, one finds [453, 454]

51 $$\begin{aligned} \frac{\mathrm {d}p}{\mathrm {d}t} = -\frac{\pi \sqrt{\lambda }}{2}T_{SYM}^2\frac{v}{\sqrt{1-v^2}} \;\; \Longrightarrow \;\; \frac{\mathrm {d}p}{\mathrm {d}t} = -\mu _Q\,p, \end{aligned}$$

where $\mu _Q = \pi \sqrt{\lambda }T_{SYM}^2/2m_Q$, and the energy loss reduces to a simple drag relationship in the limit of a very heavy quark, for which corrections to the usual dispersion relation $p/m_Q \, = \, v/\sqrt{1-v^2}$ are small.

As the string is dragged, an induced black hole horizon forms in the induced metric on the worldsheet of the string. This horizon emits Hawking radiation that is dual in the field theory to the influence of the thermal fluctuations of the plasma on the motion of the heavy quark. The diffusion coefficients have been derived in the large mass, constant motion limit [573, 574] as

52 $$\begin{aligned} \kappa _\mathrm{T} = \pi \sqrt{\lambda }\gamma ^{1/2}T_{SYM}^3,\quad \;\;\kappa _\mathrm{L} = \pi \sqrt{\lambda }\gamma ^{5/2}T_{SYM}^3. \end{aligned}$$

Note that for $v\,\ne \,0$ the above diffusion coefficients deviate from the usual ones found via the fluctuation–dissipation theorem, which, for the $\mu _Q$ of Eq. (51), yields $\kappa _\mathrm{T}\,=\,\kappa _\mathrm{L}\,=\,\pi \sqrt{\lambda }\gamma \, T_{SYM}$. The entire set-up breaks down at a characteristic “speed limit,” $\gamma _{\mathrm{crit}}^{SL} = (1 + 2 \, m_Q / \sqrt{\lambda }\,T_{SYM})^2 \, \approx 4\,m_Q^2 / \lambda \, T^2$, which corresponds to the velocity at which the induced horizon on the worldsheet moves above the string endpoint (equivalently, if an electric field maintains the constant velocity of the heavy quark, at the critical velocity the field strength is so large that it begins to pair-produce heavy quarks) [573]. Above the critical velocity, it no longer makes sense to treat the heavy quark as heavy, and one must resort to light-flavour energy-loss methods in AdS/CFT.

### Theoretical overview: medium modelling and medium-induced modification of heavy-flavour production

Besides modelling the energy loss as described in Sect. 4.3, each model aiming at explaining open heavy flavour observables in AA collisions needs to include several key ingredients. These are: the “initial” production of heavy flavour (see Sect. 2.1.1) possibly affected by cold nuclear matter effects (see Sect. 3), a space-time description of the QGP evolution up to the freeze-out, mechanisms for hadronisation (including specific processes like the so-called “coalescence”) and, ultimately, D-meson and B-meson interactions in the ensuing hadronic matter. For a given energy-loss model, it has been shown that various choices of these auxiliary ingredients could generate a factor of 2 in the observables [575]. In this section, the solutions adopted in the various models are described in order to better understand their predictions for the modification of heavy-flavour production in AA.

#### pQCD energy loss in a static fireball (Djordjevic et al.)

The dynamical energy-loss formalism discussed in Sect. 4.3.1 (Djordjevic et al.) was incorporated by the same authors into a numerical procedure in order to calculate medium-modified heavy-flavour hadron momentum distributions. This procedure includes (i) production of light and heavy-flavour partons based on the non-zero-mass variable flavour number scheme VFNS [391], NLO [422] and FONLL calculations [44], respectively, (ii) multi-gluon [576] and path-length [519, 577] fluctuations, (iii) light [578] and heavy [579–581] flavour fragmentation functions, and (iv) decay of heavy-flavour mesons to single electrons/muons and $\mathrm {J}/\psi$  [44]. This model does not include hadronisation via recombination. In-medium path length is sampled from a distribution corresponding to a static fireball at fixed effective temperature. The $R_{\mathrm {AA}}$ predictions are provided for both RHIC and LHC energies, various light and heavy-flavour probes and different collision centralities. This model does not include any free parameter. All implementation details are provided in Ref. [582]. A representative set of these predictions will be presented in Sect. 4.5, while other predictions and detailed comparison with experimental data are provided in Ref. [515, 582, 583]. In summary, this formalism provides a robust agreement with experimental data, across diverse probes, experiments and experimental conditions.

#### pQCD embedded in viscous hydro (POWLANG and Duke)

The starting point of the POWLANG set-up [507] is the generation of the ${Q\overline{Q}}$ pairs in elementary nucleon–nucleon collisions. For this purpose the POWHEG-BOX package [48, 152] is employed: the latter deals with the initial production in the hard pQCD event (evaluated at NLO accuracy), interfacing it to PYTHIA 6.4 [151] to simulate the associated initial and final-state radiation and other effects, like e.g. the intrinsic-$k_{\mathrm {T}}$ broadening. In the AA case, EPS09 nuclear corrections [364] are applied to the PDFs and the ${Q\overline{Q}}$ pairs are distributed in the transverse plane according to the local density of binary collisions given by the geometric Glauber model. Furthermore, a further $k_{\mathrm {T}}$ broadening depending on the crossed nuclear matter thickness is introduced, as described in Ref. [508]. Both in the $\mathrm pp$ benchmark and in the AA case (at the decoupling from the fireball) hadronisation is modelled through independent fragmentation of the heavy quarks, with in-vacuum fragmentation functions tuned by the FONLL authors [431]. Concerning the modelling of the fireball evolution, the latter is taken from the output of the $(2+1)$d viscous fluid dynamics code of Ref. [584]. At each time step, the update of the heavy-quark momentum according to the Langevin equation is performed in the local rest frame of the fluid, boosting then back the results into the laboratory frame. In setting the transport coefficients entering into the Langevin equation, the approach adopted in Ref. [507] was to derive the momentum broadening $\kappa _\mathrm{T/L}(p)$ and to fix the friction coefficient $\eta _D(p)$ so to satisfy the Einstein relation. For the former, two different sets of values were explored: the ones from a weak-coupling calculations described in Sect. 4.3.1 and the ones provided by the lattice QCD calculations described in Sect. 4.3.5. The local character of these energy-loss models indeed allows for their implementation with fluid dynamics as a background.

In first phenomenological studies performed with the POWLANG set-up [507, 508] hadronisation was modelled as occurring in the vacuum, neglecting the possibility of recombination of the heavy quarks with light thermal partons from the medium. Hence no modification of the heavy flavour spectra or hadrochemistry at hadronisation was considered, charm and beauty going into hadrons with the same fragmentation fractions as in the vacuum. A medium-modified hadronisation scheme has been recently developed in Refs. [585, 586]. Note that the recombination with light thermal quarks would make the final charmed hadrons inherit part of the flow of the medium, moving present POWLANG results closer to the experimental data. First numerical results [585, 586] show that this is actually the case, in particular for what concerns the elliptic flow of D mesons at LHC and their $R_{\mathrm {AA}}$ at low $p_{\mathrm {T}}$ at RHIC.

In the Duke model [587], the Langevin approach was generalised by Cao, Qin and Bass in order to include the contribution of radiative energy loss, thus offering a complementary perspective both with respect to the approach of Djordjevic (where static medium is considered) and to POWLANG (where no radiative energy loss is implemented). The generalised Langevin equation reads

53 $$\begin{aligned} \frac{\mathrm {d}\vec {p}}{\mathrm {d}t}=-\eta _D(p) \vec {p} +\vec {\xi } + \vec {f}_g \end{aligned}$$

where $\vec {f}_g$ is the semi-classical recoil force exerted on heavy quark due to medium-induced gluon radiation. In a (Ito)-discretised scheme, the associated recoil momentum $\Delta \vec {p}_g$ is obtain, at each time step $\Delta t$, by sampling the radiated gluon radiation spectrum $\frac{\mathrm {d}N_g}{\mathrm {d}x \mathrm {d}k_\perp ^2 \mathrm {d}t}$, with an absolute probability of radiation $P_\mathrm{rad}=\int _t^{t+ \Delta t} \mathrm {d}t \int \mathrm {d}x \mathrm {d}k_\perp ^2 \frac{\mathrm {d}N_g}{\mathrm {d}x \mathrm {d}k_\perp ^2 \mathrm {d}t}$. In [587], the “usual” stochastic forces $\vec {\xi }$ associated to the collisional processes are chosen to be autocorrelated in time according to $\langle \xi ^i(t) \xi ^j(t')\rangle =\kappa \delta ^{i j} \delta (t-t')$, with a spatial diffusion coefficient $D_s=\frac{2 T^2}{\kappa }$ set to $\frac{6}{2\pi T}$, while the gluon radiation spectrum is computed with the pQCD higher-twist approach [458].

The space-time evolution of the temperature and collective flow profiles of the thermalised medium are described with a $(2+1)$d viscous fluid dynamics [588–590]. At the end of the QGP phase, the hadronisation of heavy quarks is modelled with a hybrid fragmentation plus recombination scenario. Fragmentation processes are simulated by PYTHIA 6.4 [151] while the heavy quark coalescence with light quarks is treated with the “sudden recombination” approach developed in [591].

#### pQCD-inspired energy loss with running $\alpha _s$ in a fluid-dynamical medium and in Boltzmann transport

The implementation of the microscopic models based on a running coupling constant – described in Sect. 4.3.2 – in the MC@$$_s$$HQ and BAMPS frameworks is presented here. In its latest version, MC@$$_s$$HQ couples a Boltzmann transport of heavy quarks to the ($$3+1$$)d ideal-fluid-dynamical evolution from EPOS2 initial conditions. In its integral version, which includes the hadronic final-state interactions, the EPOS2 model describes very well a large variety of observables measured in the light-flavour sector in nucleus–nucleus, proton–nucleus and proton–proton collisions at RHIC and LHC [264, 592–594]. Therefore, it provides as a reliable description of the medium from which the thermal scattering partners of the heavy quarks are sampled. Due to the fluctuating initial conditions of the fluid dynamics, the heavy-quark evolution can be treated in an event-by-event set-up. Initially, the heavy quarks are produced at the spatial scattering points of the incoming nucleons with a momentum distribution from either FONLL [41, 44, 595] or MC@NLO [47, 153]. The latter combines next-to-leading order pQCD cross sections with a parton shower evolution, which provides more realistic distributions for the initial correlations of heavy quark–antiquark pairs than the back-to-back initialisation applied to single inclusive spectra obtained with FONLL. In recent implementations [596], a convolution of the initial $$p_{\mathrm {T}}$$ spectrum was applied in order to include (anti-)shadowing at (high) low $$p_{\mathrm {T}}$$ in central collisions at the LHC according to the EPS09 nuclear shadowing effect [364]. After propagation in the deconfined medium heavy quarks hadronise at a transition temperature of $$T=155$$$$~\text {MeV}$$, which is well in the range of transition temperatures given by lattice QCD calculations [597]. As described in [529], hadronisation of heavy quarks into D and B mesons can proceed through coalescence (predominant at low $$p_{\mathrm {T}}$$) or fragmentation (predominant at large $$p_{\mathrm {T}}$$). Recently, MC@$$_s$$HQ$$+$$EPOS2 has also been used to study heavy-flavour correlation observables [530] (see Sect. 4.6) and higher-order flow coefficients [598].

In the BAMPS model [541, 599], the initial heavy quark distribution is obtained from MC@NLO [47, 153] for $$\mathrm pp$$ collisions through scaling with the number of binary collisions to heavy-ion collisions without taking cold nuclear matter effects into account. After the QGP evolution (that is, after the local energy density has fallen below $$\epsilon = 0.6$$$$~\text {GeV}$$/fm$$^3$$) heavy quarks are fragmented via Peterson fragmentation [600] to D and B mesons. Recombination processes are not considered for the hadronisation.

RHIC heavy-flavour decay-electron data can be reproduced with only collisional interactions if their cross section is increased by a *K*-factor of 3.5 [601]. With this parameter fixed, BAMPS predictions [601] for $v_{2}$ at LHC for various heavy-flavour particles can describe the data, but the $R_{\mathrm {AA}}$ is slightly underestimated. However, the need of the phenomenological *K*-factor is rather unsatisfying from the theory perspective, especially if *K* is found to deviate vastly from unity. Therefore, radiative processes were recently included in BAMPS [540] and the *K*-factor mocking higher-order effects abandoned.^21^ The ensuing predictions are in satisfactory agreement with the data, which seems to favour this recent development of the BAMPS model.

#### Non-perturbative *T*-matrix approach in a fluid-dynamic model (TAMU) and in UrQMD transport

The *T*-matrix approach for heavy-flavour diffusion through QGP, hadronisation and hadronic matter [558, 565] described in Sect. 4.3.4 has been implemented into a fluid-dynamic background medium [561]. The latter is based on the ($2+1$)-dimensional ideal-fluid dynamics code of Kolb and Heinz [602], but several amendments have been implemented to allow for an improved description of bulk-hadron observables at RHIC and LHC [603]. First, the quasi-particle QGP equation of state (EoS) with first-order transition has been replaced by a lattice-QCD EoS which allows for a near-smooth matching into the hadron-resonance gas. Second, the initialisation at the thermalisation time has been augmented to account for a non-trivial flow field, in particular a significant radial flow [604]. Third, the initial energy-density profile has been chosen in more compact form, close to a collision profile that turns out to resemble initial states from saturation models. All three amendments generate a more violent transverse expansion of the medium, which, e.g., have been identified as important ingredients to solve the discrepancy between the fluid dynamics predictions and the measured HBT radii at RHIC (the so-called HBT puzzle [605]). These features furthermore lead to an “early” saturation of the bulk-medium $v_{2}$  [603], close to the phase-transition region. Consequently, multi-strange hadrons ($\phi$, $\Xi$ and $\Omega ^{-}$) need to freeze out at this point to properly describe their $p_{\mathrm {T}}$ spectra and $v_{2}$. This provides a natural explanation of the phenomenologically well-established universal kinetic-energy scaling of hadron $v_{2}$ at RHIC. For the medium evolution at LHC, an initial radial flow is phenomenologically less compelling, and has not been included in the tune of fluid dynamics for Pb–Pb collisions at $\sqrt{s_{\mathrm{NN}}} =2.76$ $\text {TeV}$, while the thermalisation time ($\tau _0=0.4$ fm/*c*) is assumed to be shorter than at RHIC (0.6 fm/*c*). Representative bulk-hadron observables at LHC are reasonably well described as a function of both $p_{\mathrm {T}}$ and centrality [606].

Heavy-flavour diffusion is implemented into the fluid-dynamical medium employing relativistic Langevin simulations of the Fokker–Planck equation. The pertinent non-perturbative transport coefficients from the heavy-light *T*-matrix in the QGP and effective hadronic theory for D mesons in the hadronic phase are utilised in the local rest frame of the expanding medium. The initial heavy-quark momentum distributions are taken from FONLL pQCD calculations [595], which describe $$\mathrm pp$$ spectra with suitable fragmentation functions. After diffusion through the QGP the HQ distributions are converted into D/D$$^*$$ mesons using the resonance recombination model (RRM) [607] with $$p_{\mathrm {T}}$$-dependent formation probabilities from the heavy-light *T*-matrices in the colour-singlet channels. The hadronisation is carried out on the hyper-surface corresponding to $$T_\mathrm{pc}=170$$$$~\text {MeV}$$. The heavy quarks that do not recombine are hadronised via the same fragmentation function as used in $$\mathrm pp$$ collisions. The resulting D-meson distributions are further evolved through the hadronic phase until kinetic freeze-out of the fluid-dynamical medium. However, the distributions of $$\mathrm {D}^{+}_{s} =(c{\overline{s}})$$ mesons, which do not contain any light quarks, are frozen out right after hadronisation, in line with the early freeze-out of multi-strange mesons.

In recent years, another model [608–610] implementing the non-perturbative *T*-matrix approach described in Sect. 4.3.4 has been put forward. It was motivated by the necessity of a realistic description for the bulk evolution of the fireball created in ultra-relativistic heavy-ion collisions. For this purpose, a transport fluid-dynamics hybrid model of the bulk has been developed [611]. It combines the Ultra-relativistic Quantum Molecular Dynamics (UrQMD) to describe the initial and final stages and ideal-fluid dynamics for the intermediate stage of the evolution. In this model, the initial collision of the two nuclei is simulated with the UrQMD cascade model [612, 613]. After a time $t_{\text {start}}=2 R/\sqrt{\gamma _{\text {cm}}^2-1}$, (*R*: radius of the colliding nuclei), when the Lorentz-contracted nuclei have passed through each other ($\gamma _{\text {cm}}$: the Lorentz-contraction factor in the centre-of-mass frame) the evolution is switched to a relativistic ideal-fluid simulation using the full ($3+1$)-dimensional SHASTA algorithm [614–616] by mapping the energy, baryon number, and momenta of all particles within UrQMD onto a spatial grid. Thermal freeze-out is assumed to occur approximately on equal proper-time hyper-surfaces and performed in terms of the usual Cooper–Frye prescription [617].

The diffusion of heavy quarks is described during the fluid-dynamics stage of the simulation using a Fokker–Planck description [531, 560, 618–623] (“Brownian motion”) employing a Monte-Carlo implementation of the relativistic Langevin approach, with quark-*Q* (light-quark–heavy-quark) drag and diffusion coefficients calculated as explained in Sect. 4.3.4.^22^ The elastic gluon–*Q* interaction is computed using a leading-order pQCD cross section [369] with a Debye screening mass of $m_{Dg}=g T$ in the gluon propagators, which regularises the *t*-channel singularities in the matrix elements. The strong coupling constant is set to the constant value $\alpha _s=g^2/(4 \pi )=0.4$.

Heavy-quark production is evaluated perturbatively on the time-dependent background by UrQMD/hybrid. A first UrQMD run is used to determine the collision coordinates of the nucleons within the nuclei according to a Glauber initial-state geometry. The corresponding space-time coordinates are saved and used in a second full UrQMD run as possible production coordinates for the heavy quarks. The initial $$p_{\mathrm {T}}$$ distributions of heavy quarks at $$\sqrt{s} =200$$$$~\text {GeV}$$ is an ad hoc parametrisation, such that the decay-electron $$p_{\mathrm {T}}$$ distribution from the calculation describes the distribution measured in pp collisions at RHIC [560, 622]. For LHC energy, heavy-quark $$p_{\mathrm {T}}$$ distributions obtained from the PYTHIA event generator are used. Finally, at freeze-out temperature the heavy quarks decouple and are hadronised either via Peterson [600] fragmentation or coalescence.

#### Lattice-QCD embedded in viscous fluid dynamics (POWLANG)

In the POWLANG framework (see Sect. 4.4.3) a set of diffusion coefficients $\kappa$ provided by the lattice QCD calculations and described in Sect. 4.3.5 was also implemented.

The main limitation of the lattice QCD approach, providing in principle a non-perturbative result, is the absence of any information on the momentum dependence of $\kappa$. The authors of POWLANG make the choice of keeping $\kappa$ constant. On the contrary, in the weak-coupling pQCD calculation the longitudinal momentum broadening coefficient $\kappa _\mathrm{L}(p)$, although starting from a much lower value than the l-QCD one, displays a steep rise with the heavy-quark momentum, which for high enough energy makes it overshoot the lattice-QCD result, taken as constant. Experimental data on the $R_{\mathrm {AA}}$ of D mesons and heavy-flavour decay electrons seem to favour an intermediate scenario.

#### AdS/CFT calculations in a static fireball

In the model of Refs. [624, 625], FONLL [44] provides both the heavy-flavour production and the fragmentation to D and B mesons. The medium is described with a static fireball with a transverse profile $$T(\vec {x},\,\tau )\propto \rho _{part}(\vec {x})\tau ^{-1/3}$$ based on the Glauber model. The energy loss of a heavy quark propagating through the plasma is then given by the AdS/CFT drag derivation, Eq. (51), starting at an early thermalisation time $$\tau _0 = 0.6$$ fm and continuing until $$T = T_{\mathrm{hadronisation}} = 160$$$$~\text {MeV}$$. Path lengths are sampled through a participant transverse density distribution taking into account the nuclear diffuseness.

It is non-trivial to connect the parameters of QCD to those of the SYM theory in which the AdS/CFT derivations were performed. Two common prescriptions [573] for determining the parameters in the SYM theory are to take: (i) $\alpha _{SYM} = \alpha _s$ and $T_{SYM} = T_{QCD}$ or (ii) $\lambda _{SYM} = 5.5$ and $e_{SYM} = e_{QCD}$ (and hence $T_{SYM} = T_{QCD} / 3^{1/4}$). In the first prescription, the SYM coupling is taken equal to the QCD coupling and the temperatures are equated. In the second prescription, the energy densities of QCD and SYM are equated and the coupling is fitted by comparing the static quark–antiquark force from AdS/CFT to lattice results.

Comparing with RHIC data, the pure drag energy loss is qualitatively consistent with electrons from the semi-leptonic decays of heavy mesons [420, 469]. In the RHIC calculation, the proportionality constant between the medium temperature and the Glauber participant density is set in such a way that, in the Stefan–Boltzmann limit, the rapidity density of gluons in the medium is $\mathrm {d}N_g/\mathrm {d}y = 1000$, which is similar to that required by perturbative energy-loss calculations and is not too different from the entropy one expects from the measured hadronic multiplicity [519]. With that proportionality constant fixed, predictions for the suppression at LHC are performed assuming that the temperature of the medium scales with the measured hadronic multiplicity [626].

**Table 11:** Comparative overview of the models for heavy-quark energy loss or transport in the medium described in the previous sections

Model	Heavy-quark production	Medium modelling	Quark–medium interactions	Heavy-quark hadronisation	Tuning of medium coupling (or density) parameter(s)
Djordjevic et al. [511–515]	FONLL no PDF shadowing	Glauber model nuclear overlap no fl. dyn. evolution	Rad. + coll. energy loss finite magnetic mass	Fragmentation	Medium temperature fixed separately at RHIC and LHC
WHDG [459, 519]	FONLL no PDF shadowing	Glauber model nuclear overlap no fl. dyn. evolution	Rad. + coll. energy loss	Fragmentation	RHIC (then scaled with $\mathrm{d}N_\mathrm{ch}/\mathrm{d}\eta$)
Vitev et al. [422, 460]	Non-zero-mass VFNS no PDF shadowing	Glauber model nuclear overlap ideal fl. dyn. $1+1$d Bjorken expansion	Radiative energy loss in-medium meson dissociation	Fragmentation	RHIC (then scaled with $\mathrm{d}N_\mathrm{ch}/\mathrm{d}\eta$)
AdS/CFT (HG) [624, 625]	FONLL no PDF shadowing	Glauber model nuclear overlap no fl. dyn. evolution	AdS/CFT drag	Fragmentation	RHIC (then scaled with $\mathrm{d}N_\mathrm{ch}/\mathrm{d}\eta$)
POWLANG [507–509, 585, 586]	POWHEG (NLO) EPS09 (NLO) PDF shadowing	$2+1$d expansion with viscous fl. dyn. evolution	Transport with Langevin eq. collisional energy loss	Fragmentation recombination	Assume pQCD (or l-QCD *U* potential)
MC@$_s$HQ+EPOS2[528–530]	FONLL EPS09 (LO) PDF shadowing	$3+1$d expansion (EPOS model)	Transport with Boltzmann eq. rad. $+$ coll. energy loss	Fragmentation recombination	QGP transport coefficient fixed at LHC, slightly adapted for RHIC
BAMPS [537–540]	MC@NLO no PDF shadowing	$3+1$d expansion parton cascade	Transport with Boltzmann eq. rad. + coll. energy loss	Fragmentation	RHIC (then scaled with $\mathrm{d}N_\mathrm{ch}/\mathrm{d}\eta$)
TAMU [491, 565, 606]	FONLL EPS09 (NLO) PDF shadowing	2$+$1d expansion ideal fl. dyn.	Transport with Langevin eq. collisional energy loss diffusion in hadronic phase	Fragmentation recombination	Assume l-QCD *U* potential
UrQMD [608–610]	PYTHIA no PDF shadowing	3$+$1d expansion ideal fl. dyn.	Transport with Langevin eq. collisional energy loss	fragmentation recombination	Assume l-QCD *U* potential
Duke [587, 628]	PYTHIA EPS09 (LO) PDF shadowing	2$+$1d expansion viscous fl. dyn.	Transport with Langevin eq. rad. + coll. energy loss	Fragmentation recombination	QGP transport coefficient fixed at RHIC and LHC (same value)

The kinematic range of applicability of the model can be estimated through the $p_{\mathrm {T}}$ scale at which including momentum fluctuations becomes important. By comparing the momentum lost to drag to the potential momentum gain of the fluctuations, one expects that momentum fluctuations become important at a scale $\gamma _{\mathrm{crit}}^{\Delta p^2} = m_Q^2 / 4 \, T^2$. One can see from above that the speed limit at which the entire calculational framework breaks down, $\gamma _{\mathrm{crit}}^{SL}$, is parametrically in $\lambda$ smaller than $\gamma _{\mathrm{crit}}^{\Delta p^2}$; however, numerically for the finite values of $\lambda$ phenomenologically relevant at RHIC and LHC $\gamma _{\mathrm{crit}}^{\Delta p^2} < \gamma _{\mathrm{crit}}^{SL}$. In particular, one expects non-trivial corrections to the drag results for $e^\pm$ and D mesons from open heavy flavour for $p_{\mathrm {T}} <4$–5$~\text {GeV}/c$. Other calculations [621, 627] have attempted to include the effect of fluctuations; however, their diffusion coefficients were set by the Einstein relations and not those derived from AdS/CFT (recall that the derived diffusion coefficients are qualitatively different from those based on the fluctuation–dissipation theorem except in the limit of $v = 0$).

### Comparative overview of model features and comparison with data

The theoretical models described in the previous sections are compared in Table 11 in terms of their “ingredients” for heavy-quark production, medium modelling, quark–medium interactions, and heavy-quark hadronisation.

In this section we compile a comparison of model calculations with heavy-flavour $R_{\mathrm {AA}}$ and $v_{2}$ measurements by the RHIC and LHC experiments.

**Fig. 66:**
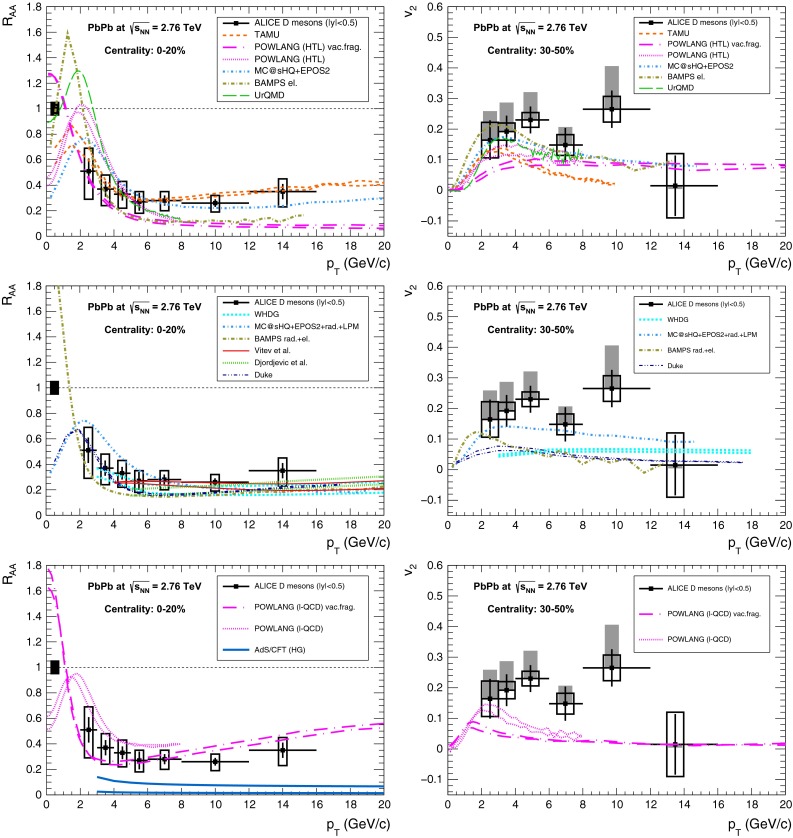
*Left* nuclear modification factor as a function of transverse momentum of averaged prompt D mesons in the 0–20 % most central Pb–Pb collisions at $\sqrt{s_{\mathrm{NN}}}$ $=$ 2.76 TeV [477] (the *filled box* at $R_{\mathrm {AA}} =1$ is the systematic uncertainty on the normalisation). *Right* $v_{2}$ as a function of transverse momentum of D mesons in the 30–50 % centrality Pb–Pb collisions at $\sqrt{s_{\mathrm{NN}}}$ $=$ 2.76 TeV [479] (the *filled boxes* are the systematic uncertainties on the feed-down subtraction). The results are obtained as an average of the $\mathrm {D}^{0}$, $\mathrm {D}^{+}$ and $\mathrm {D}^{*+}$ measurements. The results are compared to model calculations implementing collisional energy loss (*top panels*), collisional and radiative energy loss (*middle panels*) and to models which cannot be ascribed to the previous categories (*bottom panels*)

**Fig. 67:**
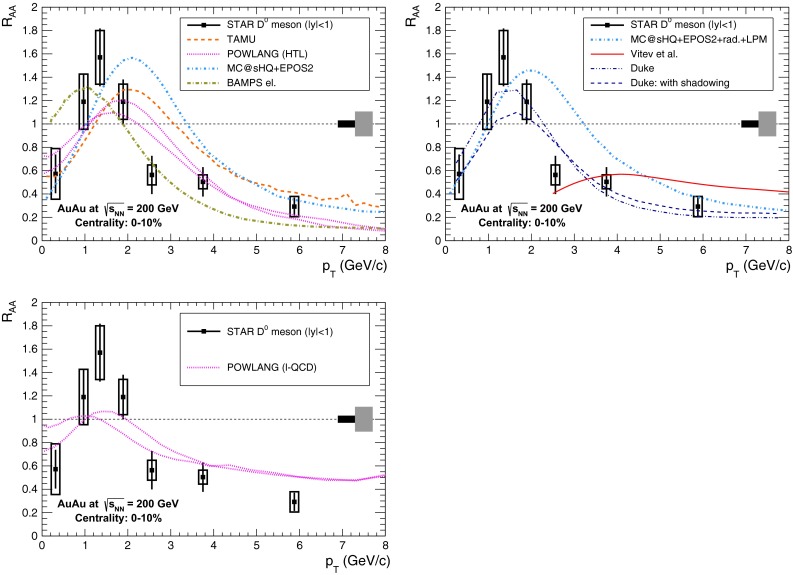
Nuclear modification factor as a function of transverse momentum of $\mathrm {D}^{0}$ mesons in the 0–10 % most central Au–Au collisions at $\sqrt{s_{\mathrm{NN}}}$ $=$ 200 GeV [475]. The *filled boxes* at $R_{\mathrm {AA}} =1$ are, from *left* to *right*, the systematic uncertainties on the normalisation of Au–Au and $\mathrm pp$ data. The results are compared to model calculations implementing collisional energy loss (*top left*), collisional and radiative energy loss (*top right*) and to models which cannot be ascribed to the previous categories (*bottom left*)

**Fig. 68:**
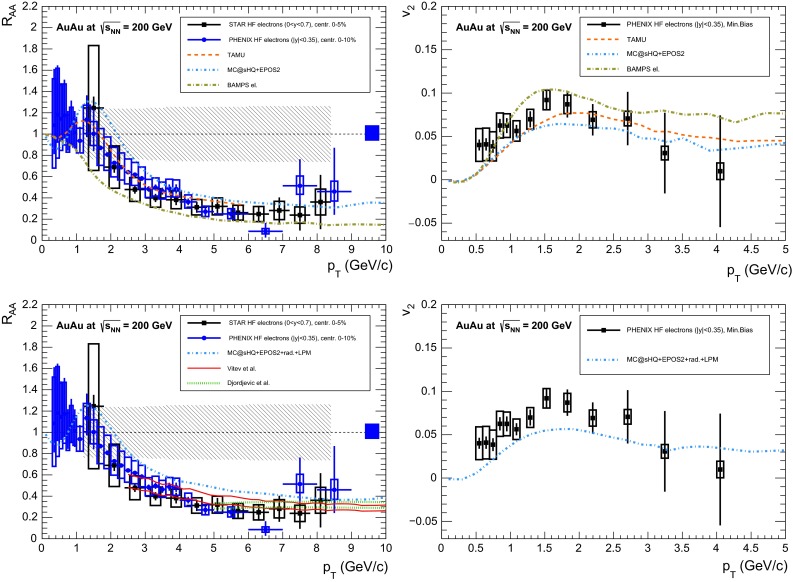
*Left* nuclear modification factor as a function of transverse momentum of heavy flavour electrons in the 0–5 % [420] and 0–10 % [469] most central Au–Au collisions at $\sqrt{s_{\mathrm{NN}}}$ $=$ 200 GeV. The *dashed band* (*filled box*) at $R_{\mathrm {AA}} =1$ is the normalisation uncertainty for STAR (PHENIX) data. *Right* $v_{2}$ as a function of transverse momentum of heavy flavour electrons in Au–Au collisions at $\sqrt{s_{\mathrm{NN}}}$ $=$ 200 GeV [469]. The results are compared to model calculations implementing collisional energy loss (*top panels*) and collisional and radiative energy loss (*bottom panels*)

**Fig. 69:**
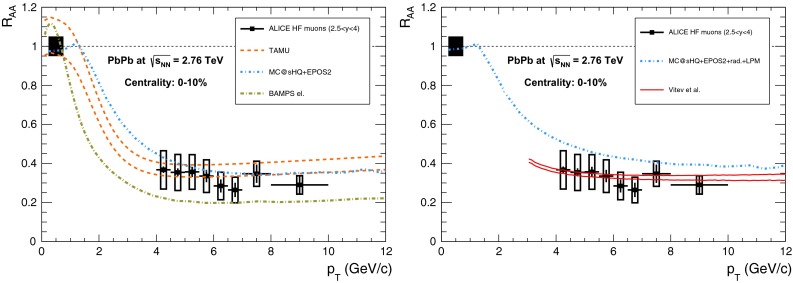
Nuclear modification factor as a function of transverse momentum of heavy flavour muons with $2.5<y<4$ measured in the 0–10 % most central Pb–Pb collisions at $\sqrt{s_{\mathrm{NN}}}$ $=$ 2.76 TeV [120]. The *filled box* at $R_{\mathrm {AA}} =1$ is the systematic uncertainty on the normalisation. The results are compared to model calculations implementing collisional energy loss (*left*) and collisional and radiative energy loss (*right*)

**Fig. 70:**
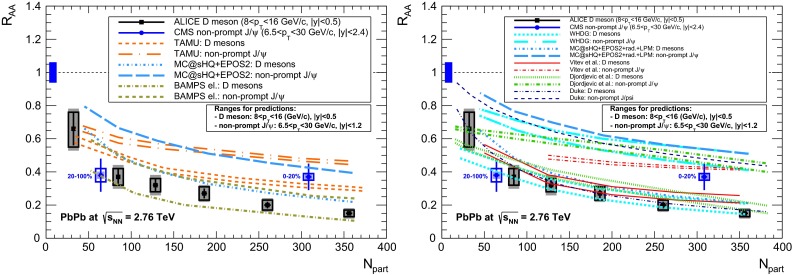
Nuclear modification factor as a function of the number of participants of averaged prompt D mesons [477] and non-prompt $\mathrm {J}/\psi$  [482] measured in Pb–Pb collisions at $\sqrt{s_{\mathrm{NN}}}$ =2.76 TeV compared to model calculations implementing collisional (*left*) and collisional and radiative energy loss (*right*). The *filled box* at $R_{\mathrm {AA}} =1$ is the systematic uncertainty on the normalisation of non-prompt $\mathrm {J}/\psi$ data. Note that: **a** model predictions refer to CMS preliminary data, in a slightly different rapidity range, **b** the point at low $\mathrm {N_{part}}$ for the non-prompt $\mathrm {J}/\psi$ $R_{\mathrm {AA}}$ refers to a very large centrality interval (20–100 %)

Figure 66 shows the comparison for D mesons in Pb–Pb collisions at $\sqrt{s_{\mathrm{NN}}} = 2.76~\text {TeV}$, measured by the ALICE Collaboration [477, 479]. The left panels show $R_{\mathrm {AA}}$ in the centrality class 0–20 %, the right panels show $v_{2}$ in the centrality class 30–50 %. The models that include only collisional energy loss are shown in the upper panels. These models provide in general a good description of $v_{2}$. The original version of the POWLANG model does not exhibit a clear maximum in $v_{2}$ like the other models, which could be due to the fact that it does not include, in such a version, hadronisation via recombination. The latter has been recently introduced in the POWLANG model and the additional flow inherited by the D mesons from the light quarks moves the calculations to higher values of $v_{2}$. In the TAMU model the decrease of $v_{2}$ towards high $p_{\mathrm {T}}$ is faster than in the other models, which reflects a moderate coupling with the medium, also seen in the rise of $R_{\mathrm {AA}}$ of D mesons at large $p_{\mathrm {T}}$. In this range, some of the other models over-suppress the $R_{\mathrm {AA}}$ and one observes large discrepancies between them, which mostly originate from the medium description as well as from the transport coefficients adopted in each model. At low $p_{\mathrm {T}}$ the models (UrQMD, BAMPS) that do not include PDF shadowing give a $R_{\mathrm {AA}}$ value larger than observed in the data. The models that include both radiative and collisional energy loss are shown in the central panels. All these models provide a good description of $R_{\mathrm {AA}}$, but most of them underestimate the maximum of $v_{2}$ observed in data. This could be due to the fact that the inclusion of radiative process reduces the weight of collisional process, which are more effective in building up the azimuthal anisotropy. In addition, some of these models (Djordjevic et al., WHDG, Vitev et al.) do not include a fluid-dynamical medium (for this reason, the Djordjevic et al. and Vitev et al. models do not provide a calculation for $v_{2}$), and none of them implements the detailed balance reaction which is mandatory to reach thermalisation and then undergo the full drift from the medium. The POWLANG model with l-QCD-based transport coefficient and the AdS/CFT predictions are plotted in the lower panels. POWLANG provides a good description of $R_{\mathrm {AA}}$, while, for what concerns $v_{2}$, the results depend crucially on the way hadronisation is described, recombination scenarios leading to a larger elliptic flow (although still smaller than the experimental data in the accessible $p_{\mathrm {T}}$ range). AdS/CFT, on the other hand, over-predicts the suppression in the full $p_{\mathrm {T}}$ range explored.

Figure 67 shows the comparison for the $$\mathrm {D}^{0}$$ meson $$R_{\mathrm {AA}}$$ in Au–Au collisions at $$\sqrt{s_{\mathrm{NN}}} =200$$$$~\text {GeV}$$, measured by the STAR Collaboration [475]. The models that include collisional interactions in an expanding fluid-dynamical medium (TAMU, BAMPS, Duke, MC@$$_s$$HQ, POWLANG) describe qualitatively the shape of $$R_{\mathrm {AA}}$$ in the interval 0–3$$~\text {GeV}/c$$, with a rise, a maximum at 1.5$$~\text {GeV}/c$$ with $$R_{\mathrm {AA}} >1$$, and a decrease. In these models, this shape is the effect of radial flow on light and charm quarks. The TAMU model also includes flow in the hadronic phase. It can be noted that these models predict a similar bump also at LHC energy (left panels of Fig. 66): the bump reaches $$R_{\mathrm {AA}} >1$$ for the models that do not include PDF shadowing, while it stays below $$R_{\mathrm {AA}} =0.8$$ for the models that include it. The present ALICE data for $$p_{\mathrm {T}} >2$$$$~\text {GeV}/c$$ do not allow one to draw a strong conclusion. However, the preliminary ALICE data reaching down to 1$$~\text {GeV}/c$$ in the centrality class 0–7.5 % [488] do not favour models that predict a bump with $$R_{\mathrm {AA}} >1$$.

The comparisons with measurements of heavy-flavour decay leptons at RHIC and LHC are shown in  Figs. 68 and 69, respectively. The $$R_{\mathrm {AA}}$$ and $$v_{2}$$ of heavy-flavour decay electrons in Au–Au collisions at top RHIC energy, measured by PHENIX [469] and STAR [420], are well described by all model calculations. Note that in some of the models the quark–medium coupling (medium density or temperature or interaction cross section) is tuned to describe the $$R_{\mathrm {AA}}$$ of pions (Djordjevic et al., WHDG, Vitev et al.) or electrons (BAMPS) at RHIC. The $$R_{\mathrm {AA}}$$ of heavy-flavour decay muons at forward rapidity ($$2.5<y<4$$), measured by ALICE in central Pb–Pb collisions at LHC [120], is well described by most of the models. The BAMPS model tends to over-suppress this $$R_{\mathrm {AA}}$$, as observed also for the high-$$p_{\mathrm {T}}$$$$R_{\mathrm {AA}}$$ of D mesons at RHIC and LHC. The MC@$$_s$$HQ model describes the data better when radiative energy loss is not included. In general, it can be noted that the differences between the various model predictions are less pronounced in the case of heavy-flavour decay lepton observables than in the case of D mesons. This is due to the fact that the former include a $$p_{\mathrm {T}}$$-dependent contribution of charm and beauty decays. In addition, the decay kinematics shifts the lepton spectra towards low momentum, reducing the impact on $$R_{\mathrm {AA}}$$ of effects like PDF shadowing, radial flow and recombination.

**Fig. 71:**
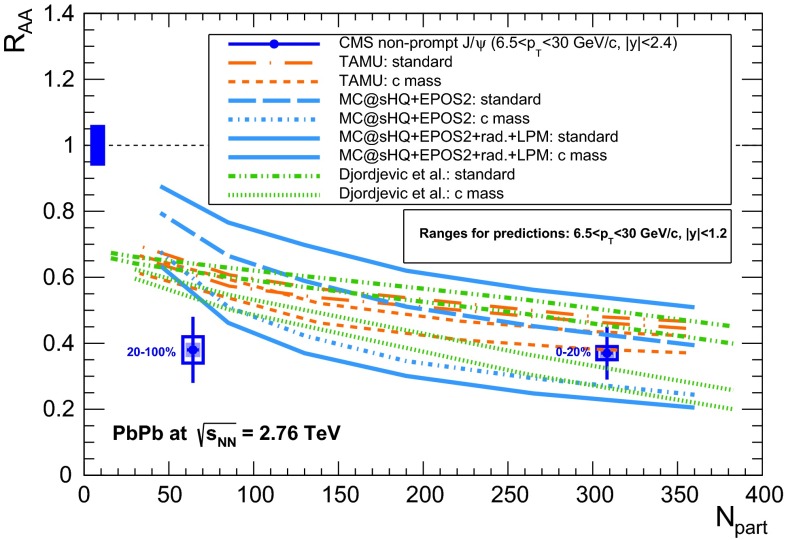
Quark mass dependence of the energy loss. The nuclear modification factor of non-prompt $\mathrm {J}/\psi$  [482] measured in Pb–Pb collisions at $\sqrt{s_{\mathrm{NN}}}$ $=$ 2.76 TeV is compared to model calculations obtained in the same way as in Fig. 70 and assuming that the $b$ quark has the mass of the $c$ quark

In Fig. 70 we compile the model calculations for the centrality dependence of the nuclear modification factors of D mesons in the interval $$8<p_{\mathrm {T}} <16$$$$~\text {GeV}/c$$ and non-prompt $$\mathrm {J}/\psi $$ mesons in the interval $$6.5<p_{\mathrm {T}} <30$$$$~\text {GeV}/c$$, in Pb–Pb collisions at $$\sqrt{s_{\mathrm{NN}}} = 2.76$$ $$\text {TeV}$$. All models predict the $$R_{\mathrm {AA}}$$ of D mesons to be lower by about 0.2–0.3 units than that of non-prompt $$\mathrm {J}/\psi $$. This difference arises from the mass dependence of quark–medium interactions. The available published data, from the first limited-statistics Pb–Pb run at LHC (in 2010), are reported in the figure: note that the D-meson $$R_{\mathrm {AA}}$$ measured by the ALICE experiment [477] corresponds to the interval 6–12$$~\text {GeV}/c$$ (slightly lower than that of the calculations), while the non-prompt $$\mathrm {J}/\psi $$$$R_{\mathrm {AA}}$$ measured by the CMS experiment [482] corresponds to the large centrality classes 0–20 and 20–100 %. Due to the large uncertainties and the large centrality intervals, the data do not allow for a clear conclusion on the comparison with models. The preliminary ALICE [497] and CMS [494] measurements using the higher-statistics 2011 Pb–Pb sample are well described by the model calculations. The effect of the heavy-quark mass on the nuclear modification factor is illustrated in Fig. 71, where the $$R_{\mathrm {AA}}$$ of non-prompt J/$$\psi $$ is obtained in the Djordjevic et al., MC@$$_s$$HQ and TAMU models using the *c*-quark mass value for the calculation of the in-medium interactions of *b* quarks. In this case, substantially lower values of $$R_{\mathrm {AA}}$$ are obtained.

**Fig. 72:**
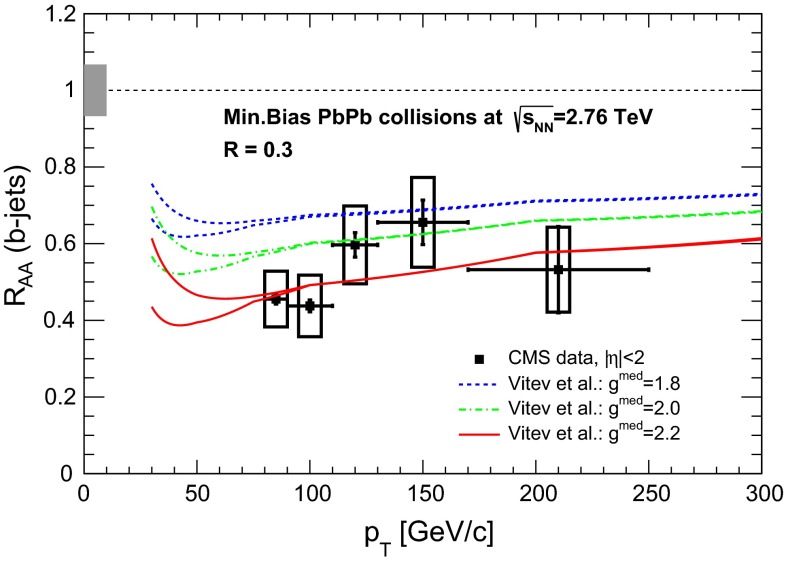
Nuclear modification factor as a function of transverse momentum of $b$-jets measured in Pb–Pb collisions at $\sqrt{s_{\mathrm{NN}}}$ $=$ 2.76 TeV [481] compared to model calculations. For each value of $g^\mathrm{med}$, the *upper curve* is the calculation with the *b*-quark mass and the *lower curve* is the massless case. The *filled box* at $R_{\mathrm {AA}} =1$ is the systematic uncertainty on the normalisation

Finally, in Fig. 72 the nuclear modification factor of $b$-tagged jets measured by the CMS Collaboration in minimum-bias Pb–Pb collisions at $\sqrt{s_{\mathrm{NN}}} = 2.76$ $\text {TeV}$ is compared with the model described at the end of Sect. 4.3.1, including radiative and collisional energy loss. The calculation is shown for three values of the quark–medium coupling parameter $g^\mathrm{med}$ [495]. A precise measurement of this observable in future LHC runs should allow one to constrain this parameter to the 10 % level. In addition, an extension of the measurement to transverse momenta lower than 50$~\text {GeV}/c$ should allow one to observe the reduction of suppression due to the mass dependence of the energy loss.

In summary, the comparison of model calculations with currently available data from RHIC and LHC allows for the following considerations:

- the D-meson $v_{2}$ measurements at LHC are best described by the models that include collisional interactions within a fluid-dynamical expanding medium, as well as hadronisation via recombination;
- however, theoretical predictions of the $R_{\mathrm {AA}}$ of D mesons from these models are scattered, both at RHIC and LHC, which leaves room for theoretical improvement in the future before reliable conclusions can be drawn;
- on the contrary, the models that include radiative and collisional energy loss provide a good description of the D-meson $R_{\mathrm {AA}}$, but they underestimate the value of $v_{2}$ at LHC;
- the models that include collisional energy loss in a fluid-dynamical expanding medium, hence radial flow, exhibit a bump in the low-$p_{\mathrm {T}}$ D-meson $R_{\mathrm {AA}}$, which is qualitatively consistent with the RHIC data;
- these models predict a bump also at LHC energy, the size of which depends strongly on the nuclear modification of the PDFs (shadowing); the current data at LHC are not precise enough to be conclusive in this respect;
- most of the models can describe within uncertainties the measurements of $R_{\mathrm {AA}}$ and $v_{2}$ for heavy-flavour decay electrons at RHIC (in some models, the quark–medium coupling is tuned to describe these data) and of $R_{\mathrm {AA}}$ for heavy-flavour decay muons at LHC;
- all models predict that the $$R_{\mathrm {AA}}$$ of non-prompt $$\mathrm {J}/\psi $$ from B decays is larger than that of D mesons by about 0.2–0.3 units for the $$p_{\mathrm {T}}$$ region ($$\sim 10$$$$~\text {GeV}/c$$) for which preliminary data from the LHC experiments exist.

### Heavy-flavour correlations in heavy-ion collisions: status and prospects

Angular correlations of charged hadrons proved to be key observables at RHIC and LHC energies to study energy loss and QGP properties [629–634], providing measurements that are complementary to single-particle observables like the $R_{\mathrm {AA}}$ and $v_{2}$. Two-particle correlation distributions are defined in terms of the ($\Delta \phi$, $\Delta \eta$) distance between a $p_{\mathrm {T}}$-selected trigger particle and a (set of) associated particles, generally with lower $p_{\mathrm {T}}$ than the trigger particle. On the near side ($\Delta \phi \sim 0$), the correlations provide information on the properties of the jet leaving the medium, while on the away side ($\Delta \phi \sim \pi$) they reflect the “survival” probability of the recoiling parton that traverses the medium. Di-hadron correlation measurements typically carry geometrical and kinematical biases [635]. Triggering on a high-$p_{\mathrm {T}}$ particle tends to favour the selection of partons produced near the surface of the medium, which lost a small fraction of their energy and could still fragment to hadrons at high $p_{\mathrm {T}}$ (geometrical bias). In addition, when comparing to the vacuum case ($\mathrm pp$ collisions) with the same conditions on the trigger particles, one might have different contributions of quark and gluon jets and different partonic energies in nucleus–nucleus and $\mathrm pp$ collisions (parton and kinematical biases). Together with single-particle measurements and fully reconstructed jets, di-hadron correlations can constrain energy-loss models by adding information on the path length dependence of the energy loss and the relative contributions of collisional and radiative energy loss.

**Fig. 73:**
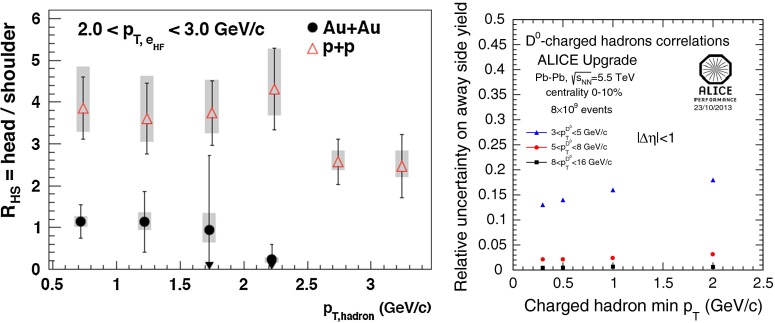
*Left* ratio of yields in the away-side region ($2.51<\phi <\pi$) to those in the shoulder region ($1.25<\phi <2.51$) in $\mathrm pp$ and Au–Au collisions from PHENIX [113]. *Right* relative uncertainty on the away-side yield in D–h correlations as a function of the charged hadron $p_{\mathrm {T}}$ for three ranges of the D-meson transverse momenta, with the ALICE and LHC upgrades [638]

Recent works [530, 539, 586, 610, 636, 637] have shown that the azimuthal distributions of heavy quark-antiquark pairs are sensitive to the different interaction mechanisms, collisional and radiative. The relative angular broadening of the ${Q\overline{Q}}$ pair does not only depend on the drag coefficient discussed above (see Sect. 4.3) but also on the momentum broadening in the direction perpendicular to the initial quark momentum, $\langle p_\perp ^2\rangle$, which is not probed directly in the traditional $R_{\mathrm {AA}}$ and $v_{2}$ observables. This is one of the motivation for measuring azimuthal correlations of heavy-flavour.

The experimental challenges in measurements like D–$\overline{\mathrm{D}}$ correlations in heavy-ion collisions come from the reconstruction of both the hadronic decays of the back-to-back D mesons, which require large statistics to cope with low branching ratios and low signal-to-background in nucleus–nucleus collisions. As an alternative, correlations of D mesons with charged hadrons (D–h), correlations of electrons/muons from decays of heavy-flavour particles with charged hadrons ($e$–h) and correlations of D–$e$, $e^{+}-e^{-}$, $\mu ^{+}-\mu ^{-}$ and $e$–$\mu$ pairs (where electrons and muons come from heavy-flavour decays) can be studied. Such observables might, however, hide decorrelation effects intrinsic to the decay of heavy mesons. In addition, in the case of correlations triggered by electrons or muons from heavy-flavour decays, the interpretation of the results is complicated by the fact that the lepton carries only a fraction of the momentum of the parent meson. This makes the understanding of $\mathrm pp$ collisions as baseline a very crucial aspect of these analyses (see Sect. 2.4.2).

Heavy-flavour azimuthal correlations are being studied in d–Au collisions at RHIC and p–Pb collisions both at RHIC and at the LHC to understand how the presence of the nucleus might affect the properties of heavy-flavour pair production. The results are discussed in Sect. 3.3.2.

**Fig. 74:**
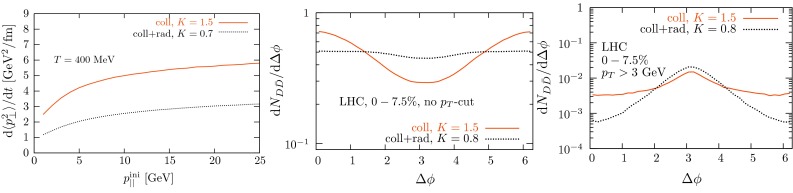
Perpendicular momentum of charm quarks acquired in a QGP medium at $T=400$ $~\text {MeV}$ as a function of the initial momentum $p_{||}^\mathrm{ini}$ (*left*). Angular correlations of $\mathrm {D\overline{D}}$ pairs in Pb–Pb collisions at LHC without (*centre*) and with (*right*) a lower momentum cut [530]

Measurements of heavy-flavour correlations in nucleus–nucleus collisions were carried out at both RHIC and LHC with $e$–h correlations [113, 639, 640] (where electrons come from heavy-flavour decays), but the current statistics prevents us from drawing quantitative conclusions. Such measurements are expected to provide more information as regards how the charm and beauty quarks propagate through the hot and dense medium and how this affects and modifies the correlation structures. In particular, PHENIX reported a decrease of the ratio of yields in the away-side region ($2.51<\Delta \phi <\pi$) to those in the shoulder region ($1.25<\Delta \phi <2.51$) from $\mathrm pp$ to Au–Au collisions (left panel of Fig. 73).

Further measurements of heavy-flavour triggered azimuthal correlations will be promising in future data takings at both RHIC (with the new silicon tracker detectors) and LHC (with the machine and detector upgrades). As reported in Fig. 73, right, the relative uncertainty on the away-side yield in D–h correlations in central Pb–Pb collisions with the ALICE and LHC upgrades will be $\approx$ 15 % for low-$p_{\mathrm {T}}$ D mesons and only a few percent for intermediate/high $p_{\mathrm {T}}$.

Several theoretical works have recently addressed angular correlations of heavy-flavour particles in nucleus–nucleus collisions [530, 539, 586, 610, 636, 637]. However, none of these approaches presently includes the interactions of D and B mesons in the hadronic phase present in the late stages of the system evolution. These interactions could add a further smearing on top of QGP-induced modification of the heavy-quark angular correlations. For the traditional $$R_{\mathrm {AA}}$$ and $$v_{2}$$ observables, a first step in this direction was made in Refs. [491, 641, 642], with effects found of the order of 20 % at most. We now focus on a particular model in order to illustrate the sensitivity of heavy-flavour angular correlations to the type of interaction mechanism [530]. In Fig. 74 (left), the transverse momentum broadening per unit of time is shown as a function of the initial momentum $$p_{||}^\mathrm{ini}$$ of charm quarks for the purely collisional and collisional plus radiative (LPM) interactions as applied within the MC@$$_s$$HQ model (see Sect. 4.3.2). For all initial momenta, $$\langle p_\perp ^2\rangle $$ is larger in a purely collisional interaction mechanism. $$\langle p_\perp ^2\rangle $$ has similar numerical values for charm and for beauty quarks. A larger $$\langle p_\perp ^2\rangle $$ leads to a more significant change of the initial relative pair azimuthal angle $$\Delta \phi $$ during the evolution in the medium. This means that for a purely collisional interaction mechanism one expects a stronger broadening of the initial correlation at $$\Delta \phi =\pi $$, as seen in the central and right panels of Fig. 74. In the central panel, the $$\Delta \phi $$ distribution of all initially correlated pairs is shown after hadronisation into $$\mathrm {D\overline{D}}$$ pairs. Since no cut in $$p_{\mathrm {T}}$$ is applied, these distributions are dominated by low-momentum pairs, while in the right panel a cut of $$p_{\mathrm {T}} >3$$$$~\text {GeV}/c$$ is applied. The low-momentum pairs show the influence of the radial flow of the underlying QGP medium, which tends to align the directions of the quark and the antiquarks toward smaller opening angles. It again happens more efficiently for larger $$\langle p_\perp ^2\rangle $$ of the underlying interaction mechanism. This effect, which was called “partonic wind” [643], is thus only seen for the purely collisional interaction mechanism. A $$p_{\mathrm {T}}$$ threshold reveals clearly the residual correlation around $$\Delta \phi \sim \pi $$. Here in the purely collisional scenario one sees a larger background of pairs that decorrelated during the evolution in the QGP than for the collisional plus radiative (LPM) scenario.

For these calculations an initial back-to-back correlation has been assumed. Next-to-leading order processes, however, destroy this strict initial correlation already in proton–proton collisions. Unfortunately the theoretical uncertainties on these initial distributions are very large, especially for charm quarks. Here, a thorough experimental study of heavy-flavour correlations in proton–proton and proton–nucleus collisions is very important for validating different initial models. Also enhanced theoretical effort in these reference systems is necessary.

### Summary and outlook

The LHC Run 1 has provided a wealth of measurements of heavy-flavour production in heavy-ion collisions, which have extended and complemented the results from the RHIC programme. The main observations and their present interpretation are summarised in the following.

*High-*$p_{\mathrm {T}}$*region* (above 5–10 GeV/*c*): in this region, heavy-flavour measurements are expected to provide information mainly on the properties of in-medium energy loss.

- The $R_{\mathrm {AA}}$ measurements show a strong suppression with respect to binary scaling in central nucleus–nucleus collisions for D mesons, heavy-flavour decay leptons and J$/\psi$ from B decays. The suppression of D mesons and heavy-flavour decay leptons is similar, within uncertainties, at RHIC and LHC energies. Given that a suppression is not observed in proton(deuteron)–nucleus collisions, the effect in nucleus–nucleus collisions can be attributed to in-medium energy loss.
- The suppression of D mesons with average $p_{\mathrm {T}}$ of about 10 GeV/*c* is stronger than that of J/$\psi$ decaying from B mesons with similar average $p_{\mathrm {T}}$. This observation, still based on preliminary results, is consistent with the expectation of lower energy loss for heavier quarks and it is described by model calculations that implement radiative and collisional energy loss with this feature.
- The suppression of D mesons and pions is consistent within uncertainties at both RHIC and LHC. While there is no experimental evidence of the colour-charge dependence of the energy loss, model calculations indicate that similar $R_{\mathrm {AA}}$ values can result from the combined effect of colour-charge dependent energy loss and the softer $p_{\mathrm {T}}$ distribution and fragmentation function of gluons with respect to *c* quarks.
- At very high-$p_{\mathrm {T}}$ (above 100 GeV/*c*), a similar $R_{\mathrm {AA}}$ is observed for *b*-tagged jets and inclusive jets. This observation is consistent with a negligible effect of the heavy quark mass at these scales.

*Low-*$p_{\mathrm {T}}$*region* (below 5–10 GeV/*c*): in this region, heavy-flavour measurements are expected to provide information on the total production yields (and the role of initial-state effects) and on heavy-quark in-medium dynamics (participation to collective expansion, in-medium hadronisation effects).

- The measurements of electrons (in particular) and D mesons at RHIC show that the total production of charm quarks is consistent with binary scaling within uncertainties of about 30–40 %. The available measurements at LHC do not extend to sufficiently small $p_{\mathrm {T}}$ to provide an estimate of the total yields.
- The D-meson $R_{\mathrm {AA}}$ at RHIC energy shows a pronounced maximum at $p_{\mathrm {T}}$ of about 1–2 GeV/*c* (where $R_{\mathrm {AA}}$ becomes larger than unity). This feature is not observed in the measurements at LHC energy. Model calculations including collisional (elastic) interaction processes in an expanding medium and a contribution of hadronisation via in-medium quark recombination, as well as initial-state gluon shadowing, describe qualitatively the behaviour observed at both energies. In these models the bump at RHIC is due to radial flow and the effect on $R_{\mathrm {AA}}$ at LHC is strongly reduced because of the harder $p_{\mathrm {T}}$ distributions and of the effect of gluon shadowing.
- A positive elliptic flow $v_2$ is measured in non-central collisions for D mesons at LHC and heavy-flavour decay leptons at RHIC and LHC. The D-meson $v_2$ at LHC is comparable to that of light-flavour hadrons (within uncertainties of about 30 %). These measurements indicate that the interaction with the medium constituents transfers information as regards the azimuthal anisotropy of the system to charm quarks. The $v_2$ measurements are best described by the models that include collisional interactions within a fluid-dynamical expanding medium, as well as hadronisation via recombination.

The main open questions in light of these observations are:

- Does the total charm and beauty production follow binary scaling or is there a significant gluon shadowing effect? This requires a precise measurement of charm and beauty production down to zero $p_{\mathrm {T}}$, in proton–proton, proton–nucleus and nucleus–nucleus collisions.
- Can there be an experimental evidence of the colour-charge dependence of the energy loss? This requires a precise comparison of D mesons and pions in the intermediate $p_{\mathrm {T}}$ region, at both RHIC and LHC.
- Is the difference in the nuclear modification factor of charm and beauty hadrons consistent with the quark mass dependent mechanisms of the energy loss? Can it provide further insight on these mechanisms (for example, the gluon radiation angular distribution)? This requires a precise measurement of D and B meson (or J/$\psi$ from B) $R_{\mathrm {AA}}$ over a wide $p_{\mathrm {T}}$ range and as a function of collision centrality. This will also be mandatory in order to extract the precise path-length dependence of the energy loss, which cannot be extracted from the actual data.
- Does the positive elliptic flow observed for D mesons and heavy-flavour decay leptons result from the charm quark interactions in the expanding medium? Are charm quarks thermalised in the medium? Is there a contribution (dominant?) inherited from light quarks via the recombination process? What is the contribution from the path length dependence of the energy loss? This requires precise measurements of the elliptic flow and of the higher-order flow coefficients of charm and beauty hadrons over a wide $p_{\mathrm {T}}$ interval, and their comparison with light-flavour hadrons.
- What is the role of in-medium hadronisation and of radial flow for heavy quarks? This requires measurements of $R_{\mathrm {AA}}$ and $v_2$ of heavy flavour hadrons with different quark composition and different masses, namely D, $\mathrm{D}_s$, B, $\mathrm{B}_s$, $\Lambda _c$, $\Xi _c$, $\Lambda _b$.
- What is the relevance of radiative and collisional processes in heavy quark energy loss? What is the path length dependence of the two types of processes? This requires precise simultaneous measurements of the $R_{\mathrm {AA}}$ and $v_2$ and their comparison with model calculations. Heavy-quark correlations are also regarded as a promising tool in this context.

The outlook for addressing these open questions with the future experimental programmes at RHIC and LHC is discussed in Sect. 7.

From the theoretical point of view, a wide range of models, also with somewhat different “ingredients”, can describe most of the available data, at least qualitatively. The main challenges in the theory sector is thus to connect the data with the fundamental properties of the QGP and of the theory of the strong interaction. For this purpose, it is important to identify the features of the quark–medium interaction that are needed for an optimal description of all aspects of the data and to reach a uniform treatment of the “external inputs” in the models (e.g. using state-of-the-art pQCD baseline, fragmentation functions and fluid-dynamical medium description, and fixing transport coefficients on those that will be ultimately obtained from lattice calculations for finite $p_{\mathrm {T}}$).

## Quarkonia in nucleus–nucleus collisions

Quarkonia are considered important probes of the QGP formed in heavy-ion collisions. In a hot and deconfined medium quarkonium production is expected to be significantly suppressed with respect to the proton–proton yield, scaled by the number of binary nucleon–nucleon collisions, as long as the total charm cross section remains unmodified.^23^ The origin of such a suppression, taking place in the QGP, is thought to be the colour screening of the force that binds the ${c\overline{c}}$ (${b\overline{b}}$) state [644]. In this scenario, quarkonium suppression should occur sequentially, according to the binding energy of each meson: strongly bound states, such as the $\Upsilon \text {(1S)}$ or the $\mathrm {J}/\psi$, should melt at higher temperatures with respect to the more loosely bound ones, such as the $\chi _b$, $\Upsilon \text {(2S)}$, or $\Upsilon \text {(3S)}$ for the bottomonium family or the $\psi \text {(2S)}$ and the $\chi _c$ for the charmonium one. As a consequence, the in-medium dissociation probability of these states should provide an estimate of the initial temperature reached in the collisions [645]. However, the prediction of a sequential suppression pattern is complicated by several factors. Feed-down decays of higher-mass resonances, and of *b*-hadrons in the case of charmonium, contribute to the observed yield of quarkonium states. Furthermore, other hot and cold matter effects can play a role, competing with the suppression mechanism.

On the one hand, the production of *c* and $\overline{c}$ quarks increases with increasing centre-of-mass energy. Therefore, at high energies, as at the LHC, the abundance of *c* and $\overline{c}$ quarks might lead to a new charmonium production source: the (re)combination of these quarks throughout the collision evolution [646] or at the hadronisation stage [647, 648]. This additional charmonium production mechanism, taking place in a deconfined medium, enhances the $\mathrm {J}/\psi$ yield and might counterbalance the expected $\mathrm {J}/\psi$ suppression. Also the ${b\overline{b}}$ cross section increases with energy, but, given the smaller number of ${b\overline{b}}$ pairs, with respect to ${c\overline{c}}$, this contribution is less important for bottomonia even in high-$\sqrt{s_{\mathrm{NN}}}$ collisions.

On the other hand, quarkonium production is also affected by several effects related to cold matter (the so-called cold nuclear matter effects, CNM) discussed in Sect. 3. For example, the production cross section of the ${Q\overline{Q}}$ pair is influenced by the kinematic parton distributions in nuclei, which are different from those in free protons and neutrons (the so-called nuclear PDF effects). In a similar way, approaches based on the Colour-Glass Condensate (CGC) effective theory assume that a gluon saturation effect sets in at high energies. This effect influences the quarkonium production occurring through fusion of gluons carrying small values of the Bjorken-*x* in nuclei. Furthermore, parton energy loss in the nucleus may decrease the pair momentum, causing a reduction of the quarkonium production at large longitudinal momenta. Finally, while the ${Q\overline{Q}}$ pair evolves towards the fully formed quarkonium state, it may also interact with partons of the crossing nuclei and eventually break-up. This effect is expected to play a dominant role only for low-$\sqrt{s_{\mathrm{NN}}}$ collisions, where the crossing time of the (pre)-resonant state in the nuclear environment is rather large. On the contrary, this contribution should be negligible at high-$\sqrt{s_{\mathrm{NN}}}$, where, due to the decreased crossing time, resonances are expected to form outside the nuclei. Cold nuclear matter effects are investigated in proton–nucleus collisions. Since these effects are present also in nucleus–nucleus interactions, a precise knowledge of their role is crucial in order to correctly quantify the effects related to the formation of hot QCD matter.

The in-medium modification of quarkonium production, induced by either hot or cold matter mechanisms, is usually quantified through the nuclear modification factor $R_{\mathrm {AA}}$, defined as the ratio of the quarkonium yield in AA collisions ($N_\mathrm{AA}^{{Q\overline{Q}}}$) and the expected value obtained by scaling the production cross section in pp collisions ($\sigma _\mathrm{pp}^{{Q\overline{Q}}}$) by the average nuclear overlap function ($\left\langle T_{\mathrm {AA}} \right\rangle$), evaluated through a Glauber model calculation [626]:

54 $$\begin{aligned} R_{\mathrm {AA}} = \frac{N_\mathrm{AA}^{{Q\overline{Q}}}}{\langle T_\mathrm{AA} \rangle \times \sigma _\mathrm{pp}^{{Q\overline{Q}}}}. \end{aligned}$$

$R_{\mathrm {AA}}$ is expected to equal unity if nucleus–nucleus collisions behave as a superposition of nucleon–nucleon interactions. This is, e.g., the case for electroweak probes (direct $\gamma$, W, and Z) that do not interact strongly [649–653]. Such a scaling is assumed to approximately hold for the total charm cross section, although an experimental verification has large uncertainties at RHIC ($\approx$30 %) [469, 475] and is still lacking at the LHC (see discussion in Sect. 4). A value of $R_{\mathrm {AA}}$ different from unity implies that the quarkonium production in AA is modified with respect to a binary nucleon–nucleon scaling. Further insight on the in-medium modification of quarkonium production can be obtained by investigating the rapidity and transverse momentum dependence of the nuclear modification factor.

**Fig. 75:**
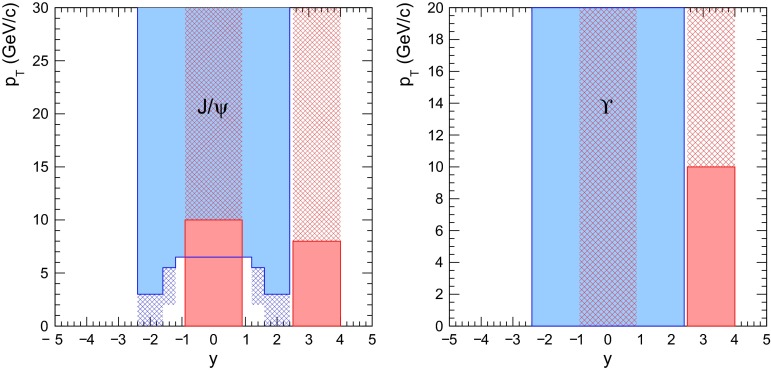
*Left* $p_{\mathrm {T}}$-*y* acceptance coverage of the ALICE (*red*) and CMS (*blue*) experiments for $\mathrm {J}/\psi$. *Right* $p_{\mathrm {T}}$-*y* acceptance coverage of the ALICE and CMS experiments for $\Upsilon \text {(nS)}$. *Filled areas* correspond the ranges investigated in recent ALICE and CMS quarkonium publications. The *hashed areas* correspond to the acceptance range which can potentially be covered by the experiments. In fact, while the high-$p_{\mathrm {T}}$ reach in ALICE is limited by statistics, the low-$p_{\mathrm {T}}$ $\mathrm {J}/\psi$ coverage by CMS is limited by the muon identification capabilities, affected by the large background in Pb–Pb collisions

The information from $R_{\mathrm {AA}}$ can be complemented by the study of the quarkonium azimuthal distribution with respect to the reaction plane, defined by the beam axis and the impact-parameter vector of the colliding nuclei. The second coefficient of the Fourier expansion of the azimuthal distribution, $v_{2}$, is called elliptic flow, as explained in Sect. 4. Being sensitive to the dynamics of the partonic stages of heavy-ion collisions, $v_{2}$ can provide details on the quarkonium production mechanisms: in particular, $\mathrm {J}/\psi$ produced through a recombination mechanism, should inherit the elliptic flow of the charm quarks in the QGP, acquiring a positive $v_{2}$.

Studies performed for thirty years, first at the SPS ($$\sqrt{s_{\mathrm{NN}}}$$$$=$$ 17$$~\text {GeV}$$) and then at RHIC ($$\sqrt{s_{\mathrm{NN}}}$$$$=$$ 39–200$$~\text {GeV}$$),^24^ have indeed shown a reduction of the $$\mathrm {J}/\psi $$ yield beyond the expectations from cold nuclear matter effects (such as nuclear shadowing and $${c\overline{c}}$$ break-up). Even if the centre-of-mass energies differ by a factor of ten, the amount of suppression, with respect to $$\mathrm pp$$ collisions, observed by SPS and RHIC experiments at mid-rapidity is rather similar. This observation suggests the existence of an additional contribution to $$\mathrm {J}/\psi $$ production, the previously mentioned (re)combination process, which sets in already at RHIC energies and can compensate for some of the quarkonium suppression due to screening in the QGP. Furthermore, $$\mathrm {J}/\psi $$ suppression at RHIC is, unexpectedly, smaller at mid-rapidity than at forward rapidity (*y*), in spite of the higher energy density which is reached close to $$y \sim 0$$. The stronger $$\mathrm {J}/\psi $$ suppression at forward-*y* might be considered a further indication of the role played by (re)combination processes. Note, however, that the rapidity dependence of the (re)combination contribution is expected to be rather small [654, 655]. On the other hand, at RHIC energies, cold nuclear matter effects can also explain the observed difference [64], at least partially.

The measurement of charmonium production is especially promising at the LHC, where the higher energy density reached in the medium and the larger number of ${c\overline{c}}$ pairs produced in central Pb–Pb collisions (increased by a factor ten with respect to RHIC energies, see Fig. 1) should help to disentangle suppression and (re)combination scenarios. Furthermore, at LHC energies also bottomonium states, which were barely accessible at lower energies, are abundantly produced. Bottomonium resonances should shed more light on the processes affecting the quarkonium behaviour in the hot matter. The $\Upsilon$ mesons are, as previously discussed, expected to be less affected by production through (re)combination processes, due to the much smaller abundance of *b* and $\overline{b}$ quarks in the medium with respect to *c* and $\overline{c}$ (in central Pb–Pb collisions at LHC energies, the number of ${c\overline{c}}$ is a factor $\sim$20 higher than the number of ${b\overline{b}}$ pairs). Furthermore, due to the larger mass of the *b* quark, cold nuclear matter effects, such as shadowing, are expected to be less important for bottomonium than for charmonium states.

**Table 12:** Quarkonium results obtained in AA at SPS. The nucleon–nucleon energy in the centre-of-mass frame ($\sqrt{s_{\mathrm{NN}}}$), the covered kinematic range, the probes and observables are reported

Probe	Colliding system	$\sqrt{s_{\mathrm{NN}}}$ ($~\text {GeV}$)	*y*	$p_{\mathrm {T}}$ ($\text {GeV}/c$)	Observables	References
NA38						
$\mathrm {J}/\psi$	S–U	17.2	$0<y<1$	$p_{\mathrm {T}} >0$	$\sigma _{\mathrm {J}/\psi }$, $\sigma _{\mathrm {J}/\psi }/\sigma _{\text {Drell}{-}\text {Yan}}(\text {cent.})$	[656]
$\psi \text {(2S)}$					$\sigma _{\psi \text {(2S)}}$, $\sigma _{\psi \text {(2S)}}/\sigma _{\text {Drell}{-}\text {Yan}}(\text {cent.})$	
NA50						
$\mathrm {J}/\psi$	Pb–Pb	17.2	$0<y<1$	$p_{\mathrm {T}} >0$	Yield($p_{\mathrm {T}}$), $\sigma _{\mathrm {J}/\psi }$ and $\sigma _{\mathrm {J}/\psi }/\sigma _{\text {Drell}{-}\text {Yan}}(\text {cent.})$	[657–663]
$\psi \text {(2S)}$					Yield($p_{\mathrm {T}}$), $\sigma _{\psi \text {(2S)}}/\sigma _{\text {Drell}{-}\text {Yan}}$ and $\sigma _{\psi \text {(2S)}}/\sigma _{\mathrm {J}/\psi }(\text {cent.})$	[661, 664]
NA60						
$\mathrm {J}/\psi$	In–In	17.2	$0<y<1$	$p_{\mathrm {T}} >0$	$\sigma _{\mathrm {J}/\psi }/\sigma _{\text {Drell}{-}\text {Yan}}(\text {cent.})$	[236, 665]
					Polarisation	[236]

**Table 13:** Quarkonium results obtained in AA from RHIC experiments. The experiment, the probes, the collision energy ($\sqrt{s_{\mathrm{NN}}}$), the covered kinematic range and the observables are indicated

Probe	Colliding system	$\sqrt{s_{\mathrm{NN}}}$ ($~\text {GeV}$)	*y*	$p_{\mathrm {T}}$ ($\text {GeV}/c$)	Observables	References
PHENIX						
$\mathrm {J}/\psi$	Au–Au	200	$1.2<|y|<2.2$	$p_{\mathrm {T}} >0$	Yield and $R_{\mathrm {AA}} (\text {cent.},\,p_{\mathrm {T}},\,y)$	[666–668]
			$$|y|<0.35$$			
				$0<p_{\mathrm {T}} <5$	$v_{2} (p_{\mathrm {T}},\,y)$	[669]
			$$1.2<|y|<2.2$$			[670]
	Cu–Cu		$1.2<|y|<2.2$	$p_{\mathrm {T}} >0$	Yield and $R_{\mathrm {AA}} (\text {cent.},\,p_{\mathrm {T}},\,y)$	[671]
			$$|y|<0.35$$			
	Cu–Au		$1.2<|y|<2.2$		Yield and $R_{\mathrm {AA}} (\text {cent.},\,y)$	[672]
	U–U	193	$1.2<|y|<2.2$	$p_{\mathrm {T}} >0$	$R_{\mathrm {AA}} (\text {cent.})$	[673]
	Au–Au	62.4			Yield$(\text {cent.},\,p_{\mathrm {T}})$, $R_{\mathrm {AA}} (\text {cent.})$	[674]
		39				
$\Upsilon \text {(1S+2S+3S)}$		200	$|y|<0.35$		Yield, $R_{\mathrm {AA}} \text {(cent.)}$	[675]
STAR						
$\mathrm {J}/\psi$	Au–Au	200	$|y|<1$	$p_{\mathrm {T}} >0$	Yield and $R_{\mathrm {AA}} (\text {cent.},\,p_{\mathrm {T}})$	[239, 676]
					$$v_{2} (\text {cent.},\,p_{\mathrm {T}})$$	[677]
	Cu–Cu				Yield and $R_{\mathrm {AA}} (\text {cent.},\,p_{\mathrm {T}})$	[277, 676]
	U–U	193			$R_{\mathrm {AA}} (p_{\mathrm {T}})$	[678]
	Au–Au	62.4			Yield, $R_{\mathrm {AA}} (\text {cent.},\,p_{\mathrm {T}})$	
		39				
$\Upsilon \text {(1S)}$		200			$\sigma$ and $R_{\mathrm {AA}} (\text {cent.})$	[323]
$\Upsilon \text {(1S+2S+3S)}$					$R_{\mathrm {AA}} (\text {cent.})$	
	U–U	193				[678]

The four large LHC experiments (ALICE, ATLAS, CMS, and LHCb) have carried out studies on quarkonium production either in Pb–Pb collisions at $$\sqrt{s_{\mathrm{NN}}}$$$$=$$ 2.76 $$\text {TeV}$$^25^ or in p–Pb collisions at $$\sqrt{s_{\mathrm{NN}}}$$$$=$$ 5.02 $$\text {TeV}$$. Quarkonium production has also been investigated in $$\mathrm pp$$ interactions at $$\sqrt{s}$$$$=$$ 2.76, 7 and 8 $$\text {TeV}$$. The four experiments are characterised by different kinematic coverages, allowing one to investigate quarkonium production in $$|y|<4$$, down to zero transverse momentum.

ATLAS and CMS are designed to measure quarkonium production by reconstructing the various states in their dimuon decay channel. They both cover the mid-rapidity region: depending on the quarkonium state under study and on the $p_{\mathrm {T}}$ range investigated, the CMS rapidity coverage can reach up to $|y|<2.4$, and a similar *y* range is also covered by ATLAS. ALICE measures quarkonium in two rapidity regions: at mid-rapidity ($|y|<0.9$) in the dielectron decay channel and at forward rapidity ($2.5<y<4$) in the dimuon decay channel, in both cases down to zero transverse momentum. LHCb has taken part only in the $\mathrm pp$ and p–A LHC programmes during Run 1 and their results on quarkonium production, reconstructed through the dimuon decay channel, are provided at forward rapidity ($2<y<4.5$), down to zero $p_{\mathrm {T}}$. As an example, the $p_{\mathrm {T}}$-*y* acceptance coverages of the ALICE and CMS experiments are sketched in Fig. 75 for $\mathrm {J}/\psi$ (left) and $\Upsilon$ (right).

In Tables  12, 13 and 14, a summary of the charmonium and bottomonium results obtained in AA collisions by the SPS, RHIC, and LHC experiments are presented, respectively.

**Table 14:** Quarkonium results obtained in AA from LHC experiments. The experiment, the probes, the collision energy ($\sqrt{s_{\mathrm{NN}}}$), the covered kinematic range and the observables are indicated

Probe	Colliding system	$\sqrt{s_{\mathrm{NN}}}$ ($\text {TeV}$)	*y*	$p_{\mathrm {T}}$ ($\text {GeV}/c$)	Observables	References
ALICE						
$\mathrm {J}/\psi$	Pb–Pb	2.76	$|y|<0.9$	$p_{\mathrm {T}} >0$	$R_{\mathrm {AA}} (\text {cent.,}\,p_{\mathrm {T}})$	[480, 679]
			$2.5<y<4$	$p_{\mathrm {T}} >0$	$R_{\mathrm {AA}} (\text {cent.},\,p_{\mathrm {T}},\,y)$	[679, 680]
				$0<p_{\mathrm {T}} <10$	$v_{2} (\text {cent.},\,p_{\mathrm {T}})$	[681]
$\psi \text {(2S)}$				$p_{\mathrm {T}} <3$	$\frac{(N_{\psi \text {(2S)}}/N_{\mathrm {J}/\psi })_{\mathrm {Pb}{-}\mathrm {Pb}}}{(N_{\psi \text {(2S)}}/N_{\mathrm {J}/\psi })_{\mathrm {pp}}}(\text {cent.})$	[682]
				$$3<p_{\mathrm {T}} <8$$		
$\Upsilon \text {(1S)}$				$p_{\mathrm {T}} >0$	$R_{\mathrm {AA}} (\text {cent.},\,y)$	[683]
ATLAS						
$\mathrm {J}/\psi$	Pb–Pb	2.76	$|\eta |<2.5$	$p_{\mathrm {T}} \gtrsim 6.5$	$R_{\mathrm {CP}} (\text {cent.})$	[684]
CMS						
$\mathrm {J}/\psi$ (prompt)	Pb–Pb	2.76	$|y|<2.4$	$6.5<p_{\mathrm {T}} <30$	Yield and $R_{\mathrm {AA}} (\text {cent.},\,p_{\mathrm {T}},\,y)$	[482]
					$$v_{2} (\text {cent.},\,p_{\mathrm {T}},\,y)$$	[685]
			$1.6<|y|<2.4$	$3<p_{\mathrm {T}} <30$		
			$|y|<1.2$	$6.5<p_{\mathrm {T}} <30$	Yield and $R_{\mathrm {AA}}$	[482]
			$1.2<|y|<1.6$	$5.5<p_{\mathrm {T}} <30$		
			$1.6<|y|<2.4$	$3<p_{\mathrm {T}} <30$		
$\psi \text {(2S)}$ (prompt)			$1.6<|y|<2.4$	$3<p_{\mathrm {T}} <30$	$R_{\mathrm {AA}}$, $\frac{(N_{\psi \text {(2S)}}/N_{\mathrm {J}/\psi })_{\mathrm {Pb}{-}\mathrm {Pb}}}{(N_{\psi \text {(2S)}}/N_{\mathrm {J}/\psi })_{\mathrm {pp}}}(\text {cent.})$	[686]
			$|y|<1.6$	$6.5<p_{\mathrm {T}} <30$		
$\Upsilon \text {(1S)}$			$|y|<2.4$	$p_{\mathrm {T}} >0$	Yield and $R_{\mathrm {AA}} (\text {cent.},\,p_{\mathrm {T}},\,y)$	[482]
$\Upsilon \text {(nS)}$			$|y|<2.4$	$p_{\mathrm {T}} >0$	$R_{\mathrm {AA}} (\text {cent.})$	[687, 688]
					$$\frac{(N_{\Upsilon \text {(2S)}}/N_{\Upsilon \text {(1S)}})_{\mathrm {Pb}{-}\mathrm {Pb}}}{(N_{\Upsilon \text {(2S)}}/N_{\Upsilon \text {(1S)}})_{\mathrm {pp}}}(\text {cent.})$$	[268]

This section is organised as follows. In the first part, a theoretical overview is presented, in which the sequential suppression pattern of quarkonia and the lattice calculations are introduced. Other effects, such as modifications of the parton distribution functions inside nuclei and their influence on nucleus–nucleus collisions are discussed. Along with the suppression, the enhancement of quarkonia is also considered through two different approaches to (re)generation: the statistical hadronisation model and transport models. In the context of bottomonium studies, non-equilibrium effects on quarkonium suppression in the anisotropic hydrodynamic framework are also discussed. Finally, the collisional dissociation model and the comover interaction model are briefly introduced.

In the second part, experimental quarkonium results are reviewed. The recent LHC results, starting with a brief discussion on the quarkonium production cross sections in $\mathrm pp$ collisions as necessary references to build the nuclear modification factors, are presented. The description of the experimental $R_{\mathrm {AA}}$ results for $\mathrm {J}/\psi$ production, both at low and high $p_{\mathrm {T}}$ is then addressed. The LHC results are compared to those at RHIC energies and to theoretical models. A similar discussion is also introduced for the $\mathrm {J}/\psi$ azimuthal anisotropy. Results obtained at RHIC from variations of the beam energy and collision system are also addressed. The charmonium section is concluded with a discussion of $\psi \text {(2S)}$ production. Next, the bottomonium results on ground and excited states at RHIC and LHC energies are discussed.

Finally, other possible references for the quarkonium behaviour in nucleus–nucleus collisions, namely proton–nucleus collisions and open heavy flavour, production are discussed.

### Theory overview

#### Sequential suppression and lattice QCD

Historically, the large masses of charm and beauty quarks provide the basis for a quarkonium spectroscopy through non-relativistic potential theory, introducing a confining potential in terms of a string tension [437].

**Table 15:** Mass, binding energy and radius for charmonia and bottomonia [437]

State	$J/\psi$	$\chi _c \text {(1P)}$	$\psi \text {(2S)}$	$\Upsilon \text {(1S)}$	$\chi _b \text {(1P)}$	$\Upsilon \text {(2S)}$	$\chi _b \text {(2P)}$	$\Upsilon \text {(3S)}$
Mass (GeV$/c^2$)	3.07	3.53	3.68	9.46	9.99	10.02	10.26	10.36
$\mathrm{Binding}$ (GeV)	0.64	0.20	0.05	1.10	0.67	0.54	0.31	0.20
Radius (fm)	0.25	0.36	0.45	0.14	0.22	0.28	0.34	0.39

The QGP consists of deconfined colour charges, so that the binding of a ${Q\overline{Q}}$ pair is subject to the effect of colour screening which limits the range of strong interactions. Intuitively, the fate of heavy quark bound states in a QGP depends on the size of the colour screening radius $r_D$ (which is inversely proportional to the temperature, so that it decreases with increasing temperature) in comparison to the quarkonium binding radius $r_Q$: if $r_D \gg r_Q$, the medium does not really affect the heavy quark binding. Once $r_D \ll r_Q$, however, the two heavy quarks cannot “see” each other any more and hence the bound state will melt. It is therefore expected that quarkonia will survive in a QGP through some range of temperatures above $T_c$, and then dissociate once *T* becomes large enough. Recent studies have shown that the Debye-screened potential develops an imaginary part, implying a class of thermal effects that generate a finite width for the quarkonium peak in the spectral function. These results can be used to study quarkonium in a weakly coupled Quark Gluon Plasma within an Effective Field Theories (EFT) framework [689]. On the other hand lattice-QCD enables ab initio study of quarkonium correlation functions in the strongly coupled regime. The sequential dissociation scenario is confirmed by all these approaches [64].

In vacuum, progress in lattice calculations and effective field theories have turned quarkonium physics into a powerful tool to determine the heavy-quark masses and the strength of the QCD coupling, with an accuracy comparable to other techniques. The measurements of quarkonia in heavy-ion collisions provide quantitative inputs for the study of QCD at high density and temperature, providing an experimental basis for analytical and lattice studies to extract the in-medium properties of heavy-flavor particles and the implications for the QCD medium [64, 690–692].

Finite-temperature lattice studies on quarkonium mostly consist of calculations of spectral functions for temperatures in the range explored by the experiments. The spectral function $\rho (\omega )$ is the basic quantity encoding the equilibrium properties of a quarkonium state. It characterises the spectral distribution of binding strength as a function of energy $\omega$. Bound or resonance states manifest themselves as peaks with well-defined mass and spectral width. The in-medium spectral properties of quarkonia are related to phenomenology, since the masses determine the equilibrium abundances, their inelastic widths determine formation and destruction rates (or chemical equilibration times) and their elastic widths affect momentum spectra (and determine the kinetic equilibration times).

Spectral functions play an important role in understanding how elementary excitations are modified in a thermal medium. They are the power spectrum of autocorrelation functions in real time, hence provide a direct information on large time propagation. In the lattice approach such real-time evolution is not directly accessible: the theory is formulated in a four-dimensional box – three dimensions are spatial dimensions, the fourth is the imaginary (Euclidean) time $\tau$. The lattice temperature $T_L$ is realised through (anti)periodic boundary conditions in the Euclidean time direction – $T_L = 1/N_\tau$, where $N_\tau$ is the extent of the time direction, and can be converted to physical units once the lattice spacing is known. The spectral functions appear now in the decomposition of a (zero-momentum) Euclidean propagator $G(\tau )$: $G(\tau ) = \int _{0}^\infty \rho (\omega ) \frac{\mathrm {d}\omega }{2\pi }\, K(\tau ,\omega )$, with $K(\tau ,\omega ) = \frac{(e^{-\omega \tau } + e^{-\omega (1/T - \tau )})}{1 - e^{-\omega /T}}$. The $\tau$ dependence of the kernel *K* reflects the periodicity of the relativistic propagator in imaginary time, as well as its *T* symmetry. The Bose–Einstein distribution, intuitively, describes the wrapping around the periodic box, which becomes increasingly important at higher temperatures.

The procedure is, then, based on the generation of an appropriate ensemble of lattice gauge fields at a temperature of choice, on the computation on such an ensemble of the Euclidean propagators $G(\tau )$, and on the extraction of the spectral functions. All such quarkonium studies yield qualitatively the same result: a given quarkonium state melts at a temperature above, or possibly at, the phase-transition temperature. There is, however, disagreement between different calculations in the precise temperatures for the following reasons. First, experience with lattice calculations has demonstrated that it is extremely important to have results in the continuum limit, and with the proper matter content. This means that the masses of the dynamical quark fields which are used in the generation of the gauge ensembles must be as close as possible to the physical ones, and the lattice spacing should be fine enough to allow for making contact with continuum physics. These systematic effects, which have been studied in detail for bulk thermodynamics, are still under scrutiny for the spectral functions. Second, the calculation of spectral functions using Euclidean propagators as an input is a difficult, possibly ill-defined, problem. It has been mostly tackled by using the Maximum Entropy Method (MEM) [693], which has proven successful in a variety of applications. Recently, an alternative Bayesian reconstruction of the spectral functions has been proposed in Refs. [694, 695] and applied to the analysis of configurations from the HotQCD Collaboration [696].

Most calculations of charmonium spectral functions have been performed in the quenched approximation – neglecting quark loops –, although recently the spectral functions of the charmonium states have been studied as a function of both temperature and momentum, using as input relativistic propagators with two light quarks [697, 698] and, more recently, including the strange quark, for temperatures ranging between 0.76 and $1.9\,T_c$. The sequential dissolution of the peaks corresponding to the S- and P-wave states is clearly seen. The results are consistent with the expectation that charmonium melts at high temperature, however, as of today they lack quantitative precision and control over systematic errors.

**Fig. 76:**
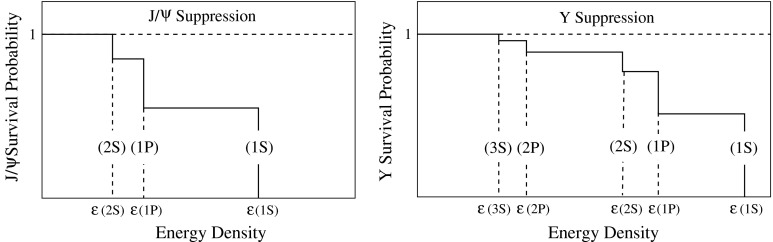
Sequential quarkonium suppression for $\mathrm {J}/\psi$ (*left*) and $\Upsilon \text {(1S)}$ (*right*) states [701]

The survival probability for a given quarkonium state depends on its size and binding energy (see Table 15 for details^26^). Hence the excited states will be dissolved at a lower initial temperature than the more tightly bound ground states. However, only a fraction (about 60 %) of the observed $\mathrm {J}/\psi$ is a directly produced $\text {(1S)}$ state, the remainder is due to the feed-down of excited states, with about 30 % from $\chi _c \text {(1P)}$ and 10 % from $\psi \text {(2S)}$ decays [699, 700]. A similar decay pattern arises for $\Upsilon$ production [200, 203, 207, 209, 441]. The decay processes occur far outside the produced medium, so that the medium affects only the excited states. As a result, the formation of a hot deconfined medium in nuclear collisions will produce a sequential quarkonium suppression pattern [701], as illustrated in Fig. 76. Increasing the energy density of the QGP above deconfinement first leads to $\psi \text {(2S)}$ dissociation, removing those $\mathrm {J}/\psi$ ’s which otherwise would have come from $\psi \text {(2S)}$ decays. Next the $\chi _c$ melts, and only for a sufficiently hot medium also the direct $\mathrm {J}/\psi$ disintegrate. For the bottomonium states, a similar pattern holds [702–705].

#### Effect of nuclear PDFs on quarkonium production in nucleus–nucleus collisions

The predictions for quarkonium suppression in AA collisions, considering only modifications of the parton densities in the nucleus, the so-called nuclear PDFs, are described in this subsection. There are other possible cold matter effects on quarkonium production in matter in addition to shadowing: break-up of the quarkonium state due to inelastic interactions with nucleons (absorption) or produced hadrons (comovers) and energy loss in cold matter, as discussed in Sect. 3. The mid-rapidity quarkonium absorption cross section for break-up by nucleon interactions decreases with centre-of-mass energy [399, 408], becoming negligible at LHC energies. In addition, cold matter suppression due to energy loss does not have a strong rapidity dependence. Thus, shadowing is expected to be the dominant cold matter effect in what concerns the modification of the shape of the quarkonium rapidity distribution. It will also produce a relatively small effect on the shape of the quarkonium $p_{\mathrm {T}}$ distribution at low $p_{\mathrm {T}}$.

**Fig. 77:**
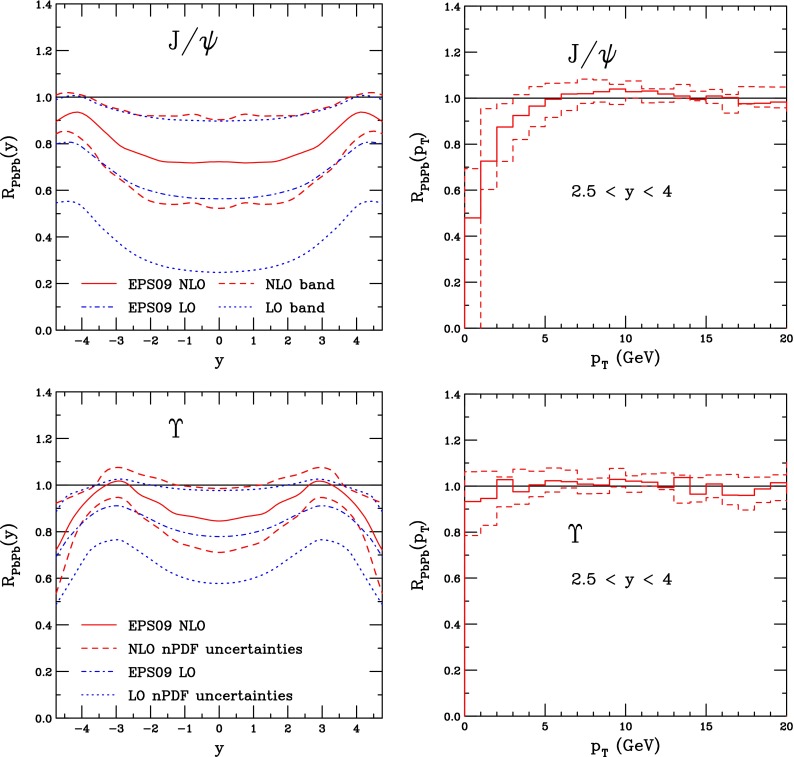
The nuclear modification factor $R_\mathrm{AA}$ for $\mathrm {J}/\psi$ (*upper*) and $\Upsilon$ (*lower*) production, calculated in the CEM model using the EPS09 modifications [364], is shown for Pb–Pb collisions at $\sqrt{s_{\mathrm{NN}}}$ $=$ 2.76  $\text {TeV}$. The results are presented as a function of rapidity (*left*) and $p_{\mathrm {T}}$ (*right*) [363]. The *dashed red histogram* shows the EPS09 NLO uncertainties. The *blue curves* show the LO modification and the corresponding uncertainty band as a function of rapidity only

**Fig. 78:**
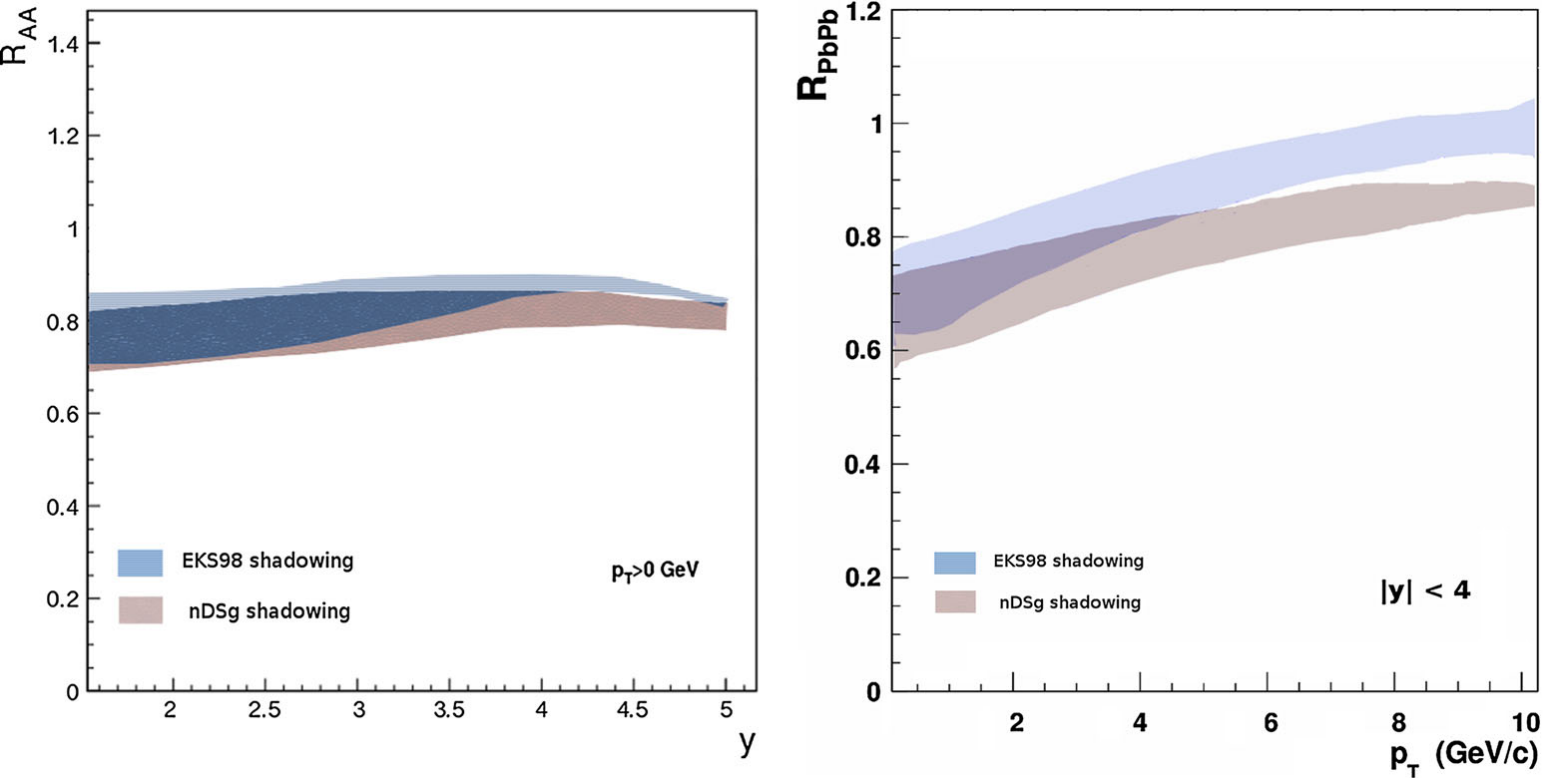
$\mathrm {J}/\psi$ rapidity (*left*) and $p_{\mathrm {T}}$ dependence (*right*) of the EKS98 LO and nDSg LO shadowing corrections performed using the CSM model according to [432, 706] in Pb–Pb collisions at $\sqrt{s_{\mathrm{NN}}}$ = 2.76 $\text {TeV}$. The *bands* for the EKS98/nDSg models shown in the figure correspond to the variation of the factorisation scale

Figure 77 shows the results for the dependence of shadowing on rapidity, transverse momentum, and centrality are shown for $$\mathrm {J}/\psi $$ and $$\Upsilon $$ production in Pb–Pb collisions at $$\sqrt{s_{\mathrm{NN}}}$$$$=$$ 2.76 $$\text {TeV}$$, neglecting absorption. Results obtained in the colour evaporation model (CEM) at next-to-leading order (NLO) in the total cross section (leading order in $$p_{\mathrm {T}}$$) are discussed first, followed by results from a leading-order colour singlet model (CSM) calculation.

The CEM calculation was described in Sect. 3. Here only a few pertinent points are repeated. In the CEM, the quarkonium production cross section in $\mathrm pp$ collisions is some fraction, $F_\Phi$, of all ${Q\overline{Q}}$ pairs below the $H \overline{H}$ threshold where *H* is the lowest-mass heavy-flavour hadron,

55 $$\begin{aligned}&\sigma _{\mathrm{PbPb}\rightarrow \Phi +X}^\mathrm{CEM}[\sqrt{s} ]\nonumber \\&\quad = A^2\, F_\Phi \sum _{i,j} \int _{4m_\mathrm{Q}^2}^{4m_H^2} \mathrm {d}\hat{s} \int _0^1 \mathrm {d}x_i \int _0^1 \mathrm {d}x_j~ R_i^\mathrm{Pb}(x_i,\mu _F^2)\nonumber \\&\quad \times \,f_i(x_i,\mu _F^2)~ R_j^\mathrm{Pb}(x_j,\mu _F^2)\,f_j(x_j,\mu _F^2)~ \mathcal{J}~{\hat{\sigma }}_{ij\rightarrow {Q\overline{Q}} +X}\nonumber \\&\quad \times [\hat{s},\mu _F^2, \mu _R^2] , \end{aligned}$$

where $ij = {q\overline{q}}$ or *gg* and ${\hat{\sigma }}_{ij\rightarrow {Q\overline{Q}} +X}$ is the $ij\rightarrow {Q\overline{Q}}$ sub-process cross section at centre-of-mass energy $\hat{s}$, while $\mathcal {J}$ is an appropriate Jacobian with dimension $1/\hat{s}$. The normalisation factor $F_\Phi$ is fitted to an appropriate subset of the available data, restricting the fits to measurements on light nuclear targets to avoid any significant cold matter effects. For the $\mathrm {J}/\psi$ and $\Upsilon$ results shown here, the normalisation $F_\Phi$ is based on the same central mass and scale parameter values as those obtained for open charm, $(m_c,\mu _F/m_c, \mu _R/m_c) = (1.27\mathrm {\,GeV}/c^2,\,2.1,\,1.6)$ [169], and beauty, $(m_b,\mu _F/m_b, \mu _R/m_b) = (4.65 \, \mathrm{GeV}/c^2, 1.4,1.1)$ [365]. The mass and scale uncertainties on the CEM calculation are shown in the previous section. They are smaller than those due to the uncertainties of the EPS09 shadowing parametrisation [364]. All the CEM calculations are NLO in the total cross section and assume that the intrinsic $k_{\mathrm {T}}$ broadening is the same in Pb–Pb as in pp.

The upper left-hand panel of Fig. 77 shows the uncertainty in the shadowing effect on $\mathrm {J}/\psi$ due to the variations in the 30 EPS09 NLO sets [364] (red). The uncertainty band calculated in the CEM at LO with the EPS09 LO sets is shown for comparison (blue). It is clear that the LO results exhibit a larger shadowing effect. This difference between the LO results, also shown in Ref. [363], and the NLO calculations arises because the EPS09 LO and NLO gluon shadowing parametrisations differ significantly at low *x* [364].

In principle, the shadowing results should be the same for LO and NLO. Unfortunately, however, the gluon modifications, particularly at low *x* and moderate $Q^2$, are not yet sufficiently constrained. The lower left panel shows the same calculation for $\Upsilon$ production. Here, the difference between the LO and NLO calculations is reduced because the mass scale, as well as the range of *x* values probed, is larger. Differences in LO results relative to, e.g., the colour singlet model arise less from the production mechanism than from the different mass and scale values assumed, as we discuss below.

It should be noted that the convolution of the two nuclear parton densities results in a $$\sim $$20 % suppression at NLO for $$|y|\le 2.5$$ with a mild decrease in suppression at more forward rapidities. The gluon anti-shadowing peak at $$|y| \sim 4$$ for $$\mathrm {J}/\psi $$ and $$|y| \sim 2$$ for $$\Upsilon $$ with large *x* in the nucleus is mitigated by the shadowing at low *x* in the opposite nucleus with the NLO parametrisation. The overall effect due to NLO nPDFs in both nuclei is a result with moderate rapidity dependence and $$R_\mathrm{AA}^{\mathrm {J}/\psi } \sim 0.7$$ for $$|y|\le 5$$ and $$R_\mathrm{AA}^{\Upsilon } \sim 0.84$$ for $$|y| \le 3$$. The nPDF effect gives more suppression at central rapidity than at forward rapidity, albeit less so for the LHC energies than for RHIC where the anti-shadowing peak at $$\sqrt{s_{\mathrm{NN}}}$$$$=$$ 200$$~\text {GeV}$$ is at $$|y| \sim 2$$. The difference between the central value of $$R_\mathrm{AA}$$ at LO and NLO is $$\sim $$30 % for the $$\mathrm {J}/\psi $$ and $$\sim $$10 % for the $$\Upsilon $$. If a different nPDF set with LO and NLO parametrisations, such as nDSg [367], is used, the difference between LO and NLO is reduced to a few percent since the difference between the underlying LO and NLO proton parton densities at low *x* is much smaller for nDSg than for EPS09 [707].

The uncertainty is larger in the LO CEM calculation for several reasons. First and foremost is the choice of the underlying proton parton densities. If the *x* and $Q^2$ dependence at LO and NLO is very different, the resulting nuclear parton densities will reflect this [707]. Other factors play a smaller role. For example, the *x* values in the $2 \rightarrow 1$ kinematics at LO is somewhat lower than the $2 \rightarrow 2$ and $2\rightarrow 3$ kinematics (for the LO+virtual and real NLO contributions respectively) of the NLO CEM calculation. Next, the $p_{\mathrm {T}}$ scale enters in the complete NLO calculation where it does not in the LO, leading to both a slightly larger *x* value for higher $p_{\mathrm {T}}$ as well as a larger scale so that the NLO calculation is on average at a higher scale than the LO.

**Fig. 79:**
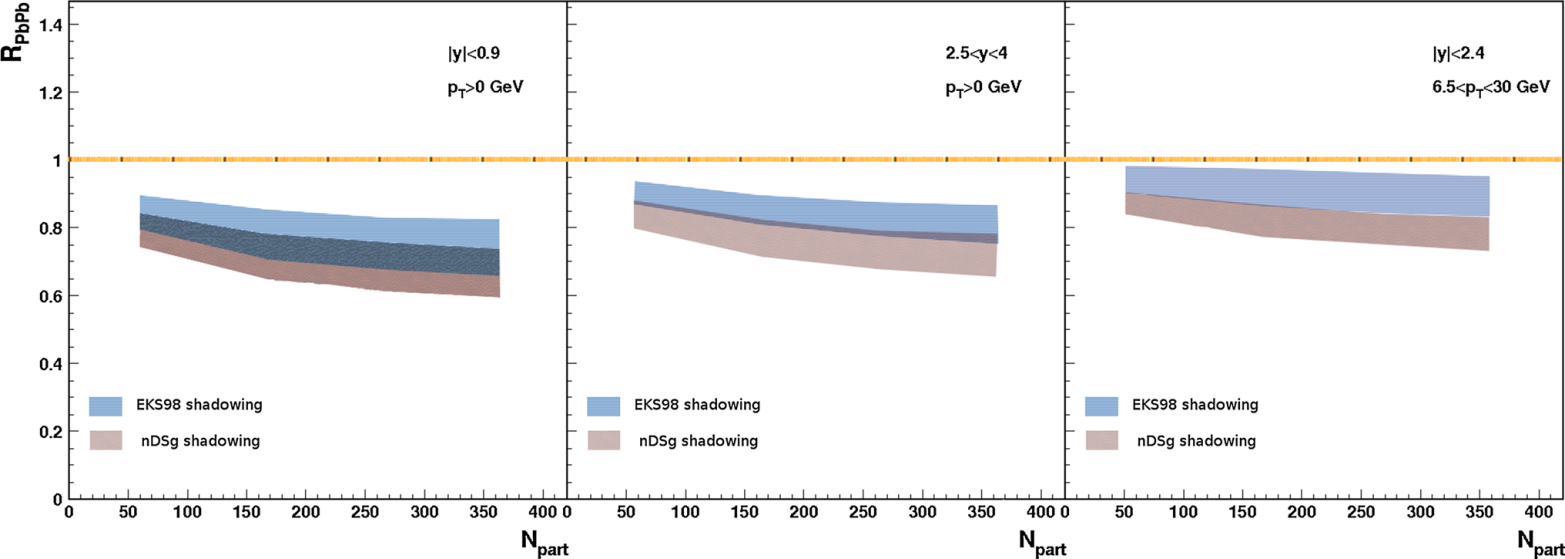
$\mathrm {J}/\psi$ centrality dependence of the EKS98 LO and nDSg LO shadowing corrections performed using the CSM model according to [432, 706] in Pb–Pb collisions at $\sqrt{s_{\mathrm{NN}}}$ $=$ 2.76 $\text {TeV}$. The *bands* for the EKS98/nDSg models shown in the figure correspond to the uncertainty in the factorisation scale

The right panels of Fig. 77 show the $p_{\mathrm {T}}$ dependence of the effect at forward rapidity for $\mathrm {J}/\psi$ (upper) and $\Upsilon$ (lower). The effect is rather mild and increases slowly with $p_{\mathrm {T}}$. There is little difference between the $\mathrm {J}/\psi$ and $\Upsilon$ results for $R_{\text {{Pb--Pb}}}(p_{\mathrm {T}})$ because, for $p_{\mathrm {T}}$ above a few GeV, the $p_{\mathrm {T}}$ scale is dominant. There is no LO comparison here because the $p_{\mathrm {T}}$ dependence cannot be calculated in the LO CEM.

However, the leading-order colour single model calculation (LO CSM) of $\mathrm {J}/\psi$ production, shown to be compatible with the magnitude of the of the $p_{\mathrm {T}}$-integrated cross sections, is a $2 \rightarrow 2$ process, $g + g \rightarrow \mathrm {J}/\psi + g$, which has a calculable $p_{\mathrm {T}}$ dependence at LO, as in the so-called *extrinsic* scheme [432].

In this approach, one can use the partonic differential cross section computed from any $2 \rightarrow 2$ theoretical model that satisfactorily describes the data down to low $p_{\mathrm {T}}$. Here, a generic $2\rightarrow 2$ matrix element which matches the $p_{\mathrm {T}}$ dependence of the data has been used and the parametrisations EKS98 LO [401] and nDSg LO [367] have been employed. The former coincides with the mid value of EPS09 LO [364]. The error bands for the EKS98 and nDSg models shown in Fig. 78 correspond to the variation of the factorisation scale ($0.5 m_\mathrm{T} < \mu _F < 2 m_\mathrm{T}$).

The spatial dependence of the nPDF has been included in this approach through a probabilistic Glauber Monte-Carlo framework, JIN [373], assuming an inhomogeneous shadowing proportional to the local density [370, 371]. Results are shown in Fig. 79.

#### Statistical (re)generation models

Over the past 20 years thorough evidence has been gathered that production of hadrons with *u*, *d*, *s*-valence quarks in heavy-ion collisions can be described using a statistical model reflecting a hadro-chemical equilibrium approach [648, 708]. Hadron yields from top AGS energy ($\sim$10$~\text {GeV}$) up to the LHC are reproduced over many orders of magnitude employing a statistical operator that incorporates a complete hadron-resonance gas. In a grand canonical treatment, the only thermal parameters are the chemical freeze-out temperature *T* and the baryo-chemical potential $\mu _b$ (and the fireball volume *V*, in case yields rather than ratios of yields are fitted). These parameters are fitted to data for every collision system as a function of collision energy. The temperature initially rises with $\sqrt{s_{\mathrm{NN}}}$ and flattens at a value of $(159\pm 2)~\text {MeV}$ close to top SPS energy, while the baryo-chemical potential drops smoothly and reaches a value compatible with zero at LHC energies. In the energy range where *T* saturates, it has been found to coincide with the (quasi-)critical temperature found in lattice QCD.

Deconfinement of quarks is expected in a QGP and for heavy quarks, in particular, this has been formulated via modification of the heavy quark potential in a process analogous to Debye screening in QED [644] (see Sect. 5.1.1). Heavy quarks are not expected to be produced thermally but rather in initial hard-scattering processes. Even at top LHC energy thermal production is only a correction at maximally the 10 % level [709]. Therefore a scenario was proposed, in which charm quarks, formed in a high energy nuclear collision in initial hard scattering, find themselves colour-screened, therefore deconfined in a QGP, and hadronise with light quarks and gluons at the phase boundary [647, 710, 711]. At hadronisation open charm hadrons as well as charmonia are formed according to their statistical weights and the mass spectrum of charmed hadrons.

Since for each beam energy the values of *T* and $\mu _b$ are already fixed by the measured light hadron yields, the only additional input needed is the initial charm production cross section per unit rapidity in the appropriate rapidity interval. The conservation of the number of charm quarks is introduced in the statistical model via a fugacity $g_c$, where all open charm hadron yields scale proportional to $g_c$, while charmonia scale with $g_c^2$ since they are formed from a charm and an anticharm quark. A logical consequence of this is that at energies below LHC energy, where the charm yield is small, charmonium production is suppressed in comparison to scaled pp collisions, while for LHC energies, the charm yield is larger and the charmonium yield is enhanced [647, 710, 711].

Already a comparison to first data on $$\mathrm {J}/\psi $$ production from PHENIX at RHIC using a charm cross section from perturbative QCD proved successful [465]. When more data became available it was found that in particular the rapidity and centrality dependence of $$\mathrm {J}/\psi $$$$R_{\mathrm {AA}}$$ from RHIC and the $$\psi \text {(2S)}$$ to $$\mathrm {J}/\psi $$ ratio from NA50 at the SPS were well reproduced by this approach [712, 713]. In order to treat properly the centrality dependence, also production in the dilute corona using the pp production cross section of $$\mathrm {J}/\psi $$ is considered [712, 713]. While it was clear that for LHC energies larger values for $$R_{\mathrm {AA}}$$ of $$\mathrm {J}/\psi $$ are expected than at RHIC, $$R_{\mathrm {AA}}$$ depends linearly on the unknown $${c\overline{c}}$$ cross section. Predictions for an expected range were given in [714].

The comparison of the statistical hadronisation predictions with the LHC data require the knowledge of the $${c\overline{c}}$$ cross section. This quantity has been measured in $$\mathrm pp$$ collisions at $$\sqrt{s}$$$$=$$ 7 $$\text {TeV}$$ and is then extrapolated to the lower Pb–Pb beam energy, i.e. $$\sqrt{s_{\mathrm{NN}}}$$$$=$$ 2.76 $$\text {TeV}$$. Since the current data are for half the LHC design energy, the open charm cross section is at the lower end of the range considered in Ref. [714]. The uncertainty on this model prediction comes from the uncertainty on the $${c\overline{c}}$$ cross section and it stems from the measurement of the $${c\overline{c}}$$ cross section itself, $$\sqrt{s}$$, and shadowing extrapolations.

As it will be discussed in Sect. 5.2.2, the statistical model reproduces the significant increase observed, for central collisions, in the $$\mathrm {J}/\psi $$$$R_{\mathrm {AA}}$$ from RHIC to the LHC (see Fig. 85).

The statistical hadronisation picture, and therefore the increase in $R_{\mathrm {AA}}$ at LHC, applies to thermalised charm quarks and, therefore, is necessarily a low $p_{\mathrm {T}}$ phenomenon. This is in line with a drop in $R_{\mathrm {AA}}$ for larger $p_{\mathrm {T}}$ observed in the data. The statistical hadronisation model in itself makes no prediction of spectra without additional input. Given a velocity distribution of the quarks at hadronisation, the spectra and their moments are fixed. As examples in Refs. [712, 713], $\mathrm {J}/\psi$ spectra are predicted for different *T* and collective expansion velocity of the medium at hadronisation. The narrowing of $\langle p_T \rangle$ and its root-mean-square as compared to $\mathrm pp$ collisions in the ALICE data are in line with this expectation. A precise measurement of the spectral shape is an important test of the model awaiting larger data samples.

**Fig. 80:**
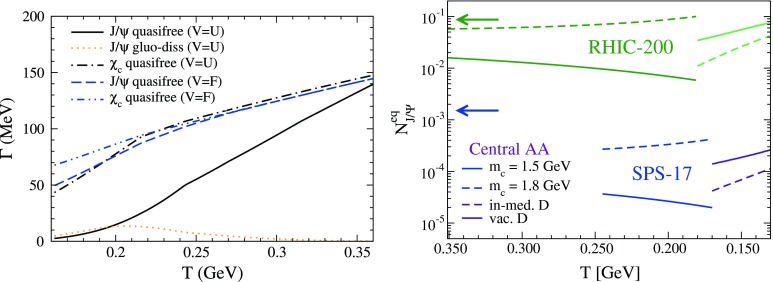
Transport coefficients of charmonia in the QGP. *Left* inelastic reaction rates for J$/\psi$ and $\chi _c$ in strong- (*V*=*U*) and weak-binding (*V*=*F*) scenarios defined in the text. *Right* $\mathrm {J}/\psi$ equilibrium numbers for conditions in central Pb–Pb and Au–Au at full SPS and RHIC energies, respectively, using different values of the in-medium *c*-quark mass in the QGP ($T\ge 180~\text {MeV}$) and for D-mesons in hadronic matter ($T\le 180~\text {MeV}$); in practice the equilibrium numbers are constructed as continuous across the transition region

Another characteristic feature of the statistical hadronisation model is an excited state population driven by Boltzmann factors at the hadronisation temperature. So far the only successful test of this prediction is the $\psi \text {(2S)}$/$\mathrm {J}/\psi$ ratio at the SPS. Data for $\psi \text {(2S)}$ and $\chi _c$ at LHC and RHIC will be crucial tests of this model and will allow one to differentiate between transport model predictions (see Sect. 5.1.4) and statistical hadronisation at the phase boundary, if measured with sufficient precision (10–20 %).

#### Transport approach for in-medium quarkonia

In the transport models, there is continuous dissociation and (re)generation of quarkonia over the entire lifetime of the deconfined stage. The space-time evolution of the phase-space distribution, $f_\mathcal{Q}$, of a quarkonium state $\mathcal{Q} = \Psi , \Upsilon$ ($\Psi$=$J/\psi , \chi _c, \dots$; $\Upsilon$=$\Upsilon (1S),\chi _b, \dots$) in hot and dense matter may be described by the relativistic Boltzmann equation,

56 $$\begin{aligned} p^\mu \partial _\mu f_\mathcal{Q}(\vec r,\tau ;\vec p)= & {} - E_p \ \Gamma _\mathcal{Q}(\vec r,\tau ;\vec p) \ f_\mathcal{Q}(\vec r,\tau ;\vec p)\nonumber \\&+ E_p \ \beta _\mathcal{Q}(\vec r,\tau ;\vec p) \ \end{aligned}$$

where $p_0=E_p=(\vec p^2 +m_\mathcal{Q}^2)^{1/2}$, $\tau$ is the proper time, and $\vec {r}$ is the spatial coordinate. $\Gamma _\mathcal{Q}$ denotes the dissociation rate^27^ and the gain term, $\beta _\mathcal{Q}$, depends on the phase-space distribution of the individual heavy (anti-)quarks, $Q=c,\,b$ in the QGP (or $\mathrm D$, $\overline{\mathrm{D}}$ mesons in hadronic matter). If the open charm states are thermalised, and in the limit of a spatially homogeneous medium, one may integrate over the spatial and three-momentum dependencies to obtain the rate equation [466, 715, 716]

57 $$\begin{aligned} \frac{dN_\mathcal{Q}}{d\tau } = -\Gamma _\mathcal{Q}(T) [ N_\mathcal{Q} - N_\mathcal{Q}^\mathrm{eq}(T) ]. \end{aligned}$$

The key ingredients to the rate equation are the *transport coefficients*: the inelastic reaction rate, $\Gamma _\mathcal{Q}$, for both dissociation and formation – *detailed balance* –, and the quarkonium equilibrium limit, $N_\mathcal{Q}^\mathrm{eq}(T)$.

The reaction rate can be calculated from inelastic scattering amplitudes of quarkonia on the constituents of the medium (light quarks and gluons, or light hadrons). The relevant processes depend on the (in-medium) properties of the bound state [717]. In the QGP, for a tightly bound state (binding energy $E_B\ge T$), an efficient process is gluo-dissociation [718], $g+\mathcal{Q}\rightarrow Q+\overline{Q}$, where all of the incoming gluon energy is available for break-up. However, for loosely bound states ($E_B <T$ for excited and partially screened states), the phase space for gluo-dissociation rapidly shuts off, rendering “quasi-free” dissociation, $p+\mathcal{Q}\rightarrow Q+\overline{Q}+p$ ($p=q,\overline{q},g$), the dominant process [717], cf. Fig. 80 (left). Gluo-dissociation and inelastic parton scattering-dissociation of quarkonia have also been studied within an EFT approach [719].

The equilibrium number densities are simply those of *Q* quarks (with spin-colour and particle-antiparticle degeneracy $6 \times 2$) and quarkonium states (summed over including their spin degeneracies $d_\mathcal{Q}$).

The quarkonium equilibrium number is given by:

58 $$\begin{aligned} N_\mathcal{Q}^\mathrm{eq}= & {} V_{\mathrm {FB}} \sum \limits _\mathcal{Q} n_\mathcal{Q}^\mathrm{eq}(m_\mathcal{Q};T,\gamma _Q)\nonumber \\= & {} V_{\mathrm {FB}} \ \sum \limits _\mathcal{Q} d_\mathcal{Q} \ \gamma _Q^2 \int \frac{d^3p}{(2\pi )^3} f_\mathcal{Q}^B(E_p;T) \ \end{aligned}$$

where $V_\mathrm{FB}$ refers to the fireball volume, $d_\mathcal{Q}$ is the spin degeneracy and $f_\mathcal{Q}^B$ corresponds to the Bose distribution.

The open heavy-flavour (HF) number, $N_\mathrm{{op}}$, follows from the corresponding equilibrium densities, e.g.

59 $$\begin{aligned} N_\mathrm{{op}}=N_\mathcal{Q}+N_{\overline{\mathcal{Q}}} = V_\mathrm{FB} 12\gamma _Q \int \frac{d^3p}{(2\pi )^3} f_{Q}^F(E_p;T) \end{aligned}$$

for heavy (anti-)quarks in the QGP.

**Fig. 81:**
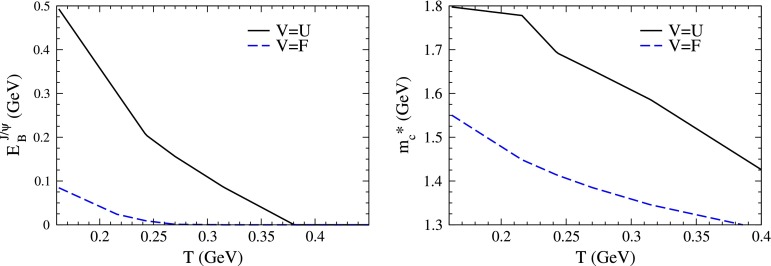
Temperature dependence of J$/\psi$ binding energy (*left panel*) and charm-quark mass (*right panel*) in the QGP in the strong- and weak-binding scenarios [*solid* (*V*=*U*) and *dashed lines* (*V*=*F*), respectively] as implemented into the rate equation approach [723]

Assuming relative chemical equilibrium between all available states containing heavy-flavoured quarks at a given temperature and volume of the system, the number of ${Q\overline{Q}}$ pairs in the fireball – usually determined by the initial hard production – is matched to the equilibrium numbers of HF states, using a fugacity factor $\gamma _Q=\gamma _{\overline{Q}}$, by the condition:

60 $$\begin{aligned} N_{{Q\overline{Q}}}=\frac{1}{2} N_\mathrm{{op}}\frac{I_1(N_\mathrm{{op}})}{I_0(N_\mathrm{{op}})}+ V_{\mathrm {FB}} \ \gamma _Q^2\sum \limits _\mathcal{Q} n_\mathcal{Q}^\mathrm{eq}(T). \end{aligned}$$

The ratio of Bessel functions above, $I_1/I_0$, enforces the canonical limit for small $N_\mathrm{op} \le 1$.

The quarkonium equilibrium limit is thus coupled to the open HF spectrum in medium; e.g., a smaller *c*-quark mass increases the *c*-quark density, which decreases $\gamma _c$ and therefore reduces $N_{J/\psi }^\mathrm{eq}$, by up to an order of magnitude for $m_c=1.8 \rightarrow 1.5$ GeV/$c^2$, cf. Fig. 80 (right).

In practice, further corrections to $N_\mathcal{Q}^\mathrm{eq}$ are needed for more realistic applications in heavy-ion collisions. First, heavy quarks cannot be expected to be thermalised throughout the course of a heavy-ion collision; harder heavy-quark (HQ) momentum distributions imply reduced phase-space overlap for quarkonium production, thus suppressing the gain term. In the rate equation approach this has been implemented through a relaxation factor $\mathcal{R} = 1-\exp (-\int \mathrm {d}\tau /\tau _Q^\mathrm{therm})$ multiplying $N_\mathcal{Q}^\mathrm{eq}$, where $\tau _Q^\mathrm{therm}$ represents the kinetic relaxation time of the HQ distributions [715, 720]. This approximation has been quantitatively verified in Ref. [721]. Second, since HQ pairs are produced in essentially point-like hard collisions, they do not necessarily explore the full volume in the fireball. This has been accounted for by introducing a correlation volume in the argument of the Bessel functions, in analogy to strangeness production at lower energies [722].

An important aspect of this transport approach is a controlled implementation of in-medium properties of the quarkonia [715, 723]. Colour-screening of the QCD potential reduces the quarkonium binding energies, which, together with the in-medium HQ mass, $m_Q^*$, determines the bound-state mass, $m_\mathcal{Q} = 2m_Q^*- E_B$. As discussed above, the interplay of $m_\mathcal{Q}$ and $m_Q^*$ determines the equilibrium limit, $N_\mathcal{Q}^\mathrm{eq}$, while $E_B$ also affects the inelastic reaction rate, $\Gamma _\mathcal{Q}(T)$. To constrain these properties, pertinent spectral functions have been used to compute Euclidean correlators for charmonia, and required to approximately agree with results from lattice QCD [723]. Two basic scenarios have been put forward for tests against charmonium data at the SPS and RHIC: a strong-binding scenario (SBS), where the J$/\psi$ survives up to temperatures of about 2 $T_c$, and a weak-binding scenario (WBS) with $T_\mathrm{diss}\simeq 1.2\,T_c$, cf. Fig. 81. These scenarios are motivated by microscopic *T*-matrix calculations [558] where the HQ internal ($U_{{Q\overline{Q}}}$) or free energy ($F_{{Q\overline{Q}}}$) have been used as potential, respectively. A more rigorous definition of the HQ potential, and a more direct implementation of the quarkonium properties from the *T*-matrix approach is warranted for future work. The effects of the hadronic phase are generally small for $\mathrm {J}/\psi$ and bottomonia, but important for the $\psi \text {(2S)}$, especially, if its direct decay channel $\psi \text {(2S)} \rightarrow \overline{\mathrm{D}}\mathrm{D}$ is opened (due to reduced masses and/or finite widths of the D mesons) [715, 720].

The rate equation approach has been extended to compute $p_{\mathrm {T}}$ spectra of charmonia in heavy-ion collisions [654]. Toward this end, the loss term was solved with a three-momentum dependent dissociation rate and a spatial dependence of the charmonium distribution function, while for the gain term blast-wave distributions at the phase transition were assumed (this should be improved in the future by an explicit evaluation of the gain term from the Boltzmann equation using realistic time-evolving HQ distributions; see Ref. [724] for initial studies) [725]. In addition, formation time effects are included, which affect quarkonium suppression at high $p_{\mathrm {T}}$  [726].

To close the quarkonium rate equations, several input quantities are required which are generally taken from experimental data in $\mathrm pp$ and p–A collisions, e.g., quarkonia and HQ production cross sections (with shadowing corrections), and primordial nuclear absorption effects encoded in phenomenological absorption cross sections. Feed-down effects from excited quarkonia (and *b*-hadron decays into charmonium) are accounted for. The space-time evolution of the medium is constructed using an isotropically expanding fireball model reproducing the measured hadron yields and their $p_{\mathrm {T}}$ spectra. The fireball resembles the basic features of hydrodynamic models [727], but an explicit use of the latter is desirable for future purposes.

Two main model parameters have been utilised to calibrate the rate equation approach for charmonia using the centrality dependence of inclusive $$\mathrm {J}/\psi $$ production in Pb–Pb collisions at the SPS ($$\sqrt{s_{\mathrm{NN}}}$$$$=$$ 17 GeV) and in Au–Au collisions at RHIC ($$\sqrt{s_{\mathrm{NN}}}$$$$=$$ 200$$~\text {GeV}$$): the strong-coupling constant $$\alpha _s$$, controlling the inelastic reaction rate, and the *c*-quark relaxation time affecting the gain term through the amended charmonium equilibrium limit. With $$\alpha _s\simeq 0.3$$ and $$\tau _c^{\text {therm}}\simeq $$ 4–6 (1.5–2) fm/*c* for the SBS (WBS), the inclusive $$\mathrm {J}/\psi $$ data at SPS and RHIC can be reasonably well reproduced, albeit with different decompositions into primordial and regenerated yields (the former are larger in the SBS than in the WBS). The $$\tau _c^{\text {therm}}$$ obtained in the SBS is in the range of values calculated microscopically from the *T*-matrix approach using the *U*-potential [558], while for the WBS it is much smaller than calculated from the *T*-matrix using the *F*-potential. Thus, from a theoretical point of view, the SBS is the preferred scenario.

With this set-up, namely the TAMU transport model, quantitative predictions for Pb–Pb collisions at the LHC ($$\sqrt{s_{\mathrm{NN}}}$$$$=$$ 2.76 TeV) have been carried out for the centrality dependence and $$p_{\mathrm {T}}$$ spectra of $$\mathrm {J}/\psi $$  [728], as well as for $$\Upsilon \text {(1S)}$$, $$\chi _b$$, and $$\Upsilon \text {(2S)}$$ production [729].

Similar results are obtained in the transport approach THU developed by the Tsinghua group [730, 731], which differs in details of the implementation, but overall asserts the robustness of the conclusions. In the THU model, the quarkonium distribution is also governed by the Boltzmann-type transport equation. The cold nuclear matter effects change the initial quarkonium distribution and heavy quark distribution at $\tau _0$. The interaction between the quarkonia and the medium is reflected in the loss and gain terms and depends on the local temperature $T(\vec {r},\tau )$ and velocity $u_\mu (\vec {r},\tau )$, which are controlled by the energy-momentum and charge conservations of the medium, $\partial _\mu T^{\mu \nu }=0$ and $\partial _\mu n^\mu =0$.

Within this approach, the centrality dependence of the nuclear modification factor $R_{\mathrm {AA}}$ can be obtained and compared to experimental results at low $p_{\mathrm {T}}$. In contrast to collisions at SPS and RHIC energies, at LHC energies the large abundance of *c* and $\overline{c}$ quarks increases their combining probability to form charmonia. Hence this regeneration mechanism becomes the dominant source of charmonium production for semi-central and central collisions at the LHC. The competition between dissociation and regeneration leads to a flat structure of the $\mathrm {J}/\psi$ yield as a function of centrality. This flat behaviour should disappear at higher energies or, regeneration being a $p_{\mathrm {T}}$-dependent mechanism, with increasing $p_{\mathrm {T}}$.

The charmonium transverse momentum distribution contains more dynamic information on the hot medium and can be calculated within the transport approach. The regeneration occurs in the fireball, and therefore the thermally produced charmonia are mainly distributed at low $p_{\mathrm {T}}$, their contribution increasing with centrality. On the other hand, those charmonia from the initial hard processes carry high momenta and dominate the high $p_{\mathrm {T}}$ region at all centralities. This different $p_{\mathrm {T}}$ behaviour of the initially produced and regenerated charmonia can even lead to a minimum located at intermediate $p_{\mathrm {T}}$. Moreover, this particular $p_{\mathrm {T}}$ behaviour will lead to an evolution of the mean transverse momentum, $\langle p_{\mathrm {T}} \rangle$, with centrality that would be higher for SPS than for LHC nuclear collisions, once normalised to the corresponding proton–proton $\langle p_{\mathrm {T}} \rangle$ [732, 733]. At the SPS, almost all the measured $\mathrm {J}/\psi$ are produced through initial hard processes and carry high momentum. At RHIC, the regeneration starts to play a role and even becomes equally important as the initial production in central collisions. At the LHC, regeneration becomes dominant, and results in a decreasing of $\langle p_{\mathrm {T}} \rangle$ with increasing centrality.

**Fig. 82:**
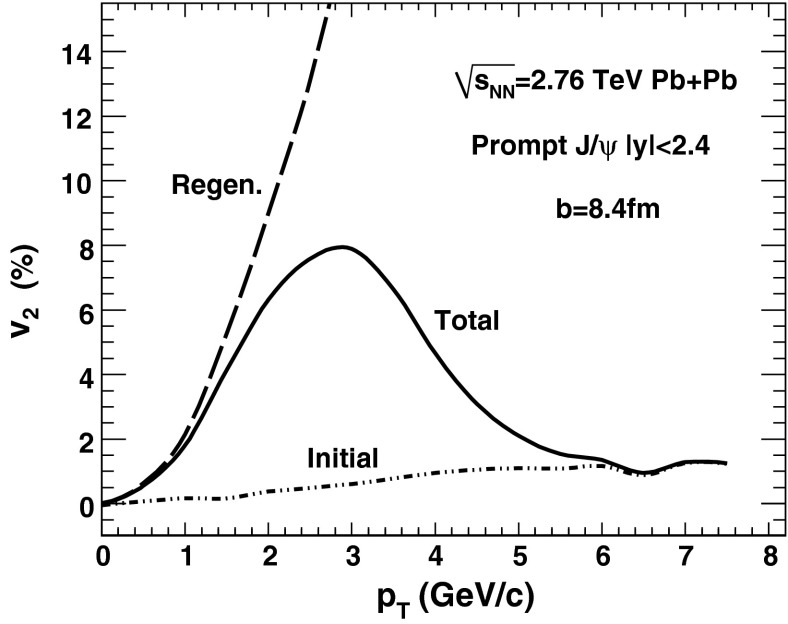
Elliptic flow $v_{2}$ for prompt $\mathrm {J}/\psi$ in Pb–Pb collisions at $\sqrt{s_{\mathrm{NN}}}$ $=$ 2.76 $\text {TeV}$ as predicted by the THU model. The calculation is with impact parameter $b = 8.4$ fm, corresponding to the 0–100 % centrality range. The *dot-dashed, dashed* and *solid lines* represent the initial, regeneration, and total contributions, respectively

Concerning the $$\mathrm {J}/\psi $$ elliptic flow, due to the strong interaction between the heavy quarks and the hot medium, the regenerated charmonia inherit collective flow from the charm quarks. Furthermore, primordial $$\mathrm {J}/\psi $$ might acquire a $$v_{2}$$ induced by a path-length dependent suppression. As shown in Fig. 82, the $$\mathrm {J}/\psi $$$$v_{2}$$ will, therefore, result from the interplay of two contributions, a regeneration component, dominant at lower $$p_{\mathrm {T}}$$ and the primordial $$\mathrm {J}/\psi $$ component that takes over at higher $$p_{\mathrm {T}}$$. Hence, given the increasing regeneration fraction with colliding energy, the $$\mathrm {J}/\psi $$ elliptic flow is expected to become sizeable at LHC while it should be almost zero at RHIC.

#### Non-equilibrium effects on quarkonium suppression

Since heavy quarkonium states have a short formation time in their rest frame ($< 1~\text {fm}/c$), they are sensitive to the early-time dynamics of the QGP. As a consequence, it is necessary to have dynamical models that can accurately describe the bulk dynamics of the QGP during the first $\text {fm}/c$ of its lifetime. This is complicated by the fact that, at the earliest times after the initial nuclear impact, the QGP is momentum-space anisotropic in the local rest frame. The existence of large QGP momentum-space anisotropies is found in both the weak and the strong coupling limits (see e.g. Ref. [734–737]). In both limits, one finds that the longitudinal pressure, $\mathcal{P}_L = T^{zz}$, is much less than the transverse pressure, $\mathcal{P}_T = (T^{xx}+T^{yy})/2$, at times smaller than $1~\text {fm}/c$. During the QGP evolution this momentum-space anisotropy relaxes to zero, but it does so only on a time scale of several $\text {fm}/c$. In addition, the momentum-space anisotropy grows larger as one approaches the transverse edge of the QGP, where the system is colder. The existence of such momentum-space anisotropies is consistent with first- and second-order viscous hydrodynamics; however, since these approaches rely on linearisation around an isotropic background, it is not clear that these methods can be applied in a far-from-equilibrium situation. In order to address this issue, a non-perturbative framework, called anisotropic hydrodynamics (aHYDRO), has been developed. This framework allows the system to be arbitrarily anisotropic [738–741].

The time-evolution provided by aHYDRO has to be folded together with the non-equilibrium (anisotropic) quarkonium rates. These were first considered in Refs. [742–746] where the effect of momentum-space anisotropy was included for both the real and imaginary parts of potential. In this context, the imaginary part of the potential plays the most important role, as it sets the in-medium decay rate of heavy quarkonium states. The calculations of the resulting decay rates in Ref. [746] demonstrated that these in-medium decay rates were large with the corresponding lifetime of the states being on the order of $\text {fm}/c$. In practice, one integrates the decay rate over the lifetime of the state in the plasma as a function of its three-dimensional position in the system and its transverse momentum. The result of this is a prediction for the $R_{\mathrm {AA}}$ that depends on the assumed shear-viscosity to entropy density ratio ($\eta /s$) of the QGP since this ratio determines the degree to which the system remains isotropic. The results obtained for the inclusive $\Upsilon \text {(1S)}$ and $\Upsilon \text {(2S)}$ suppression [747–749] have a significant dependence on the assumed value of $\eta /s$, in particular for the inclusive $\Upsilon \text {(1S)}$. This ratio can be determined from independent collective flow measurements and at the energies probed by the LHC one finds that $4\pi \,\eta /s \sim 1\text {--}3$ [748, 750]. The upper limit of this range seems to be compatible with the CMS data (the comparison will be shown in Sect. 5.2.7); however, since the model used did not include any regeneration effects, it is possible that the final $\eta /s$ could be a bit lower than three times the lower bound. Furthermore, it should be pointed out that feed-down fractions based on CDF measurements with $p_{\mathrm {T}} >8~\text {GeV}/c$ are used [209, 441], which with $\approx$50 % is larger than the fraction one would obtain when using the recent $\chi _b \text {nP}\rightarrow \Upsilon \text {(1S)}$ measurements by LHCb that extend to slightly lower $p_{\mathrm {T}}$  [203]. In the later case the total $\Upsilon \text {(1S)}$ feed-down contribution is $\approx$30 % for $p_{\mathrm {T}} >6~\text {GeV}/c$.

#### Collisional dissociation of quarkonia from final-state interactions

The model described in Sect. 4.3.3 can also be modified to describe the dissociation dynamics of quarkonia in the QGP. The model includes both initial-state cold nuclear matter energy-loss and final-state effects, such as radiative energy loss for the colour-octet state and collisional dissociation for quarkonia, as they traverse the created hot medium. The main differences with respect to the formalism discussed in Sect. 4.3.3 are (a) that once a high-$p_{\mathrm {T}}$ quarkonium is dissociated, it is unlikely that it will fragment again to form a new quarkonium, (b) the formation time is given not by fragmentation dynamics but by binding energies. A self-consistent description of the formation of a quarkonium in a thermal QGP is a challenging problem [83] and assumes that the formation time lies between $1/(2E_b)$ and $1/(E_b)$, and that the wave function does not show significant thermal effects in this short time.

When compared to the $$\mathrm {J}/\psi $$$$R_{\mathrm {AA}}$$ results, obtained by the CMS experiment, the model is consistent with the observations for the peripheral events, but underestimates the suppression for the most central events, suggesting that thermalisation effects on the wave functions may be substantial.

#### Comover models

The comover interaction model (CIM) was originally developed in the 1990s in order to explain both the suppression of charmonium yields and the strangeness enhancement in nucleus–nucleus collisions at the SPS [751–753]. It includes the initial-state nuclear effects, the so-called nuclear shadowing. It takes into account the quarkonium dissociation due to interactions with the comoving medium and the recombination of ${Q\overline{Q}}$ into secondary quarkonium states. It is based on the well-known gain and loss differential equations in transport theory for a quarkonium state $\mathcal {Q}$:

61 $$\begin{aligned}&\tau \frac{\mathrm {d}N_\mathcal{Q}}{\mathrm {d}\tau }\left( b,s,y \right) = -\sigma _{\text {co}} \nonumber \\&\quad \times [ N^{\text {co}}(b,s,y) N_\mathcal{Q}(b,s,y) \,-\, N_Q(b,s,y) N_{\overline{Q}}(b,s,y) ],\nonumber \\ \end{aligned}$$

as a function of impact parameter *b*, centre-of-mass energy squared *s*, and rapidity *y*. The first term refers to the quarkonium dissociation and the second term takes care of the recombination of ${Q\overline{Q}}$ into secondary quarkonium states. The variable $\sigma _{\text {co}}$ denotes the cross section of quarkonium dissociation due to interactions with the comoving medium, with density $N^{\text {co}}$.

Assuming a dilution in time of the densities due to longitudinal motion, which leads to a $\tau ^{-1}$ dependence on proper time, the approximate solution of Eq. (61) gives the survival probability:

62 $$\begin{aligned}&S^{\text {co}}(b,s,y) \nonumber \\&\quad =\exp \left\{ -\sigma _{\text {co}} \left[ N^{\text {co}}(b,s,y)\,-\, \frac{N_Q(b,s,y) N_{\overline{Q}} (b,s,y)}{N_\mathcal{Q}(b,s,y)} \right] \right. \nonumber \\&\qquad \times \left. \ln \left[ \frac{N^{\text {co}}(b,s,y)}{N_\mathrm{pp} (y)}\right] \right\} . \end{aligned}$$

Using the inverse proportionality between proper time and densities,i.e. $\tau _f/ \tau _0= N^{\text {co}}(b, s, y)/N_\mathrm{pp}(y)$ – the interaction stops when the densities have diluted, reaching the value of the $\mathrm pp$ density at the same energy – it can be concluded that the result depends only on the ratio $\tau _f/ \tau _0$ of final over initial time.

#### Summary of theoretical models for experimental comparison

Different theoretical models are available for comparison. Among them, the statistical hadronisation model, the transport model, the collisional dissociation model, and the comover model will be compared to charmonium experimental results in the next section. Their principal characteristics can be summarised as follows.

In the statistical hadronisation model, the charm (beauty) quarks and antiquarks, produced in initial hard collisions, thermalise in a QGP and form hadrons at chemical freeze-out. It is assumed that no quarkonium state survives in the deconfined state (full suppression) and, as a consequence, also CNM effects are not included in this model. An important aspect in this scenario is the canonical suppression of open charm or beauty hadrons, which determines the centrality dependence of production yields in this model. The overall magnitude is determined by the input charm (beauty) production cross section.

Kinetic (re)combination of heavy quarks and antiquarks in a QGP provides an alternative quarkonium production mechanism. In transport models, there is continuous dissociation and (re)generation of quarkonia over the entire lifetime of the deconfined state. A hydrodynamical-like expansion of the fireball of deconfined matter, constrained by data, is part of such models, alongside an implementation of the screening mechanism with inputs from lattice QCD. Other important ingredients are parton-level cross sections. Cold nuclear matter effects are incorporated by means of an overall effective absorption cross section that accounts for (anti-)shadowing, nuclear absorption, and Cronin effects.

The collisional dissociation model considers, in addition to modifications of the binding potential by the QGP and cold nuclear matter effects, radiative energy loss of the colour octet quarkonium precursor and collisional dissociation processes inside the QGP.

Similarly, the comover interaction model includes dissociation of quarkonia by interactions with the comoving medium of hadronic and partonic origin. Regeneration reactions are also included. Their magnitude is determined by the production cross section of ${c\overline{c}}$ pairs and quarkonium states. Cold nuclear matter effects are taken into account by means of (anti-)shadowing models.

Summarising:

- Statistical hadronisation assumes full suppression of primordial quarkonia and regeneration at the phase boundary.
- Transport models include cold nuclear absorption, direct suppression, and regeneration.
- Collisional dissociation models include initial-state cold nuclear matter effects and final-state effects based on radiative energy loss and collisional dissociation.
- Comover models include shadowing, interaction with comoving medium, and regeneration.

In transport and comover models, at LHC energies, a large fraction of $$\mathrm {J}/\psi $$ ($$>$$50 % in most central collisions) is produced by charm quark recombination. In the statistical hadronisation model, all $$\mathrm {J}/\psi $$ are generated at the hadronisation stage by purely statistical mechanisms. In order to include (re)generation, a cross section $$\mathrm {d}\sigma _\mathrm{pp}^{{c\overline{c}}}/\mathrm {d}y \approx 0.6\text {--}0.8$$ mb at mid-rapidity at $$\sqrt{s_{\mathrm{NN}}}$$$$=$$ 2.76 $$\text {TeV}$$ has been considered in the transport and comover models. It corresponds to $$\sigma _\mathrm{pp}^{{c\overline{c}}}$$ around 5 mb, which agrees with experimental data (see Fig. 1). Currently available data, however, offer only very little constraints at 2.76 $$\text {TeV}$$ due to the lack of D-meson measurements at $$p_{\mathrm {T}} <2~\text {GeV}/c $$ in Pb–Pb collisions. The used value is about a factor of two higher than the one used in the statistical hadronisation model. Note nevertheless that there is no contradiction, since in the latter the initial-state shadowing is not modelled. The choice of smaller cross section in $$\mathrm pp$$, $$\mathrm {d}\sigma _\mathrm{pp}^{{c\overline{c}}}/\mathrm {d}y \approx 0.3\text {--}0.4$$ mb, takes into account a shadowing effect that reduces the charm cross section in Pb–Pb by up to a factor of two.

In order to compare with experimental data on bottomonium, also the hydrodynamical formalism assuming finite local momentum-space anisotropy due to finite shear viscosity will be considered. The main ingredients are: a screened potential, an hydrodynamical-like evolution of the QGP, and feed-down from higher-mass states. Neither cold nuclear matter effects nor recombination are included.

### Experimental overview of quarkonium results at RHIC and LHC

#### Proton–proton collisions as a reference for $R_{\mathrm {AA}}$ at the LHC

The medium effects on quarkonia are usually quantified via the nuclear modification factor $R_{\mathrm {AA}}$, basically comparing the quarkonium yields in AA to the $\mathrm pp$ ones. A crucial ingredient for the $R_{\mathrm {AA}}$ evaluation is, therefore, $\sigma _\mathrm{pp}$, the quarkonium production cross section in $\mathrm pp$ collisions measured at the same energy as the AA data.

During LHC Run 1, $$\mathrm pp$$ data at $$\sqrt{s}$$$$=$$ 2.76  $$\text {TeV}$$ were collected in two short data taking periods in 2011 and 2013. When the collected data sample was large enough, the quarkonium $$\sigma _\mathrm{pp}$$ was experimentally measured, otherwise an interpolation of results obtained at other energies was made.

More in detail, the $$\mathrm {J}/\psi $$ cross section ($$\sigma _\mathrm{pp}^{\mathrm {J}/\psi }$$) adopted by ALICE for the forward rapidity $$R_{\mathrm {AA}}$$ results is based on the 2011 $$\mathrm pp$$ data taking. The $$\mathcal {L} _{\text {int}} = 19.9\text {~nb}^{-1} $$ integrated luminosity, corresponding to $$1364\pm 53$$$$\mathrm {J}/\psi $$ reconstructed in the dimuon decay channel, allows for the extraction of both the integrated as well as the $$p_{\mathrm {T}}$$ and *y* differential cross sections [412]. The statistical uncertainty is 4 % for the integrated result, while it ranges between 6 and 20 % for the differential measurement. Systematic uncertainties are $$\sim $$8 %. The collected data ($$\mathcal {L} _{\text {int}} = 1.1\text {~nb}^{-1} $$) allow for the evaluation of $$\sigma _\mathrm{pp}^{\mathrm {J}/\psi }$$ also in the ALICE mid-rapidity region, where $$\mathrm {J}/\psi $$ are reconstructed through their dielectron decay. The measurement is, in this case, affected by larger statistical and systematic uncertainties, of about 23 and 18 %, respectively. Therefore, the $$\sigma _\mathrm{pp}^{\mathrm {J}/\psi }$$ reference for the $$R_{\mathrm {AA}}$$ result at mid-rapidity was obtained performing an interpolation based on mid-rapidity results from PHENIX at $$\sqrt{s}$$$$=$$ 0.2 $$\text {TeV}$$  [411], CDF at $$\sqrt{s}$$$$=$$ 1.96 $$\text {TeV}$$  [414], and ALICE at $$\sqrt{s}$$$$=$$ 2.76 [412] and 7 $$\text {TeV}$$  [413]. The interpolation is done by fitting the data points with several functions assuming a linear, an exponential, a power law, or a polynomial $$\sqrt{s}$$-dependence. The resulting systematic uncertainty is, in this case, 10 %, i.e. smaller than the one obtained directly from the data at $$\sqrt{s}$$$$=$$ 2.76 $$\text {TeV}$$.

The $$\mathrm {J}/\psi $$$$\mathrm pp$$ cross section used as a reference for the $$R_{\mathrm {AA}}$$ measurements obtained by CMS is based on the results extracted from the data collected at $$\sqrt{s}$$$$=$$ 2.76 $$\text {TeV}$$ in 2011, corresponding to an integrated luminosity $$\mathcal {L} _{\text {int}} = 231\text {~nb}^{-1} $$ [482]. The number of prompt and non-prompt $$\mathrm {J}/\psi $$ in the range $$|y|<2.4$$ and $$6.5<p_{\mathrm {T}} <30~\text {GeV}/c $$ are $$830\pm 34$$ and $$206\pm 20$$, respectively. The systematic uncertainty on the signal extraction varies between 0.4 and 6.2 % for prompt $$\mathrm {J}/\psi $$ and 5 and 20 % for non-prompt $$\mathrm {J}/\psi $$. Since the adopted reconstruction procedure is the same in $$\mathrm pp$$ and Pb–Pb collisions, many of the reconstruction-related systematic uncertainties cancel when $$R_{\mathrm {AA}}$$ is computed.

**Fig. 83:**
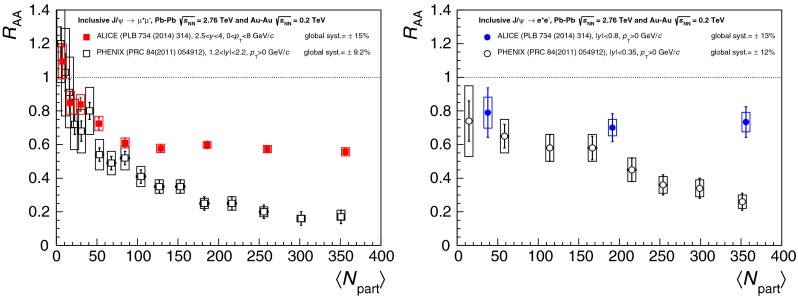
ALICE [679, 680] (*closed symbols*) and PHENIX [668] (*open symbols*) inclusive $\mathrm {J}/\psi$ nuclear modification factor versus the number of participant nucleons, at forward rapidity (*left*) and at mid-rapidity (*right*)

**Fig. 84:**
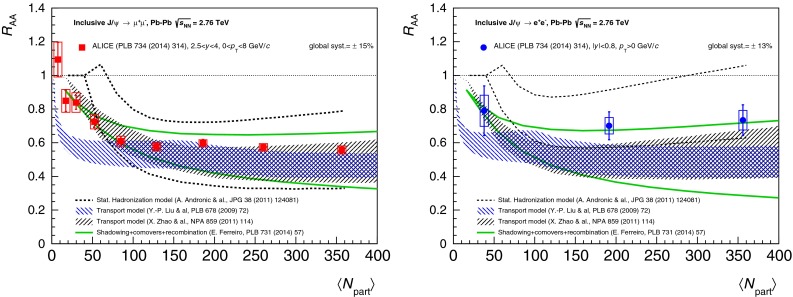
Comparison of the ALICE $\mathrm {J}/\psi$ $R_{\mathrm {AA}}$ at forward rapidity (*left*) and mid-rapidity (*right*) with the theory predictions based on the TAMU (Zhao et al.) and THU (Liu et al.) transport models discussed in Sect. 5.1.4. *Bands* correspond to the uncertainty associated to the model, i.e. to the variation of the charm cross section for the THU model and a variation of the shadowing amount for the TAMU approach. Predictions from the statistical model discussed in Sect. 5.1.3 (Andronic et al.) are also shown. The *two curves* correspond, in this case, to two assumptions on the values of the $d\sigma _{c\bar{c}}/dy$ cross sections. Calculations based on the comover model (Ferreiro), presented in Sect. 5.1.7, are included in the plot. The *lower and upper curves* correspond to variations of the charm cross section

The limited size of the $\mathrm pp$ data sample at $\sqrt{s}$ = 2.76 $\text {TeV}$ has not allowed ALICE to measure the $\Upsilon$ cross section. The reference adopted by ALICE for the $R_{\mathrm {AA}}$ studies [683] is, in this case, based on the $\mathrm pp$ measurement by LHCb [419]. However, since the LHCb result is obtained in a rapidity range ($2<y<4.5$) not exactly matching the ALICE one ($2.5<y<4$), the measurement is corrected through a rapidity interpolation based on a Gaussian shape.

For the $$\Upsilon $$$$R_{\mathrm {AA}}$$, CMS results are based on the $$\mathrm pp$$ reference cross section extracted from $$\mathrm pp$$ data at $$\sqrt{s}$$ = 2.76 $$\text {TeV}$$  [482]. The number of $$\Upsilon \text {(1S)}$$ with $$|y|<2.4$$ and $$0<p_{\mathrm {T}} <20~\text {GeV}/c $$ is $$101\pm 12$$, with a systematic uncertainty on the signal extraction of $$\sim $$10 %.

In Table 16 the datasets and the approach adopted for the evaluation of the $\mathrm pp$ reference are summarised.

**Table 16:** Overview of the $\mathrm pp$ datasets and approaches adopted for the evaluation of the $\sigma _\mathrm{pp}$ production cross section for the quarkonium states under study

	ALICE	CMS
$\mathrm {J}/\psi$	Forward-*y*: $\sigma ^{\mathrm {J}/\psi }_\mathrm{pp}$ from $\mathrm pp$ data at $\sqrt{s}$ = 2.76 $\text {TeV}$	$\sigma ^{\mathrm {J}/\psi }_\mathrm{pp}$ from $\mathrm pp$ data at $\sqrt{s}$ = 2.76 $\text {TeV}$
	Mid-*y*: $\sigma ^{\mathrm {J}/\psi }_\mathrm{pp}$ from interpolation of ALICE, CDF and PHENIX data	
$\Upsilon$	$\sigma ^{\Upsilon }_\mathrm{pp}$ from LHCb $\mathrm pp$ data at $\sqrt{s}$ = 2.76 $\text {TeV}$ + *y*-interpolation	$\sigma ^{\Upsilon }_\mathrm{pp}$ from $\mathrm pp$ data at $\sqrt{s}$ = 2.76 $\text {TeV}$

#### $$\mathrm J/\psi $$$$R_{\mathrm {AA}}$$ results at low $$p_{\mathrm {T}}$$

The experiments ALICE at the LHC and PHENIX and STAR at RHIC measure the inclusive $\mathrm {J}/\psi$ production (prompt $\mathrm {J}/\psi$ plus those coming from *b*-hadron decays) in the low $p_{\mathrm {T}}$ region, down to $p_{\mathrm {T}} = 0$. STAR measures $\mathrm {J}/\psi$ reconstructed from their $e^{+}e^{-}$ decay at mid-rapidity ($|y| <$ 1), while PHENIX detects charmonia in two rapidity ranges: at mid-rapidity ($|y|<0.35$) in the $e^{+}e^{-}$ decay channel and at forward rapidity ($1.2<|y|<2.2$) in the $\mu ^{+}\mu ^{-}$ decay channel. Similarly, ALICE studies the inclusive $\mathrm {J}/\psi$ production in the $e^{+}e^{-}$ decay channel at mid-rapidity ($|y|<0.9$) and in the $\mu ^{+}\mu ^{-}$ decay channel at forward rapidity ($2.5<y<4$). A summary of the main experimental results, together with their kinematic coverage and references, is given in Tables 13 and  14. The experiments have investigated the centrality dependence of the $\mathrm {J}/\psi$ nuclear modification factor measured in AA collisions, i.e. Au–Au at $\sqrt{s_{\mathrm{NN}}}$ = 200$~\text {GeV}$ for PHENIX [668] and STAR [676] and Pb–Pb at $\sqrt{s_{\mathrm{NN}}}$ = 2.76 $\text {TeV}$ in the ALICE case [679, 680]. As an example, PHENIX and ALICE results are shown in Fig. 83 for the forward (left) and the mid-rapidity (right) regions. While the RHIC results show an increasing suppression towards more central collisions, the ALICE $R_{\mathrm {AA}}$ has a flatter behaviour both at forward and at mid-rapidity. In the two *y* ranges there is clear evidence for a smaller suppression at the LHC than at RHIC.

Partonic transport models that include a (re)generation process for $$\mathrm {J}/\psi $$ due to the (re)combination of $${c\overline{c}}$$ pairs along the history of the collision indeed predict such a behaviour [728, 731, 753], the smaller suppression at the LHC being due to the larger $${c\overline{c}}$$ pair multiplicity which compensates the suppression from colour screening in the deconfined phase. The $$R_{\mathrm {AA}}$$ centrality dependence was predicted by the TAMU and THU transport models, discussed in Sect. 5.1.4. For both models, (re)generation becomes the dominant source for charmonium production for semi-central and central collisions and the competition between the dissociation and (re)generation mechanisms leads to the observed flat structure of the $$\mathrm {J}/\psi $$$$R_{\mathrm {AA}}$$ as a function of centrality. The comparison of the predictions of the two transport models with the ALICE data is shown in Fig. 84 for the forward (left) and mid-rapidity (right) regions.

**Fig. 85:**
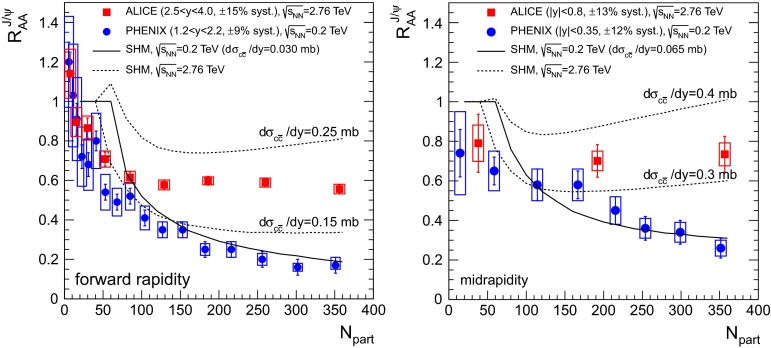
$\mathrm {J}/\psi$ $R_{\mathrm {AA}}$ from ALICE [679] and PHENIX [668] compared to predictions from the statistical hadronisation model [754]

**Fig. 86:**
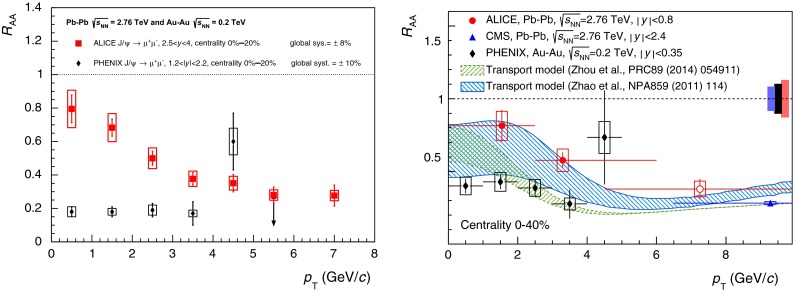
ALICE inclusive $\mathrm {J}/\psi$ $R_{\mathrm {AA}}$ versus $p_{\mathrm {T}}$  [679]. *Left* forward-rapidity result compared to the PHENIX result [668] in a similar rapidity region. Both results are obtained in the 0–20 % most central collisions. *Right* mid-rapidity result [480] compared to the PHENIX result [668], both evaluated in the 0–40 % most central collisions

A similar behaviour is expected by the statistical model [754], discussed in Sect. 5.1.3, where the $$\mathrm {J}/\psi $$ yield is completely determined by the chemical freeze-out conditions and by the abundance of $$c\overline{c}$$ pairs. In Figs. 84 and 85, the statistical model predictions are compared to the ALICE $$R_{\mathrm {AA}}$$ in the two covered rapidity ranges. As discussed in Sect. 5.1.3, a crucial ingredient in this approach is the $${c\overline{c}}$$ production cross section: the error band in the figures stems from the measurement of the $${c\overline{c}}$$ cross section itself and from the correction introduced to take into account the $$\sqrt{s}$$ extrapolation to evaluate the cross section at the Pb–Pb energy ($$\sqrt{s_{\mathrm{NN}}}$$ = 2.76 $$\text {TeV}$$). In Fig. 85 the RHIC data [668] and the corresponding statistical model calculations are also shown. Inspecting Fig. 85, for central collisions a significant increase in the $$\mathrm {J}/\psi $$$$R_{\mathrm {AA}}$$ at LHC as compared to RHIC is visible and well reproduced by the statistical hadronisation model. In particular, as a characteristic feature of the model, the shape as a function of centrality is entirely given by the charm cross section at a given energy and rapidity and is well reproduced both at RHIC and LHC. This applies also to the maximum in $$R_{\mathrm {AA}}$$ at mid-rapidity due to the peaking of the charm cross section there.

The (re)combination or the statistical hadronisation process are expected to be dominant in central collisions and, for kinematical reasons, they should contribute mainly at low $$p_{\mathrm {T}}$$, becoming negligible as the $$\mathrm {J}/\psi $$$$p_{\mathrm {T}}$$ increases. This behaviour is investigated by further studying the $$R_{\mathrm {AA}}$$$$p_{\mathrm {T}}$$-dependence. In Fig. 86, the ALICE $$\mathrm {J}/\psi $$$$R_{\mathrm {AA}}$$ ($$p_{\mathrm {T}}$$), measured at forward rapidity (left) or at mid-rapidity [480] (right), are compared to corresponding PHENIX results obtained in similar rapidity ranges. The forward-rapidity result has been obtained in the centrality class 0–20 %, while the mid-rapidity one in 0–40 %. In both rapidity regions, a striking different pattern is observed: while the ALICE $$\mathrm {J}/\psi $$$$R_{\mathrm {AA}}$$ shows a clear decrease from low to high $$p_{\mathrm {T}}$$, the pattern observed at low energies is rather different, being almost flat versus $$p_{\mathrm {T}}$$, with a suppression up to a factor four (two) stronger than at LHC at forward rapidity (mid-rapidity).

**Fig. 87:**
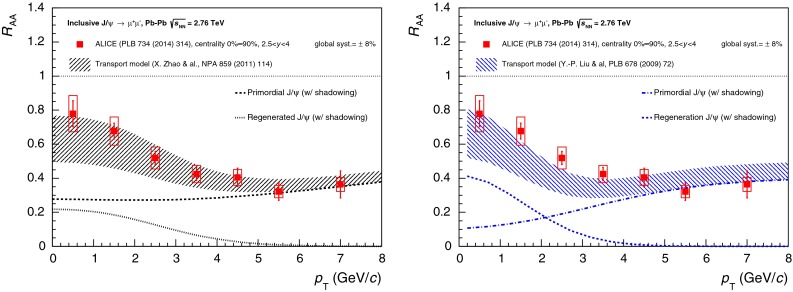
ALICE inclusive $\mathrm {J}/\psi$ $R_{\mathrm {AA}}$, measured in the forward rapidity region, versus $p_{\mathrm {T}}$  [679], compared to the TAMU (*left*) and THU (*right*) theoretical transport calculations including a (re)combination component to the $\mathrm {J}/\psi$ production

Models, such as TAMU and the THU that include a $p_{\mathrm {T}}$-dependent contribution from (re)combination, amounting to $\approx$50 % at low $p_{\mathrm {T}}$ and vanishing for high $p_{\mathrm {T}}$  [728, 731], are found to provide, also in this case, a reasonable description of the data, as can be observed in Fig. 87 for the forward-rapidity result or in Fig. 86 (right) for the mid-rapidity one.

**Fig. 88:**
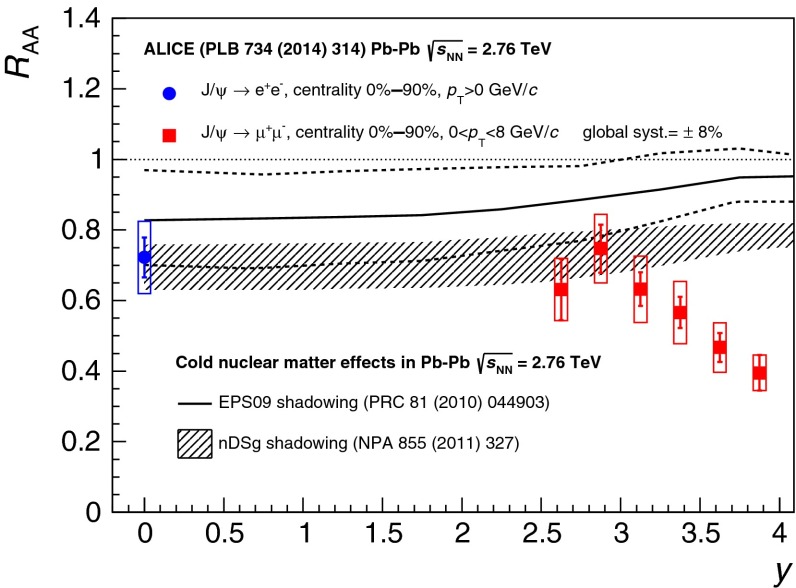
ALICE inclusive $\mathrm {J}/\psi$ $R_{\mathrm {AA}}$ versus rapidity [679], compared to nPDF calculations (see Sect. 5.1.2)

Finally, the rapidity dependence of the $$\mathrm {J}/\psi $$$$R_{\mathrm {AA}}$$ is shown in Fig. 88. At forward-*y* the $$\mathrm {J}/\psi $$$$R_{\mathrm {AA}}$$ decreases by about 40 % from $$y = 2.5$$ to $$y = 4$$. The $$R_{\mathrm {AA}}$$*y*-dependence is compared to shadowing calculations discussed in Sect. 5.1.2. As expected, the contribution of cold nuclear matter alone, such as shadowing, cannot account for the observed suppression, clearly indicating the need of the aforementioned hot matter effects.

As discussed, the ALICE results are for inclusive $$\mathrm {J}/\psi $$, therefore including two contributions: the first one from $$\mathrm {J}/\psi $$ direct production and feed-down from higher charmonium states and the second one from $$\mathrm {J}/\psi $$ originating from *b*-hadron decays. Beauty hadrons decay mostly outside the fireball, hence the measurement of non-prompt $$\mathrm {J}/\psi $$$$R_{\mathrm {AA}}$$ is mainly connected to the *b* quark in-medium energy loss, discussed in Sect. 4.1.3. Non-prompt $$\mathrm {J}/\psi $$ are, therefore, expected to behave differently with respect to the prompt ones. In the low-$$p_{\mathrm {T}}$$ region covered by ALICE the fraction of non-prompt $$\mathrm {J}/\psi $$ is smaller than 15 % [755] (slightly depending on the *y* range). Based on this fraction, the ALICE Collaboration has estimated the influence of the non-prompt contribution on the measured inclusive $$R_{\mathrm {AA}}$$. At mid-rapidity the prompt $$\mathrm {J}/\psi $$$$R_{\mathrm {AA}}$$ can vary within $$-7$$ and $$+17$$ % with respect to the inclusive $$\mathrm {J}/\psi $$$$R_{\mathrm {AA}}$$ assuming no suppression ($$R_{\mathrm {AA}} ^{\mathrm{non-prompt}}=1$$) or full suppression ($$R_{\mathrm {AA}} ^{\mathrm{non-prompt}}=0$$) for beauty, respectively. At forward-*y*, the prompt $$\mathrm {J}/\psi $$$$R_{\mathrm {AA}}$$ would be 6 % lower or 7 % higher than the inclusive result in the two aforementioned cases [679].

#### $$\mathrm J/\psi $$$$R_{\mathrm {AA}}$$ results at high $$p_{\mathrm {T}}$$

The CMS experiment is focussed on the study of the $\mathrm {J}/\psi$ production at high $p_{\mathrm {T}}$. The limit in the charmonium acceptance at low-$p_{\mathrm {T}}$ is due to the fact that muons from the charmonium decay need a minimum momentum ($p\approx 3\text {--}5~\text {GeV}/c$) to reach the muon tracking stations, overcoming the strong CMS magnetic field (3.8 T) and the energy loss in the magnet and its return yoke. The CMS vertex reconstruction capabilities allow for the separation of non-prompt $\mathrm {J}/\psi$ from *b*-hadron decays from prompt $\mathrm {J}/\psi$, using the reconstructed decay vertex of the $\mu ^{+}\mu ^{-}$ pair. The prompt $\mathrm {J}/\psi$ include directly produced $\mathrm {J}/\psi$ as well as those from decays of higher charmonium states (e.g. $\psi \text {(2S)}$ and $\chi _c$), which cannot be removed because their decay lengths are orders of magnitude smaller compared to those from *b* decays, and not distinguishable in the analysis of the Pb–Pb data.

As discussed in Sect. 5.2.1, the $\mathrm pp$ reference sample, recorded in 2011 at the same centre-of-mass energy per nucleon–nucleon pair as the Pb–Pb data, was used to evaluate the Pb–Pb $R_{\mathrm {AA}}$.

**Fig. 89:**
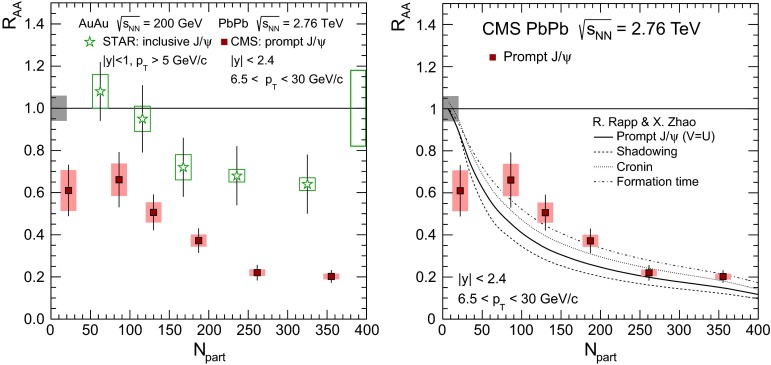
$\mathrm {J}/\psi$ $R_{\mathrm {AA}}$ as a function of centrality. *Left* CMS high-$p_{\mathrm {T}}$ prompt $\mathrm {J}/\psi$, $6.5<p_{\mathrm {T}} <30~\text {GeV}/c$ and $|y|<2.4$ (*squares*) [482], and STAR inclusive $\mathrm {J}/\psi$ with $p_{\mathrm {T}} >5~\text {GeV}/c$ measured in $|y|<1$ (*open stars*) [239]; *right* CMS high-$p_{\mathrm {T}}$ prompt $\mathrm {J}/\psi$ compared to the TAMU transport model calculation discussed in Sect. 5.1.4

**Fig. 90:**
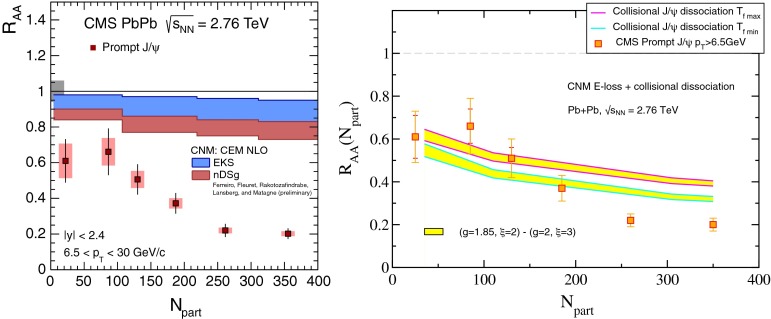
CMS prompt $\mathrm {J}/\psi$ $R_{\mathrm {AA}}$ as a function of centrality [482], compared to the nPDF calculations discussed in Sect. 5.1.2 (*left*) and to the collisional dissociation model described in Sect. 5.1.6 (*right*)

The $$\mathrm {J}/\psi $$$$R_{\mathrm {AA}}$$ was evaluated in the Pb–Pb data sample collected in 2010, corresponding to $$\mathcal {L} _{\text {int}} = 7.3~\mu \text {b}^{-1} $$. The nuclear modification factor, integrated over the rapidity range $$|y|<2.4$$ and $$p_{\mathrm {T}}$$ range $$6.5<p_{\mathrm {T}} <30~\text {GeV}/c $$, was measured in six centrality bins [482], starting with the 0–10 % bin (most central), up to the 50–100 % bin (most peripheral). The $$R_{\mathrm {AA}}$$ obtained for prompt $$\mathrm {J}/\psi $$, when integrating over the $$p_{\mathrm {T}}$$ range $$6.5<p_{\mathrm {T}} <30~\text {GeV}/c $$ and $$|y|<2.4$$, is shown in Fig. 89 ( left). The same centrality dependence, with a smooth decrease towards most central collisions, is observed also for inclusive $$\mathrm {J}/\psi $$, even if the suppression is slightly more important for prompt $$\mathrm {J}/\psi $$. In both cases the $$\mathrm {J}/\psi $$$$R_{\mathrm {AA}}$$ is still suppressed even in the (rather wide) most peripheral bin. A more recent analysis, based on the larger 2011 Pb–Pb data sample ($$\mathcal {L} _{\text {int}} = 150 ~\mu \text {b}^{-1} $$), has allowed one to study the $$R_{\mathrm {AA}}$$ in a much narrower centrality binning (12 centrality bins) and confirms the observed pattern [494].

In the left panel of Fig. 89, a comparison is made with the inclusive $\mathrm {J}/\psi$ measurement from the STAR Collaboration [239], at a more than ten times smaller collision energy, but in a similar high-$p_{\mathrm {T}}$ kinematic region: $p_{\mathrm {T}} >5~\text {GeV}/c$ and $|y|<1$. The rightmost bin corresponds to 0–10 % centrality, while the leftmost bin to 40–60 % centrality. The suppression is smaller at RHIC than at LHC energies, with no significant suppression for collisions with a centrality more peripheral than 30 % in the RHIC case. These results seem to support a higher medium temperature reached in Pb–Pb collisions at $\sqrt{s_{\mathrm{NN}}}$ = 2.76 $\text {TeV}$ collisions than in Au–Au collisions at $\sqrt{s_{\mathrm{NN}}}$ = 0.2 $\text {TeV}$.

**Fig. 91:**
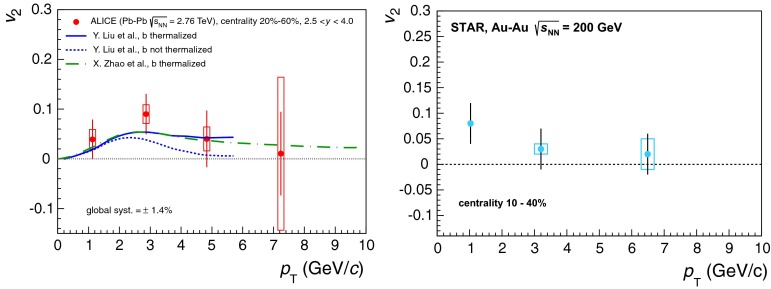
*Left* ALICE inclusive $\mathrm {J}/\psi$ measurement as a function of transverse momentum for semi-central Pb–Pb collisions [681], compared to TAMU [756] and THU [757] transport models calculations. *Right* STAR inclusive $\mathrm {J}/\psi$ measurement as a function of transverse momentum in different centrality bins [677]

In Fig. 89 (right) the prompt $$\mathrm {J}/\psi $$$$R_{\mathrm {AA}}$$ centrality dependence is compared with the predictions of the TAMU transport model. The observed suppression, increasing as a function of centrality, is due to the melting of primordial $$\mathrm {J}/\psi $$. The TAMU model provided a reasonable description of the ALICE low-$$p_{\mathrm {T}}$$$$\mathrm {J}/\psi $$$$R_{\mathrm {AA}}$$ (see Fig. 84), with a significant recombination contribution. On the contrary, no recombination component is needed to describe the high-$$p_{\mathrm {T}}$$$$\mathrm {J}/\psi $$ results.

In Fig. 90 (left), the prompt $$\mathrm {J}/\psi $$$$R_{\mathrm {AA}}$$ is compared to shadowing calculations. As already discussed for low-$$p_{\mathrm {T}}$$$$\mathrm {J}/\psi $$ results, shadowing, here considered as the only cold nuclear matter effect, cannot account for the observed suppression, clearly indicating that other cold or hot matter effects are needed to describe the experimental results.

In Fig. 90 (right), the centrality dependence of the prompt $$\mathrm {J}/\psi $$$$R_{\mathrm {AA}}$$ is compared to the collisional dissociation model, discussed in Sect. 5.1.6. The model describes the more peripheral events, but underestimates the suppression for the most central events. It also underestimate the $$p_{\mathrm {T}}$$ dependence of the $$\mathrm {J}/\psi $$$$R_{\mathrm {AA}}$$.

The CMS Collaboration also measured the $R_{\mathrm {AA}}$ of non-prompt $\mathrm {J}/\psi$, presented in Sect. 4.1.3.

#### $\mathrm J/\psi$ azimuthal anisotropy

Further information on the $\mathrm {J}/\psi$ production mechanism can be accessed by studying the azimuthal distribution of $\mathrm {J}/\psi$ with respect to the reaction plane. As discussed in Sect. 4, the positive $v_2$ measured for D mesons at LHC and heavy-flavour decay electrons at RHIC suggests that charm quarks participate in the collective expansion of the medium and do acquire some elliptic flow as a consequence of the multiple collisions with the medium constituents. $\mathrm {J}/\psi$ produced through a recombination mechanism, should inherit the elliptic flow of the charm quarks in the QGP and, as a consequence, $\mathrm {J}/\psi$ are expected to exhibit a large $v_{2}$. Hence this quantity is a further signature to identify the charmonium production mechanism.

ALICE measured the inclusive $$\mathrm {J}/\psi $$ elliptic flow in Pb–Pb collisions at forward rapidity [681], using the event-plane technique. For semi-central collisions there is an indication of a positive $$v_{2}$$, reaching $$v_{2} = 0.116\pm 0.046\,\text {(stat.)}\pm 0.029\,\text {(syst.)}$$ in the transverse momentum range $$2<p_{\mathrm {T}} <4~\text {GeV}/c $$, for events in the 20–40 % centrality class. In Fig. 91 (left), the $$\mathrm {J}/\psi $$$$v_{2}$$ in the 20–60 % centrality class is compared with the TAMU and THU transport model calculations, which also provide a fair description of the $$R_{\mathrm {AA}}$$ results, discussed in Sect. 5.2.2. Both models, which reasonably describe the data, include a fraction ($$\approx $$30 % in the centrality range 20–60 %) of $$\mathrm {J}/\psi $$ produced through (re)generation mechanisms, under the hypothesis of thermalisation or non-thermalisation of the *b*-quarks. More in detail, charm quarks, in the hot medium created in Pb–Pb collisions at the LHC, should transfer a significant elliptic flow to regenerated $$\mathrm {J}/\psi $$. Furthermore, primordial $$\mathrm {J}/\psi $$ might acquire a $$v_{2}$$ induced by a path-length dependent suppression due to the fact that $$\mathrm {J}/\psi $$ emitted out-of-plane traverse a longer path through the medium than those emitted in-plane. Thus, out-of-plane emitted $$\mathrm {J}/\psi $$ will spend a longer time in the medium and have a higher chance to melt. The predicted maximum $$v_{2}$$ at $$p_{\mathrm {T}}$$ = 2.5$$~\text {GeV}/c$$ is, therefore, the result of an interplay between the regeneration component, dominant at low $$p_{\mathrm {T}}$$ and the primordial $$\mathrm {J}/\psi $$ component which takes over at high $$p_{\mathrm {T}}$$ (see Fig. 82). The $$v_{2}$$ measurement complements the $$R_{\mathrm {AA}}$$ results, favouring a scenario with a significant fraction of $$\mathrm {J}/\psi $$ produced by (re)combination in the ALICE kinematical range.

At RHIC, measurements by the STAR Collaboration [677] of the $$\mathrm {J}/\psi $$$$v_{2}$$ in Au–Au collisions at $$\sqrt{s_{\mathrm{NN}}}$$ = 200$$~\text {GeV}$$ are consistent with zero for $$p_{\mathrm {T}} >2~\text {GeV}/c $$ albeit with large uncertainties, as shown in Fig. 91 (right), while a hint for a positive $$v_{2}$$ might be visible in the lowest $$p_{\mathrm {T}}$$ bin ($$0<p_{\mathrm {T}} <2~\text {GeV}/c $$). Results do not show a dependence on centrality. The measurement seems to disfavour the $$\mathrm {J}/\psi $$ formation through recombination mechanisms at RHIC energies, contrarily to what happens in Pb–Pb collisions at the LHC.

**Fig. 92:**
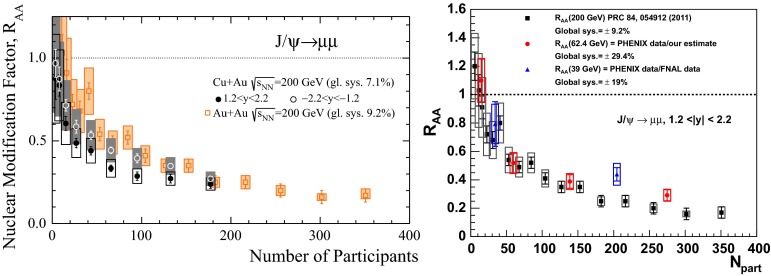
*Left* PHENIX $\mathrm {J}/\psi$ $R_{\mathrm {AA}}$ measured in Cu–Au  [672] and Au–Au collisions, shown as a function of the collision centrality. *Right* PHENIX $\mathrm {J}/\psi$ $R_{\mathrm {AA}}$ at various collision energies ($\sqrt{s_{\mathrm{NN}}}$ = 39, 62.4 and 200 $~\text {GeV}$) [674]

CMS has investigated the prompt $$\mathrm {J}/\psi $$$$v_{2}$$ as a function of the centrality of the collisions and as a function of transverse momentum [685]. Preliminary results indicate a positive $$v_{2}$$. The observed anisotropy shows no strong centrality dependence when integrated over rapidity and $$p_{\mathrm {T}}$$. The $$v_{2}$$ of prompt $$\mathrm {J}/\psi $$, measured in the 10–60 % centrality class, has no significant $$p_{\mathrm {T}}$$ dependence either, whether it is measured at low $$p_{\mathrm {T}}$$, $$3<p_{\mathrm {T}} <6.5~\text {GeV}/c $$, in the forward-rapidity interval $$1.6<|y|<2.4$$, or at high $$p_{\mathrm {T}}$$, $$6.5<p_{\mathrm {T}} <30~\text {GeV}/c $$, in the rapidity interval $$|y|<2.4$$. The preliminary CMS result supports the presence of a small anisotropy over the whole $$p_{\mathrm {T}}$$ range, but the present level of precision does not allow for a definitive answer on whether this anisotropy is constant or not. In the rapidity interval $$|y|<2.4$$, for $$p_{\mathrm {T}} >8~\text {GeV}/c $$, the anisotropy is similar to that observed for charged hadrons, the latter being attributed to the path-length dependence of the partonic energy loss [758].

#### $$\mathrm J/\psi $$$$R_{\mathrm {AA}}$$ results for various colliding systems and beam energies at RHIC

A unique feature of RHIC is the possibility of accelerating various symmetric or asymmetric ion species, allowing for the study of charmonium suppression as a function of the system size. Furthermore, since at RHIC it is possible to collect data at various $\sqrt{s_{\mathrm{NN}}}$, the charmonium production beam-energy dependence was also investigated from the top energy $\sqrt{s_{\mathrm{NN}}}$ = 200$~\text {GeV}$ down to $\sqrt{s_{\mathrm{NN}}}$ = 39$~\text {GeV}$.

The PHENIX Collaboration measured $\mathrm {J}/\psi$ production from asymmetric Cu–Au heavy-ion collisions at $\sqrt{s_{\mathrm{NN}}}$ = 200 $~\text {GeV}$ at both forward (Cu-going direction) and backward (Au-going direction) rapidities [672]. The nuclear modification of $\mathrm {J}/\psi$ yields in Cu–Au collisions in the Au-going direction is found to be comparable to that in Au–Au collisions when plotted as a function of the number of participating nucleons, as shown in Fig. 92 (left). In the Cu-going direction, $\mathrm {J}/\psi$ production shows a stronger suppression. This difference is comparable to expectation from nPDF effects due to stronger low-*x* gluon suppression in the larger Au nucleus.

Moreover, the PHENIX Collaboration measured nuclear modification factors also by varying the collision energies, studying Au–Au data at $\sqrt{s_{\mathrm{NN}}}$ = 39 and 62.4$~\text {GeV}$  [674]. The observed suppression patterns follow a trend very similar to those previously measured at $\sqrt{s_{\mathrm{NN}}}$ = 200$~\text {GeV}$, as shown in Fig. 92 (right). Similar conclusions can be drawn also from preliminary STAR results [678].

In spite of the large uncertainties associated to these results, up to now, this similarity presents a challenge to theoretical models that contain competing hot and cold matter effects with possibly different energy dependencies. For example, in the TAMU transport model [720], the larger $\mathrm {J}/\psi$ suppression towards higher collision energies due to higher energy-densities is counter-balanced by a larger contribution from (re)combination due to the increase of the total charm cross section, leading to an overall $\mathrm {J}/\psi$ suppression that is nearly independent of the collision energy in the range probed by the SPS and RHIC.

#### Excited charmonium states

The measurement of excited charmonium states in heavy-ion collisions is experimentally challenging. The $\psi \text {(2S)}$, observed via its $\mu ^{+}\mu ^{-}$ decay, is expected to yield 50 times less events than the corresponding $\mathrm {J}/\psi$ decay, while being subject to similar background rates. The P-wave states decay radiatively into $\mathrm {J}/\psi$ and a low energy photon that is difficult to find in the background of thousands of photons resulting from neutral pion decays. So far, only the $\psi \text {(2S)}$ was measured in heavy-ion collisions, by NA50 at the SPS [664] and by CMS at the LHC [686] (preliminary measurements also exist from the ALICE Collaboration [682]). NA50 found a suppression of $\psi \text {(2S)}$ relative to $\mathrm {J}/\psi$ that increases with centrality, an observation that is consistent with a sequential dissociation of charmonia. At the same time the $\psi \text {(2S)}$ to $\mathrm {J}/\psi$ ratio reached in central Pb–Pb collisions is also consistent with the prediction of the statistical hadronisation model, leaving open the question whether all charmonia melt at the SPS.

**Fig. 93:**
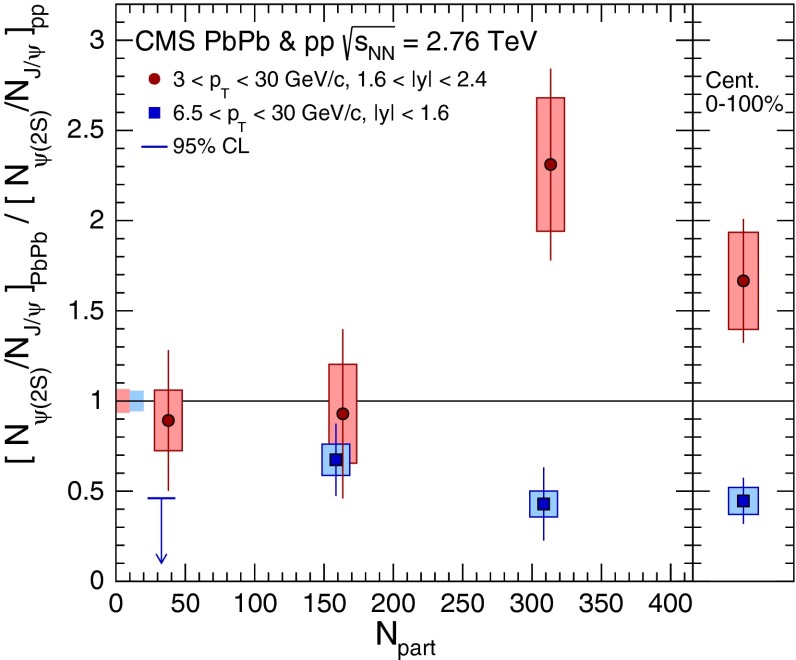
Mid-rapidity (*blue squares*) and forward-rapidity (*red circles*, slightly shifted) double ratio of measured yields, ($N_{\psi (2S)}/N_{J/\psi })$ Pb–Pb/($N_{\psi (2S)}/N_{J/\psi })_{pp}$, as a function of centrality [686]. The centrality-integrated results are displayed in the *right panel*. Statistical (systematic) uncertainties are shown as *bars* (*boxes*). The *boxes* at unity indicate the (global) $\mathrm pp$ uncertainties

At the LHC, CMS measured the yields of prompt $\mathrm {J}/\psi$ and $\psi \text {(2S)}$ in Pb–Pb and $\mathrm pp$ collisions at $\sqrt{s_{\mathrm{NN}}}$ = 2.76 $\text {TeV}$. The result is presented in Fig. 93 as a double ratio, $\left. (N_{\psi \text {(2S)}}/N_{\mathrm {J}/\psi })_{\mathrm {Pb}{-}\mathrm {Pb}}/(N_{\psi \text {(2S)}}/N_{\mathrm {J}/\psi })_{\mathrm {pp}}\right.$, as a function of event centrality for two kinematic regions: at mid-rapidity, $|y|<1.6$, $\psi \text {(2S)}$ are measured with $6.5<p_{\mathrm {T}} <30~\text {GeV}/c$, while at forward rapidity, $1.6<|y|<2.4$, the acceptance extends to $3<p_{\mathrm {T}} <30~\text {GeV}/c$.

**Fig. 94:**
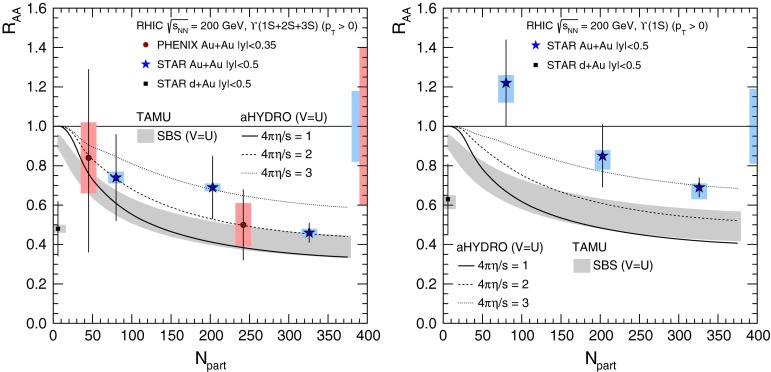
*Left* $R_{\mathrm {AA}}$ of $\Upsilon \text {(1S+2S+3S)}$ as a function of centrality measured by PHENIX in $|y|<0.35$ [321, 675] and STAR in $|y|<0.5$ [323] compared to TAMU (*grey band*) and aHYDRO (*lines*). *Right* STAR measurement of the $R_{\mathrm {AA}}$ of $\Upsilon \text {(1S)}$ with $|y|<0.5$ as a function of centrality compared to the same models

A clear difference between the centrality integrated double ratios in the two kinematic regions is found. At forward rapidity and low $p_{\mathrm {T}}$, the double ratio is larger than unity, i.e. the $\psi \text {(2S)}$ to $\mathrm {J}/\psi$ ratio is larger in Pb–Pb than in $\mathrm pp$. In contrast, the $\psi \text {(2S)}$ to $\mathrm {J}/\psi$ ratio is reduced in Pb–Pb compared to the ratio found in $\mathrm pp$ at mid-rapidity and high $p_{\mathrm {T}}$. Peripheral and semi-central collisions show a double ratio consistent with unity at forward rapidity, whereas the most central bin shows an increase of the double ratio. In contrast, the suppression of the double ratio at mid-rapidity appears to be independent of centrality. The difference between the two kinematic domains is highly unexpected. While the mid-rapidity and high-$p_{\mathrm {T}}$ result is in line with the expectation of sequential melting, the opposite behaviour is observed at forward rapidity and low $p_{\mathrm {T}}$. While regeneration is not expected to contribute in the investigated $p_{\mathrm {T}}$ ranges, it is worth to note that also the statistical hadronisation model predicts a $p_{\mathrm {T}}$-integrated double-ratio of $\approx$0.2. It remains to be seen which effects can explain these results, e.g. if regeneration of $\psi \text {(2S)}$ can be enhanced relative to $\mathrm {J}/\psi$ due to the larger binding radius. First attempts have been made to explain this observation, arguing that $\psi \text {(2S)}$ are regenerated at later stages than $\mathrm {J}/\psi$, i.e. when a stronger radial flow is present [759]. On the experimental side, it will be important to isolate whether the difference is due to the change in rapidity or $p_{\mathrm {T}}$, and what happens at $p_{\mathrm {T}} =0$. Preliminary results from the ALICE Collaboration at forward rapidity ($2.5<y<4$) and $p_{\mathrm {T}} >0$ are not precise enough to draw a conclusion [682].

#### Bottomonium $R_{\mathrm {AA}}$ results

With the advent of the LHC, bottomonia have become a new probe of the QGP. While their production rate is 200 times smaller than the one of $\mathrm {J}/\psi$, they offer several advantages. The three S-wave states $\Upsilon \text {(1S)}$, $\Upsilon \text {(2S)}$, and $\Upsilon \text {(3S)}$ have very different binding energies and appear at very similar rates in the $\mu ^{+}\mu ^{-}$ decay channel. Their relative abundances are 7:2:1, while the $\mathrm {J}/\psi$ to $\psi \text {(2S)}$ ratio is 50:1. Hence these three states, which include with the $\Upsilon \text {(1S)}$ the strongest bound state of all quarkonia, allow one to probe a much wider temperature range than previously accessible with charmonia. A further advantage is the absence of feed down from heavier-flavour decays, that are a background for high $p_{\mathrm {T}}$ charmonium studies. The higher masses also ease theoretical calculations. In the context of sequential dissociation, bottomonia may provide another advantage: the approximately 20 times smaller beauty production cross section will lead to a smaller contribution from regeneration that complicates the picture for charmonia. However, the closed-to-open heavy flavour production ratio for beauty is roughly ten times smaller than for charm, which increases the relative contribution of recombination to bottomonia and complicates the situation.

Unfortunately, feed-down contributions to the $\Upsilon \text {(1S)}$ from excited state decays that are crucial for a quantitative understanding of a sequential dissociation are not very well understood at low $p_{\mathrm {T}}$. Measurements of feed-down fractions exist only for $p_{\mathrm {T}} >6~\text {GeV}/c$, where about 30 % of $\Upsilon \text {(1S)}$ result from decays of $\chi _b(nP)$ and $\Upsilon \text {(2S+3S)}$ decays, reaching $\approx$50 % at higher $p_{\mathrm {T}}$  [200, 203, 207, 209].

At RHIC, where the $\Upsilon$ production cross sections are low, a measurement of the $\Upsilon$ suppression in d–Au and Au–Au collisions was performed by the PHENIX and STAR experiments [321, 323, 675]. Integrating the yield of the three $\Upsilon$ states, they observe a reduction of the yield in central Au–Au collisions, compared to the binary-scaled $\mathrm pp$ reference as shown in the left panel of Fig. 94. Because of the large statistical uncertainties, the experiments cannot yet assess a possible centrality dependence in Au–Au. STAR finds in the 10 % most central collisions a nuclear modification factor of $R_{\mathrm {AA}} = 0.49 \pm 0.13\,\text {({Au--Au} stat.)} \pm 0.07\,{(\mathrm pp \text {stat.})} \pm 0.02\,\text {({Au--Au} syst.)} \pm 0.06\,\text {(pp syst.)}$. Constraining the measurement to the $\Upsilon \text {(1S)}$ alone, as shown in the right panel of Fig. 94, only the $R_{\mathrm {AA}}$ for the most central Au–Au collisions exhibits a significant suppression. Assuming a feed-down contribution of $\approx$50 % this could signal the onset of a suppression of excited states in central Au–Au collisions. However, the $R_{\mathrm {AA}}$ in most central Au–Au collisions is also comparable to the $R_{\mathrm {dAu}}$, so more precise measurements are necessary before drawing such a conclusion.

A comparison of TAMU and aHYDRO calculations with the measured $\Upsilon \text {(1S+2S+3S)}$ nuclear modification factors shows good agreement within the experimental uncertainties. Experimental data cannot yet constrain the $\eta /s$ free parameter of aHYDRO. The band of the TAMU curve represents the uncertainty on cold nuclear matter effects. These are included by employing nuclear absorption cross sections of 1.0 and 3.1 mb, but the data cannot yet constrain their size. The $\Upsilon \text {(1S)}$ suppression, however, seems to be slightly over-predicted by both models, though not beyond the experimental uncertainties, with the data preferring small values of $\eta /s$.

**Fig. 95:**
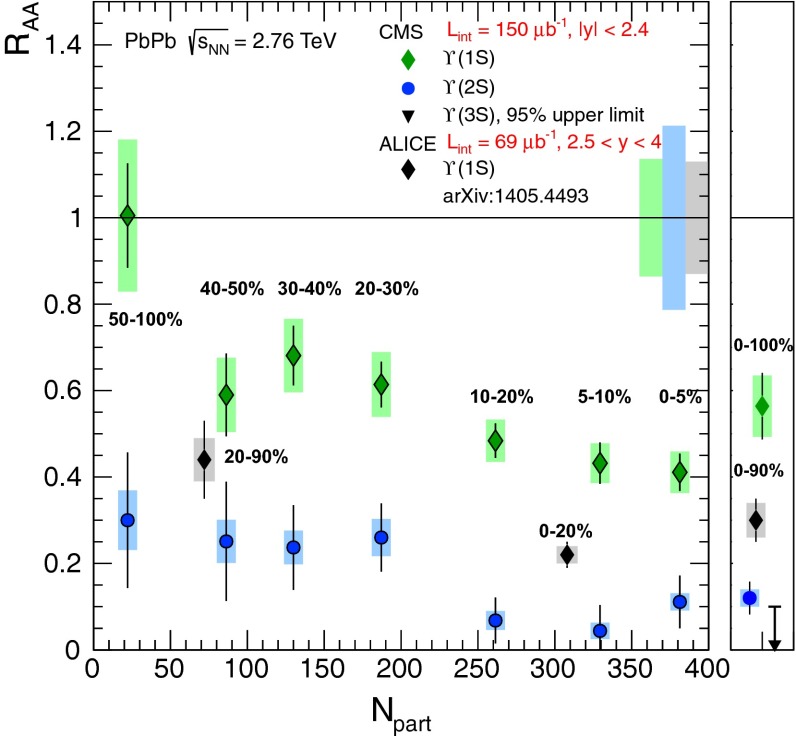
$\Upsilon \text {(nS)}$ $R_{\mathrm {AA}}$ as a function of centrality. $\Upsilon \text {(1S)}$ $R_{\mathrm {AA}}$ is measured in $2.5<|y|<4$ by ALICE [683]. CMS measured the centrality dependence of the $\Upsilon \text {(1S)}$ and $\Upsilon \text {(2S)}$ $R_{\mathrm {AA}}$ at $|y|<2.4$ [687, 688]. Centrality integrated values are shown in the *right panel*, including an upper limit at 95 % confidence level of the $\Upsilon \text {(3S)}$ $R_{\mathrm {AA}}$ by CMS in $|y|<2.4$

**Fig. 96:**
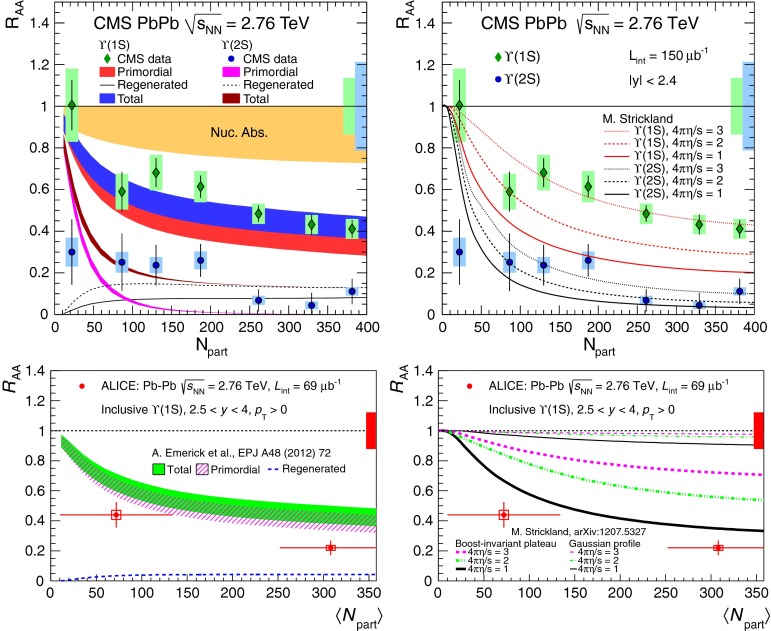
$\Upsilon \text {(1S)}$ $R_{\mathrm {AA}}$ versus centrality at $|y|<2.4$ [687, 688] (*top*) and $2.5<y<4$ [683] (*bottom*), compared to TAMU (*left*) and aHYDRO (*right*) model calculations discussed in Sects. 5.1.4 and 5.1.5

**Fig. 97:**
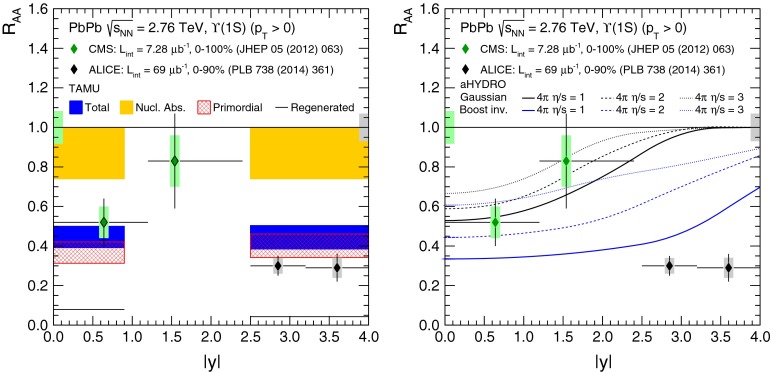
$\Upsilon \text {(1S)}$ $R_{\mathrm {AA}}$ versus rapidity from ALICE [683] and CMS [687, 688]. In the *bottom row*, the $R_{\mathrm {AA}}$ is to TAMU (*left*) and aHYDRO (*right*) model calculations discussed in Sects. 5.1.4 and 5.1.5

CMS measured the suppression of the first three S states integrated over all $$p_{\mathrm {T}}$$ and the rapidity range $$|y|<2.4$$ in Pb–Pb collisions at $$\sqrt{s_{\mathrm{NN}}}$$ = 2.76 $$\text {TeV}$$  [687, 688]. Following a first tantalising indication in 2011 that the excited states are suppressed relative to the $$\Upsilon \text {(1S)}$$, this was confirmed a year later. The centrality integrated $$R_{\mathrm {AA}}$$ was measured for all three states, exhibiting a clear ordering with binding energy: $$R_{\mathrm {AA}} (\Upsilon \text {(1S)}) = 0.56 \pm 0.08\text {(stat.)} \pm 0.07\text {(syst.)}$$, $$ R_{\mathrm {AA}} (\Upsilon \text {(2S)}) = 0.12 \pm 0.04\text {(stat.)} \pm 0.02\text {(syst.)}$$, and the $$\Upsilon \text {(3S)}$$ being so strongly suppressed that only an upper limit of $$R_{\mathrm {AA}} (\Upsilon \text {(3S)})<0.10$$ at 95 % CL could be quoted. The centrality dependence of the $$\Upsilon \text {(1S)}$$ and $$\Upsilon \text {(2S)}$$$$R_{\mathrm {AA}}$$ are shown in Fig. 95. With the $$\Upsilon \text {(2S)}$$ and $$\Upsilon \text {(3S)}$$ essentially completely suppressed in central Pb–Pb collisions, a more precise understanding of the feed-down contributions to the $$\Upsilon \text {(1S)}$$ is required to assess whether any directly produced $$\Upsilon \text {(1S)}$$ are suppressed in such collisions. Furthermore, the role of the $$\chi _b \text {(nP)}$$ states in Pb–Pb are (and may remain) completely unknown so far.

In the top row of Fig. 96, the centrality dependence of the CMS $\Upsilon \text {(1S)}$ and $\Upsilon \text {(2S)}$ results are compared to TAMU (left) and aHYDRO (right) calculations, described in Sects. 5.1.4 and 5.1.5, respectively. Both models reproduce the data reasonably well, simultaneously describing the $\Upsilon \text {(1S)}$ and $\Upsilon \text {(2S)}$ suppression over the full centrality range. The aHYDRO approach has maybe some slight tension describing both states with the same choice for $\eta /s$, though the experimental uncertainties are large enough to account for the differences. Regarding the TAMU model, it is worth to highlight that it includes a non-negligible regeneration contribution. In fact, it is the sole source of $\Upsilon \text {(2S)}$ in central Pb–Pb collisions. It is also interesting to point out that regeneration favours the production of $\Upsilon \text {(2S)}$ over $\Upsilon \text {(1S)}$, which is opposite to the predictions for charmonia. This difference is the result of temperature dependent dissociation rates and equilibrium numbers that enter the rate equation (Eq. (57)). In contrast to the other states, which all have dissociation temperatures in the vicinity of $T_c$, the strong-binding energy will stop the dissociation of $\Upsilon \text {(1S)}$ much earlier, when the equilibrium number is still small [729]. Significantly less regeneration of $\Upsilon \text {(1S)}$ is necessary to reach this equilibrium number.

The production of excited $\Upsilon$ states in Pb–Pb collisions has also been reported by CMS as fully corrected cross section ratio relative to the $\Upsilon \text {(1S)}$: $\sigma (\Upsilon \text {(2S)})/\sigma (\Upsilon \text {(1S)}) = 0.09 \pm 0.02\,(\text {stat.}) \pm 0.02\,(\text {syst.}) \pm 0.01\,(\text {glob.})$ integrated over centrality, $p_{\mathrm {T}}$, and $|y|<2.4$ [268]. For the ratio $\sigma (\Upsilon \text {(3S)})/\sigma (\Upsilon \text {(1S)})$ an upper limit of 0.04 at 95 % CL has been set. These values can be directly compared to theoretical expectations, e.g. the statistical hadronisation model, which predicts $\sigma (\Upsilon \text {(2S)})/\sigma (\Upsilon \text {(1S)}) \approx 0.032$ [760]. This value is consistent with the measured cross section ratio, though quite a bit lower than the central value of the measurement.

A comparison of the CMS measurement at mid-rapidity to $R_{\mathrm {AA}} (\Upsilon \text {(1S)}) = 0.30 \pm 0.05\text {(stat.)} \pm 0.04\text {(syst.)}$ measured by ALICE at forward rapidity ($2.5<y<4$) [683], integrated over $p_{\mathrm {T}}$ and centrality, as well as the centrality dependence overlaid in Fig. 95, reveal a surprising similarity to the $\mathrm {J}/\psi$ suppression observed at RHIC: $\Upsilon \text {(1S)}$ are more suppressed at forward rapidity than at mid-rapidity. At RHIC such rapidity dependence was explained with a larger contribution of regeneration at mid-rapidity and/or stronger shadowing effects at forward rapidity. This similarity is also reflected in the centrality integrated rapidity dependence of $R_{\mathrm {AA}} (\Upsilon \text {(1S)})$ shown in Fig. 97. However, the large statistical uncertainties on the CMS measurement [482] that is still based on the first Pb–Pb and $\mathrm pp$ runs at $\sqrt{s_{\mathrm{NN}}}$ = 2.76 $\text {TeV}$ prevents conclusions on the $R_{\mathrm {AA}}$ in the intermediate rapidity range.

The simultaneous description of ALICE and CMS data provides a real challenge for the models so successful in reproducing the mid-rapidity data. As shown in Figs. 96 and 97, they completely fail to predict the rapidity dependence of $R_{\mathrm {AA}} (\Upsilon \text {(1S)})$. The aHYDRO model, curves taken from Ref. [761], predicts a disappearance of the suppression at forward rapidity and does not get even close to the ALICE data. The TAMU transport model approach, including a regeneration component, predicts a rather constant rapidity dependence of the suppression though still overshoots the forward rapidity data slightly. In both models the $\Upsilon \text {(1S)}$ suppression is dominated by the in-medium dissociation of the higher-mass bottomonium states. Therefore, a precise measurement of $\Upsilon \text {(1S)}$ feed-down contributions, as well as an accurate estimate of CNM effects in the kinematic ranges probed by ALICE and CMS is required in order to make a more stringent comparison with data.

It is interesting to compare the $$R_{\mathrm {AA}}$$ of the three bottomonium states to the $$R_{\mathrm {AA}}$$ of the $$\mathrm {J}/\psi $$ and $$\psi \text {(2S)}$$ at high $$p_{\mathrm {T}}$$. The charmonium states follow nicely the established pattern of the $$\Upsilon $$ states of a reduced suppression with increasing binding energy as predicted by the sequential dissociation picture. If one, however, uses the $$p_{\mathrm {T}}$$ integrated $$\mathrm {J}/\psi $$$$R_{\mathrm {AA}}$$, one observes a deviation from this pattern that can be explained with a (re)generation contribution. It will be interesting to see how low $$p_{\mathrm {T}}$$$$\psi \text {(2S)}$$ will fit in.

The picture may be complicated further by the observed multiplicity dependence of the $\Upsilon \text {(2S)}/\Upsilon \text {(1S)}$ and $\Upsilon \text {(3S)}/\Upsilon \text {(1S)}$ ratios in $\mathrm pp$ and p–Pb collisions [268] that is discussed in Sects. 2.4.1 and 3.3.3. It is unclear whether the dependence is caused by a suppression of the excited states by surrounding particles or by the multiplicity being biased by the presence of the $\Upsilon$ states.

### Alternative references for quarkonium production in nucleus–nucleus collisions

#### Proton–nucleus collisions

As discussed in Sect. 3, proton–nucleus data can provide information on CNM effects on quarkonium production. Since these mechanisms are present also in AA collisions, their precise evaluation is mandatory to correctly quantify the hot matter effects. However, the extrapolation of CNM effects evaluated in p–A to AA collisions is model dependent and it has to rely on assumptions as those discussed in detail in Sect. 3.4.

The ALICE Collaboration investigated the role of CNM effects on the $$\mathrm {J}/\psi $$$$R_{\mathrm {AA}}$$ in Pb–Pb collisions, extrapolating the $$R_{\mathrm {pA}}$$ results obtained in p–Pb collisions at $$\sqrt{s_{\mathrm{NN}}}$$ = 5.02 $$\text {TeV}$$  [325]. Although the forward-rapidity ALICE p–A data were collected at a higher $$\sqrt{s_{\mathrm{NN}}}$$ energy and cover a slightly different centre of mass rapidity range with respect to Pb–Pb collisions ($$2.03<y<3.53$$ and $$-4.46<y<-2.96$$ in p–Pb and $$2.5<y<4$$ in Pb–Pb), the Bjorken-*x* regions probed by the $$\mathrm {J}/\psi $$ production process in the colliding nuclei are rather similar, differing by less than $$\approx $$10 %. The *x* values covered in Pb–Pb collisions are $$2\times 10^{-5}<x<9\times 10^{-5}$$ and $$1\times 10^{-2}<x<6\times 10^{-2}$$, for Pb nuclei moving away from or towards the ALICE muon spectrometer, in which the $$\mathrm {J}/\psi $$ at forward rapidity are detected. In p–Pb collisions the corresponding figures are $$2\times 10^{-5}<x<8\times 10^{-5}$$ and $$1\times 10^{-2}<x<5\times 10^{-2}$$ for the Pb nucleus going away or towards the ALICE muon spectrometer. Under the assumptions that shadowing is the main nuclear mechanism and that its influence on the two nuclei in Pb–Pb collisions can be factorised, cold nuclear matter effects are then evaluated as the product of the $$\mathrm {J}/\psi $$$$R_{\mathrm {pA}}$$ computed at forward and backward rapidities, i.e. $$R_{\mathrm {pA}} (y) \times R_{\mathrm {pA}} (-y)$$. The $$R_{\mathrm {pA}}$$ product, in the ALICE forward-rapidity region, is 0.75 $$\pm $$ 0.10 (stat) $$\pm $$ 0.12 (syst) when integrated over $$p_{\mathrm {T}}$$. With $$R_{\mathrm {AA}}$$ = 0.57 $$\pm $$ 0.01 (stat) $$\pm $$ 0.09 (syst), this is a hint that the observed $$\mathrm {J}/\psi $$ suppression in Pb–Pb cannot be ascribed to shadowing effects alone. Similar conclusions, even if with larger uncertainties, can be obtained from ALICE results at mid-rapidity. This observation can be strengthened by comparing the $$p_{\mathrm {T}}$$ dependence of the $$\mathrm {J}/\psi $$$$R_{\mathrm {AA}}$$ to the one of the CNM effects evaluated as $$R_{\mathrm {pA}} (y) \times R_{\mathrm {pA}} (-y)$$ [327]. In this case, an opposite transverse momentum dependence is observed for the extrapolated shadowing, increasing from low to high $$p_{\mathrm {T}}$$, and the $$\mathrm {J}/\psi $$$$R_{\mathrm {AA}}$$ pattern, showing a decrease towards high $$p_{\mathrm {T}}$$, with a hint of an enhancement at low $$p_{\mathrm {T}}$$. In particular, at high $$p_{\mathrm {T}}$$, the observed $$R_{\mathrm {AA}}$$ suppression is much larger than the shadowing extrapolation. Moreover, coherent energy-loss effects are also expected to weaken at large $$p_{\mathrm {T}}$$  [396], unlike the trend of the data. This clearly points to the existence of strong hot matter effects [327].

**Fig. 98:**
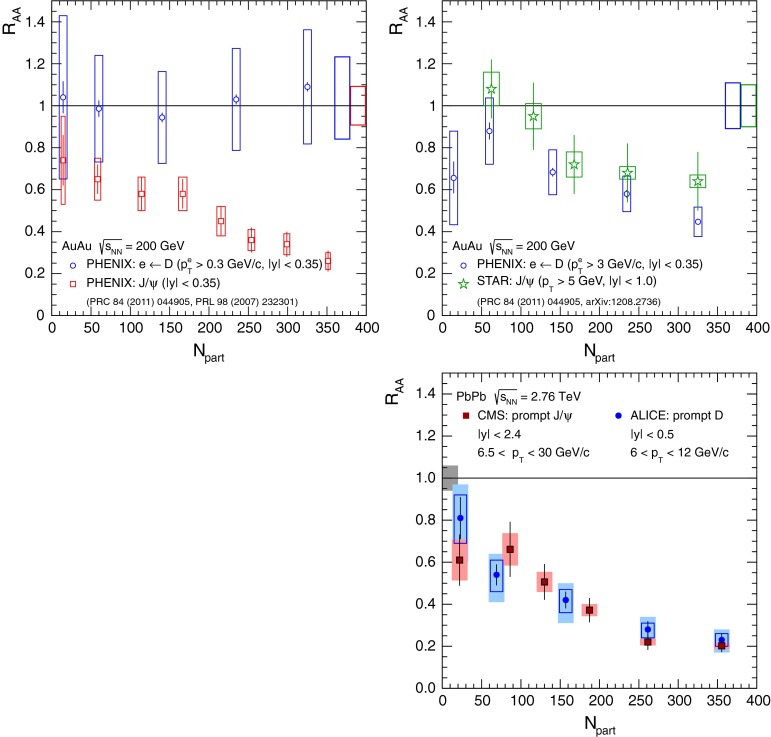
Comparison of nuclear modification factors for open and closed charm at RHIC (*top*) and the LHC (*bottom*) [239, 469, 477, 482, 667]. Transverse momentum integrated results are shown on the *left*, while high $p_{\mathrm {T}}$ $R_{\mathrm {AA}}$ are compared on the *right*. Due to the lack of low $p_{\mathrm {T}}$ open charm data at the LHC, the *bottom left panel* is missing

#### Open heavy flavour

To study the effect of a deconfined medium on quarkonium production, we first recall the underlying dynamics, using the $\mathrm {J}/\psi$ for illustration. The production process in elementary hadronic collisions begins with the formation of a ${c\overline{c}}$ pair; this pair can then either lead to open charm production (about 90 %) or subsequently bind to form a charmonium state (about 10 % for all charmonia). Since quarkonium production is to be used as a tool to study the medium produced in nuclear collisions, the primary concern is not if such collisions produce more or fewer ${c\overline{c}}$ pairs than proton–proton collisions, but rather if the presence of the medium modifies the fraction of produced ${c\overline{c}}$ pairs going into charmonium formation. In other words, the crucial quantity is the amount of charmonium production relative to that of open charm. Hence the relevant observable is the fraction of charmonia to open charm, or more generally, that of quarkonia to the relevant open heavy flavour production [762, 763]. In this quantity, if measured over the entire phase space, down to $p_{\mathrm {T}} =0$, the effects of possible initial-state nuclear modifications cancel out, so that whatever changes it shows relative to the $\mathrm pp$ pattern is due to final-state effects. Here it should be noted that, since the distribution of the different open charm channels is in good approximation energy-independent, the measurement of a single such channel is sufficient – it gives, up to a constant, the total open charm cross section [763].

A direct comparison of measured open and closed heavy-flavour cross sections has not been performed yet at RHIC or the LHC. However, one can compare the measured nuclear modification factors of D mesons (or heavy-flavour decay electrons as their proxy) and $$\mathrm {J}/\psi $$. At RHIC, the open charm cross section has been measured in $$\mathrm pp$$ and Au–Au via non-photonic single electrons from semi-leptonic charm decays [469] as well as with fully reconstructed D mesons via hadronic decays [475]. As shown in the top left of Fig. 98, the resulting $$R_{\mathrm {AA}}$$ shows no deviation from binary scaling, though the uncertainties are sizeable. Hence, one can conclude that the modification of the $$\mathrm {J}/\psi $$$$R_{\mathrm {AA}}$$ at RHIC [667] is a true final-state effect and not just a reduction of charm production by initial-state effects. As evident from the large uncertainties, the measurement of the total charm cross section is extremely challenging. At the LHC this has not been achieved yet, preventing such a comparison in the bottom left of Fig. 98. Instead one can try to make a comparison at high $$p_{\mathrm {T}}$$ where both, open and closed charm, have been measured [477, 482]. This then opens the question which $$p_{\mathrm {T}}$$ intervals are appropriate for such a comparison. A comparison of D and $$\mathrm {J}/\psi $$ in the same $$p_{\mathrm {T}}$$ range will not access the same charm quarks and/or gluons. This is an issue to be addressed on theoretical grounds. The comparison is anyway performed, as shown in the bottom right panel of Fig. 98. The $$R_{\mathrm {AA}}$$ of high-$$p_{\mathrm {T}}$$ D and $$\mathrm {J}/\psi $$ show a surprising similar centrality dependence. This, however, is nothing new at the LHC. Already at RHIC the same trend can be observed [239, 469], as shown in the top right of Fig. 98. The suppression of high-$$p_{\mathrm {T}}$$ D mesons has been linked to charm quark energy loss inside the QGP. While the $$\mathrm {J}/\psi $$ itself is a colourless object, its coloured precursor may be subject to similar energy loss though current models underestimate the $$\mathrm {J}/\psi $$ suppression at high $$p_{\mathrm {T}}$$ in Pb–Pb collisions at the LHC [83].

### Summary and outlook

With the LHC Run 1 a large wealth of quarkonium measurements has enriched and complemented the observations from SPS and RHIC experiments. The main results and their current interpretation are summarised in the following.

- In the charmonium sector, the $$\mathrm {J}/\psi $$$$R_{\mathrm {AA}}$$ at high transverse momentum shows a clear suppression. This suppression is stronger than the one observed at RHIC energies, as expected in a sequential melting scenario.
- An opposite behaviour was in the low-$$p_{\mathrm {T}}$$ region, where the $$\mathrm {J}/\psi $$$$R_{\mathrm {AA}}$$ measured at LHC is larger than the one obtained at lower energies. This observation can be interpreted as evidence for a new production mechanism setting in at high energies, based on the (re)combination of *c* and $$\bar{c}$$ quarks either during the collision history or at the hadronisation. The measurement of a positive $$v_{2}$$, for low-$$p_{\mathrm {T}}$$$$\mathrm {J}/\psi $$, is considered a further confirmation of the important role played by this additional contribution.
- Theoretical models assuming a fraction of $\mathrm {J}/\psi$ produced by (re)combination of the order of 50 % at low $p_{\mathrm {T}}$ and then vanishing at high $p_{\mathrm {T}}$, provide a fair description of the experimental data. On the contrary, calculations including only shadowing effects cannot account for the observed suppression. Note, however, that coherent energy-loss effects in cold nuclear matter are able to reproduce the $\mathrm {J}/\psi$ suppression, yet the agreement may be of accidental origin [404].
- For the first time, the $R_{\mathrm {AA}}$ for $\Upsilon \text {(1S)}$, $\Upsilon \text {(2S)}$, and $\Upsilon \text {(3S)}$ was measured. Results indicate a clear ordering with binding energy, as expected in the sequential melting scenario.

Even if a qualitative understanding of the quarkonium behaviour at LHC energies is nowadays rather well assessed, there are still several aspects which are required to be further studied:

- In order to quantify the hot matter effects on quarkonium production, a precise knowledge of the cold nuclear matter effects is required. Accurate quarkonium measurements in p–A collisions are, therefore, mandatory to refine the interpretation of the AA results.
- Theoretical calculations are, as of today, still affected by large uncertainties, mainly due to the uncertainties on the cold nuclear matter effects and, for models including a (re)generation component, also on the uncertainties on the $${c\overline{c}}$$ production cross section. The comparison of the measured $$\mathrm {J}/\psi $$$$R_{\mathrm {AA}}$$ and theory predictions will benefit from the measurement of the latter down to zero $$p_{\mathrm {T}}$$.
- Intriguing results have been obtained on the $\psi \text {(2S)}$ in the LHC Run 1. Given the observed dependence on rapidity and transverse momentum, the interpretation of the $\psi \text {(2S)}$ behaviour will clearly gain from a more differential study feasible with larger data sample.
- Similarly, also bottomonia will benefit from multi-differential studies to assess the kinematic dependence of all the $\Upsilon$ states.
- Finally, the availability of charmonium and bottomonium results spanning almost three orders of magnitude in $\sqrt{s_{\mathrm{NN}}}$ and covering very different kinematic regions represents clearly a challenge for all theoretical models, which should now move towards a consistent description of quarkonium data.

The incoming LHC Run 2 data are expected to shed more light, moving from a qualitative understanding of the quarkonium fate in a hot medium towards a more quantitative one.

## Quarkonium photoproduction in nucleus–nucleus collisions

In 2011, the LHC produced collisions of lead ions at a centre-of-mass energy per nucleon pair $\sqrt{s_\mathrm{NN}} = 2.76$ TeV. These collisions have been used to perform different measurements of charmonium photonuclear production.^28^ All but one of the studies described in this section have been carried out using ultra-peripheral collisions (UPC). These are interactions where the impact parameter exceeds the sum of the radii of the colliding nuclei. In such collisions, the cross section for hadronic processes is strongly suppressed, while the cross section for electromagnetic interactions remains large. The analysis not related to UPC has investigated the photoproduction of $\mathrm {J}/\psi$ overlapped with a standard hadronic Pb–Pb collision.

Two types of photonuclear production of charmonium have been studied: coherent and incoherent. In the first case, the incoming quasi-real photon interacts coherently with the whole nucleus to produce the charmonium. The coherence condition, both in the emission of the photon and in the interaction with the nuclear target, constrains the transverse momentum of the produced vector meson to be of the order of the inverse of the nucleus diameter, which translates into approximately 60 MeV/*c*. In the incoherent case, the quasi-real photon couples only to one nucleon, and thus the transverse momentum of the produced vector meson is constrained by the size of the nucleon, which translates into approximately 300 MeV/*c*. In a fraction of the coherent interactions and in all incoherent processes, one or a few neutrons are produced at the rapidity of the incoming beams. The experimental signature of these processes is therefore a vector meson with fairly small transverse momentum, possibly one or a few neutrons detected at zero degrees, and nothing else in the detector.

**Fig. 99:**
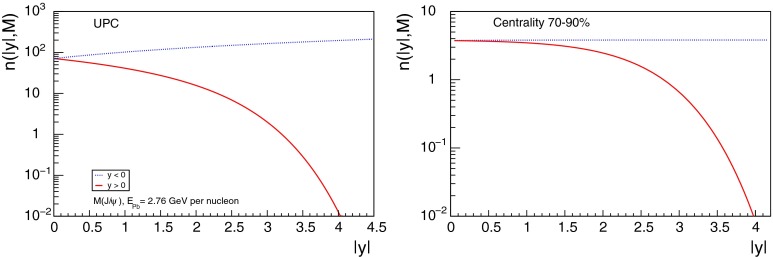
Flux of photons emitted by one nucleus for positive and negative values of the rapidity *y* for the case of $\mathrm {J}/\psi$ coherent photoproduction and an energy of the lead-ion beam of 2.76 GeV per nucleon. Positive rapidities correspond to the direction of the lead ion. The *left panel* shows the UPC case given by Eq. (64), while the *right panel* shows the integration of Eq. (63) for the impact parameters corresponding to the 70–90 % centrality class in hadronic Pb–Pb collisions

In this section, we review these measurements and discuss the models proposed to describe them. Previous reviews addressing these subjects can be found in [765–768]. This section is organised as follows. First, in Sect. 6.1 we discuss the origin and characteristics of the photon flux at the LHC. Section 6.2 describes previous results from RHIC and the existing measurements from LHC. Section 6.3 presents the current theoretical models and the main differences among them. Section 6.4 discusses how the models compare to the experimental results. We conclude in Sect. 6.5 with a brief summary of the lessons learnt and with an outlook of what could be possible with the data from the LHC Run 2.

### The flux of photons from lead ions at the LHC

The lead beams in the LHC are an intense source of photons, because the electromagnetic field of charged particles accelerated to ultra-relativistic velocities can be seen as a flux of quasi-real photons, according to a proposal made by Fermi [769, 770], and later refined by Weizsäcker [771] and Williams [772]. In this section we discuss the emission of photons from one nucleus. Next section discusses the cross section taking into account the contribution from both nuclei participating in the collision.

The photons are emitted by the nucleus coherently and thus their virtuality is restricted to be of the order of the inverse of the nucleus diameter, which for lead implies an upper limit for the virtuality around 30 MeV/*c*; i.e., the photons can be considered as quasi-real. The intensity of the flux depends on the square of the electric charge of the incoming particle, so it is large for the lead nuclei at the LHC. In the semi-classical description (see for example [766]) the photon flux per unit area is given by

63 $$\begin{aligned} \frac{\mathrm {d}^3n(k,\vec {b})}{\mathrm {d}k\mathrm {d}^2\vec {b}} = \frac{\alpha _\mathrm{em} Z^2}{\pi ^2 kb^2}x^2\left[ K^2_1(x)+\frac{1}{\gamma ^2}K^2_0(x)\right] , \end{aligned}$$

where $\alpha _\mathrm{em}$ is the fine structure constant, *k* is the photon energy in the frame where the photon emitter has a Lorentz factor gamma, *Z* is the electric charge of the lead nucleus, $K_{0}$ and $K_1$ are modified Bessel functions, $\vec {b}$ is the impact-parameter vector with *b* its magnitude and $x=kb/\gamma$.

The measurements of ultra-peripheral collisions described in the next sections were obtained requiring the absence of a hadronic collision between the incoming nuclei. This requirement is implemented into the computation of the photon flux in two different ways. The simpler option is to integrate Eq. (63) starting from a minimum impact parameter $b_\mathrm{min}$ given by the sum of the radii of the incoming nuclei. In this case, known as the hard-sphere approximation, the flux of quasi-real photons is given by

64 $$\begin{aligned} \frac{\mathrm {d}n(k)}{\mathrm {d}k} = \frac{2\alpha _\mathrm{em} Z^2}{\pi k}\left[ \xi K_0(\xi )K_1(\xi )-\frac{\xi ^2}{2}\left( K^2_1(\xi )-K^2_0(\xi )\right) \right] , \nonumber \\ \end{aligned}$$

with $$\xi =kb_\mathrm{min}/\gamma $$.

Another option is to convolute the flux per unit area with the probability of no hadronic interaction, which is obtained using the nuclear overlap function and the total nucleon–nucleon interaction cross section and averaging the flux over the target nucleus (for further details see [773]). The resulting integral can be calculated numerically to obtain the photon flux *n*(*k*).

All the measurements described below are elastic in the sense that the measurement of the charmonium fixes completely the kinematics of the process. In this case the energy of the photon can be expressed in terms of the mass *M* of the charmonium and its rapidity *y* as

65 $$\begin{aligned} k = \frac{M}{2}\exp {(y)}, \end{aligned}$$

and thus, the photon flux can be written as

66 $$\begin{aligned} n(y,M)\equiv k\frac{\mathrm {d}n(k)}{\mathrm {d}k}. \end{aligned}$$

**Fig. 100:**
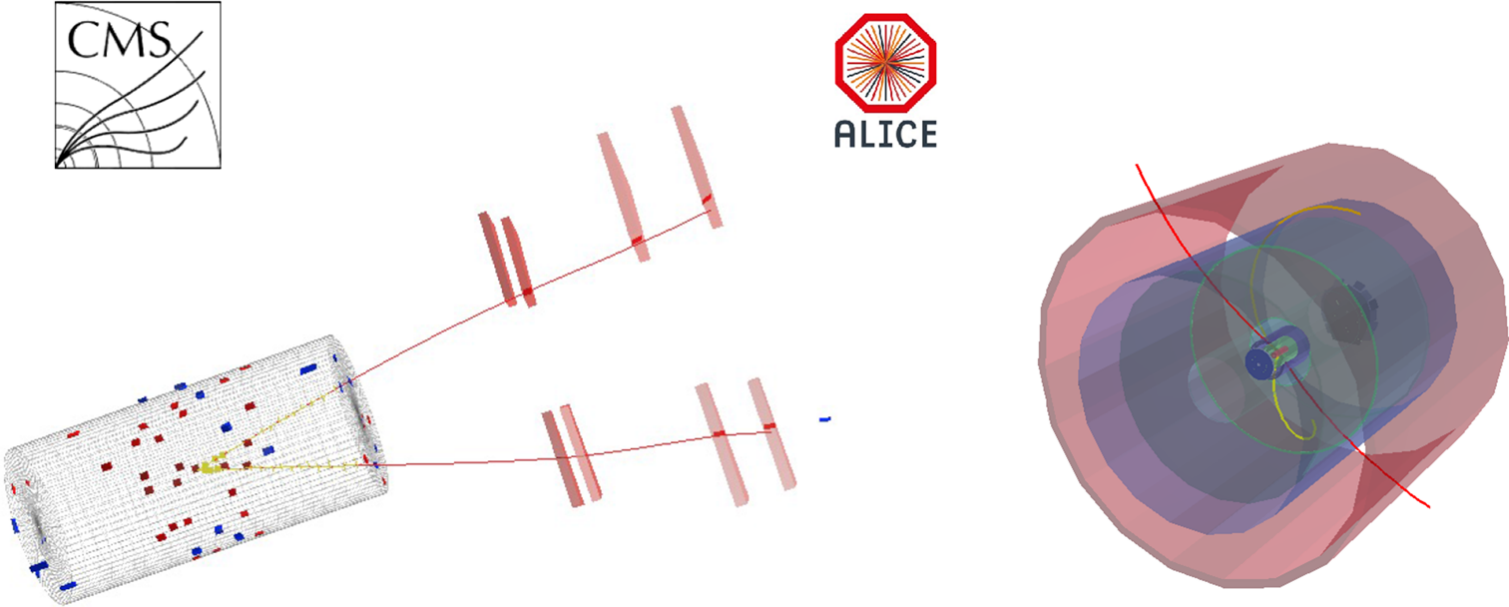
Event displays of coherent photonuclear production of $\mathrm {J}/\psi \rightarrow \mu ^{+}\mu ^{-}$ (*left*) and $\psi \text {(2S)} \rightarrow \mathrm {J}/\psi \pi ^{+}\pi ^{-} \rightarrow \mu ^{+}\mu ^{-}\pi ^{+}\pi ^{-}$ (*right*) in UPC as measured with the CMS and ALICE detectors, respectively

Figure 99 shows the flux of quasi-real photons with the energy required to produce a $\mathrm {J}/\psi$ at rapidity *y*. At large negative *y* the centre-of-mass energy of the photon-lead system is not large enough to produce the vector meson. For the energies of the lead beams during Run 1 and the case of a $\mathrm {J}/\psi$ this happens at $y\approx -6.8$; see e.g. Eq. (68). The left panel shows the UPC case, computed with Eq. (64): as the rapidity increases the flux decreases. At large rapidities the fast decrease of the flux is related to the behaviour of the Bessel functions. The right panel shows the integration of Eq. (63) for the impact parameters corresponding to the 70–90 % centrality class in hadronic Pb–Pb collisions according to [774]. This case is discussed in Sect. 6.2.5.

### Measurements of photonuclear production of charmonium during the Run 1 at the LHC

In both coherent and incoherent photonuclear production of charmonium the target is not broken by the interaction and in this sense the processes may be considered elastic. Therefore, the measurement of the produced charmonium completely fixes the kinematics.

As mentioned above, the experimental signature for these processes in UPC consists then in the decay products of a charmonium with fairly small transverse momentum and in some cases one or a few neutrons at zero degrees. No other event activity is measured in the detector. Figure 100 shows two event displays of the coherent photonuclear production of $\mathrm {J}/\psi$ and $\psi \text {(2S)}$ in UPC as measured with the CMS and ALICE detectors.

**Table 17:** Summary of published measurements of photonuclear production of charmonium in Pb–Pb UPC at $\sqrt{s_\mathrm{NN}} = 2.76$ $\text {TeV}$ at the LHC (the preliminary results on $\mathrm {J}/\psi$ from CMS [781] and $\psi \text {(2S)}$ from ALICE [786] are not included)

Experiment	Vector meson	d$\sigma /$d*y* (mb)	Rapidity range	References
ALICE	Coherent $\mathrm {J}/\psi$	1.00 $\pm$ 0.18(stat) $^{+0.24}_{-0.26}$ (syst)	$-3.6<y<-2.6$	[780]
ALICE	Coherent $\mathrm {J}/\psi$	2.38 $^{+0.34}_{-0.24}$ (stat $+$ syst)	$|y|<0.9$	[779]
ALICE	Incoherent $\mathrm {J}/\psi$	0.98 $^{+0.19}_{-0.17}$ (stat $+$ syst)	$|y|<0.9$	[779]

The cross section for these photonuclear processes in Pb–Pb collisions, with the charmonium measured at rapidity *y*, has two contributions:

67 $$\begin{aligned} \frac{\mathrm {d}\sigma _\mathrm{PbPb}(y)}{\mathrm {d}y}= n(y,M)\sigma _{\gamma \mathrm Pb}(y) + n(-y,M)\sigma _{\gamma \mathrm Pb}(-y), \nonumber \\ \end{aligned}$$

where the first term corresponds to one of the incoming lead nucleus acting as the source of the photon and the second term corresponds to the other incoming nucleus acting as the source of the photon. When the charmonium is measured at mid-rapidities, $y=0$, both terms are equal and can be summed. On the other hand, when the charmonium is measured at rapidities around 3, the flux at positive *y* is strongly suppressed and the term at negative *y* dominates. Note that for the case of photonuclear production overlapped with a hadronic collision, both fluxes contribute even at the forward rapidities measured at the LHC. This is illustrated in Fig. 99.

As mentioned before, the measurement of the charmonium fixes the kinematics. The centre of mass energy of the $\gamma$-Pb system is given by

68 $$\begin{aligned} W^2_{\gamma \mathrm {Pb}} = 2k\sqrt{s_\mathrm{NN}} = M\exp {(y)}\sqrt{s_\mathrm{NN}}, \end{aligned}$$

where in these expressions, both *k* and *y* are evaluated in the nucleus–nucleus centre-of-mass frame. In a leading-order pQCD approach $W_{\gamma \mathrm {Pb}}$ is related to *x*–Bjorken by

69 $$\begin{aligned} x = \frac{M^2}{W^2_{\gamma \mathrm {Pb}} }. \end{aligned}$$

According to this prescription, a measurement of charmonium photonuclear production at large rapidities in UPC samples mainly the low $W_{\gamma \mathrm {Pb}}$, alternatively large *x*, contribution to Eq. (67).

#### Photonuclear production of $\mathrm {J}/\psi$ at RHIC

The first measurement of photonuclear production of charmonium in UPC of relativistic heavy ions was performed by the PHENIX Collaboration using Au–Au collisions at $\sqrt{s_\mathrm{NN}} = 200$ GeV [775]. The events were triggered by tagging the production of neutrons at zero degrees and the $\mathrm {J}/\psi$ were measured at mid-rapidity using the decay to an electron–positron pair. PHENIX found 9.9 $\pm$ 4.1 (stat) $\pm$ 1 (syst) $\mathrm {J}/\psi$ candidates. The smallness of the sample did not allow one to separate the coherent and incoherent contributions. Their measurement corresponded to $W_{\gamma \mathrm {Au}}\approx 24$ GeV ($x\approx 1.5\cdot 10^{-2}$). The cross section for Au–Au UPC at mid-rapidity was measured to be 76 $\pm$ 31 (stat) $\pm$ 15 (syst) $\mu$b, which agreed, within the errors, with the theoretical models available at that time [773, 776–778]. Although the large experimental errors precluded setting strong constraints on the models, this study was very important as a proof of principle.

#### Coherent production of $\mathrm {J}/\psi$ in Pb–Pb UPC at the LHC

The coherent photonuclear production of $\mathrm {J}/\psi$ has been measured in three different rapidity (equivalently $W_{\gamma \mathrm {Pb}}$) ranges at the LHC. ALICE has measured it at mid- [779] and forward rapidity [780], while CMS has recently released preliminary results at semi-forward rapidities [781]. Table 17 summarises these measurements.

The ALICE detector [782] measures charmonium either in the central barrel using a combination of silicon trackers (ITS), a time projection chamber (TPC) and a time of flight system (TOF); or in the forward part where a muon spectrometer is installed. In addition to requiring the decay products of the charmonium to be either in the central barrel or in the muon spectrometer, the exclusivity condition is realised vetoing activity in a set of two scintillator arrays (VZERO) which cover 4 units of rapidity in the forward/backward region, while the absence of neutrons at zero degrees or the measurements of one or few of them is performed with zero-degree calorimeters (ZDC) located 116 m away and on both sides of the interaction point.

The first measurement of coherent production of $$\mathrm {J}/\psi $$ in Pb–Pb UPC was performed by ALICE using the muon spectrometer [780]. The trigger required a muon above threshold (1 GeV/*c* of transverse momentum) and no activity in the opposite side of the detector. The coherent contribution was obtained selecting candidates with transverse momentum less than 0.3 GeV/*c*. The cross section in Pb–Pb UPC was measured to be 1.00 $$\pm $$ 0.18(stat) $$^{+0.24}_{-0.26}$$ (syst) mb. As this measurement is performed at large rapidities, the dominant contribution to the cross section (about 95 %) originates from the low energy part of the flux (see Fig. 99) with the corresponding average energy in the centre-of-mass system of the photon and the target being $$W_{\gamma \mathrm {Pb}}\approx 20$$ GeV ($$x\approx 2.2\cdot 10^{-2}$$). The second measurement was performed at mid-rapidity using the central barrel detectors [779]. The trigger required hits in ITS and TOF (in TOF with a back-to-back topology) and absence of activity in VZERO. In this case, using the PID capabilities of the ALICE TPC, two decay channels have been used: $$\mu ^{+}\mu ^{-}$$ and $$e^{+}e^{-}$$. The transverse momentum distribution of the $$\mathrm {J}/\psi $$ candidates was used to extract the coherent contribution. The measured Pb–Pb UPC coherent cross section was 2.38 $$\pm $$$$^{0.34}_{0.24}$$ (stat $$+$$ syst) mb and corresponded to $$W_{\gamma \mathrm {Pb}}\approx 92$$ GeV ($$x\approx 10^{-3}$$). For this sample, the fraction of coherent events with no activity in the ZDC was measured to be 0.70 $$\pm $$ 0.05 (stat).

The central barrel of the CMS detector [783] contains a silicon pixel and strip tracker, a lead tungstate crystal electromagnetic calorimeter and a brass/scintillator hadron calorimeter; all of them within a superconducting solenoid of 6 m internal diameter, providing a magnetic field of 3.8 T. Muons are measured within pseudo-rapidity $|\eta |< 2.4$ by gas-ionisation detectors embedded in the steel return yoke outside the solenoid. The UPC trigger used by CMS requires (i) the presence of at least one muon candidate with a minimal transverse-momentum threshold, (ii) at least one track in the pixel detector, (iii) rejection of events with activity in the scintillator counters covering the pseudo-rapidity range between 3.9 and 4.4 on both sides of the interaction point and (iv) energy deposit consistent with at least one neutron in either of the ZDCs. This last requirement is similar to what was done by PHENIX and triggers only on a fraction of the cross section. To obtain the total coherent cross section, models of neutron emission in coherent production were used [773, 784]. Note that the CMS result is not corrected for feed-down from $\psi \text {(2S)}$ and that the contribution of both terms in Eq. (67) is important, so that it is not possible to assign a unique value of $W_{\gamma \mathrm {Pb}}$ to this measurement. The preliminary cross section can be found in [781]. It agrees with expectations of models adjusted to describe ALICE data [773, 784, 785].

#### Coherent production of $\psi \text {(2S)}$ in Pb–Pb UPC at the LHC

The ALICE Collaboration carried out a preliminary measurement of the coherent production of $\psi \text {(2S)}$ in Pb–Pb UPC [786] at mid-rapidity using the same trigger and detectors as for the $\mathrm {J}/\psi$ case. The $\psi \text {(2S)}$ was identified in the following decay channels: to $l^{+}l^{-}$ and to $\mathrm {J}/\psi \pi ^{+}\pi ^{-}$, with $\mathrm {J}/\psi \rightarrow l^{+}l^{-}$, where $l=e,\mu$. The right panel of Fig. 100 shows an event display of a coherently produced $\psi \text {(2S)} \rightarrow \mathrm {J}/\psi \pi ^{+} \pi ^{-}$ candidate. A preliminary measurement of the ratio of the cross sections for $\mathrm {J}/\psi$ and $\psi \text {(2S)}$ coherent photonuclear production was carried out as well [786]. This measurement is significantly higher (about a factor of two for the central value) than the ratios 0.166 $\pm$ 0.007(stat) $\pm$ 0.008 (syst) $\pm$ 0.007 (BR), 0.14 $\pm$ 0.05 and 0.19 $\pm$ 0.04 measured by H1 [787], CDF [788] and LHCb [789], respectively.

#### Incoherent production of $\mathrm {J}/\psi$ in Pb–Pb UPC at the LHC

ALICE has also measured the incoherent production of $\mathrm {J}/\psi$ in Pb–Pb UPC at mid-rapidity [779] using the same trigger and detectors as for the coherent case. The incoherent contribution was obtained from the distribution of transverse momentum. The centre of mass energy in the $\gamma$-Pb system is the same as for the coherent case. The measured cross section is 0.98 $^{+0.19}_{-0.17}$ (stat $+$ syst) mb.

#### Coherent photonuclear production of $\mathrm {J}/\psi$ in coincidence with a hadronic Pb–Pb collision at the LHC

When studying the inclusive distribution of transverse momentum of $\mathrm {J}/\psi$ in hadronic Pb–Pb collisions at large rapidities (in the range 2.5 to 4.0), a significant excess of $\mathrm {J}/\psi$ candidates was found for transverse momentum smaller than 0.3 GeV/*c* for the centrality bin 70–90 % [790]. One possible explanation of this observation is the coherent photonuclear production of the $\mathrm {J}/\psi$ in coincidence with a hadronic interaction. Although the possibility of such a process has been discussed in the past [791], currently there is no theoretical calculation available for this process. Such a calculation is a challenge for theorists. Note that an excess is also observed, with reduced significance, in the centrality bin 50–70 % and that there is a framework in place to extract the photonuclear coherent cross section in these cases [792].

### Models for photonuclear production of charmonium

The following models will be discussed in this section:

- AB-AN: Model by Adeluyi and Bertulani  [785] and Adeluyi and Nguyen [793].
- CSS: Model by Cisek, Schäfer and Szczurek  [794].
- KN: Model by Klein and Nystrand implemented in the STARLIGHT Monte Carlo program  [773, 776, 795].
- LM: Model by Lappi and Mantysaari  [796, 797].
- GM-GDGM: Model by Goncalves and Machado  [798] and by Gay-Ducati, Griep and Machado  [799].
- RSZ: Model by Rebyakova, Strikman, and Zhalov  [800].

All models start from Eq. (67) which has two ingredients: the photon flux and the photonuclear cross section. The first difference among the models is that some of them (CSS, LM, GM-GDGM) use the hard-sphere approximation of the photon flux; i.e., Eq. (64), and other models (AB-AN, KN, RSZ-GZ) integrate the convolution of Eq. (63) with the probability of no hadronic interaction.

Regarding the photonuclear cross section the models contain the following ingredients: (i) an assumption on the nuclear distribution in the transverse plane, (ii) an implicit or explicit prescription for the wave function of the vector meson and finally (iii) all models fix some of the parameters using data on exclusive photoproduction of charmonium off the proton and thus have to include a prescription to link the photoproduction off protons with the photonuclear interaction. In this context the models can be grouped in three different classes: models based on the generalised vector dominance model (KN), on LO pQCD (AB-AN, RSZ) and on the colour dipole model (CSS, LM, GM-GDGM).

#### Models based on vector dominance

The only model in this class is KN. There are three main ingredients in this model: (i) the vector dominance model (VDM) relates both the $\gamma \mathrm {+Pb}\rightarrow \mathrm {Pb+V}$ and the $\gamma \mathrm {+p}\rightarrow \mathrm {p+V}$ processes to $\mathrm {Pb+V}\rightarrow \mathrm {Pb+V}$ and $\mathrm {p+V}\rightarrow \mathrm {p+V}$ respectively (Here *V* represents a vector meson.); (ii) the optical theorem relates these last processes to the total cross section; (iii) a classical Glauber model relates the total production cross section off a proton, to that off a nucleus.

In more detail:

70 $$\begin{aligned} \sigma _{\gamma \mathrm Pb}(y)\equiv & {} \sigma (\gamma \mathrm{+ Pb}\rightarrow \mathrm{V+Pb}) \nonumber \\= & {} \left. \frac{\mathrm {d}\sigma (\gamma \mathrm{+ Pb}\rightarrow \mathrm{V+Pb}) }{\mathrm {d}t} \right| _{t=0} \int ^\infty _{t_{min}} \mathrm {d}t |F(t)|^2,\nonumber \\ \end{aligned}$$

where *F*(*t*) is the nuclear form factor and *t* the momentum transferred to the nucleus. Using VDM and the optical theorem yields

71 $$\begin{aligned} \left. \frac{\mathrm {d}\sigma (\gamma \mathrm{+ Pb}\rightarrow \mathrm{V+Pb}) }{\mathrm {d}t} \right| _{t=0} = \frac{\alpha \sigma ^2_\mathrm{TOT}(\mathrm{V+Pb})}{4f^2_V},\nonumber \\ \end{aligned}$$

where $f_V$ is the vector meson photon coupling. A classical Glauber model produces

72 $$\begin{aligned} \sigma _\mathrm{TOT}(\mathrm{V+Pb})\approx & {} \sigma _\mathrm{inel}(\mathrm{V+Pb})\nonumber \\= & {} \int \mathrm {d}^2\vec {b}( 1-\exp [-\sigma _\mathrm{TOT}(\mathrm{V+p}) T_\mathrm{Pb}(\vec {b}) ] ),\nonumber \\ \end{aligned}$$

where $T_{\mathrm{Pb}}$ is the nuclear thickness function and $\sigma _\mathrm{TOT}(\mathrm{p+V})$ is obtained from the optical theorem, now applied at the nucleon level

73 $$\begin{aligned} \sigma ^2_\mathrm{TOT}(\mathrm{V+p}) = 16\pi \left. \frac{\mathrm {d}\sigma (\mathrm{V+p}\rightarrow \mathrm{V+p}) }{\mathrm {d}t} \right| _{t=0}. \end{aligned}$$

Using VDM leads to

74 $$\begin{aligned} \left. \frac{\mathrm {d}\sigma (\mathrm{V\!+\!p}\!\rightarrow \!\mathrm{V\!+\!p}) }{\mathrm {d}t} \right| _{t=0} = \frac{f^2_V}{4\pi \alpha } \left. \frac{\mathrm {d}\sigma (\gamma \mathrm{+ p}\rightarrow \mathrm{V+p}) }{\mathrm {d}t} \right| _{t=0},\nonumber \\ \end{aligned}$$

where the elementary cross section

75 $$\begin{aligned} \left. \frac{\mathrm {d}\sigma (\gamma \mathrm{+ p}\rightarrow \mathrm{V+p}) }{\mathrm {d}t} \right| _{t=0} = b_V( XW^\epsilon _{\gamma \mathrm p} + YW^{-\eta }_{\gamma \mathrm p}) \end{aligned}$$

is fitted to experimental data to obtain the values for the *X*, *Y*, $\epsilon$, $\eta$ and $b_V$ parameters.

#### Models based on LO pQCD

These models start from Eq. (70) and use the LO pQCD calculation [801, 802] for the forward cross section

76 $$\begin{aligned} \left. \frac{\mathrm {d}\sigma (\gamma \mathrm{+ Pb}\rightarrow \mathrm{V+Pb}) }{\mathrm {d}t} \right| _{t=0}= \frac{16\pi ^3\alpha ^2_s \Gamma _{ee}}{3\alpha M^5} [ xG_A(x,Q^2) ]^2, \nonumber \\ \end{aligned}$$

where $\Gamma _{ee}$ is the decay width to electrons and $G_A$ is the nuclear gluon density distribution at a scale $Q^2$, which for the models described below was chosen to be $Q^2 = M^2/4$, although other options are possible and may describe better the experimental data [803]. It is important to note that this equation contains implicitly a model for the wave function of the vector meson, but in the final result the only trace of it is the presence of $\Gamma _{ee}$.

The AB-AN model modifies Eq. (76) by adding a normalisation parameter to the right side, which should take into account effects missing in the approximation. This factor is then fitted to reproduce HERA data using the same type of equation applied to the $\gamma \mathrm { + p}\rightarrow \mathrm {p}+\mathrm {J}/\psi$ case. Nuclear effects are modelled as $G_A(x,Q^2) = g_p(x,Q^2) R^A_g(x,Q^2)$ where $g_p$ is the gluon distribution in the proton and $R^A_g$ is the nuclear modification factor of the gluon distribution. MSTW08 [804] is used for the gluon distribution in the proton, while several different choices are made for $R^A_g$ to estimate nuclear effects: EPS08 [805], EPS09 [364], HKN07 [806] and $R^A_g = 1$ to model the absence of nuclear effects.

The RSZ model computes $R^A_g$ in the leading twist approach to nuclear shadowing [807]. The main ingredients are the factorisation theorem for hard diffraction and the theory of inelastic shadowing by Gribov. The evolution is done using the DGLAP equations. The experimental input to fix the parameters of the model is given by inclusive diffractive parton distribution functions of nucleons as measured at HERA. For the gluon distribution in the proton the LO distribution from [808] was used.

**Fig. 101:**
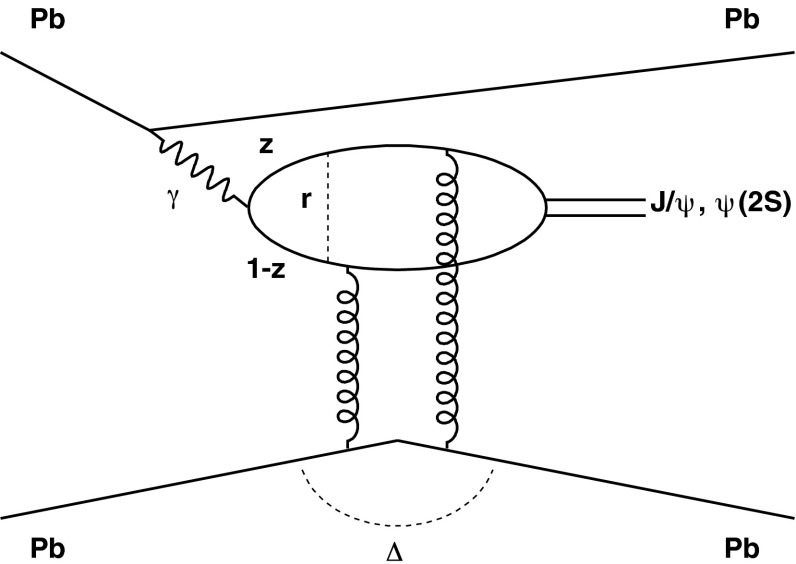
Lowest-order diagram for the photoproduction of charmonium within the colour dipole model

#### Models based on the colour dipole approach

The basic idea of this formalism is illustrated in Fig. 101: long before the interaction, the photon splits into a quark-antiquark pair, which forms a colour dipole. Then this dipole interacts with the target and after another long time the dipole creates a vector meson. The cross section in this formalism is given by

77 $$\begin{aligned} \frac{\mathrm {d}\sigma (\gamma \mathrm{+Pb}\rightarrow \mathrm {J}/\psi + \mathrm{Pb}) }{\mathrm {d}t} = \frac{R^2_g(1+\beta ^2)}{16\pi }\left| A(x,Q^2,\vec {\Delta })\right| ^2,\nonumber \\ \end{aligned}$$

where the so-called skewedness correction $R^2_g$ compensate for the fact that only one value of *x* is used, even though the two gluons participating in the interaction have different *x* [809], while $(1+\beta ^2)$ is the correction that takes into account the contribution from the real part of the amplitude [810]. The imaginary part of the scattering amplitude is given by

78 $$\begin{aligned} A(x,Q^2,\vec {\Delta })= & {} \int \mathrm {d}z \mathrm {d}^2\vec {r}\mathrm {d}^2\vec {b} e^{-i(\vec {b}-(1-z)\vec {r})\cdot \vec {\Delta }} [\Psi ^*_{\mathrm {J}/\psi }\Psi ]\nonumber \\&\times 2\left[ 1-\exp \left\{ -\frac{1}{2}\sigma _\mathrm{dip}T_\mathrm{Pb}(b) \right\} \right] , \end{aligned}$$

where the integration variable $\vec {r}$ represents the distance between the quark and the antiquark in the plane transverse to the collision, *z* quantifies the fraction of the photon momentum carried by the quark and *b* is the distance between the centres of the target and the dipole; $\vec {\Delta }$ is the transverse momentum transferred to the nucleus; the virtuality of the incoming photon is denoted by $Q^2$ and for the case of photoproduction discussed here is zero; $\Psi$ describes the splitting of the photon into the dipole and $\Psi _{\mathrm {J}/\psi }$ is the wave function of the $\mathrm {J}/\psi$; the term $i(1-z)\vec {r}\cdot \vec {\Delta }$ in the exponential is a third correction to take into account non-forward contributions to the wave function $\Psi _{\mathrm {J}/\psi }$, which is modelled for the forward case [811]; and finally $\sigma _\mathrm{dip}$ is the universal cross section for the interaction of a colour dipole with a nuclear target. The models differ in the functional form of $\Psi _{\mathrm {J}/\psi }$, in the corrections that were considered and in the formulation of the universal dipole cross section.

In the case of LM the non-forward correction to the wave function was not considered. LM use two different prescriptions for the wave function: the Gauss-LC [812] and the boosted Gaussian [813, 814]. $\sigma _\mathrm{dip}$ is written in terms of the cross section of a dipole and a proton, $\sigma ^p_\mathrm{dip}$; assuming a Gaussian profile in impact parameter for the proton, $\exp (-b^2/(2B_p))$:

79 $$\begin{aligned} \frac{1}{2}\sigma _\mathrm{dip} = 2\pi B_p A N(r,x), \end{aligned}$$

where *N*(*r*, *x*) is the dipole target amplitude. LM use two different models for *N*(*r*, *x*): the IIM model [815], which is a parametrisation of the expected behaviour of the solution to the BK equation [816–818], which includes a non-linear term for the evolution of *N*(*r*, *x*); and the IPsat model [812, 819], which uses DGLAP equations to evolve an eikonalised gluon distribution.

The GM-GDGM model uses the boosted Gaussian prescription for the wave function. The dipole cross section is given by $\sigma _\mathrm{dip} = R^A_g(x,Q^2)\sigma ^\mathrm{p}_\mathrm{dip}$, where $\sigma ^\mathrm{p}_\mathrm{dip}$ is given according to the IIM model and the leading twist approximation is used for $R^A_g(x,Q^2)$. The CSS model is similar to the GM model, but it uses the unintegrated gluon distribution of the nucleus including multiple scattering corrections. It also takes into account higher-order fluctuations of the incoming photon. The Gaussian form of the wave function is used.

**Fig. 102:**
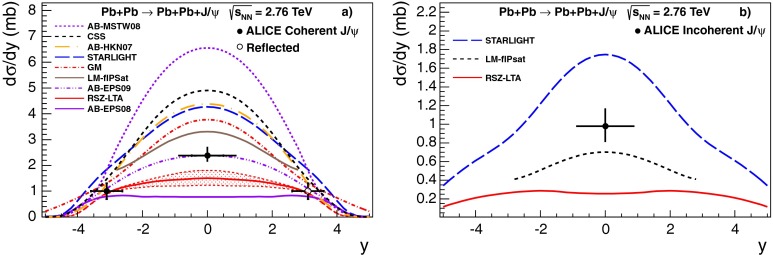
Cross section for coherent (*left*) and incoherent (*right*) photonuclear production of $\mathrm {J}/\psi$ as measured by ALICE [779] compared to theoretical predictions

### Photonuclear production of charmonium: comparing models to measurements

The comparison of the cross sections measured with ALICE and the predictions of the different models is shown in Fig. 102. Several comments are in order. The spread among the different predictions is quite large, so data impose strong constraints into the different ingredients of the models, in particular at mid-rapidity, which corresponds to the smallest *x*–Bjorken in a pQCD interpretation. The left panel of the figure shows the measurement for $\mathrm {J}/\psi$ coherent production. The AB-MSTW08 curve, corresponding to the absence of nuclear effect in that model, and the AB-EPS08 model, corresponding to strong shadowing, are both disfavoured. The other models are closer to the data and there are several natural refinements that can be done. For example, after the publication of ALICE data the model labelled RSZ-LTA was revisited and a study of the variations of the model with different scales for the coupling constant was performed [803]. It was found that using data, one could constrain within this model the value of this scale such that data are correctly described. Similar improvements could be made to other models. The preliminary data from CMS (not shown in the figure) is also well described by this updated model and by AB-EPS09, which corresponds to mild shadowing.

The situation with respect to the measurement of incoherent photonuclear production of $\mathrm {J}/\psi$ is not that clear. There are less models and less data. The LM–fIPsat model slightly underestimates the data, while the prediction of the same model is above the data for the coherent case. The modification of the RSZ-LTA model from [803] has not such a large effect in the incoherent case, so that this model is still below the measurement.

The prediction of the cross section for coherent photonuclear production of $\psi \text {(2S)}$ and its comparison to data is even more difficult. As mentioned above, all models fixed the predictions such that they reproduce data from HERA on photoproduction off protons. But HERA data for $\psi \text {(2S)}$ is less abundant and less precise than for the case of $\mathrm {J}/\psi$. One of the consequences is that the STARLIGHT and AB models when all nuclear effects are switched off, have the same prediction for $\mathrm {J}/\psi$ but a different prediction for $\psi \text {(2S)}$. It also happens that the wave function of the $\psi \text {(2S)}$ is more complex than that of the $\mathrm {J}/\psi$ [820] and not all models, see for example [793], take into account the full complexity of the wave function. Finally, the preliminary results from ALICE for the coherent production of $\psi \text {(2S)}$ appeared after the publication of the $\mathrm {J}/\psi$ measurements, so that some models were already updated to improve the description of ALICE data. Taking into account these caveats the general conclusion seems to be that models with strong shadowing or without any nuclear effects are disfavoured, while models incorporating a mild form of shadowing are, within the current large experimental uncertainties, relatively close to data.

A somehow surprising result was the ratio of the coherent cross section of $\psi \text {(2S)}$ to that of $\mathrm {J}/\psi$ at mid-rapidity. The measured value is around two times larger than what has been measured in the photoproduction off protons, while most models expected these ratios to be similar [786]. Note that the experimental uncertainties are still quite large, so this discrepancy is only a bit larger than two sigmas. All models, except AB, are quite close to the ratio measured at HERA, and thus far from that measured by ALICE [786]. The AB-EPS09 model is closer to the measured ratio, but one has to say that the wave function of the $\psi \text {(2S)}$ was not included in its full complexity, and it is not clear what would happen if it were included. In this case, new data and improved models are much needed.

Another area to watch is the measurement of coherent production of charmonium in peripheral or even semi-central Pb–Pb collisions. As mentioned above, this possibility was discussed some time ago [791], but no full calculation of the process has been performed. The possibility of a measurement at different centralities, which will be possible during Run 2, will allow one to test different model implementations. For example, if the coherent source were the spectator nucleons, then the distribution of transverse momentum would depend on the centrality.

### Summary and outlook

The LHC Collaborations have demonstrated with data from Run 1 that measurements of photonuclear production of charmonium are possible and that these measurements provide valuable information to constrain models and can contribute to a better understanding of shadowing. Present data favour the existence of a shadowing as predicted by EPS09 in the x range $10^{-3}$–10$^{-2}$. Furthermore, these data have given a glimpse of two remarkable results: (i) the ratio of $\psi \text {(2S)}$ to $\mathrm {J}/\psi$ cross section for coherent photoproduction seems to be sensitive to nuclear effects; and (ii) it seems to be possible to measure coherent production of charmonium overlapped with hadronic collisions. During Run 2, new data with large statistics will be collected and a definitive answer to these two questions may be given. The new data will also allow one to explore the dependence of the $\mathrm {J}/\psi$ coherent cross section on transverse momentum with great detail and potentially the measurement of $\Upsilon$ production. The already existing measurements and the forthcoming ones represent important milestones in the path going from HERA towards a future dedicated electron–ion facility [821, 822].

## Upgrade programmes and planned experiments

### Introduction

As seen in the previous sections, and also summarised in the next one, alongside the great progress in understanding the physics of heavy quarks in proton–proton and heavy-ion collisions, a lot of questions emerged too. Those, as well as the quest for a quantitative description of the hot deconfined quark–gluon matter, call for upgrades in existing experiments and also for new ones, in which the potential of heavy quarks in answering those questions is fully exploited. Below, we discuss the ongoing efforts and the possibilities for new experiments.

### Collider experiments

#### The LHC upgrade programme

The LHC roadmap foresees three long shut-downs (LS) of the machine in order to perform major upgrades. The objective of LS1, recently completed, was the preparation of the machine for 6.5–7 $$\text {TeV}$$ operation in 2015 [823] reaching (close to) the design energy and the nominal peak luminosity of $$\mathcal {L} _\mathrm{pp}=10^{34}~\mathrm{cm}^{-2}\,\mathrm{s}^{-1}$$ or even higher. For heavy ions, interaction rates of 10–15 kHz are expected in Pb–Pb in Run 2. The goal of the heavy-ion programme for Run 2 is to collect about a factor 10 more statistics for Pb–Pb collisions with respect to Run 1, while the new energy of $$\sqrt{s_{\mathrm{NN}}}$$$$\simeq $$5 $$\text {TeV}$$ will push the frontier of high-energy-density quark–gluon matter.

LS2, scheduled for 2018–2019, will be mainly devoted to a major upgrade of the injectors as well as interventions performed on the LHC itself aiming at increasing its instantaneous luminosity in $\mathrm pp$ and heavy-ion running modes to $\mathcal {L} _\mathrm{pp}=3\cdot 10^{34}~\mathrm{cm}^{-2}\mathrm{s}^{-1}$ and $\mathcal {L} _\mathrm{PbPb}=6\cdot 10^{27}~\mathrm{cm}^{-2}\mathrm{s}^{-1}$, respectively. After LS2, the interaction rate in Pb–Pb collisions is foreseen to be 50 kHz. After LS3, envisaged in 2023–2024, the LHC peak luminosity is expected to reach $\mathcal {L} _\mathrm{pp}=$5–7$\cdot 10^{34}~\mathrm{cm}^{-2}\,\mathrm{s}^{-1}$ levelled down from higher luminosities [824] (HL-LHC). The LHC heavy-ion programme is currently planned to extend to Run 4 (2026–2028).

**Fig. 103:**
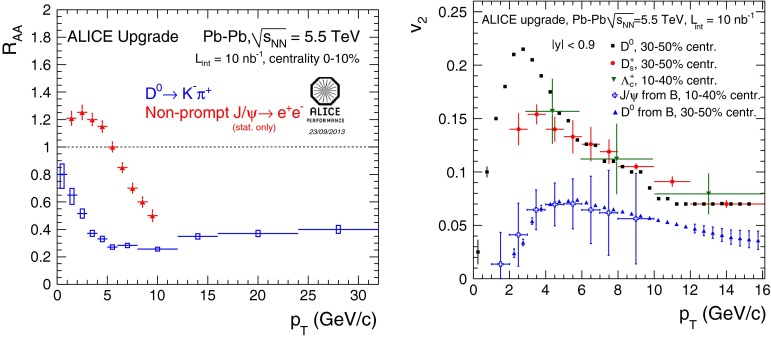
Estimated performance of open heavy flavour nuclear modification factor (*left*) and elliptic flow (*right*) with the ALICE upgrade. The $p_{\mathrm {T}}$ dependence of the two observables is assumed based on current measurements and model predictions [829]

The four major LHC experiments, ALICE, ATLAS, CMS and LHCb, have rich detector upgrade programmes to fully exploit the accelerator upgrades. Three different phases, corresponding to the three LHC long shut-downs towards the HL-LHC, are planned. Heavy-flavour and quarkonium physics, both in $\mathrm pp$ and Pb–Pb collisions, either drive or strongly benefit from the upgrade programmes.

ALICE is the dedicated heavy-ion experiment, whose strengths, unique at the LHC, are measurements at low $p_{\mathrm {T}}$ and involving a wide range of identified hadrons, and access to forward rapidities ($y\sim 4$) in muon decay channels. This implies primarily minimum-bias (or centrality-selected) collisions, as trigger selectivity for low-$p_{\mathrm {T}}$ observables is obviously weak.

The ATLAS and CMS experiments are general-purpose experiments designed primarily for the investigations of $\mathrm pp$ collisions, the Higgs boson discovery [825, 826] being the major achievement of Run 1 for this physics programme. Both detectors have demonstrated very good performance in heavy-ion collisions too, including measurements devoted to heavy-quarks, as seen in the previous sections. The upgrade programmes of both ATLAS and CMS detectors will extend the studies of Higgs and of physics beyond the standard model with improved detector performances to match the LHC luminosity increases. This will benefit the ATLAS and CMS heavy-ion physics programme as well which is focussed on higher $p_{\mathrm {T}}$, complementary to the ALICE programme.

The LHCb experiment is the dedicated experiment for the studies of *b*-quark physics in $\mathrm pp$ collisions. The measurements performed by LHCb, for both charm and beauty hadrons, in $\mathrm pp$ and p–Pb collisions are of a large variety and unique quality. The upgrades of the LHCb detector retain the focus on heavy-flavour studies, which will be performed in $\mathrm pp$ and p–Pb collisions, as well as in a fixed-target configuration.

*The ALICE experiment* The ALICE Collaboration consolidated and completed the installation of current detectors during LS1 with the aim to accumulate 1 $\text {~nb}^{-1}$ of Pb–Pb collisions during Run 2 corresponding to about ten times the Run 1 integrated luminosity. In parallel, the ALICE Collaboration makes a major effort to upgrade the apparatus, in particular to improve the tracking precision and to enable the read-out of all interactions at 50 kHz, with the goal to accumulate 10 $\text {~nb}^{-1}$ of Pb–Pb collisions after LS2. A low-B field (0.2 T) run to collect 3 $\text {~nb}^{-1}$ is also envisaged. The implementation of this upgrade programme [827, 828], foreseen in LS2, includes: a new low material Inner Tracking System [829] with a forward-rapidity extension (MFT [830]) to add vertexing capabilities to the current Muon Spectrometer; the replacement of the Time Projection Chamber (TPC) wire chambers with gas electron multiplier (GEM) read-out; a new read-out electronics for most of the detectors and an updated trigger system; a new set of forward trigger detectors [831] and a new integrated online–offline system.

The new Inner Tracking System [829], covering mid-rapidity ($|\eta |<1.3$), consists of seven concentric layers. For the forward region ($2.5<\eta <3.6$) muon tracker (MFT), five detection planes are envisaged. Both systems are composed of CMOS Monolithic Active Pixel Sensors with a pixel cell size of about $20\times 30$ $\upmu$m. At mid-rapidity, the total material budget per layer is 0.3 and 0.8 % of $X_0$ for the three inner and four outer layers, respectively. It is 0.6 % of $X_0$ per detection plane at forward rapidity [830]. These low material budget, high granularity detectors, in conjunction with the reduction of the beam pipe diameter in the centre of the ALICE detector from the present value of 58–36 mm, lead to a significantly improved measurement of the track impact parameter (distance of closest approach to the primary vertex). It reaches 40 $\upmu$m at $p_{\mathrm {T}}$  $\simeq$ 0.5$~\text {GeV}/c$ at mid-rapidity and 90 $\upmu$m at $p_{\mathrm {T}}$  $\simeq$ 1$~\text {GeV}/c$ at forward rapidity.

Thanks to this improved performance and the high statistics, a large number of new measurements become possible and the existing measurements will be repeated with improved performance. In the charm sector, at mid-rapidity the $p_{\mathrm {T}}$ coverage will be extended towards zero $p_{\mathrm {T}}$ and the uncertainties will be significantly reduced for the D and $\mathrm {D}_{s}$ meson nuclear modification factor and $v_{2}$. The $\Lambda _c$ baryon reconstruction in its three-prong decay (p, K and $\pi$) will become possible down to $p_{\mathrm {T}}$ = 2$~\text {GeV}/c$, allowing for the measurement of baryon/meson ratio ($\Lambda _c$/D) crucial for the study of thermalisation and hadronisation of charm quarks in the medium. Two-particle correlation studies with a D meson as “trigger” particles (see Sect. 4) as well as a measurement of the D-jet fragmentation function will be performed. At forward rapidity the separation of open charm and open beauty production cross sections can be performed via the semi-muonic and $\mathrm {J}/\psi$ decay channels.

The ALICE detector upgrade opens up the possibility to fully reconstruct B$^{+}$ meson (B$^{+} \rightarrow \overline{\mathrm {D}^0}\pi ^{+}$) down to $p_{\mathrm {T}}$ = 2$~\text {GeV}/c$ and the $\Lambda _b$ baryon ($\Lambda _b\rightarrow \Lambda _c^{+}\pi ^{-}$) down to $p_{\mathrm {T}}$ = 7$~\text {GeV}/c$. The thermalisation of *b*-quarks in the medium will be studied via the measurement of the elliptic flow in the semi-leptonic as well as $\mathrm {J}/\psi$ or D-meson decay channels, both at mid- and forward rapidities. Figure 103 gives an example of the expected performance of open heavy flavour nuclear modification factor and elliptic flow measurements [829].

**Fig. 104:**
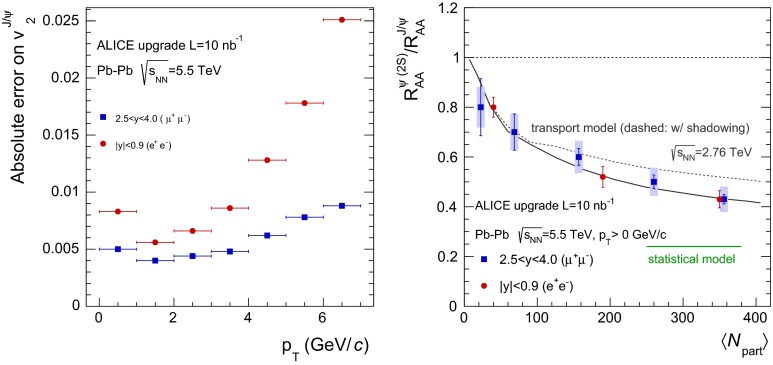
*Left* estimated statistical uncertainties of $v_{2}$ measurement of $\mathrm {J}/\psi$ with the ALICE upgrade [827]. *Right* estimated performance of the measurement of ratio of the nuclear modification factor of $\psi \text {(2S)}$ and $\mathrm {J}/\psi$  [828] compared to two charmonium production model calculations [728, 832]

The ALICE detector upgrade will lead to a significant improvement of the (prompt) $\mathrm {J}/\psi$ measurement, both at mid- and forward rapidities. The expected performance of the $v_{2}$ measurement is illustrated in Fig. 104 (left). At forward rapidity, the measurement of $\mathrm {J}/\psi$ polarisation in Pb–Pb collisions will become possible, as well as precision measurements of the production of $\Upsilon$ states.

The measurement of the $\psi \text {(2S)}$ meson, combined with the $\mathrm {J}/\psi$ measurement, offers an important tool to discriminate between different charmonium production models. The $\psi \text {(2S)}$ measurement is a challenge in heavy-ion experiments, in particular at low $p_{\mathrm {T}}$. At forward rapidity, the addition of the MFT will allow a precise measurement to be made of $\psi \text {(2S)}$ down to zero $p_{\mathrm {T}}$ even in the most central Pb–Pb collisions. At mid-rapidity the measurement remains very challenging, but a significant result is expected with the full statistics of the ALICE data after the upgrade (Fig. 104 right).

Comparable detection performances at mid and forward rapidities will place the ALICE experiment in the position of studying the heavy flavour QGP probes as a function of rapidity. This will help imposing tighter experimental constraints to the theoretical models.

The physics programme for ultra-peripheral AA collisions (UPC, see Sect. 6) in ALICE for Run 3 and Run 4 will have the advantage of increased luminosity by a factor of 10 (100) with respect to Run 2 (Run 1). The large increase in statistics could as well allow for the detailed study of processes with a small cross section like the coherent production of $\Upsilon$ or the production of $\eta _c$ in $\gamma \gamma$ collisions.

*The ATLAS experiment* During LS1, ATLAS achieved the installation of the Insertable B-Layer (IBL) [833], which is an additional fourth pixel layer, placed closer to the beam pipe at an average radius of 33 mm. It will add redundancy to the inner tracking system, leading to improved tracking robustness. This new layer provides improved pointing resolution of the inner tracker for $p_{\mathrm {T}}$ as low as 1$~\text {GeV}/c$ and a pseudo-proper decay length resolution improved by about 30 % compared to Run 1, leading to an improved *b*-quark tagging performance [834]. The inner tracker will be completely replaced during the LS3  [835] in order to cope with the high luminosity after LS3 of the LHC. This new tracker, composed of silicon pixel and strip layers, will have capabilities equivalent to the current tracker (with the IBL).

During LS2, ATLAS envisages the installation of new Muon Small Wheels and more selective (“topological”) Level-1 trigger criteria [836, 837], which carry the potential to improve the dimuon acceptance at low $p_{\mathrm {T}}$. The cavern background leads to fake triggers in the forward muon spectrometer, with adverse impact on its physics capability. In order to be able to handle the high luminosity, it is proposed to replace the first end-cap station by the New Small Wheel (NSW) covering the rapidity range $1.2<|\eta |<2.4$. The NSW will be integrated into the Level-1 trigger, improving the background rejection. Two technologies, MicroMegas detectors and small-strip Thin Gap chambers, will be used in order to have both a good position resolution ($<100~\upmu$m) and a fast trigger function. A longer upgrade plan of the muon end-caps, consisting in the extension of the muon reconstruction coverage to higher rapidities, is under study. One of the possible scenarios consists in the addition of a warm toroid at small angles combined with new muon chambers at high rapidity. The extension would cover the rapidity range $2.5<|y|<4.0$ and should have a $p_{\mathrm {T}}$ resolution of the order of 15–40 % for $p_{\mathrm {T}}$ ranging from 10 to 100$~\text {GeV}/c$.

The high luminosity of Run 2 and Run 3 imposes to rise the muon $$p_{\mathrm {T}}$$ trigger threshold. In order to partially limit this increase, a new Level-1 topological trigger algorithm has been implemented. A $$p_{\mathrm {T}}$$ threshold of the order of 15$$~\text {GeV}/c$$ at the $$\mathrm {J}/\psi $$ mass which will increase to 30$$~\text {GeV}/c$$ for higher luminosities is foreseen. The ATLAS quarkonia and heavy flavour programmes will therefore concentrate on the high-$$p_{\mathrm {T}}$$ range ($$p_{\mathrm {T}}$$$$\gtrsim $$ 30$$~\text {GeV}/c$$).

**Fig. 105:**
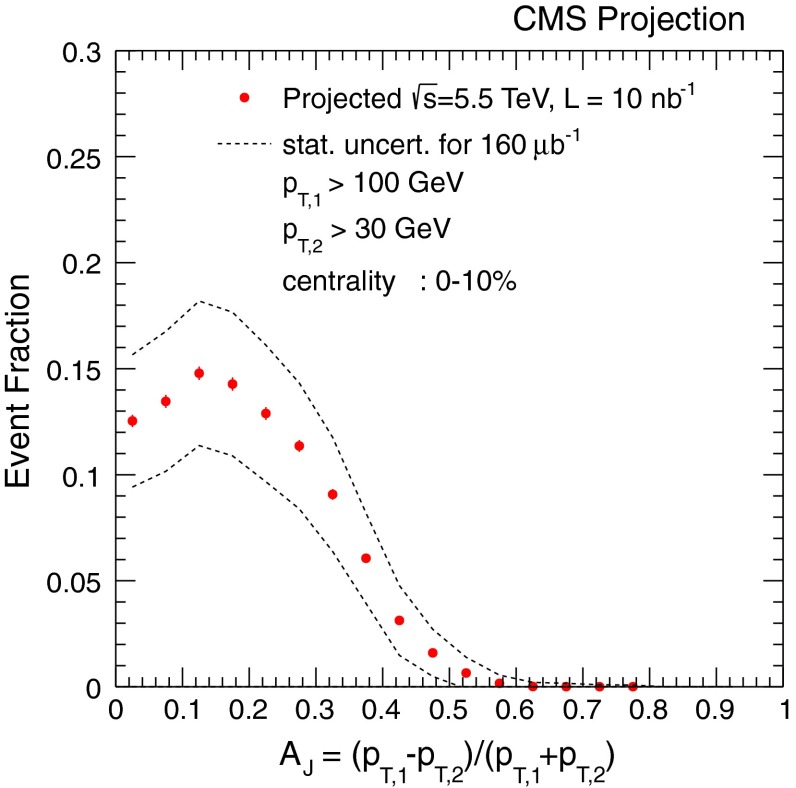
Estimated performance of the measurement in CMS of the doubly tagged *b*-jet asymmetry $A_J$ distribution in $|\eta | < 2$ [841]

*The CMS experiment* Two phases compose the CMS experiment upgrade programme. The first one, spread over LS1 and LS2, involves consolidation of the current detectors. The upgrade activities in LS1 and LS2 are focussed on the inner pixel detector, the hadron calorimeter, the forward muon systems, and the Level-1 trigger [838–840]. The entire pixel detector will be replaced during the 2016–2017 yearly shut-down. The new device adds a fourth detection layer for redundancy in tracking and leads to an improved fake-track rejection. The pointing resolution will be improved thanks to the reduced material budget and by moving the first detection layer closer to the interaction point, which will substantially improve the *b*-quark tagging capability. The upgrade in the hadron calorimeter Level-1 trigger is motivated by the heavy-ion programme; the significantly improved selectivity for high-$p_{\mathrm {T}}$ jets opens up precision measurements of *b*-tagged jets in Pb–Pb collisions. An illustration of the expected performance is given in Fig. 105 for the doubly tagged *b*-jet asymmetry parameter $A_J$ [841].

The muon system will be completed during LS2 by adding a fourth end-cap layer for $1.2<|\eta |<1.8$ and by improving the read-out granularity at mid-rapidity. During LS1, Level-1 hardware trigger algorithms was upgraded, resulting in an improved muon trigger selectivity [841]. The impact on the HI physics programme will be a better non-prompt $\mathrm {J}/\psi$ extraction and an improved dimuon mass resolution.

The second phase of the CMS upgrades [842] planned to be completed during LS3, leads to the readiness of the experiment for physics at the HL-LHC. This includes the extension of the inner tracking system to $|\eta |<4$ with triggering capability and additional muon redundancy with a possible extension of the muon system to cover $|\eta |<4$. It will result in an improved mass resolution (gain of 1.2–1.5) with an improved quarkonia triggering performance. High-statistics measurements of prompt and non-prompt $\mathrm {J}/\psi$, $\psi \text {(2S)}$ and $\Upsilon$ states will become available [841].

With regard to UPC (see Sect. 6), thanks to the increase of statistics expected in Run 3, and pending detail performance studies, CMS would be able to carry out systematic studies for the various $\Upsilon$ states, as well as detailed studies on UPC dijets produced in photon–nucleus collisions.

*The LHCb experiment* The upgrade programme of the LHCb Collaboration [843] has two major facets: (i) the replacement of all front-end electronics,^29^ which will enable continuous detector read-out at 40 MHz, followed by a full software trigger [844], (ii) detector upgrades designed for operation at a luminosity increased by a factor of 5 compared to current conditions (levelled nominal luminosity will be 2$\cdot 10^{33}$ cm$^{-2}~\mathrm{s}^{-1}$ in pp collisions). This comprises the replacement of the VELO silicon vertex detector [845], new tracker systems before and after the dipole magnet [846], and major upgrades for the systems performing particle identification: the RICH, the calorimeter and the muon system [847].

The upgraded LHCb detector components will be installed during LS2 and is envisaged to collect a pp data sample of at least 50 $\text {~fb}^\text {-1}$. This will significantly enhance the unique physics capability of LHCb for heavy-flavour measurements also in p–Pb collisions. The focus is on rare observables in connection to physics beyond the standard model, but the high-precision measurements of production cross sections for quarkonia and open charm hadrons is a direct bonus.

#### The RHIC programme

The current plans [848] envisage measurement at RHIC up to mid-2020s, followed by eRHIC. During the 2014 run, thanks to the full implementation of 3D stochastic cooling, RHIC achieved, in Au–Au collisions at $\sqrt{s_{\mathrm{NN}}}$ = 200 $~\text {GeV}$, an average stored luminosity of $\mathcal {L} =5\cdot 10^{27}$ cm$^{-2}~\mathrm{s}^{-1}$ reaching 25 times the design value. The ongoing measurements focus on heavy flavour probes of QGP exploiting the newly installed silicon vertex detectors in both PHENIX and STAR experiments. These campaigns will extend up to 2016, when the electron cooling of RHIC is expected to enter in operation. The 2015 run modes are scheduled to be $\mathrm pp$, p–Au and possibly p–Al collisions at $\sqrt{s_{\mathrm{NN}}}$ =100$~\text {GeV}$. The RHIC luminosity upgrade plan is to operate the collider in Au–Au collisions at $\sqrt{s_{\mathrm{NN}}}$ = 200 $~\text {GeV}$ at an average stored luminosity of $\mathcal {L} =10^{28}$ cm$^{-2}\mathrm{s}^{-1}$ [849]. The second phase (2018–2019) of the Beam Energy Scan (BES-II), spanning $\sqrt{s_{\mathrm{NN}}}$ = 7–20 $~\text {GeV}$, opens up the potential of heavy flavour measurements at lowest to-date collider energies.

*The sPHENIX project* The PHENIX Collaboration has a radical upgrade plan consisting on replacing the existing PHENIX central detectors, which have small acceptance and lack hadronic calorimetry, with a compact calorimeter and 1.5 T superconducting magnetic solenoid [850]. Full azimuthal calorimeter coverage, both electromagnetic and hadronic, will be available in $$|\eta |<1$$. The sPHENIX detector will be capable of identifying heavy-quark jets and separating $$\Upsilon $$ states in their dielectron decay channel with a mass resolution better than 100 $$\mathrm {MeV}/c^2$$. The current PHENIX detectors will be removed during the 2016 shut-down and sPHENIX detectors will be installed during the 2020 shut-down of RHIC. The sPHENIX running plan consists of two years of data taking (2021–2022) in Au–Au, d–Au and $$\mathrm pp$$ collisions at $$\sqrt{s_{\mathrm{NN}}}$$$$=$$ 200 $$~\text {GeV}$$.

**Fig. 106:**
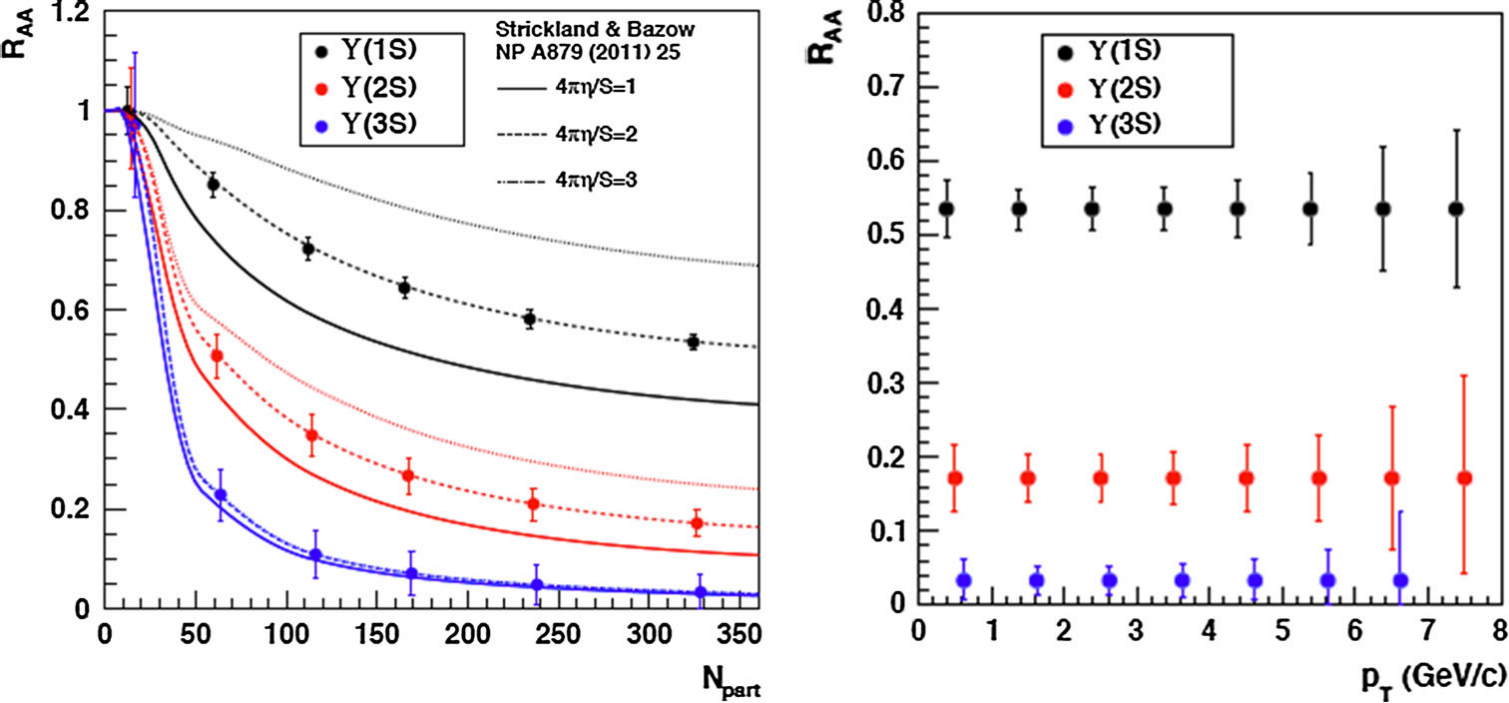
Estimated performance (quoted are the statistical uncertainties) of the measurement of the $\Upsilon$ states $R_{\mathrm {AA}}$ using sPHENIX in Au–Au collisions at $\sqrt{s_{\mathrm{NN}}}$ = 200 $~\text {GeV}$  [851]

**Fig. 107:**
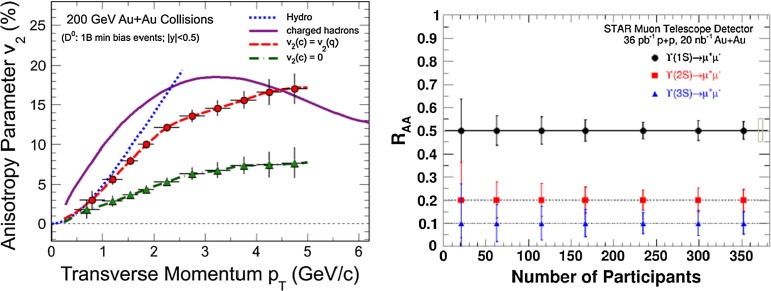
Estimated performance (quoted are the statistical uncertainties) of the STAR measurement of the $\mathrm {D}^0$ meson elliptic flow (*left panel*) and the $\Upsilon$ states $R_{\mathrm {AA}}$ (*right panel*) in Au–Au collisions at $\sqrt{s_{\mathrm{NN}}}$ = 200 $~\text {GeV}$  [852, 853]

The inner tracking system will be composed of the currently existing Silicon Vertex Detector (VTX) with three additional silicon layers at larger radii to improve the momentum resolution and the track-finding capability. The reconstruction efficiency will be as high as 97 % for $$p_{\mathrm {T}}$$$$>2$$$$~\text {GeV}/c$$, with a momentum resolution of the order of 1–1.5 % (depending on $$p_{\mathrm {T}}$$). The pointing resolution will be better than 30 $$\upmu $$m for $$p_{\mathrm {T}}$$$$>3$$$$~\text {GeV}/c$$. The electrons from the $$\Upsilon $$ decays are identified using a combination of the electromagnetic calorimeter and the inner hadron calorimeter with a pion rejection power better than 100 at a 95 % electron efficiency.

Exploiting this very good performance, sPHENIX will be able to measure the suppression pattern of the three $\Upsilon$ states. The high RHIC luminosity and the sPHENIX data acquisition bandwidth (10 kHz) will give to sPHENIX the opportunity to record 10$^{11}$ Au–Au collisions, leading to an unprecedented precision of $\Upsilon$ measurements; see Fig. 106.

Thanks to the combination of its high-precision inner tracking and calorimetry systems, sPHENIX will be able to perform *b*-quark jet tagging by requiring the presence of charged tracks within the jet with a large distance of closest approach to the primary vertex. Simulations show that for jet $p_{\mathrm {T}}$ = 20$~\text {GeV}/c$, a *b*-jet purity of 50 % will be reached with an efficiency of the order of 40–50 % [851], which will allow one to extract *b*-jet $R_{\mathrm {AA}}$ down to $p_{\mathrm {T}}$ = 20$~\text {GeV}/c$ in central Au–Au collisions. Tagging of a *c*-quark jet using the same technique is challenging due to the shorter *c*-hadron lifetime. Nevertheless, *c*-jet tagging performance is under study by associating fully reconstructed D meson with reconstructed jets in the calorimeter. Those heavy-quark jet measurements will be an excellent test of in-medium parton energy-loss mechanisms and will give some insights on the fragmentation functions of heavy quarks.

*STAR experiment* The recently installed Heavy Flavour Tracker (HFT) and Muon Telescope Detector (MTD) will allow for significantly improved measurements for heavy flavour observables already in the 2016 run [852], as illustrated for the $$\mathrm {D}^0$$ meson elliptic flow and the $$\Upsilon $$$$R_{\mathrm {AA}}$$ in Fig. 107. Among the observables expected to become accessible in this run are $$\Lambda _c$$ baryon and open beauty meson production.

The next stages of the STAR upgrade programme [854] include on a short term (with focus on BES-II, 2018–2019) an upgrade of the inner part of the TPC read-out chambers as well as a new Event Plane Detector (EPD) covering the rapidity range $1.5<|y|<5$. The TPC upgrade will improve the tracking and PID performances and extend the TPC coverage from $|y|<1$ to $|y|<1.7$. The long-term part of the upgrade (foreseen for the 2021–2022 heavy-ion programme and the 2025+ eRHIC programme) includes upgrades on the HFT for faster read-out and a forward rapidity system with tracking and calorimetry. The focus will be on measurements relevant for QGP in AA collisions, CNM effects in p–A collisions and for the spin programme at RHIC [848]. All heavy-flavour observables will receive significant improvements in precision in these measurements.

### The fixed-target experiments

Fixed-target experiments using hadron beams have played a major role in quarkonium physics, starting with the co-discovery in 1974 of the $\mathrm {J}/\psi$  [855] at BNL with a 30 GeV proton beam on a Be target, the discovery of the $\Upsilon$ [856] with 400 GeV protons on Cu and Pt targets and the first observation of $h_c$ [857] at Fermilab with antiprotons on an internal hydrogen jet target. In addition, fixed-target experiments have revealed, through high-precision quarkonium studies, many novel and mostly unexpected features of quark and gluon dynamics, which included the anomalous suppression of $\mathrm {J}/\psi$  [660] in Pb–Pb collisions at SPS, the strong non-factorising nuclear suppression of $\mathrm {J}/\psi$ hadroproduction at high $x_F$ [858] and the large-$x_F$ production of $\mathrm {J}/\psi$ pairs [859].

A few fixed-target projects in connection with heavy-flavour and quarkonium are being discussed in our community at the SPS, Fermilab, FAIR and the LHC. These are reported below.

#### Low energy projects at SPS, Fermilab and FAIR

Using a 120 $~\text {GeV}$ proton beam extracted from the Fermilab Main Injector, the Fermilab E-906/SeaQuest experiment [860], which is part of a series of fixed target Drell–Yan experiments done at Fermilab, aims to examine the modifications to the antiquark structure of the proton from nuclear binding and to better quantify the energy loss of a coloured parton (quark) travelling through cold, strongly interacting matter. In the context of this review, one should stress that their muon spectrometer covering dimuon mass from roughly 3 to 7 $\mathrm {GeV}/c^2$ also allows one to perform $\mathrm {J}/\psi$ and $\psi \text {(2S)}$ cross section measurements with a good accuracy.

The COMPASS Collaboration has recently started to look at Drell–Yan measurements using a 190 GeV pion beam [861] with the aim of measuring single-transverse spin asymmetries and of measuring the quark Sivers functions [862]. Data taken during tests in 2009 have revealed that, with the same set-up, they can also measure, with a good accuracy, pion-induced $\mathrm {J}/\psi$ (probably also $\psi \text {(2S)}$) production cross sections at $\sqrt{s_{\mathrm{NN}}}$ = 18.8$~\text {GeV}$ on nuclear targets.

Another experiment at SPS, NA61/SHINE also has plans to move ahead to charm production in the context of heavy-ion physics. Their upgrade relies on the installation of a new silicon vertex detector [863] which would allow for precise track reconstructions and, in turn, for $\mathrm {D}^{0}$ production studies in Pb–Pb collisions at $\sqrt{s_{\mathrm{NN}}}$ = 8.6 and 17.1$~\text {GeV}$.

Finally, the FAIR project, presently under construction at GSI Darmstadt, has a nucleus–nucleus collisions programme devoted to the study of baryon-dominated matter at high densities [864]. The dedicated Compressed Baryonic Matter (CBM) experiment is designed with the initial priority on the study of open charm and charmonium close to production threshold in Au–Au collisions around 25 GeV-per-nucleon beam energy ($\sqrt{s_{\mathrm{NN}}}$  $\simeq$ 7$~\text {GeV}$). The current baseline of the FAIR project envisages collisions with Au beams only up to 10 $~\text {GeV}$ per nucleon (SIS-100), implying that the charm sector of CBM cannot be covered in nucleus–nucleus collisions. Production in proton–nucleus collisions will be studied, while the possible addition of a second ring will bring the accelerator to the initially designed energy (SIS-300).

**Table 18:** Expected yields (assuming no nuclear effects) and luminosities obtained for a 7 (2.76) TeV proton (Pb) beam extracted by means of bent crystal (upper part) and obtained with an internal gas target (lower part)

Beam	Flux (s$$^{-1}$$)	Target	$$\sqrt{s_{\mathrm{NN}}}$$ (GeV)	Thickness (cm)	$$\rho $$ (g cm$$^{-3}$$)	*A*	$$\mathcal {L}$$ ($$\mu $$b$$^{-1}$$s$$^{-1}$$)	$$\int \mathcal{{L}}$$ (pb$$^{-1}$$ y$$^{-1})$$	$$\mathrm{Br}_{\ell \ell } \left. \frac{\mathrm {d}N_{\mathrm {J}/\psi }}{\mathrm {d}y}\right| _{y=0} $$ (y$$^{-1}$$)	$$\mathrm{Br}_{\ell \ell } \left. \frac{\mathrm {d}N_{\Upsilon }}{\mathrm {d}y}\right| _{y=0} $$(y$$^{-1}$$)
p	$5 \times 10^8$	Liquid H	115	100	0.068	1	2000	20000	$4.0 \times 10^8$	$8.0 \times 10^5$
p	$5 \times 10^8$	Liquid D	115	100	0.16	2	2400	24000	$9.6 \times 10^8$	$1.9 \times 10^6$
p	$5 \times 10^8$	Pb	115	1	11.35	207	16	160	$6.7 \times 10^8$	$1.3 \times 10^6$
Pb	$2 \times 10^5$	Liquid H	72	100	0.068	1	0.8	0.8	$3.4 \times 10^6$	$6.9 \times 10^3$
Pb	$2 \times 10^5$	Liquid D	72	100	0.16	2	1	1	$8.0 \times 10^6$	$1.6 \times 10^4$
Pb	$2 \times 10^5$	Pb	72	1	11.35	207	0.007	0.007	$5.7 \times 10^6$	$1.1 \times 10^4$

Beam	Flux (s$$^{-1}$$)	Target	$$\sqrt{s_{\mathrm{NN}}}$$ (GeV)	Usable gas zone (cm)	Pressure (in units of $$10^{-9}$$ bar)	*A*	$$\mathcal {L}$$ ($$\mu $$b$$^{-1}$$s$$^{-1}$$)	$$\int \mathcal{{L}}$$ (pb$$^{-1}$$ y$$^{-1})$$	$$\mathrm{Br}_{\ell \ell } \left. \frac{\mathrm {d}N_{\mathrm {J}/\psi }}{\mathrm {d}y}\right| _{y=0} $$ (y$$^{-1}$$)	$$\mathrm{Br}_{\ell \ell } \left. \frac{\mathrm {d}N_{\Upsilon }}{\mathrm {d}y}\right| _{y=0} $$ (y$$^{-1}$$)
p	$3 \times 10^{18}$	ideal gas	115	100	*P*	*A*	10$\times P$	100$\times P$	$2 \times 10^6 \times A \times P$	$4 \times 10^3\times A \times P$
Pb	$5 \times 10^{14}$	ideal gas	72	100	*P*	*A*	0.001$\times P$	0.001$\times P$	$4.25 \times 10^3 \times A \times P$	$8.6 \times A \times P$

#### Plans for fixed-target experiments using the LHC beams

Historically, the first proposal to perform fixed-target experiments with the LHC beams dates back to the early 1990s along with the LHB proposal (see e.g. [865]) to perform flavour physics studies using the expected $10^{10}$ B mesons produced per year using an extracted beam with a flux of more than $10^8$ protons per second obtained with a bent crystal positioned in the halo of the beam. This idea was revived in the mid-2000s [866] and it is now being investigated at the LHC along with the smart collimator solution proposed by the (L)UA9 Collaboration.^30^

More generally, a beam of 7 $\text {TeV}$ protons colliding on fixed targets results in a centre-of-mass energy close to 115$~\text {GeV}$, in a range where few systems have been studied at a limited luminosity. With the 2.76 $\text {TeV}$ Pb beam, $\sqrt{s_{\mathrm{NN}}}$ amounts to 72$~\text {GeV}$, approximately half way between the top Au–Au and Cu–Cu energy at RHIC and the typical energies studied at the SPS. As discussed in Refs. [867–870], colliding the LHC proton and heavy-ion beams on fixed targets offers a remarkably wide range of physics opportunities. The fixed-target mode with TeV beams has four critical advantages: (i) very large luminosities, (ii) an easy access over the full target-rapidity domain, (iii) the target versatility and (iv) the target polarisation. This, respectively, allows for: (i) decisive statistical precision for many processes, (ii) the first experiment covering the whole negative $x_F$ domain up to $- 1$, (iii) an ambitious spin programme relying on the study of single-transverse spin asymmetries and (iv) a unique opportunity to study in detail the nuclear matter versus the hot and dense matter formed in heavy-ion collisions, including the formation of the quark–gluon plasma down to the target rapidities.

*SMOG – the first step* A first – probably decisive – step towards such a project has been made by the LHCb Collaboration using SMOG, a system designed to perform imaging of the beam profiles towards luminosity determination [871]. SMOG consists in the injection of a gas (Ne for Run 1) in the VErtex LOcator of LHCb; this also allows one to record fixed-target collisions. During test beams, data have been recorded in p–Ne collisions at $\sqrt{s_{\mathrm{NN}}}$ = 87 $~\text {GeV}$ and Pb–Ne at $\sqrt{s_{\mathrm{NN}}}$ = 54.4 $~\text {GeV}$. The current limited statistics – due to the limited gas pressure and the short run durations – has for now only allowed for strange-hadron reconstruction. A handful of $\mathrm {J}/\psi$ and charmed mesons might be extracted from these data. In any case, they have illustrated that a detector like LHCb has a very good coverage for the fixed-target mode and proved that this system can be used beyond its primary goal and offers new physics opportunities. LHCb plans in taking more data using SMOG during Run 2. The goal is to accumulate about 0.5 $\text {~nb}^{-1}$ of Pb–Ne collisions with the aim of studying $\mathrm {J}/\psi$ and $\mathrm {D}^{0}$ productions. Let us stress here that, thanks to the boost between the cms and laboratory frame, the rapidity shift between them is 4.8 with the 7 $\text {TeV}$ proton beam. Hence, a detector covering $\eta _\mathrm{lab} \in [1,5]$ allows for measurements in essentially the whole backward hemisphere, i.e. $y_\mathrm{cms}\le 0$ or $x_F \le 0$.

*A Fixed-Target ExpeRiment at the LHC, AFTER@LHC* With a dedicated set-up and run schedule (see below), $\mathrm pp$ and p–A collisions can be studied, during the $10^7$ s LHC proton run, with luminosities three orders of magnitude larger than at RHIC. Pb–A collisions can be studied, during the $10^6$ s LHC Pb run, at a luminosity comparable to that of RHIC and the LHC over the full range of the target-rapidity domain with a large variety of nuclei. Quarkonium production, open heavy-flavour hadrons and prompt photons in p–A collisions can thus be investigated [867, 868] with statistics previously unheard of (see Table 18) and in the backward region, $x_F < 0$, which is essentially uncharted. This would certainly complement the studies discussed in Sect. 3. In complement to conventional nuclear targets made of Pb, Au, W, Cu, etc., high precision QCD measurements (including some of those discussed in Sect. 2) can also obviously be performed in $\mathrm pp$ and p–d collisions with hydrogen and deuterium targets. Finally, looking at ultra-relativistic nucleus–nucleus collisions from the rest frame of one of the colliding nuclei offers the opportunity to study in detail its remnants in collisions where the QGP can be formed. Thanks to the use of the recent ultra-granular calorimetry technology, studies of direct photons, $\chi _c$ and even $\chi _b$ production in heavy-ion collisions – two measurements not available in any other experimental configuration – can be envisioned (see Ref. [872] for a similar idea at SPS energies).

To substantiate these claims, we have gathered in Table 18 a set of key quantities for two scenarios – a beam extracted/splitted with a bent crystal with a dense target and a gas target intercepting the full LHC flux –, such as the cms energy, the flux through the target, its length, its density/pressure, the instantaneous and yearly luminosities as well as the $\mathrm {J}/\psi$ and $\Upsilon$ yields close to $y=0$. Another possibility, which consists in positioning a 500 $\upmu$m thick lead ribbon in the halo of the proton or lead LHC beams, would lead to instantaneous luminosities of 100 mb$^{-1}$ s$^{-1}$ and 2.2 mb$^{-1}$ s$^{-1}$, respectively [873].

## Concluding remarks

The first Run of the LHC has provided a wealth of measurements in proton–proton and heavy-ion collisions for hadrons with open and hidden charm and beauty. The LHC data complement the rich experimental programmes at Tevatron, SPS and RHIC, extending by factors of about 4, 14 and 25 the centre-of-mass energies accessible in pp, AA and p–A collisions, respectively.

The main features of the data are in general understood. However, the current experimental precision (statistical) and accuracy (systematic uncertainties) is in most cases still limited. This, along with the lack of precise enough guidance from theoretical models, still prevents definite conclusions on production mechanisms in pp collisions (for quarkonia), their modification in p–A, and extraction of key quantities for the QGP produced in AA collisions.

In pp collisions, pQCD calculations at NLO or FONLL describe very well the open charm and beauty production cross sections within, however, rather large theoretical uncertainties, especially for charm at low $p_{\mathrm {T}}$. At the LHC, this uncertainty also impacts the scaling of the cross sections measured at top pp centre-of-mass energy to the lower Pb–Pb and p–Pb energies. Therefore, it is crucial that the future LHC programme includes adequate pp reference runs at the heavy-ion energies. In the quarkonium sector, there is a large variety of quarkonium production models. To date, none describes consistently the available measurements on production cross section and polarisation. Future data will allow one to constrain the models further and also address the question whether a single production mechanism is responsible for the low- and high-$p_{\mathrm {T}}$ quarkonia. Understanding the production process will provide insight on the quarkonium formation time, which is an important aspect for the study of medium-induced effects in p–A and A–A collisions. For both open and hidden heavy-flavour hadrons, the correlation of production with the event multiplicity is an interesting facet that may shed light on production mechanisms and the general features of proton–proton collisions at high energy. The connection of these effects with the studies in proton–nucleus and nucleus–nucleus collisions is an open and interesting field of theoretical and experimental investigation.

Initially thought of as a reference for nucleus–nucleus studies, p–A collisions provided a host of interesting results of their own. Nuclear medium effects are observed in p–A collisions on open and hidden heavy flavour at both RHIC and LHC, especially for $\mathrm {J}/\psi$ production at forward rapidity. None of the individual cold nuclear matter (CNM) effects are able to describe the data in all kinematic regions, suggesting that a mixture of different effects are at work. The approach of nuclear parton distribution functions with shadowing explains the basic features of open and hidden heavy flavour despite large uncertainties at forward rapidity and the coherent energy-loss model explains the main characteristics of quarkonium production. Theoretical interpretation of quarkonium excited states is still challenging. The impact of these CNM studies for the understanding of the nucleus–nucleus data in terms of a combination of cold and hot medium effects is yet to be fully understood. In addition, it is still an open question whether the possible signals of collective behaviour observed in high-multiplicity proton–nucleus collisions in the light-flavour sector could manifest also for heavy-flavour production. This question could become accessible with future higher-statistics proton–nucleus data samples at RHIC and LHC.

The strong electromagnetic field of lead ions circulating in the LHC is an intense source of quasi-real photons, which allows for the study of $\gamma \gamma$, $\gamma \mathrm {p}$ and $\gamma \mathrm {Pb}$ reactions at unprecedented high energies. The coherent and the incoherent photoproduction of $\mathrm {J}/\psi$ and $\psi \text {(2S)}$ is a powerful tool to study the gluon distribution in the target hadron and the first data from the LHC using Run 1 already set strong constraints to shadowing models. The statistical precision is one of the main, and in some cases the dominant, sources of uncertainty of the current measurements. The large increase in statistics expected for Run 2 and other future data-taking periods, as well as improvements in the detectors, the trigger and the data acquisition systems, will allow for a substantial reduction of the uncertainties. These future measurements will then shed a brighter light on the phenomena of shadowing and the gluon structure of dense sources, like lead ions.

The measurements of open heavy flavour production in nucleus–nucleus, proton–proton and proton–nucleus collisions at RHIC and the LHC allow us to conclude that heavy quarks experience energy loss in the hot and dense QGP. A colour charge dependence in energy loss is not clearly emerging from the data, but it is implied by the fair theoretical description of the observed patterns. A quark mass ordering is suggested by the data (some of them still preliminary, though) and the corresponding model comparisons. However, this observation is still limited to a restricted momentum and centrality domain. The important question of thermalisation of heavy quarks appears to be partly answered for charm: the positive elliptic flow observed at both RHIC and LHC indicates that charm quarks take part in the collective expansion of the QGP. This is consistent with thermalisation, but the degree of thermalisation is not yet constrained. For the beauty sector, thermalisation remains an open issue entirely. The role of the different in-medium interaction mechanisms, such as radiative, collisional energy loss and in-medium hadronisation, is still not completely clarified, although the comparison of data with theoretical models suggests the relevance of all these effects.

For the quarkonium families, the LHC data demonstrated the presence of colour screening for both charmonium and bottomonium. In the case of $\mathrm {J}/\psi$, the LHC data implies the presence of other production mechanisms, generically called (re)generation. Whether production takes place throughout the full (or most of the) lifetime of the deconfined state or rather suddenly at the confinement transition (crossover) cannot be disentangled using the existing measurements. The $\Upsilon$ production seems to exhibit a sequential pattern, but several assumed quantities in this interpretation (e.g. the feed-down contributions) make the situation not satisfactory enough.

The next steps in the study of heavy-flavour hadron production in heavy-ion collisions will lead to a stage of quantitative understanding of the data, towards the extraction of the charm and beauty quarks transport coefficients and the temperature history of the deconfined state, including the temperature of the confinement crossover. An incremental, but nevertheless important, progress is expected with the existing experimental set-ups at RHIC and the LHC (where in particular the increased collision energy enhances the relevance of the data in the next three years). The ultimate goal can only be achieved with upgraded or new detectors, which will allow for the extension of the set of observables and the precision of the measurements over a broad range of collision energies.

This experimental effort needs to be matched on the theory side. Even though the field of study of extreme deconfined matter with heavy quarks seems to be driven by experiment, the contribution of theory is of crucial importance. In particular, accurate theoretical guidance and modelling are required to interpret the measurements in terms of the QGP properties mentioned in the previous paragraph. Ultimately, the quantitative stage can only be reached in a close collaboration of experiment and theory.

## Footnotes

1 https://twiki.cern.ch/twiki/bin/view/ReteQuarkonii/SaporeGravis.

2 https://indico.cern.ch/event/247609.

3 https://indico.cern.ch/event/305164.

4 We stress here that this widespread procedure to assess theoretical uncertainties associated with pQCD computations does not provide values which should be interpreted as coming from a statistical procedure. This is only an estimate of the missing contributions at higher-order QCD corrections.

5 We, however, note that a first attempt to evaluate colour octet *decay* LDMEs was made in [79]. In principle they can be related by crossing symmetry to the *production* LDMEs which are relevant for the present discussion. Quantitative results are, however, still lacking.

6 A recent LO study has also been performed including LHC data in the used sample [83].

7 For larger $p_{\mathrm {T}}$ or *y*, such a distance can significantly be larger.

8 Let us stress that the NRQCD band in Fig. 9a is not drawn for $p_{\mathrm {T}}$ lower than 5 GeV because such a NLO NRQCD fit overshoots the data in this region and since data at low $p_{\mathrm {T}}$ are in fact not used in this fit. For a complete discussion of NLO CSM/NRQCD results for the $p_{\mathrm {T}}$-integrated yields; see [67]. As regards the CEM curves, an uncertainty band should also be drawn (see for instance [169]).

9 The expected difference between prompt and direct is discussed later on.

10 As encoded in ChiGen: https://superchic.hepforge.org/chigen.html.

11 The present ratio depends on the polarisation of the $\chi _c$ since it induces different acceptance correction.

12 For the *P* wave case, the distinction between colour-singlet and colour-octet transition is not as clear that for the *S* wave. In particular the separation between CS and CO contribution depends on the NRQCD factorisation scale $\mu _\Lambda$.

13 See [224] for the definition of the corresponding axes.

14 Irrespective of the experimental techniques used to extract it, a sample of *inclusive* low $$p_{\mathrm {T}}$$$$\psi \text {(2S)} $$ at energies around 100 $$~\text {GeV}$$ is essentially purely direct.

15 In the following we will use the generic symbol p–A to denote both p–A and d–A collisions.

16 By coherence with the use of LO hard matrix elements, one may prefer to use LO nPDFs.

17 To simplify the comparison, one simply uses the central curve of EPS09 as well as four specific extreme curves (minimal/maximal shadowing, minimal/maximal EMC effect), which reproduce the envelope of the gluon nPDF uncertainty encoded in EPS09 LO.

18 As for instance in the colour Evaporation Model. In the colour Singlet Model for $\mathrm {J}/\psi$ production, a *colour singlet*${c\overline{c}}$ pair is produced, but in conjunction with a hard gluon, thus making no qualitative difference with the production of a compact colour octet state.

19 This is the so-called “grazing expansion” [502], well justified for non-relativistic heavy quarks.

20 The Einstein relation is not necessarily satisfied for all momenta $p_Q$ in an independent calculation of *B* and $\eta _D$ and hence has to be enforced.

21 Strictly speaking, the radiative processes include some phenomenological parameter named “*X*” accounting for the LPM effect and calibrated on the $\pi$ production in central Pb–Pb collisions.

22 An alternative consists in using an effective model for quark-*Q* scattering via D-meson-like resonance excitations in the QGP based on heavy-quark effective theory (HQET) and chiral symmetry in the light-quark sector [620].

23 As open heavy flavour and quarkonia are produced via the same processes, any modifications of the initial state will not modify the yield ratio of quarkonia to open heavy flavour states.

24 References to experimental results are reported in Tables 12 and 13.

25 References to experimental results are reported in Table 14.

26 Note that in this Table the calculation of the binding energies and radii of the quarkonium states is made with an arbitrary potential model with arbitrary parameters, so they do not correspond to the experimental masses of quarkonia but are model dependent. These values are to be taken as an illustration of the expected Debye screening ordering.

27 A possible mean-field term has been neglected.

28 The integrated luminosity of the 2010 Pb–Pb run, ten times smaller than in 2011, was not sufficient to perform the measurements described below, although it was enough to measure the coherent production of $\rho ^0$ mesons [764].

29 The replacement of front-end electronics implies, for some of the LHCb detectors, like the silicon trackers, the replacement of the active elements.

30 http://home.web.cern.ch/about/experiments/ua9.

## References

[1] A. Bhattacharya, R. Enberg, M.H. Reno, I. Sarcevic, A. Stasto, Perturbative charm production and the prompt atmospheric neutrino flux in light of RHIC and LHC. arXiv:1502.01076 [hep-ph]

[2] Nason P, Dawson S, Ellis RK (1988). The total cross-section for the production of heavy quarks in hadronic collisions. Nucl. Phys. B.

[3] P. Nason, S. Dawson, R.K. Ellis, The one particle inclusive differential cross-section for heavy quark production in hadronic collisions. Nucl. Phys. B **327**, 49–92 (1989). **(Erratum-ibid. B 335, 260 (1990))**

[4] Beenakker W, Kuijf H, van Neerven WL, Smith J (1989). QCD corrections to heavy quark production in $${\text{ p }\overline{\text{ p }}}$$ collisions. Phys. Rev. D.

[5] Beenakker W, van Neerven WL, Meng R, Schuler GA, Smith J (1991). QCD corrections to heavy quark production in hadron hadron collisions. Nucl. Phys. B.

[6] Mangano ML, Nason P, Ridolfi G (1992). Heavy quark correlations in hadron collisions at next-to-leading order. Nucl. Phys. B.

[7] B. Kniehl, G. Kramer, I. Schienbein, H. Spiesberger, Inclusive B meson production at small $${p_\text{ T }}$$ in the general-mass variable-flavor-number scheme. arXiv:1502.01001 [hep-ph]

[8] Czakon M, Fiedler P, Mitov A (2013). Total top-quark pair-production cross section at hadron colliders through $${\cal O}(\alpha _s^4)$$. Phys. Rev. Lett..

[9] M. Czakon, P. Fiedler, A. Mitov, Resolving the Tevatron top quark forward–backward asymmetry puzzle. arXiv:1411.3007 [hep-ph] 10.1103/PhysRevLett.115.052001 26274412

[10] Aversa F, Chiappetta P, Greco M, Guillet JP (1989). QCD corrections to parton–parton scattering processes. Nucl. Phys. B.

[11] Cacciari M, Greco M (1994). Large $${p_\text{ T }}$$ hadroproduction of heavy quarks. Nucl. Phys. B.

[12] Binnewies J, Kniehl BA, Kramer G (1998). Predictions for $$\text{ D }^{*\pm }$$ photoproduction at HERA with new fragmentation functions from LEP1. Phys. Rev. D.

[13] Binnewies J, Kniehl BA, Kramer G (1997). Coherent description of $${\text{ D }}^{*\pm }$$ production in $${e^{+}e^{-}}$$ and low-$$Q^2$$$$\text{ ep }$$ collisions. Z. Phys. C.

[14] Binnewies J, Kniehl BA, Kramer G (1998). Inclusive B meson production in $${e^{+}e^{-}}$$ and $${\text{ p }\overline{\text{ p }}}$$ collisions. Phys. Rev. D.

[15] Kniehl BA, Kramer G, Schienbein I, Spiesberger H (2005). Inclusive $${\text{ D }}^{*\pm }$$ production in $${\text{ p }\overline{\text{ p }}}$$ collisions with massive charm quarks. Phys. Rev. D.

[16] B. Kniehl, G. Kramer, I. Schienbein, H. Spiesberger, Collinear subtractions in hadroproduction of heavy quarks. Eur. Phys. J. C **41**, 199–212 (2005). arXiv:hep-ph/0502194 [hep-ph]

[17] Aivazis MAG, Olness FI, Tung W-K (1994). Leptoproduction of heavy quarks. 1. General formalism and kinematics of charged current and neutral current production processes. Phys. Rev. D.

[18] Aivazis MAG, Collins JC, Olness FI, Tung W-K (1994). Leptoproduction of heavy quarks. 2. A unified QCD formulation of charged and neutral current processes from fixed target to collider energies. Phys. Rev. D.

[19] Krämer M, Olness FI, Soper DE (2000). Treatment of heavy quarks in deeply inelastic scattering. Phys. Rev. D.

[20] Tung W-K, Kretzer S, Schmidt C (2002). Open heavy flavor production in QCD: conceptual framework and implementation issues. J. Phys..

[21] Kretzer S, Schienbein I (1998). Heavy quark initiated contributions to deep inelastic structure functions. Phys. Rev. D.

[22] Stavreva T, Olness F, Schienbein I, Jezo T, Kusina A (2012). Heavy quark production in the ACOT scheme at NNLO and N3LO. Phys. Rev. D.

[23] Guzzi M, Nadolsky PM, Lai H-L, Yuan C-P (2012). General-mass treatment for deep inelastic scattering at two-loop accuracy. Phys. Rev. D.

[24] Collins JC (1998). Hard-scattering factorization with heavy quarks: a general treatment. Phys. Rev. D.

[25] R. Thorne, W. Tung, PQCD formulations with heavy quark masses and global analysis. arXiv:0809.0714 [hep-ph]

[26] Olness F, Schienbein I (2009). Heavy quarks: lessons learned from HERA and Tevatron. Nucl. Phys. Proc. Suppl..

[27] S. Kretzer, I. Schienbein, Charged current leptoproduction of $${\text{ D }}$$ mesons in the variable flavor scheme. Phys. Rev. D **56**, 1804–1807 (1997). arXiv:hep-ph/9702296 [hep-ph]

[28] S. Kretzer, I. Schienbein, Heavy quark fragmentation in deep inelastic scattering. Phys. Rev. D **59**, 054004 (1999). arXiv:hep-ph/9808375 [hep-ph]

[29] F.I. Olness, R. Scalise, W.-K. Tung, Heavy quark hadroproduction in perturbative QCD. Phys. Rev. D **59**, 014506 (1999). arXiv:hep-ph/9712494 [hep-ph]

[30] G. Kramer, H. Spiesberger, Inclusive $${\text{ D }}^{*}$$ collisions at next-to-leading order QCD. Eur. Phys. J. C **22**, 289–301 (2001). arXiv:hep-ph/0109167 [hep-ph]

[31] G. Kramer, H. Spiesberger, Inclusive $${\text{ D }}^{*}$$ collisions: including the single resolved contribution with massive quarks. Eur. Phys. J. C **28**, 495–513 (2003). arXiv:hep-ph/0302081 [hep-ph]

[32] G. Kramer, H. Spiesberger, Inclusive photoproduction of $${\text{ D }}^{*}$$ mesons with massive charm quarks. Eur. Phys. J. C **38**, 309–318 (2004). arXiv:hep-ph/0311062 [hep-ph]

[33] B. Kniehl, G. Kramer, I. Schienbein, H. Spiesberger, Reconciling open charm production at the Fermilab Tevatron with QCD. Phys. Rev. Lett. **96**, 012001 (2006). arXiv:hep-ph/0508129 [hep-ph] 10.1103/PhysRevLett.96.012001 16486440

[34] Kneesch T, Kniehl B, Kramer G, Schienbein I (2008). Charmed-meson fragmentation functions with finite-mass corrections. Nucl. Phys. B.

[35] Kniehl BA, Kramer G, Schienbein I, Spiesberger H (2008). Finite-mass effects on inclusive B meson hadroproduction. Phys. Rev. D.

[36] Kniehl BA, Kramer G, Schienbein I, Spiesberger H (2009). Inclusive photoproduction of $$\text{ D }^{*\pm }$$ mesons at next-to-leading order in the general-mass variable-flavor-number scheme. Eur. Phys. J. C.

[37] Kniehl B, Kramer G, Schienbein I, Spiesberger H (2011). Inclusive B-meson production at the LHC in the GM-VFN scheme. Phys. Rev. D.

[38] Kniehl B, Kramer G, Schienbein I, Spiesberger H (2012). Inclusive charmed-meson production at the CERN LHC. Eur. Phys. J. C.

[39] Bolzoni P, Kramer G (2013). Inclusive lepton production from heavy-hadron decay in $${\text{ pp }}$$ collisions at the LHC. Nucl. Phys. B.

[40] Bolzoni P, Kramer G (2014). Inclusive charmed-meson production from bottom hadron decays at the LHC. J. Phys..

[41] M. Cacciari, M. Greco, P. Nason, The $$P(T)$$ spectrum in heavy flavor hadroproduction. JHEP **9805**, 007 (1998). arXiv:hep-ph/9803400 [hep-ph]

[42] S. Forte, L. Garrido, J.I. Latorre, A. Piccione, Neural network parametrization of deep inelastic structure functions. JHEP **0205**, 062 (2002). arXiv:hep-ph/0204232 [hep-ph]

[43] Ball RD, Bertone V, Cerutti F, Del Debbio L, Forte S (2011). Impact of heavy quark masses on parton distributions and LHC phenomenology. Nucl. Phys. B.

[44] Cacciari M, Frixione S, Houdeau N, Mangano ML, Nason P (2012). Theoretical predictions for charm and bottom production at the LHC. JHEP.

[45] Sjostrand T, Mrenna S, Skands PZ, Brief A (2008). Introduction to PYTHIA 8.1. Comput. Phys. Commun..

[46] G. Corcella, I. Knowles, G. Marchesini, S. Moretti, K. Odagiri et al., HERWIG 6: an event generator for hadron emission reactions with interfering gluons (including supersymmetric processes). JHEP **0101**, 010 (2001). arXiv:hep-ph/0011363 [hep-ph]

[47] Frixione S, Nason P, Webber BR (2003). Matching NLO QCD and parton showers in heavy flavour production. JHEP.

[48] Frixione S, Nason P, Ridolfi G (2007). A positive-weight next-to-leading-order Monte Carlo for heavy flavour hadroproduction. JHEP.

[49] Klasen M, Klein-Bösing C, Kovarik K, Kramer G, Topp M (2014). NLO Monte Carlo predictions for heavy-quark production at the LHC: $${\text{ pp }}$$ collisions in ALICE. JHEP.

[50] Fritzsch H (1977). Producing heavy quark flavors in hadronic collisions: a test of quantum chromodynamics. Phys. Lett. B.

[51] Halzen F (1977). CVC for gluons and hadroproduction of quark flavors. Phys. Lett. B.

[52] J. Amundson, O.J. Eboli, E. Gregores, F. Halzen, Colorless states in perturbative QCD: charmonium and rapidity gaps. Phys. Lett. B **372**, 127–132 (1996). arXiv:hep-ph/9512248 [hep-ph]

[53] M. Bedjidian, D. Blaschke, G.T. Bodwin, N. Carrer, B. Cole et al., Hard probes in heavy ion collisions at the LHC: heavy flavor physics. arXiv:hep-ph/0311048 [hep-ph]

[54] Chang C-H (1980). Hadronic production of $${\text{ J }}/\psi $$ associated with a gluon. Nucl. Phys. B.

[55] Baier R, Ruckl R (1981). Hadronic production of $${\text{ J }}/\psi $$ and $${\Upsilon }$$: transverse momentum distributions. Phys. Lett. B.

[56] Baier R, Ruckl R (1983). Hadronic collisions: a quarkonium factory. Z. Phys. C.

[57] J.M. Campbell, F. Maltoni, F. Tramontano, QCD corrections to $$J/\psi $$ and $$\Upsilon $$ production at hadron colliders. Phys. Rev. Lett. **98**, 252002 (2007). arXiv:hep-ph/0703113 [HEP-PH] 10.1103/PhysRevLett.98.252002 17678016

[58] P. Artoisenet, J. Lansberg, F. Maltoni, Hadroproduction of $${\text{ J }}/\psi $$ in association with a heavy-quark pair. Phys. Lett. B **653**, 60–66 (2007). arXiv:hep-ph/0703129 [HEP-PH]

[59] Gong B, Wang J-X (2008). Next-to-leading-order QCD corrections to $${\text{ J }}/\psi $$ polarization at Tevatron and large-hadron-collider energies. Phys. Rev. Lett..

[60] Gong B, Wang J-X (2008). QCD corrections to polarization of $${\text{ J }}/\psi $$ and $${\Upsilon }$$ at Tevatron and LHC. Phys. Rev. D.

[61] Artoisenet P, Campbell JM, Lansberg J, Maltoni F, Tramontano F (2008). $${\Upsilon }$$ production at fermilab Tevatron and LHC energies. Phys. Rev. Lett..

[62] Lansberg J (2009). On the mechanisms of heavy-quarkonium hadroproduction. Eur. Phys. J. C.

[63] Conesa del Valle Z, Corcella G, Fleuret F, Ferreiro E, Kartvelishvili V (2011). Quarkonium production in high energy proton–proton and proton–nucleus collisions. Nucl. Phys. Proc. Suppl..

[64] Brambilla N, Eidelman S, Heltsley B, Vogt R, Bodwin G (2011). Heavy quarkonium: progress, puzzles, and opportunities. Eur. Phys. J. C.

[65] Brodsky SJ, Lansberg J-P (2010). Heavy-quarkonium production in high energy proton–proton collisions at RHIC. Phys. Rev. D.

[66] J. Lansberg, Total $${\text{ J }}/\psi $$ production cross section at the LHC: theory vs. experiment. PoS ICHEP **2010**, 206 (2010). arXiv:1012.2815 [hep-ph]

[67] Feng Y, Lansberg J-P, Wang J-X (2015). Energy dependence of direct-quarkonium production in $${\text{ pp }}$$ collisions from fixed-target to LHC energies: complete one-loop analysis. Eur. Phys. J. C.

[68] Ma Y-Q, Zhang Y-J, Chao K-T (2009). QCD correction to $$e^{+} e^{-} \longrightarrow {\text{ J }}/\psi g g$$ at B factories. Phys. Rev. Lett..

[69] Gong B, Wang J-X (2009). Next-to-leading-order QCD corrections to $${e^{+}e^{-}} \longrightarrow {\text{ J }}/\psi g g$$ at the B factories. Phys. Rev. Lett..

[70] He Z-G, Fan Y, Chao K-T (2010). Relativistic correction to $$e^{+} e^{-} {\text{ J }}/\psi + gg$$ at B factories and constraint on color-octet matrix elements. Phys. Rev. D.

[71] H1 Collaboration, F. Aaron et al., Inelastic production of $${\text{ J }}/\psi $$ mesons in photoproduction and deep inelastic scattering at HERA. Eur. Phys. J. C **68**, 401–420 (2010). arXiv:1002.0234 [hep-ex]

[72] Lansberg J (2011). QCD corrections to $${\text{ J }}/\psi $$ polarisation in $${\text{ pp }}$$ collisions at RHIC. Phys. Lett. B.

[73] Lansberg J (2011). $$J/\psi $$ production at $${\sqrt{s}}$$ = 1.96 and 7 TeV: color-singlet model, NNLO* and polarisation. J. Phys..

[74] Lansberg J (2013). $$\Upsilon $$ production in $${\text{ pp }}$$ and p-A collisions: from RHIC to the LHC. Nucl. Phys..

[75] J. Lansberg, $$\Psi (2S)$$ production in proton–proton collisions at RHIC, Tevatron and LHC energies. PoS ICHEP **2012**, 293 (2013). arXiv:1303.2858 [hep-ph]

[76] Barbieri R, Gatto R, Remiddi E (1976). Singular binding dependence in the hadronic widths of $$1++$$ and $$1+-$$ heavy quark anti-quark bound states. Phys. Lett. B.

[77] G.T. Bodwin, E. Braaten, G.P. Lepage, Rigorous QCD predictions for decays of P wave quarkonia. Phys. Rev. D **46**, 1914–1918 (1992). arXiv:hep-lat/9205006 [hep-lat] 10.1103/physrevd.46.r1914 10015156

[78] G.T. Bodwin, E. Braaten, G.P. Lepage, Rigorous QCD analysis of inclusive annihilation and production of heavy quarkonium. Phys. Rev. D **51**, 1125–1171 (1995). arXiv:hep-ph/9407339 [hep-ph]. **(Erratum: Phys. Rev. D 55,5853(1997))** 10.1103/physrevd.51.1125 10018572

[79] G.T. Bodwin, J. Lee, D.K. Sinclair, Spin correlations and velocity-scaling in color-octet NRQCD matrix elements. Phys. Rev. D **72**, 014009 (2005). arXiv:hep-lat/0503032 [hep-lat]

[80] Butenschoen M, Kniehl BA (2012). $${\text{ J }}/\psi $$ polarization at Tevatron and LHC: nonrelativistic-QCD factorization at the crossroads. Phys. Rev. Lett..

[81] Gong B, Wan L-P, Wang J-X, Zhang H-F (2013). Polarization for prompt $${\text{ J }}/\psi $$, $${\psi \text{(2S) }}$$ production at the Tevatron and LHC. Phys. Rev. Lett..

[82] Chao K-T, Ma Y-Q, Shao H-S, Wang K, Zhang Y-J (2012). $$J/\psi $$ polarization at hadron colliders in nonrelativistic QCD. Phys. Rev. Lett..

[83] Sharma R, Vitev I (2013). High transverse momentum quarkonium production and dissociation in heavy ion collisions. Phys. Rev. C.

[84] G.C. Nayak, J.-W. Qiu, G.F. Sterman, Fragmentation, NRQCD and NNLO factorization analysis in heavy quarkonium production. Phys. Rev. D **72**, 114012 (2005). arXiv:hep-ph/0509021 [hep-ph]

[85] G.C. Nayak, J.-W. Qiu, G.F. Sterman, Fragmentation, factorization and infrared poles in heavy quarkonium production. Phys. Lett. B **613**, 45–51 (2005). arXiv:hep-ph/0501235 [hep-ph]

[86] Kang Z-B, Qiu J-W, Sterman G (2012). Heavy quarkonium production and polarization. Phys. Rev. Lett..

[87] Ma Y-Q, Qiu J-W, Sterman G, Zhang H (2014). Factorized power expansion for high-$$p_T$$ heavy quarkonium production. Phys. Rev. Lett..

[88] Fleming S, Leibovich AK, Mehen T, Rothstein IZ (2012). The systematics of quarkonium production at the LHC and double parton fragmentation. Phys. Rev. D.

[89] P. Hagler, R. Kirschner, A. Schafer, L. Szymanowski, O. Teryaev, Towards a solution of the charmonium production controversy: $$k^{-}$$ perpendicular factorization versus color octet mechanism. Phys. Rev. Lett. **86**, 1446–1449 (2001). arXiv:hep-ph/0004263 [hep-ph] 10.1103/PhysRevLett.86.1446 11290164

[90] F. Yuan, K.-T. Chao, Polarizations of $$J/\psi $$ factorization approach. Phys. Rev. Lett. **87**, 022002 (2001). arXiv:hep-ph/0009224 [hep-ph]

[91] Boer D, Pisano C (2012). Polarized gluon studies with charmonium and bottomonium at LHCb and AFTER. Phys. Rev. D.

[92] Ma J, Wang J, Zhao S (2013). Transverse momentum dependent factorization for quarkonium production at low transverse momentum. Phys. Rev. D.

[93] Kang Z-B, Ma Y-Q, Venugopalan R (2014). Quarkonium production in high energy proton–nucleus collisions: CGC meets NRQCD. JHEP.

[94] Ma Y-Q, Venugopalan R (2014). Comprehensive description of $${\text{ J }}/\psi $$ production in proton–proton collisions at collider energies. Phys. Rev. Lett..

[95] Nayak GC, Qiu J-W, Sterman GF (2007). Color transfer in associated heavy-quarkonium production. Phys. Rev. Lett..

[96] CMS Collaboration, S. Chatrchyan et al., Identification of b-quark jets with the CMS experiment. JINST **8**, P04013 (2013). arXiv:1211.4462 [hep-ex]

[97] NA38, NA50s Collaboration, M. Abreu et al., Dimuon and charm production in nucleus-nucleus collisions at the CERN SPS. Eur. Phys. J. C **14**, 443–455 (2000)

[98] LHCb Collaboration, R. Aaij et al., Measurement of the $${\eta _c} (1S)$$ production cross-section in proton–proton collisions via the decay $${\eta _c} (1S)\rightarrow p\bar{p}$$ (2014). arXiv:1409.3612 [hep-ex]

[99] M. Cacciari, S. Frixione, M. Mangano, P. Nason, G. Ridolfi, QCD analysis of first $$b$$ cross-section data at 1.96 TeV. JHEP **0407**, 033 (2004). arXiv:hep-ph/0312132 [hep-ph]

[100] CLEO Collaboration, D. Bortoletto et al., Charm production in nonresonant $${e^{+}e^{-}}$$ annihilations at $${\sqrt{s}}$$ = 10.55 GeV. Phys. Rev. D **37**, 1719 (1988) 10.1103/physrevd.37.1719 9958864

[101] ALEPH Collaboration, R. Barate et al., Study of charm production in Z decays. Eur. Phys. J. C **16**, 597–611 (2000). arXiv:hep-ex/9909032 [hep-ex]

[102] CDF Collaboration, D. Acosta et al., Measurement of prompt charm meson production cross sections in $${\text{ p }\overline{\text{ p }}}$$ = 1.96 TeV. Phys. Rev. Lett. **91**, 241804 (2003). arXiv:hep-ex/0307080 [hep-ex] 10.1103/PhysRevLett.91.241804 14683110

[103] STAR Collaboration, L. Adamczyk et al., Measurements of $$\text{ D }^{0}$$ = 200 GeV. Phys. Rev. D **86**, 072013 (2012). arXiv:1204.4244 [nucl-ex]

[104] ALICE Collaboration, B. Abelev et al., Measurement of charm production at central rapidity in proton–proton collisions at $$\sqrt{s}=2.76$$ TeV. JHEP **1207**, 191 (2012). arXiv:1205.4007 [hep-ex]

[105] LHCb Collaboration, Prompt charm production in $${{\text{ pp }}}$$ = 7 TeV, LHCb-CONF-2010-013 (2010)

[106] LHCb Collaboration, R. Aaij et al., Prompt charm production in pp collisions at $${\sqrt{s}}$$ = 7 TeV. Nucl. Phys. B **871**, 1–20 (2013). arXiv:1302.2864 [hep-ex]

[107] ALICE Collaboration, B.B. Abelev et al., Beauty production in pp collisions at $$\sqrt{s}$$ = 2.76 TeV measured via semi-electronic decays. Phys. Lett. B **738**, 97–108 (2014). arXiv:1405.4144 [nucl-ex]

[108] ALICE Collaboration, B. Abelev et al., Measurement of prompt $$J/\psi $$ TeV. JHEP **1211**, 065 (2012). arXiv:1205.5880 [hep-ex]

[109] UA1 Collaboration, C. Albajar et al., Beauty production at the CERN p anti-p collider. Phys. Lett. B **256**, 121–128 (1991)

[110] CDF Collaboration, F. Abe et al., Measurement of the B meson differential cross-section, $${\text{ d }} \sigma / {\text{ d }} p_T$$ TeV. Phys. Rev. Lett. **75**, 1451–1455 (1995). arXiv:hep-ex/9503013 [hep-ex] 10.1103/PhysRevLett.75.1451 10060302

[111] PHENIX Collaboration, A. Adare et al., Measurement of bottom versus charm as a function of transverse momentum with electron–hadron correlations in $$p^{+} p$$ GeV. Phys. Rev. Lett. **103**, 082002 (2009). arXiv:0903.4851 [hep-ex] 10.1103/PhysRevLett.103.082002 19792719

[112] PHENIX Collaboration, A. Adare et al., Measurement of high-p(T) single electrons from heavy-flavor decays in p+p collisions at s**(1/2) = 200-GeV. Phys. Rev. Lett. **97**, 252002 (2006). arXiv:hep-ex/0609010 [hep-ex] 10.1103/PhysRevLett.97.252002 17280343

[113] PHENIX exp RHIC-BNL-PHENIX Collaboration, A. Adare et al., Azimuthal correlations of electrons from heavy-flavor decay with hadrons in p+p and Au+Au collisions at $$\sqrt{s_{NN}}=200$$ GeV. Phys. Rev. C **83**, 044912 (2011). arXiv:1011.1477 [nucl-ex]

[114] PHENIX Collaboration, A. Adare et al., Heavy-flavor electron–muon correlation in p+p and d+Au collisions at $$\sqrt{s_{{\text{ NN }}}}$$ = 200 GeV. Phys. Rev. C **89**, 034915 (2014). arXiv:1311.1427 [nucl-ex]

[115] STAR Collaboration, M. Aggarwal et al., Measurement of the bottom contribution to non-photonic electron production in p+p collisions at $$\sqrt{s} $$ = 200 GeV. Phys. Rev. Lett. **105**, 202301 (2010). arXiv:1007.1200 [nucl-ex] 10.1103/PhysRevLett.105.202301 21231222

[116] STAR Collaboration, H. Agakishiev et al., High $$p_{T}$$ GeV. Phys. Rev. D **83**, 052006 (2011). arXiv:1102.2611 [nucl-ex]

[117] ALICE Collaboration, B. Abelev et al., Measurement of electrons from semileptonic heavy-flavour hadron decays in pp collisions at $$\sqrt{s}$$ = 7 TeV. Phys. Rev. D **86**, 112007 (2012). arXiv:1205.5423 [hep-ex]

[118] ALICE Collaboration, B.B. Abelev et al., Measurement of electrons from semileptonic heavy-flavor hadron decays in pp collisions at $$\sqrt{s} = 2.76$$ TeV. Phys. Rev. D **91**(1), 012001 (2015). arXiv:1405.4117 [nucl-ex]

[119] ALICE Collaboration, B. Abelev et al., Heavy flavour decay muon production at forward rapidity in proton–proton collisions at $$\sqrt{s} = 7$$ TeV. Phys. Lett. B **708**, 265–275 (2012). arXiv:1201.3791 [hep-ex]

[120] ALICE Collaboration, B. Abelev et al., Production of muons from heavy flavour decays at forward rapidity in pp and Pb–Pb collisions at $$\sqrt{s_{NN}}$$ = 2.76 TeV. Phys. Rev. Lett. **109**, 112301 (2012). arXiv:1205.6443 [hep-ex] 10.1103/PhysRevLett.109.112301 23005621

[121] ATLAS Collaboration, G. Aad et al., Measurements of the electron and muon inclusive cross-sections in proton–proton collisions at $$\sqrt{s}=7$$ TeV with the ATLAS detector. Phys. Lett. B **707** , 438–458 (2012). arXiv:1109.0525 [hep-ex]

[122] Maciula R, Szczurek A (2013). Open charm production at the LHC—$$k_{t}$$-factorization approach. Phys. Rev. D.

[123] ALICE Collaboration, B. Abelev et al., Measurement of electrons from beauty hadron decays in pp collisions at $$\sqrt{s}=7$$ TeV. Phys. Lett. B **721**, 13–23 (2013). arXiv:1208.1902 [hep-ex]

[124] CDF Collaboration, B. Reisert, Charm production studies at CDF. Nucl. Phys. Proc. Suppl. **170**, 243–247 (2007)

[125] ALICE Collaboration, B. Abelev et al., Measurement of charm production at central rapidity in proton-proton collisions at $$\sqrt{s} = 7$$ TeV. JHEP **1201**, 128 (2012). arXiv:1111.1553 [hep-ex]

[126] ALICE Collaboration, B. Abelev et al., $${\text{ D }}^{+}_{s}$$ TeV. Phys. Lett. B **718**, 279–294 (2012). arXiv:1208.1948 [hep-ex]

[127] CDF Collaboration, T. Aaltonen et al., Measurements of the properties of $$\Lambda _c(2595)$$ baryons. Phys. Rev. D **84**, 012003 (2011). arXiv:1105.5995 [hep-ex]

[128] E791 Collaboration, E. Aitala et al., Mass splitting and production of sigma(c)0 and sigma(c)++ measured in 500 GeV pi-n interactions. Phys. Lett. B **379**, 292–298 (1996). arXiv:hep-ex/9604007 [hep-ex]

[129] FOCUS Collaboration, J. Link et al., Measurement of natural widths of Sigma0(c) and Sigma++(c) baryons. Phys. Lett. B **525**, 205–210 (2002). arXiv:hep-ex/0111027 [hep-ex]

[130] CLEO Collaboration, M. Artuso et al., Measurement of the masses and widths of the Sigma++(c) and Sigma0(c) charmed baryons. Phys. Rev. D **65**, 071101 (2002). arXiv:hep-ex/0110071 [hep-ex]

[131] Particle Data Group Collaboration, K. Olive et al., Review of particle physics. Chin. Phys. C **38**, 090001 (2014)

[132] Briere RA (2006). The renaissance of charm physics. AIP Conf. Proc..

[133] Klempt E, Richard J-M (2010). Baryon spectroscopy. Rev. Mod. Phys..

[134] Quark Flavor Physics Working Group Collaboration, J. Butler et al., Working group report: quark flavor physics. arXiv:1311.1076 [hep-ex]

[135] BaBar, Belle Collaboration, A. Bevan et al., The physics of the B factories. Eur. Phys. J. C **74**(11), 3026 (2014). arXiv:1406.6311 [hep-ex]

[136] ATLAS Collaboration, G. Aad et al., Measurement of the production cross-section of $$\psi (2S) \rightarrow J/\psi ( \rightarrow \mu ^{+} \mu ^{-}) \pi ^{+} \pi ^{-}$$ = 7 TeV at ATLAS. JHEP **1409**, 79 (2014). arXiv:1407.5532 [hep-ex]

[137] ATLAS Collaboration, G. Aad et al., Measurement of $$\chi _{c1}$$ collisions at ATLAS. JHEP **1407**, 154 (2014). arXiv:1404.7035 [hep-ex]

[138] M.L. Mangano, Two lectures on heavy quark production in hadronic collisions (1997). arXiv:hep-ph/9711337 [hep-ph]

[139] CMS Collaboration, V. Khachatryan et al., Measurement of the $$\text{ B }^{+}$$ TeV. Phys. Rev. Lett. **106**, 112001 (2011). arXiv:1101.0131 [hep-ex] 10.1103/PhysRevLett.106.112001 21469857

[140] ATLAS Collaboration, G. Aad et al., Measurement of the differential cross-section of $$\text{ B }^{+}$$ = 7 TeV at ATLAS. JHEP **1310**, 042 (2013). arXiv:1307.0126 [hep-ex]

[141] CMS Collaboration, S. Chatrchyan et al., Measurement of the $$\Lambda $$ TeV. Phys. Lett. B **714**, 136–157 (2012). arXiv:1205.0594 [hep-ex]

[142] CDF Collaboration, T. Aaltonen et al., Evidence for a narrow near-threshold structure in the $$J/\psi \phi $$ Decays. Phys. Rev. Lett. **102**, 242002 (2009). arXiv:0903.2229 [hep-ex] 10.1103/PhysRevLett.102.242002 19658999

[143] D0 Collaboration, V.M. Abazov et al., Search for the $$X(4140)$$ decays with the D0 detector. Phys. Rev. D **89**(1), 012004 (2014). arXiv:1309.6580 [hep-ex]

[144] LHCb Collaboration, R. Aaij et al., Measurement of b-hadron masses. Phys. Lett. B **708**, 241–248 (2012). arXiv:1112.4896 [hep-ex]

[145] LHCb Collaboration, R. Aaij et al., Measurement of B meson production cross-sections in proton–proton collisions at $$\sqrt{s}$$ = 7 TeV. JHEP **1308**, 117 (2013). arXiv:1306.3663

[146] LHCb Collaboration, R. Aaij et al., Measurement of the $${{\text{ B }}^{\pm }}$$ TeV. JHEP **1204**, 093 (2012). arXiv:1202.4812 [hep-ex]

[147] CMS Collaboration, S. Chatrchyan et al., Measurement of the $$\text{ B }^{0}$$ TeV. Phys. Rev. Lett. **106**, 252001 (2011). arXiv:1104.2892 [hep-ex] 10.1103/PhysRevLett.106.252001 21770632

[148] CMS Collaboration, S. Chatrchyan et al., Measurement of the strange B meson production cross section with J/Psi $$\phi $$ TeV. Phys. Rev. D **84**, 052008 (2011). arXiv:1106.4048 [hep-ex]

[149] CMS Collaboration, S. Chatrchyan et al., Inclusive b-jet production in pp collisions at $$\sqrt{s}=7$$ TeV. JHEP **1204**, 084 (2012). arXiv:1202.4617 [hep-ex]

[150] ATLAS Collaboration, G. Aad et al., Measurement of the inclusive and dijet cross-sections of b-jets in pp collisions at $$\sqrt{s}=7$$ TeV with the ATLAS detector. Eur. Phys. J. C **71**, 1846 (2011). arXiv:1109.6833 [hep-ex]

[151] T. Sjostrand, S. Mrenna, P.Z. Skands, PYTHIA 6.4 physics and manual. JHEP **0605**, 026 (2006). arXiv:hep-ph/0603175 [hep-ph]

[152] Alioli S, Nason P, Oleari C, Re E (2010). A general framework for implementing NLO calculations in shower Monte Carlo programs: the POWHEG BOX. JHEP.

[153] Frixione S, Webber BR (2002). Matching NLO QCD computations and parton shower simulations. JHEP.

[154] CDF Collaboration, T.A. Aaltonen et al., Mass and lifetime measurements of bottom and charm baryons in $${{\text{ p }}\overline{{\text{ p }}}}$$ TeV. Phys. Rev. D **89**(7), 072014 (2014). arXiv:1403.8126 [hep-ex]

[155] D0 Collaboration, V. Abazov et al., Observation of the doubly strange b baryon $$\Omega _b^{-}$$. Phys. Rev. Lett. **101**, 232002 (2008). arXiv:0808.4142 [hep-ex] 10.1103/PhysRevLett.101.232002 19113541

[156] CDF Collaboration, T. Aaltonen et al., Observation of the $$\Omega _b^{-}$$. Phys. Rev. D **80**, 072003 (2009). arXiv:0905.3123 [hep-ex]

[157] CMS Collaboration, S. Chatrchyan et al., Observation of a new Xi(b) baryon. Phys. Rev. Lett. **108**, 252002 (2012). arXiv:1204.5955 [hep-ex] 10.1103/PhysRevLett.108.252002 23004588

[158] LHCb Collaboration, R. Aaij et al., Observation of two new $$\Xi _b^{-}$$ baryon resonances. arXiv:1411.4849 [hep-ex]

[159] D. Ebert, R. Faustov, V. Galkin, Masses of heavy baryons in the relativistic quark model. Phys. Rev. D **72**, 034026 (2005). arXiv:hep-ph/0504112 [hep-ph]

[160] Liu X, Chen H-X, Liu Y-R, Hosaka A, Zhu S-L (2008). Bottom baryons. Phys. Rev. D.

[161] Jenkins EE (2008). Model-independent bottom baryon mass predictions in the 1/N($$c$$) expansion. Phys. Rev. D.

[162] Karliner M, Keren-Zur B, Lipkin HJ, Rosner JL (2009). The quark model and $$b$$ baryons. Ann. Phys..

[163] Lewis R, Woloshyn R (2009). Bottom baryons from a dynamical lattice QCD simulation. Phys. Rev. D.

[164] M. Karliner, Heavy quark spectroscopy and prediction of bottom baryon masses. Nucl. Phys. Proc. Suppl. **187**, 21–28 (2009). arXiv:0806.4951 [hep-ph]

[165] Zhang J-R, Huang M-Q (2008). Heavy baryon spectroscopy in QCD. Phys. Rev. D.

[166] Detmold W, Lin CD, Meinel S (2012). Calculation of the heavy-hadron axial couplings $$g_1$$, $$g_2$$, and $$g_3$$ using lattice QCD. Phys. Rev. D.

[167] DELPHI Collaboration, J. Abdallah et al., A study of the b-quark fragmentation function with the DELPHI detector at LEP I and an averaged distribution obtained at the Z Pole. Eur. Phys. J. C **71**, 1557 (2011). arXiv:1102.4748 [hep-ex]

[168] P. Nason, A new method for combining NLO QCD with shower Monte Carlo algorithms. JHEP **0411**, 040 (2004). arXiv:hep-ph/0409146 [hep-ph]

[169] Nelson R, Vogt R, Frawley A (2013). Narrowing the uncertainty on the total charm cross section and its effect on the $$J/\psi $$ cross section. Phys. Rev. C.

[170] ATLAS Collaboration, G. Aad et al., Measurement of the differential cross-sections of inclusive, prompt and non-prompt $$J/\psi $$ TeV. Nucl. Phys. B **850**, 387–444 (2011). arXiv:1104.3038 [hep-ex]

[171] CMS Collaboration, S. Chatrchyan et al., $$J/\psi $$ TeV. JHEP **1202**, 011 (2012). arXiv:1111.1557 [hep-ex]

[172] LHCb Collaboration, R. Aaij et al., Measurement of $$J/\psi $$. Eur. Phys. J. C **71**, 1645 (2011). arXiv:1103.0423 [hep-ex]

[173] Ma Y-Q, Wang K, Chao K-T (2011). $$J/\psi (\psi ^\prime )$$ production at the Tevatron and LHC at $${\cal O}(\alpha _s^4v^4)$$ in nonrelativistic QCD. Phys. Rev. Lett..

[174] A.D. Frawley, T. Ullrich, R. Vogt, Heavy flavor in heavy-ion collisions at RHIC and RHIC II. Phys. Rept. **462**, 125–175 (2008). arXiv:0806.1013 [nucl-ex]

[175] CDF Collaboration, D. Acosta et al., Observation of the narrow state $$X(3872) \rightarrow J/\psi \pi ^{+} \pi ^{-}$$ TeV. Phys. Rev. Lett. **93**, 072001 (2004). arXiv:hep-ex/0312021 [hep-ex] 10.1103/PhysRevLett.93.072001 15324226

[176] CDF Collaboration, G. Bauer, The $$X(3872) $$ at CDF II. Int. J. Mod. Phys. A **20**, 3765–3767 (2005). arXiv:hep-ex/0409052 [hep-ex]

[177] CMS Collaboration, S. Chatrchyan et al., Measurement of the X(3872) production cross section via decays to $${\text{ J }}/\psi $$ = 7 TeV. JHEP **1304**, 154 (2013). arXiv:1302.3968 [hep-ex]

[178] LHCb Collaboration, R. Aaij et al., Observation of $$X(3872) $$ TeV. Eur. Phys. J. C **72**, 1972 (2012). arXiv:1112.5310 [hep-ex]

[179] Butenschoen M, He Z-G, Kniehl BA (2013). NLO NRQCD disfavors the interpretation of X(3872) as $$\chi _{c1}(2P)$$. Phys. Rev. D.

[180] Han H, Ma Y-Q, Meng C, Shao H-S, Chao K-T (2015). $${\eta _c}$$ production at LHC and indications on the understanding of $${\text{ J }}/\psi $$ production. Phys. Rev. Lett..

[181] D0 Collaboration, V. Abazov et al., Observation and properties of the $$X(3872)$$ TeV. Phys. Rev. Lett. **93**, 162002 (2004). arXiv:hep-ex/0405004 [hep-ex] 10.1103/PhysRevLett.93.162002 15524981

[182] LHCb Collaboration, R. Aaij et al., Determination of the X(3872) meson quantum numbers. Phys. Rev. Lett. **110**, 222001 (2013). arXiv:1302.6269 [hep-ex] 10.1103/PhysRevLett.110.222001 23767712

[183] Butenschoen M, He Z-G, Kniehl BA (2015). $${\eta _c}$$ production at the LHC challenges nonrelativistic-QCD factorization. Phys. Rev. Lett..

[184] Zhang H-F, Sun Z, Sang W-L, Li R (2015). Impact of $${\eta _c}$$ hadroproduction data on charmonium production and polarization within NRQCD framework. Phys. Rev. Lett..

[185] LHCb Collaboration, R. Aaij et al., Measurement of the relative rate of prompt $$\chi _{c0}$$ TeV. JHEP **1310**, 115 (2013). arXiv:1307.4285 [hep-ex]

[186] CDF Collaboration, F. Abe et al., Production of $$J/\psi $$ mesons from $$\chi _c$$ meson decays in $${\text{ p }\overline{\text{ p }}}$$ collisions at $$\sqrt{s} = 1.8$$ TeV. Phys. Rev. Lett. **79**, 578–583 (1997)

[187] M. Kramer, Quarkonium production at high-energy colliders. Prog. Part. Nucl. Phys. **47**, 141–201 (2001). arXiv:hep-ph/0106120 [hep-ph]

[188] J. Lansberg, $$J/\psi $$ production at hadron colliders: a review. Int. J. Mod. Phys. A **21**, 3857–3916 (2006). arXiv:hep-ph/0602091 [hep-ph]

[189] Ma Y-Q, Wang K, Chao K-T (2011). QCD radiative corrections to $$\chi _{cJ}$$ production at hadron colliders. Phys. Rev. D.

[190] CMS Collaboration, S. Chatrchyan et al., Measurement of the relative prompt production rate of $$\chi _{c2}$$ TeV. Eur. Phys. J. C **72**, 2251 (2012). arXiv:1210.0875 [hep-ex] 10.1140/epjc/s10052-012-2251-3 PMC4370953 25814834

[191] CDF Collaboration, A. Abulencia et al., Measurement of $$\sigma _{\chi _{c2}}{\cal B}(\chi _{c2} \rightarrow J/\psi \gamma )/\sigma _{\chi _{c1}} {\cal B}(\chi _{c1} \rightarrow J/\psi \gamma )$$ = 1.96 TeV. Phys. Rev. Lett. **98**, 232001 (2007). arXiv:hep-ex/0703028 [HEP-EX]

[192] A. Likhoded, A. Luchinsky, S. Poslavsky, Hadronic production of $$\chi _c$$-mesons at LHC. arXiv:1305.2389 [hep-ph]

[193] Shao H-S (2013). HELAC-Onia: an automatic matrix element generator for heavy quarkonium physics. Comput. Phys. Commun..

[194] Baranov S, Lipatov A, Zotov N (2012). Prompt $${\text{ J }}/\psi $$ production at LHC: new evidence for the $$k_t$$-factorization. Phys. Rev. D.

[195] Baranov S (2011). On the sigma($$\chi _{c1}$$)/sigma($$\chi _{c2}$$) ratio in the k(t)-factorization approach. Phys. Rev. D.

[196] Gong B, Lansberg J-P, Lorce C, Wang J (2013). Next-to-leading-order QCD corrections to the yields and polarisations of $${\text{ J }}/\psi $$ and $${\Upsilon }$$ directly produced in association with a Z boson at the LHC. JHEP.

[197] ATLAS Collaboration, G. Aad et al., Measurement of Upsilon production in 7 TeV pp collisions at ATLAS. Phys. Rev. D **87**(5), 052004 (2013). arXiv:1211.7255 [hep-ex]

[198] CMS Collaboration, S. Chatrchyan et al., Measurement of the $$\Upsilon (1S), \Upsilon (2S)$$ = 7 TeV. Phys. Lett. B **727**, 101–125 (2013). arXiv:1303.5900 [hep-ex]

[199] ALICE Collaboration, B.B. Abelev et al., Measurement of quarkonium production at forward rapidity in pp collisions at $$\sqrt{s} = 7$$ TeV. Eur. Phys. J. C **74**(8), 2974 (2014). arXiv:1403.3648 [nucl-ex] 10.1140/epjc/s10052-014-2974-4 PMC4370879 25814905

[200] ATLAS Collaboration, G. Aad et al., Observation of a new $$\chi _b$$ at ATLAS. Phys. Rev. Lett. **108**, 152001 (2012). arXiv:1112.5154 [hep-ex] 10.1103/PhysRevLett.108.152001 22587245

[201] CMS Collaboration, V. Khachatryan et al., Measurement of the production cross section ratio $$\sigma (\chi _{b2}(1\text{ P }))/ \sigma (\chi _{b1}(1\text{ P }))$$ = 8 TeV. arXiv:1409.5761 [hep-ex]

[202] LHCb Collaboration, R. Aaij et al., Measurement of the $$\chi _b(3P)$$ production. JHEP **1410**, 88 (2014). arXiv:1409.1408 [hep-ex]

[203] LHCb Collaboration, R. Aaij et al., Study of $$\chi _{{\text{ b }}}$$. Eur. Phys. J. C **74**(10), 3092 (2014). arXiv:1407.7734 [hep-ex]

[204] ATLAS Collaboration, G. Aad et al., Measurement of the $$\Upsilon \text{(1S) }$$ TeV in ATLAS. Phys. Lett. B **705**, 9–27 (2011). arXiv:1106.5325 [hep-ex]

[205] CMS Collaboration, V. Khachatryan et al., Upsilon production cross-section in $${\text{ pp }}$$ TeV. Phys. Rev. D **83**, 112004 (2011). arXiv:1012.5545 [hep-ex]

[206] LHCb Collaboration, R. Aaij et al., Production of $${\text{ J }}/\psi $$ TeV. JHEP **1306**, 064 (2013). arXiv:1304.6977 [hep-ex]

[207] LHCb Collaboration, R. Aaij et al., Measurement of the fraction of $$\Upsilon \text{(1S) }$$. JHEP **11**, 031 (2012). arXiv:1209.0282 [hep-ex]

[208] LHCb Collaboration, R. Aaij et al., Measurement of upsilon production in pp collisions at $$\sqrt{s}$$ = 7 TeV. Eur. Phys. J. C **72**, 2025 (2012). arXiv:1202.6579 [hep-ex]

[209] CDF Collaboration, T. Affolder et al., Production of $$\Upsilon (1S)$$ TeV. Phys. Rev. Lett. **84**, 2094–2099 (2000). arXiv:hep-ex/9910025 [hep-ex]

[210] LHCb Collaboration, R. Aaij et al., *Measurement of*$${\text{ B }^{+}_{c}}$$ TeV. arXiv:1411.2943 [hep-ex]

[211] C.-H. Chang, C. Driouichi, P. Eerola, X.G. Wu, BCVEGPY: an event generator for hadronic production of the $$\text{ B }_{c}$$ meson. Comput. Phys. Commun. **159**, 192–224 (2004). arXiv:hep-ph/0309120 [hep-ph]

[212] C.-H. Chang, J.-X. Wang, X.-G. Wu, BCVEGPY2.0: a upgrade version of the generator BCVEGPY with an addendum about hadroproduction of the P-wave B(c) states. Comput. Phys. Commun. **174**, 241–251 (2006). arXiv:hep-ph/0504017 [hep-ph]

[213] Chang C-H, Chen Y-Q (1993). The hadronic production of the B(c) meson at Tevatron, CERN LHC and SSC. Phys. Rev. D.

[214] C.-H. Chang, Y.-Q. Chen, G.-P. Han, H.-T. Jiang, On hadronic production of the B(c) meson. Phys. Lett. B **364**, 78–86 (1995). arXiv:hep-ph/9408242 [hep-ph]

[215] C.-H. Chang, Y.-Q. Chen, R.J. Oakes, Comparative study of the hadronic production of B(c) mesons. Phys. Rev. D **54**, 4344–4348 (1996). arXiv:hep-ph/9602411 [hep-ph] 10.1103/physrevd.54.4344 10021115

[216] C.-H. Chang, C.-F. Qiao, J.-X. Wang, X.-G. Wu, The color-octet contributions to P-wave $$\text{ B }_{c}$$ meson hadroproduction. Phys. Rev. D **71**, 074012 (2005). arXiv:hep-ph/0502155 [hep-ph]

[217] K. Kolodziej, A. Leike, R. Ruckl, Production of B(c) mesons in hadronic collisions. Phys. Lett. B **355**, 337–344 (1995). arXiv:hep-ph/9505298 [hep-ph]

[218] A. Berezhnoy, A. Likhoded, M. Shevlyagin, Hadronic production of B(c) mesons. Phys. Atom. Nucl. **58**, 672–689 (1995). arXiv:hep-ph/9408284 [hep-ph]

[219] Berezhnoy A, Kiselev V, Likhoded A (1996). Photonic production of S- and P wave B/c states and doubly heavy baryons. Z. Phys. A.

[220] Baranov S (1997). Pair production of B(c)* mesons in $${\text{ pp }}$$ and $$\gamma \gamma $$ collisions. Phys. Rev. D.

[221] LHCb Collaboration, R. Aaij et al., Search for the doubly charmed baryon $$\Xi _{cc}^{+}$$. JHEP **1312**, 090 (2013). arXiv:1310.2538 [hep-ex]

[222] SELEX Collaboration, M. Mattson et al., First observation of the doubly charmed baryon Xi+(cc). Phys. Rev. Lett. **89**, 112001 (2002). arXiv:hep-ex/0208014 [hep-ex] 10.1103/PhysRevLett.89.112001 12225136

[223] SELEX Collaboration, A. Ocherashvili et al., Confirmation of the double charm baryon Xi+(cc)(3520) via its decay to p $${\text{ D }}^{+}$$. Phys. Lett. B **628**, 18–24 (2005). arXiv:hep-ex/0406033 [hep-ex]

[224] Faccioli P, Lourenco C, Seixas J, Wohri HK (2010). Towards the experimental clarification of quarkonium polarization. Eur. Phys. J. C.

[225] Collins JC, Soper DE (1977). Angular distribution of dileptons in high-energy hadron collisions. Phys. Rev. D.

[226] Gottfried K, Jackson JD (1964). On the connection between production mechanism and decay of resonances at high-energies. Nuovo Cim..

[227] Braaten E, Kang D, Lee J, Yu C (2009). Optimal spin quantization axes for the polarization of dileptons with large transverse momentum. Phys. Rev. D.

[228] Palestini S (2011). Angular distribution and rotations of frame in vector meson decays into lepton pairs. Phys. Rev. D.

[229] LHCb Collaboration, R. Aaij et al., Measurement of $$J/\psi $$ TeV. Eur. Phys. J. C **73**(11), 2631 (2013). arXiv:1307.6379 [hep-ex]

[230] LHCb Collaboration, R. Aaij et al., Measurement of $$\psi (2S)$$ TeV. Eur. Phys. J. C **74**(5), 2872 (2014). arXiv:1403.1339 [hep-ex]

[231] H. Shao, H. Han, Y. Ma, C. Meng, Y. Zhang et al., Yields and polarizations of prompt $${\text{ J }}/\psi $$ production in hadronic collisions. arXiv:1411.3300 [hep-ph]

[232] CMS Collaboration, S. Chatrchyan et al., Measurement of the prompt $$J/\psi $$ = 7 TeV. Phys. Lett. B **727**, 381–402 (2013). arXiv:1307.6070 [hep-ex]

[233] CMS Collaboration, S. Chatrchyan et al., Measurement of the $$\Upsilon \text{(1S) }$$ TeV. Phys. Rev. Lett. **110**, 081802 (2013). arXiv:1209.2922 [hep-ex]

[234] Shao H-S, Chao K-T (2014). Spin correlations in polarizations of P-wave charmonia $$\chi _{cJ}$$ and impact on $$J/\psi $$ polarization. Phys. Rev. D.

[235] Faccioli P, Lourenco C, Seixas J, Wohri HK (2011). Determination of $${\chi _c}$$ and $${\chi _b}$$ polarizations from dilepton angular distributions in radiative decays. Phys. Rev. D.

[236] NA60 Collaboration, R. Arnaldi, $${\text{ J }}/\psi $$ production in p–A and A–A collisions at fixed target experiments. Nucl. Phys. A **830** 345C–352C (2009). arXiv:0907.5004 [nucl-ex]

[237] Faccioli P, Seixas J (2012). Observation of $$\chi _c$$ and $$\chi _b$$ nuclear suppression via dilepton polarization measurements. Phys. Rev. D.

[238] PHENIX Collaboration, A. Adare et al., Transverse momentum dependence of $${\text{ J }}/\psi $$ = 200 GeV. Phys. Rev. D **82**, 012001 (2010). arXiv:0912.2082 [hep-ex]

[239] STAR Collaboration, L. Adamczyk et al., $${\text{ J }}/\psi $$ GeV. Phys. Lett. B **722**, 55–62 (2013). arXiv:1208.2736 [nucl-ex]

[240] PHENIX Collaboration, A. Adare et al., Ground and excited charmonium state production in $${{\text{ pp }}}$$ GeV. Phys. Rev. D **85**, 092004 (2012). arXiv:1105.1966 [hep-ex]

[241] STAR Collaboration, L. Adamczyk et al., $$J/\psi $$ = 200 GeV in STAR. arXiv:1311.1621 [nucl-ex]

[242] CDF Collaboration, T. Affolder et al., Measurement of $$J/\psi $$ TeV. Phys. Rev. Lett. **85**, 2886–2891 (2000). arXiv:hep-ex/0004027 [hep-ex]

[243] CDF Collaboration, F. Abe et al., $$J/\psi $$ and $$\psi (2S)$$ production in $${\text{ p }\overline{\text{ p }}}$$ collisions at $$\sqrt{s} = 1.8$$ TeV. Phys. Rev. Lett. **79**, 572–577 (1997)

[244] CDF Collaboration, A. Abulencia et al., Polarization of $$J/\psi $$ = 1.96 TeV. Phys. Rev. Lett. **99**, 132001 (2007). arXiv:0704.0638 [hep-ex] 10.1103/PhysRevLett.99.132001 17930577

[245] ALICE Collaboration, B. Abelev et al., $$J/\psi $$ TeV. Phys. Rev. Lett. **108**, 082001 (2012). arXiv:1111.1630 [hep-ex]

[246] LHCb Collaboration, R. Aaij et al., Measurement of the ratio of prompt $$\chi _{c}$$ TeV. Phys. Lett. B **718**, 431–440 (2012). arXiv:1204.1462 [hep-ex]

[247] LHCb Collaboration, R. Aaij et al., Measurement of $$\psi (2S)$$ = 7 TeV. Eur. Phys. J. C **72**, 2100 (2012). arXiv:1204.1258 [hep-ex]

[248] HERA-B Collaboration, I. Abt et al., $$J/\psi $$) decays in 920-GeV pA interactions. Phys. Lett. B **561**, 61–72 (2003). arXiv:hep-ex/0211033 [hep-ex]

[249] E771 Collaboration, T. Alexopoulos et al., Measurement of $${\text{ J }}/\psi $$ total cross-sections in 800 GeV/c p-Si interactions. Phys. Lett. B **374**, 271–276 (1996)

[250] E672s, E706 Collaboration, A. Gribushin et al., Production of $$J/\psi $$ mesons in p-Be collisions at 530 GeV/c and 800 GeV/c. Phys. Rev. D **62**, 012001 (2000). arXiv:hep-ex/9910005 [hep-ex]

[251] E-771 Collaboration, T. Alexopoulos et al., Differential cross-sections of $${\text{ J }}/\psi $$ in 800 GeV/c p-Si interactions. Phys. Rev. D **55**, 3927–3932 (1997)

[252] NuSea Collaboration, T. Chang et al., $$J/\psi $$ Cu interactions. Phys. Rev. Lett. **91**, 211801 (2003). arXiv:hep-ex/0308001 [hep-ex] 10.1103/PhysRevLett.91.211801 14683289

[253] HERA-B Collaboration, I. Abt et al., Angular distributions of leptons from $$J/\psi $$ produced in 920 GeV fixed-target proton–nucleus collisions. Eur. Phys. J. C **60**, 517–524 (2009). arXiv:0901.1015 [hep-ex]

[254] HERA-B Collaboration, I. Abt et al., A Measurement of the $$\psi ^\prime $$ production ratio in 920-GeV proton-nucleus interactions. Eur. Phys. J. C **49**, 545–558 (2007). arXiv:hep-ex/0607046 [hep-ex]

[255] CDF Collaboration, D. Acosta et al., $$\Upsilon $$ production and polarization in $${\text{ p }\overline{\text{ p }}}$$ collisions at $$\sqrt{s}=$$ 1.8 TeV. Phys. Rev. Lett. **88**, 161802 (2002) 10.1103/PhysRevLett.88.161802 11955227

[256] CDF Collaboration, T. Aaltonen et al., Measurements of angular distributions of muons from $$\Upsilon $$ meson decays in $${\text{ p }\overline{\text{ p }}}$$ collisions at $$\sqrt{s}=1.96$$ TeV. Phys. Rev. Lett. **108**, 151802 (2012). arXiv:1112.1591 [hep-ex] 10.1103/PhysRevLett.108.151802 22587242

[257] D0 Collaboration, V. Abazov et al., Measurement of the polarization of the $$\Upsilon \text{(1S) }$$ = 1.96 TeV. Phys. Rev. Lett. **101**, 182004 (2008). arXiv:0804.2799 [hep-ex] 10.1103/PhysRevLett.101.182004 18999821

[258] UA5 Collaboration, G. Alner et al., Scaling violations in multiplicity distributions at 200 GeV and 900 GeV. Phys. Lett. B **167**, 476–480 (1986)

[259] Wang X-N, Gyulassy M (1992). A systematic study of particle production in $$p$$ + $$p$$ (anti-p) collisions via the HIJING model. Phys. Rev. D.

[260] Sjostrand T, van Zijl M (1987). A multiple interaction model for the event structure in hadron collisions. Phys. Rev. D.

[261] P. Bartalini, L. Fano, Multiple partonic interactions at the LHC. in *Proceedings, 1st International Workshop, MPI’08, Perugia, Italy, October* 27–31, 2008, DESY-PROC-2009-06. (2010). arXiv:1003.4220 [hep-ex]

[262] R. Maciula, M. Luszczak, A. Szczurek, Production of charm quark/antiquark pairs at LHC. PoS QNP2012 **125** (2012). arXiv:1207.6533 [hep-ph]

[263] S. Porteboeuf, R. Granier de Cassagnac, J$$/\psi $$ yield vs. multiplicity in proton-proton collisions at the LHC. Nucl. Phys. Proc. Suppl. **214**, 181–184 (2011). arXiv:1012.0719 [hep-ex]

[264] K. Werner, I. Karpenko, T. Pierog, M. Bleicher, K. Mikhailov, Evidence for hydrodynamic evolution in proton–proton scattering at 900 GeV. Phys. Rev. C **83**, 044915 (2011). arXiv:1010.0400 [nucl-th]

[265] Lang T, Bleicher M (2013). Possibility for $$J/\psi $$ suppression in high-multiplicity proton-proton collisions at $$\sqrt{s_{NN}}=7$$ TeV. Phys. Rev. C.

[266] ALICE Collaboration, B. Abelev et al., $$J/\psi $$ TeV. Phys. Lett. B **712**, 165–175 (2012). arXiv:1202.2816 [hep-ex]

[267] Ferreiro E, Pajares C (2012). High multiplicity $${\text{ pp }}$$ events and $$J/\psi $$ production at LHC. Phys. Rev. C.

[268] CMS Collaboration, S. Chatrchyan et al., Event activity dependence of Y(nS) production in $$\sqrt{s_{NN}}$$=2.76 TeV pp collisions. JHEP **04**, 103 (2014). arXiv:1312.6300 [nucl-ex]

[269] ALICE Collaboration, J. Adam et al., Measurement of charm and beauty production at central rapidity versus charged-particle multiplicity in proton–proton collisions at $$\mathbf{\sqrt{{ s}}}=7$$ TeV. arXiv:1505.00664 [nucl-ex]

[270] E. Ferreiro, C. Pajares, Open charm production in high multiplicity proton–proton events at the LHC. arXiv:1501.03381 [hep-ph]

[271] H. Drescher, M. Hladik, S. Ostapchenko, T. Pierog, K. Werner, Parton based Gribov–Regge theory. Phys. Rept. **350**, 93–289 (2001). arXiv:hep-ph/0007198 [hep-ph] 10.1103/PhysRevLett.92.072301 14995841

[272] K. Werner, B. Guiot, I. Karpenko, T. Pierog, Analysing radial flow features in $${\text{ p-Pb }}$$ collisions at several TeV by studying identified particle production in EPOS3. Phys. Rev. C **89**(6), 064903 (2014). arXiv:1312.1233 [nucl-th]

[273] R. Field, The sources of $$b$$ quarks at the Tevatron and their correlations. Phys. Rev. D **65**, 094006 (2002). arXiv:hep-ph/0201112 [hep-ph]

[274] LHCb Collaboration, R. Aaij et al., Observation of double charm production involving open charm in pp collisions at $$\sqrt{s}$$ = 7 TeV. JHEP **1206**, 141 (2012). doi:10.1007/JHEP06(2012)141. arXiv:1205.0975 [hep-ex] [Erratum: JHEP **1403**, 108 (2014). doi:10.1007/JHEP03(2014)108

[275] CMS Collaboration, V. Khachatryan et al., Measurement of $$B\bar{B}$$ TeV. JHEP **1103**, 136 (2011). arXiv:1102.3194 [hep-ex]

[276] ALICE Collaboration, S. Bjelogrlić, Heavy-flavour correlations in pp, pPb and PbPb collisions. Nucl. Phys. A **931**, 563–568 (2014)

[277] STAR Collaboration, B. Abelev et al., J/psi production at high transverse momentum in $${\text{ pp }}$$ = 200 GeV. Phys. Rev. C **80**, 041902 (2009). arXiv:0904.0439 [nucl-ex]

[278] LHCb Collaboration, R. Aaij et al., Observation of $$J/\psi $$ TeV. Phys. Lett. B **707**, 52–59 (2012). arXiv:1109.0963 [hep-ex]

[279] D0 Collaboration, V.M. Abazov et al., Observation and studies of double $$J/\psi $$ production at the Tevatron. Phys. Rev. D **90**(11), 111101 (2014). arXiv:1406.2380 [hep-ex]

[280] CMS Collaboration, V. Khachatryan et al., Measurement of prompt $$J/\psi $$ = 7 Tev. JHEP **1409**, 094 (2014). arXiv:1406.0484 [hep-ex]

[281] LHCb Collaboration, R. Aaij et al., Observation of associated production of a Z boson with a D meson in the forward region. JHEP **1404**, 091 (2014). arXiv:1401.3245 [hep-ex]

[282] D0 Collaboration, V.M. Abazov et al., Measurement of associated production of Z bosons with charm quark jets in $${{\text{ p }}\overline{{\text{ p }}}}$$ TeV. Phys. Rev. Lett. **112**(4), 042001 (2014). arXiv:1308.4384 [hep-ex] 10.1103/PhysRevLett.112.042001 24580440

[283] CMS Collaboration, S. Chatrchyan et al., Measurement of associated W + charm production in pp collisions at $$\sqrt{s}$$ = 7 TeV. JHEP **1402**, 013 (2014). arXiv:1310.1138 [hep-ex]

[284] ATLAS Collaboration, G. Aad et al., Measurement of the production cross section of prompt $$J/\psi $$ 7 TeV with the ATLAS detector. JHEP **1404** 172 (2014). arXiv:1401.2831 [hep-ex]

[285] ATLAS Collaboration, G. Aad et al., Observation and measurements of the production of prompt and non-prompt $$J/\psi $$ TeV with the ATLAS detector. arXiv:1412.6428 [hep-ex]

[286] CDF Collaboration, T. Aaltonen et al., Measurement of cross sections for $$b$$ = 1.96 TeV. Phys. Rev. D **79**, 052008 (2009). arXiv:0812.4458 [hep-ex]

[287] D0 Collaboration, V.M. Abazov et al., Measurement of the ratio of differential cross sections $${\sigma }({{\text{ p }}\overline{{\text{ p }}}} \rightarrow Z + b jet)/{\sigma }({{\text{ p }}\overline{{\text{ p }}}} \rightarrow Z + jet)$$ TeV. Phys. Rev. D **87**(9), 092010 (2013). arXiv:1301.2233 [hep-ex]

[288] ATLAS Collaboration, G. Aad et al., Measurement of the cross-section for $$b$$ TeV with the ATLAS detector. Phys. Lett. B **706**, 295–313 (2012). arXiv:1109.1403 [hep-ex]

[289] CMS Collaboration, S. Chatrchyan et al., Measurement of the cross section and angular correlations for associated production of a Z boson with b hadrons in pp collisions at $$\sqrt{s} =$$ 7 TeV. JHEP **1312**, 039 (2013). arXiv:1310.1349 [hep-ex]

[290] **LHCb** Collaboration, R. Aaij et al., Measurement of the Z+b-jet cross-section in pp collisions at $$\sqrt{s}=7$$ TeV in the forward region. arXiv:1411.1264 [hep-ex]

[291] CDF Collaboration, T. Aaltonen et al., Search for production of an $$\Upsilon $$ collision data set at CDF. Phys. Rev. D **91**(5), 052011 (2015). arXiv:1412.4827 [hep-ex]

[292] ATLAS Collaboration, G. Aad et al., Search for higgs and Z boson decays to $${\text{ J }}/\psi $$ with the ATLAS detector. Phys. Rev. Lett. **114**(12), 121801 (2015). arXiv:1501.03276 [hep-ex] 10.1103/PhysRevLett.114.121801 25860734

[293] A. Berezhnoy, V. Kiselev, A. Likhoded, A. Onishchenko, Doubly charmed baryon production in hadronic experiments. Phys. Rev. D **57**, 4385–4392 (1998). arXiv:hep-ph/9710339 [hep-ph]

[294] Kom C, Kulesza A, Stirling W (2011). Pair production of $${\text{ J }}/\psi $$ as a probe of double parton scattering at LHCb. Phys. Rev. Lett..

[295] Baranov S, Snigirev A, Zotov N (2011). Double heavy meson production through double parton scattering in hadronic collisions. Phys. Lett. B.

[296] Berezhnoy A, Likhoded A, Luchinsky A, Novoselov A (2012). Double $$c {\bar{c}}$$ production at LHCb. Phys. Rev. D.

[297] Baranov S, Snigirev A, Zotov N, Szczurek A, Schfer W (2013). Interparticle correlations in the production of $$J/\psi $$ pairs in proton–proton collisions. Phys. Rev. D.

[298] Kartvelishvili V, Esakiya S (1983). On hadron induced production of $${\text{ J }}/\psi $$ meson pairs (In Russian). Yad. Fiz..

[299] Humpert B, Mery P (1983). $$\psi \psi $$ production at colliders energy. Z. Phys. C.

[300] R. Vogt, S. Brodsky, Intrinsic charm contribution to double quarkonium hadroproduction. Phys. Lett. B **349**, 569–575 (1995). arXiv:hep-ph/9503206 [hep-ph]

[301] Li R, Zhang Y-J, Chao K-T (2009). Pair production of heavy quarkonium and B(c)(*) mesons at hadron colliders. Phys. Rev. D.

[302] Qiao C-F, Sun L-P, Sun P (2010). Testing charmonium production mechamism via polarized $${\text{ J }}/\psi $$ pair production at the LHC. J. Phys..

[303] Ko P, Yu C, Lee J (2011). Inclusive double-quarkonium production at the large hadron collider. JHEP.

[304] Berezhnoy A, Likhoded A, Luchinsky A, Novoselov A (2011). Double $${\text{ J }}/\psi $$-meson production at LHC and 4c-tetraquark state. Phys. Rev. D.

[305] Li Y-J, Xu G-Z, Liu K-Y, Zhang Y-J (2013). Relativistic correction to $$J/\psi $$ and $$\Upsilon $$ pair production. JHEP.

[306] Lansberg J-P, Shao H-S (2013). Production of $$J/\psi + \eta _{c}$$ versus $$J/\psi + J/\psi $$ at the LHC: importance of real $$\alpha ^{5}_{s}$$ corrections. Phys. Rev. Lett..

[307] L.-P. Sun, H. Han, K.-T. Chao, Impact of $$J/\psi $$ pair production at the LHC and predictions in nonrelativistic QCD. arXiv:1404.4042 [hep-ph]

[308] J.-P. Lansberg, H.-S. Shao, $$J/\psi $$ pair production at large momenta: indications for double-parton scatterings and large $$\alpha _{s}^{5}$$ contributions. Phys. Lett. B **751**, 479–486 (2015). arXiv:1410.8822 [hep-ph]

[309] Mao S, Wen-Gan M, Gang L, Ren-You Z, Lei G (2011). QCD corrections to $$J/\psi $$ plus $$Z^0$$-boson production at the LHC. JHEP.

[310] M. Strikman, D. Treleani, Measuring double parton distributions in nucleons at proton nucleus colliders. Phys. Rev. Lett. **88**, 031801 (2002). arXiv:hep-ph/0111468 [hep-ph] 10.1103/PhysRevLett.88.031801 11801053

[311] d’Enterria D, Snigirev AM (2013). Enhanced $${\text{ J }}/\psi $$ production from double parton scatterings in nucleus–nucleus collisions at the large Hadron collider. Phys. Lett. B.

[312] M.L. Miller, K. Reygers, S.J. Sanders, P. Steinberg, Glauber modeling in high energy nuclear collisions. Ann. Rev. Nucl. Part. Sci. **57**, 205–243 (2007). arXiv:nucl-ex/0701025 [nucl-ex]

[313] PHENIX Collaboration, A. Adare et al., Cold-nuclear-matter effects on heavy-quark production in $${\text{ d-Au }}$$ GeV. Phys. Rev. Lett. **109**(24), 242301 (2012). arXiv:1208.1293 [nucl-ex] 10.1103/PhysRevLett.109.242301 23368311

[314] PHENIX Collaboration, A. Adare et al., Cold-nuclear-matter effects on heavy-quark production at forward and backward rapidity in d+Au collisions at $$\sqrt{s_{{\text{ NN }}}}$$ = 200 GeV. arXiv:1310.1005 [nucl-ex] 10.1103/PhysRevLett.112.252301 25014805

[315] PHENIX Collaboration, A. Adare et al., Cross section for bb production via dielectrons in d+Au collisions at $$\sqrt{s_{{\text{ NN }}}}$$ = 200 GeV. arXiv:1405.4004 [nucl-ex]

[316] PHENIX Collaboration, S.S. Adler et al., $${\text{ J }}/\psi $$ = 200 GeV. Phys. Rev. Lett. **96**, 012304 (2006). arXiv:nucl-ex/0507032 [nucl-ex]

[317] PHENIX Collaboration, A. Adare et al., Cold nuclear matter effects on J/$$\psi $$ GeV. Phys. Rev. C **77**, 024912 (2008). arXiv:0711.3917 [nucl-ex]

[318] PHENIX Collaboration, A. Adare et al., Cold nuclear matter effects on $$J/\psi $$ GeV. Phys. Rev. Lett. **107**, 142301 (2011). arXiv:1010.1246 [nucl-ex] 10.1103/PhysRevLett.107.142301 22107186

[319] PHENIX Collaboration, A. Adare et al., Transverse-momentum dependence of the $${\text{ J }}/\psi $$ = 200 GeV. Phys. Rev. C **87**(3), 034904 (2013). arXiv:1204.0777 [nucl-ex]

[320] PHENIX Collaboration, A. Adare et al., Nuclear modification of $${\psi \text{(2S) }}$$ = 200 GeV). Phys. Rev. Lett. **111**(20), 202301 (2013). arXiv:1305.5516 [nucl-ex]

[321] PHENIX Collaboration, A. Adare et al., Upsilon(1S,2S,3S) production in d+Au and p+p collisions at $$\sqrt{s_{{\text{ NN }}}}$$ = 200 GeV and cold-nuclear matter effects. Phys. Rev. C **87**, 044909 (2013). arXiv:1211.4017 [nucl-ex]

[322] STAR Collaboration, J. Adams et al., Open charm yields in d + Au collisions at $$\sqrt{s_{\text{ NN }}}$$ = 200 GeV. Phys. Rev. Lett. **94**, 062301 (2005). arXiv:nucl-ex/0407006 [nucl-ex] 10.1103/PhysRevLett.94.062301 15783724

[323] STAR Collaboration, L. Adamczyk et al., Suppression of upsilon production in d+Au and Au+Au collisions at $$\sqrt{s_{{\text{ NN }}}}$$ = 200 GeV. arXiv:1312.3675 [nucl-ex]

[324] ALICE Collaboration, B.B. Abelev et al., Measurement of prompt D-meson production in $${{\text{ p-Pb }}}$$ = 5.02 TeV. arXiv:1405.3452 [nucl-ex]

[325] ALICE Collaboration, B.B. Abelev et al., $$J/\psi $$ = 5.02 TeV. JHEP **1402**, 073 (2014). arXiv:1308.6726 [nucl-ex]

[326] ALICE Collaboration, B.B. Abelev et al., Suppression of $$\psi $$ = 5.02 TeV. arXiv:1405.3796 [nucl-ex]

[327] ALICE Collaboration, J. Adam et al., Rapidity and transverse-momentum dependence of the inclusive J/$$\mathbf{\psi }$$ TeV. arXiv:1503.07179 [nucl-ex]

[328] ALICE Collaboration, B.B. Abelev et al., Production of inclusive $$\Upsilon $$ TeV. Phys. Lett. B **740**, 105–117 (2015). arXiv:1410.2234 [nucl-ex]

[329] ATLAS Collaboration, G. Aad et al., Measurement of differential $${\text{ J }/ \psi }$$ collisions with the ATLAS detector. Phys. Rev. C **92**(3), 034904 (2015). arXiv:1505.08141 [hep-ex]

[330] LHCb Collaboration, R. Aaij et al., Study of $$J/\psi $$ TeV. JHEP **1402**, 072 (2014). arXiv:1308.6729 [nucl-ex]

[331] LHCb Collaboration, R. Aaij et al., Study of upsilon. arXiv:1405.5152 [nucl-ex]

[332] NA3 Collaboration, J. Badier et al., Experimental J/$$\psi $$ hadronic production from 150 GeV/c to 280 GeV/c. Z. Phys. C **20**, 101 (1983)

[333] NA3 Collaboration, J. Badier et al., $$\psi \psi $$ protons, Phys. Lett. B **158**, 85 (1985)

[334] NA38 Collaboration, C. Baglin et al., Transverse momentum of $${\text{ J }{}}/\psi $$ produced in p-Cu, p-U, O-16-Cu, O-16-U and S-32-U collisions at 200 GeV per nucleon. Phys. Lett. B **262**, 362–368 (1991)

[335] NA38 Collaboration, C. Baglin et al., $${\psi \text{(2S) }}$$ production in p-W, p-U and S-U interactions at 200 GeV/nucleon. Phys. Lett. B **345**, 617–621 (1995)

[336] Baglin C, Baldisseri A, Bussiere A, Guillaud J, Kossakowski R (1991). $${\text{ J }}/\psi $$ and muon pair cross-sections in proton–nucleus and nucleus–nucleus collisions at 200 GeV per nucleon. Phys. Lett. B.

[337] NA38 Collaboration, C. Lourenco et al., $${\text{ J }}/\psi $$ and muon pair production in p-W and S-U collisions. Nucl. Phys. A **566**, 77C–85C (1994)

[338] NA38 Collaboration, M.C. Abreu et al., Charmonia production in 450 GeV/c proton induced reactions. Phys. Lett. B **444**, 516–522 (1998)

[339] Abreu M, Astruc J, Baglin C, Baldit A, Bedjidian M (1999). $${\text{ J }}/\psi $$ and $${\psi \text{(2S) }}$$ production in p, O and S induced reactions at SPS energies. Phys. Lett. B.

[340] NA50 Collaboration, B. Alessandro et al., Charmonia and Drell–Yan production in proton–nucleus collisions at the CERN SPS. Phys. Lett. B **553**, 167–178 (2003)

[341] NA50 Collaboration, B. Alessandro et al., $${\text{ J }}/\psi $$ production and their normal nuclear absorption in proton–nucleus collisions at 400 GeV. Eur. Phys. J. C **48**, 329 (2006). arXiv:nucl-ex/0612012 [nucl-ex]

[342] NA50 Collaboration, B. Alessandro et al., Charmonium production and nuclear absorption in p–A interactions at 450 GeV. Eur. Phys. J. C **33**, 31 (2004)

[343] NA50 Collaboration, B. Alessandro et al., Bottomonium and Drell–Yan production in p–A collisions at 450 GeV. Phys. Lett. B **635**, 260–269 (2006). arXiv:hep-ex/0603049 [hep-ex]

[344] NA60 Collaboration, R. Arnaldi et al., $${\text{ J }}/\psi $$ production in proton–nucleus collisions at 158 and 400 GeV. Phys. Lett. B **706**, 263–267 (2012). arXiv:1004.5523 [nucl-ex]

[345] Alde D, Baer H, Carey T, Garvey G, Klein A (1991). The A-dependence of $${\text{ J }}/\psi $$ and $${\psi \text{(2S) }}$$ production at 800 GeV/c. Phys. Rev. Lett..

[346] Alde D, Baer H, Carey T, Garvey G, Klein A (1991). Nuclear dependence of the production of upsilon resonances at 800 GeV. Phys. Rev. Lett..

[347] E789 Collaboration, M. Leitch et al., Nuclear dependence of neutral D meson production by 800 GeV/c protons. Phys. Rev. Lett. **72**, 2542–2545 (1994) 10.1103/PhysRevLett.72.2542 10055910

[348] Jansen D, Schub M, Mishra C, Ho P, Brown C (1995). Measurement of the bottom quark production cross-section in 800 GeV/c proton–gold collisions. Phys. Rev. Lett..

[349] Kowitt M, Gidal G, Ho P, Luk K, Pripstein D (1994). Production of $${\text{ J }}/\psi $$ at large x(F) in 800 GeV/c p-Cu and p-Be collisions. Phys. Rev. Lett..

[350] E789 Collaboration, M. Schub et al., Measurement of $$J/\psi $$ production in 800 GeV/c proton–gold collisions. Phys. Rev. D **52**, 1307 (1995) 10.1103/physrevd.52.1307 10019354

[351] Leitch M, Boissevain J, Brown C, Carey T, Chen Y (1995). Nuclear dependence of $${\text{ J }}/\psi $$ production by 800 GeV/c protons near x(F) = 0. Phys. Rev. D.

[352] NuSea Collaboration, M. Leitch et al., Measurement of $${\text{ J }}/\psi $$ suppression in p-A collisions at 800 GeV/c. Phys. Rev. Lett. **84**, 3256–3260 (2000). arXiv:nucl-ex/9909007 [nucl-ex]

[353] NuSea Collaboration, C. Brown et al., Observation of polarization in bottomonium production at $$\sqrt{s}$$ = 38.8 GeV. Phys. Rev. Lett. **86**, 2529–2532 (2001). arXiv:hep-ex/0011030 [hep-ex] 10.1103/PhysRevLett.86.2529 11289972

[354] HERA-B Collaboration, I. Abt et al., Measurement of $${\text{ D }}^{0}$$ Production in fixed target 920 GeV proton–nucleus collisions. Eur. Phys. J. C **52**, 531–542 (2007). arXiv:0708.1443 [hep-ex]

[355] HERA-B Collaboration, I. Abt et al., Improved measurement of the b-anti-b production cross section in 920 GeV fixed-target proton–nucleus collisions. Phys. Rev. D **73**, 052005 (2006). arXiv:hep-ex/0512030 [hep-ex]

[356] HERA-B Collaboration, I. Abt et al., Bottom production cross-section from double muonic decays of b-flavoured hadrons in 920 GeV proton–nucleus collision. Phys. Lett. B **650**, 103–110 (2007). arXiv:hep-ex/0612024 [hep-ex]

[357] HERA-B Collaboration, I. Abt et al., Measurement of the b anti-b production cross-section in 920 GeV fixed target proton nucleus collisions. Eur. Phys. J. C **26**, 345–355 (2003). arXiv:hep-ex/0205106 [hep-ex]

[358] HERA-B Collaboration, I. Abt et al., Measurement of the $$J/\psi $$ production cross section in 920 GeV/c fixed-target proton–nucleus interactions. Phys. Lett. B **638**, 407–414 (2006). arXiv:hep-ex/0512029 [hep-ex]

[359] HERA-B Collaboration, I. Abt et al., Kinematic distributions and nuclear effects of $${\text{ J }}/\psi $$ production in 920 GeV fixed-target proton–nucleus collisions. Eur. Phys. J. C **60**, 525–542 (2009). arXiv:0812.0734 [hep-ex]

[360] HERA-B Collaboration, I. Abt et al., Production of the charmonium states $$\chi _{c1}$$ = 41.6 GeV. Phys. Rev. D **79**, 012001 (2009). arXiv:0807.2167 [hep-ex]

[361] HERA-B Collaboration, I. Abt et al., Measurement of the $$\Upsilon $$ production cross-section in 920 GeV fixed-target proton–nucleus collisions. Phys. Lett. B **638**, 13–21 (2006). arXiv:hep-ex/0603015 [hep-ex]

[362] Kopeliovich B, Zakharov B (1991). Quantum effects and color transparency in charmonium photoproduction on nuclei. Phys. Rev. D.

[363] Vogt R (2010). Cold nuclear matter effects on $$J/\psi $$ and $$\Upsilon $$ production at the LHC. Phys. Rev. C.

[364] Eskola K, Paukkunen H, Salgado C (2009). EPS09: a new generation of NLO and LO nuclear parton distribution functions. JHEP.

[365] R. Nelson, R. Vogt, A. Frawley (2014). **(in progress)**

[366] Albacete J, Armesto N, Baier R, Barnafoldi G, Barrette J (2013). Predictions for p-Pb collisions at $$\sqrt{s_{NN}} = 5$$ TeV. Int. J. Mod. Phys. E.

[367] D. de Florian, R. Sassot, Nuclear parton distributions at next-to-leading order. Phys. Rev. D **69**, 074028 (2004). arXiv:hep-ph/0311227 [hep-ph]

[368] Conesa del Valle Z, Ferreiro E, Fleuret F, Lansberg J, Rakotozafindrabe A (2014). Open-beauty production in $${{\text{ p }-Pb}}$$ collisions at $$\sqrt{s_{NN}}$$ = 5 TeV: effect of the gluon nuclear densities. Nucl. Phys. A.

[369] Combridge B (1979). Associated production of heavy flavor states in $${\text{ pp }}$$ and $${\text{ p }\overline{\text{ p }}}$$ interactions: some QCD estimates. Nucl. Phys. B.

[370] S. Klein, R. Vogt, Inhomogeneous shadowing effects on $${\text{ J }}/\psi $$ production in d-A collisions. Phys. Rev. Lett. **91**, 142301 (2003). arXiv:nucl-th/0305046 [nucl-th] 10.1103/PhysRevLett.91.142301 14611517

[371] R. Vogt, Shadowing and absorption effects on $${\text{ J }}/\psi $$ production in d-A collisions. Phys. Rev. C **71**, 054902 (2005). arXiv:hep-ph/0411378 [hep-ph]

[372] Helenius I, Eskola KJ, Honkanen H, Salgado CA (2012). Impact-parameter dependent nuclear parton distribution functions: EPS09s and EKS98s and their applications in nuclear hard processes. JHEP.

[373] Ferreiro E, Fleuret F, Rakotozafindrabe A (2009). Transverse momentum dependence of $${\text{ J }}/\psi $$ shadowing effects. Eur. Phys. J. C.

[374] Ferreiro E, Fleuret F, Lansberg J, Rakotozafindrabe A (2010). Centrality, rapidity and transverse-momentum dependence of cold nuclear matter effects on $${\text{ J }}/\psi $$ production in d–Au, Cu–Cu and Au–Au collisions at $$\sqrt{s_{\text{ NN }}}$$ = 200 GeV. Phys. Rev. C.

[375] A. Rakotozafindrabe, E.G. Ferreiro, F. Fleuret, J.-P. Lansberg, N. Matagne, Cold nuclear matter effects in upsilon production in d–Au collisions at RHIC. PoS QNP **2012**, 159 (2012). arXiv:1207.3193 [hep-ph]

[376] Ferreiro E, Fleuret F, Lansberg J, Matagne N, Rakotozafindrabe A (2013). Upsilon production in p(d)–A collisions at RHIC and the LHC. Eur. Phys. J. C.

[377] Ferreiro E, Fleuret F, Lansberg J, Rakotozafindrabe A (2013). Impact of the nuclear modification of the gluon densities on $$J/\psi $$ production in $${\text{ p-Pb }}$$ collisions at $$\sqrt{s_{NN}} =$$ 5 TeV. Phys. Rev. C.

[378] Fujii H, Watanabe K (2013). Heavy quark pair production in high energy p–A collisions: open heavy flavors. Nucl. Phys. A.

[379] Fujii H, Watanabe K (2013). Heavy quark pair production in high energy p–A collisions: quarkonium. Nucl. Phys. A.

[380] J.P. Blaizot, F. Gelis, R. Venugopalan, High-energy p–A collisions in the color glass condensate approach. 2. Quark production. Nucl. Phys. A **743**, 57–91 (2004). arXiv:hep-ph/0402257 [hep-ph]

[381] H. Fujii, F. Gelis, R. Venugopalan, Quantitative study of the violation of k-perpendicular-factorization in hadroproduction of quarks at collider energies. Phys. Rev. Lett. **95**, 162002 (2005). arXiv:hep-ph/0504047 [hep-ph] 10.1103/PhysRevLett.95.162002 16241786

[382] Albacete JL, Armesto N, Milhano JG, Salgado CA (2009). Non-linear QCD meets data: a global analysis of lepton–proton scattering with running coupling BK evolution. Phys. Rev. D.

[383] B. Ducloué, T. Lappi, H. Mäntysaari, Forward $$J/\psi $$ production in proton–nucleus collisions at high energy. arXiv:1503.02789 [hep-ph]

[384] Y.-Q. Ma, R. Venugopalan, H.-F. Zhang, $$J/\psi $$ production and suppression in high energy proton–nucleus collisions. arXiv:1503.07772 [hep-ph]

[385] Kopeliovich B, Potashnikova I, Pirner H, Schmidt I (2011). Heavy quarkonium production: nontrivial transition from p–A to $${\text{ AA }}$$ collisions. Phys. Rev. C.

[386] Kopeliovich B, Potashnikova I, Schmidt I (2011). Nuclear suppression of $${\text{ J }}/\psi $$: from RHIC to the LHC. Nucl. Phys. A.

[387] B.Z. Kopeliovich, A. Schafer, A.V. Tarasov, Nonperturbative effects in gluon radiation and photoproduction of quark pairs. Phys. Rev. D **62**, 054022 (2000). arXiv:hep-ph/9908245 [hep-ph]

[388] B. Kopeliovich, A. Tarasov, J. Hufner, Coherence phenomena in charmonium production off nuclei at the energies of RHIC and LHC. Nucl. Phys. A **696**, 669–714 (2001). arXiv:hep-ph/0104256 [hep-ph]

[389] J. Hufner, B. Kopeliovich, A.B. Zamolodchikov, Inelastic $${\text{ J }}/\psi $$ photoproduction off nuclei: gluon enhancement or double color exchange? Z. Phys. A **357**, 113–120 (1997). arXiv:nucl-th/9607033 [nucl-th]

[390] B. Kopeliovich, I. Potashnikova, I. Schmidt, M. Siddikov (2015). **(in progress)**

[391] I. Vitev, J.T. Goldman, M. Johnson, J. Qiu, Open charm tomography of cold nuclear matter. Phys. Rev. D **74**, 054010 (2006). arXiv:hep-ph/0605200 [hep-ph]

[392] I. Vitev, Non-abelian energy loss in cold nuclear matter. Phys. Rev. C **75**, 064906 (2007). arXiv:hep-ph/0703002 [hep-ph]

[393] Neufeld R, Vitev I, Zhang B-W (2011). A possible determination of the quark radiation length in cold nuclear matter. Phys. Lett. B.

[394] Arleo F, Peigné S, Sami T (2011). Revisiting scaling properties of medium-induced gluon radiation. Phys. Rev. D.

[395] Arleo F, Peigné S (2013). Heavy-quarkonium suppression in p-A collisions from parton energy loss in cold QCD matter. JHEP.

[396] Arleo F, Kolevatov R, Peigné S, Rustamova M (2013). Centrality and $$p_{\bot }$$ dependence of $$J/\psi $$ suppression in proton–nucleus collisions from parton energy loss. JHEP.

[397] S. Peigné, F. Arleo, R. Kolevatov, Medium-induced gluon radiation: an update. arXiv:1402.1671 [hep-ph]

[398] S. Peigné, R. Kolevatov, On the process-dependence of coherent medium-induced gluon radiation. arXiv:1405.4241 [hep-ph]

[399] Lourenço C, Vogt R, Wöhri HK (2009). Energy dependence of $${\text{ J }}/\psi $$ absorption in proton–nucleus collisions. JHEP.

[400] K. Eskola, V. Kolhinen, P. Ruuskanen, Scale evolution of nuclear parton distributions. Nucl. Phys. B **535**, 351 (1998). arXiv:hep-ph/9802350 [hep-ph]

[401] K. Eskola, V. Kolhinen, C. Salgado, The scale dependent nuclear effects in parton distributions for practical applications. Eur. Phys. J. C **9**, 61 (1999). arXiv:hep-ph/9807297 [hep-ph]

[402] J. Pumplin, D. Stump, J. Huston, H. Lai, P.M. Nadolsky et al., New generation of parton distributions with uncertainties from global QCD analysis. JHEP **0207**, 012 (2002). arXiv:hep-ph/0201195 [hep-ph]

[403] D. Stump, J. Huston, J. Pumplin, W.-K. Tung, H. Lai et al., Inclusive jet production, parton distributions, and the search for new physics. JHEP **0310**, 046 (2003). arXiv:hep-ph/0303013 [hep-ph]

[404] Arleo F, Peigné S (2014). Quarkonium suppression in heavy-ion collisions from coherent energy loss in cold nuclear matter. JHEP.

[405] F. Arleo, V.-N. Tram, A systematic study of $${\text{ J }}/\psi $$ suppression in cold nuclear matter. Eur. Phys. J. C **55**, 449–461 (2008). arXiv:hep-ph/0612043 [hep-ph]

[406] NA60 Collaboration, E. Scomparin, J/$$\psi $$ production in p–A collisions at 158 GeV and 400 GeV: recent results from the NA60 experiment. Nucl. Phys. A **830**, 239C (2009). arXiv:0907.3682 [nucl-ex]

[407] PHENIX Collaboration, C.L. da Silva, Quarkonia measurement in p+p and d+Au collisions at $$\sqrt{s_{NN}} = 200$$ GeV by PHENIX detector. Nucl. Phys. A **830**, 227C (2009). arXiv:0907.4696 [nucl-ex]

[408] D. McGlinchey, A. Frawley, R. Vogt, Impact parameter dependence of the nuclear modification of J/$$\psi $$ GeV. Phys. Rev. C **87**(5), 054910 (2013). arXiv:1208.2667 [nucl-th]

[409] R. Averbeck, N. Bastid, Z.C. del Valle, P. Crochet, A. Dainese et al., Reference heavy flavour cross sections in pp collisions at $$\sqrt{s} = 2.76$$ TeV. arXiv:1107.3243 [hep-ph]

[410] B. Kniehl, G. Kramer, I. Schienbein, H. Spiesberger, Hadroproduction of D and B mesons in a massive VFNS. AIP Conf. Proc. **792**, 867–870 (2005). arXiv:hep-ph/0507068 [hep-ph]

[411] PHENIX Collaboration, A. Adare et al., $${\text{ J }}/\psi $$. Phys. Rev. Lett. **98**, 232002 (2007)

[412] ALICE Collaboration, B. Abelev et al., Inclusive $$J/\psi $$ TeV. Phys. Lett. B **718**, 295–306 (2012). arXiv:1203.3641 [hep-ex]

[413] ALICE Collaboration, K. Aamodt et al., Rapidity and transverse momentum dependence of inclusive $${\text{ J }}/\psi $$. Phys. Lett. B **704**, 442–455 (2011). arXiv:1105.0380 [hep-ex]

[414] CDF Collaboration, D. Acosta et al., Measurement of the J/$$\psi $$ GeV. Phys. Rev. D **71**, 032001 (2005). arXiv:hep-ex/0412071

[415] F. Bossu, Z.C. del Valle, A. de Falco, M. Gagliardi, S. Grigoryan et al., Phenomenological interpolation of the inclusive $${\text{ J }}/\psi $$ cross section to proton–proton collisions at 2.76 TeV and 5.5 TeV. arXiv:1103.2394 [nucl-ex]

[416] ALICE and LHCbs Collaboration, Reference $${{\text{ pp }}}$$ TeV and comparisons between ALICE and LHCb results, LHCb-CONF-2013-013, CERN-LHCb-CONF-2013-013, ALICE-PUBLIC-2013-002, LHCB-CONF-2013-013-002 (2013)

[417] LHCb Collaboration, R. Aaij et al., Measurement of $$J/\psi $$ TeV. JHEP **1302**, 041 (2013). arXiv:1212.1045 [hep-ex]

[418] LHCb Collaboration, Reference $${{\text{ pp }}}$$ TeV and comparisons between ALICE and LHCb results, LHCb-CONF-2014-003, CERN-LHCb-CONF-2014-003 (2014)

[419] LHCb Collaboration, R. Aaij et al., Measurement of $$\Upsilon $$ TeV. Eur. Phys. J. C **74**(4), 2835 (2014). arXiv:1402.2539 [hep-ex]

[420] STAR Collaboration, B. Abelev et al., Erratum: transverse momentum and centrality dependence of high-$$p_T$$ GeV. Phys. Rev. Lett. **98**, 192301 (2007). arXiv:nucl-ex/0607012 [nucl-ex] 10.1103/PhysRevLett.98.192301 17677616

[421] ALICE Collaboration, S. Li, Measurements of the heavy-flavour nuclear modification factor in p-Pb collisions at $$\sqrt{s_{\text{ NN }}}$$ = 5.02 TeV with ALICE at the LHC. Nucl. Phys. A **931**, 546–551 (2014). arXiv:1408.1915 [hep-ex]

[422] Sharma R, Vitev I, Zhang B-W (2009). Light-cone wave function approach to open heavy flavor dynamics in QCD matter. Phys. Rev. C.

[423] ALICE Collaboration, R. Russo, Measurement of heavy-flavour production as a function of multiplicity in $${{\text{ pp }}}$$ collisions with ALICE. Nucl. Phys. A **931**, 552–557 (2014)

[424] CMS Collaboration, Measurements of the $$\text{ B }^{+}$$ = 5.02 TeV, CMS-PAS-HIN-14-004 (2014)

[425] CMS Collaboration, G.M. Innocenti, B-meson reconstruction performance and spectra in $${\text{ pp }}$$ collisions in CMS. Nucl. Phys. A **931**, 1184–1188 (2014)

[426] CMS Collaboration, Nuclear modification factor $${R_{\text{ pA }}}$$ collisions, CMS-PAS-HIN-14-007 (2014)

[427] Gelis F, Iancu E, Jalilian-Marian J, Venugopalan R (2010). The color glass condensate. Ann. Rev. Nucl. Part. Sci..

[428] Marquet C (2007). Forward inclusive dijet production and azimuthal correlations in p-A collisions. Nucl. Phys. A.

[429] Lappi T, Mantysaari H (2013). Forward dihadron correlations in deuteron–gold collisions with the Gaussian approximation of JIMWLK. Nucl. Phys. A.

[430] Kang Z-B, Vitev I, Xing H (2012). Dihadron momentum imbalance and correlations in d+Au collisions. Phys. Rev. D.

[431] M. Cacciari, P. Nason, R. Vogt, QCD predictions for charm and bottom production at RHIC. Phys. Rev. Lett. **95**, 122001 (2005). arXiv:hep-ph/0502203 [hep-ph] 10.1103/PhysRevLett.95.122001 16197066

[432] Ferreiro E, Fleuret F, Lansberg J, Rakotozafindrabe A (2009). Cold nuclear matter effects on $${\text{ J }}/\psi $$ production: intrinsic and extrinsic transverse momentum effects. Phys. Lett. B.

[433] ALICE Collaboration, J. Martín Blanco, $$J/\psi $$ production in p-Pb collisions with ALICE at the LHC. Nucl. Phys. A **931**, 612–616 (2014)

[434] ALICE Collaboration, I. Lakomov, Event activity dependence of inclusive $$J/{\psi }$$ TeV with ALICE at the LHC. Nucl. Phys. A **931**, 1179–1183 (2014). arXiv:1408.0702 [hep-ex]

[435] ALICE Collaboration, J. Adam et al., Centrality dependence of particle production in p-Pb collisions at $$\sqrt{s_{\text{ NN }}}$$= 5.02 TeV. arXiv:1412.6828 [nucl-ex]

[436] E. Ferreiro, $${\psi \text{(2S) }}$$ suppression in the comover interaction approach. arXiv:1411.0549 [hep-ph]

[437] H. Satz, Colour deconfinement and quarkonium binding. J. Phys. G **32**, R25 (2006). arXiv:hep-ph/0512217 [hep-ph]

[438] Ferreiro E, Fleuret F, Lansberg J, Rakotozafindrabe A (2013). $$J/\psi $$ and $$\psi ^{\prime }$$ production in proton(deuteron)–nucleus collisions: lessons from RHIC for the proton-lead LHC run. J. Phys. Conf. Ser..

[439] ALICE Collaboration, R. Arnaldi, Inclusive $$\psi $$(2S) production in p-Pb collisions with ALICE. Nucl. Phys. A (2014). arXiv:1407.7451 [nucl-ex]

[440] Y. Liu, C.M. Ko, T. Song, Hot medium effects on $$J/\psi $$ production in p+Pb collisions at $$\sqrt{s_{NN}}=5.02$$ TeV. Phys. Lett. B **728**, 437–442 (2014). arXiv:1309.5113 [nucl-th]

[441] CDF Collaboration, F. Abe et al., $$\Upsilon $$ TeV. Phys. Rev. Lett. **75**, 4358 (1995)

[442] Vogt R (2015). Shadowing effects on $$J/\psi $$ and $$\Upsilon $$ production at energies available at the CERN Large Hadron Collider. Phys. Rev. C.

[443] R. Granier de Cassagnac, A d-Au data-driven prediction of cold nuclear matter effects on $${\text{ J }}/\psi $$ production in Au–Au collisions at RHIC. J. Phys G **34**, S955–958 (2007). arXiv:hep-ph/0701222 [hep-ph]

[444] Rakotozafindrabe A, Ferreiro E, Fleuret F, Lansberg J (2010). On the theoretical and experimental uncertainties in the extraction of the $${\text{ J }}/\psi $$ absorption cross section in cold nuclear matter. J. Phys..

[445] Mueller A, Nason P (1985). Heavy particle content in QCD jets. Phys. Lett. B.

[446] Mangano ML, Nason P (1992). Heavy quark multiplicities in gluon jets. Phys. Lett. B.

[447] Banfi A, Salam GP, Zanderighi G (2007). Accurate QCD predictions for heavy-quark jets at the Tevatron and LHC. JHEP.

[448] Gyulassy M, Plumer M (1990). Jet quenching in dense matter. Phys. Lett. B.

[449] R. Baier, Y.L. Dokshitzer, A.H. Mueller, S. Peigne, D. Schiff, Radiative energy loss and p(T) broadening of high-energy partons in nuclei. Nucl. Phys. B **484**, 265–282 (1997). arXiv:hep-ph/9608322 [hep-ph]

[450] Thoma MH, Gyulassy M (1991). Quark damping and energy loss in the high temperature QCD. Nucl. Phys. B.

[451] Braaten E, Thoma MH (1991). Energy loss of a heavy fermion in a hot plasma. Phys. Rev. D.

[452] Braaten E, Thoma MH (1991). Energy loss of a heavy quark in the quark–gluon plasma. Phys. Rev. D.

[453] C. Herzog, A. Karch, P. Kovtun, C. Kozcaz, L. Yaffe, Energy loss of a heavy quark moving through N=4 supersymmetric Yang–Mills plasma. JHEP **0607**, 013 (2006). arXiv:hep-th/0605158 [hep-th]

[454] S.S. Gubser, Drag force in AdS/CFT. Phys. Rev. D **74**, 126005 (2006). arXiv:hep-th/0605182 [hep-th]

[455] Y.L. Dokshitzer, D. Kharzeev, Heavy quark colorimetry of QCD matter. Phys. Lett. B **519**, 199–206 (2001). arXiv:hep-ph/0106202 [hep-ph]

[456] N. Armesto, C.A. Salgado, U.A. Wiedemann, Medium induced gluon radiation off massive quarks fills the dead cone. Phys. Rev. D **69**, 114003 (2004). arXiv:hep-ph/0312106 [hep-ph]

[457] M. Djordjevic, M. Gyulassy, Heavy quark radiative energy loss in QCD matter. Nucl. Phys. A **733**, 265–298 (2004). arXiv:nucl-th/0310076 [nucl-th]

[458] B.-W. Zhang, E. Wang, X.-N. Wang, Heavy quark energy loss in nuclear medium. Phys. Rev. Lett. **93**, 072301 (2004). arXiv:nucl-th/0309040 [nucl-th] 10.1103/PhysRevLett.93.072301 15324227

[459] S. Wicks, W. Horowitz, M. Djordjevic, M. Gyulassy, Heavy quark jet quenching with collisional plus radiative energy loss and path length fluctuations. Nucl. Phys. A **783**, 493–496 (2007). arXiv:nucl-th/0701063 [nucl-th]

[460] A. Adil, I. Vitev, Collisional dissociation of heavy mesons in dense QCD matter. Phys. Lett. B **649**, 139–146 (2007). arXiv:hep-ph/0611109 [hep-ph]

[461] P. Braun-Munzinger, Quarkonium production in ultra-relativistic nuclear collisions: suppression versus enhancement. J. Phys. G **34**, S471–478 (2007). arXiv:nucl-th/0701093 [NUCL-TH]

[462] B.-W. Zhang, C.-M. Ko, W. Liu, Thermal charm production in a quark–gluon plasma in $${\text{ Pb-Pb }}$$ collisions at $$\sqrt{s_{\text{ NN }}}$$ = 5.5 TeV. Phys. Rev. C **77**, 024901 (2008). arXiv:0709.1684 [nucl-th]

[463] S. Batsouli, S. Kelly, M. Gyulassy, J. Nagle, Does the charm flow at RHIC? Phys. Lett. B **557**, 26–32 (2003). arXiv:nucl-th/0212068 [nucl-th]

[464] V. Greco, C. Ko, R. Rapp, Quark coalescence for charmed mesons in ultrarelativistic heavy ion collisions. Phys. Lett. B **595**, 202–208 (2004). arXiv:nucl-th/0312100 [nucl-th]

[465] A. Andronic, P. Braun-Munzinger, K. Redlich, J. Stachel, Statistical hadronization of charm in heavy ion collisions at SPS, RHIC and LHC. Phys. Lett. B **571**, 36–44 (2003). arXiv:nucl-th/0303036 [nucl-th]

[466] R. Rapp, H. van Hees, Heavy quarks in the quark–gluon plasma. in R.C. Hwa, X.-N. Wang, eds. Quark–Gluon Plasma 4, vol. 111. (World Scientific, Singapore, 2009). arXiv:0903.1096 [hep-ph]

[467] PHENIX Collaboration, A. Adare et al., Heavy-quark production and elliptic flow in Au$$+$$ GeV. Phys. Rev. C (2014). **(submitted)**. arXiv:1405.3301 [nucl-ex]

[468] PHENIX Collaboration, K. Adcox et al., Measurement of single electrons and implications for charm production in Au+Au collisions at $$\sqrt{s_{{\text{ NN }}}}$$ = 130 GeV. Phys. Rev. Lett. **88**, 192303 (2002). arXiv:nucl-ex/0202002 [nucl-ex] 10.1103/PhysRevLett.88.192303 12005627

[469] PHENIX Collaboration, A. Adare et al., Heavy quark production in p+p and energy loss and flow of heavy quarks in Au+Au collisions at $$\sqrt{s_{NN}}=200$$ GeV. Phys. Rev. C **84**, 044905 (2011). arXiv:1005.1627 [nucl-ex]

[470] PHENIX Collaboration, A. Adare et al., Energy loss and flow of heavy quarks in Au+Au collisions at $$\sqrt{s_{{\text{ NN }}}}$$ = 200 GeV. Phys. Rev. Lett. **98**, 172301 (2007). arXiv:nucl-ex/0611018 [nucl-ex]

[471] PHENIX Collaboration, S. Adler et al., Nuclear modification of electron spectra and implications for heavy quark energy loss in Au+Au collisions at $$\sqrt{s_{{\text{ NN }}}}$$ = 200 GeV. Phys. Rev. Lett. **96**, 032301 (2006). arXiv:nucl-ex/0510047 [nucl-ex] 10.1103/PhysRevLett.96.032301 16486687

[472] PHENIX Collaboration, S. Adler et al., Centrality dependence of charm production from single electrons measurement in Au + Au collisions at $$\sqrt{s_{{\text{ NN }}}}$$ = 200 GeV. Phys. Rev. Lett. **94**, 082301 (2005). arXiv:nucl-ex/0409028 [nucl-ex] 10.1103/PhysRevLett.94.082301 15783878

[473] PHENIX Collaboration, A. Adare et al., System-size dependence of open-heavy-flavor production in nucleus–nucleus collisions at $$\sqrt{s_{_{NN}}}$$=200 GeV. Phys. Rev. C **90**(3), 034903 (2014). arXiv:1310.8286 [nucl-ex]

[474] PHENIX Collaboration, A. Adare et al., Nuclear-modification factor for open-heavy-flavor production at forward rapidity in Cu+Cu collisions at $$\sqrt{s_{NN}}=200$$ GeV. Phys. Rev. C **86**, 024909 (2012). arXiv:1204.0754 [nucl-ex]

[475] STAR Collaboration, L. Adamczyk et al., Observation of $$\text{ D }^{0}$$ meson nuclear modifications in Au+Au collisions at $$\sqrt{s_{_\text{ NN }}}$$ = 200 GeV. Phys. Rev. Lett. **113**, 142301 (2014). arXiv:1404.6185 [nucl-ex] 10.1103/PhysRevLett.113.142301 25325635

[476] STAR Collaboration, L. Adamczyk et al., Elliptic flow of non-photonic electrons in Au+Au collisions at $$\sqrt{s_{\text{ NN }}} = $$ 200, 62.4 and 39 GeV. Phys. Lett. B (2014). **(Submitted)**. arXiv:1405.6348 [hep-ex]

[477] ALICE Collaboration, B. Abelev et al., Suppression of high transverse momentum D mesons in central Pb-Pb collisions at $$\sqrt{s_{NN}}=2.76$$ TeV. JHEP **1209**, 112 (2012). arXiv:1203.2160 [nucl-ex]

[478] ALICE Collaboration, B.B. Abelev et al., Azimuthal anisotropy of D meson production in Pb–Pb collisions at $$\sqrt{s_{\text{ NN }}} = 2.76$$ TeV. Phys. Rev. C **90**, 034904 (2014). arXiv:1405.2001 [nucl-ex]

[479] ALICE Collaboration, B. Abelev et al., D meson elliptic flow in non-central Pb–Pb collisions at $$\sqrt{s_{NN}} = 2.76$$ TeV. Phys. Rev. Lett. **111**, 102301 (2013). arXiv:1305.2707 [nucl-ex] 10.1103/PhysRevLett.111.102301 25166659

[480] ALICE Collaboration, J. Adam et al., Inclusive, prompt and non-prompt J/$$\psi $$ = 2.76 TeV. arXiv:1504.07151 [nucl-ex]

[481] CMS Collaboration, S. Chatrchyan et al., Evidence of b-Jet Quenching in $$\text{ Pb-Pb }$$ TeV. Phys. Rev. Lett. **113**(13), 132301 (2014). arXiv:1312.4198 [nucl-ex] 10.1103/PhysRevLett.113.132301 25302881

[482] CMS Collaboration, S. Chatrchyan et al., Suppression of non-prompt $$J/\psi $$ TeV. JHEP **1205**, 063 (2012). arXiv:1201.5069 [nucl-ex]

[483] ATLAS Collaboration, Measurement of the centrality dependence of open heavy flavour production in lead-lead collisions at $${\sqrt{s}}$$ = 2.76 TeV with the ATLAS detector, ATLAS-CONF-2012-050, ATLAS-COM-CONF-2012-081 (2012)

[484] STAR Collaboration, M. Mustafa, Measurements of non-photonic electron production and azimuthal anisotropy in $$\sqrt{s_{NN}} = 39, 62.4$$ and 200 GeV Au+Au collisions from STAR at RHIC. Nucl. Phys. A **904**–**905**, 665c–668c (2013). arXiv:1210.5199 [nucl-ex]

[485] ALICE Collaboration, S. Sakai, Measurement of $$R_{AA}$$ = 2.76 TeV with ALICE, Nucl. Phys. A **904**–**905**, 661c–664c (2013)

[486] ATLAS Collaboration, D.V. Perepelitsa, Measurement of muon tagged open heavy flavor production in Pb+Pb collisions at 2.76 TeV with ATLAS. Nucl. Phys. A **904**–**905**, 669c–672c (2013)

[487] STAR Collaboration, Z. Ye, Open charm hadron production in p+p, Au$$+$$Au and U$$+$$U collisions at STAR. Nucl. Phys. A **931**, 520–524 (2014)

[488] ALICE Collaboration, A. Grelli, D meson nuclear modification factors in Pb-Pb collisions at $$\sqrt{s_{NN}}=2.76$$ TeV with the ALICE detector. Nucl. Phys. A **904**–**905**, 635c–638c (2013). arXiv:1210.7332 [hep-ex]

[489] ALICE Collaboration, G.M. Innocenti, $${\text{ D }}^{+}_{s}$$ TeV with the ALICE detector. Nucl. Phys. A **904**–**905**, 433c–436c (2013). arXiv:1210.6388 [nucl-ex]

[490] I. Kuznetsova, J. Rafelski, Non-equilibrium heavy flavored hadron yields from chemical equilibrium strangeness-rich QGP. J. Phys. G **35**, 044043 (2008). arXiv:0801.0788 [hep-ph]

[491] M. He, R.J. Fries, R. Rapp, $$\text{ D }^{+}_{s}$$-meson as quantitative probe of diffusion and hadronization in nuclear collisions. Phys. Rev. Lett. **110**(11), 112301 (2013). arXiv:1204.4442 [nucl-th] 10.1103/PhysRevLett.110.112301 25166524

[492] ALICE Collaboration, A. Festanti, Heavy-flavour production and nuclear modification factor in Pb–Pb collisions at $$\sqrt{s_{\text{ NN }}}$$=2.76 TeV with ALICE. Nucl. Phys. A **931**, 514–519 (2014). arXiv:1407.6541 [nucl-ex]

[493] Mischke A (2009). A new correlation method to identify and separate charm and bottom production processes at RHIC. Phys. Lett. B.

[494] CMS Collaboration, $${\text{ J }}/\psi $$, CMS Physics Analysis Summary CMS-PAS-HIN-12-014 (2012). https://cdsweb.cern.ch/record/1472735

[495] Huang J, Kang Z-B, Vitev I (2013). Inclusive b-jet production in heavy ion collisions at the LHC. Phys. Lett. B.

[496] CMS Collaboration, S. Chatrchyan et al., Nuclear modification factor of high transverse momentum jets in $$\text{ Pb-Pb }$$ = 2.76 TeV. Tech. Rep. CMS-PAS-HIN-12-004 (2012)

[497] ALICE Collaboration, E. Bruna, D-meson nuclear modification factor and v$$_2$$ in Pb–Pb collisions at the LHC. J. Phys. Conf. Ser. **509**, 012080 (2014). arXiv:1401.1698 [nucl-ex]

[498] A.M. Poskanzer, S. Voloshin, Methods for analyzing anisotropic flow in relativistic nuclear collisions. Phys. Rev. C **5**, 1671–1678 (1998). arXiv:nucl-ex/9805001 [nucl-ex]

[499] STAR Collaboration, C. Adler et al., Elliptic flow from two and four particle correlations in Au+Au collisions at $$\sqrt{s_{\text{ NN }}}$$ = 130 GeV. Phys. Rev. C **66**, 034904 (2002). arXiv:nucl-ex/0206001 [nucl-ex]

[500] A. Bilandzic, R. Snellings, S. Voloshin, Flow analysis with cumulants: direct calculations. Phys. Rev. C **83**, 044913 (2011). arXiv:1010.0233 [nucl-ex]

[501] ALICE Collaboration, R. Bailhache, Heavy-flavour elliptic flow measured in $$\text{ Pb--Pb }$$ TeV with ALICE. Nucl. Phys. A **931**, 530–534 (2014)

[502] R. Balescu, *Equilibrium and Non-Equilibrium Statistical Mechanics* (Wiley, New York, 1975)

[503] H. Risken, *The Fokker-Planck Equation: Methods of solution and applications*. Springer series in synergetics (Springer, Berlin, 1989)

[504] Berrehrah H, Bratkovskaya E, Cassing W, Gossiaux PB, Aichelin J, Bleicher M (2014). Collisional processes of on-shell and off-shell heavy quarks in vacuum and in the quark–gluon–plasma. Phys. Rev. C.

[505] Berrehrah H, Gossiaux P-B, Aichelin J, Cassing W, Bratkovskaya E (2014). Dynamical collisional energy loss and transport properties of on- and off-shell heavy quarks in vacuum and in the Quark Gluon Plasma. Phys. Rev. C.

[506] T. Song, H. Berrehrah, D. Cabrera, J.M. Torres-Rincon, L. Tolos, W. Cassing, E. Bratkovskaya, Tomography of the quark–gluon–plasma by charm quarks. Phys. Rev. C **92**(1), 014910 (2015). arXiv:1503.03039 [nucl-th]

[507] Alberico W, Beraudo A, De Pace A, Molinari A, Monteno M (2013). Heavy flavors in $${\text{ AA }}$$ collisions: production, transport and final spectra. Eur. Phys. J. C.

[508] Alberico W, Beraudo A, De Pace A, Molinari A, Monteno M (2011). Heavy-flavour spectra in high energy nucleus–nucleus collisions. Eur. Phys. J. C.

[509] Beraudo A, De Pace A, Alberico W, Molinari A (2009). Transport properties and Langevin dynamics of heavy quarks and quarkonia in the quark gluon plasma. Nucl. Phys. A.

[510] Itō K (1951). On stochastic differential equations. Memoirs Am. Math. Soc..

[511] M. Djordjevic, Theoretical formalism of radiative jet energy loss in a finite size dynamical QCD medium. Phys. Rev. C **80**, 064909 (2009). arXiv:0903.4591 [nucl-th] 10.1103/PhysRevLett.101.022302 18764174

[512] M. Djordjevic, U.W. Heinz, Radiative energy loss in a finite dynamical QCD medium. Phys. Rev. Lett. **101**, 022302 (2008). arXiv:0802.1230 [nucl-th] 10.1103/PhysRevLett.101.022302 18764174

[513] M. Djordjevic, Collisional energy loss in a finite size QCD matter. Phys. Rev. C **74**, 064907 (2006). arXiv:nucl-th/0603066 [nucl-th]

[514] M. Djordjevic, M. Djordjevic, Generalization of radiative jet energy loss to non-zero magnetic mass. Phys. Lett. B **709**, 229–233 (2012). arXiv:1105.4359 [nucl-th]

[515] Djordjevic M, Djordjevic M (2014). LHC jet suppression of light and heavy flavor observables. Phys. Lett. B.

[516] J. Kapusta, C. Gale, *Finite-Temperature Field Theory: Principles and Applications*. Cambridge Monographs on Mathematical Physics (Cambridge University Press, Cambridge, 2006)

[517] M. Bellac, *Thermal Field Theory*. Cambridge Monographs on Mathematical Physics (Cambridge University Press, Cambridge, 2000)

[518] M. Gyulassy, P. Levai, I. Vitev, Reaction operator approach to nonAbelian energy loss. Nucl. Phys. B **594**, 371–419 (2001). arXiv:nucl-th/0006010 [nucl-th]

[519] S. Wicks, W. Horowitz, M. Djordjevic, M. Gyulassy, Elastic, inelastic, and path length fluctuations in jet tomography. Nucl. Phys. A **784**, 426–442 (2007). arXiv:nucl-th/0512076 [nucl-th]

[520] WHOT-QCD Collaboration, Y. Maezawa et al., Electric and Magnetic Screening Masses at Finite Temperature from Generalized Polyakov-Line Correlations in Two-flavor Lattice QCD. Phys. Rev. D **81**, 091501 (2010). arXiv:1003.1361 [hep-lat]

[521] WHOT-QCD Collaboration, Y. Maezawa et al., Magnetic and electric screening masses from Polyakov-loop correlations. PoS LATTICE **2008**, 194 (2008). arXiv:0811.0426 [hep-lat]

[522] A. Nakamura, T. Saito, S. Sakai, Lattice calculation of gluon screening masses. Phys. Rev. D **69**, 014506 (2004). arXiv:hep-lat/0311024 [hep-lat]

[523] A. Hart, M. Laine, O. Philipsen, Static correlation lengths in QCD at high temperatures and finite densities. Nucl. Phys. B **586**, 443–474 (2000). arXiv:hep-ph/0004060 [hep-ph]

[524] Bak D, Karch A, Yaffe LG (2007). Debye screening in strongly coupled N=4 supersymmetric Yang–Mills plasma. JHEP.

[525] Neufeld R, Vitev I (2012). Parton showers as sources of energy–momentum deposition in the QGP and their implication for shockwave formation at RHIC and at the LHC. Phys. Rev. C.

[526] Neufeld R, Vitev I, Xing H (2014). Operator definition and derivation of collisional energy and momentum loss in relativistic plasmas. Phys. Rev. D.

[527] J. Huang, Z.-B. Kang, I. Vitev, H. Xing, Photon-tagged and B-meson-tagged b-jet production at the LHC. arXiv:1505.03517 [hep-ph]

[528] Gossiaux P, Aichelin J (2008). Towards an understanding of the RHIC single electron data. Phys. Rev. C.

[529] Gossiaux PB, Bierkandt R, Aichelin J (2009). Tomography of a quark gluon plasma at RHIC and LHC energies. Phys. Rev. C.

[530] Nahrgang M, Aichelin J, Gossiaux PB, Werner K (2014). Azimuthal correlations of heavy quarks in Pb+Pb collisions at $$\sqrt{s} = 2.76$$ TeV at the CERN Large Hadron Collider. Phys. Rev. C.

[531] Svetitsky B (1988). Diffusion of charmed quarks in the quark–gluon plasma. Phys. Rev. D.

[532] Y.L. Dokshitzer, G. Marchesini, B. Webber, Dispersive approach to power behaved contributions in QCD hard processes. Nucl. Phys. B **469**, 93–142 (1996). arXiv:hep-ph/9512336 [hep-ph]

[533] Peshier A (2012). Turning on the charm. Nucl. Phys. A.

[534] Weldon HA (1982). Covariant calculations at finite temperature: the relativistic plasma. Phys. Rev. D.

[535] A. Peshier, The QCD collisional energy loss revised. Phys. Rev. Lett. 97, 212301 (2006). arXiv:hep-ph/0605294 [hep-ph] 10.1103/PhysRevLett.97.212301 17155739

[536] Peigne S, Peshier A (2008). Collisional energy loss of a fast heavy quark in a quark–gluon plasma. Phys. Rev. D.

[537] Uphoff J, Fochler O, Xu Z, Greiner C (2010). Heavy quark production at RHIC and LHC within a partonic transport model. Phys. Rev. C.

[538] Uphoff J, Fochler O, Xu Z, Greiner C (2011). Elliptic flow and energy loss of heavy quarks in ultra-relativistic heavy ion collisions. Phys. Rev. C.

[539] Uphoff J, Senzel F, Xu Z, Greiner C (2014). Momentum imbalance of D mesons in ultra-relativistic heavy-ion collisions at LHC. Phys. Rev. C.

[540] J. Uphoff, O. Fochler, Z. Xu, C. Greiner, Elastic and radiative heavy quark interactions in ultra-relativistic heavy-ion collisions, arXiv:1408.2964 [hep-ph]

[541] Xu Z, Greiner C (2005). Thermalization of gluons in ultrarelativistic heavy ion collisions by including three-body interactions in a parton cascade. Phys. Rev. C.

[542] Gossiaux P, Aichelin J, Gousset T, Guiho V (2010). Competition of heavy quark radiative and collisional energy loss in deconfined matter. J. Phys..

[543] Aichelin J, Gossiaux PB, Gousset T (2014). Gluon radiation by heavy quarks at intermediate energies. Phys. Rev. D.

[544] Gunion J, Bertsch G (1982). Hadronization by color bremsstrahlung. Phys. Rev. D.

[545] Gossiaux PB (2013). Recent results on heavy quark quenching in ultrarelativistic heavy ion collisions: the impact of coherent gluon radiation. Nucl. Phys..

[546] R. Baier, Y.L. Dokshitzer, A.H. Mueller, S. Peigne, D. Schiff, Radiative energy loss of high-energy quarks and gluons in a finite volume quark–gluon plasma. Nucl. Phys. B **483**, 291–320 (1997). arXiv:hep-ph/9607355 [hep-ph]

[547] Abir R, Greiner C, Martinez M, Mustafa MG, Uphoff J (2012). Soft gluon emission off a heavy quark revisited. Phys. Rev. D.

[548] Fochler O, Uphoff J, Xu Z, Greiner C (2013). Radiative parton processes in perturbative QCD—an improved version of the Gunion and Bertsch cross section from comparisons to the exact result. Phys. Rev. D.

[549] Fochler O, Xu Z, Greiner C (2010). Energy loss in a partonic transport model including bremsstrahlung processes. Phys. Rev. C.

[550] Ding H, Francis A, Kaczmarek O, Karsch F, Satz H (2012). Charmonium properties in hot quenched lattice QCD. Phys. Rev. D.

[551] Banerjee D, Datta S, Gavai R, Majumdar P (2012). Heavy quark momentum diffusion coefficient from lattice QCD. Phys. Rev. D.

[552] Kang Z-B, Lashof-Regas R, Ovanesyan G, Saad P, Vitev I (2015). Jet quenching phenomenology from soft-collinear effective theory with Glauber gluons. Phys. Rev. Lett..

[553] Redmer R (1997). Physical properties of dense, low-temperature plasmas. Phys. Rept..

[554] Jeukenne J, Lejeune A, Mahaux C (1976). Many body theory of nuclear matter. Phys. Rept..

[555] Brockmann R, Machleidt R (1990). Relativistic nuclear structure. 1: nuclear matter. Phys. Rev. C.

[556] M. Mannarelli, R. Rapp, Hadronic modes and quark properties in the quark–gluon plasma. Phys. Rev. C **72**, 064905 (2005). arXiv:hep-ph/0505080 [hep-ph]

[557] D. Cabrera, R. Rapp, T-matrix approach to quarkonium correlation functions in the QGP. Phys. Rev. D **76**, 114506 (2007). arXiv:hep-ph/0611134 [hep-ph]

[558] Riek F, Rapp R (2010). Quarkonia and heavy-quark relaxation times in the quark–gluon plasma. Phys. Rev. C.

[559] F. Riek, R. Rapp, Selfconsistent evaluation of charm and charmonium in the quark–gluon plasma. N. J. Phys. **13**, 045007 (2011). arXiv:1012.0019 [nucl-th]

[560] van Hees H, Mannarelli M, Greco V, Rapp R (2008). Nonperturbative heavy-quark diffusion in the quark–gluon plasma. Phys. Rev. Lett..

[561] M. He, R.J. Fries, R. Rapp, Heavy-quark diffusion and hadronization in quark–gluon plasma. Phys. Rev. C **86**, 014903 (2012). arXiv:1106.6006 [nucl-th]

[562] Eichten E, Gottfried K, Kinoshita T, Lane K, Yan T-M (1978). Charmonium: the model. Phys. Rev. D.

[563] O. Kaczmarek, Screening at finite temperature and density. PoS CPOD **07**, 043 (2007). arXiv:0710.0498 [hep-lat]

[564] Huggins K, Rapp R (2012). A T-matrix calculation for in-medium heavy-quark gluon scattering. Nucl. Phys. A.

[565] M. He, R.J. Fries, R. Rapp, Thermal relaxation of charm in hadronic matter. Phys. Lett. B **701**, 445–450 (2011). arXiv:1103.6279 [nucl-th]

[566] H. Ding, A. Francis, O. Kaczmarek, F. Karsch, H. Satz et al., Heavy quark diffusion from lattice QCD spectral functions. J. Phys. G **38**, 124070 (2011). arXiv:1107.0311 [nucl-th]

[567] J. Casalderrey-Solana, D. Teaney, Heavy quark diffusion in strongly coupled N=4 Yang–Mills. Phys. Rev. D **74**, 085012 (2006). arXiv:hep-ph/0605199 [hep-ph]

[568] Caron-Huot S, Laine M, Moore GD (2009). A way to estimate the heavy quark thermalization rate from the lattice. JHEP.

[569] D. Banerjee, S. Datta, R.V. Gavai, P. Majumdar, An estimate of heavy quark momentum diffusion coefficient in gluon plasma. PoS LATTICE **2012**, 093 (2012). arXiv:1211.2418 [hep-lat]

[570] A. Francis, O. Kaczmarek, M. Laine, J. Langelage, Towards a non-perturbative measurement of the heavy quark momentum diffusion coefficient. PoS LATTICE **2011**, 202 (2011). arXiv:1109.3941 [hep-lat]

[571] J. Casalderrey-Solana, H. Liu, D. Mateos, K. Rajagopal, U.A. Wiedemann, Gauge/string duality, hot QCD and heavy ion collisions. arXiv:1101.0618 [hep-th]

[572] DeWolfe O, Gubser SS, Rosen C, Teaney D (2014). Heavy ions and string theory. Prog. Part. Nucl. Phys..

[573] S.S. Gubser, Momentum fluctuations of heavy quarks in the gauge-string duality. Nucl. Phys. B **790**, 175–199 (2008). arXiv:hep-th/0612143 [hep-th]

[574] Son DT, Teaney D (2009). Thermal noise and stochastic strings in AdS/CFT. JHEP.

[575] P.B. Gossiaux, S. Vogel, H. van Hees, J. Aichelin, R. Rapp et al., The influence of bulk evolution models on heavy-quark phenomenology. arXiv:1102.1114 [hep-ph]

[576] M. Gyulassy, P. Levai, I. Vitev, Jet tomography of Au+Au reactions including multigluon fluctuations. Phys. Lett. B **538**, 282–288 (2002). arXiv:nucl-th/0112071 [nucl-th]

[577] ALICE Collaboration, A. Dainese, Perspectives for the study of charm in-medium quenching at the LHC with ALICE. Eur. Phys. J. C **33**, 495–503 (2004). arXiv:nucl-ex/0312005 [nucl-ex]

[578] D. de Florian, R. Sassot, M. Stratmann, Global analysis of fragmentation functions for pions and kaons and their uncertainties. Phys. Rev. D **75**, 114010 (2007). arXiv:hep-ph/0703242 [HEP-PH]

[579] M. Cacciari, P. Nason, Charm cross-sections for the Tevatron Run II. JHEP **0309**, 006 (2003). arXiv:hep-ph/0306212 [hep-ph]

[580] E. Braaten, K.-M. Cheung, S. Fleming, T.C. Yuan, Perturbative QCD fragmentation functions as a model for heavy quark fragmentation. Phys. Rev. D **51**, 4819–4829 (1995). arXiv:hep-ph/9409316 [hep-ph] 10.1103/physrevd.51.4819 10018957

[581] Kartvelishvili V, Likhoded A, Petrov V (1978). On the fragmentation functions of heavy quarks into hadrons. Phys. Lett. B.

[582] M. Djordjevic, M. Djordjevic, B. Blagojevic, RHIC and LHC jet suppression in non-central collisions. Phys. Lett. B **737**, 298–302 (2014). arXiv:1405.4250 [nucl-th]

[583] M. Djordjevic, Heavy flavor puzzle at LHC: a serendipitous interplay of jet suppression and fragmentation. Phys. Rev. Lett. **112**, 042302 (2014). arXiv:1307.4702 [nucl-th] 10.1103/PhysRevLett.112.042302 24580442

[584] P. Romatschke, U. Romatschke, Viscosity information from relativistic nuclear collisions: how perfect is the fluid observed at RHIC? Phys. Rev. Lett. **99**, 172301 (2007). arXiv:0706.1522 [nucl-th] 10.1103/PhysRevLett.99.172301 17995321

[585] Beraudo A (2014). Dynamics of heavy flavor quarks in high energy nuclear collisions. Nucl. Phys. A.

[586] Beraudo A, De Pace A, Monteno M, Nardi M, Prino F (2015). Heavy flavors in heavy-ion collisions: quenching, flow and correlations. Eur. Phys. J. C.

[587] S. Cao, G.-Y. Qin, S.A. Bass, Heavy-quark dynamics and hadronization in ultrarelativistic heavy-ion collisions: collisional versus radiative energy loss. Phys. Rev. C **88**(4), 044907 (2013). arXiv:1308.0617 [nucl-th]

[588] H. Song, U.W. Heinz, Suppression of elliptic flow in a minimally viscous quark–gluon plasma. Phys. Lett. B **658**, 279–283 (2008). arXiv:0709.0742 [nucl-th]

[589] H. Song, U.W. Heinz, Causal viscous hydrodynamics in 2+1 dimensions for relativistic heavy-ion collisions. Phys. Rev. C **77**, 064901 (2008). arXiv:0712.3715 [nucl-th]

[590] Z. Qiu, C. Shen, U. Heinz, Hydrodynamic elliptic and triangular flow in Pb–Pb collisions at $$\sqrt{s}=2.76$$ ATeV. Phys. Lett. B **707**, 151–155 (2012). arXiv:1110.3033 [nucl-th]

[591] Y. Oh, C.M. Ko, S.H. Lee, S. Yasui, Ratios of heavy baryons to heavy mesons in relativistic nucleus-nucleus collisions. Phys. Rev. C **79**, 044905 (2009). arXiv:0901.1382 [nucl-th]

[592] K. Werner, I. Karpenko, T. Pierog, M. Bleicher, K. Mikhailov, Event-by-event simulation of the three-dimensional hydrodynamic evolution from flux tube initial conditions in ultrarelativistic heavy ion collisions. Phys. Rev. C **82**, 044904 (2010). arXiv:1004.0805 [nucl-th]

[593] K. Werner, I. Karpenko, M. Bleicher, T. Pierog, S. Porteboeuf-Houssais, Jets, bulk matter, and their interaction in heavy ion collisions at several TeV. Phys. Rev. C **85**, 064907 (2012). arXiv:1203.5704 [nucl-th]

[594] K. Werner, M. Bleicher, B. Guiot, I. Karpenko, T. Pierog, Evidence for flow from hydrodynamic simulations of $${\text{ p-Pb }}$$ collisions at 5.02 TeV from $$\nu _2$$ mass splitting. Phys. Rev. Lett. **112**(23), 232301 (2014). arXiv:1307.4379 [nucl-th] 10.1103/PhysRevLett.112.232301 24972201

[595] M. Cacciari, S. Frixione, P. Nason, The p(T) spectrum in heavy flavor photoproduction. JHEP **0103**, 006 (2001). arXiv:hep-ph/0102134 [hep-ph]

[596] Nahrgang M, Aichelin J, Bass S, Gossiaux PB, Werner K (2014). Heavy-flavor observables at RHIC and LHC. Nucl. Phys. A.

[597] Wuppertal-Budapest Collaboration, S. Borsanyi et al., Is there still any Tc mystery in lattice QCD? Results with physical masses in the continuum limit III. JHEP **1009**, 073 (2010). arXiv:1005.3508 [hep-lat]

[598] Nahrgang M, Aichelin J, Bass S, Gossiaux PB, Werner K (2015). Elliptic and triangular flow of heavy flavor in heavy-ion collisions. Phys. Rev. C.

[599] Xu Z, Greiner C (2007). Transport rates and momentum isotropization of gluon matter in ultrarelativistic heavy-ion collisions. Phys. Rev. C.

[600] Peterson C, Schlatter D, Schmitt I, Zerwas PM (1983). Scaling violations in inclusive $${e^{+}e^{-}}$$ annihilation spectra. Phys. Rev. D.

[601] Uphoff J, Fochler O, Xu Z, Greiner C (2012). Open heavy flavor in Pb+Pb collisions at $$\sqrt{s}=2.76$$ TeV within a transport model. Phys. Lett. B.

[602] P.F. Kolb, J. Sollfrank, U.W. Heinz, Anisotropic transverse flow and the quark hadron phase transition. Phys. Rev. C **62**, 054909 (2000). arXiv:hep-ph/0006129 [hep-ph]

[603] M. He, R.J. Fries, R. Rapp, Ideal hydrodynamics for bulk and multistrange hadrons in $$\sqrt{s_{NN}}$$=200 AGeV Au–Au collisions. Phys. Rev. C **85**, 044911 (2012). arXiv:1112.5894 [nucl-th]

[604] P.F. Kolb, R. Rapp, Transverse flow and hadrochemistry in Au+Au collisions at $$\sqrt{s_{\text{ NN }}}$$ = 200 GeV. Phys. Rev. C 67, 044903 (2003). arXiv:hep-ph/0210222 [hep-ph]

[605] S. Pratt, Resolving the HBT puzzle in relativistic heavy ion collision. Phys. Rev. Lett. **102**, 232301 (2009). arXiv:0811.3363 [nucl-th] 10.1103/PhysRevLett.102.232301 19658926

[606] M. He, R.J. Fries, R. Rapp, Heavy flavor at the large hadron collider in a strong coupling approach. Phys. Lett. B **735**, 445–450 (2014). arXiv:1401.3817 [nucl-th]

[607] Ravagli L, Rapp R (2007). Quark coalescence based on a transport equation. Phys. Lett. B.

[608] T. Lang, H. van Hees, J. Steinheimer, M. Bleicher, Heavy quark transport in heavy ion collisions at RHIC and LHC within the UrQMD transport model. arXiv:1211.6912 [hep-ph]

[609] T. Lang, H. van Hees, J. Steinheimer, M. Bleicher, Elliptic flow and nuclear modification factors of D-mesons at FAIR in a hybrid-Langevin approach. arXiv:1305.1797 [hep-ph]

[610] T. Lang, H. van Hees, J. Steinheimer, M. Bleicher, Dileptons from correlated D- and $$\bar{\text{ D }}$$-meson decays in the invariant mass range of the QGP thermal radiation using the UrQMD hybrid model. arXiv:1305.7377 [hep-ph]

[611] H. Petersen, J. Steinheimer, G. Burau, M. Bleicher, H. Stocker, A fully integrated transport approach to heavy ion reactions with an intermediate hydrodynamic stage. Phys. Rev. C **78**, 044901 (2008). arXiv:0806.1695 [nucl-th]

[612] S. Bass, M. Belkacem, M. Bleicher, M. Brandstetter, L. Bravina et al., Microscopic models for ultrarelativistic heavy ion collisions. Prog. Part. Nucl. Phys. **41**, 255–369 (1998). arXiv:nucl-th/9803035 [nucl-th]

[613] M. Bleicher, E. Zabrodin, C. Spieles, S. Bass, C. Ernst et al., Relativistic hadron hadron collisions in the ultrarelativistic quantum molecular dynamics model. J. Phys. G **25**, 1859–1896 (1999). arXiv:hep-ph/9909407 [hep-ph]

[614] D.H. Rischke, Y. Pursun, J.A. Maruhn, Relativistic hydrodynamics for heavy ion collisions. 2. Compression of nuclear matter and the phase transition to the quark–gluon plasma. Nucl. Phys. A **595**, 383–408 (1995). arXiv:nucl-th/9504021 [nucl-th]

[615] D.H. Rischke, S. Bernard, J.A. Maruhn, Relativistic hydrodynamics for heavy ion collisions. 1. General aspects and expansion into vacuum. Nucl. Phys. A **595**, 346–382 (1995). arXiv:nucl-th/9504018 [nucl-th]

[616] J. Steinheimer, M. Bleicher, H. Petersen, S. Schramm, H. Stocker et al., (3+1)-dimensional hydrodynamic expansion with a critical point from realistic initial conditions. Phys. Rev. C **77**, 034901 (2008). arXiv:0710.0332 [nucl-th]

[617] Cooper F, Frye G (1974). Comment on the single particle distribution in the hydrodynamic and statistical thermodynamic models of multiparticle production. Phys. Rev. D.

[618] M. Golam Mustafa, D. Pal, D. Kumar Srivastava, Propagation of charm quarks in equilibrating quark–gluon plasma. Phys. Rev. C **57**, 889–898 (1998). arXiv:nucl-th/9706001 [nucl-th]

[619] P.B. Gossiaux, V. Guiho, J. Aichelin, Charmonia enhancement in quark–gluon plasma with improved description of c-quarks phase-distribution. J. Phys. G **31**, S1079–S1082 (2005). arXiv:hep-ph/0411324 [hep-ph]

[620] H. van Hees, R. Rapp, Thermalization of heavy quarks in the quark–gluon plasma. Phys. Rev. C **71**, 034907 (2005). arXiv:nucl-th/0412015 [nucl-th]

[621] G.D. Moore, D. Teaney, How much do heavy quarks thermalize in a heavy ion collision? Phys. Rev. C **71**, 064904 (2005). arXiv:hep-ph/0412346 [hep-ph]

[622] H. van Hees, V. Greco, R. Rapp, Heavy-quark probes of the quark–gluon plasma at RHIC. Phys. Rev. C **73**, 034913 (2006). arXiv:nucl-th/0508055 [nucl-th] 10.1103/PhysRevLett.100.192301 18518442

[623] M. He, H. van Hees, P.B. Gossiaux, R.J. Fries, R. Rapp, Relativistic Langevin Dynamics in Expanding Media. Phys. Rev. E 88, 032138 (2013). arXiv:1305.1425 [nucl-th] 10.1103/PhysRevE.88.032138 24125244

[624] W. Horowitz, M. Gyulassy, Heavy quark jet tomography of Pb+Pb at LHC: AdS/CFT drag or pQCD energy loss? Phys. Lett. B **666**, 320–323 (2008). arXiv:0706.2336 [nucl-th]

[625] Horowitz W (2012). Testing pQCD and AdS/CFT energy loss at RHIC and LHC. AIP Conf. Proc..

[626] ALICE Collaboration, K. Aamodt et al., Centrality dependence of the charged-particle multiplicity density at mid-rapidity in Pb–Pb collisions at $$\sqrt{s_{NN}}=2.76$$ TeV. Phys. Rev. Lett. 106, 032301 (2011). arXiv:1012.1657 [nucl-ex] 10.1103/PhysRevLett.106.032301 21405267

[627] Akamatsu Y, Hatsuda T, Hirano T (2009). Heavy quark diffusion with relativistic langevin dynamics in the quark–gluon fluid. Phys. Rev. C.

[628] S. Cao, S.A. Bass, Thermalization of charm quarks in infinite and finite QGP matter. Phys. Rev. C **84**, 064902 (2011). arXiv:1108.5101 [nucl-th]

[629] BRAHMS Collaboration, I. Arsene et al., Quark gluon plasma and color glass condensate at RHIC? The perspective from the BRAHMS experiment. Nucl. Phys. A **757**, 1–27 (2005). arXiv:nucl-ex/0410020 [nucl-ex]

[630] PHENIX Collaboration, K. Adcox et al., Formation of dense partonic matter in relativistic nucleus–nucleus collisions at RHIC: experimental evaluation by the PHENIX collaboration. Nucl. Phys. A **757**, 184–283 (2005). arXiv:nucl-ex/0410003 [nucl-ex]

[631] B. Back, M. Baker, M. Ballintijn, D. Barton, B. Becker et al., The PHOBOS perspective on discoveries at RHIC. Nucl. Phys. A **757**, 28–101 (2005). arXiv:nucl-ex/0410022 [nucl-ex]

[632] STAR Collaboration, J. Adams et al., Experimental and theoretical challenges in the search for the quark gluon plasma: the STAR Collaboration’s critical assessment of the evidence from RHIC collisions. Nucl. Phys. A **757**, 102–183 (2005). arXiv:nucl-ex/0501009 [nucl-ex]

[633] CMS Collaboration, S. Chatrchyan et al., Long-range and short-range dihadron angular correlations in central $$\text{ Pb--Pb }$$ collisions at a nucleon–nucleon center of mass energy of 2.76 TeV. JHEP **1107**, 076 (2011). arXiv:1105.2438 [nucl-ex]

[634] ALICE Collaboration, K. Aamodt et al., Particle-yield modification in jet-like azimuthal di-hadron correlations in Pb–Pb collisions at $$\sqrt{s_{NN}} = 2.76$$ TeV. Phys. Rev. Lett. **108**, 092301 (2012). arXiv:1110.0121 [nucl-ex] 10.1103/PhysRevLett.108.092301 22463626

[635] T. Renk, K. Eskola, Prospects of medium tomography using back-to-back hadron correlations. Phys. Rev. C **75**, 054910 (2007). arXiv:hep-ph/0610059 [hep-ph]

[636] S. Cao, G.-Y. Qin, S.A. Bass, Dynamical evolution, hadronization and angular de-correlation of heavy flavor in a hot and dense QCD medium. Nucl. Phys. A **932**, 38–44 (2014). arXiv:1404.1081 [nucl-th]

[637] Renk T (2014). Charm energy loss and D-D correlations from a shower picture. Phys. Rev. C.

[638] ALICE Collaboration, F. Colamaria, Measurement of azimuthal correlations between D mesons and charged hadrons with ALICE at the LHC. EPJ Web Conf. **80**, 00034 (2014). arXiv:1408.6038 [hep-ex]

[639] STAR Collaboration, G. Wang, Non-photonic electron–hadron correlations at STAR. J. Phys. G **35**, 104107 (2008). arXiv:0804.4448 [nucl-ex]

[640] ALICE Collaboration, D. Thomas, Measurement of electrons from heavy-flavour decays in Pb–Pb collisions at $$\sqrt{s_{NN}}=2.76$$ TeV with ALICE. J. Phys. Conf. Ser. **509**, 012079 (2014)

[641] Ozvenchuk V, Torres-Rincon JM, Gossiaux PB, Tolos L, Aichelin J (2014). D-meson propagation in hadronic matter and consequences for heavy-flavor observables in ultrarelativistic heavy-ion collisions. Phys. Rev. C.

[642] S. Cao, G.-Y. Qin, S.A. Bass, Heavy flavor dynamics in QGP and hadron gas. Nucl. Phys. A **931**, 569–574 (2014). arXiv:1408.0503 [nucl-th]

[643] X. Zhu, N. Xu, P. Zhuang, The effect of partonic wind on charm quark correlations in high-energy nuclear collisions. Phys. Rev. Lett. **100**, 152301 (2008). arXiv:0709.0157 [nucl-th] 10.1103/PhysRevLett.100.152301 18518098

[644] Matsui T, Satz H (1986). $$\text{ J }/\psi $$ suppression by quark–gluon plasma formation. Phys. Lett. B.

[645] S. Digal, P. Petreczky, H. Satz, Quarkonium feed down and sequential suppression. Phys. Rev. D **64**, 094015 (2001). arXiv:hep-ph/0106017 [hep-ph]

[646] R.L. Thews, M. Schroedter, J. Rafelski, Enhanced $$\text{ J }/\psi $$ production in deconfined quark matter. Phys. Rev. C **63**, 054905 (2001). arXiv:hep-ph/0007323 [hep-ph]

[647] P. Braun-Munzinger, J. Stachel, (Non)thermal aspects of charmonium production and a new look at $${\text{ J }}/\psi $$ suppression. Phys. Lett. B **490**, 196–202 (2000). arXiv:nucl-th/0007059 [nucl-th]

[648] J. Stachel, A. Andronic, P. Braun-Munzinger, K. Redlich, Confronting LHC data with the statistical hadronization model. J. Phys. Conf. Ser. **509**, 012019 (2014). arXiv:1311.4662 [nucl-th]

[649] PHENIX Collaboration, S. Afanasiev et al., Measurement of direct photons in $$\text{ Au--Au }$$. Phys. Rev. Lett. **109**, 152302 (2012). arXiv:1205.5759 [nucl-ex] 10.1103/PhysRevLett.109.152302 23102300

[650] CMS Collaboration, S. Chatrchyan et al., Measurement of isolated photon production in $${{\text{ pp }}}$$. Phys. Lett. B **710**, 256–277 (2012). arXiv:1201.3093 [nucl-ex]

[651] CMS Collaboration, S. Chatrchyan et al., Study of W boson production in $${\text{ Pb--Pb }}$$, Phys. Lett. B **715**, 66–87 (2012). arXiv:1205.6334 [nucl-ex]

[652] ATLAS Collaboration, G. Aad et al., Measurement of Z boson production in $${\text{ Pb-Pb }}$$ with the ATLAS detector. Phys. Rev. Lett. **110**, 022301 (2013). arXiv:1210.6486 [hep-ex] 10.1103/PhysRevLett.110.022301 23383894

[653] CMS Collaboration, S. Chatrchyan et al., Study of Z production in $${\text{ Pb-Pb }}$$ in the dimuon and dielectron decay channels. JHEP **03**, 022 (2015). arXiv:1410.4825 [nucl-ex]

[654] Zhao X, Rapp R (2008). Transverse momentum spectra of $$\text{ J }/\psi $$ in heavy-ion collisions. Phys. Lett. B.

[655] X. Zhao, R. Rapp, Forward and midrapidity charmonium production at RHIC. Eur. Phys. J. C **62**, 109–117 (2009). arXiv:0810.4566 [nucl-th]

[656] NA38 Collaboration, M. Abreu et al., $$J/\psi , \psi \prime $$ and Drell-Yan production in S–U interactions at 200 GeV per nucleon. Phys. Lett. B **449**, 128–136 (1999)

[657] NA50 Collaboration, M. Abreu et al., $${\text{ J }}/\psi $$ per nucleon. Phys. Lett. B **410**, 327–336 (1997)

[658] NA50 Collaboration, M. Abreu et al., Anomalous $${\text{ J }}/\psi $$ per nucleon. Phys. Lett. B **410**, 337–343 (1997)

[659] NA50 Collaboration, M. Abreu et al., Observation of a threshold effect in the anomalous $${\text{ J }}/\psi $$ suppression. Phys. Lett. B **450**, 456–466 (1999)

[660] NA50 Collaboration, M. Abreu et al., Evidence for deconfinement of quarks and gluons from the $${\text{ J }}/\psi $$ collisions at the CERN SPS. Phys. Lett. B **477**, 28–36 (2000)

[661] NA50 Collaboration, M. Abreu et al., Transverse momentum distributions of $${\text{ J }}/\psi $$ interactions at the SPS. Phys. Lett. B **499**, 85–96 (2001)

[662] NA50 Collaboration, M. Abreu et al., The dependence of the anomalous $${\text{ J }}/\psi $$ suppression on the number of participant nucleons. Phys. Lett. B **521**, 195–203 (2001)

[663] NA50 Collaboration, B. Alessandro et al., A new measurement of $${\text{ J }}/\psi $$ per nucleon. Eur. Phys. J. C **39**, 335 (2005). arXiv:hep-ex/0412036

[664] NA50 Collaboration, B. Alessandro et al., $${\psi \text{(2S) }}$$ collisions at 158 GeV/nucleon. Eur. Phys. J. C **49**, 559–567 (2007). arXiv:nucl-ex/0612013 [nucl-ex]

[665] NA60 Collaboration, R. Arnaldi et al., $${\text{ J }}/\psi $$/nucleon. Phys. Rev. Lett. **99**, 132302 (2007)

[666] PHENIX Collaboration, S. Adler et al., $${\text{ J }}/\psi $$, Phys. Rev. C **69**, 014901 (2004). arXiv:nucl-ex/0305030 [nucl-ex]

[667] PHENIX Collaboration, A. Adare et al., $${\text{ J }}/\psi $$. Phys. Rev. Lett. **98**, 232301 (2007). arXiv:nucl-ex/0611020

[668] PHENIX Collaboration, A. Adare et al., $${\text{ J }}/\psi $$. Phys. Rev. C **84**, 054912 (2011). arXiv:1103.6269 [nucl-ex]

[669] PHENIX Collaboration, C. Silvestre, PHENIX first measurement of the $${\text{ J }}/\psi $$. J. Phys. G **35**, 104136 (2008). arXiv:0806.0475 [nucl-ex]

[670] PHENIX Collaboration, E.T. Atomssa, $${\text{ J }}/\psi $$ collisions by the PHENIX experiment. Nucl. Phys. A **830**, 331C–334C (2009). arXiv:0907.4787 [nucl-ex]

[671] PHENIX Collaboration, A. Adare et al., $${\text{ J }}/\psi $$ collisions. Phys. Rev. Lett. **101**, 122301 (2008). arXiv:0801.0220 [nucl-ex]

[672] PHENIX Collaboration, A. Adare, Nuclear matter effects on $${\text{ J }}/\psi $$. Phys. Rev. C **90**, 064908 (2014). arXiv:1404.1873 [nucl-ex]

[673] PHENIX Collaboration, C.L. da Silva, $$\text{ J }/\psi $$ collisions. Nucl. Phys. A **931**, 638–642 (2014)

[674] PHENIX Collaboration, A. Adare et al., $$\text{ J }/\psi $$. Phys. Rev. C **86**, 064901 (2012). arXiv:1208.2251 [nucl-ex]

[675] PHENIX Collaboration, A. Adare et al., Measurement of $${\Upsilon \text{(1S+2S+3S) }}$$. arXiv:1404.2246 [nucl-ex] (accepted by Phys. Rev. C)

[676] STAR Collaboration, L. Adamczyk et al., $$\text{ J }/\psi $$ = 200 GeV with the STARdetector. Phys. Rev. C **90**, 024906 (2014). arXiv:1310.3563 [nucl-ex]

[677] STAR Collaboration, L. Adamczyk et al., Measurement of $$\text{ J }/\psi $$ azimuthal anisotropy in $$\text{ Au-Au }$$ collisions at $$\sqrt{s_{\text{ NN }}}$$ = 200$${\text{ GeV }}$$. Phys. Rev. Lett. **111**, 052301 (2013). arXiv:1212.3304 [nucl-ex] 10.1103/PhysRevLett.111.052301 23952389

[678] STAR Collaboration, W. Zha, Recent measurements of quarkonium production in $${\text{ pp }}$$ and $$\text{ AA }$$ collisions from the STAR experiment. Nucl. Phys. A **931**, 596–600 (2014)

[679] ALICE Collaboration, B. B. Abelev et al., Centrality, rapidity and transverse momentum dependence of $$\text{ J }/\psi $$. Phys. Lett. B **734**, 314–327 (2014). arXiv:1311.0214 [nucl-ex]

[680] ALICE Collaboration, B. Abelev et al., $$\text{ J }/\psi $$. Phys. Rev. Lett. **109**, 072301 (2012). arXiv:1202.1383 [hep-ex]

[681] ALICE Collaboration, E. Abbas et al., $$\text{ J }/\psi $$. Phys. Rev. Lett. **111**, 162301 (2013). arXiv:1303.5880 [nucl-ex]

[682] ALICE Collaboration, R. Arnaldi, $$\text{ J }/\psi $$ collisions with the ALICE muon spectrometer at the LHC. Nucl. Phys. A **904–905**, 595c–598c (2013). arXiv:1211.2578 [nucl-ex]

[683] ALICE Collaboration, B. B. Abelev et al., Suppression of $$\Upsilon \text{(1S) }$$. Phys. Lett. B **738**, 361–372 (2014). arXiv:1405.4493 [nucl-ex]

[684] ATLAS Collaboration, G. Aad et al., Measurement of the centrality dependence of $$\text{ J }/\psi $$ yields and observation of Z production in lead–lead collisions with the ATLAS detector at the LHC. Phys. Lett. B **697**, 294–312 (2011). arXiv:1012.5419 [hep-ex]

[685] CMS Collaboration, Measurement of the azimuthal anisotropy of prompt and non-prompt $$\text{ J }/\psi $$. CMS physics analysis summary CMS-PAS-HIN-12-001 (2013). https://cds.cern.ch/record/1626596?ln=en

[686] CMS Collaboration, V. Khachatryan et al., Measurement of prompt $${\psi \text{(2S) }}$$ to $$\text{ J }/\psi $$ yield ratios in $${\text{ Pb--Pb }}$$ and $${\text{ pp }}$$ collisions at $$\sqrt{s_{\text{ NN }}}$$ = 2.76 TeV. Phys. Rev. Lett. **113**, 262301 (2014). arXiv:1410.1804 [nucl-ex] 10.1103/PhysRevLett.113.262301 25615312

[687] CMS Collaboration, S. Chatrchyan et al., Indications of suppression of excited $${\Upsilon }$$ states in $${\text{ Pb--Pb }}$$ collisions at $$\sqrt{s_{\text{ NN }}}$$ = 2.76$${\text{ TeV }}$$. Phys. Rev. Lett. **107**, 052302 (2011). arXiv:1105.4894 [nucl-ex] 10.1103/PhysRevLett.107.052302 21867063

[688] CMS Collaboration, S. Chatrchyan et al., Observation of sequential $${\Upsilon }$$ suppression in $${\text{ Pb--Pb }}$$ collisions. Phys. Rev. Lett. **109**, 222301 (2012). arXiv:1208.2826 [nucl-ex] 10.1103/PhysRevLett.109.222301 23368113

[689] Brambilla N, Escobedo MA, Ghiglieri J, Soto J, Vairo A (2010). Heavy quarkonium in a weakly-coupled quark-gluon plasma below the melting temperature. JHEP.

[690] Quarkonium Working Group Collaboration, N. Brambilla et al., Heavy quarkonium physics. arXiv:hep-ph/0412158 [hep-ph]

[691] N. Brambilla, A. Pineda, J. Soto, A. Vairo, Effective field theories for heavy quarkonium. Rev. Mod. Phys. **77**, 1423 (2005). arXiv:hep-ph/0410047 [hep-ph]

[692] Brambilla N (2014). QCD and strongly coupled gauge theories: challenges and perspectives. Eur. Phys. J. C.

[693] M. Asakawa, T. Hatsuda, Y. Nakahara, Maximum entropy analysis of the spectral functions in lattice QCD. Prog. Part. Nucl. Phys. **46**, 459–508 (2001). arXiv:hep-lat/0011040 [hep-lat]

[694] A. Rothkopf, Improved maximum entropy analysis with an extended search space. J. Comput. Phys. **238**, 106–114 (2013). arXiv:1110.6285 [physics.comp-ph]

[695] Burnier Y, Rothkopf A (2013). Bayesian approach to spectral function reconstruction for euclidean quantum field theories. Phys. Rev. Lett..

[696] S. Kim, P. Petreczky, A. Rothkopf, Lattice NRQCD study of S- and P-wave bottomonium states in a thermal medium with $$N_f=2+1$$ light flavors. arXiv:1409.3630 [hep-lat]

[697] Aarts G, Allton C, Oktay MB, Peardon M, Skullerud J-I (2007). Charmonium at high temperature in two-flavor QCD. Phys. Rev. D.

[698] A. Kelly, J.-I. Skullerud, C. Allton, D. Mehta, M. B. Oktay, Spectral functions of charmonium from 2 flavour anisotropic lattice data, PoS LATTICE2013, p. 170 (2014). arXiv:1312.0791 [hep-lat]

[699] E705 Collaboration, L. Antoniazzi et al., Measurement of $$\text{ J }/\psi $$ interactions with nuclei. Phys. Rev. D **46**, 4828–4835 (1992) 10.1103/physrevd.46.4828 10014861

[700] E705 Collaboration, L. Antoniazzi et al., Production of $$\text{ J }/\psi $$-nucleon interactions. Phys. Rev. Lett. **70**, 383–386 (1993) 10.1103/PhysRevLett.70.383 10054098

[701] Karsch F, Kharzeev D, Satz H (2006). Sequential charmonium dissociation. Phys. Lett. B.

[702] Aarts G, Allton C, Kim S, Lombardo M, Ryan S (2013). Melting of P wave bottomonium states in the quark-gluon plasma from lattice NRQCD. JHEP.

[703] Aarts G, Allton C, Kim S, Lombardo M, Oktay M (2011). What happens to the $${\Upsilon }$$ and $$\eta _{b}$$ in the quark-gluon plasma? Bottomonium spectral functions from lattice QCD. JHEP.

[704] Aarts G, Kim S, Lombardo M, Oktay M, Ryan S (2011). Bottomonium above deconfinement in lattice nonrelativistic QCD. Phys. Rev. Lett..

[705] Aarts G, Allton C, Harris T, Kim S, Lombardo MP (2014). The bottomonium spectrum at finite temperature from N$$_{f}$$ = 2 + 1 lattice QCD. JHEP.

[706] Rakotozafindrabe A, Ferreiro EG, Fleuret F, Lansberg JP, Matagne N (2011). Cold nuclear matter effects on extrinsic $$\text{ J }/\psi $$ production at $$\sqrt{s_{\text{ NN }}}$$ = 2.76$${\text{ TeV }}$$ at the LHC. Nucl. Phys. A.

[707] R. Vogt, In progress (2015) (in progress)

[708] A. Andronic, P. Braun-Munzinger, J. Stachel, Thermal hadron production in relativistic nuclear collisions: the Hadron mass spectrum, the horn, and the QCD phase transition. Phys. Lett. B **673**, 142–145 (2009). arXiv:0812.1186 [nucl-th]

[709] P. Braun-Munzinger, K. Redlich, Charmonium production from the secondary collisions at LHC energy. Eur. Phys. J. C **16**, 519–525 (2000). arXiv:hep-ph/0001008 [hep-ph]

[710] P. Braun-Munzinger, J. Stachel, On charm production near the phase boundary. Nucl. Phys. A **690**, 119–126 (2001). arXiv:nucl-th/0012064 [nucl-th]

[711] P. Braun-Munzinger, J. Stachel, Charmonium from statistical hadronization of heavy quarks: a probe for deconfinement in the quark-gluon plasma. arXiv:0901.2500 [nucl-th]

[712] A. Andronic, P. Braun-Munzinger, K. Redlich, J. Stachel, Statistical hadronization of heavy quarks in ultra-relativistic nucleus–nucleus collisions. Nucl. Phys. A **789**, 334–356 (2007). arXiv:nucl-th/0611023 [nucl-th]

[713] A. Andronic, P. Braun-Munzinger, K. Redlich, J. Stachel, Evidence for charmonium generation at the phase boundary in ultra-relativistic nuclear collisions. Phys. Lett. B **652**, 259–261 (2007). arXiv:nucl-th/0701079 [NUCL-TH]

[714] A. Andronic, P. Braun-Munzinger, K. Redlich, J. Stachel, Statistical hadronization of charm: from FAIR to the LHC. J. Phys. G **35**, 104155 (2008). arXiv:0805.4781 [nucl-th]

[715] L. Grandchamp, R. Rapp, G.E. Brown, In medium effects on charmonium production in heavy ion collisions. Phys. Rev. Lett. **92**, 212301 (2004). arXiv:hep-ph/0306077 [hep-ph] 10.1103/PhysRevLett.92.212301 15245274

[716] L. Grandchamp, S. Lumpkins, D. Sun, H. van Hees, R. Rapp, Bottomonium production at $$\sqrt{s_{\text{ NN }}}$$ = 200$${\text{ GeV }}$$ and $$\sqrt{s_{\text{ NN }}}$$ = 5.5$${\text{ TeV }}$$. Phys. Rev. C **73**, 064906 (2006). arXiv:hep-ph/0507314 [hep-ph]

[717] L. Grandchamp, R. Rapp, Thermal versus direct $$\text{ J }/\psi $$ production in ultrarelativistic heavy-ion collisions. Phys. Lett. B **523**, 60–66 (2001). arXiv:hep-ph/0103124 [hep-ph]

[718] Bhanot G, Peskin ME (1979). Short distance analysis for heavy quark systems: 2. Appl. Nucl. Phys..

[719] Brambilla N, Escobedo MA, Ghiglieri J, Vairo A (2013). Thermal width and quarkonium dissociation by inelastic parton scattering. JHEP.

[720] L. Grandchamp, R. Rapp, Charmonium suppression and regeneration from SPS to RHIC. Nucl. Phys. A **709**, 415–439 (2002). arXiv:hep-ph/0205305 [hep-ph]

[721] T. Song, K.C. Han, C.M. Ko, Charmonium production from nonequilibrium charm and anticharm quarks in quark-gluon plasma. Phys. Rev. C **85**, 054905 (2012). arXiv:1203.2964 [nucl-th]

[722] S. Hamieh, K. Redlich, A. Tounsi, Canonical description of strangeness enhancement from p-A to $${\text{ Pb--Pb }}$$ collisions. Phys. Lett. B **486**, 61–66 (2000). arXiv:hep-ph/0006024 [hep-ph]

[723] Zhao X, Rapp R (2010). Charmonium in medium: from correlators to experiment. Phys. Rev. C.

[724] X. Zhao, Charmonium in hot medium. PhD thesis, Texas A&M University (2010). arXiv:1203.2572 [nucl-th]

[725] E. Schnedermann, J. Sollfrank, U. W. Heinz, Thermal phenomenology of hadrons from 200 $$A{{\text{ GeV }}}$$ collisions. Phys. Rev. C **48**, 2462–2475 (1993). arXiv:nucl-th/9307020 [nucl-th] 10.1103/physrevc.48.2462 9969103

[726] X. Zhao, R. Rapp, Charmonium production at high $${p_{\text{ T }}}$$ at RHIC. arXiv:0806.1239 [nucl-th]

[727] H. van Hees, M. He, R. Rapp, Pseudo-critical enhancement of thermal photons in relativistic heavy-ion collisions. arXiv:1404.2846 [nucl-th] (submitted to Nucl. Phys. A)

[728] Zhao X, Rapp R (2011). Medium modifications and production of charmonia at LHC. Nucl. Phys. A.

[729] Emerick A, Zhao X, Rapp R (2012). Bottomonia in the quark-gluon plasma and their production at RHIC and LHC. Eur. Phys. J. A.

[730] L. Yan, P. Zhuang, N. Xu, $$\text{ J }/\psi $$ production in quark-gluon plasma. Phys. Rev. Lett. **97**, 232301 (2006). arXiv:nucl-th/0608010 [nucl-th] 10.1103/PhysRevLett.97.232301 17280196

[731] Y.-P. Liu, Z. Qu, N. Xu, P.-F. Zhuang, $$\text{ J }/\psi $$ transverse momentum distribution in high energy nuclear collisions at RHIC. Phys. Lett. B **678**, 72 (2009). arXiv:0901.2757 [nucl-th]

[732] K. Zhou, N. Xu, P. Zhuang, Transverse momentum distribution as a probe of $$\text{ J }/\psi $$ production mechanism in heavy ion collisions. Nucl. Phys. A **834**, 249C–252C (2010). arXiv:0911.5008 [nucl-th]

[733] K. Zhou, N. Xu, Z. Xu, P. Zhuang, Medium effects on charmonium production at ultrarelativistic energies available at the CERN large haron collider. Phys. Rev. C **89**, 054911 (2014). arXiv:1401.5845 [nucl-th]

[734] Attems M, Rebhan A, Strickland M (2013). Instabilities of an anisotropically expanding non-Abelian plasma: 3D+3V discretized hard-loop simulations. Phys. Rev. D.

[735] Berges J, Boguslavski K, Schlichting S (2012). Nonlinear amplification of instabilities with longitudinal expansion. Phys. Rev. D.

[736] Heller MP, Janik RA, Witaszczyk P (2012). A numerical relativity approach to the initial value problem in asymptotically anti-de Sitter spacetime for plasma thermalization—an ADM formulation. Phys. Rev. D.

[737] van der Schee W, Romatschke P, Pratt S (2013). Fully dynamical simulation of central nuclear collisions. Phys. Rev. Lett..

[738] W. Florkowski, R. Ryblewski, Highly-anisotropic and strongly-dissipative hydrodynamics for early stages of relativistic heavy-ion collisions. Phys. Rev. C **83**, 034907 (2011). arXiv:1007.0130 [nucl-th]

[739] M. Martinez, M. Strickland, Dissipative dynamics of highly anisotropic systems. Nucl. Phys. A **848**, 183 (2010). arXiv:1007.0889 [nucl-th]

[740] M. Martinez, R. Ryblewski, M. Strickland, Boost-invariant (2+1)-dimensional anisotropic hydrodynamics. Phys. Rev. C **85**, 064913 (2012). arXiv:1204.1473 [nucl-th]

[741] D. Bazow, U.W. Heinz, M. Strickland, Second-order (2+1)-dimensional anisotropic hydrodynamics. Phys. Rev. C **90**, 054910 (2014). arXiv:1311.6720 [nucl-th]

[742] Dumitru A, Guo Y, Strickland M (2008). The heavy-quark potential in an anisotropic (viscous) plasma. Phys. Lett. B.

[743] Dumitru A, Guo Y, Mocsy A, Strickland M (2009). Quarkonium states in an anisotropic QCD plasma. Phys. Rev. D.

[744] Burnier Y, Laine M, Vepsalainen M (2009). Quarkonium dissociation in the presence of a small momentum space anisotropy. Phys. Lett. B.

[745] Dumitru A, Guo Y, Strickland M (2009). The imaginary part of the static gluon propagator in an anisotropic (viscous) QCD plasma. Phys. Rev. D.

[746] Margotta M, McCarty K, McGahan C, Strickland M, Yager-Elorriaga D (2011). Quarkonium states in a complex-valued potential. Phys. Rev. D.

[747] Strickland M (2011). Thermal $$\Upsilon \text{(1S) }$$ and $$\chi _{b1}$$ suppression in $$\sqrt{s_{\text{ NN }}}$$=2.76 TeV $$\text{ Pb-Pb }$$ collisions at the LHC. Phys. Rev. Lett..

[748] M. Strickland, D. Bazow, Thermal bottomonium suppression at RHIC and LHC. Nucl. Phys. A **879**, 25 (2012). arXiv:1112.2761 [nucl-th]

[749] M. Strickland, Bottomonia in the quark gluon plasma. J. Phys. Conf. Ser. **432**, 012015 (2013). arXiv:1210.7512 [nucl-th]

[750] Schenke B, Jeon S, Gale C (2011). Anisotropic flow in $${\sqrt{s}}$$ = 2.76$${\text{ TeV }}$$$${\text{ Pb-Pb }}$$ collisions at the LHC. Phys. Lett. B.

[751] A. Capella, E. Ferreiro, A. Kaidalov, Nonsaturation of the $$\text{ J }/\psi $$ suppression at large transverse energy in the comovers approach. Phys. Rev. Lett. **85**, 2080–2083 (2000). arXiv:hep-ph/0002300 [hep-ph] 10.1103/PhysRevLett.85.2080 10970467

[752] Capella A, Bravina L, Ferreiro E, Kaidalov A, Tywoniuk K (2008). Charmonium dissociation and recombination at RHIC and LHC. Eur. Phys. J. C.

[753] Ferreiro E (2014). Charmonium dissociation and recombination at LHC: revisiting comovers. Phys. Lett. B.

[754] A. Andronic, P. Braun-Munzinger, K. Redlich, J. Stachel, The thermal model on the verge of the ultimate test: particle production in $${\text{ Pb--Pb }}$$ collisions at the LHC. J. Phys. G **38**, 124081 (2011). arXiv:1106.6321 [nucl-th]

[755] ALICE Collaboration, J. Book, $$\text{ J }/\psi $$. Nucl. Phys. A **931**, 591–595 (2014)

[756] Zhao X, Emerick A, Rapp R (2013). In-medium quarkonia at SPS. RHIC and LHC. Nucl. Phys..

[757] Y. Liu, N. Xu, P. Zhuang, $$\text{ J }/\psi $$ elliptic flow in relativistic heavy ion collisions. Nucl. Phys. A **834**, 317C–319C (2010). arXiv:0910.0959 [nucl-th]

[758] CMS Collaboration, S. Chatrchyan et al., Azimuthal anisotropy of charged particles at high transverse momenta in $${\text{ Pb--Pb }}$$ collisions at $$\sqrt{s_{\text{ NN }}}$$ = 2.76$${\text{ TeV }}$$. Phys. Rev. Lett. **109**, 022301 (2012). arXiv:1204.1850 [nucl-ex] 10.1103/PhysRevLett.109.022301 23030154

[759] X. Du, R. Rapp, Sequential regeneration of charmonia in heavy-ion collisions. arXiv:1504.00670 [hep-ph]

[760] A. Andronic, Experimental results and phenomenology of quarkonium production in relativistic nuclear collisions. Nucl. Phys. A **931**, 135–144 (2014). arXiv:1409.5778 [nucl-ex]

[761] Strickland M (2013). Thermal bottomonium suppression. AIP Conf. Proc..

[762] Satz H, Sridhar K (1994). Charmonium production versus open charm in nuclear collisions. Phys. Rev. D.

[763] Satz H (2013). Calibrating the in-medium behavior of quarkonia. Adv. High Energy Phys..

[764] ALICE Collaboration, J. Adam et al., Coherent $$\rho ^0$$ TeV. arXiv:1503.09177 [nucl-ex]

[765] G. Baur, K. Hencken, D. Trautmann, Photon–photon and photon–hadron interactions at relativistic heavy ion colliders. Prog. Part. Nucl. Phys. **42**, 357–366 (1999). arXiv:nucl-th/9810078 [nucl-th]

[766] G. Baur, K. Hencken, D. Trautmann, S. Sadovsky, Y. Kharlov, Coherent gamma gamma and gamma-A interactions in very peripheral collisions at relativistic ion colliders. Phys. Rep. **364**, 359–450 (2002). arXiv:hep-ph/0112211 [hep-ph]

[767] C. A. Bertulani, S. R. Klein, J. Nystrand, Physics of ultra-peripheral nuclear collisions. Ann. Rev. Nucl. Part. Sci. **55**, 271–310 (2005). arXiv:nucl-ex/0502005 [nucl-ex]

[768] A. Baltz, G. Baur, D. d’Enterria, L. Frankfurt, F. Gelis, et al., The physics of ultraperipheral collisions at the LHC. Phys. Rep. **458**, 1–171 (2008). arXiv:0706.3356 [nucl-ex]

[769] Fermi E (1924). On the theory of the impact between atoms and electrically charged particles. Z. Phys..

[770] E. Fermi, On the theory of collisions between atoms and electrically charged particles. Nuovo Cim. **2**, 143–158 (1925). arXiv:hep-th/0205086 [hep-th]

[771] von Weizsacker C (1934). Radiation emitted in collisions of very fast electrons. Z. Phys..

[772] Williams E (1934). Nature of the high-energy particles of penetrating radiation and status of ionization and radiation formulae. Phys. Rev..

[773] S. Klein, J. Nystrand, Exclusive vector meson production in relativistic heavy ion collisions. Phys. Rev. C **60**, 014903 (1999). arXiv:hep-ph/9902259 [hep-ph] 10.1103/PhysRevLett.84.2330 11018877

[774] ALICE Collaboration, B. Abelev et al., Centrality determination of Pb–Pb collisions at $$\sqrt{s_{NN}}$$ = 2.76 TeV with ALICE. Phys. Rev. C **88**(4), 044909 (2013). arXiv:1301.4361 [nucl-ex]

[775] PHENIX Collaboration, S. Afanasiev et al., Photoproduction of $$\text{ J }/\psi $$ = 200 GeV. Phys. Lett. B **679**, 321–329 (2009). arXiv:0903.2041 [nucl-ex]

[776] A.J. Baltz, S.R. Klein, J. Nystrand, Coherent vector meson photoproduction with nuclear breakup in relativistic heavy ion collisions. Phys. Rev. Lett. **89**, 012301 (2002). arXiv:nucl-th/0205031 [nucl-th] 10.1103/PhysRevLett.89.012301 12097035

[777] M. Strikman, M. Tverskoy, M. Zhalov, Neutron tagging of quasielastic $${\text{ J }}/\psi $$ photoproduction off nucleus in ultraperipheral heavy ion collisions at RHIC energies. Phys. Lett. B **626**, 72–79 (2005). arXiv:hep-ph/0505023 [hep-ph]

[778] Armesto N, Borghini N, Jeon S, Wiedemann UA, Abreu S, Akkelin V, Alam J, Albacete JL, Andronic A, Antonov D (2008). Heavy ion collisions at the LHC—last call for predictions. J. Phys..

[779] ALICE Collaboration, E. Abbas et al., Charmonium and $${e^{+}e^{-}}$$ pair photoproduction at mid-rapidity in ultra-peripheral Pb–Pb collisions at $$\sqrt{s_{NN}}$$ = 2.76 TeV. Eur. Phys. J. C **73**, 2617 (2013). arXiv:1305.1467 [nucl-ex] 10.1140/epjc/s10052-013-2617-1 PMC4371050 25814847

[780] ALICE Collaboration, B. Abelev et al., Coherent $$J/\psi $$ photoproduction in ultra-peripheral Pb–Pb collisions at $$\sqrt{s_{NN}}=2.76$$ TeV. Phys. Lett. B **718**, 1273–1283 (2013). arXiv:1209.3715 [nucl-ex]

[781] CMS Collaboration, Photoproduction of the coherent $$J/\psi $$ collisions at 2.76 TeV. CMS-PAS-HIN-12-009 (2014)

[782] ALICE Collaboration, K. Aamodt et al., The ALICE experiment at the CERN LHC. JINST **3**, S08002 (2008)

[783] CMS Collaboration, S. Chatrchyan et al., The CMS experiment at the CERN LHC. JINST **3**, S08004 (2008)

[784] Guzey V, Strikman M, Zhalov M (2014). Disentangling coherent and incoherent quasielastic $$\text{ J }/\psi $$ photoproduction on nuclei by neutron tagging in ultraperipheral ion collisions at the LHC. Eur. Phys. J. C.

[785] A. Adeluyi, C. Bertulani, Constraining gluon shadowing using photoproduction in ultraperipheral p-A and AA collisions. Phys. Rev. C **85**, 044904 (2012). arXiv:1201.0146 [nucl-th]

[786] ALICE Collaboration, M. Broz, Charmonium photoproduction in ultra-peripheral p–Pb and Pb–Pb collisions at the LHC with the ALICE experiment. arXiv:1409.6169 [hep-ex]

[787] H1 Collaboration, C. Adloff et al., Diffractive photoproduction of $${\psi \text{(2S) }}$$ mesons at HERA. Phys. Lett. B **541**, 251–264 (2002). arXiv:hep-ex/0205107 [hep-ex]

[788] CDF Collaboration, T. Aaltonen et al., Observation of exclusive charmonium production and gamma gamma $$\rightarrow $$$${\mu ^{+}\mu ^{-}}$$ in $${\text{ p }\overline{\text{ p }}}$$ collisions at $${\sqrt{s}}$$ = 1.96 TeV. Phys. Rev. Lett. **102**, 242001 (2009). arXiv:0902.1271 [hep-ex] 10.1103/PhysRevLett.102.242001 19658998

[789] LHCb Collaboration, R. Aaij et al., Exclusive $$J/\psi $$ TeV. J. Phys. G **40**, 045001 (2013). arXiv:1301.7084 [hep-ex]

[790] ALICE Collaboration, A. Lardeux, $$\text{ J }/\psi $$ = 2.76 TeV in the ALICE experiment. J. Phys. Conf. Ser. **446**, 012042 (2013)

[791] J. Nystrand, Photoproduction of quarkonium. https://indico.cern.ch/event/93174/ (2010) (first ReteQuarkonii workshop, 25–28th October 2010, Nantes)

[792] ALICE Collaboration, L. Massacrier, Low $${p_\text{ T }}$$ in Pb–Pb collisions at forward rapidity with the ALICE experiment. Poster presented in quark matter (2014)

[793] A. Adeluyi, T. Nguyen, Coherent photoproduction of $$\psi $$ and $$\Upsilon $$ mesons in ultraperipheral $${\text{ p--Pb }}$$ and $$\text{ Pb--Pb }$$ collisions at the CERN large hadron collider at $$\sqrt{s_{NN}}=5$$ TeV and $$\sqrt{s_{NN}}=2.76$$ TeV. Phys. Rev. C **87**, 027901 (2013). arXiv:1302.4288 [nucl-th]

[794] Cisek A, Schafer W, Szczurek A (2012). Exclusive coherent production of heavy vector mesons in nucleus-nucleus collisions at LHC. Phys. Rev. C.

[795] S.R. Klein, J. Nystrand, Photoproduction of quarkonium in proton–proton and nucleus–nucleus collisions. Phys. Rev. Lett. **92**, 142003 (2004). arXiv:hep-ph/0311164 [hep-ph] 10.1103/PhysRevLett.92.142003 15089531

[796] Lappi T, Mantysaari H (2011). Incoherent diffractive $$\text{ J }/\psi $$ -production in high energy nuclear DIS. Phys. Rev. C.

[797] Lappi T, Mantysaari H (2013). $$\text{ J }/\psi $$ production in ultraperipheral Pb+Pb and p+Pb collisions at LHC energies. Phys. Rev. C.

[798] Goncalves V, Machado M (2011). Vector meson production in coherent hadronic interactions: an update on predictions for RHIC and LHC. Phys. Rev. C.

[799] Ducati MBG, Griep M, Machado M (2013). Diffractive photoproduction of radially excited $${\psi \text{(2S) }}$$ mesons in photon-Pomeron reactions in $$\text{ Pb-Pb }$$ collisions at the CERN LHC. Phys. Rev. C.

[800] Rebyakova V, Strikman M, Zhalov M (2012). Coherent $$\rho $$ and $$\text{ J }/\psi $$ photoproduction in ultraperipheral processes with electromagnetic dissociation of heavy ions at RHIC and LHC. Phys. Lett. B.

[801] Ryskin M (1993). Diffractive $$\text{ J }/\psi $$ electroproduction in LLA QCD. Z. Phys. C.

[802] S. J. Brodsky, L. Frankfurt, J. Gunion, A. H. Mueller, M. Strikman, Diffractive leptoproduction of vector mesons in QCD. Phys. Rev. D **50**, 3134–3144 (1994). arXiv:hep-ph/9402283 [hep-ph] 10.1103/physrevd.50.3134 10017947

[803] Guzey V, Zhalov M (2013). Exclusive $$J/{\psi }$$ production in ultraperipheral collisions at the LHC: constrains on the gluon distributions in the proton and nuclei. JHEP.

[804] Martin A, Stirling W, Thorne R, Watt G (2009). Parton distributions for the LHC. Eur. Phys. J. C.

[805] Eskola KJ, Paukkunen H, Salgado CA (2008). An improved global analysis of nuclear parton distribution functions including RHIC data. JHEP.

[806] M. Hirai, S. Kumano, T.-H. Nagai, Nuclear parton distribution functions and their uncertainties. Phys. Rev. C **70**, 044905 (2004). arXiv:hep-ph/0404093 [hep-ph]

[807] Frankfurt L, Guzey V, Strikman M (2012). Leading twist nuclear shadowing phenomena in hard processes with nuclei. Phys. Rep..

[808] Martin A, Nockles C, Ryskin MG, Teubner T (2008). Small x gluon from exclusive $$\text{ J }/\psi $$ production. Phys. Lett. B.

[809] A. Shuvaev, K.J. Golec-Biernat, A.D. Martin, M. Ryskin, Off diagonal distributions fixed by diagonal partons at small x and xi. Phys. Rev. D **60**, 014015 (1999). arXiv:hep-ph/9902410 [hep-ph]

[810] Gribov V, Migdal AA (1968). The Pomeranchuk quasi-stable pole and diffraction scattering at ultrahigh-energy. Yad. Fiz..

[811] J. Bartels, K. J. Golec-Biernat, K. Peters, On the dipole picture in the nonforward direction, Acta Phys. Polon. B **34**, 3051–3068 (2003). arXiv:hep-ph/0301192 [hep-ph]

[812] H. Kowalski, D. Teaney, An impact parameter dipole saturation model. Phys. Rev. D **68**, 114005 (2003). arXiv:hep-ph/0304189 [hep-ph]

[813] J. Nemchik, N. N. Nikolaev, B. Zakharov, Scanning the BFKL pomeron in elastic production of vector mesons at HERA. Phys. Lett. B **341**, 228–237 (1994). arXiv:hep-ph/9405355 [hep-ph]

[814] J. Nemchik, N. N. Nikolaev, E. Predazzi, B. Zakharov, Color dipole phenomenology of diffractive electroproduction of light vector mesons at HERA, Z. Phys. C **75**, 71–87 (1997). arXiv:hep-ph/9605231 [hep-ph]

[815] E. Iancu, K. Itakura, S. Munier, Saturation and BFKL dynamics in the HERA data at small x. Phys. Lett. B **590**, 199–208 (2004). arXiv:hep-ph/0310338 [hep-ph]

[816] I. Balitsky, Operator expansion for high-energy scattering. Nucl. Phys. B **463**, 99–160 (1996). arXiv:hep-ph/9509348 [hep-ph]

[817] Y.V. Kovchegov, Small x F(2) structure function of a nucleus including multiple pomeron exchanges. Phys. Rev. D **60**, 034008 (1999). arXiv:hep-ph/9901281 [hep-ph]

[818] Y.V. Kovchegov, Unitarization of the BFKL pomeron on a nucleus. Phys. Rev. D **61**, 074018 (2000). arXiv:hep-ph/9905214 [hep-ph]

[819] H. Kowalski, L. Motyka, G. Watt, Exclusive diffractive processes at HERA within the dipole picture. Phys. Rev. D **74**, 074016 (2006). arXiv:hep-ph/0606272 [hep-ph]

[820] J. Nemchik, Wave function of 2S radially excited vector mesons from data for diffraction slope. Phys. Rev. D **63**, 074007 (2001). arXiv:hep-ph/0003245 [hep-ph]

[821] A. Deshpande, R. Milner, R. Venugopalan, W. Vogelsang, Study of the fundamental structure of matter with an electron-ion collider. Ann. Rev. Nucl. Part. Sci. **55**, 165–228 (2005). arXiv:hep-ph/0506148 [hep-ph]

[822] J. Dainton, M. Klein, P. Newman, E. Perez, F. Willeke, Deep inelastic electron-nucleon scattering at the LHC. JINST **1**, P10001 (2006). arXiv:hep-ex/0603016 [hep-ex]

[823] F. Bordry et al., The first long shutdown (LS1) for the LHC, CERN-ACC-2013-0084 (2013)

[824] L. Rossi, O. Brusing, High luminosity large hadron collider a description for the European Strategy Preparatory Group, CERN-ATS-2012-236 (2012)

[825] CMS Collaboration, S. Chatrchyan et al., Observation of a new boson at a mass of 125 GeV with the CMS experiment at the LHC. Phys. Lett. B **716**(1), 30–61 (2012). http://www.sciencedirect.com/science/article/pii/S0370269312008581

[826] ATLAS Collaboration, G. Aad et al., Observation of a new particle in the search for the Standard Model Higgs boson with the ATLAS detector at the LHC. Phys. Lett. B **716**(1), 1–29 (2012) . http://www.sciencedirect.com/science/article/pii/S037026931200857X

[827] ALICE Collaboration, B. Abelev et al., Upgrade of the ALICE experiment: letter of intent. J. Phys. G **41**, 087001 (2014)

[828] ALICE Collaboration, B. Abelev et al., Addendum of the Letter of intent for the upgrade of the ALICE experiment: the muon forward tracker, CERN-LHCC-2013-014 (2013)

[829] ALICE Collaboration, B. Abelev et al., Technical design report for the upgrade of the ALICE inner tracking system. J. Phys. G **41**, 087002 (2013)

[830] ALICE Collaboration, B. Abelev et al., Technical design report for the muon forward tracker, CERN-LHCC-2015-001 (2015)

[831] ALICE Collaboration, B. Abelev et al., Upgrade of the ALICE readout and trigger system, CERN-LHCC-2013-019 (2013)

[832] Andronic A, Beutler F, Braun-Munzinger P, Redlich K, Stachel J (2009). Statistical hadronization of heavy flavor quarks in elementary collisions: successes and failures. Phys. Lett. B.

[833] ATLAS Collaboration, M. Capeans et al., ATLAS insertable B-layer technical design report, CERN-LHCC-2010-013 (2010). https://cds.cern.ch/record/1291633/

[834] ATLAS Collaboration, ATLAS B-physics studies at increased LHC luminosity, potential for CP-violation measurement in the B$$^0_s\rightarrow \text{ J }/\psi \phi $$ decay, ATL-PHYS-PUB-2013-010 (2013). https://cds.cern.ch/record/1604429/

[835] ATLAS Collaboration, Letter of intent for the phase-II upgrade of the ATLAS experiment, CERN-LHCC-2012-022 (2012). https://cds.cern.ch/record/1502664/

[836] ATLAS Collaboration, Letter of intent for the phase-I upgrade of the ATLAS experiment, CERN-LHCC-2011-012 (2011). https://cds.cern.ch/record/1402470/

[837] (ATLAS) Collaboration, R. Bartoldus et al., Technical design report for the phase-I upgrade of the ATLAS TDAQ system, CERN-LHCC-2013-018 (2013). https://cds.cern.ch/record/1602235/

[838] CMS Collaboration, CMS technical design report for the level-1 trigger upgrade, CERN-LHCC-2013-011 (2013)

[839] CMS Collaboration, CMS technical design report for the pixel detector upgrade, CERN-LHCC-2012-016 (2012)

[840] CMS Collaboration, CMS technical design report for the phase 1 upgrade of the hadron calorimeter, CERN-LHCC-2012-015 (2012)

[841] CMS Collaboration, Projections for heavy ions with HL-LHC, CMS-PAS-FTR-13-025 (2013)

[842] CMS Collaboration, D. Contardo et al., CMS phase 2 upgrade: preliminary plan and cost estimate, CERN-RRB-2013-124 (2013). http://cds.cern.ch/record/1605208/

[843] LHCb Collaboration, Framework TDR for the LHCb upgrade: technical design report. Technical Report CERN-LHCC-2012-007. LHCb-TDR-12, CERN, Geneva (2012)

[844] LHCb Collaboration, LHCb trigger and online upgrade technical design report. Technical Report CERN-LHCC-2014-016. LHCB-TDR-016, CERN, Geneva (2014)

[845] LHCb Collaboration, LHCb VELO upgrade technical design report. Technical Report CERN-LHCC-2013-021. LHCB-TDR-013, CERN, Geneva (2013)

[846] LHCb Collaboration, LHCb tracker upgrade technical design report. Technical Report CERN-LHCC-2014-001. LHCB-TDR-015, CERN, Geneva (2014)

[847] LHCb Collaboration, LHCb PID upgrade technical design report. Technical Report CERN-LHCC-2013-022. LHCB-TDR-014, CERN, Geneva (2013)

[848] Y. Akiba, A. Angerami, H. Caines, A. Frawley, U. Heinz, et al., The hot QCD white paper: exploring the phases of QCD at RHIC and the LHC. arXiv:1502.02730 [nucl-ex]

[849] W. Fischer et al., RHIC Collider Projections, http://www.rhichome.bnl.gov/RHIC/Runs/RhicProjections.pdf (2014)

[850] A. Adare, S. Afanasiev, C. Aidala, N. Ajitanand, Y. Akiba, et al., An upgrade proposal from the PHENIX collaboration, arXiv:1501.06197 [nucl-ex]

[851] PHENIX Collaboration, C. Aidala et al., sPHENIX: an upgrade concept from the PHENIX collaboration, arXiv:1207.6378 [nucl-ex]

[852] STAR Collaboration, STAR, RHIC beam use request for runs 16 and 17. Technical report (2015). https://drupal.star.bnl.gov/STAR/starnotes/public/sn0625

[853] STAR Collaboration, STAR, RHIC beam use request for runs 14 and 15. Technical report (2013). https://indico.bnl.gov/conferenceDisplay.py?confId=632

[854] STAR Collaboration, STAR decadal plan. http://www.bnl.gov/npp/docs/STAR_Decadal_Plan_Final[1].pdf (2010)

[855] E598 Collaboration, J. Aubert et al., Experimental Observation of a Heavy Particle J, Phys. Rev. Lett. 33 (1974) 1404–1406

[856] Herb S, Hom D, Lederman L, Sens J, Snyder H (1977). Observation of a dimuon resonance at 9.5 GeV in 400 GeV proton–nucleus collisions. Phys. Rev. Lett..

[857] Armstrong T, Bettoni D, Bharadwaj V, Biino C, Borreani G (1992). Observation of the p wave singlet state of charmonium. Phys. Rev. Lett..

[858] Hoyer P, Vanttinen M, Sukhatme U (1990). Violation of factorization in charm hadroproduction. Phys. Lett. B.

[859] NA3 Collaboration, J. Badier et al., Evidence for $$\psi \psi $$. Phys. Lett. B **114**, 457 (1982)

[860] P. E. Reimer, Measuring the anti-quark sea asymmetry at high x: Fermilab P906, eConf C010630, E505 (2001)

[861] COMPASS Collaboration, C. Quintans, Future Drell–Yan measurements in COMPASS. J. Phys. Conf. Ser. **295**, 012163 (2011)

[862] Sivers DW (1990). Single spin production asymmetries from the hard scattering of point-like constituents. Phys. Rev. D.

[863] Ali Y, Staszel P (2013). Future vertex detector for open charm measurements with the NA61/SHINE experiment at the CERN SPS. Acta Phys. Polon. Suppl..

[864] B. Friman, C. Hohne, J. Knoll, S. Leupold, J. Randrup et al., The CBM physics book: compressed baryonic matter in laboratory experiments. Lect. Notes Phys. **814**, 1–980 (2011)

[865] Costantini F (1993). LHB: a fixed target experiment at LHC to measure CP violation in B mesons. Nucl. Instrum. Methods.

[866] Uggerhoj E, Uggerhj UI (2005). Strong crystalline fields: a possibility for extraction from the LHC. Nucl. Instrum. Methods.

[867] Brodsky S, Fleuret F, Hadjidakis C, Lansberg J (2013). Physics opportunities of a fixed-target experiment using the LHC beams. Phys. Rep..

[868] Lansberg J, Brodsky S, Fleuret F, Hadjidakis C (2012). Quarkonium physics at a fixed-target experiment using the LHC beams. Few Body Syst..

[869] J. Lansberg, V. Chambert, J. Didelez, B. Genolini, C. Hadjidakis, et al., A Fixed-Target ExpeRiment at the LHC (AFTER@LHC) : luminosities, target polarisation and a selection of physics studies, PoS QNP2012, 049 (2012). arXiv:1207.3507 [hep-ex]

[870] A. Rakotozafindrabe, R. Arnaldi, S. Brodsky, V. Chambert, J. Didelez, et al., Ultra-relativistic heavy-ion physics with AFTER@LHC. Nucl. Phys. A **904–905**, 957c–960c (2013). arXiv:1211.1294 [nucl-ex]

[871] Ferro-Luzzi M (2005). Proposal for an absolute luminosity determination in colliding beam experiments using vertex detection of beam–gas interactions. Nucl. Instrum. Methods.

[872] F. Arleo, F. Fleuret, E. Ferreiro, P.-B. Gossiaux, S. Peigne, Expression of interest for an experiment to study charm production with proton and heavy ion beams, CERN-SPSC-2012-031, SPSC-EOI-008 (2012)

[873] Kurepin A, Topilskaya N, Golubeva M (2011). Charmonium production in fixed-target experiments with SPS and LHC beams at CERN. Phys. Atom. Nucl..

